# Taxonomic revision and phylogenetic analysis of *Xylaria* in French Guiana, including 38 new species and one new combination

**DOI:** 10.1186/s40529-026-00496-0

**Published:** 2026-08-11

**Authors:** Yu-Ming Ju, Jacques Fournier, Huei-Mei Hsieh, Christian Lechat

**Affiliations:** 1 Institute of Plant and Microbial Biology, Academia Sinica, Nankang, Taipei, 115 Taiwan; 2 Las Muros, Rimont, 09420 France; 3 64 route de Chizé, Villiers-en-Bois, 79360 France

**Keywords:** Ascomycota, Fungal survey, Phylogeny, Sordariomycetes, Taxonomy, Xylariaceae

## Abstract

**Background:**

French Guiana is one of the earliest regions in the Neotropics where *Xylaria* species were extensively explored, notably by François Mathias René Leprieur during the 1830s, with many taxa subsequently described as new by Jean Pierre François Camille Montagne. Despite this long history and its clear biogeographical importance, the *Xylaria* diversity of French Guiana has remained insufficiently documented. Numerous species have been poorly defined, misidentified, or lack molecular data, and no modern, integrative synthesis combining morphology, typification, and phylogenetics has been available for the region.

**Results:**

Based on extensive recent field collections by two of us (JF and CL) and a re-examination of historical material, a total of 98 taxa of *Xylaria* are documented from French Guiana, with *X*. *cantareirensis* and *X*. *guadalupensis* additionally treated from Brazil and the French West Indies, respectively. Among these, 39 taxa are considered novel to science, including 38 newly described species and one newly recombined name. Multilocus phylogenetic analyses based on protein-coding loci support the separation of the analyzed taxa and clarify relationships among morphologically similar taxa. Of the newly described species, 37 were included in the phylogenetic analyses and are further confirmed as distinct. Several species closely related to *X*. *scruposa* are shown to constitute a species complex. Numerous historical names originally reported from French Guiana are re-evaluated through type studies, epitypifications, and critical morphological reassessments. These results highlight the remarkable *Xylaria* species richness of the region.

**Conclusion:**

This study demonstrates that French Guiana harbors a remarkably rich and complex *Xylaria* diversity that remains only partially resolved. The integration of detailed morphology and molecular phylogenetics reveals a much higher level of diversity than previously recognized and exposes significant taxonomic uncertainty in several species complexes. These findings underscore the need for continued sampling, especially of fresh material suitable for culturing, sequencing, and emphasize the importance of French Guiana as a key region for understanding the taxonomy, evolution, and biogeography of *Xylaria* in the Neotropics.

**Supplementary information:**

The online version contains supplementary material available at 10.1186/s40529-026-00496-0.

## Background

Since the early days of descriptive mycology, the pyrenomycetous Ascomycota currently classified in the genus *Xylaria* Hill ex Schrank have attracted the attention of naturalists (Pfister 2008). This interest is undoubtedly due to their relatively large size, their sometimes surprising shapes that are often striking in their variability, and their attractive appearance almost always linked to a somewhat diabolical, even disturbing look. When choosing a particular fungal group for study, field mycologists often acknowledge being guided by aesthetic or, at least, subjective considerations. This was very likely the case for the naturalist F. M. R. Leprieur (JSTOR), a naval pharmacist stationed in French Guiana in the 1830s, a botanist in the broad sense, and the first to collect numerous Xylariaceae in this region, particularly those now classified in *Camillea* Fr., *Hypoxylon* Bull. and *Xylaria*. His collections were sent to J. P. F. C. Montagne (JSTOR), an eminent botanist in Paris, who studied them and subsequently published an impressive number of new species (Montagne 1840, 1855). In his first publication, Montagne (1840) cited 21 species referable to *Xylaria*, of which 16 are currently accepted, including 12 newly described species—*X*. *collabens* (Mont.) Fr. *X*. *comosa* (Mont.) Fr., *X*. *cubensis* (Mont.) Fr., *X*. *enterogena* (Mont.) Fr., *X. grammica* (Mont.) Fr., *X*. *guyanensis* (Mont.) Fr., *X*. *heliscus* (Mont.) J. D. Rogers & Y.-M. Ju, *X*. *hyperythra* (Mont.) Fr., *X*. *ianthinovelutina* (Mont.) Fr., *X*. *microceras* (Mont.) Fr., *X*. *rhizocola* (Mont.) Fr., and *X*. *rhizomorpha* (Mont.) Mont.—as well as four previously known species—*X*. *globosa* (Spreng.) Mont., *X*. *gracillima* (Fr.) Fr., *X*. *multiplex* (Kunze) Fr., and *X*. *scruposa* (Fr.) Fr. In his subsequent publication, Montagne (1855) cited 40 species referable to *Xylaria*, of which 29 are currently accepted, including 10 newly described species—*X*. *aenea* Mont., *X*. *apeibae* Mont., *X*. *aristata* Mont., *X*. *axifera* Mont., *X*. *coccophora* Mont., *X*. *columnifera* Mont., *X*. *myosurus* Mont., *X*. *phyllocharis* Mont., *X*. *rhytidophloea* Mont., and *X*. *ruginosa* Mont.—as well as three previously known species—*X*. *cylindrica* Lév. (as *X*. *berkeleyi* Mont.), *X*. *kegeliana* (Lév.) Fr., and *X*. *telfairii* (Berk.) Sacc. (as *X*. *tabacina* Kickx f.)—in addition to the 16 species already reported in 1840. These two works documented 29 distinct *Xylaria* species from French Guiana, including 22 newly described species. One might have expected that such findings would have prompted further taxonomic investigation, but nearly two centuries would pass before such research was resumed.

Exploring and investigating the diversity suggested by these early results was the first step of this study. Our fieldwork was initiated by CL between 2007 and 2010 during two-week expeditions to several regions of French Guiana, including the areas around Cayenne, Régina, Roura, Saül, and Sinnamary. It was then continued by CL and JF in 2012 at the Nouragues Natural Reserve (Régina) and the Paracou Field Center (Sinnamary). The final stage consisted of two one-week expeditions to Saül, within the French Guiana Amazonian Park, in 2018 and 2019, with JF accompanied by a few colleagues, followed by a final field trip of JF and CL in 2021 for five days near Roura and ten days in Saül.

Our project was inspired by the pioneering work of Leprieur and Montagne and was quickly stimulated by the remarkable abundance of diverse and attractive species encountered in the field, many of which could not be readily assigned to known species. To understand the reasons for this exuberant biological richness, one must consider the vast extent of the Guianese forest, covering approximately 8 million hectares, remaining relatively undisturbed by human activities, and characterized by extraordinary botanical richness and a humid tropical climate. Together, these factors contribute greatly to its outstanding biodiversity.

The abundant material (646 specimens in good condition) collected over these years was systematically examined morphologically by JF, who proposed provisional identifications or suggested possible taxonomic affinities before sending the material to YMJ and HMH in Taiwan for confirmation and cultivation, with the aim of conducting phylogenetic analyses. Over time, it became apparent that phylogenetic studies based on morphologically well-characterized collections could enable significant progress in the taxonomy of *Xylaria* by improving often poorly defined species concepts and facilitating the designation of epitypes. Finally, we considered the establishment of a database of ITS sequences based on correctly identified material deposited in a public herbarium to be essential for future studies on endophytes of neotropical plants. Among other applications, such a resource would help clarify the ecology and distribution of the *Xylaria* species documented in this work.

## Methods

### Fungal materials and observations

Stromata of *Xylaria* species were collected in French Guiana. Cultures were obtained by placing stromatal tissue and/or ascospore masses on malt extract agar prepared from scratch (Kenerley and Rogers 1976), These cultures were used for DNA extraction and sequencing.

Microscopic observations were conducted on dry material rehydrated in water. Measurements of asci and ascospores were made in water, and ascospores measurements processed using the software Piximetre 5.2 (http://ach.log.free.fr/Piximetre/). In the formula generated by this software, values in parentheses represent the extreme values (20%) that are excluded from the calculation; N represents the number of ascospores measured; Q the quotient length/width; Me the mean values of length × width; and Qe the mean value of the length/width quotient. The amyloid reaction of the ascus apical apparatus was tested by adding a drop of Melzer’s reagent to a water mount of centrum contents. Microscopic observations of the asci and the ascogenous hyphae were carried out after 1 min in 1% SDS, followed by mounting in blue or black Waterman inks, chlorazol black, black Pelikan ink, aqueous nigrosin, Congo red followed by 3% KOH, or India ink. When ascospore germ slit morphology was difficult to observe, ascospores were either mounted in PVA-lactophenol and examined after 48 h of incubation or directly observed in heated chloral-lactophenol. Anamorphic structures were observed in 1% SDS. Measurements of perithecia, asci, and ascal apical apparati are recorded as height × width.

Photomacrographs were taken with a Nikon Coolpix 995 digital camera, either mounted directly on a stand or, at higher magnifications, attached to the eyepiece of an Olympus SZ60 stereomicroscope using a 30-mm-diam adapter. Photomicrographs were taken with the same camera mounted on the trinocular port of a Leitz Orthoplan microscope fitted with Planapo objectives. Digital images were processed using Adobe Photoshop Elements 10, and figure plates were assembled with the same software.

Specimens and cultures were deposited at Herbarium of Biodiversity Research Museum, Academia Sinica, Taiwan (HAST), and Bioresource Collection and Research Center, Taiwan (BCRC), respectively.

### DNA extraction, PCR amplification, and sequencing

Sequences of the loci encoding β-tubulin (β-TUB) and α-actin (α-ACT) were obtained from the studied *Xylaria* species following Hsieh et al. (2005), while those of the loci for the second largest subunit of RNA polymerase II (RPB2) were obtained following Hsieh et al. (2010). Sequences of nuclear rDNA internal transcribed spacers (ITS = ITS1-5.8 S-ITS2) were obtained following Hsieh et al. (2009).

The combined RPB2-TUB-ACT dataset (Additional file 1), comprising concatenated sequences of α-ACT, RPB2, and β-TUB of the taxa listed in Table 1, was aligned using Clustal X 1.81 (Thompson et al. 1997) with “gap penalty” set to 10 and “gap extension penalty” set to 0.2, and was subsequently manually refined. The dataset was subjected to maximum likelihood (ML) and Bayesian inference (BI) analyses. *Biscogniauxia arima* F. San Martín, Y.-M. Ju & J. D. Rogers was selected as the outgroup. Models of evolution for both ML and BI analyses were determined using MrModeltest 2.4 (Nylander 2004). Consensus trees were viewed using FigTree ver. 1.4.4 (http://tree.bio.ed.ac.uk/software/figtree/).

**Table 1:** List of sequences of taxa and isolates included in the ML and BI analyses. Concatenated sequences of *β-tubulin, α-actin*, and *RPB2* lci were analyzed and are presented in Fig. 1. Sequences in boldface were generated in this study

Taxon	Origin	Collecting data	GenBank accession number			
			β-tubulin	α-actin	RPB2	ITS
*Amphirosellinia fushanensis* Y.-M. Ju et al.	Taiwan	From HOLOTYPE (Ju et al. 2004)	GQ495950	GQ452360	GQ848339	GU339496
*Am. nigrospora* Y.-M. Ju et al.	Taiwan	From HOLOTYPE (Ju et al. 2004)	GQ495951	GQ452361	GQ848340	GU322457
*Astrocystis bambusae* (Henn.) Læssøe & Spooner	Taiwan	*Ju & Hsieh 89021904* (Hsieh et al. 2010)	GQ495942	GQ449239	GQ844836	GU322449
*As. mirabilis* Berk. & Broome	Taiwan	*Ju & Hsieh 94070803* (Hsieh et al. 2010)	GQ495941	GQ449238	GQ844835	GU322448
*As. sublimbata* (Durieu & Mont.) G. C. Hughes	Taiwan	*Ju & Hsieh 89032207* (Hsieh et al. 2010)	GQ495940	GQ449236	GQ844834	GU322447
*Biscogniauxia arima* San Martín et al.	Mexico	*YMJ122* from ISOTYPE (Ju et al. 1998; Hsieh et al. 2005)	AY951672	AY951784	GQ304736	EF026150
*Discoxylaria myrmecophila* J. C. Lindq. & J. E. Wright	Mexico	*YMJ169* from *Moreno 713* (Rogers et al. 1995)	GQ487710	GQ438747	GQ844819	GU322433
*Entoleuca mammata* (Wahlenb.) J. D. Rogers & Y.-M. Ju	France	*YMJ100* from *Candoussau, F. 5254* (Hsieh et al. 2010)	GQ470230	GQ398230	GQ844782	GU300072
*Euepixylon sphaeriostomum* (Schwein.) Y.-M. Ju & J. D. Rogers	USA	*YMJ261* from *Huhndorf, S. M. 1447* (Hsieh et al. 2010)	GQ470224	GQ389696	GQ844774	GU292821
*Kretzschmaria clavus* (Fr.) Sacc.	French Guiana	*YMJ114* from *Huhndorf 803* (Rogers and Ju 1998; Ju et al. 2007)	EF025611	EF025596	GQ844789	EF026126
*K. guyanensis* J. D. Rogers & Y.-M. Ju	Taiwan	*Ju & Hsieh 89062903* (Hsieh et al. 2010)	GQ478214	GQ408901	GQ844792	GU300079
*K. lucidula* (Mont.) Dennis	French Guiana	*YMJ112* from *Huhndorf 677* (Rogers and Ju 1998; Ju et al. 2007)	EF025610	EF025595	GQ844790	EF026125
*K. megalospora* J. D. Rogers & Y.-M. Ju	Malaysia	*YMJ229* from *Whalley, M. FH 64–97* (Ju et al. 2007)	EF025609	EF025594	GQ844791	EF026124
*K. neocaledonica* (Har. & Pat.) J. D. Rogers & Y.-M. Ju	Taiwan	*Guu, J.-R. 94031003* (Hsieh et al. 2010)	GQ478213	GQ398236	GQ844788	GU300078
*K. pavimentosa* (Ces.) P. Martin	Taiwan	*YMJ109* from *Wang 511* (Rogers and Ju 1998)	GQ478212	GQ398235	GQ844787	GU300077
*K. sandvicensis* (Reichardt) J. D. Rogers & Y.-M. Ju	USA, Hawaiian Islands	*YMJ113* from *Rogers/4 Jan 1996* (Rogers and Ju 1998)	GQ478211	GQ398234	GQ844786	GU300076
*Nemania abortiva* J. D. Rogers et al.	USA, Hawaiian Islands	*YMJ467* from HOLOTYPE (Rogers et al. 2006)	GQ470219	GQ374123	GQ844768	GU292816
*N. beaumontii* (Berk. & M. A. Curtis) Y.-M. Ju & J. D. Rogers	French West Indies	*YMJ405* from *Lechat, C. CLL2039* (Hsieh et al. 2010; Fournier et al. 2018b)	GQ470222	GQ389694	GQ844772	GU292819
*N. bipapillata* (Berk. & M. A. Curtis) Pouzar	Taiwan	*Ju & Hsieh 90080610* (Hsieh et al. 2010)	GQ470221	GQ389693	GQ844771	GU292818
*N. diffusa* (Sowerby) S. F. Gray	Taiwan	*Ju & Hsieh 91020401* (Hsieh et al. 2010)	GQ470220	GQ389692	GQ844769	GU292817
*N. illita* (Schwein.) Pouzar	USA	*YMJ236* from *Tsai, S.-J.* (Ju et al. 2007)	EF025608	EF025593	GQ844770	EF026122
*N. macrocarpa* Y.-M. Ju & J. D. Rogers	USA, Hawaiian Islands	*YMJ265* from HOLOTYPE (Ju and Rogers 2002)	GQ470226	GQ389698	GQ844776	GU292823
*N. maritima* Y.-M. Ju & J. D. Rogers	Taiwan	From HOLOTYPE (Ju and Rogers 2002)	GQ470225	GQ389697	GQ844775	GU292822
*N. primolutea* Y.-M. Ju et al.	Taiwan	From HOLOTYPE (Ju et al. 2005, 2007)	EF025607	EF025592	GQ844767	EF026121
*N. serpens* (Pers.) S. F. Gray (Barron isolate)	Canada	*YMJ235* from *Barron, G.*, as *Hypoxylon* in Petrini and Rogers (1986)	GQ470223	GQ389695	GQ844773	GU292820
*Podosordaria mexicana* Ellis & Holw.	Mexico	*YMJ176* from *San Martín 6013T* (Rogers et al. 1998)	GQ844840	GQ455451	GQ853039	GU324762
*Pod. muli* J. D. Rogers et al.	Mexico	*YMJ167* from HOLOTYPE (Rogers et al. 1998)	GQ844839	GQ455450	GQ853038	GU324761
*Poronia ingii* (J. D. Rogers & Læssøe) J. D. Rogers et al.	Spain, Canary Islands	*YMJ1351* from *Negrín, R.* (Ju et al. 2025)	PV207617	PV207613	PV207621	PV098401
*Por. leporina* Ellis & Everh.	Taiwan	*YMJ2269* from *Ju, Y.-M. & Hsieh, H.-M. 100052001* (Ju et al. 2025)	PV207618	PV207614	PV207622	PV098402
*Por. phosphorea* (Berk.) Y.-M. Ju et al.	USA	*YMJ2230* from *Paxton, C. & Paxton, K.* (Ju et al. 2025)	PV207620	PV207616	PV207624	PV098404
*Por. pileiformis* (Berk.) Fr.	Taiwan	ATCC MYA-1222 (Ju and Rogers 2001)	GQ502720	GQ455449	GQ853037	GU324760
*Rosellinia buxi* Fabre	France	*YMJ99* from *Candoussau, F.* (Hsieh et al. 2010)	GQ470228	GQ398228	GQ844780	GU300070
*R. lamprostoma* Syd. & P. Syd.	Taiwan	*Ju & Hsieh 89112602* (Ju et al. 2007)	EF025604	EF025589	GQ844778	EF026118
*R. merrillii* Syd. & P. Syd.	Taiwan	*Ju & Hsieh 89112601* (Hsieh et al. 2010)	GQ470229	GQ398229	GQ844781	GU300071
*R. necatrix* (R. Hartig) Berl.	Taiwan	*Ju & Hsieh 89062904* (Ju et al. 2007)	EF025603	EF025588	GQ844779	EF026117
*R. sanctaecruciana* Ferd. & Winge	Taiwan	*Ju & Hsieh 90072903* (Hsieh et al. 2010)	GQ470227	GQ389699	GQ844777	GU292824
*Stilbohypoxylon elaeidicola* (Henn.) L. E. Petrini	French Guiana	*YMJ173* from *Huhndorf 928* (Ju et al. 2007), as *S. moelleri* in Rogers and Ju (1997)	EF025616	EF025601	GQ844826	EF026148
*S. elaeidicola* (Henn.) L. E. Petrini	Taiwan	*Ju & Hsieh 94082615* (Hsieh et al. 2010)	GQ495933	GQ438754	GQ844827	GU322440
*S. quisquiliarum* (Mont.) J. D. Rogers & Y.-M. Ju	French Guiana	*YMJ172* from *Huhndorf 940* (Rogers and Ju 1997; Ju et al. 2007)	EF025605	EF025590	GQ853020	EF026119
*S. quisquiliarum* (Mont.) J. D. Rogers & Y.-M. Ju	Taiwan	*Ju & Hsieh 89091608* (Ju et al. 2007)	EF025606	EF025591	GQ853021	EF026120
*Xylaria acuminatilongissima* Y.-M. Ju & H.-M. Hsieh	Taiwan	*YMJ623* from HOLOTYPE (Ju and Hsieh 2007)	GQ502711	GQ853046	GQ853028	EU178738
*X. adscendens* (Fr.) Fr.	French West Indies	*YMJ570* from *Lechat, C. CLL5347* (Hsieh et al. 2010)	GQ487708	GQ438745	GQ844817	GU300101
*X. adscendens* (Fr.) Fr.	Thailand	*YMJ865* from *Bandoni, R. J., Bandoni, A. A. & Flegel, T. W. 12017* (Hsieh et al. 2010)	GQ487709	GQ438746	GQ844818	GU322432
*X. adscendens* (Fr.) Fr.	French Guiana	*YMJ3153* from *Fournier, J. GYJF21016* (this study)	**PZ393366**	**PZ370558**	**PZ393526**	**PZ373809**
*X. aenea* Mont.	Venezuela	*YMJ1546* from stroma of *Rossman, A. 1849–434* (Ju and Hsieh 2014)	**PZ393367**	**PZ370559**	**PZ393527**	**PZ373810**
*X. aethiopica* J. Fourn. et al.	Ethiopia	BCRC FU31033 from ISOTYPE (Fournier et al. 2018a)	MH785221	MH785223	MH785222	MH790445
*X. alboareolata* Y.-M. Ju & J.D. Rogers	French West Indies	*YMJ543* from *Chabrol, J. CLL5372* (Fournier et al. 2018c), as *X. areolata* in Hsieh et al. (2010)	GQ478215	GQ408902	GQ844793	GU300080
*X. allantoidea* (Berk.) Fr.	Taiwan	*Ju & Hsieh 94042903* (Hsieh et al. 2010)	GQ502692	GQ452377	GQ848356	GU324743
*X. amphithele* San Martín & J. D. Rogers	French West Indies	*YMJ529* from *Lechat, C. CLL5352* (Hsieh et al. 2010)	GQ478218	GQ408905	GQ844796	GU300083
*X. angulosa* J. D. Rogers et al.	Taiwan	*YMJ3267* from Okane et al. (2025)	PQ605184	PQ605194	PQ605189	PQ582213
*X. apeibae* Mont.	French Guiana	*YMJ1926* from *Fournier, J. GYJF19071* (this study)	**PZ393368**	**PZ370560**	**PZ393528**	**PZ373811**
*X. apeibae* Mont.	French Guiana	*YMJ1867* from *Fournier, J. GYJF18080* (this study)	**PZ393369**	**PZ370561**	**PZ393529**	**PZ373812**
*X. apoda* (Berk. & Broome) J. D. Rogers & Y.-M. Ju	Taiwan	*Ju & Hsieh 90080804* (Hsieh et al. 2010)	GQ495930	GQ438751	GQ844823	GU322437
*X. arbuscula* Sacc. var. *arbuscula*	Taiwan	*Ju & Hsieh 89041211* (Hsieh et al. 2010)	GQ478226	GQ421286	GQ844805	GU300090
*X. arbuscula* var. *plenofissura* Y.-M. Ju & S.-S. Tzean	Taiwan	*Ju & Hsieh 93082814* (Hsieh et al. 2010)	GQ478225	GQ421285	GQ844804	GU339495
*X. aristata* Mont. var. *aristata*	Taiwan	*Ju & Hsieh 90071613*, as *X. sicula* f. *major* in Hsieh et al. (2010)	GQ478216	GQ408903	GQ844794	GU300081
*X. aristata* Mont. var. *aristata*	Indonesia	*YMJ1823* (Ju and Hsieh 2023)	**PZ393370**	**PZ370562**	**PZ393530**	**PZ373813**
*X. asperula* (Starb.) J. Fourn. et al.	French Guiana	*YMJ1925* from *Fournier, J. GYJF19049* (this study)	**PZ393371**	**PZ370563**	**PZ393531**	**PZ373814**
*X. asperula* (Starb.) J. Fourn. et al.	French Guiana	*YMJ1860* from *Fournier, J. GYJF18006* (this study)	**PZ393372**	**PZ370564**	**PZ393532**	**PZ373815**
*X. atractoidosperma* J. Fourn. et al.	French Guiana	*YMJ1930* from *Fournier, J. GYJF19117* (this study)	**PZ393373**	**PZ370565**	**PZ393533**	**PZ373816**
*X. atractoidosperma* J. Fourn. et al.	French Guiana	*YMJ1933* from *Fournier, J. GYJF19131* (this study)	**PZ393374**	**PZ370566**	**PZ393534**	**PZ373817**
*X. atrodivaricata* Y.-M. Ju & H.-M. Hsieh	Taiwan	*YMJ615* from HOLOTYPE (Ju and Hsieh 2007)	GQ502713	GQ853048	GQ853030	EU178739
*X. atrosphaerica* (Cooke & Massee) Callan & J. D. Rogers	Taiwan	*Ju & Hsieh 91111214* (Hsieh et al. 2010)	GQ495953	GQ452363	GQ848342	GU322459
*X. axifera* Mont.	French Guiana	*YMJ3156* from *Fournier, J. GYJF21028* (this study)	**PZ393375**	**PZ370567**	**PZ393535**	**PZ373818**
*X. badia* Pat.	Taiwan	*Ju & Hsieh 95070101* (Hsieh et al. 2010)	GQ495939	GQ449235	GQ844833	GU322446
*X. bambusicola* Y.-M. Ju & J. D. Rogers	Taiwan	*YMJ205* from HOLOTYPE (Ju and Rogers 1999; Hsieh et al. 2005)	AY951762	AY951873	GQ844802	EF026123
*X. bambusicola* Y.-M. Ju & J. D. Rogers	Thailand	*YMJ162* from *Bandoni, R. J. & A. A. et al.* (Hsieh et al. 2010)	GQ478223	GQ408910	GQ844801	GU300088
*X. berteri* (Mont.) Cooke	USA, Hawaiian Islands	*YMJ256* from *Rogers, J. D. K-1* (Hsieh et al. 2010)	GQ502698	GQ455442	GQ848363	GU324750
*X. berteri* (Mont.) Cooke	Taiwan	*Ju & Hsieh 90112623* (Hsieh et al. 2005)	AY951763	AY951874	GQ848362	GU324749
*X. botuliformis* Rehm	Taiwan	*YMJ2434* from EPITYPE (Ju and Hsieh 2019)	MN095400	MN095398	MN095399	MN089652
*X. brevifurcata* Y.-M. Ju & H.-M. Hsieh	Taiwan	*YMJ1381* from HOLOTYPE (Ju et al. 2023)	OQ818432	OQ818372	OQ851526	OQ845482
*X. brunneovinosa* Y.-M. Ju & H.-M. Hsieh	Taiwan	*YMJ720* from HOLOTYPE (Ju and Hsieh 2007)	GQ502706	GQ853041	GQ853023	EU179862
*X. bubalina* J. Fourn. et al.	French Guiana	*YMJ1092* from *Lechat, C. CLL9076* (this study)	**PZ393376**	**PZ370568**	**PZ393536**	**PZ373819**
*X. candelabroides* J. Fourn. et al.	French Guiana	*YMJ3258* from *Fournier, J. GYJF21425* (this study)	**PZ393377**	**PZ370569**	**PZ393537**	**PZ373820**
*X. castorea* Berk.	New Zealand	*YMJ600* from *Samuels 85–75* (Hsieh et al. 2010)	GQ502703	GQ455447	GQ853018	GU324751
*X.* cf. *castorea* Berk.	Taiwan	*Ju & Hsieh 91092303* (Hsieh et al. 2010)	GQ502704	GQ455448	GQ853019	GU324752
*X. chaiyaphumensis* Wangsawat et al.	Thailand	*SWUF17-49.2* from HOLOTYPE (Wangsawat et al. 2021)	OQ845434	OQ845426	OQ851586	MT622775
*X. chiuyuanii* Y.-M. Ju et al.	Taiwan	*YMJ3271* from HOLOTYPE (Hsieh et al. 2024)	PP818666	PP818667	PP818665	PP820520
*X. cirrata* Pat.	Taiwan	*YMJ664* from EPITYPE (Ju and Hsieh 2007)	GQ502707	GQ853042	GQ853024	EU179863
*X. coccophora* Mont.	French Guiana	*YMJ786* from *Lechat, C. CLL7056* (Hsieh et al. 2010)	GQ487701	GQ421289	GQ844809	GU300093
*X. coccophora* Mont.	French Guiana	*YMJ3217* from *Fournier, J. GYJF21236* (this study)	**PZ393378**	**PZ370570**	**PZ393538**	**PZ373821**
*X. collabens* (Mont.) Fr.	French West Indies	*YMJ475* from *Lechat, C. CLL2146* (Hsieh et al. 2010; Fournier et al. 2019)	GQ478209	GQ398232	GQ844784	GU300074
*X. columnifera* Mont.	French Guiana	*YMJ3154* from *Fournier, J. GYJF21018* (this study)	**PZ393379**	**PZ370571**	**PZ393539**	**PZ373822**
*X. comosa* (Mont.) Fr.	French Guiana	*YMJ3187* from *Fournier, J. GYJF21129* (this study)	**PZ393380**	**PZ370572**	**PZ393540**	**PZ373823**
*X. comosa* (Mont.) Fr.	French Guiana	*YMJ3224* from *Fournier, J. GYJF21253* (this study)	**PZ393381**	**PZ370573**	**PZ393541**	**PZ373824**
*X. comosoides* Læssøe	French Guiana	*YMJ3250* from *Thonnel, A. GYJF21373* (this study)	**PZ393382**	**PZ370574**	**PZ393542**	**PZ373825**
*X. confragosa* J. Fourn. et al.	French Guiana	*YMJ1935* from *Corriol, G. GYJF19139* (this study)	**PZ393383**	**PZ370575**	**PZ393543**	**PZ373826**
*X. coprinicola* Y.-M. Ju et al.	China	*YMJ1145* from HOLOTYPE (Ju et al. 2011)	HM585018	HM585017	HM585019	HM585020
*X. corniformis* (Fr.) Fr.	Czech Republic	*YMJ1419* from stroma of *Beran, M. JF11006* (Fournier 2014)	**PZ393384**	**PZ370576**	**PZ393544**	**PZ373827**
*X. corriolii* J. Fourn. et al.	French Guiana	*YMJ1937* from *Fournier, J. GYJF19151* (this study)	**PZ393385**	**PZ370577**	**PZ393545**	**PZ373828**
*X. corriolii* J. Fourn. et al.	French Guiana	*YMJ3212* from *Fournier, J. GYJF21209* (this study)	**PZ393386**	**PZ370578**	**PZ393546**	**PZ373829**
*X. cranioides* (Sacc. & Paol.) Dennis	Taiwan	*YMJ226* from *Wen 712* (Ju and Rogers 2001)	GQ478210	GQ398233	GQ844785	GU300075
*X. crassipes* J. Fourn. et al.	French Guiana	*YMJ1086* from *Lechat, C. CLL9022* (this study)	**PZ393387**	**PZ370579**	**PZ393547**	**PZ373830**
*X. crozonensis* P. Leroy & Mornand	France	*YMJ398* from *Mornand, F. JF04151* (Hsieh et al. 2010)	GQ502697	GQ455441	GQ848361	GU324748
*X. crustulata* J. Fourn. et al.	French Guiana	*YMJ1920* from *Fournier, J. GYJF19024* (this study)	**PZ393388**	**PZ370580**	**PZ393548**	**PZ373831**
*X. crustulata* J. Fourn. et al.	French Guiana	*YMJ3204* from *Fournier, J. GYJF21182* (this study)	**PZ393389**	**PZ370581**	**PZ393549**	**PZ373832**
*X. cubensis* (Mont.) Fr.	French West Indies	*YMJ419* from *Lechat, C. CLL2179* (Fournier et al. 2019), as *X*. *laevis* in Hsieh et al. (2010)	GQ502695	GQ455439	GQ848359	GU324746
*X. cubensis* (Mont.) Fr.	Taiwan	*Ju & Hsieh 95072910*, as *X*. *laevis* in Hsieh et al. (2010)	GQ502696	GQ455440	GQ848360	GU324747
*X. cubensis* (Mont.) Fr.	French Guiana	*YMJ3244* from *Fournier, J. GYJF21348* (this study)	**PZ393390**	**PZ370582**	**PZ393550**	**PZ373833**
*X. cubensis* (Mont.) Fr.	French Guiana	*YMJ1864* from *Fournier, J. GYJF18072* (this study)	**PZ393391**	**PZ370583**	**PZ393551**	**PZ373834**
*X. culleniae* Berk. & Broome	Thailand	*YMJ189* from *Whalley, M. F.NH9* (Hsieh et al. 2010)	GQ495935	GQ438756	GQ844829	GU322442
*X. cuneata* C. G. Lloyd	French West Indies	*YMJ495* from *Lechat, C. CLL5131* (Fournier et al. 2019), as *X*. *montagnei* Hamme & Guerrero in Hsieh et al. (2010)	GQ495948	GQ449244	GQ848337	GU322455
*X. cuneata* C. G. Lloyd	French Guiana	*YMJ3242* from *Fournier, J. GYJF21327-2* (this study)	**PZ393392**	**PZ370584**	**PZ393552**	**PZ373835**
*X. cuneata* C. G. Lloyd	French Guiana	*YMJ3253* from *Fournier, J. GYJF21381* (this study)	**PZ393393**	**PZ370585**	**PZ393553**	**PZ373836**
*X. cuneata* C. G. Lloyd	French Guiana	*YMJ3254* from *Fournier, J. GYJF21393* (this study)	**PZ393394**	**PZ370586**	**PZ393554**	**PZ373837**
*X. cuneata* C. G. Lloyd	French Guiana	*YMJ3210* from *Fournier, J. GYJF21207* (this study)	**PZ393395**	**PZ370587**	**PZ393555**	**PZ373838**
*X. cuneata* C. G. Lloyd	French Guiana	*YMJ1863* from *Fournier, J. GYJF18149* (this study)	**PZ393396**	**PZ370588**	**PZ393556**	**PZ373839**
*X. curta* Fr.	French West Indies	*YMJ494* from *Lechat, C. CLL5044* (Hsieh et al. 2010)	GQ495937	GQ449233	GQ844831	GU322444
*X. curta* Fr.	Taiwan	*Ju & Hsieh 92092022* (Hsieh et al. 2010)	GQ495936	GQ438757	GQ844830	GU322443
*X. curta* Fr.	French Guiana	*YMJ3185* from *Fournier, J. GYJF21123* (this study)	**PZ393397**	**PZ370589**	**PZ393557**	**PZ373840**
*X. cylindrica* Ces.	French West Indies	*YMJ367* from *Lechat, C. CLL0928* (Hsieh et al. 2010)	GQ470231	GQ398231	GQ844783	GU300073
*X. cylindrica* Ces.	French Guiana	*YMJ3182* from *Fournier, J. GYJF21114* (this study)	**PZ393398**	**PZ370590**	**PZ393558**	**PZ373841**
*X. depressa* J. Fourn. et al.	French Guiana	*YMJ1850* from *Fournier, J. GYJF18041* (this study)	**PZ393399**	**PZ370591**	**PZ393559**	**PZ373842**
*X. digitata* (L.) Grev.	Ukraine	*YMJ919* from *Prilutsky, O.* (Hsieh et al. 2010)	GQ495949	GQ449245	GQ848338	GU322456
*X. discolor* (Berk. & Broome) Y.-M. Ju et al.	Portugal	BCRC 34912 from EPITYPE (Ju et al. 2012)	JQ087414	JQ087408	JQ087411	JQ087405
*X. enterogena* (Mont.) Fr.	French Guiana	*YMJ785* from *Lechat, C. CLL7043* (Hsieh et al. 2010)	GQ502685	GQ452370	GQ848349	GU324736
*X. enterogena* (Mont.) Fr.	French Guiana	*YMJ3241* from *Fournier, J. GYJF21324* (this study)	**PZ393400**	**PZ370592**	**PZ393560**	**PZ373843**
*X. escharoidea* (Berk.) Fr.	Taiwan	*YMJ658* from EPITYPE (Ju and Hsieh 2007)	GQ502709	GQ853044	GQ853026	EU179864
*X. exechostoma* J. Fourn. et al.	French Guiana	*YMJ3223* from *Fournier, J. GYJF21251* (this study)	**PZ393401**	**PZ370593**	**PZ393561**	**PZ373844**
*X. feejeensis* (Berk.) Fr.	French West Indies	*YMJ565* from *Lechat, C. CLL5653* (Hsieh et al. 2010)	GQ495945	GQ449241	GQ848334	GU322452
*X. feejeensis* (Berk.) Fr.	Taiwan	*Ju & Hsieh 92092013* (Hsieh et al. 2010)	GQ495947	GQ449243	GQ848336	GU322454
*X. feejeensis* (Berk.) Fr.	Thailand	*YMJ180* from *Whalley, M. UN515* (Hsieh et al. 2010)	GQ495946	GQ449242	GQ848335	GU322453
*X. fimbriata* C. G. Lloyd	French West Indies	*YMJ491* from *Lechat, C. CLL5010* (Hsieh et al. 2010)	GQ502705	GQ853040	GQ853022	GU324753
*X. flabelliformis* (Schwein.) Fr.	USA	*YMJ860* from *Rogers, J. D.*, as *X*. *cubensis* in Hsieh et al. (2010)	GQ502700	GQ455444	GQ848365	GU991523
*X. flabelliformis* (Schwein.) Fr.	Papua New Guinea	*YMJ159* from *Van der Gucht & De Meester 92–521*, as *X*. *cubensis* in Van der Gucht (1995) and Hsieh et al. (2010)	GQ502702	GQ455446	GQ853017	MZ854247
*X. flabelliformis* (Schwein.) Fr.	Russian Far East	*YMJ477* from *Vasilyeva, L. N.*, as *X*. *cubensis* in Hsieh et al. (2010)	GQ502699	GQ455443	GQ848364	MZ854248
*X. flabelliformis* (Schwein.) Fr.	French West Indies	*YMJ515* from *Lechat, C. CLL5121* (Fournier et al. 2019), as *X*. *cubensis* in Hsieh et al. (2010)	GQ502701	GQ455445	GQ848366	GU373810
*X. flabelliformis* (Schwein.) Fr.	French Guiana	*YMJ3237* from *Fournier, J. GYJF21318* (this study)	**PZ393402**	**PZ370594**	**PZ393562**	**PZ373845**
*X. frustulosa* (Berk. & M. A. Curtis) Cooke	French West Indies	*YMJ771* from *Lechat, C. CLL6002-2* (Hsieh et al. 2010; Fournier et al. 2018c)	GQ495943	GQ449237	GQ844837	GU322450
*X. frustulosa* (Berk. & M. A. Curtis) Cooke	Taiwan	*Ju & Hsieh 92092010* (Hsieh et al. 2010)	GQ495944	GQ449240	GQ844838	GU322451
*X. furcata* Fr.	Taiwan	*YMJ1730* from EPITYPE (Ju et al. 2023)	OQ818471	OQ818411	OQ851565	OQ842995
*X. furcatula* Y.-M. Ju & H.-M. Hsieh	Taiwan	*YMJ1099* from HOLOTYPE (Ju et al. 2023)	OQ818433	OQ818373	OQ851527	OQ845483
*X. gigartosperma* J. Fourn. et al.	French Guiana	*YMJ3257* from *Fournier, J. GYJF21418* (this study)	**PZ393403**	**PZ370595**	**PZ393563**	**PZ373846**
*X. gigartosperma* J. Fourn. et al.	French Guiana	*YMJ3261* from *Fournier, J. GYJF21434* (this study)	**PZ393404**	**PZ370596**	**PZ393564**	**PZ373847**
*X. gigartosperma* J. Fourn. et al.	French Guiana	*YMJ1857* from *Fournier, J. GYJF18056* (this study)	**PZ393405**	**PZ370597**	**PZ393565**	**PZ373848**
*X. gigartosperma* J. Fourn. et al.	French Guiana	*YMJ3243* from *Fournier, J. GYJF21334* (this study)	**PZ393406**	**PZ370598**	**PZ393566**	**PZ373849**
*X. gigaspora* J. Fourn. et al.	French Guiana	*YMJ1861* from *Fournier, J. GYJF18099-1* (this study)	**PZ393407**	**PZ370599**	**PZ393567**	**PZ373850**
*X. globosa* (Spreng.) Mont.	French West Indies	*YMJ775* from *Lechat, C. CLL6033* (Hsieh et al. 2010)	GQ502684	GQ452369	GQ848348	GU324735
*X. globosa* (Spreng.) Mont.	French Guiana	*YMJ3207* from *Fournier, J. GYJF21193* (this study)	**PZ393408**	**PZ370600**	**PZ393568**	**PZ373851**
*X. gracillima* (Fr.) Fr.	French Guiana	*YMJ3249* from *Fournier, J. GYJF21372* (this study)	**PZ393409**	**PZ370601**	**PZ393569**	**PZ373852**
*X. gracillima* (Fr.) Fr.	French Guiana	*YMJ3203* from *Fournier, J. GYJF21177* (this study)	**PZ393410**	**PZ370602**	**PZ393570**	**PZ373853**
*X. gracillima* (Fr.) Fr.	French Guiana	*YMJ1928* from *Fournier, J. GYJF19087* (this study)	**PZ393411**	**PZ370603**	**PZ393571**	**PZ373854**
*X. gracillima* (Fr.) Fr.	French Guiana	*YMJ3226* from *Fournier, J. GYJF21256* (this study)	**PZ393412**	**PZ370604**	**PZ393572**	**PZ373855**
*X. gracillima* (Fr.) Fr.	French Guiana	*YMJ3259* from *Fournier, J. GYJF21429* (this study)	**PZ393413**	**PZ370605**	**PZ393573**	**PZ373856**
*X. gracillima* (Fr.) Fr.	French Guiana	*YMJ3213* from *Fournier, J. GYJF21211* (this study)	**PZ393414**	**PZ370606**	**PZ393574**	**PZ373857**
*X. grammica* (Mont.) Fr.	Taiwan	*YMJ479* from *Chen, G.-T.* (Hsieh et al. 2010)	GQ487704	GQ427197	GQ844813	GU300097
*X. grammica* (Mont.) Fr.	French Guiana	*YMJ1873* from *Fournier, J. GYJF18017* (this study)	**PZ393415**	**PZ370607**	**PZ393575**	**PZ373858**
*X. griseosepiacea* Y.-M. Ju & H.-M. Hsieh	Taiwan	*YMJ641* from HOLOTYPE (Ju and Hsieh 2007)	GQ502714	GQ853049	GQ853031	EU179865
*X. guadalupensis* J. Fourn. et al.	French West Indies	*YMJ526* from *Lechat, C. CLL5437* (Fournier et al. 2018c), as *Penzigia* in Hsieh et al. (2010)	GQ478220	GQ408907	GQ844798	GU300085
*X. guepini* (Fr.) Fr.	France	*YMJ2019* from *Huart, D. HD 20120101* (Hsieh et al. 2022)	OP856892	OP856890	OP856891	OP863307
*X. guianica* J. Fourn. et al.	French Guiana	*YMJ1929* from *Fournier, J. GYJF19091-2* (this study)	**PZ393416**	**PZ370608**	**PZ393576**	**PZ373859**
*X. guianica* J. Fourn. et al.	French Guiana	*YMJ3219* from *Fournier, J. GYJF21240* (this study)	**PZ393417**	**PZ370609**	**PZ393577**	**PZ373860**
*X. guianica* J. Fourn. et al.	French Guiana	*YMJ3147* from *Fournier, J. GYJF21007* (this study)	**PZ393418**	**PZ370610**	**PZ393578**	**PZ373861**
*X. guianica* J. Fourn. et al.	French Guiana	*YMJ3169* from *Fournier, J. GYJF21085* (this study)	**PZ393419**	**PZ370611**	**PZ393579**	**PZ373862**
*X. guyanensis* (Mont.) Fr.	French Guiana	*YMJ1852* from *Fournier, J. GYJF18117* (this study)	**PZ393420**	**PZ370612**	**PZ393580**	**PZ373863**
*X. haemorrhoidalis* Berk. & Broome	Taiwan	*Ju & Hsieh 89041207* (Hsieh et al. 2010)	GQ502683	GQ452368	GQ848347	GU322464
*X. haemorrhoidalis* Berk. & Broome	French Guiana	*YMJ3205* from *Fournier, J. GYJF21188* (this study)	**PZ393421**	**PZ370613**	**PZ393581**	**PZ373864**
*X. heliscus* (Mont.) J. D. Rogers & Y.-M. Ju	French Guiana	*YMJ3175* from *Fournier, J. GYJF21096* (this study)	**PZ393422**	**PZ370614**	**PZ393582**	**PZ373865**
*X.* cf. *heliscus* (Mont.) J. D. Rogers & Y.-M. Ju	Taiwan	*Ju & Hsieh 88113010* (Hsieh et al. 2010)	GQ502691	GQ452376	GQ848355	GU324742
*X. hoehnelii* Y.-M. Ju & H.-M. Hsieh	Taiwan	*YMJ643* from HOLOTYPE (Ju et al. 2023)	OQ818439	OQ818379	OQ851533	OQ845489
*X. holmbergii Speg.*	French Guiana	*YMJ3231 from Fournier, J. & Thonnel, A. GYJF21286 (this study)*	**PZ393423**	**PZ370615**	**PZ393583**	**PZ373866**
*X. holmbergii Speg.*	French Guiana	*YMJ3248 from Fournier, J. GYJF21370-2 (this study)*	**PZ393424**	**PZ370616**	**PZ393584**	**PZ373867**
*X. holmbergii Speg.*	French Guiana	*YMJ3227 from Fournier, J. GYJF21257 (this study)*	**PZ393425**	**PZ370617**	**PZ393585**	**PZ373868**
*X. holmbergii Speg.*	French Guiana	*YMJ3192 from Fournier, J. GYJF21138 (this study)*	**PZ393426**	**PZ370618**	**PZ393586**	**PZ373869**
*X. hypoxylon* (L.) Grev.	Belgium	*YMJ152* from *Ju, Y.-M.* (Hsieh et al. 2010)	GQ260187	GQ427196	GQ844812	GU300096
*X. hypoxylon* (L.) Grev.	Taiwan	*Guu, J.-R. 95082001* (Hsieh et al. 2010)	GQ487703	GQ427195	GQ844811	GU300095
*X. ianthinovelutina* (Mont.) Fr.	French West Indies	*YMJ553* from *Lechat, C. CLL5599* (Hsieh et al. 2010)	GQ495934	GQ438755	GQ844828	GU322441
*X. ianthinovelutina* (Mont.) Fr.	French Guiana	*YMJ1938* from *Corriol, G. GYJF19175* (this study)	**PZ393427**	**PZ370619**	**PZ393587**	**PZ373870**
*X. insignifurcata* Y.-M. Ju & H.-M. Hsieh	Taiwan	*YMJ649* from HOLOTYPE (Ju et al. 2023)	OQ818446	OQ818386	OQ851540	OQ845496
*X. insolita* Y.-M. Ju et al.	Taiwan	*YMJ1251* from HOLOTYPE (Hsieh et al. 2020)	MN656983	MN656985	MN656981	MN655979
*X. intracolorata* (J. D. Rogers et al.) J. D. Rogers & Y.-M. Ju	Taiwan	*Ju & Hsieh 90080402* (Hsieh et al. 2010)	GQ502690	GQ452375	GQ848354	GU324741
*X. intraflava* Y.-M. Ju & H.-M. Hsieh	Taiwan	*YMJ725* from HOLOTYPE (Ju and Hsieh 2007)	GQ502718	GQ853053	GQ853035	EU179866
*X. iriomotensis* Okane et al.	Japan	*YMJ1497* from HOLOTYPE (Okane et al. 2025)	PQ605185	PQ605195	PQ605190	AB274815
*X. ischnostroma* Wangsawat et al.	Thailand	*SWUF18-22.1* from HOLOTYPE (Wangsawat et al. 2021)	OQ845435	OQ845427	OQ851587	MT622788
*X. juruensis* Henn.	Taiwan	*Ju & Hsieh 92042501* (Hsieh et al. 2010)	GQ495932	GQ438753	GQ844825	GU322439
*X. kegeliana* (Lév.) Fr.	French Guiana	*YMJ3255* from *Fournier, J. GYJF21398* (this study)	**PZ393428**	**PZ370620**	**PZ393588**	**PZ373871**
*X. kegeliana* (Lév.) Fr.	French Guiana	*YMJ3199* from *Fournier, J. GYJF21164* (this study)	**PZ393429**	**PZ370621**	**PZ393589**	**PZ373872**
*X. lechatii* Y.-M. Ju et al.	French West Indies	*YMJ780* from ISOTYPE (HAST 131,025) (Ju et al. 2012)	JQ087415	JQ087409	JQ087412	JQ087406
*X. liquidambar* J. D. Rogers et al.	Taiwan	*Ju & Hsieh 93090701* (Hsieh et al. 2010)	GQ487702	GQ421290	GQ844810	GU300094
*X. macouriana* J. Fourn. et al.	French Guiana	*YMJ961* from *Lechat, C. CLL8095* (this study)	**PZ393430**	**PZ370622**	**PZ393590**	**PZ373873**
*X. mamillata* J. Fourn. et al.	French Guiana	*YMJ3232* from *Fournier, J. GYJF21291* (this study)	**PZ393431**	**PZ370623**	**PZ393591**	**PZ373874**
*X. martinicensis* J. Fourn. & Lechat var. *martinicensis*	French West Indies	*YMJ508* from ISOTYPE (HAST145132) (Fournier et al. 2020), also in Hsieh et al. (2010), as *X*. *luteostromata* var. *macrospora*	GQ502688	GQ452373	GQ848352	GU324739
*X. martinicensis* var. *microspora* J. Fourn. & Lechat	French West Indies	*YMJ2236* from ISOTYPE (HAST) (Fournier et al. 2020)	**PZ393432**	**PZ370624**	**PZ393592**	**PZ373875**
*X. melaniae* J. Fourn. et al.	French Guiana	*YMJ2259* from stroma of *Roy, M. GYJF12098* (this study)	–	**PZ370625**	–	**PZ373876**
*X. meliacearum* Læssøe	Puerto Rico	*YMJ148* from *Lodge, D. J. PR-894* (Læssøe and Lodge 1994)	GQ478219	GQ408906	GQ844797	GU300084
*X. meliacearum* Læssøe	French Guiana	*YMJ3197* from *Fournier, J. GYJF21160* (this study)	**PZ393433**	**PZ370626**	**PZ393593**	**PZ373877**
*X. mianyangensis* Y.-M. Ju et al.	China	*YMJ1321* from HOLOTYPE (Ju et al. 2022)	MZ901319	MZ901347	MZ901333	MZ888986
*X. microceras* (Mont.) Fr.	French West Indies	*YMJ414* from *Lechat, C. CLL2265* (Hsieh et al. 2010)	GQ478221	GQ408908	GQ844799	GU300086
*X. minima* Wangsawat et al.	Thailand	*SWUF18-3.2* from HOLOTYPE (Wangsawat et al. 2021)	OQ845436	OQ845428	OQ851588	MT622789
*X. minor* J. Fourn. et al.	French Guiana	*YMJ3166* from *Fournier, J. GYJF21066* (this study)	**PZ393434**	**PZ370627**	**PZ393594**	**PZ373878**
*X. minor* J. Fourn. et al.	French Guiana	*YMJ1945* from *Fournier, J. GYJF19218* (this study)	**PZ393435**	**PZ370628**	**PZ393595**	**PZ373879**
*X. minor* J. Fourn. et al.	French Guiana	*YMJ3165* from *Fournier, J. GYJF21057* (this study)	**PZ393436**	**PZ370629**	**PZ393596**	**PZ373880**
*X. minor* J. Fourn. et al.	French Guiana	*YMJ1858* from *Gardiennet, A. GYJF18128* (this study)	**PZ393437**	**PZ370630**	**PZ393597**	**PZ373881**
*X. minuscula* Y.-M. Ju & H.-M. Hsieh	Taiwan	*YMJ2369* from HOLOTYPE (Ju and Hsieh 2023)	**PZ393438**	**PZ370631**	**PZ393598**	OQ883721
*X. multiplex* (Kunze) Fr.	French West Indies	*YMJ580* from *Lechat, C. CLL5287* (Hsieh et al. 2010)	GQ487705	GQ427198	GQ844814	GU300098
*X. multiplex* (Kunze) Fr.	USA, Hawaiian Islands	*YMJ259* from *Hemmes, D. E. Xy-7* (Hsieh et al. 2010)	GQ487706	GQ438743	GQ844815	GU300099
*X. multiplex* (Kunze) Fr.	French Guiana	*YMJ3195* from *Fournier, J. GYJF21148* (this study)	**PZ393439**	**PZ370632**	**PZ393599**	**PZ373882**
*X. muscula* C. G. Lloyd	French West Indies	*YMJ520* from *Lurel, D. CLL5323* (Hsieh et al. 2010)	GQ478222	GQ408909	GQ844800	GU300087
*X. myosurus* Mont.	French Guiana	*YMJ3247* from stroma of *Fournier, J. GYJF21363* (this study)	**PZ393440**	**PZ370633**	**PZ393600**	–
*X. nelumboniformis* Hai X. Ma et al.	French Guiana	*YMJ3236* from *Fournier, J. GYJF21306* (this study)	**PZ393441**	**PZ370634**	**PZ393601**	**PZ373883**
*X. nelumboniformis* Hai X. Ma et al.	French Guiana	*YMJ1865* from *Fournier, J. GYJF18099* (this study)	**PZ393442**	**PZ370635**	**PZ393602**	**PZ373884**
*X. neonigripes* Y.-M. Ju et al.	Taiwan	*YMJ1202* from HOLOTYPE (Ju et al. 2022)	MZ901321	MZ901349	MZ901335	MZ888988
*X. nigripes* (Klotzsch) Fr.	Taiwan	*YMJ653* from *Chou, K.-H. 94053001* (Ju and Hsieh 2007; Ju et al. 2022)	GQ502710	GQ853045	GQ853027	EU179868
*X. nigrosticta* J. Fourn. et al.	French Guiana	*YMJ1931* from *Fournier, J. GYJF19120* (this study)	**PZ393443**	**PZ370636**	**PZ393603**	**PZ373885**
*X. nigrosticta* J. Fourn. et al.	French Guiana	*YMJ3235* from *Fournier, J. GYJF21305* (this study)	**PZ393444**	**PZ370637**	**PZ393604**	**PZ373886**
*X. nigrosticta* J. Fourn. et al.	French Guiana	*YMJ1924* from *Fournier, J. GYJF19043* (this study)	**PZ393445**	**PZ370638**	**PZ393605**	**PZ373887**
*X. nigrosticta* J. Fourn. et al.	French Guiana	*YMJ3218* from *Fournier, J. GYJF21238* (this study)	**PZ393446**	**PZ370639**	**PZ393606**	**PZ373888**
*X. nigrosticta* J. Fourn. et al.	French Guiana	*YMJ3216* from *Fournier, J. GYJF21229* (this study)	**PZ393447**	**PZ370640**	**PZ393607**	**PZ373889**
*X. nigrosticta* J. Fourn. et al.	French Guiana	*YMJ3256* from *Fournier, J. GYJF21407* (this study)	**PZ393448**	**PZ370641**	**PZ393608**	**PZ373890**
*X. ochraceostroma* Y.-M. Ju & H.-M. Hsieh	Taiwan	*YMJ401* from HOLOTYPE (Ju and Hsieh 2007)	GQ502717	GQ853052	GQ853034	EU179869
*X. oligotoma* Sacc. & Paol.	French Guiana	*YMJ784* from *Lechat, C. CLL7031* (Hsieh et al. 2010)	GQ487700	GQ421288	GQ844808	GU300092
*X. oligotoma* Sacc. & Paol.	French Guiana	*YMJ1088* from *Lechat, C. CLL9040* (this study)	**PZ393449**	**PZ370642**	**PZ393609**	**PZ373891**
*X. oligotoma* Sacc. & Paol.	French Guiana	*YMJ3239* from *Fournier, J. GYJF21322* (this study)	**PZ393450**	**PZ370643**	**PZ393610**	**PZ373892**
*X. oligotoma* Sacc. & Paol.	French Guiana	*YMJ1943* from *Fournier, J. GYJF19208* (this study)	**PZ393451**	**PZ370644**	**PZ393611**	**PZ373893**
*X. oligotoma* Sacc. & Paol.	French Guiana	*YMJ1853* from *Fournier, J. GYJF18045* (this study)	**PZ393452**	**PZ370645**	**PZ393612**	**PZ373894**
*X. oligotoma* Sacc. & Paol.	French Guiana	*YMJ1941* from *Fournier, J. GYJF19200* (this study)	**PZ393453**	**PZ370646**	**PZ393613**	**PZ373895**
*X. ophiopoda* Sacc.	Taiwan	*Ju & Hsieh 93082805* (Hsieh et al. 2010)	GQ495955	GQ452365	GQ848344	GU322461
*X. orichalcea* J. Fourn. et al.	French Guiana	*YMJ1946* from *Fournier, J. GYJF19225* (this study)	**PZ393454**	**PZ370647**	**PZ393614**	**PZ373896**
*X. orichalcea* J. Fourn. et al.	French West Indies	*YMJ522* from *Lechat, C. CLL5534* (this study)	**PZ393455**	**PZ370648**	**PZ393615**	**PZ373897**
*X. oxyacanthae* Tul. & C. Tul.	USA	*YMJ859* from *Yeomans, R.* (Hsieh et al. 2010)	GQ495927	GQ438748	GQ844820	GU322434
*X. oxycephala* J. Fourn. et al.	French Guiana	*YMJ1851* from *Fournier, J. GYJF18018* (this study)	**PZ393456**	**PZ370649**	**PZ393616**	**PZ373898**
*X. palifera* J. Fourn. et al.	French Guiana	*YMJ3180* from *Fournier, J. GYJF21105* (this study)	**PZ393457**	**PZ370650**	**PZ393617**	**PZ373899**
*X. palifera* J. Fourn. et al.	French Guiana	*YMJ1940* from *Fournier, J. GYJF19187* (this study)	**PZ393458**	**PZ370651**	**PZ393618**	**PZ373900**
*X. pallidocylindracea* J. Fourn. & Lechat	French West Indies	*YMJ1572* from ISOTYPE (HAST 145151) (Fournier et al. 2020)	**PZ393459**	**PZ370652**	**PZ393619**	**PZ373901**
*X. pallidocylindracea* J. Fourn. & Lechat	French Guiana	*YMJ962* from *Lechat, C. CLL8006* (this study)	**PZ393460**	**PZ370653**	**PZ393620**	**PZ373902**
*X. palmicola* G. Winter	New Zealand	*YMJ604* from *Samuels, G. J. 85–83* (Hsieh et al. 2010)	GQ495929	GQ438750	GQ844822	GU322436
*X. papillatoides* J. Fourn. & Lechat	French Guiana	*YMJ1859* from *Fournier, J. GYJF18140* (this study)	**PZ393461**	**PZ370654**	**PZ393621**	**PZ373903**
*X. papulis* C. G. Lloyd	Taiwan	*Ju & Hsieh 89021903* (Hsieh et al. 2010)	GQ487707	GQ438744	GQ844816	GU300100
*X. partita* C. G. Lloyd	French Guiana	*YMJ3194* from *Fournier, J. GYJF21144* (this study)	**PZ393462**	**PZ370655**	**PZ393622**	**PZ373904**
*X. parvispora* J. Fourn. et al.	French Guiana	*YMJ3234* from *Fournier, J. GYJF21304* (this study)	**PZ393463**	**PZ370656**	**PZ393623**	**PZ373905**
*X. parvispora* J. Fourn. et al.	French Guiana	*YMJ3201* from *Fournier, J. GYJF21168* (this study)	**PZ393464**	**PZ370657**	**PZ393624**	**PZ373906**
*X. penicilliopsis* (Henn.) Y.-M. Ju	Taiwan	*YMJ2456* from *Tang, Y.-Y. 107090606*, Hsin-chu County, Jian-shi Township, Yu-lao, on wood (HAST)	**PZ393465**	**PZ370658**	**PZ393625**	**PZ373907**
*X. penicilliopsis* (Henn.) Y.-M. Ju	French Guiana	*YMJ792* from *Lechat, C. CLL8109* (this study)	**PZ393466**	**PZ370659**	**PZ393626**	**PZ373908**
*X. penicilliopsis* (Henn.) Y.-M. Ju	French Guiana	*YMJ967* from *Lechat, C. CLL7080A* (this study)	**PZ393467**	**PZ370660**	**PZ393627**	**PZ373909**
*X. petiolorum* J. Fourn. et al.	French Guiana	*YMJ1942* from *Fournier, J. GYJF19201* (this study)	**PZ393468**	**PZ370661**	**PZ393628**	**PZ373910**
*X. phyllocharis* Mont.	French West Indies	*YMJ528* from *Lechat, C. CLL5302* (Hsieh et al. 2010)	GQ495938	GQ449234	GQ844832	GU322445
*X. piloides* J. Fourn. et al.	French Guiana	*YMJ793* from *Lechat, C. CLL7081* (this study)	**PZ393469**	**PZ370662**	**PZ393629**	**PZ373911**
*X. plebeja* Ces.	Taiwan	*Ju & Hsieh 91122401* (Hsieh et al. 2010)	GQ502689	GQ452374	GQ848353	GU324740
*X. polymorpha* (Pers.) Grev.	USA	*YMJ1012* from *Rogers, J. D.* (Hsieh et al. 2010)	GQ495954	GQ452364	GQ848343	GU322460
*X. primorskensis* Y.-M. Ju et al.	Russian Far East	*YMJ478* from HOLOTYPE (Ju et al. 2009)	**PZ393470**	**PZ370663**	**PZ393630**	FJ707473
*X. reevesiae* Y.-M. Ju et al.	Taiwan	From HOLOTYPE (Ju et al. 2018), as *X*. sp. 7 in Hsieh et al. (2010)	GQ495928	GQ438749	GQ844821	GU322435
*X. regalis* Cooke	India	*YMJ920* from *Gailawad, S. AMH 9204* (Hsieh et al. 2010)	GQ502694	GQ452379	GQ848358	GU324745
*X. regalis* Cooke	Taiwan	*Ju & Hsieh 92072001* (Hsieh et al. 2010)	GQ502693	GQ452378	GQ848357	GU324744
*X. rhytidophloea* Mont.	French Guiana	*YMJ3202* from *Fournier, J. GYJF21174* (this study)	**PZ393471**	**PZ370664**	**PZ393631**	**PZ373912**
*X. rhytidophloea* Mont.	French Guiana	*YMJ3159* from *Fournier, J. GYJF21033* (this study)	**PZ393472**	**PZ370665**	**PZ393632**	**PZ373913**
*X. rhytidosperma* J. Fourn. & Lechat	French West Indies	*YMJ431* from ISOTYPE (Fournier et al. 2018c), as *X*. cf. *glebulosa* in Hsieh et al. (2010)	GQ495956	GQ452366	GQ848345	GU322462
*X. rhytidosperma* J. Fourn. & Lechat	French Guiana	*YMJ3206* from *Fournier, J. GYJF21191* (this study)	**PZ393473**	**PZ370666**	**PZ393633**	**PZ373914**
*X. rhytidosperma* J. Fourn. & Lechat	French Guiana	*YMJ1862* from *Fournier, J. GYJF18151* (this study)	**PZ393474**	**PZ370667**	**PZ393634**	**PZ373915**
*X. robusta* J. Fourn. et al.	French Guiana	*YMJ3191* from *Fournier, J. GYJF21137* (this study)	**PZ393475**	**PZ370668**	**PZ393635**	**PZ373916**
*X. rogersionigripes* Y.-M. Ju et al.	Taiwan	*YMJ1795* from HOLOTYPE (Ju et al. 2022)	MZ901325	MZ901353	MZ901339	MZ888992
*X. rohrensis* Friebes et al.	Austria	*YMJ2006* from HOLOTYPE (Friebes et al. 2022)	ON262768	ON262771	ON262774	ON261187
*X. rossmaniae* Y.-M. Ju et al.	French Guiana	*YMJ3148* from *Fournier, J. GYJF21009* (this study)	**PZ393476**	**PZ370669**	**PZ393636**	**PZ373917**
*X. saulensis* J. Fourn. et al.	French Guiana	*YMJ3228* from *Fournier, J. GYJF21260* (this study)	**PZ393477**	**PZ370670**	**PZ393637**	**PZ373918**
*X. saulensis* J. Fourn. et al.	French Guiana	*YMJ3152* from *Fournier, J. GYJF21013* (this study)	**PZ393478**	**PZ370671**	**PZ393638**	**PZ373919**
*X. saulensis* J. Fourn. et al.	French Guiana	*YMJ1919* from *Fournier, J. GYJF19003* (this study)	**PZ393479**	**PZ370672**	**PZ393639**	**PZ373920**
*X. saulensis* J. Fourn. et al.	French Guiana	*YMJ1934* from *Corriol, G. GYJF19138* (this study)	**PZ393480**	**PZ370673**	**PZ393640**	**PZ373921**
*X. saulensis* J. Fourn. et al.	French Guiana	*YMJ3238* from *Fournier, J. GYJF21319* (this study)	**PZ393481**	**PZ370674**	**PZ393641**	**PZ373922**
*X. saulensis* J. Fourn. et al.	French Guiana	*YMJ3150* from *Fournier, J. GYJF21011* (this study)	**PZ393482**	**PZ370675**	**PZ393642**	**PZ373923**
*X. scabra* J. Fourn. et al.	French Guiana	*YMJ1866* from *Fournier, J. GYJF18104* (this study)	**PZ393483**	**PZ370676**	**PZ393643**	**PZ373924**
*X. schweinitzii* Berk. & M. A. Curtis	Taiwan	*Ju & Hsieh 92092023* (Hsieh et al. 2010)	GQ495957	GQ452367	GQ848346	GU322463
*X. schweinitzii* Berk. & M. A. Curtis	French Guiana	*YMJ3186* from *Fournier, J. GYJF21128* (this study)	**PZ393484**	**PZ370677**	**PZ393644**	**PZ373925**
*X. scoparia* Pat.	Taiwan	*YMJ1435* from EPITYPE (Ju et al. 2023)	OQ818489	OQ818429	OQ851583	OQ843014
*X. scruposa* (Fr.) Fr.	French West Indies	*YMJ497* from *Lechat, C. CLL5025* (Hsieh et al. 2010)	GQ495952	GQ452362	GQ848341	GU322458
*X. scutulata* J. Fourn. et al.	French Guiana	*YMJ3225* from *Fournier, J. GYJF21255* (this study)	**PZ393485**	**PZ370678**	**PZ393645**	**PZ373926**
*X. scutulata* J. Fourn. et al.	French Guiana	*YMJ3184* from *Fournier, J. GYJF21118* (this study)	**PZ393486**	**PZ370679**	**PZ393646**	**PZ373927**
*X. scutulata* J. Fourn. et al.	French Guiana	*YMJ1932* from *Fournier, J. GYJF19126* (this study)	**PZ393487**	**PZ370680**	**PZ393647**	**PZ373928**
*X. scutulata* J. Fourn. et al.	French Guiana	*YMJ3251* from *Fournier, J. GYJF21375* (this study)	**PZ393488**	**PZ370681**	**PZ393648**	**PZ373929**
*X. scutulata* J. Fourn. et al.	French Guiana	*YMJ1939* from *Fournier, J. GYJF19176* (this study)	**PZ393489**	**PZ370682**	**PZ393649**	**PZ373930**
*X. siamensis* Wangsawat et al.	Thailand	*SWUF17-20.2* from HOLOTYPE (Wangsawat et al. 2021; Ju et al. 2023)	OQ845437	OQ845429	OQ851589	MT622765
*X. sibirica* Y.-M. Ju et al.	Russian Far East	*YMJ762* from ISOTYPE (HAST) (Ju et al. 2009)	**PZ393490**	**PZ370683**	**PZ393650**	FJ707474
*X. siliquarum* J. Fourn. et al.	French Guiana	*YMJ1898* from *Courtecuisse, R. GYJF18165* (this study)	**PZ393491**	**PZ370684**	**PZ393651**	**PZ373931**
*X. siliquarum* J. Fourn. et al.	French Guiana	*YMJ3196* from *Fournier, J. GYJF21157* (this study)	**PZ393492**	**PZ370685**	**PZ393652**	**PZ373932**
*X. simplicissima* (Pers.) Y.-M. Ju & H.-M. Hsieh	Finland	*YMJ1454* from EPITYPE (Ju and Hsieh 2023)	**PZ393493**	**PZ370686**	**PZ393653**	OQ883722
*X. sinuosirima* J. Fourn. et al.	French Guiana	*YMJ963* from *Lechat, C. CLL8098* (this study)	**PZ393494**	**PZ370687**	**PZ393654**	**PZ373933**
*X.* sp. A	China	From sclerotium of *WLS2077* (Ju et al. 2022)	MZ901328	MZ901356	MZ901342	MZ888995
*X.* sp. B	China	From sclerotium of *WLS2084* (Ju et al. 2022)	MZ901329	MZ901357	MZ901343	MZ888996
*X*. sp. cf. *X. cantareirensis* (Henn.) J. Fourn. & Lechat	French West Indies	*YMJ377* from *Lechat, C. CLL0863* (this study), as *X. cantareirensis* in (Fournier et al. 2018c)	**PZ393495**	**PZ370688**	**PZ393655**	**PZ373934**
*X*. sp. cf. *X. cantareirensis* (Henn.) J. Fourn. & Lechat	French West Indies	*YMJ2266* from stroma of *Fournier, J. & Lechat, C. MJF10052* (this study), as *X. cantareirensis* in (Fournier et al. 2018c)	–	**PZ370689**	–	–
*X*. sp. cf. *X. cantareirensis* (Henn.) J. Fourn. & Lechat	French West Indies	*YMJ1707* from *Fournier, J. MJF14028* (this study), as *X. cantareirensis* in (Fournier et al. 2018c)	**PZ393496**	**PZ370690**	**PZ393656**	**PZ373935**
*X*. sp. cf. *X. cantareirensis* (Henn.) J. Fourn. & Lechat	French West Indies	*YMJ1015* from *Lechat, C. CLL8275* (this study), as *X. cantareirensis* in (Fournier et al. 2018c)	**PZ393497**	**PZ370691**	**PZ393657**	**PZ373936**
*X*. sp. cf. *X. cantareirensis* (Henn.) J. Fourn. & Lechat	French Guiana	*YMJ2263* from *Lechat, C. CLL7103* (this study)	–	**PZ370692**	–	–
*X*. sp. cf. *X. cantareirensis* (Henn.) J. Fourn. & Lechat	French Guiana	*YMJ3189* from *Fournier, J. GYJF21131* (this study)	**PZ393498**	**PZ370693**	**PZ393658**	**PZ373937**
*X.* sp. I-1 cf. *X. scruposa* (Fr.) Fr.	French Guiana	*YMJ3229* from *Fournier, J. GYJF21272* (this study)	**PZ393499**	**PZ370694**	**PZ393659**	**PZ373938**
*X.* sp. I-2 cf. *X. scruposa* (Fr.) Fr.	French Guiana	*YMJ1872* from *Fournier, J. GYJF18083* (this study)	**PZ393500**	**PZ370695**	**PZ393660**	**PZ373939**
*X.* sp. II-1 cf. *X. scruposa* (Fr.) Fr.	French Guiana	*YMJ3230* from *Fournier, J. GYJF21285* (this study)	**PZ393501**	**PZ370696**	**PZ393661**	**PZ373940**
*X.* sp. II-1 cf. *X. scruposa* (Fr.) Fr.	French Guiana	*YMJ3167* from *Fournier, J. GYJF21067* (this study)	**PZ393502**	**PZ370697**	**PZ393662**	**PZ373941**
*X.* sp. II-2 cf. *X. scruposa* (Fr.) Fr.	French Guiana	*YMJ1869* from *Fournier, J. GYJF18104-1* (this study)	**PZ393503**	**PZ370698**	**PZ393663**	**PZ373942**
*X.* sp. II-3 cf. *X. scruposa* (Fr.) Fr.	French Guiana	*YMJ1870* from *Fournier, J. GYJF18133* (this study)	**PZ393504**	**PZ370699**	**PZ393664**	**PZ373943**
*X.* sp. II-3 cf. *X. scruposa* (Fr.) Fr.	French Guiana	*YMJ1871* from *Gardiennet, A. GYJF18122* (this study)	**PZ393505**	**PZ370700**	**PZ393665**	**PZ373944**
*X.* sp. II-3 cf. *X. scruposa* (Fr.) Fr.	French Guiana	*YMJ1868* from *Fournier, J. GYJF18113* (this study)	**PZ393506**	**PZ370701**	**PZ393666**	**PZ373945**
*X. spegazziniana* J. Fourn. et al.	French Guiana	*YMJ1922* from *Fournier, J. GYJF19027* (this study)	**PZ393507**	**PZ370702**	**PZ393667**	**PZ373946**
*X. spegazziniana* J. Fourn. et al.	French Guiana	*YMJ3246* from *Fournier, J. GYJF21361* (this study)	**PZ393508**	**PZ370703**	**PZ393668**	**PZ373947**
*X. squamulosa* J. Fourn. et al.	French Guiana	*YMJ1923* from *Lechat, C. GYJF19028* (this study)	**PZ393509**	**PZ370704**	**PZ393669**	**PZ373948**
*X. squamulosa* J. Fourn. et al.	French Guiana	*YMJ3157* from *Fournier, J. GYJF21031* (this study)	**PZ393510**	**PZ370705**	**PZ393670**	**PZ373949**
*X. striata* Pat.	China	*YMJ304* from *Leu, L.-S.* (Hsieh et al. 2010)	GQ478224	GQ421284	GQ844803	GU300089
*X. stupenda* J. Fourn. et al.	French Guiana	*YMJ3163* from *Fournier, J. GYJF21045* (this study)	**PZ393511**	**PZ370706**	**PZ393671**	**PZ373950**
*X. subescharoidea* Y.-M. Ju et al.	Taiwan	*YMJ1188* from HOLOTYPE (Hsieh et al. 2020; Ju et al. 2022)	MN656984	MN656986	MN656982	MN655980
*X. subintraflava* Wangsawat et al.	Thailand	*SWUF16-4.3* (Wangsawat et al. 2021)	OQ845438	OQ845430	OQ851590	MT622762
*X. subplebeja* J. Fourn. et al.	French Guiana	*YMJ3183* from *Fournier, J. GYJF21117* (this study)	**PZ393512**	**PZ370707**	**PZ393672**	**PZ373951**
*X. subplebeja* J. Fourn. et al.	French Guiana	*YMJ3164* from *Fournier, J. GYJF21046* (this study)	**PZ393513**	**PZ370708**	**PZ393673**	**PZ373952**
*X. telfairii* (Berk.) Fr.	French West Indies	*YMJ421* from *Lechat, C. CLL2224* (Hsieh et al. 2010; Fournier et al. 2019)	GQ502686	GQ452371	GQ848350	GU324731
*X. telfairii* (Berk.) Fr.	Taiwan	*Ju & Hsieh 90081901* (Hsieh et al. 2010)	GQ502687	GQ452372	GQ848351	GU324738
*X. tenellifurcata* Y.-M. Ju & H.-M. Hsieh	Taiwan	*YMJ1070* from HOLOTYPE (Ju et al. 2023)	OQ818448	OQ818388	OQ851542	OQ845498
*X. terricola* Y.-M. Ju et al.	Taiwan	*YMJ1375* from HOLOTYPE (Chou et al. 2017)	MF577044	MF577045	MF577043	MF577042
*X. termiticola* (Okane & Nakagiri) Y.-M. Ju	Japan	NBRC 102095 from HOLOTYPE (Okane and Nakagiri 2007), as *Geniculisynnema termiticola*	PQ605186	PQ605196	PQ605191	AB274813
*X. theinhirunae* Wangsawat et al.	Thailand	*SWUF17-44.1* from HOLOTYPE (Wangsawat et al. 2021)	OQ845440	OQ845432	OQ851592	MT622771
*X. turbiniformis* J. Fourn. et al.	French Guiana	*YMJ2261* from *Fournier, J. GYJF12141* (this study)	**PZ393514**	**PZ370709**	**PZ393674**	–
*X. uncinata* J. Fourn. et al.	French Guiana	*YMJ1921* from *Fournier, J. GYJF19024-2* (this study)	**PZ393515**	**PZ370710**	**PZ393675**	**PZ373953**
*X. uncinata* J. Fourn. et al.	French Guiana	*YMJ3233* from *Fournier, J. GYJF21295* (this study)	**PZ393516**	**PZ370711**	**PZ393676**	**PZ373954**
*X. vagans* Petch	USA, Hawaiian Islands	*YMJ258* from *Hemmes, D. E. DEH-1052, as X.* sp. 6 in Hsieh et al. (2010)	GQ478217	GQ408904	GQ844795	GU300082
*X. venosula* Speg.	USA, Hawaiian Islands	*Ju & Hsieh 94080508* (Ju et al. 2007)	EF025617	EF025602	GQ844806	EF026149
*X. venustula* Sacc.	Taiwan	*Ju & Hsieh 88113002* (Hsieh et al. 2010)	GQ487699	GQ421287	GQ844807	GU300091
*X. villosipes* J. Fourn. et al.	French West Indies	*YMJ510* from *Lechat, C. CLL5109* (Fournier et al. 2018c)	**PZ393517**	**PZ370712**	**PZ393677**	**PZ373955**
*X. villosipes* J. Fourn. et al.	French Guiana	*YMJ3240* from HOLOTYPE (this study)	**PZ393518**	**PZ370713**	**PZ393678**	**PZ373956**
*X. vinacea* Wangsawat et al.	Thailand	*SWUF18-2.10* from HOLOTYPE (Wangsawat et al. 2021)	PQ605187	PQ605197	PQ605192	MT622783
*X. vinosa* J. Fourn. et al.	French Guiana	*YMJ1936* from *Fournier, J. GYJF19148* (this study)	**PZ393519**	**PZ370714**	**PZ393679**	**PZ373957**
*X. vinosa* J. Fourn. et al.	French Guiana	*YMJ1944* from *Fournier, J. GYJF19208-2* (this study)	**PZ393520**	**PZ370715**	**PZ393680**	**PZ373958**
*X. vinosa* J. Fourn. et al.	French Guiana	*YMJ1856* from *Fournier, J. GYJF18050* (this study)	**PZ393521**	**PZ370716**	**PZ393681**	**PZ373959**
*X. vinosa* J. Fourn. et al.	French Guiana	*YMJ1927* from *Fournier, J. GYJF19083* (this study)	**PZ393522**	**PZ370717**	**PZ393682**	**PZ373960**
*X. vinosa* J. Fourn. et al.	French Guiana	*YMJ3170* from *Fournier, J. GYJF21086* (this study)	**PZ393523**	**PZ370718**	**PZ393683**	**PZ373961**
*X. vinosa* J. Fourn. et al.	French Guiana	*YMJ3211* from *Fournier, J. GYJF21208* (this study)	**PZ393524**	**PZ370719**	**PZ393684**	**PZ373962**
*X. vittatipiliformis* Y.-M. Ju et al.	French West Indies	*YMJ1281* from ISOTYPE (HAST 145985) (Ju and Hsieh 2023)	**PZ393525**	**PZ370720**	**PZ393685**	OQ883723
*X. vivantii* Y.-M. Ju et al.	French West Indies	*YMJ519* from HOLOTYPE (Ju et al. 2018), as *X*. sp. 8 in Hsieh et al. (2010)	GQ495931	GQ438752	GQ844824	GU322438

## Results

### Morphological studies

A total of 100 *Xylaria* taxa are documented from French Guiana, including 11 species previously reported from the region that were not recollected during recent fieldwork by JF and CL. In addition, *X*. *cantareirensis* and *X*. *guadalupensis* are included from Brazil and the French West Indies, respectively, for comparison with morphologically similar Guianese taxa. Among the taxa treated, 38 are newly described species, and one represents a new recombination, highlighting a remarkably high proportion (39%) of previously undocumented diversity within the genus.

### Phylogeny

**Fig. 1:**
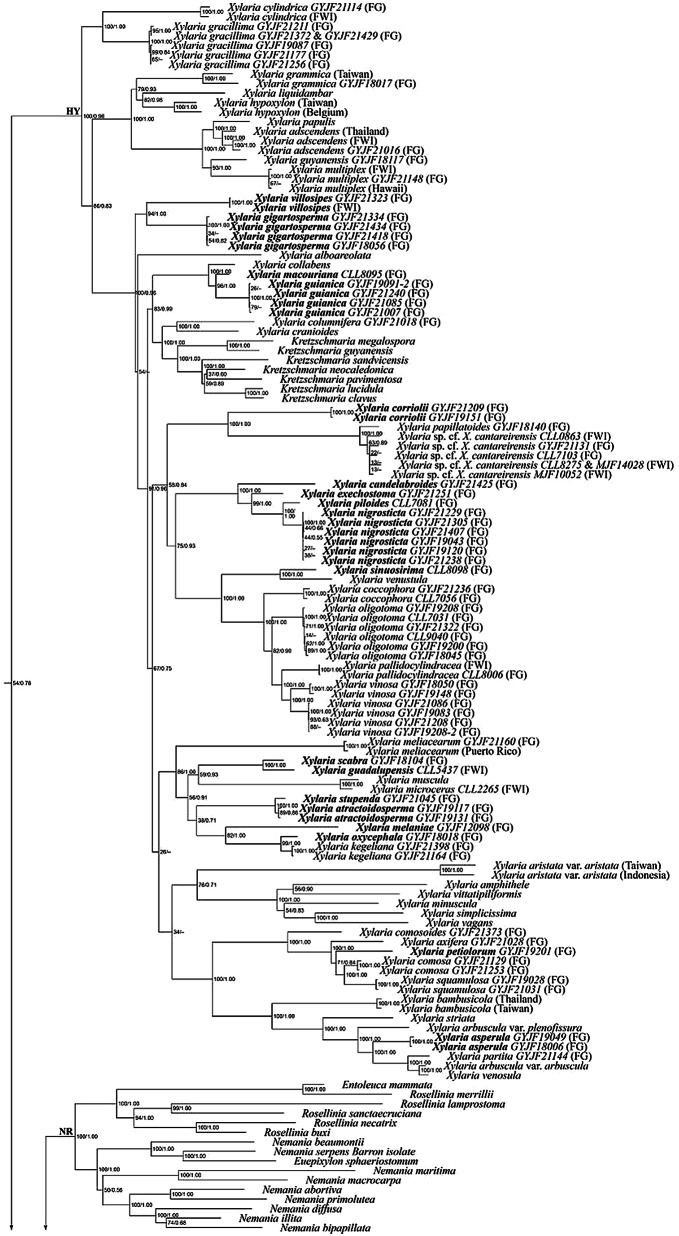

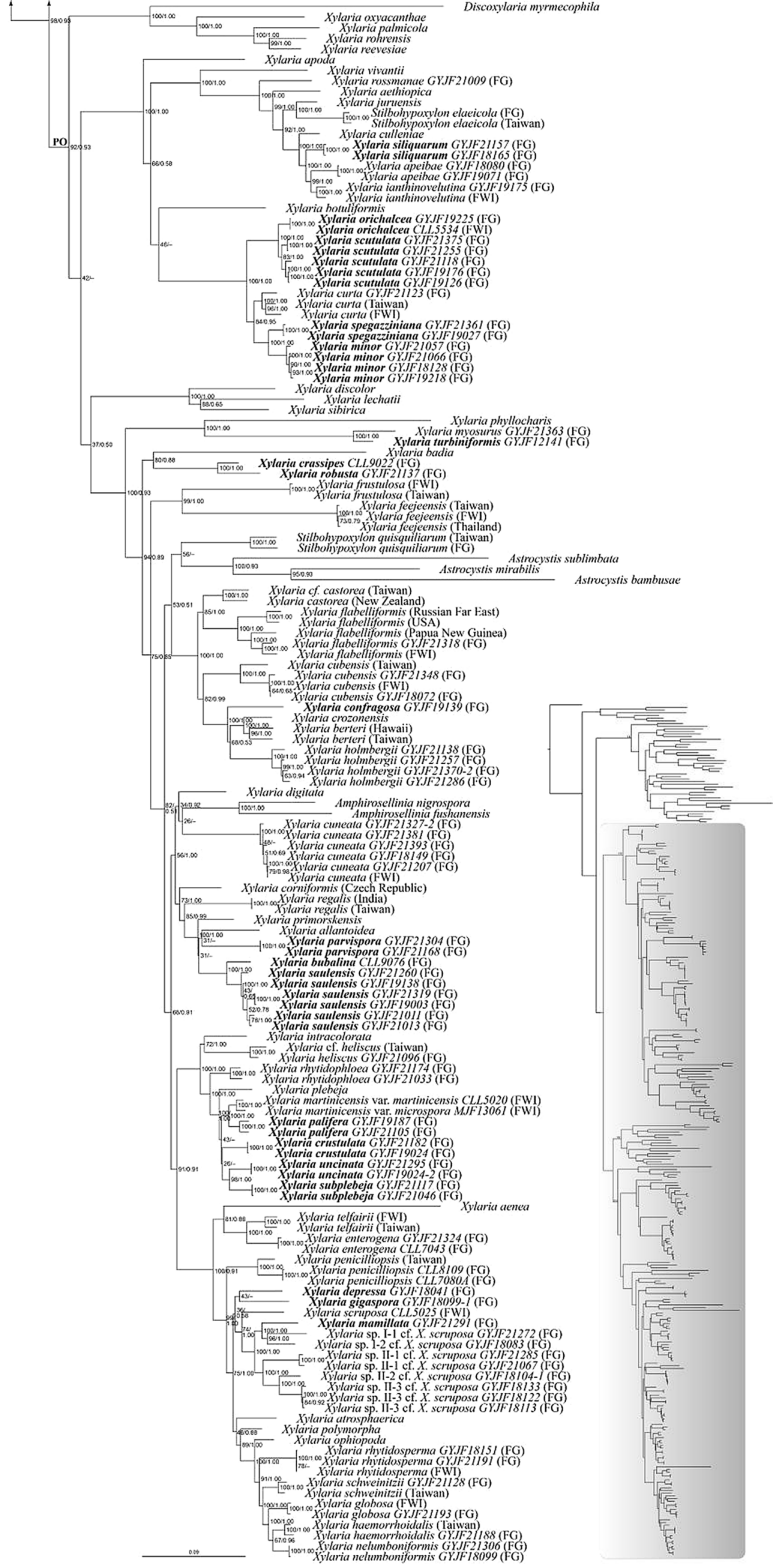
Phylogenetic tree inferred by ML analysis based on the combined RPB2-TUB-ACT dataset. The complete tree is presented in Additional file 1; only a schematic representation is shown in the lower right, with the boxed region enlarged to highlight the clades containing *Xylaria* species from French Guiana, indicated by “FG” in parentheses. Names newly proposed in the present study are shown in bold. Numbers at internodes represent ML bootstrap support values, immediately followed by Bayesian posterior probabilities from the BI analysis

Maximum likelihood (ML) and Bayesian inference (BI) phylogenetic trees inferred from protein-coding loci place the Guianese *Xylaria* taxa within either the HY or PO clades sensu Hsieh et al. (2010). Figure 1 shows only the portion of the ML tree containing these taxa, whereas Additional file 2 presents the complete ML tree. Phylogenetic relationships and taxonomic implications for taxa represented by available sequence data are discussed under the Comments section for each species. Of the newly described species, 37 of 39 species (all except *X*. *fallax* and *X*. *pachylepidota*) were included in the phylogenetic analyses. In all cases where sequence data were available, the results corroborate species delimitations inferred from morphology and further reveal cryptic diversity in at least one morphologically conservative species complex, most notably among taxa allied to *X*. *scruposa*.

## Taxonomy

### Dichotomous keys to *Xylaria* species known from French Guiana

Ascospore dimensions are given as mean values to facilitate their comparison and only occasionally presented as size ranges. New names proposed in the present study are indicated with an asterisk (*).

### Comments on the features used in the keys

Constructing the following keys proved to be a challenging task due to the large number of species involved and the limited number of stable characters with sufficient discriminatory power. In this context, it is difficult to guarantee fully satisfactory effectiveness for the user in all cases.

Most macromorphological features used in the keys are recorded on dry material. However, stromatal appearance may differ in the fresh state, especially with respect to the presence or absence of longitudinal splitting, bumps, wrinkles, or constrictions.

It is generally straightforward to assign a collection to the group of species with penzigioid stromata (Key A), but it should be borne in mind that a typically stipitate species may occasionally produce sessile stromata. A good illustration of this situation was provided in this study by *X. meliacearum*, in which long-stipitate stromata were associated on the same substrate with atypical sessile, subglobose forms.

The group of species with slender stromata apically sterile and attenuated or mucronate (Key B) is generally consistent, but atypical stromata with a rounded apex may sometimes be encountered, leading to dead ends or erroneous identifications. The risk of error is reduced when collections are abundant, as this allows recognition of the typical stromata, which are usually in the majority.

Distinguishing a leathery stromatal texture from what is here refer to as carbonaceous is critical for the successful use of these keys. This requires a specific approach detailed in Material and Methods of Fournier et al. (2018c).

*Xylaria* stromata develop over extended periods, from several weeks to several months, progressing from an immature sterile stage to a mature fertile stage and eventually to a senescent stage, often gradually becoming sterile again and morphologically distinct from early stages. Users of the keys are therefore advised to rely, as far as possible, on specimens that are fully mature and representative. Recognizing the optimal stage of maturity is one of the primary challenges for field taxonomists and requires experience, which is best gained through the study of abundant collections. Such material increases the likelihood of observing the full range of stromatal development and selecting specimens suitable for reliable identification.

For a long time, the taxonomy of *Xylaria* relied on characters visible to the naked eye, such as stromatal stature, color, shape, and branching pattern. Although some of these characters remain useful in some cases, they often prove to be inconsistent and highly variable over time and across environmental conditions, limiting their taxonomic value. For this reason, the present keys use more subtle but more reliable characters, which require the use of a hand lens in the field and a stereomicroscope with a graduated eyepiece in the laboratory.

Particular attention should be paid to the stromatal surface. It is important to observe the presence or absence of a peeling outer layer that may dissociate into patches, plaques or stripes, the color and texture of which can be diagnostically significant. When carbonaceous granules are exposed on the stromatal surface as the outer layer disintegrates, their distribution, density, and arrangement should be carefully recorded. When the peeling outer layer is absent or when it is reduced to a thin, sometimes colored film, superficial cracking may appear during the stromatal growth, resulting in the formation of scales or warts of varying morphology, the density, dimensions, and depth of which are often taxonomically informative.

The shape and dimensions of the ostioles are relatively stable characters that may be diagnostic and should be recorded accurately. The same applies to the texture and color of the stromatal interior, which should be observed in longitudinal section, including the stipe whenever possible.

The anamorph is only rarely present on mature stromata or closely associated substrates, usually as anamorphic pegs or synnemata. However, experience shows that this character is of limited reliability because it is not consistently expressed within a species and may be misleading.

In addition to macroscopic characters, microscopic characters of the ascal apical apparati and ascospores are essential for identification. For ascospores, shape, color, dimensions, presence or absence of appendages, and characteristics of germ slit are critical. Here again, the availability of mature material in good condition is a prerequisite for recording these characters.

### Key to groups

**Table:** 

1. Stromata occurring on dead woody substrates, including woody fruits or pods	**2**
1’. Stromata terrestrial or occurring on dead leaves	**Key E**
2. Stromata subglobose to pulvinate, sessile to subsessile, typically wider than high (penzigioid)	**Key A**
2’. Stromata upright, cylindrical, clavate, or fusiform, typically higher than wide	**3**
3. Stromata often slender, apically sterile and attenuated to mucronate	**Key B**
3’. Stromata often stout, apically fertile and obtusely rounded	**4**
4. Stromata carbonaceous	**Key C**
4’. Stromata leathery	**Key D**

### Key A Stromata penzigioid

**Table:** 

1. Stromata 0.5–2.5 mm diam	**2**
1’. Stromata more than 2.5 mm diam	**12**
2. Ascospores less than 10 µm long on average	**3**
2’. Ascospores 15–46 µm long on average	**5**
3. Stromata subglobose on a short densely hairy-tomentose stipe; ascospores 9.3 × 4.1 µm, inequilateral, medium brown	***X. villosipes*** *
3’. Stromata turbinate, associated with peg-like cylindrical coremial structures	**4**
4. Peg-like coremial structures central and prominent; ascospores 9.0 × 3.8 µm, equilateral, light brown, with a short germ slit 2–3 µm long	***X. turbiniformis*** *
4’. Peg-like coremial structures occasional, at the apex or base of stromata; ascospores 9.3 × 4.8 µm, inequilateral, dark brown, with a germ slit almost spore-length	***X. palifera*** *
5. Stromatal surface coarsely cracked and warty; ascospores more than 30 µm long on average	**6**
5’. Stromatal surface not warty; ascospores smaller	**7**
6. Ascospores 32.0 × 10.0 µm, with a short oblique to diagonal germ slit	***X. rhytidosperma***
6’. Ascospores 46.8 × 13.0 µm, with a short, slightly oblique germ slit	***X. gigaspora*** *
7. Stromatal outer layer white to cream-colored; ascospores with a straight germ slit almost spore-length	**8**
7’. Stromatal outer layer cracked into gray to tan scales; ascospores with a spiraling germ slit less than spore-length	**11**
8. Stromata frequently umbonate	**9**
8’. Stromata mostly lacking a central umbo	**10**
9. Stromatal outer layer white; ascospores 15.3 × 6.5 µm	***X. papillatoides***
9’. Stromatal outer layer cream-colored; ascospores 24.8 × 9.0 µm	***X. guadalupensis*** *
10. Ascospores 28.1 × 10.1 µm	***X. cantareirensis***
10’. Ascospores smaller, 18.8 × 8.0 µm to 25.7 × 9.1 µm	***X.*** **sp. cf.** ***X. cantareirensis***
11. Stromatal crust leathery, 50–60 µm thick; ascospores 24.2 × 6.8 µm, light to medium brown	***X. meliacearum***
11’. Stromatal crust carbonaceous, 80–120 µm thick; ascospores 28.7 × 10.9 µm, dark brown	***X. fallax*** *
12. Stromatal crust strongly carbonaceous, 120–210 µm thick	**13**
12’. Stromatal crust leathery to slightly carbonaceous, 40–100 µm thick	**16**
13. Stromata pulvinate to effused-pulvinate; ascal apical apparatus 1.3 × 2.6 µm; ascospores 11.4 × 6.7 µm	***X. berteri***
13’. Stromata convex, hemispherical to subglobose; ascal apical apparatus and ascospores much larger	**14**
14. Stromata subglobose, with apical conidial processes; ascospores 30.4 × 7.8 µm, medium brown, with two polar appendages and a long germ slit on the dorsal side	sessile form of*** X. comosa***
14’. Stromata hemispherical; ascospores blackish brown, with a germ slit on the ventral side	**15**
15. Stromatal surface gray, plane, with appressed white scales; ascospores inequilateral, 30.6 × 9.4 µm, with broadly rounded ends and bipolar appendages	***X. alboareolata***
15’. Stromatal surface blackish, roughened by thick warts; ascospores equilateral, 30.3 × 12.7 µm, with subacute ends, lacking appendages	***X. pachylepidota*** *
16. Stromatal surface corky-cracked; ascospores longer than 20 µm on average	**17**
16’. Stromatal surface lacking warts; ascospores shorter than 20 µm on average	**18**
17. Stromata subglobose to irregular, coarsely corky-cracked on the surface, with traces of orange at the interior of stipe base; ostioles broadly discoid; ascospores 25.0 × 7.9 µm	***X. globosa***
17’. Stromata peltate, with the surface finely corky-cracked and overlain with gray scales; ostioles finely papillate; ascospores 23.6 × 8.4 µm	***X. nelumboniformis***
18. Stromata turbinate, with a bumpy surface; ascospores 15.5 × 5.7 µm, with a short sigmoid germ slit	***X. mamillata*** *
18’. Stromata sessile, broadly attached to the substrate	**19**
19. Stromata flat-topped; ascospores 18.5 × 6.5 µm, with a short oblique germ slit	***X. depressa*** *
19’. Stromata convex, frequently umbonate; ascospores 15.3 × 6.0 µm, with a straight to slightly undulate germ slit almost spore-length	***X. asperula*** *

### Key B Stromata upright, apically sterile and attenuated or mucronate

**Table:** 

1. Stromata occurring on dead corticated or decorticated wood	**2**
1’. Stromata occurring on woody fruits	**27**
2. Stromata with a horny, gray to tan outer layer covering the apex, splitting below into elongated stripes or polygonal scales	**3**
2’. Stromata with an outer layer of different color and texture	**5**
3. Stromata subglobose to depressed-spherical; ascospores 44.9 × 13.5 µm, with a spiraling germ slit	***X. oxycephala*** *
3’. Stromata cylindrical to subfusiform; ascospores much smaller, with a straight germ slit	**4**
4. Stromatal crust 80–120 µm thick; ascospores 12.6 × 5.0 µm, with a short germ slit 6–9 µm long	***X. arbuscula***
4’. Stromatal crust 70–80 µm thick; ascospores 11.3 × 4.8 µm, with a germ slit almost spore-length	***X. partita***
5. Stromata hard-textured to brittle, with a carbonaceous crust	**6**
5’.Stromata with a leathery crust	**16**
6. Stromata with a carbonaceous crust exceeding 80 µm thick	**7**
6’. Stromata slightly carbonaceous, with a crust ca. 80 µm thick or less	**12**
7. Stromatal interior partly to entirely sooty brown, dark gray or blackish	**8**
7’. Stromatal interior whitish or grayish	**10**
8. Stromata with a white striped outer layer; ascospores 10.8 × 4.6 µm, with a sigmoid germ slit	***X. sinuosirima*** *
8’. Stromata with a brown to dark brown outer layer; ascospores with a straight germ slit	**9**
9. Stromatal outer layer reticulately cracked, lacking superficial carbonaceous granules; ostioles sharply conical, on a discoid base120–170 µm diam; ascospores 10.5 × 4.5 µm	***X. adscendens***
9’. Stromatal outer layer splitting into elongated stripes, bearing superficial carbonaceous granules, with the surface transversely striated in places; ostioles inconspicuous; ascospores 10.5 × 4.4 µm	***X. multiplex***
10. Stromata with an ellipsoid to fusiform fertile head on a long filiform glabrous stipe; ascospores 41.9 × 7.7 µm, with a dorsal spiraling germ slit	***X. comosoides***
10’. Stromata narrowly fusiform to subclavate; ascospores smaller, with a ventral germ slit	**11**
11. Stromatal surface gray, with olivaceous to purple markings; carbonaceous crust 150–170 µm thick; ascospores 9.3 × 4.2 µm, with straight germ slit	***X. vinosa***
11’. Stromatal surface white to cream-colored; carbonaceous crust 120–150 µm thick; ascospores 28.8 × 7.2 µm, with a spiraling germ slit	***X. kegeliana***
12. Ostioles broad and prominent, 120–210 µm diam; ascospores with a short germ slit 1/2 to 2/3 spore-length	**13**
12’. Ostioles inconspicuous; ascospores with a germ slit almost spore-length	**14**
13. Stromata with a crust 40–50 µm thick, overlain with a dirty gray, felty outer layer, heavily encrusted with carbonaceous granules on the surface; ascospores 16.4 × 5.5 µm (Qe = 3.0)	***X. piloides*** *
13’. Stromata with a crust 70–80 µm thick, overlain with a silvery gray outer layer, lacking carbonaceous granules on the surface; ascospores narrower, 15.8 × 4.7 µm	***X. exechostoma*** *
14. Stromatal interior sooty brown to blackish; ascospores 10.5 × 4.4 µm	***X. multiplex***
14’. Stromatal interior white, with a narrow gray or pale orange core	**15**
15. Ascospores broadly ellipsoid, 8.6 × 4.1 µm	***X. pallidocylindracea***
15’. Ascospores narrowly fusiform, 18.1 × 4.4 µm	***X. atractoidosperma*** *
16. Ascospores greater than 19 µm long	**17**
16’. Ascospores much shorter	**19**
17. Stromatal surface faintly cerebriform; ascospores 21.0 × 6.4 µm, with a dorsal germ pore	***X. rickii***
17’. Stromatal surface nodulose; ascospores with a ventral germ slit	**18**
18. Stromatal interior gray to dark gray; ascospores narrowly fusiform, light brown, 23.3 × 4.6 µm	***X. stupenda*** *
18’. Stromatal interior white to yellowish; ascospores broadly fusiform, dark brown, 19.7 × 5.8 µm	***X. scabra*** *
19. Ascospores less than 8 µm long	**20**
19’. Ascospores 8.5–10 µm long	**22**
20. Ascospores 6.5 × 3.5 µm, with broadly rounded ends	***X. candelabroides*** *
20’. Ascospores slightly larger, with narrowly rounded ends	**21**
21. Stromata with a white to cream-colored outer layer and a gray to blackish interior; ascospores 7.3 × 3.0 µm, light brown, with obtuse ends	***X. corriolii*** *
21’. Stromata with a silvery gray outer layer and a whitish interior; ascospores 7.3 × 3.3 µm, dark brown, with narrowly rounded, slightly pinched ends	***X. gigartosperma*** *
22. Stromatal surface lacking an outer layer	**23**
22’. Stromatal surface coated with an outer layer	**24**
23. Stromata cylindrical, with a plane surface and a tapering apex; ascospores 8.7 × 3.3 µm, light brown, ellipsoid-equilateral	***X. myosurus***
23’. Stromata ramified, with a nodulose surface and a spiny apex; ascospores 9.0 × 3.4 µm, brown, ellipsoid-inequilateral	atypical form of ***X. oligotoma***
24. Stromata nodulose, with strongly exposed perithecial contours, lacking superficial carbonaceous granules	**25**
24’. Stromata plane to slightly nodulose, densely granulated with clusters of superficial carbonaceous granules	**26**
25. Stromatal surface overlain by a thick bipartite gray and yellow outer layer splitting into large plaques and stripes; interior white with orange-brown strands; ascospores 10.0 × 3.8 µm	***X. coccophora***
25’. Stromatal surface initially overlain by a thin, fleeting silvery gray outer layer splitting into narrow stripes; interior partly black to blackish; ascospores 9.0 × 3.4 µm	***X. gracillima***
26. Stromatal surface plane, with a gray to white striped outer layer; ascospores 9.5 × 3.9 µm, ventrally flat	***X. nigrosticta*** *
26’. Stromatal surface nodulose, with a brownish gray, tan to orange-brown outer layer; ascospores 8.7 × 3.4 µm, frequently suballantoid	***X. oligotoma***
27. Stromata cylindrical to strap-like, densely tomentose, with a leathery crust 20–60 µm thick, occurring on woody fruits	**28**
27’. Stromata long-stipitate, with a globose to ellipsoid fertile head and a slightly carbonaceous crust 60–90 µm thick, occurring on leaf petioles	**31**
28. Ascospores light brown, narrowly fusiform-inequilateral	**29**
28’. Ascospores dark brown, ellipsoid-inequilateral	**30**
29. Stromata 10–36 mm high; ascospores 9.2 × 3.2 µm, with a straight germ slit almost spore-length; on dead fruits of *Apeiba*	***X. apeibae***
29’. Stromata 22–100 mm high; ascospores 18.7 × 4.9 µm, with an oblique to slightly sigmoid germ slit; on dead pods	***X. rossmaniae***
30. Ascospores 10.0 × 4.0 µm, with a germ slit central, less than to slightly less than spore-length, with bipolar secondary appendages; on dead pods	***X. ianthinovelutina***
30’. Ascospores 10.1 × 4.1 µm, with a germ slit long or short often located toward one end, lacking bipolar secondary appendages; on dead pods	***X. siliquarum*** *

### Key C Stromata upright, apically fertile and rounded, with a carbonaceous crust

**Table:** 

1. Stromatal surface in shades of yellow to orange-brown, with a white granular subsurface layer and an outer layer yielding yellowish pigments in 10% KOH	**2**
1’.Stromatal surface white, gray, brown, or blackish, lacking a white subsurface layer and pigments in 10% KOH	**5**
2. Stromatal surface cinnamon to orange-brown; ascospores blackish brown, with a short straight germ slit	**3**
2’. Stromatal surface yellowish; ascospores dark brown, with a short oblique germ slit	**4**
3. Stromatal stipe darkened by a dark brown tomentum; ascospores 16.3 × 6.1 µm	***X. hyperythra***
3’.Stromatal stipe glabrous; ascospores 19.5 × 6.6 µm	***X. columnifera***
4. Stromatal interior whitish to tan, solid; ascospores 18.3 × 6.5 µm	***X. enterogena***
4’. Stromatal interior gelatinous, hollow and filled with liquid when fresh; ascospores 19.6 × 6.9 µm	***X. telfairii***
5. Stromata apically acute or bearing outgrowths	**6**
5’. Stromata with evenly rounded apices	**8**
6. Stromata narrowly clavate to cylindrical; ostioles in linear rows; ascospores 11.9 × 4.4 µm, with a straight ventral germ slit	***X. grammica***
6’. Stromata with a subglobose to ellipsoid fertile head on a filiform stipe; ascospores much larger, with a dorsal germ slit	**7**
7. Stromatal stipe hairy; ascospores 30.4 × 7.8 µm, with a straight germ slit	***X. comosa***
7’. Stromatal stipe glabrous; ascospores 41.9 × 7.7 µm, with a spiraling germ slit	***X. comosoides***
8. Stromatal surface white to grayish	**9**
8’. Stromatal surface brown, blackish brown to blackish	**14**
9. Stromata cylindric, sessile, with a pure white pruinose to fibrous outer layer, only faintly carbonaceous; ascospores 7.1 × 3.0 µm, light brown, with a short germ slit	***X. muscula***
9’. Combination of characteristics different	**10**
10. Stromatal surface overlain with a thin, reticulately cracked whitish pellicle; ascospores 21.6 × 7.5 µm, with slightly pinched ends	***X. macouriana*** *
10’. Combination of characteristics different	**11**
11. Stromatal outer layer gray, with superficial blackish granules; ostioles in linear rows; ascospores 11.9 × 4.4 µm, with a straight ventral germ slit	***X. grammica***
11’. Stromatal outer layer splitting into white to gray scales; ostioles scattered	**12**
12. Stromata with a subglobose fertile head 2.5–4.5 mm diam; ascospores 33.2 × 12.7 µm, with a dorsal germ slit	***X. squamulosa***
12’. Stromata with a cylindrical to clavate fertile head; ascospores much smaller, with a ventral germ slit	**13**
13. Stromatal crust 80–100 µm thick; ostioles conspicuous, on a discoid base 80–120 µm diam; ascospores brown, 9.1 × 3.9 µm, with narrowly rounded ends	***X. curta***
13’. Stromatal crust 150–200 µm thick; ostioles inconspicuous, lacking a discoid base; ascospores dark brown, 9.3 × 4.2 µm, with mostly broadly rounded ends	***X. guianica*** *
14. Stromatal surface blackish, corky-cracked; ascospores 23.0 × 7.1 µm, with a short oblique germ slit	unusual carbonaceous forms of ***X. schweinitzii***
14’. Stromatal surface plane, not cracked into conspicuous scales	**15**
15. Stromata globose to subglobose, subsessile, with a blackish surface and a vinaceous interior; ascospores 25.8 × 6.8 µm	***X. collabens***
15’. Stromata cylindrical to clavate, with a whitish or blackish interior; ascospores smaller	**16**
16. Stromatal interior blackish	**17**
16’. Stromatal interior whitish	**18**
17. Stromatal crust 40–60 µm thick; ascospores blackish brown, broadly ellipsoid, 7.4 × 4.2 µm, with an inconspicuous germ slit spore-length	***X. nigromedullosa***
17’. Stromatal crust 80–100 µm thick; ascospores brown, fusiform, 13.4 × 4.5 µm, with a conspicuous germ slit much less than spore-length	***X. cylindrica***
18. Stromata cylindrical, with a pannose or tomentose base	**19**
18’. Stromata narrowly to broadly clavate or fusiform, lacking a pannose base	**20**
19. Stromata unbranched, with a copper-brown, finely cracked surface; ascospores 13.1 × 5.9 µm, with broadly rounded ends and a germ slit almost spore-length	***X. crassipes*** *
19’. Stromata frequently forked, with a black surface; ascospores 13.6 × 5.5 µm, with narrowly rounded ends and a germ slit 1/2–2/3 spore-length	***X. robusta*** *
20. Stromatal surface reticulately cracked, forming wide polygonal ostiolar discs; ascospores 16.6 × 5.0 µm	***X. guyanensis***
20’. Stromatal surface lacking polygonal ostiolar discs; ascospores shorter	**21**
21. Ascospores 7.8 × 3.9 µm, subequilateral, with narrowly rounded ends and a conspicuous germ slit almost spore-length	***X. cubensis***
21’. Ascospores 8.8 × 4.1 µm, inequilateral, with less narrowly rounded ends and a short inconspicuous germ slit	***X. flabelliformis***

### Key D Stromata upright, apically fertile and rounded, with a thin leathery crust

**Table:** 

1. Stromatal surface corky-cracked, with thick scales or warts	**2**
1’. Stromatal surface plane, lacking thick scales or warts	**12**
2. Ascospores less than 10 µm long on average	**3**
2’. Ascospores 15–25 µm long on average	**8**
3. Stromata turbinate, with a slightly convex to flat apex, associated with coremia	**4**
3’. Stromata clavate, fusoid-cylindrical, or with a subglobose fertile head, lacking coremia	**5**
4. Stromata with perithecia embedded in a citrine-yellow subsurface layer; ostioles 210–290 µm diam, ascospores 9.3 × 4.8 µm	***X. palifera*** *
4’. Stromata with an entirely white interior; ostioles 120–170 µm diam, ascospores 9.3 × 5.1 µm	***X. heliscus***
5. Stromatal stipe smooth, uncracked	**6**
5’. Stromatal stipe corky-cracked	**7**
6. Stromatal stipe glabrous; ascospores 8.6 × 4.5 µm	**X. rhytidophloea**
6’. Stromatal stipe more or less hairy-downy, at least at base; ascospores 8.6 × 4.6 µm	***X. crustulata*** *
7. Stromata hairy on lower half of the stipe, with perithecia embedded in a citrine-yellow subsurface layer; ascospores 8.2 × 4.3 µm	***X. uncinata*** *
7’. Stromata glabrous, corky-cracked on the entire stipe, with an entirely white interior; ascospores 8.9 × 4.3 µm	***X. subplebeja*** *
8. Ascospores less than 20 µm long on average	**9**
8’. Ascospores more than 20 µm long on average	**10**
9. Ascospores 19.4 × 6.5 µm, with an oblique to slightly sigmoid germ slit ca. 1/2 spore-length	***X. scruposa***
9’. Ascospores 14.9 × 5.9 µm, with a sigmoid germ slit ca. 2/3 spore-length small-spored form of	***X. scruposa***
10. Stromatal surface covered with small gray brown scales; ostioles 100–170 µm diam, not prominent; ascospores 23.0 × 7.1 µm	***X. schweinitzii***
10’. Stromatal surface covered with thick warts; ostioles prominent, 180–250 µm diam; ascospores larger, ca. 25 × 7.9 µm	**11**
11. Stromatal interior with orange markings in the stipe; ostioles discoid; ascospores 25.0 × 7.9 µm, with a short diagonal germ slit	***X. globosa***
11’. Stromatal interior lacking orange markings; ostioles dome-shaped; ascospores 25.2 × 8.0 µm, with a short oblique, less slanted germ slit	***X. haemorrhoidalis***
12. Stromata coated with a pure white pruinose to fibrous outer layer	**13**
12’. Stromata brown to blackish	**14**
13. Ascospores 7.1 × 3.0 µm	***X. muscula***
13’. Ascospores 10.6 × 3.7 µm	***X. microceras***
14. Stromata with a fleeting to persistent peeling outer layer on the fertile head	**15**
14’. Stromata lacking a peeling outer layer on the fertile head	**20**
15. Stromatal fertile head nodulose to cerebriform when dry	**16**
15’. Stromatal fertile head lacking exposed perithecial contours	**18**
16. Stromatal fertile head cerebriform, with a golden-yellow outer layer; ascospores 9.0 × 4.1 µm	***X. orichalcea*** *
16’. Stromatal fertile head nodulose, with a grayish outer layer	**17**
17. Stromata less than 12 mm high × 2 mm wide; ascospores 8.2 × 3.7 µm, with a consistently ventral germ slit	***X. minor*** *
17’. Stromata 7–32 mm high × 1.5–4.2 mm wide; ascospores 9.3 × 3.8 µm, with a frequently dorsal germ slit	***X. spegazziniana*** *
18. Stromata narrowly cylindrical, 1.3–2.6 mm wide; ascospores subequilateral, 5.6 × 2.9 µm	***X. parvispora*** *
18’. Stromata clavate to fusiform, 3–12 mm wide; ascospores inequilateral, larger	**19**
19. Stromatal outer layer splitting into persistent grayish white scales; ascospores ellipsoid-inequilateral, brown to dark brown, 8.4 × 4.0 µm	***X. scutulata*** *
19’. Stromatal outer layer a thin, appressed, whitish pellicle; ascospores narrowly fusiform, medium brown, 13.7 × 3.8 µm	***X. saulensis*** *
20. Ascospores less than 12 µm long	**21**
20’. Ascospores greater than 12 µm long	**24**
21. Stromata dichotomously branched; ascospores 10–11 × 4.5–5 µm	***X. ruginosa***
21’. Stromata unbranched	**22**
22. Ascospores 7.7 × 4.1 µm, blackish brown, with broadly rounded ends	***X. confragosa*** *
22’. Ascospores 8.9 µm long on average	**23**
23. Ascospores 8.9 × 3.6 µm, light brown to brown, with narrowly rounded ends	***X. bubalina*** *
23’. Ascospores 8.9 × 4.5 µm, blackish brown, with broadly rounded ends	***X. holmbergii***
24. Ascospores 30–40 × 5–8 µm	***X. aenea***
24’. Ascospores less than 20 µm long	**25**
25. Ascospores 14.4 × 4.9 µm	***X. cuneata***
25’.Ascospores 18.8 × 6.4 µm	***X. penicilliopsis***

### Key E Stromata terrestrial or leaf-inhabiting

**Table:** 

1. Stromatal terrestrial or arising from buried seeds	**2**
1’. Stromata occurring on leaves or petioles	**3**
2. Stromata filiform, arising from buried palm seeds; perithecia 0.3–0.4 mm diam, aggregated into a narrowly cylindrical, densely tomentose fertile head; ascospores 11.2 × 4.9 µm	***X. rhizocola***
2’. Stromata filiform, arising from soil, likely associated with termite nests; perithecia superficial, scattered to clustered, 0.4–0.5 mm diam, glabrous; ascospores 4.5–5 × 2–2.5 µm	***X. rhizomorpha***
3. Stromata capitate, with a subglobose to ellipsoid fertile head terminating into an acicular apex	**4**
3’. Stromata with a narrowly cylindrical to subclavate fertile head terminating into a blunt or mucronate apex	**9**
4. Ascospores shorter than 15 µm	**5**
4’. Ascospores significantly longer	**6**
5. Ascospores 11.5 × 6.5 µm, with narrowly rounded ends	**X. aristata var. aristata**
5’. Ascospores 11.3 × 6.6 µm, with broadly rounded ends, darker brown, more strongly inequilateral	***X. delicatula***
6. Stromatal fertile head subglobose; ascospores with a straight dorsal germ slit	**7**
6’. Stromatal fertile head ellipsoid; ascospores with a spiraling germ slit	**8**
7. Ascospores 22.0 × 5.6 µm	***X. axifera***
7’. Ascospores 31.3 × 7.3 µm	***X.*** **sp. cf.** ***X. axifera***
8. Ascospores 18.7 × 6.7 µm, fusiform-inequilateral, lacking bipolar appendages	***X. melaniae*** *
8’. Ascospores 21.7 × 5.8 µm, fusiform, equilateral to nearly so, with bipolar appendages	***X. petiolorum*** *
9. Ascospores 25.1 × 9.7 µm	***X. spiculaticlavata***
9’. Ascospores much smaller	**10**
10. Ascospores 13.1 × 7.2 µm with polar appendages	***X. appendiculata***
10’. Ascospores 10.6 × 6.0 µm lacking appendages	***X. phyllocharis***

### Species that were recently collected from French Guiana

***Xylaria adscendens*** (Fr.) Fr., in Fr., Nov. Act. Reg. Soc. Sci. Upsal., ser. III, 1: 128. 1851.

= *Sphaeria adscendens* Fr., Linnaea 5: 537. 1830.

The cited collection from French Guiana closely matches those reported and illustrated from the French West Indies (Fournier et al. 2020).

**Specimen examined: FRENCH GUIANA.** Roura, Cacao, Camp Bonaventure, 4.323044 N, 52.339371 W, 59 m, disturbed hygrophilic rainforest, on dead trunk, 20 Mar 2021, *Fournier, J. GYJF21016* (HAST 147388).

***Xylaria alboareolata*** Y.-M. Ju & J. D. Rogers, N. Amer. Fung. 7(9): 18. 2012.

≡ *Hypoxylon areolatum* Berk. & M. A. Curtis, in Berk., J. Linn. Soc., Bot. 10: 384. 1869; non Starb., 1905.

≡ *Penzigia areolata* (Berk. & M. A. Curtis) J. H. Miller, Monogr. of World Species of *Hypoxylon*, p. 132. 1961.

≡ *Xylaria areolata* (Berk. & M. A. Curtis) Y.-M. Ju & J. D. Rogers, Mycotaxon 73: 398. 1999.

The cited collection from French Guiana closely matches those reported and illustrated from the French West Indies (Fournier et al. 2018c).

**Specimen examined: FRENCH GUIANA.** Macouria, vicinity of Cayenne, Risquetout Forest, trail to Saut-Léodate, 4.899372 N, 52.555061 W, ca. 55 m, lowland rainforest, on dead branch, 1 May 2007, *Lechat, C. CLL7097* (JF).

**Fig. 2:**
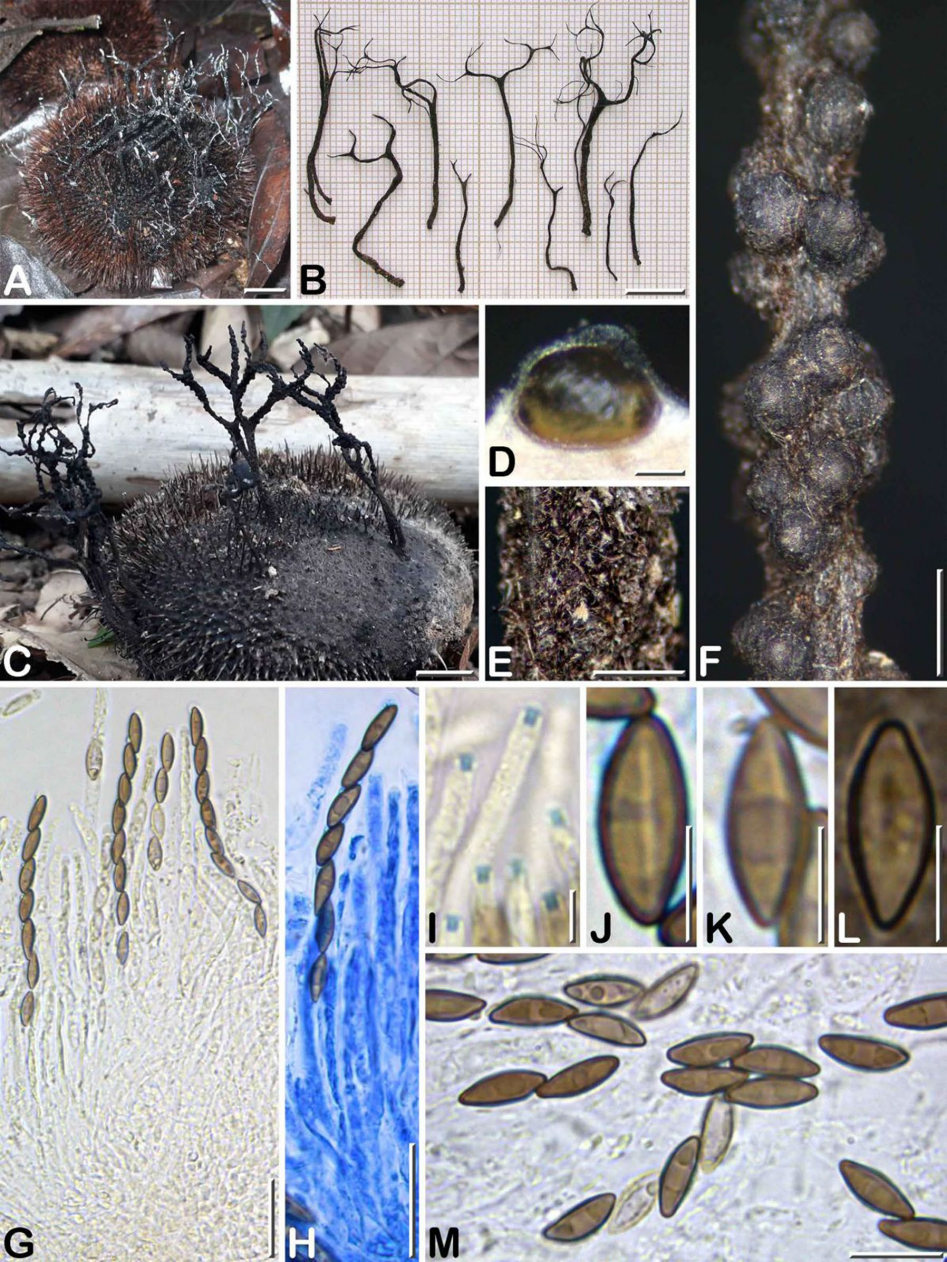
*Xylaria apeibae* (**A**, **B** from *GYJF21151*, **C** from *GYJF18080*, **D–M** from *GYJF19071*). **A** Immature stromata at anamorphic state on a dead fruit of *Apeiba*. **B** Branched immature stromata. **C** Mature stromata on a dead fruit of *Apeiba* (photo by A. Gardiennet). **D** Half-immersed perithecium in vertical section. **E** Tomentose stipe in close-up. **F** Stromatal surface showing strongly exposed perithecial contours and tomentum. **G** Asci in 1% SDS. **H** Seven-spored ascus in blue Pelikan ink. **I** Ascal apical apparati in Melzer’s reagent. **J, K** Ascospores showing a germ slit, respectively in blue Pelikan ink and 1% SDS. **L** Ascospore in India ink showing absence of sheath or appendage. **M** Mature and immature ascospores in 1% SDS. Bars in **A–C** = 10 mm; **D** = 100 µm; **E, F** = 0.5 mm; **G, H** = 20 µm; **I–L** = 5 µm; **M** = 10 µm

***Xylaria apeibae*** Mont., Ann. Sci. Nat. Bot., sér. IV, 3:105. 1855. Figure 2, Table 2

**Table 2:** Ascospore dimensions in three collections of *X. apeibae* showing a wide range of intraspecific variations, compared with those reported from the holotype of *X. apeibae* and that of *X. luzonensis*

Collections numbers	Ascospore measurements with extreme values in parentheses	Q = quotient l/w, *N* = number of measurements	Me = mean values of l/w, Qe = mean quotient l/w
*GYJF18080*	(7.5–)8.0–9.3(−9.6) × (2.8–)3.0–3.6(−3.8) µm	Q = (2.2–)2.3–2.9(−3.0), *N* = 60	Me = 8.6 × 3.3 µm, Qe = 2.6
*GYJF19071*	(7.9–)9.1–10.2(−10.7) × (2.8–)3.0–3.6(−3.7) µm	Q = (2.5–)2.7–3.2(−3.5), *N* = 60	Me = 9.6 × 3.3 µm, Qe = 2.9
*GYJF19224*	(8.5–)8.7–10.0(−10.7) × (2.4–)2.7–3.2(−3.4) µm	Q = (2.7–)2.9–3.5(−3.9), *N* = 60	Me = 9.5 × 3.0 µm, Qe = 3.1
**Cumulated values**	**(7.5–)8.0–10.2(−10.7) × (2.4–)2.7–3.6(−3.8) µm**	**Q = (2.2–)2.3–3.5(−3.9), N = 180**	**Me = 9.2 × 3.2 µm, Qe = 2.9**
*X. apeibae* holotype (Ju et al. 2018)	(9.5–)10–12(−13) × (3–)3.5–4(−4.5) μm	*N* = 40	11.0 ± 0.9 × 3.7 ± 0.2 μm
*X. luzonensis* neotype (Ju et al. 2018)	(8–)8.5–9.5(−10) × 3–3.5(−4) μm	*N* = 40	8.9 ± 0.4 × 3.4 ± 0.2 μm

**Stromata** upright, scattered, isolated to connate at base, cylindrical to slightly flattened, simple to dichotomously branched 1–3 times, 10–36 mm in total height × 0.8–1.9 mm diam, with apiculate, fragile sterile apices; the stipes ill-defined, cylindrical to flattened, black, densely hairy-tomentose, the base slightly enlarged; surface blackish, nodulose with strongly exposed perithecial contours, with persistent reddish brown tomentum between isolated perithecial contours, lacking a peeling outer layer; subsurface crust leathery, 17–25 µm thick; interior white, solid, pithy. **Perithecia** subglobose to depressed-spherical, 0.15–0.20 mm diam. **Ostioles** black, obtusely papillate most often inconspicuous.

**Paraphyses** hyphal, hyaline, remotely septate, simple to branched at base, 4–5 µm wide at base, tapering to 1.0–1.5 µm wide, discreetly embedded in mucilaginous material. **Asci** cylindrical, with (6–)8 uniseriately arranged, slightly overlapping ascospores, the spore-bearing parts 56–68 µm long × 4.0–4.6 µm wide, the stipes 40–55 µm long, with apical apparatus short tubular with a faint upper rim, 1.5–1.8 × 1.0–1.3 µm (Me = 1.6 × 1.1 µm, *N* = 25), bluing in Melzer’s reagent. **Ascospores** (7.5–)8.0–10.2(−10.7) × (2.4–)2.7–3.6(−3.8) µm, Q = (2.2–)2.3–3.5(−3.9) *N* = 180 (Me = 9.2 × 3.2 µm, Qe = 2.9), fusiform-inequilateral with narrowly rounded to subacute ends, light brown, with a straight to slightly oblique germ slit slightly less than spore-length; lacking sheath or appendages; epispore smooth.

**Asexual morph** present as a white evanescent coating on tips of ramified immature stromata.

**Specimens examined: FRENCH GUIANA.** Maripasoula, Saül, way up to Hmong village, 3.625591 N, 53.208009 W, ca. 220 m, disturbed rainforest, on dead fruits of *Apeiba* sp. (Tiliaceae) in the leaf litter, 19 Jun 2019, *Fournier, J. GYJF19107* (HAST 147202), immature; Maripasoula, Saül, trail head to Roche-Bateau, edge of the airfield, 3.620754 N, 53.200605 W, ca. 220 m, disturbed rainforest, on dead fruits of *Apeiba* sp. in the leaf litter, 23 Jun 2019, *Fournier, J. GYJF19224* (HAST 147203); Maripasoula, Saül, trail head toward Sentier des Gros Arbres, 3.620324 N, 53.207951 W, ca. 210 m, disturbed rainforest, on dead fruits of *Apeiba* sp. in the leaf litter, 18 Jun 2019, *Fournier, J. GYJF19071* (HAST 147204); Maripasoula, Saül, trail head toward Sentier des Gros Arbres, 3.620839 N, 53.208536 W, ca. 210 m, disturbed rainforest, on dead fruits of *Apeiba* sp. in the leaf litter, 26 Mar 2021, *Fournier, J. GYJF21151* (HAST 147205), immature; Maripasoula, Saül, trail head toward Sentier des Gros Arbres, 3.620589 N, 53.208547 W, ca. 210 m, disturbed rainforest, on dead fruits of *Apeiba* sp. in the leaf litter, 22 Aug 2018, *Gardiennet, A. GYJF18080* (HAST 147206); Maripasoula, Saül, way up to Hmong village, 3.6255 N, 53.208254 W, ca. 210 m, disturbed rainforest, on dead fruits of *Apeiba* sp. in the leaf litter, 21 Aug 2018, *Gardiennet, A. GYJF18055* (HAST 147207), immature; vicinity of Cayenne, on fruits of *Apeiba* sp., *Leprieur, F. R. 1207* (holotype PC 0096735 ex Montagne herb. 10088).

**Known distribution**: Brazil, French Guiana (Ju et al. 2018).

**Comments**: *Xylaria apeibae* is readily recognized in the field by its small, filiform, branched, and nodulose stromata arising from the distinctive urchin-like fruits of *Apeiba* sp. embedded in the leaf litter. The morphology of our specimens conforms well to the descriptions provided by Montagne (1855) and Ju et al. (2018) based on the type material, but our ascospore measurements reveal a notable difference (Table 2), suggesting considerable variation in size and shape within this species. Differences in ascospore dimensions were regarded by Ju et al. (2018) as sufficient to distinguish *X*. *apeibae* from *X. luzonensis* Henn.; however, this distinction becomes less clear when our data are taken into account.

***Xylaria arbuscula*** Sacc., Michelia 1: 249. 1878.

The cited collection from French Guiana closely matches those reported and illustrated from the French West Indies (Fournier et al. 2020).

**Specimen examined: FRENCH GUIANA.** Sinnamary, Paracou, CIRAD field centre, Guyaflux plot, 5.249218 N, 52.915431 W, lowland rainforest, on dead corticated branch, 25 Jun. 2012, *Fournier, J. GYJF 12195* (JF).

**Fig. 3:**
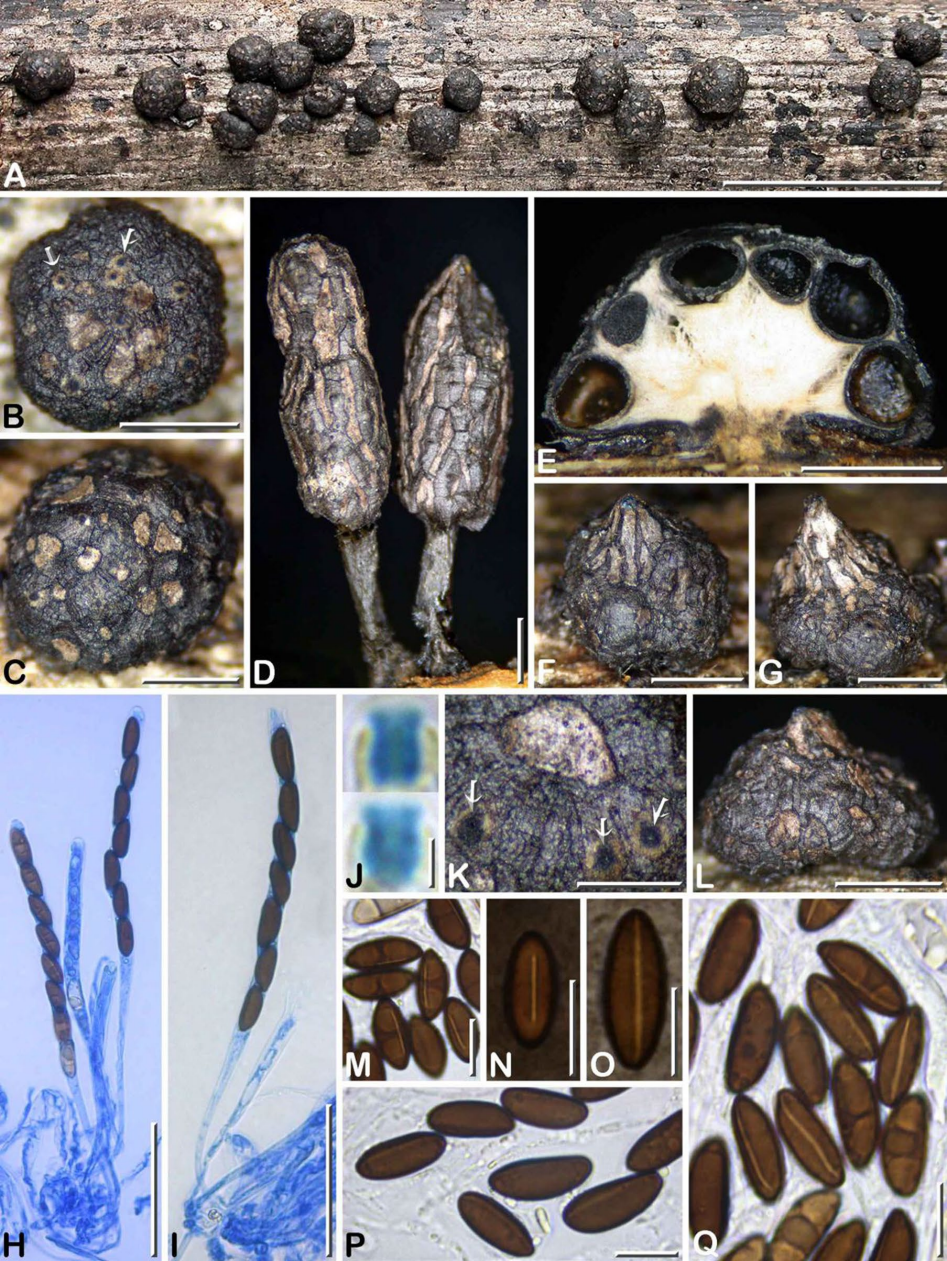
*Xylaria asperula* (**A–C**, **E–L**, **O–Q** from *GYJF19049*) and the *X. arbuscula* part intermixed with it (**D, M, N**). **A** Stromata scattered on bark. **B** Flat-topped stroma in top view, showing ostioles (arrows). **C** Hemispherical stroma in side view. **D** Two adjacent upright stromata of *X. arbuscula*. **E** Hemispherical sessile stroma in vertical section showing a short central connective. **F, G, L** Apically pointed stromata in side view. **H, I** Seven- and eight-spored asci in blue Pelikan ink. **J** Ascal apical apparati in Melzer’s reagent. **K** Stromatal surface in close-up showing a horny scale and ostioles (arrows) on a finely grooved black background. **M, P, Q** Ascospores in 1% SDS showing germ slits. **N, O** Ascospores in India ink showing a germ slit and absence of sheath or appendage. Bars in **A** = 10 mm; **B–G, L** = 1 mm; **H–I** = 50 µm; **J** = 2 µm; **K** = 0.5 mm; **M–Q** = 10 µm

***Xylaria asperula*** (Starb.) J. Fourn., Y.-M. Ju, H.-M. Hsieh & Lechat, comb. nov. Figure 3, Table 3

**Table 3:** Ascospore and ascal apical apparatus dimensions in two collections of *X. asperula* from French Guiana, compared with those of a collection of *X. arbuscula* var. *plenofissura* from Taiwan, those of a collection of *X. arbuscula* represented by two stromata (Fig. 3D) intermixed with *GYJF19049*, and the dimensions given in the protologue of *X. juniperus* var. *asperula*

Collection numbers	Ascospore measurements with extreme values in parentheses	Q = quotient l/w, *N* = number of measurements	Me = mean values of l/w, Qe = mean quotient l/w	Ascal apical apparatus h × w
*GYJF18006*	(13.1–)14.8–17.6(−20.3) × (5.5–)5.8–6.8(−7.1) µm	Q = (2.0–)2.3–2.8(−3.1), *N* = 60	Me = 16.1 × 6.3 µm, Qe = 2.6	
*GYJF19049*	(13.9–)14.3–16.3(−17.5) × (5.3–)5.6–6.4(−7.0) µm	Q = (2.2–)2.4–2.7(−2.9), *N* = 60	Me = 15.3 × 6.0 µm, Qe = 2.5	3.1–3.5 × 2.1–2.6 µm, Me = 3.3 × 2.3 µm, *N* = 25
*X. arbuscula* var. *plenofissuraYMJ 90071618*	(14.4–)15.5–17.5(−18.8) × (5.4–)6.1–7.3(−7.7) µm	Q = (2.0–)2.2–2.7(−3.1), *N* = 60	Me = 16.4 × 6.6 µm, Qe = 2.5	2.9–3.8 × 2.0–2.7 µm, Me = 3.4 × 2.3 µm, *N* = 25
*X. arbuscula GYJF19049-2*	(11.2–)12–13.4(−14.2) × (4.3–)5.1–6.0(6.2) µm	Q = (2.0–)2.1–2.5(−3.1), *N* = 60	Me = 12.7 × 5.5 µm, Qe = 2.3	
*X. juniperus* var. *asperula* (Starbäck 1901)	15–18 × 6–7 µm		Me = 16.5 × 6.5 µm, Qe = 2.5	
**Cumulated values**	**(13.1–)14.3–17.6(−20.3) × (5.3–)5.6–6.8(−7.1) μm**	**Q = (2.0–)2.3–2.8(−3.1), N = 120**	**Me = 15.7 × 6.2 μm, Qe = 2.6**	

MycoBank MB 863182

Basionym: *Xylaria juniperus* Starb. var. *asperula* Starb., Bih. Kongl. Svenska Vetensk.-Akad. Handl. 27, Afd. III (9): 20. 1901.

**Stromata** widespread, erumpent from bark, isolated or in small groups, rarely in contact, mostly pulvinate to subglobose, convex or flat-topped, less frequently hemispherical and convex or subglobose with a pointed apex, 0.9–2.1(−2.9) mm high × 1.5–3.1 mm diam, sessile, on a narrow central connective; surface black, perithecial contours slightly exposed or unexposed, finely grooved in parallel ridges, with a gray to tan horny outer layer splitting from the center into thick scales or stripes, persistent; subsurface crust 60–80(−100) µm hick, slightly carbonaceous; interior white, loosely fibrous. **Perithecia** subglobose, 0.55–0.80 mm diam. **Ostioles** flush with the surface to barely papillate, 60–80 µm diam, often inconspicuous unless they are surrounded by a thin tan flake ca 200 µm diam.

**Paraphyses** hyphal, thin-walled, 5–6 µm wide at base, tapering to 1–1.5 µm wide above asci, sparsely and minutely guttulate, embedded in mucilaginous material. **Asci** cylindrical, with (6–)8 slightly overlapping uniseriately arranged ascospores, the spore-bearing parts 99–108 × 6.0–7.0) µm, the stipes 95–110 µm long, with apical apparatus 3.1–3.5 × 2.1–2.6 µm (Me = 3.3 × 2.3 µm, *N* = 25), tubular to slightly urn-shaped with an obtuse upper rim, bluing in Melzer’s reagent. **Ascospores** (13.9–)14.3–16.3(−17.5) × (5.3–)5.6–6.4(−7.0) µm, Q = (2.2–)2.4–2.7(−2.9), *N* = 60 (Me = 15.3 × 6.0 µm, Qe = 2.5), ellipsoid-inequilateral with broadly to less frequently narrowly rounded ends, brown to dark brown, unicellular, with a conspicuous, straight to oblique or sinuous germ slit almost spore-length on the ventral side, rarely on the dorsal side; lacking sheath or appendages; epispore smooth.

**Asexual morph** on the natural substrate not observed.

**Specimens examined: FRENCH GUIANA.** Maripasoula, Saül, village, edge of the forest next to Carbets du bord, 3.623598 N, 53.210338 W, disturbed secondary rainforest, on dead corticated liana 1.5–1.8 cm diam, 19 Aug 2018, *Fournier, J. GYJF18006* (HAST 147211); Maripasoula, Saül, shortcut to the airfield, 3.623524 N, 53.206542 W, 210–220 m, disturbed secondary rainforest, on dead corticated liana ca 1.5 cm diam, associated with *Stilbohypoxylon quisquiliarum*, 17 Jun 2019, *Fournier, J. GYJF19049* (HAST 147212).

**Known distribution**: French Guiana, known from two collections; Paraguay (Starbäck 1901).

**Comments**: Both collections of this fungus are abundant and consistently feature small penzigioid stromata with a scaly to striped surface typical of species related to *X. arbuscula* Sacc., a placement supported by our phylogenetic analyses (Fig. 1), which show that it is nested in a subclade containing *X. arbuscula*, *X. arbuscula* var. *plenofissura, X. partita*, and *X. venosula*, but distinct from them. We propose to accommodate it as *X. asperula* rather than as *X. juniperus* Starb. var. *asperula* Starb., a variety of *X. juniperus* Starb. *Xylaria juniperus* is regarded as a synonym of *X. arbuscula* based on stromatal configuration and ascospores 12–15 × 5–6 µm.

Four minute stipitate stromata, 6–11 mm high, are mixed within *GYJF19049* and could have been interpreted as an upright form in addition to the penzigioid ones. This was ruled out by their smaller ascospores (Fig. 3M, N) with a short germ slit, consistent with those of typical *X. arbuscula* (Fournier et al. 2020).

**Fig. 4:**
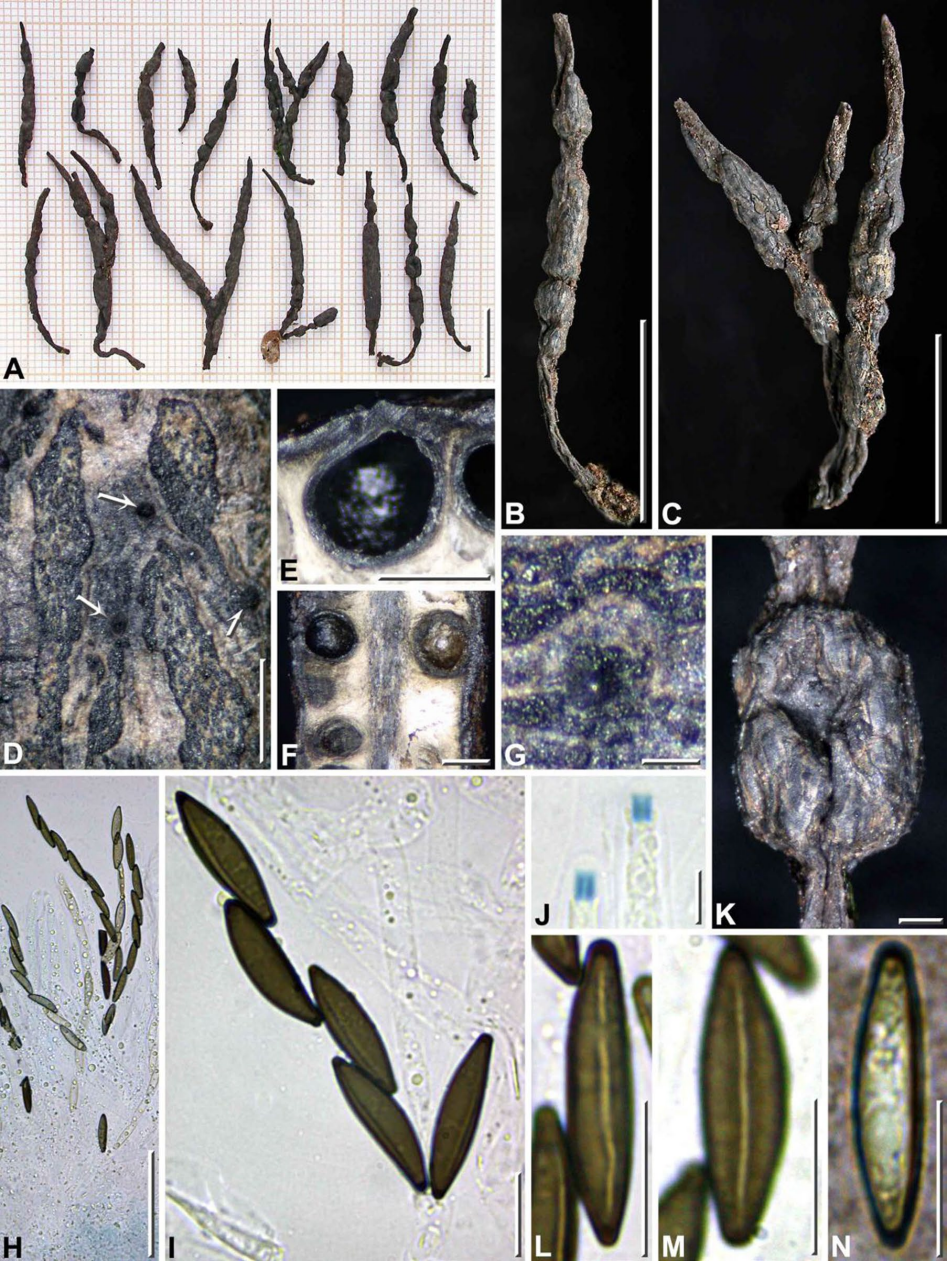
*Xylaria atractoidosperma* (*GYJF19117* holotype). **A** Habit of variously shaped stromata, some moniliform in outline. **B** Simple stroma showing deep constrictions. **C** Branched stroma. **D** Stromatal surface showing strongly granular superficial stripes and small ostioles (arrows). **E** Perithecium in vertical section, immersed beneath a thin carbonaceous crust. **F** Stroma in longitudinal section showing a whitish interior with a black core. **G** Ostiole in close-up. **H** Asci, in chlorazol black. **I** Ascospores in 1% SDS, some showing a germ slit. **J** Ascal apical apparati in Melzer’s reagent. **K** Small portion of a stroma isolated by two deep constrictions. **L, M** Ascospores in 1% SDS showing a straight, almost spore-length germ slit, ventral in L, dorsal in **M**. **N** Immature ascospore in India ink showing a lack of mucilaginous sheath or appendage. Bars in **A–C** = 10 mm; **D–F, K** = 0.5 mm; **G** = 100 µm; **H** = 50 µm; **I, L–N** = 10 µm; **J** = 5 µm

***Xylaria atractoidosperma*** J. Fourn., Y.-M. Ju, H.-M. Hsieh & Lechat, sp. nov. Figure 4, Table 4

**Table 4:** Ascospore dimensions in three collections of *X. atractoidosperma* showing the range of intraspecific variations

Collection numbers	Ascospore measurements with extreme values in parentheses	Q = quotient l/w, *N* = number of measurements	Me = mean values of l/w, Qe = mean quotient l/w
*CLL10017*	(15.7–)16.7–19.9(−20.8) × (3.9–)4.2–5.2(−5.5) µm	Q = (3.3–)3.6–4.4(−4.8), *N* = 60	Me = 18.1 × 4.6 µm, Qe = 3.9
*GYJF19117*	(14.9–)16.0–18.7(−19.8) × (3.4–)3.6–4.4(−4.8) µm	Q = (3.6–)3.9–4.9(−5.4), *N* = 60	Me = 17.5 × 4.1 µm, Qe = 4.3
*GYJF19131*	(17–)17.7–20.3(−21.4) × (3.5–)4.1–4.8(−5.3) µm	Q = (3.5–)3.8–4.7(−5.3), *N* = 60	Me = 18.8 × 4.5 µm, Qe = 4.2
**Cumulated values**	**(14.9–)16–20.3(−21.4) × (3.4–)3.6–5.2(−5.5) µm**	**Q = (3.3–)3.6–4.9(−5.4), N = 180**	**Me = 18.1 × 4.4 µm, Qe = 4.1**

MycoBank MB863148

**Typification**: **FRENCH GUIANA.** Maripasoula, Saül, shortcut toward the airfield, 3.622159 N, 53.204166 W, disturbed secondary rainforest, on dead trunk, 20 Jun 2019, *Fournier, J. GYJF19117* (holotype HAST 147215).

**Etymology**: From Greek $\mathop \alpha \limits^{\mathrm{'}\prime}$*τρακτος* = spindle, with suffix -*ε*$\tilde{\mathop \iota \limits^{\mathrm{'}}}$*δος* (*-oides*) = resembling and *sperma* = seed, spore, for the fusoid ascospores.

**Diagnosis**: Differs from the known species of *Xylaria* with needle-shaped stromata by the combination of deep circumferential constrictions, a gray to dark gray splitting outer layer densely incrusted with black granules, inconspicuous ostioles and dark brown narrowly fusiform-inequilateral ascospores 18.1 × 4.4 µm on average.

**Stromata** 22–40 mm in total height, gregarious, upright, simple to rarely branched or connate at base by two, the fertile parts 9–30 mm high × 1.5–2.2 mm wide, narrowly cylindrical with deep circumferential constrictions giving in places a moniliform outline, straight to slightly curved, with apices sterile and mucronate; surface with unexposed perithecial contours, roughened by a thick, gray to blackish gray outer layer splitting into narrow to wide longitudinal stripes densely incrusted with small black granules, exposing a gray to dark gray superficial layer lacking black granules; underlying crust 70–80 µm thick, black, slightly carbonaceous; interior solid, white to olivaceous gray, fibrous, with a conspicuous black core; the stipes ill-defined, 10–18 mm long × 1.3–1.5 mm wide, black, irregularly flattened, glabrous, smooth with sparse black granules at surface, the base slightly enlarged. **Perithecia** immersed, subglobose, 0.40–0.55 mm diam. **Ostioles** blackish, faintly papillate, ca. 80 µm diam, mostly inconspicuous.

**Paraphyses** hyphal, thin-walled, 4–7 µm wide at base, tapering to 1–1.5 µm wide above asci, sparsely and minutely guttulate, embedded in mucilaginous material. **Asci** cylindrical, with (6–)8 slightly overlapping uniseriately arranged ascospores, the spore-bearing parts 100–115 × 5.5–6.5 (−7.0) µm, the stipes 80–120 µm long, fragile, with apical apparatus 2.1–3.1 × 1.6–2.1 µm (Me = 2.7 × 1.8 µm, *N* = 40), tubular with a faint upper rim, bluing in Melzer’s reagent. **Ascospores** (14.9–)16–20.3(−21.4) × (3.4–)3.6–5.2(−5.5) µm, Q = (3.3–)3.6–4.9(−5.4), *N* = 180 (Me = 18.1 × 4.4 µm, Qe = 4.1), narrowly fusiform-inequilateral with narrowly rounded, at times obtusely truncate ends, olivaceous brown to dark brown, unicellular, with a conspicuous, straight, longitudinally oriented germ slit slightly less than spore-length on the ventral side, occasionally on the dorsal side, without sheath or appendages; epispore smooth.

**Asexual morph** on the natural substrate not observed.

**Other specimens examined (paratypes)**: **FRENCH GUIANA.** Sinnamary, Saint-Elie, Saint-Elie botanical trail, 5.292020 N, 53.05262 W, mesophilic rainforest, on dead corticated trunk, 22 Apr 2010, *Courtecuisse, R. CLL10017* (HAST 147213); Maripasoula, Saül, shortcut toward the airfield, 3.622159 N, 53.204166 W, disturbed secondary rainforest, on dead blackened trunk, 20 Jun 2019, *Fournier, J. GYJF19131* (HAST 147214).

**Known distribution**: French Guiana, known from three collections.

**Comments**: *Xylaria atractoidosperma* is diagnosed by narrowly cylindrical stromata with deep circumferential constrictions and narrowly fusiform ascospores averaging 18.1 × 4.4 µm, with a straight germ slit almost spore-length. This combination of characters sets it apart from other species with needle-shaped stromata. The most similar species in terms of stromatal and ascospore morphology is *X. exechostoma*, which can be distinguished by its more nodulose stromata with a silvery gray outer layer, larger and more prominent ostioles 100–170 µm diam, and slightly shorter ascospores averaging 16.0 × 4.5 µm, with a lower length/width ratio (l/w = 3.6 vs 4.1) (this paper).

Our phylogenetic analyses suggest that *X*. *atractoidosperma* is closely related to *X. stupenda*, from which it differs mainly by significantly longer stromata and smaller, differently shaped ascospores.

**Fig. 5:**
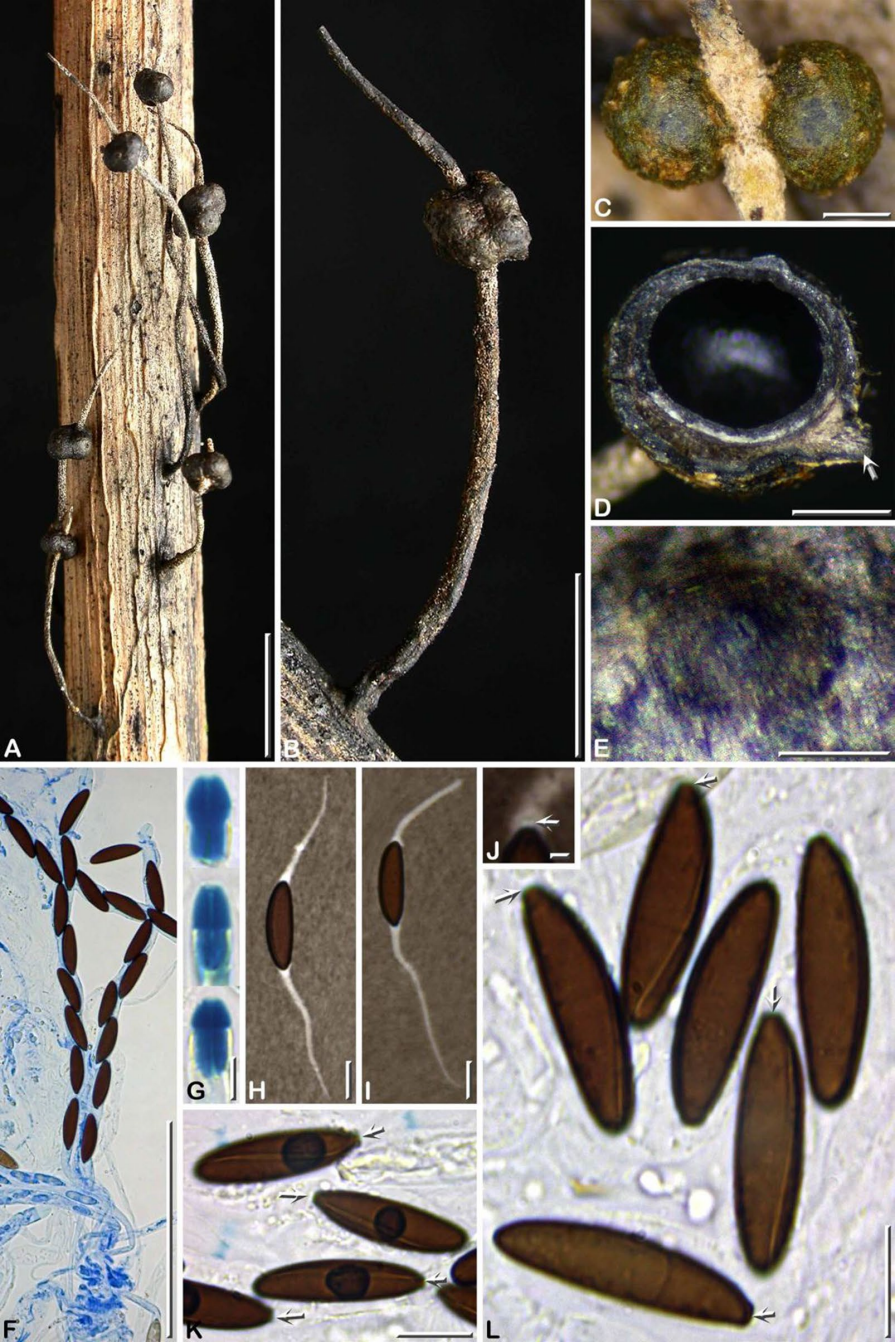
*Xylaria axifera* (*GYJF21028*). **A** Mature stromata arising from a dead petiole. **B** Multiperitheciate mature stroma. **C** Two opposite immature uniperitheciate stromata featuring a waxy olivaceous coating and orange-brown scales. **D** Uniperitheciate stroma in vertical section showing the base of the sterile apical extension (arrow). **E** Ostiole in close-up. **F** Asci in blue Pelikan ink. **G** Ascal apical apparati in Melzer’s reagent. **H, I** Ascospores with filiform secondary appendages, in India ink. **J** Minute refractive cellular appendage (arrow) at the base of a filiform appendage. **K, L** Ascospores respectively in Melzer’s reagent and 1% SDS showing straight to slightly sigmoid germ slits and truncate ends (arrows). Bars in **A, B** = 5 mm; **C, D** = 0.5 mm; **E, F** = 100 µm; **G** = 5 µm; **H, I, K, L** = 10 µm; **J** = 2 µm

***Xylaria axifera*** Mont., Ann. Sci. Nat. Bot., sér. IV, 3: 107. 1855. Figure 5

**Stromata** upright to prostrate, scattered, separate, 15–18 mm in total height, featuring a globose to subglobose fertile head 1.2–2.7 mm diam borne on a filiform stipe 5–13 mm high × 0.3–0.6 mm diam, topped by an acicular extension 12–45 mm high, 0.2–0.3 mm diam at base; fertile head 1–6-peritheciate, with perithecial contours faintly to markedly exposed; surface black, coated with a minutely granular grayish scurf, similar on stipe and acicular extension; subsurface a slightly carbonaceous crust 70–80 µm thick; underlying tissue poorly developed, fibrous, dark to light gray; the stipes minutely hirsute, arising from a black swollen base. Immature stromata coated with a waxy olivaceous yellow substance and orange-brown superficial crust flaking off at maturity. **Perithecia** subglobose, 0.5–0.8 mm diam. **Ostioles** faintly papillate on a blackish discoid base 120–170 µm diam.

**Paraphyses** hyphal, thin-walled, 6–8 µm wide at base, tapering to 1.5–2.0 µm wide above asci, sparsely and minutely guttulate, embedded in mucilaginous material. **Asci** cylindrical, with (6–)8 slightly overlapping uniseriately arranged ascospores, the spore-bearing parts 160–180 × 7.0–8.5(−11.5) µm, the stipes 100–120 µm long, with apical apparatus 8.0–9.4 × 4.5–4.9 µm (Me = 8.7 × 4.7 µm, *N* = 25), coffin- to acorn-shaped with a sharp or obtuse lateral rim, bluing in Melzer’s reagent. **Ascospores** (19.2–)20.7–23.4(−24.2) × (5.2–)5.3–6.0(−6.2) µm, Q = (3.4–)3.6–4.3(−4.6), *N* = 50 (Me = 22.0 × 5.6 µm, Qe = 3.9), narrowly ellipsoid to fusiform, slightly inequilateral with one end narrowly rounded, the other one slightly pinched and truncate bearing a minute refractive cellular appendage, brown, with a conspicuous, straight to slightly sigmoid, germ slit slightly less than spore-length on the dorsal side, with bipolar filiform, acuminate, fragile secondary appendages 30–50 µm long; epispore smooth.

**Asexual morph** on the natural substrate not observed, described from immature stromata by Læssøe and Lodge (1994) who also obtained a sterile culture on OA.

**Known distribution**: Neotropical (Læssøe and Lodge 1994)

**Specimen examined: FRENCH GUIANA.** Roura, Cacao, Camp Bonaventure, 4.323472 N, 52.338502 W, ca. 70 m, hygrophilic secondary rainforest, on dead petiole of *Cecropia* sp., 21 Mar 2021, *Fournier, J. GYJF21028* (HAST 147216).

**Comments**: The Guianese collection of *X. axifera* conforms well to the type material [**GUYANA.** on petioles, *Leprieur, F. R. 1192* (holotype PC 0086061 ex Montagne herb. 10095; isotypes K(M) 130076 ex Berkeley herb., PC ex Leprieur herb., K(M) ex Currey herb.)] and to the descriptions provided by Dennis (1956), Læssøe and Lodge (1994), and Ju and Hsieh (2023). It is distinctive for its occurrence on petioles of dead leaves, its stipitate subglobose fertile heads topped by an acicular extension and its ascospores averaging 22.0 × 5.6 µm, with a dorsal, straight to slightly sigmoid germ slit and long, filiform secondary appendages. It is frequently collected in the immature anamorphic state, in which the stromata bear a characteristic greenish-yellow waxy coating.

In our phylogenetic analyses, *X*. *axifera* forms a clade with *X*. *comosa*, *X*. *comosoides*, *X*. *petiolorum*, and *X*. *squamulosa*. These species are characterized by stromata with a long stipe, a relatively short fertile part compared with the stipe. They also share an outer coating layer that is cracked reticulately, although this feature is less conspicuous in *X*. *axifera*.

***Xylaria berteri*** (Mont.) Cooke ex J. D. Rogers & Y.-M. Ju, N. Amer. Fung. 7(9): 18. 2012.

≡ *Sphaeria berteri* Mont., Ann. Sci. Nat. Bot., sér. II, 3: 353. 1835.

≡ *Hypoxylon berteri* (Mont.) Mont., in C. Gay, Fl. Chilena VII, p. 438. 1850.

≡ *Xylaria berteri* (Mont.) Cooke, Grevillea 11: 126. 1883, nom. inval. ICN Art. 35.2 (Melbourne).

The cited collection from French Guiana closely matches those reported and illustrated from the French West Indies (Fournier et al. 2018c).

**Specimen examined: FRENCH GUIANA.** Sinnamary, Paracou, CIRAD field centre, Guyaflux plot, 5.249218 N, 52.915431 W, lowland rainforest, on dead corticated branch, 25 Jun. 2012, *Fournier, J. GYJF 12207* (JF).

**Fig. 6:**
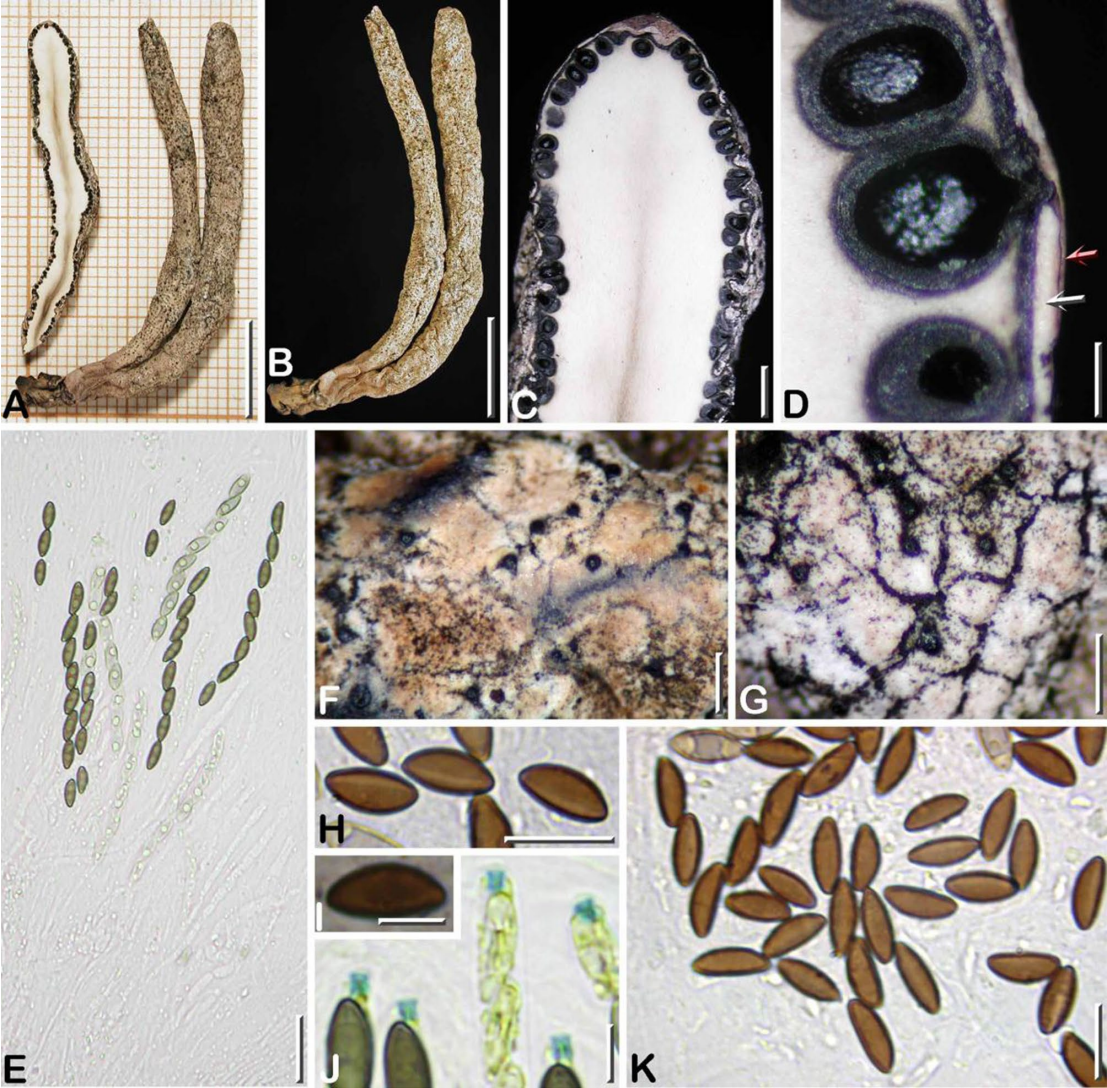
*Xylaria bubalina* (*CLL9076* holotype). **A, B** Stromata in the dry state. **C** Fertile stromatal apex in longitudinal section. **D** Stroma in longitudinal section showing perithecia beneath a thin black leathery crust, a white waxy-granular layer (white arrow) and a thin superficial beige colored outermost layer (red arrow). **E** Asci in the fresh state, in 1% SDS. **F** Beige stromatal surface next to the stipe. **G** White cracked stromatal surface in upper part showing minute ostioles. **H** Ascospores in more or less ventral view showing a germ slit. **I** Ascospore in India ink showing a lack of sheath or appendages. **J** Ascal apical apparati in Melzer’s reagent. **K** Ascospores in 1% SDS, some in ventral view showing a germ slit. Bars in **A, B** = 10 mm; **C** = 1 mm; **D, F, G** = 0.2 mm; **E** = 20 µm; **H, K** = 10 µm; **I, J** = 5 µm

***Xylaria bubalina*** J. Fourn., Y.-M. Ju, H.-M. Hsieh & Lechat, sp. nov. Figure 6

MycoBank MB863149

**Typification**: **FRENCH GUIANA.** Macouria, vicinity of Cayenne, Risquetout Forest, trail to Saut-Léodate, 4.899372 N, 52.555061 W, ca. 55 m, lowland rainforest, on dead wood, 25 Apr 2009, *Lechat, C. CLL9076* (holotype HAST 147218).

**Etymology**: From Latin *bubalinus* = yellowish-beige color of buff leather, for the stromatal surface color.

**Diagnosis**: Differs from known *Xylaria* species with a leathery texture and narrowly clavate stromata by the combination of a beige to whitish surface, minute rounded ostioles, and narrowly inequilateral, medium-brown ascospores averaging 8.9 × 3.6 µm, with narrowly rounded to subacute ends.

**Stromata** upright, simple, connate and curved at base, 38–40 mm in total height, the fertile parts ca. 30 mm high × 4–5 mm broad, narrowly clavate with obtusely rounded fertile apices, surface buff (45) to ochreous (44), finely wrinkled in the dry state, lacking exposed perithecial contours, with a thin, fleeting, rosy buff (61) outer layer over a pure white waxy-powdery layer cracking into small angular scales; underlying black crust leathery, 30–40 µm thick; interior white with a narrow brownish core, solid, spongy; the stipes ill-defined, glabrous, concolorous, blackish at the very base. **Perithecia** subglobose to obovoid, immersed, 0.4–0.6 mm diam. **Ostioles** bluntly rounded-papillate, black, 40–50 µm diam.

**Paraphyses** hyphal, thin-walled, sparse, 4–5 µm wide at base, tapering to 1.2–1.5 µm wide above asci, embedded in mucilaginous material. **Asci** cylindrical, with (6–)8 slightly overlapping uniseriately arranged ascospores, the spore-bearing parts 63–72 × 4.5–5.0 µm, the stipes 65–80 µm long, with apical apparatus 1.7–1.9 × 1.3–1.5 µm (Me = 1.8 × 1.4 µm *N* = 25), short-cylindrical to slightly tubular with a faint upper rim, bluing in Melzer’s reagent. **Ascospores** (7.8–)8.4–9.6(−10.1) × (3.2–)3.4–3.9(−4.1) µm, Q = (2.1–)2.2–2.7(−2.9), *N* = 60, (Me = 8.9 × 3.6 µm, Qe = 2.5), narrowly ellipsoid-inequilateral with narrowly rounded to subacute ends, light olivaceous brown in the fresh state, medium brown in the dry state, unicellular, with a conspicuous, straight, longitudinally oriented germ slit almost spore-length on the ventral side, without sheath or appendages; epispore smooth.

**Asexual morph** on the natural substrate not observed.

**Known distribution**: French Guiana, known only from the type collection.

**Comments**: *Xylaria bubalina* is unique in the combination of narrowly clavate, leathery-textured stromata with a beige to whitish surface, minute rounded papillate ostioles, and narrowly ellipsoid-inequilateral medium-brown ascospores 8.4–9.6 × 3.4–3.9 µm, with narrowly rounded ends and a conspicuous, straight germ slit extending almost the spore length.

The bipartite structure and coloration of the superficial layer is reminiscent of that of *X. enterogena*, a species appearing largely distant in our phylogram. Phylogenetic analyses show close affinities with *X. saulensis*, from which it can be readily distinguished by morphological comparison (this paper).

The type collection consists of only three stromata, fortunately in excellent condition.

**Fig. 7:**
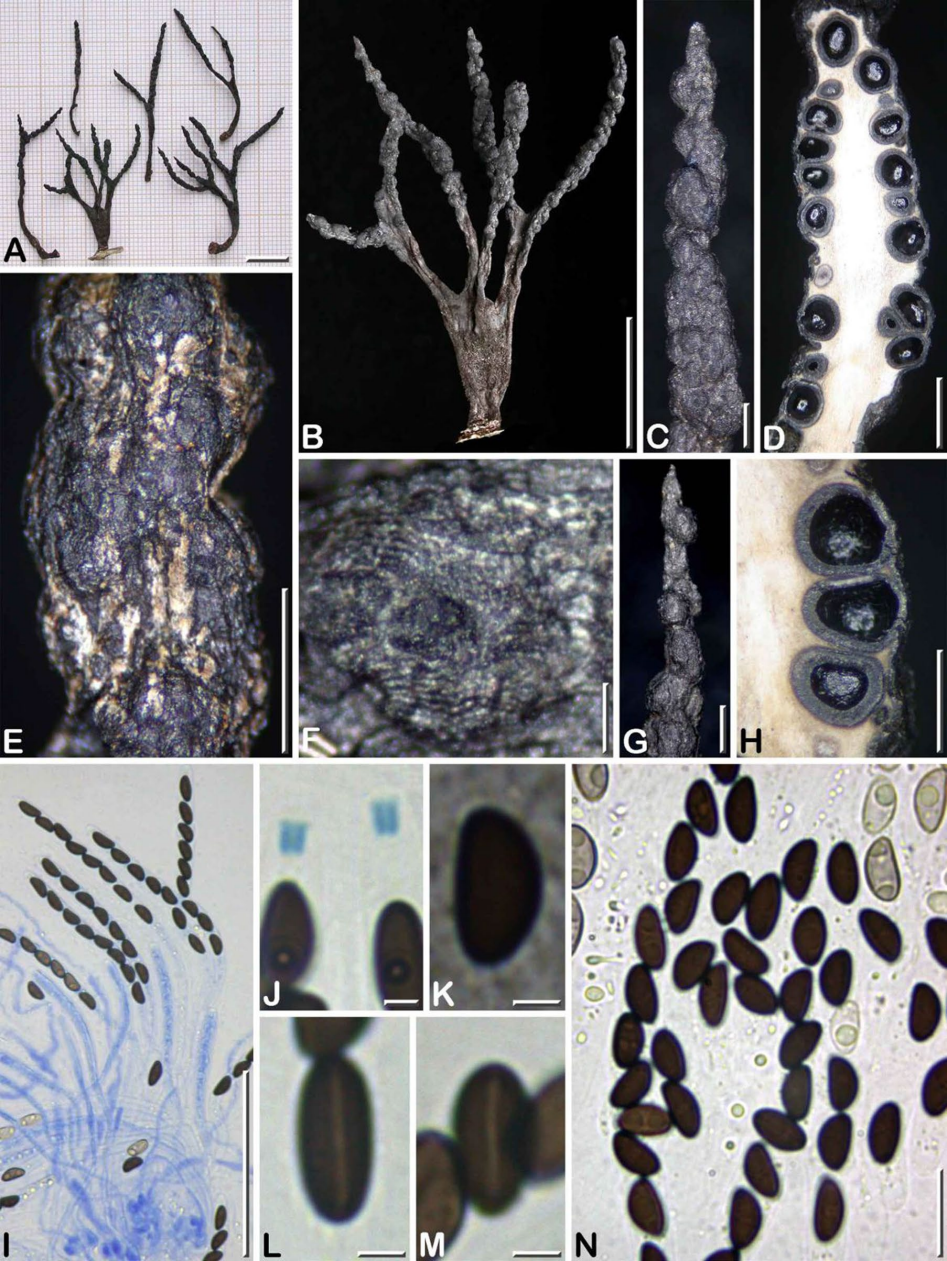
*Xylaria candelabroides* (*GYJF21425* holotype). **A** Variously branched stromata. **B** Two branched stromata connate at base. **C, G** Ascal apices with pointed tips and exposed perithecial contours. **D, H** Stromata in vertical section showing perithecia beneath a thin leathery crust. **E** Stromatal surface showing remnants of a whitish peeling outer layer. **F** Ostiole and finely striate surface in close-up. **I** Asci in blue Pelikan ink. **J** Ascal apical apparati in Melzer’s reagent. **K** Ascospore in India ink showing absence of sheath or appendages. **L, M** Ascospores in ventral view showing a germ slit, in Melzer’s reagent. **N** Ascospores in 1% SDS. Bars in **A, B** = 10 mm; **C–E**, **G** = 1 mm; **F** = 100 µm; **H** = 0.5 mm; **I** = 50 µm; **J–M** = 2 µm; **N** = 10 µm

***Xylaria candelabroides*** J. Fourn., Y.-M. Ju, H.-M. Hsieh & Lechat, sp. nov. Figure 7

MycoBank MB863150

**Typification**: **FRENCH GUIANA.** Maripasoula, Saül, trail to Monts-La-Fumée, vicinity of a tributary of Crique Queue-Hocco, 3.637173 N, 53.204727 W, disturbed mesophilic rainforest, ca. 260 m, on dead decorticated wood, 4 Apr 2021, *Fournier, J. GYJF21425* (holotype HAST 147220).

**Etymology**: From Latin *candelabrum =* branched candlestick, with Greek suffix -*ε*$\tilde{\mathop \iota \limits^{\mathrm{'}}}$*δος* (*-oides*) = resembling, for the branched stromata.

**Diagnosis**: Differs from known species of *Xylaria* with similar leathery, nodulose, acuminate and branched stromata by its smaller, ellipsoid-inequilateral, blackish-brown ascospores, averaging 6.5 × 3.5 µm, with broadly rounded ends.

**Stromata** upright, scattered, simple to dichotomously branched, the stipes often fused giving a strongly ramified outline, 25–36 mm in total height, the fertile heads 6–18 mm high, 0.9–1.8 mm diam, cylindrical to flattened, straight to slightly curved, apically mucronate and sterile; surface nodulose with perithecial contours weakly to strongly exposed and deep circumferential constrictions, black, with a white, superficially brownish gray fibrous outer layer splitting into narrow stripes, fading at maturity and gradually replaced by minute black superficial granules roughening the surface, occasionally arranged in linear rows; the stipes ill-defined, 9–18 mm high, straight to slightly curved, flattened, puckered, black, partly coated with short dark brown hairs, slightly swollen at base; subsurface a black leathery crust 30–40 µm thick; interior solid, dense, white to grayish. **Perithecia** subglobose 0.40–0.55 mm diam. **Ostioles** papillate, obtusely rounded, black, 80–100 µm diam at base, most often inconspicuous.

**Paraphyses** sparse, hyphal, remotely septate, thin-walled, 3–4 µm wide at base, tapering to 0.8–1.5 µm wide above asci, embedded in mucilaginous material. **Asci** cylindrical, with (4–)8 slightly overlapping uniseriately arranged ascospores, the spore-bearing parts 46–52 × 4.0–5.0 µm, the stipes 65–80 µm long, with apical apparatus shortly tubular, apically flattened with a faint lateral rim, 1.9–2.2 × 1.3–1.5 µm (Me = 2.0 × 1.4 µm, *N* = 20), bluing in Melzer’s reagent. **Ascospores** (5.3–)5.9–7.1(−7.7) × (2.8–)3.3–3.7(−3.9) µm, Q = (1.4–)1.7–2.1(−2.4), *N* = 60 (Me = 6.5 × 3.5 µm, Qe = 1.8), ellipsoid-inequilateral with mostly broadly rounded ends, ventrally flat to slightly convex, occasionally slightly concave, dark brown to blackish-brown, unicellular, with a conspicuous, straight, longitudinally oriented germ slit almost spore-length on the ventral side; lacking appendage or mucilaginous sheath; epispore smooth.

**Asexual morph** on the natural substrate not observed.

**Known distribution:** French Guiana, known only from the holotype collection.

**Comments**: The stromata of *X. candelabroides* are characterized by a strongly nodulose surface with a gradually worn-off whitish outer layer, mucronate apices, and a markedly ramified outline. It differs from resembling species by its small, blackish-brown ascospores, averaging 6.5 × 3.5 µm, with broadly rounded ends and a conspicuous germ slit almost spore-length.

**Fig. 8:**
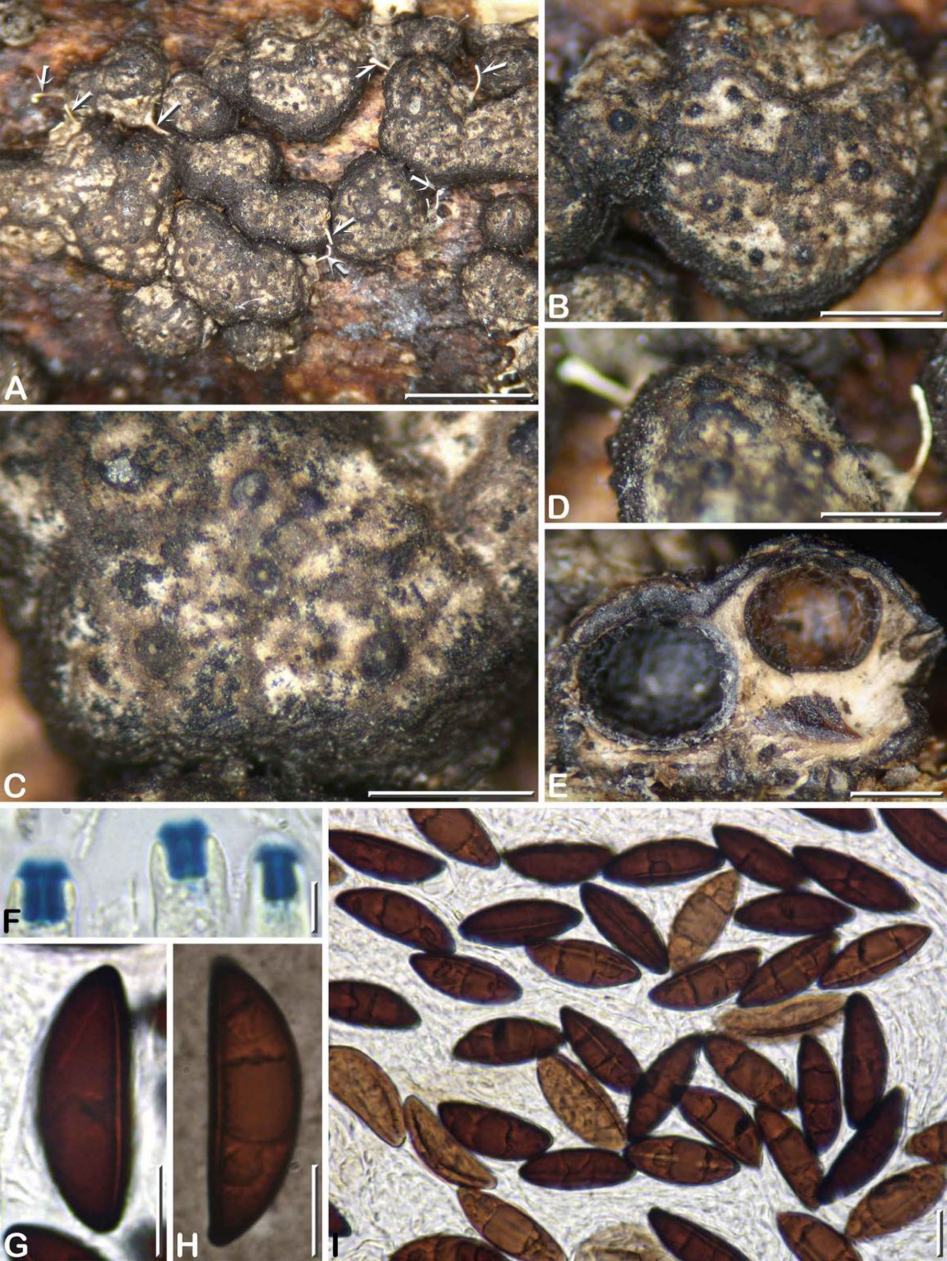
*Xylaria cantareirensis* (*Rogerson 78.259*). **A** Aggregated and coalescent stromata in top view showing white lateral synnemata (arrows). **B, C** Flattened apices of stromata in close-up showing white superficial patches on a brownish gray lower layer, ostioles and scattered clusters of black superficial granulations. **D** Lateral sterile synnemata in close-up. **E** Stroma in vertical section showing a broadly attached base. **F** Ascal apical apparati in Melzer’s reagent. **G** Ascospore in side view showing a ventral germ slit, in Melzer’s reagent. **H** Ascospore in side view with slightly pinched ends, lacking sheath or appendages, in India ink. **I** Immature and mature ascospores, some showing a long ventral germ slit, in 1% SDS. Bars in **A** = 2 mm; **B** = 1 mm; **C–E** = 0.5 mm; **F** = 5 µm; **G–I** = 10 µm

***Xylaria cantareirensis*** (Henn.) J. Fourn. & Lechat, in Fournier, Lechat & Courtecuisse, Ascomycete.org 10 (4): 140. 2018. Figure 8, Table 5

**Table 5:** Ascospore and ascal apical apparatus dimensions from the specimen *Rogerson 78.259* compared with three collections of *X. cantareirensis* from Brazil including the lectotype

Collection numbers	Ascospore measurements with extreme values in parentheses	Mean values, Q = quotient l/w, *N* = number of measurements	Ascal apical apparatus dimensions
*X. cantareirensis Rogerson 78.259*	(25.1–)26.5–29.7(−30.6) × (8.9–)9.4–10.9(−11.7) µm	Me = 28.1 × 10.1 µm, Qe = 2.8, *N* = 60	5.8–6.7 × 3.7–4.5 µm, Me = 6.2 × 4.1 µm
*Hypoxylon cantareirensis* lectotype	(24.9–)26.6–30.0(−32.0) × (9.1–)9.9–11.9(−13.3) µm	28.3 ± 1.7 × 10.9 ± 1.0 µm, Qe = 2.6, *N* = 40	
*Penzigia sessilis* lectotype	(23.4–)24.8–28.4(−31.8) × (7.0–)7.7–9.1(−10.3) µm	26.6 ± 1.8 × 8.4 ± 0.7 µm, *N* = 40	
*Hypoxylon leucodermium* holotype	(24.6–)25.4–28.4(−31.3) × (8.5–)9.7–11.7(−12.8) µm	26.9 ± 1.5 × 10.7 ± 1.0 µm, *N* = 40	

≡ *Hypoxylon cantareirense* Henn., Hedwigia 43: 207. 1904.

≡ *Penzigia cantareirensis* (Henn.) J. H. Miller, Monogr. of World Species of *Hypoxylon*, p. 134. 1961.

= *Penzigia sessilis* Theiss., Ann. Mycol. 6: 347. 1908.

= *Hypoxylon leucodermium* Shear, in Seaver & Chardon, Sci. Surv. Puerto Rico & Virgin Isl. VIII, 1, Bot., p. 66. 1926.

**Stromata** scattered to gregarious, often in contact and coalescent, subglobose to pulvinate, flat-topped to slightly convex, 1–15-peritheciate, 0.5–1.2 mm high × 0.8–1.8 mm diam, sessile, on a broad central connective. Stromatal surface coated by a white, hyphal superficial layer fragmenting into polygonal scales, from which emerge lateral clavate, sterile, white, synnematous outgrowths 0.40–0.60 mm long; the white layer is gradually worn off, revealing a brownish gray tissue over a dull black carbonaceous subsurface roughened by shallow cracks and encrusted by isolated or small clusters of black, carbonaceous, superficial granules gradually showing through the white coating; perithecial contours unexposed; outer crust weakly carbonaceous, 60–100 µm thick, black; interior white to grayish, solid to fibrous. **Perithecia** immersed, subglobose, 0.60–0.90 mm diam. **Ostioles** discoid, not to barely prominent, black, 80–125 µm diam.

**Paraphyses** copious, hyphal, thin-walled, filiform, sparsely guttulate, 4.0–4.5 µm wide at base, tapering to 1.5–2.0 µm wide above asci, embedded in mucilaginous material. **Asci** fragmentary, not measured, short-stipitate, with apical apparatus 5.8–6.7 × 3.7–4.5 µm (Me = 6.2 × 4.1 µm), cylindrical frequently slightly flaring toward the base and a thick, obtusely to sharply prominent upper rim, strongly bluing in Melzer’s reagent. **Ascospores** (25.1–)26.5–29.7(−30.6) × (8.9–)9.4–10.9(−11.7) µm, Q = (2.4–)2.6–3.0(−3.2), *N* = 60 (Me = 28.1 × 10.1 µm, Qe = 2.8), ellipsoid strongly inequilateral, ventrally flat to faintly convex, with narrowly rounded to subacute ends, occasionally beaked, unicellular, dark brown to blackish brown, with a longitudinal, straight germ slit almost spore-length on the ventral side; lacking secondary appendages or mucilaginous sheath; epispore smooth.

**Asexual morph** on the natural substrate not observed.

**Specimen examined: BRAZIL.** Roraima, estrada Boa Vista-Venezuela, 9 km after Boca de Mata, on corticated branchlet, 2 Dec 1977, *Rogerson, C. T. 78.259*, as *Hypoxylon oodes* (NY).

**Known distribution**: Brazil, Puerto Rico; São Paulo, Serra da Cantareira, on dead tree trunk, 1902, *Puttemans, A.* (lectotype of *Hypoxylon cantareirense* S); Rio Grande do Sul, São Leopoldo, on wood, 1908, *Theissen, F.* (lectotype of *Penzigia sessilis* BPI 586691). **PUERTO RICO.** on decaying corticated branches, 1923, *Seaver, F. J. & Chardon, C. E. 632* (holotype of *Hypoxylon leucodermium* BPI 586670).

**Comments**: *Rogerson78.259* from Brazil matches well the type specimens of *Hypoxylon cantareirense* Henn., *Penzigia sessilis* Theiss., and *Hypoxylon leucodermium* Shear, sharing a similar overall morphology and ascospores within exactly the same size range (Table 5). We thus agree with Miller (1961) that *P. sessilis* and *H. leucodermium* are synonyms of *X. cantareirensis*. Collections from French Guiana and Martinique have slightly smaller ascospore dimensions (Table 44), but their status remains unsettled until all members of this complex group are phylogenetically re-evaluated. They are provisionally gathered under the entry *Xylaria* sp. cf. *X*. *cantareirensis*.

See also the entry *Xylaria* sp. cf. *X*. *cantareirensis*.

**Fig. 9:**
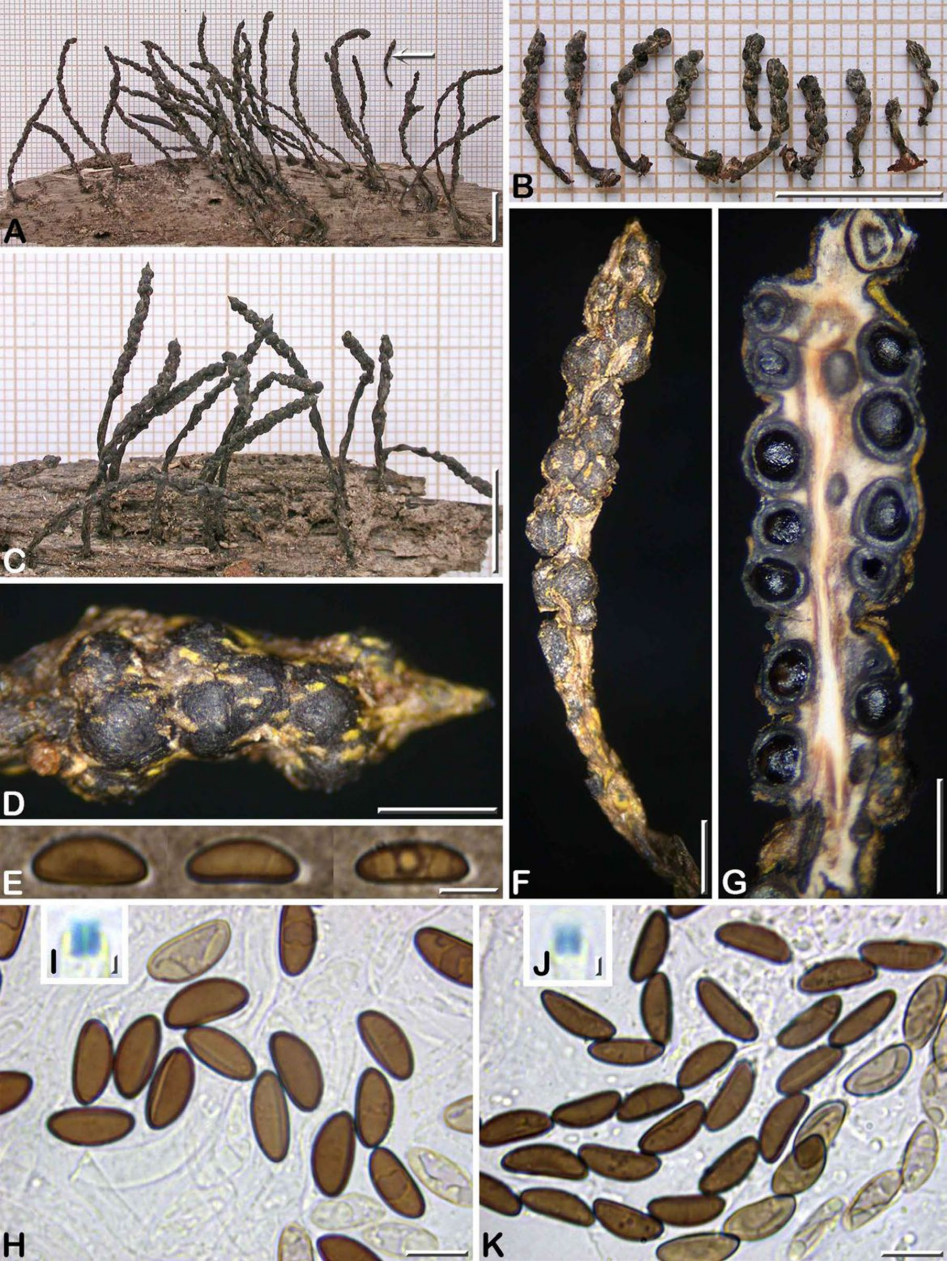
*Xylaria coccophora* (**A*** GYJF21316*; **B, F, H, I ***CLL7056*; **C–E, G, J, K*** GYJF21236*). **A** Habit of tall luxuriant stromata on host surface, in comparison with a minute stroma from *CLL7056* (arrow). **B** Stromata less than 10 mm high. **C** Habit of upright stromata. **D** Apex of a stroma showing exposed perithecial contours, minute ostioles, pointed sterile apex and remnants of a gray and yellow peeling outer layer. **E** Ascospores in side view showing uni- to bipolar secondary appendages, in India ink. **F** Small stroma in side view. **G** Stroma in vertical section showing interior with orange-brown strands and yellow outermost layer. **H, K** Ascospores in 1% SDS, some showing a germ slit. **I, J** Ascal apical apparati, in Melzer’s reagent. Bars in **A–C** = 10 mm; **D, F, G** = 1 mm; **E, H, K** = 5 µm; **I, J** = 1 µm

***Xylaria coccophora*** Mont., Ann. Sci. Nat. Bot., sér. IV, 3: 109. 1855. Figure 9, Table 6

**Table 6:** Ascospore dimensions in six collections of *X. coccophora* from French Guiana, showing a fairly wide range of intraspecific variations, compared with those reported for the holotype in literature

Collections numbers	Ascospore measurements with extreme values in parentheses	Q = quotient l/w, *N* = number of measurements	Me = mean values of l/w, Qe = mean quotient l/w
*CLL7056*	(9.4–)10.0–11.1(−12.2) × (3.3–)3.6–4.3(−4.7) µm	Q = (2.3–)2.4–2.9(−3.4), *N* = 60	Me = 10.6 × 4.0 µm, Qe = 2.7
*CLL8096*	(9.4–)9.9–11.4(−13.4) × (3.4–)3.5–4.4(−4.7) µm	Q = (2.2–)2.4–3.1(−3.3), *N* = 60	Me = 10.7 × 3.9 µm, Qe = 2.7
*GYJF19186*	(7.7–)9.3–10.3(−11.0) × (3.1–)3.5–4.1(−4.4) µm	Q = (2.0–)2.4–2.9(−3.1), *N* = 60	Me = 9.7 × 3.8 µm, Qe = 2.6
*GYJF21236*	(8.2–)8.6–10.1(−10.6) × (2.9–)3.3–4.0(−4.2) µm	Q = (2.2–)2.3–3.0(−3.4), *N* = 60	Me = 9.4 × 3.6 µm, Qe = 2.6
*GYJF21316*	(8.7–)8.9–9.9(−10.7) × (3.1–)3.3–3.8(−4.0) µm	Q = (2.3–)2.4–2.9(−3.2), *N* = 60	Me = 9.4 × 3.6 µm, Qe = 2.7
*GYJF21369*	(8.9–)9.4–10.4(−11.3) × (3.4–)3.6–4.3(−4.5) µm	Q = (2.2–)2.3–2.7(−3.1), *N* = 60	Me = 9.9 × 3.9 µm, Qe = 2.5
**Cumulated values**	**(7.7–)8.6–11.4(−13.4) × (2.9–)3.3–4.4(−4.7) µm**	**Q = (2.0–)2.3–3.1(−3.4), N = 360**	**Me = 9.95 × 3.8 µm, Qe = 2.6**
Leprieur 1398 holotype (Joly 1968)	(7–)9.47–9.58(−12) × (3–)4.22–4.3(−6) µm		Me = 9.5 × 4.3 µm, Qe = 2.2

**Stromata** scattered to gregarious, prostrate to upright, separate, rarely connate at base by two, slender, terete to slightly flattened or somewhat constricted in places, straight to curved, simple to occasionally forked, strongly nodulose, with mucronate sterile apices, subsessile to stipitate, 4–42 mm in total height, the fertile head 1.2–35 mm high (including apical sterile extension) × 0.6–1.8 mm diam, the stipe 1.5–16 mm high × 0.5–1 mm diam; surface dull black, consisting of a leathery crust 30–50 µm thick, glabrous, finely granular and roughened, overlain by a long persistent usually bipartite splitting outer layer, superficially gray to tan, with a more or less well-developed yellowish to sulphur-yellow basal layer showing through in places, releasing orange yellow pigments in 10% KOH when the yellow basal layer is present; perithecial contours strongly to fully exposed, with perithecia single or fused by 2–4; the stipes ill- to well-defined, simple, strap-like, black, glabrous, slightly enlarged at base; interior solid, whitish, spongy, with a conspicuous orange-brown inner core, blackish in places with age. **Perithecia** subglobose to depressed-spherical, 0.40–0.50 mm diam. **Ostioles** obtusely papillate, black, ca. 80 µm diam at base, barely prominent and often inconspicuous.

**Paraphyses** and **Asci** as in Fournier et al. (2020). **Ascospores** (7.7–)8.6–11.4(−13.4) × (2.9–)3.3–4.4(−4.7) µm, Q = (2.0–)2.3–3.1(−3.4), *N* = 360 (Me = 10.0 × 3.8 µm, Qe = 2.6), ellipsoid-inequilateral with broadly rounded ends, straight to slightly ventrally concave, medium brown, with a conspicuous longitudinally oriented germ slit almost spore-length on the ventral side; with a thin, inconspicuous mucilaginous sheath swollen at one or both ends to form secondary appendages visible in India ink but not in water; epispore smooth.

**Asexual morph** on the natural substrate not observed. Cultural characteristics on OMA and asexual morph were described by Rogers et al. (1988) based on material from Venezuela.

**Specimens examined: FRENCH GUIANA.** Maripasoula, Saül, trail head to Sentier des Gros Arbres, 3.6201 N, 53.207989 W, ca. 210 m, disturbed secondary rainforest, on dead corticated branchlet, 28 Mar 2021, *Fournier, J. GYJF21227* (HAST 147225); Maripasoula, Saül, trail head to Roche-Bateau, Crique-Cochon, 3.625994 N, 53.1955 W, 210–220 m, on dead rotten wood, 31 Mar 2021, *Fournier, J. GYJF21316* (HAST 147226); Maripasoula, Saül, trail head to Roche-Bateau, 3.620498 N, 53.199309 W, ca. 230 m, disturbed secondary rainforest, on dead corticated branchlet associated with *X. oligotoma*, 22 Jun 2019, *Fournier, J. GYJF19200-2* (HAST 147227); Kourou, Sentier de la Montagne des Singes, 5.068808 N, 52.69478 W, lowland rainforest, on dead corticated branch, 27 Apr 2008, *Courtecuisse, R. CLL8000* (HAST 147228); Maripasoula, Saül, shortcut to the airfield, 3.622852 N, 53.205768 W, 210–220 m, disturbed secondary rainforest, on dead corticated branch, 29 Mar 2021, *Fournier, J. GYJF21236* (HAST 147229); Maripasoula, Saül, trail to Monts-La Fumée, 3.634796 N, 53.205457 W, ca. 270 m, disturbed secondary rainforest, on dead corticated branch, 2 Apr 2021, *Fournier, J. GYJF21369* (HAST 147230); Sinnamary, Paracou, CIRAD research Centre, Guyaflux plot, 5.249218 N, 52.915431 W, lowland rainforest, on dead corticated branchlet 1 cm diam, 26 Feb 2007, *Lechat, C. CLL7056* (HAST 147231); Maripasoula, Saül, trail head to Roche-Bateau, 3.620498 N, 53.199309 W, ca. 230 m, disturbed secondary rainforest, on dead corticated branchlet, 22 Jun 2019, *Fournier, J. GYJF19186* (HAST 147232); Macouria, Risquetout Forest, trail to Saut-Léodate, 4.899372 N, 52.555061 W, ca. 55 m, lowland rainforest, on dead corticated branchlet 10–15 mm diam, 6 May 2008, *Lechat, C. CLL8096* (HAST 147233); around Cayenne, on rotten wood, *Leprieur, F. R. 1398* (holotype PC ex Montagne herb. 10098, isotype K[M] 144055).

**Known distribution**: Neotropical (Fournier et al. 2020).

**Comments**: *Xylaria coccophora*, originally described from French Guiana by Montagne (1855), is characterized by filiform to narrowly cylindrical, soft-textured, lignicolous stromata with strongly exposed perithecial contours, mucronate sterile apices, and ascospores 8.6–11.4 × 3.3–4.4 µm, with obtuse ends and a long germ slit. Its most distinctive diagnostic character is a bipartite, splitting outer layer consisting of a superficial gray to tan layer overlying a yellowish to sulphur-yellow basal layer. This feature is usually diagnostic but may be absent in old, weathered stromata, making distinction from species with similarly strongly exposed perithecial contours, such as *X. gracillima*, occasionally challenging. In such cases, *X. coccophora* can be recognized by its partly orange-brown interior and slightly larger ascospores with polar secondary appendages. Phylogenetically, *X*. *coccophora* and *X*. *gracillima* are not closely allied (Fig. 1).

The French Guiana collections that we examined showed a fairly wide range of variation in ascospore dimensions, which could not be correlated with other stromatal characteristics. The wide variation in stromatal height (Fig. 9) appears, as is often the case in the genus, to be environmentally influenced: stromata are much smaller on twigs or branchlets in aerial situations than on dead wood partly buried in leaf litter.

***Xylaria*** ***collabens*** (Mont.) Fr., Summa Veg. Scand. II, p. 381. 1849.

≡ *Hypoxylon collabens* Mont., Ann. Sci. Nat. Bot., sér. II, 13: 347. 1840.

= *Xylaria tuberoides* Rehm, Hedwigia 40: 146. 1901.

The cited collections from French Guiana closely match those reported and illustrated as *X*. *tuberoides* from the French West Indies (Fournier et al. 2019).

**Specimens examined: BRAZIL.** Amazonas, Rio Juruá, Juruá Miry, on wood, *Ule, E. 794* (isotypes of *X. tuberoides* S F5647, HBG). **FRENCH GUIANA.** Maripasoula, Saül, trail head to Sentier des Gros Arbres, 3.620839 N, 53.208536 W, ca. 210 m, disturbed mesophilic rainforest, on dead blackened wood, 26 Mar 2021, *Fournier, J. GYJF21243* (HAST 147234); *Leprieur, F. R. 407* (lectotype of *X. collabens* PC 0086067 ex Montagne herb., isolectotype K(M) 156826 ex Cooke herb.).

**Comments:** *Xylaria collabens* was synonymized with *X. comosa* by Dennis (1956), but Læssøe (1999), after studying *Leprieur 407*, suspected that it is referable to *X*. *tuberoides*.

***Xylaria columnifera*** Mont., Ann. Sci. Nat. Bot., sér. IV, 3: 102. 1855.

= *Xylaria olobapha* Berk. ex Cooke, Grevillea 11: 84. 1883.

The cited collections from French Guiana closely match those reported and illustrated as *X*. *olobapha* from the French West Indies (Fournier et al. 2019).

**Specimens examined: FRENCH GUIANA.** Roura, Cacao, Camp Bonaventure, 4.323044 N, 52.339371 W, 59 m, on blackened wood, 20 Mar 2021, *Fournier, J. GYJF21018* (HAST 147235); Cayenne, on rotten wood, *Leprieur, F. R. 1225* (lectotype of *X. columnifera* PC ex Montagne herb. MC10100); Cayenne, on rotten wood, *Leprieur, F. R. 1227* (syntype of *X. columnifera* PC ex Montagne herb. MC10099).

**Comments:** Dennis (1956) synonymized *X*. *olobapha* with *X*. *hyperythra* and considered the type of *X. columnifera* to represent a young state of *X. hyperythra*. *Xylaria hyperythra* is undoubtedly closely related to *X*. *columnifera*, but it differs mainly by its smaller ascospores.

**Fig. 10:**
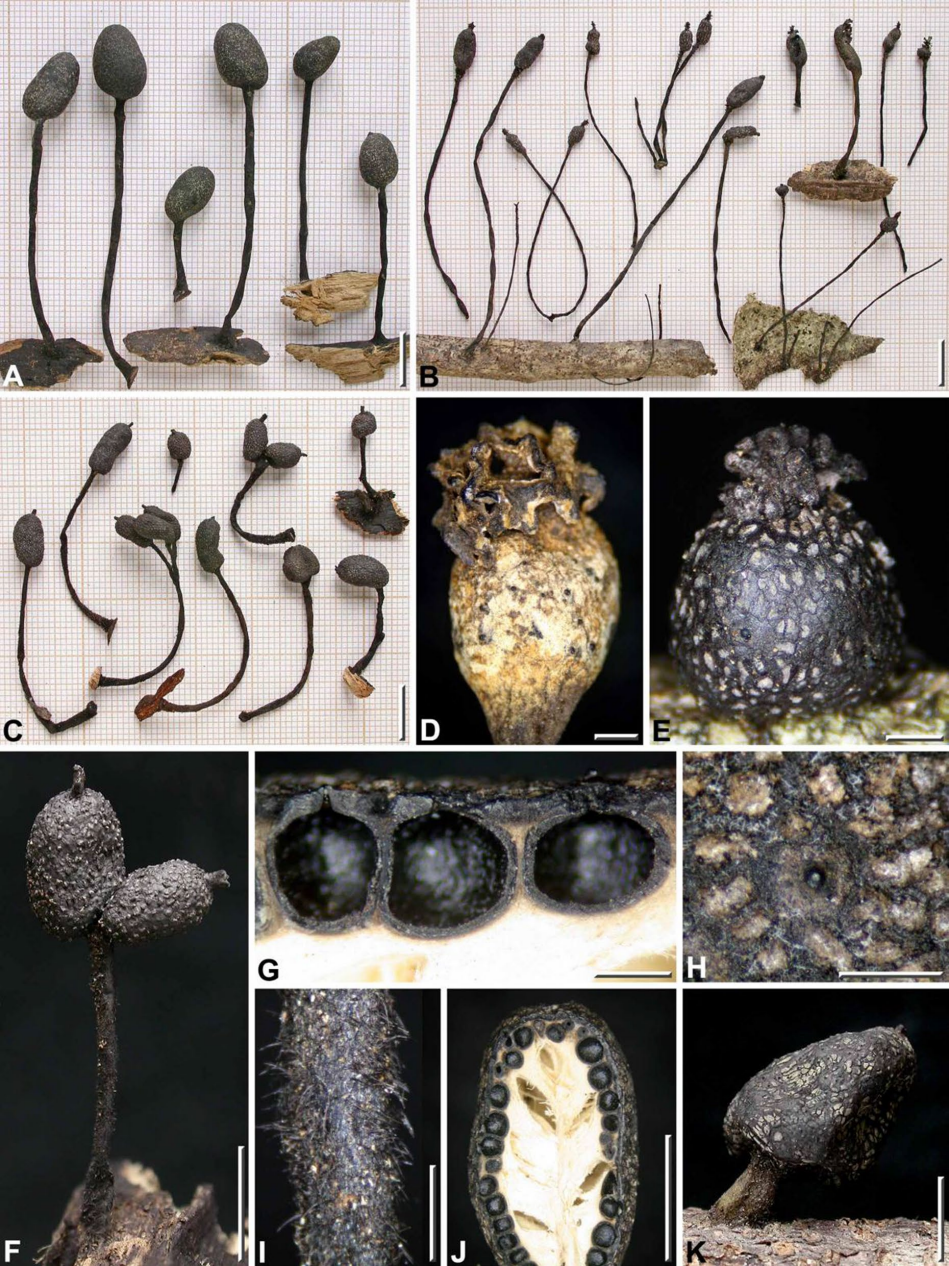
*Xylaria comosa* (**A ***GYJF12050*; **B ***GYJF21129*; **C, F ***GYJF21367*; **D ***CLL7068*; **E** *GYJF12176*; **G, H**, **J ***CLL8101*; **I ***GYJF21373-2*; **K ***GYJF21368*). **A–C, F, K** Variously shaped, apiculate and stipitate stromata. **D** Immature stroma showing a dull yellow surface and shortly branched remnants of conidial apical processes. **E** Sessile stroma. **G, J** Stroma in longitudinal section at different magnifications showing a thick black crust and a lacunose cream-white fibrous interior. **H** Shiny black ostiole and grayish superficial scales. **I** Hairy stipe in close-up. Bars in **A–C** = 10 mm; **D, E, I** = 1 mm; **F, J, K** = 5 mm; **G, H** = 0.5 mm

**Fig. 11:**
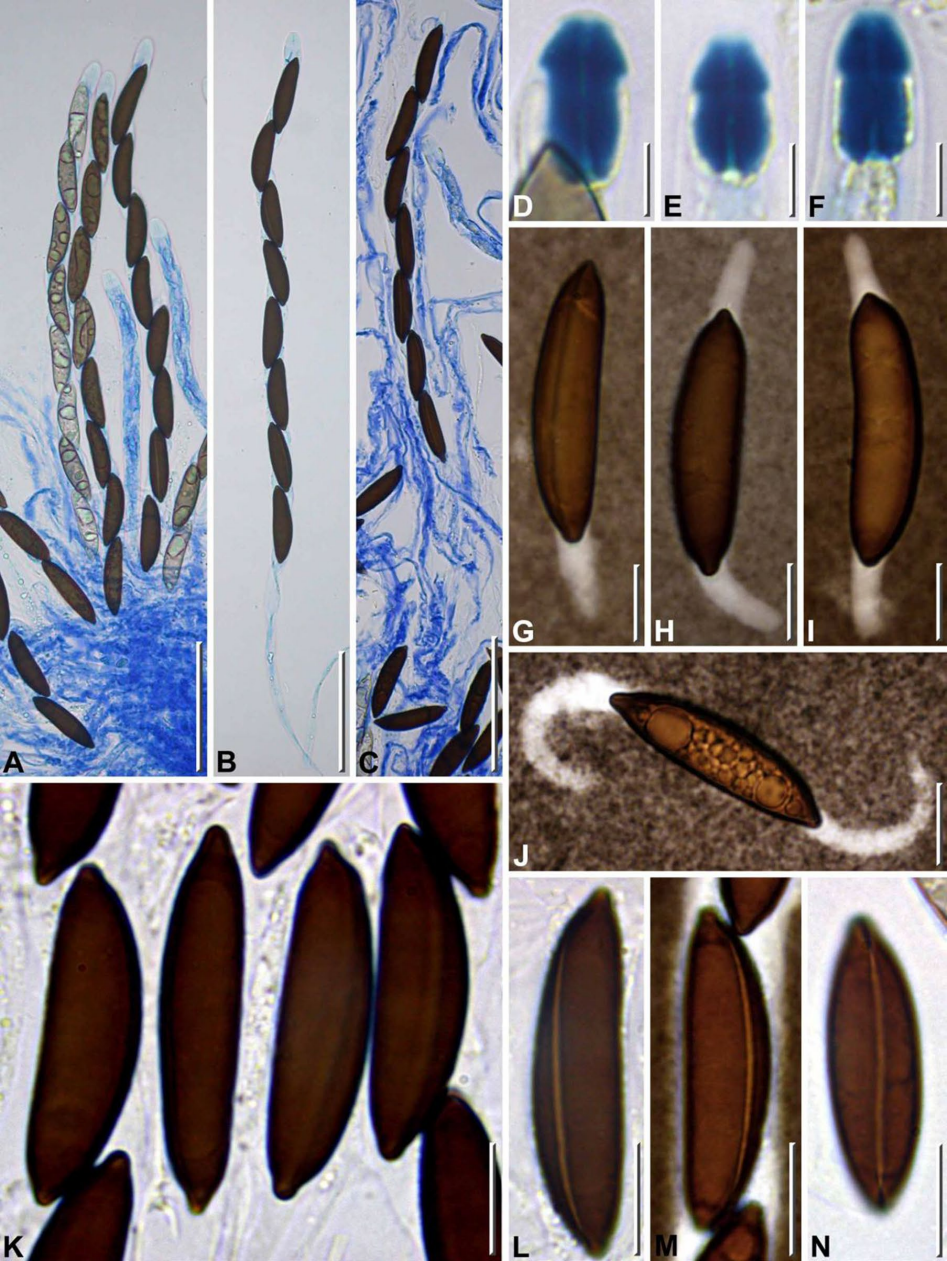
*Xylaria comosa* (**A–D, G–I, K, M ***GYJF21332*; **E ***CLL10024*; **F ***GYJF21373-2*; **J ***CLL8101*; **N ***GYJF12076*). **A** Bunch of immature and mature asci. **B** Isolated mature ascus with a broken stipe. **C** Seven-spored mature ascus and paraphyses (**A–C**, in blue Pelikan ink). **D–F** Ascal apical apparati in Melzer’s reagent. **G–J** Ascospores with uni- to bipolar appendages, in India ink. **K** Ascospores showing pinched ends, in 1% SDS. **L** Ascospore showing a slightly oblique dorsal germ slit. **M** Ascospore in the ascus showing a longitudinal dorsal germ slit, in India ink. **N** Ascospore in dorsal view showing a slightly undulate germ slit (**L** and **N** in 1% SDS). Bars in **A–C** = 50 µm; **D–F** = 5 µm; **G–N** = 10 µm

***Xylaria comosa*** (Mont.) Fr., Summa Veg. Scand. II, p. 381. 1849. Figures 10, 11, Table 7

**Table 7:** Ascospore and ascal apical apparatus dimensions in six collections of *X. comosa* showing a fairly wide variation range, compared with those reported in literature

Collection numbers	Ascospore measurements with extreme values in parentheses	Q = quotient l/w, *N* = number of measurements	Me = mean values of l/w, Qe = mean quotient l/w	Ascal apical apparatus h × w
*CLL8101*	(27.8–)28.7–32.7(−36.3) × (6.6–)6.8–7.7(−8.2) µm	Q = (3.5–)3.8–4.6(−5.1), *N* = 60	Me = 30.6 × 7.3 µm, Qe = 4.2	8.6–9.4 × 4.8–5.3 µm, Me = 8.9 × 5.0
*CLL10024*	(26.0–)28.1–31.9(−33.6) × (7.8–)8.3–9.6(−10.3) µm	Q = (2.8–)3.0–3.7(−4.1), *N* = 60	Me = 29.8 × 9.0 µm, Qe = 3.3	8.7–9.8 × 5.1–6.0 µm, Me = 9.2 × 5.6
*GYJF12050*	(26.8–)27.4–30.6(−32.6) × (6.7–)6.9–7.8(−8.0) µm	Q = (3.5–)3.6–4.2(−4.5), *N* = 60	Me = 29.2 × 7.4 µm, Qe = 4.0	8.2–9.9 × 4.6–5.3 µm, Me = 8.8 × 4.9
*GYJF19060*	(28.4–)29.4–32.6(−33.5) × (6.9–)7.2–7.9(−8.6) µm	Q = (3.6–)3.8–4.5(−4.9), *N* = 60	Me = 31.0 × 7.5 µm, Qe = 4.1	8.0–9.3 × 4.9–5.6 µm, Me = 8.8 × 5.3
*GYJF21368*	(29.1–)30.5–35.2(−36.4) × (6.3–)7.2–8.8(−9.8) µm	Q = (3.4–)3.8–4.6(−5.3), *N* = 60	Me = 32.9 × 7.9 µm, Qe = 4.2	
*GYJF21373-2*	(26.7–)27.1–30.8(−32.2) × (6.8–)7.3–8.3(−8.6) µm	Q = (3.3–)3.4–4.1(−4.5), *N* = 60	Me = 28.8 × 7.7 µm, Qe = 3.8	8.7–11 × 4.8–5.5 µm, Me = 9.8 × 5.2
**Cumulated values**	**(26.0–)27.1–35.2(−36.4) × (6.3–)6.8–9.6(−10.3) µm**	**Q = (2.8–)3.0–4.6(−5.3), N = 360**	**Me = 30.4 × 7.8 µm, Qe = 3.9**	**8.0–11 × 4.6–6.0 µm, Me = 9.1 × 5.2**
San Martín and Rogers (1989)	(25–)29–33(−36) × 7–8(−9) µm			
Læssøe (1999)	(25–)26–35(−38.3) × (5.6–)6.4–8.1(−13) µm	*N* = 165	Me = 27–33.5 × 6.8–7.8 µm	10–14.5(−18.5) × (4–)5–6.5(−7.5) µm

≡ *Hypoxylon comosum* Mont., Ann. Sci. Nat. Bot., sér. II, 13: 345. 1840.

**Typification**: **FRENCH GUIANA.** on dead wood, Jan 1839, *Leprieur, F. R. 417&418* (holotype of *Hypoxylon comosum* PC ex Montagne herb.; isotypes UPS F-176022 ex Fries herb., PC ex Leprieur herb.), immature; Maripasoula, Saül, trail head of Sentier des Gros Arbres, 3.620839 N, 53.208536 W, ca. 210 m, on dead branch, 26 Mar 2021, *Fournier, J. GYJF21129* (epitype HAST 147254).

**Stromata** scattered on host surface, rarely connate at base, 5–70(−130) mm in total height, subsessile to long-stipitate, the fertile parts cylindrical, ellipsoid, subglobose to ovoid 4–40 mm high × 2.2–10 mm wide, mostly simple, occasionally apically branched in small bunches of 2–3, straight to slightly curved, not or rarely splitting upon drying; apices obtusely rounded bearing upright, conspicuously branched conidial processes, more rarely apiculate or spiny, present on the stipe just below the fertile head or totally absent at full maturity; the stipes filiform to short-cylindrical, (0–)3.0–60(−105) mm high, 0.5–1.5(−2.0) mm wide, up to 3.4 mm diam at the very base, well-defined, fairly flexible, black, finely hairy all along to shaggy at base, with a slightly swollen base; hairy coating vanishing with age except at base. Surface gray to dull yellow when immature, becoming dark gray to black, with unexposed perithecial contours, roughened by small, long-persistent yellowish gray, brownish gray or silvery gray polygonal scales and ostioles; stromatal crust just beneath surface black, carbonaceous, 120–170 µm thick; interior white to cream-white, solid, fibrous, with a narrow light brown core, becoming lacunose upon drying but rarely hollow. **Perithecia** densely distributed, subglobose to slightly depressed-spherical, 0.6–0.9 mm diam, fully immersed. **Ostioles** bluntly papillate 70–80 µm diam at base to flattened-discoid 120–170 µm diam, black to shiny black, usually located at the center of a large, polygonal, paler gray area.

**Paraphyses** copious, hyphal, hyaline, remotely septate, simple, 8.0–9.5 µm wide at base, tapering to 1.5–2.0 µm wide, embedded in mucilaginous material. **Asci** cylindrical, with (4–6–)8 uniseriately arranged, slightly overlapping ascospores, the spore-bearing parts (165–)190–215 µm long × 9.0–11.5 µm wide, the stipes 70–90 µm long, fragile, with apical apparatus fairly variable, more or less strongly constricted at upper third, apically truncate and trapezoid with sharp lateral rims, tubular to doliform in lower part, 8.0–11.0 × 4.6–6.0 µm (Me = 9.1 × 5.2 µm, *N* = 100), strongly bluing in Melzer’s reagent. **Ascospores** (26.0–)27.1–35.2(−36.4) × (6.3–)6.8–9.6(−10.3) µm, Q = (2.8–)3.0–4.6(−5.3), *N* = 360 (Me = 30.4 × 7.8 µm, Qe = 3.9), ellipsoid-inequilateral with narrowly rounded and pinched ends, ventrally flat to slightly concave, dark brown, with a straight, occasionally slightly undulate germ slit spore-length or nearly so on the dorsal side, longitudinally oriented to slightly oblique, with fragile, straight to curved bipolar secondary appendages 7.5–13.5(−28) µm long, visible in water, best seen in India ink; primary appendages not seen, or hardly distinguishable from small mucilaginous remnants; epispore smooth.

**Asexual morph** present on apical conidial pegs and branched processes but soon vanishing and not investigated during this study.

**Other specimens examined: FRENCH GUIANA.** Sinnamary, Paracou, CIRAD field centre, Guyaflux plot, 5.249218 N, 52.915431 W, lowland rainforest, on dead wood, 30 Apr 2008, *Lechat, C. CLL8039* (HAST 147236); Sinnamary, Paracou, CIRAD field centre, Guyaflux plot, 5.249218 N, 52.915431 W, lowland rainforest, on dead wood, 27 Feb 2007, *Lechat, C. CLL7068* (HAST 147237), immature; Roura: Cacao, Sentier Molokoï, 4.560171 N, 52.461969 W, disturbed secondary rainforest, on dead wood, 23 Apr 2010, *Lechat, C. CLLG 10024* (HAST 147238); Maripasoula, Saül, trail head to Roche-Bateau, Crique-Cochon, 3.625994 N, 53.1955 W, 210–220 m, disturbed secondary rainforest, on dead branch, 23 Aug 2018, *Roy, M. GYJF18111* (HAST 147239); Sinnamary, Paracou, CIRAD field centre, Guyaflux plot, 5.249218 N, 52.915431 W, lowland rainforest, on bark, 24 Jun 2012, *Fournier, J. GYJF12176* (HAST 147240); Sinnamary, Paracou, CIRAD field centre, Guyaflux plot, 5.249218 N, 52.915431 W, lowland rainforest, on dead wood, 28 Jun 2012, *Fournier, J. GYJF12254* (HAST 147241); Sinnamary, Saint-Elie, Saint-Elie botanical trail, 5.292020 N, 53.05262 W, mesophilic rainforest, on dead corticated branch, 16 Apr 2009, *Lechat, C. CLL9002* (HAST 147242); Sinnamary, Saint-Elie, Saint-Elie botanical trail, 5.292020 N, 53.05262 W, mesophilic rainforest, on dead corticated branch, 16 Apr 2009, *Lechat, C. CLL9003* (HAST 147243), immature; Macouria, Risquetout Forest, trail to Saut-Léodate, 4.899372 N, 52.555061 W, ca. 55 m, lowland rainforest, on dead corticated branch, 6 May 2008, *Lechat, C. CLL8101* (HAST 147244); Maripasoula, Saül, shortcut to the airfield, 3.622159 N, 53.204166 W, disturbed secondary rainforest, 210–220 m, on dead corticated branch, 20 Jun 2019, *Fournier, J. GYJF19136* (HAST 147245); Maripasoula, Saül, trail head of Sentier des Gros Arbres, 3.620839 N, 53.208536 W, ca. 210 m, on dead corticated branch, 3 Apr 2021, *Fournier, J. GYJF21400* (HAST 147246); Maripasoula, Saül, trail to Monts-La Fumée, 3.634796 N, 53.205457 W, ca. 270 m, disturbed mesophilic rainforest, on dead corticated branch, 2 Apr 2021, *Fournier, J. GYJF21368* (HAST 147247); Maripasoula, Saül, shortcut to the airfield, 3.622852 N, 53.205768 W, 210–220 m, disturbed secondary rainforest, on dead wood, 29 Mar 2021, *Fournier, J. GYJF21253* (HAST 147248); Maripasoula, Saül, trail head of Sentier des Gros Arbres, 3.620324 N, 53.207951 W, ca. 210 m, disturbed secondary rainforest, on dead blackened wood, 18 Jun 2019, *Fournier, J. GYJF19060* (HAST 147249); Roura, Cacao, Camp Bonaventure, 4.326815 N, 52.341431 W, 60–65 m, on dead twigs in the litter, 22 Mar 2021, *Fournier, J. GYJF21069* (HAST 147250); Maripasoula, Saül, trail head of Sentier des Gros Arbres, 3.620839 N, 53.208536 W, ca. 210 m, on dead wood, 28 Mar 2021, *Fournier, J. GYJF21205* (HAST 147251); Maripasoula, Saül, trail head of Sentier des Gros Arbres, 3.620839 N, 53.208536 W, ca. 210 m, on dead branch, 26 Mar 2021, *Fournier, J. GYJF21121* (HAST 147252); Maripasoula, Saül, trail head of Sentier des Gros Arbres, 3.620839 N, 53.208536 W, ca. 210 m, on bark, 27 Mar 2021, *Fournier, J. GYJF21162* (HAST 147253); Maripasoula, Saül, trail head of Sentier des Gros Arbres, Crique Grand-Fossé, 3.617198 N, 53.209029 W, on dead blackened branchlet, 1 Apr 2021, *Fournier, J. GYJF21332* (HAST 147255); Maripasoula, Saül, trail to Monts-La Fumée, 3.634796 N, 53.205457 W, ca. 270 m, disturbed mesophilic rainforest, on dead blackened wood, 2 Apr 2021, *Fournier, J. GYJF21367* (HAST 147256); Maripasoula, Saül, trail to Monts-La Fumée, 3.634796 N, 53.205457 W, ca. 270 m, disturbed mesophilic rainforest, on dead wood, associated with *X. comosoides*, 2 Apr 2021, *Thonnel, A. GYJF21373-2* (HAST 147257); Maripasoula, Saül, trail head of Sentier des Gros Arbres, 3.620839 N, 53.208536 W, ca. 210 m, on dead corticated trunk, 27 Mar 2021, *Fournier, J. GYJF21189* (HAST 147258); Régina, Nouragues natural reserve, Inselberg camp, 4.311240 N, 52.132789 W, ca. 300 m, hygrophilic rainforest, on bark, 19 Jun 2012, *Fournier, J. GYJF12094* (HAST 147259); Régina, vicinity of airstrip, 4.316743 N, 52.131763 W, disturbed marshy rainforest, on dead wood, 22 Apr 2009, *Lechat, C. CLL9052* (HAST 147260); Régina, Nouragues natural reserve, Inselberg camp, 4.311240 N, 52.132789 W, ca. 300 m, hygrophilic rainforest, on blackened wood, 18 Jun 2012, *Fournier, J. GYJF12050* (HAST 147261); Maripasoula, Saül, trail head to Roche-Bateau, Crique-Cochon, 3.620498 N, 53.199309 W, 210–220 m, disturbed secondary rainforest, on dead branch, 22 Jun 2019, *Corriol, G. GYJF19179* (HAST 147262); Roura, Cacao, Camp Bonaventure, 4.323044 N, 52.339371 W, 59 m, disturbed hygrophilic rainforest, on dead branchlet, 20 Mar 2021, *Fournier, J. GYJF21021* (HAST 147263), immature; Maripasoula, Saül, trail to Monts-La Fumée, tributary of Crique Queue-Hocco, 3.637173 N, 53.204727 W, ca. 260 m, disturbed mesophilic rainforest, on dead blackened wood, 4 Apr 2021, *Fournier, J. GYJF21416* (HAST 147264).

**Known distribution**: Mostly neotropical, with a few paleotropical records (Læssøe 1999).

**Comments**: Our observations, based on 28 collections from French Guiana, are in full agreement with those reported by Læssøe (1999). Because the holotype at PC and the isotypes at PC and UPS are immature, we designate an epitype. Despite the wide variation in both macro- and micromorphological features, also noted by Læssøe (1999), *X. comosa* is usually readily recognized in the field. Its swollen fertile heads, crowned with an apical cluster of conidial processes, are borne on filiform, flexible stipes and are well illustrated in the protologue (Montagne 1840).

*Xylaria comosoides* Læssøe has long been confused with *X. comosa* because of their very similar external morphology. These two species are distinguished primarily by ascospore morphology and dimensions, as well as germ slit structure (Læssøe 1999; this paper). Both occur in similar habitats, and confusion is especially likely when they grow in the same locality. Although they are easily distinguished under the microscope, field identification is often difficult. Fertile heads of *X. comosa* are usually smaller than those of *X. comosoides*, but the most reliable macroscopic distinction lies in the hairy coating of the stipes, which is present in the former but absent in the latter. In dried specimens, fertile heads of *X. comosa* usually remain intact, whereas the larger ones of *X. comosoides* often become hollow and split lengthwise.

Læssøe (1999) also discussed certain collections deviating from *X. comosa* by smaller, shortly stipitate stromata and wider ascospores, 31–37 × 10.3–14.5 µm, which he referred to as *X*. aff. *comosa*. In our material, subsessile to sessile stromata of *X. comosa* feature ascospores typical for the species. Other collections with tiny, short-stipitate stromata and wider ascospores are here assigned to *X. squamulosa* San Martín & J. D. Rogers.

**Fig. 12:**
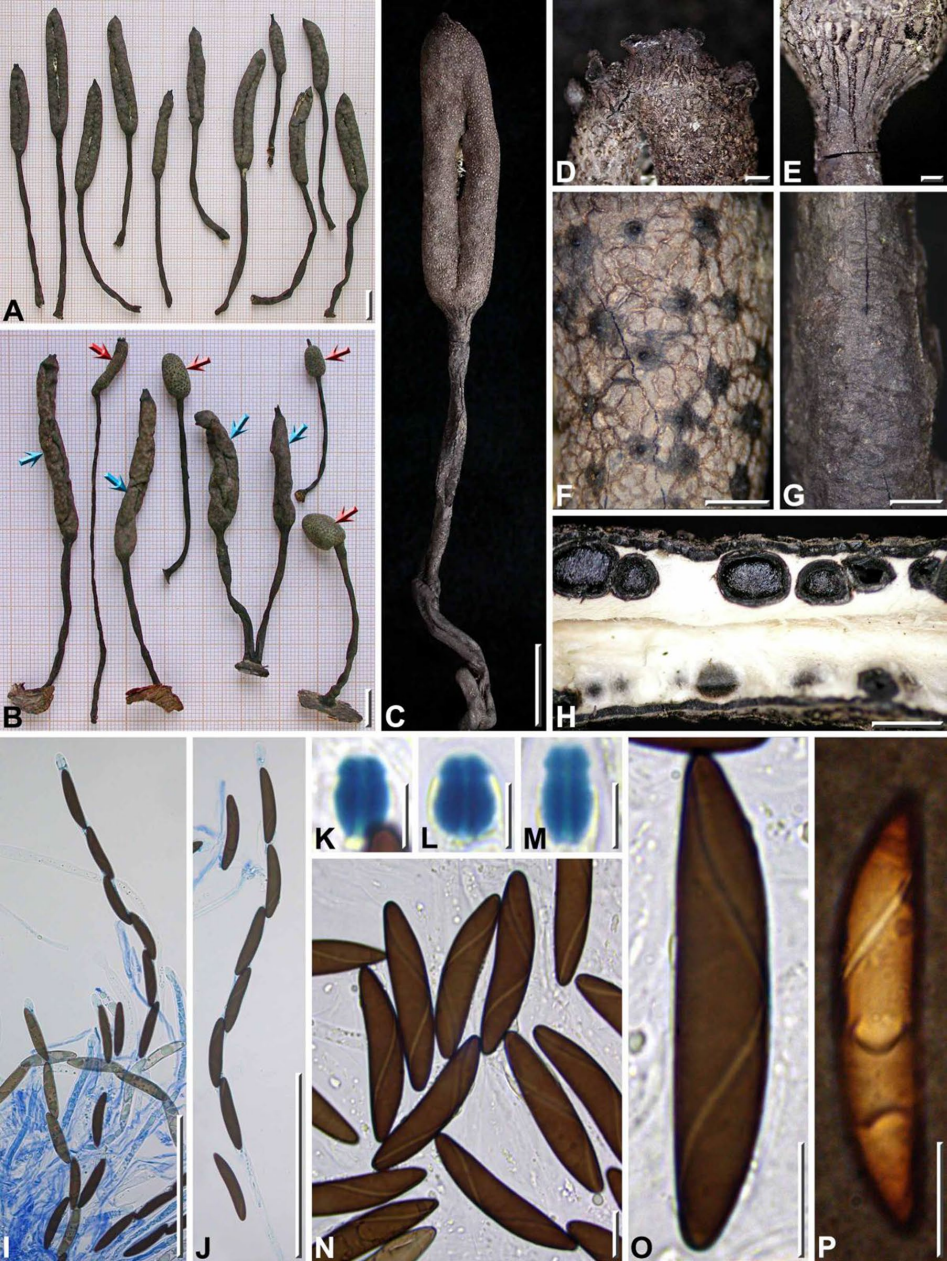
*Xylaria comosoides* (**A, C, K ***CLL8040*; **B, I, J, M–O ***GYJF21373*; **D, E, H ***CLL8010*; **F, G ***GYJF12023*; **L ***CLL9061*; **P ***CLL8141*). **A** Typical stromata. **B** Typical stromata (blue arrows) mixed with stromata of *X. comosa* (red arrows) collected together. **C** Apiculate, longitudinally split and inrolled stroma on a stout glabrous stipe. **D** Apex of a fertile head with multiple remnants of conidial processes. **E** Radially split surface at base of a fertile head. **F** Reticulately cracked stromatal surface with shiny black ostioles in blackened polygonal areas. **G** Surface of a stipe in close-up showing the absence of hairs or tomentum. **H** Stroma in longitudinal section showing perithecia beneath a thick black crust, embedded in a white fibrous interior. **I, J** Few-spored asci, in blue Pelikan ink. **K–M** Ascal apical apparati in Melzer’s reagent. **N, O** Ascospores showing spiraling germ slits, in 1% SDS. **P** Ascospore in India ink showing a lack of appendages. Bars in **A–C** = 10 mm; **D–H** = 1 mm; **I, J** = 100 µm; **K–M** = 5 µm; **N–P** = 10 µm

***Xylaria comosoides*** Læssøe, Kew Bulletin 54: 611. 1999. Figure 12. Table 8

**Table 8:** Ascospore and ascal apical apparatus dimensions in five collections of *X. comosoides* showing a wide variation range, compared with those from the protologue

Collection numbers	Ascospore measurements with extreme values in parentheses	Q = quotient l/w, *N* = number of measurements	Me = mean values of l/w, Qe = mean quotient l/w	Ascal apical apparatus h × w, *N* = 20
*CLL8040*	(38–)40.7–45.4(−47.3) × (7.0–)7.6–8.8(−9.2) µm	Q = (4.7–)4.8–5.7(−6.2), *N* = 60	Me = 42.8 × 8.1 µm, Qe = 5.3	5.9–6.4 × 3.8–4.3 µm, Me = 6.2 × 4.0 µm
*CLL8141*	(34.1–)35.6–40.1(−42.7) × (5.9–)6.3–7.3(−7.4) µm	Q = (4.8–)5.1–6.1(−7.0), *N* = 60	Me = 37.6 × 6.8 µm, Qe = 5.5	6.2–6.9 × 3.9–4.6 µm, Me = 6.5 × 4.2 µm
*CLL9061*	(40.4–)43.2–49.8(−52.5) × (6.9–)7.6–8.9(−9.6) µm	Q = (4.8–)5.0–6.3(−6.8), *N* = 60	Me = 45.8 × 8.2 µm, Qe = 5.6	6.2–7.0 × 4.3–5.0 µm, Me = 6.6 × 4.6 µm
*GYJF12023*	(37.1–)40.6–47.7(−50.5) × (6.9–)7.3–8.8(−9.7) µm	Q = (4.1–)4.9–6.1(−6.7), *N* = 60	Me = 43.6 × 8.0 µm, Qe = 5.5	6.0–6.8 × 4.1–4.6 µm, Me = 6.5 × 4.4 µm
*GYJF21373*	(35.9–)37.7–42.5(−44.0) × (6.8–)7.1–8.2(−8.7) µm	Q = (4.6–)4.7–5.8(−6.2), *N* = 60	Me = 39.8 × 7.6 µm, Qe = 5.2	6.8–7.4 × 3.6–4.1 µm, Me = 7.1 × 3.8 µm
**Cumulated values**	**(34.1–)35.6–49.8(−52.5) × (5.9–)6.3–8.9(−9.7) µm**	**Q = (4.1–)4.7–6.3(−7.0), N = 300**	**Me = 41.9 × 7.7 µm, Qe = 5.4**	**5.9–7.4 × 3.6–5.0 µm, Me = 6.6 × 4.2 µm**
Læssøe (1999)	(37.5–)40–47.5(−50.8) × (5.6–)6.3–7.3(−8.6) µm	*N* = 80	Me = 43.8 × 6.8 µm, Qe = 6.4	6–8 × 4 µm

**Stromata** scattered on host surface, rarely connate at base, 48–90 mm in total height, usually long-stipitate, the fertile heads cylindrical, flattening and shrinking upon drying and becoming longitudinally split and inrolled, 13–45 mm high × 5–13 mm wide, occasionally apically furcate, straight to slightly curved; apices obtusely rounded, mostly apiculate or bearing upright, crest-like, often more or less broken remnants of conidial processes; the stipes 15–66 mm high, 2.0–5.0 mm wide, well-defined, carbonaceous, black, glabrous, irregularly deformed upon drying, finely cracked or wrinkled, with a slightly swollen base; upper part of the stipe crust typically radially split into narrow stripes upwardly oriented at the base of the fertile head. Surface grayish brown, with unexposed perithecial contours, with a thick, persistent, reticulately cracked superficial layer, forming small polygonal scales and becoming dark gray to blackish delimiting polygonal gray to blackish areas around ostioles; stromatal crust just beneath surface black, carbonaceous, 170–210 µm thick; interior white to cream-white, solid, fibrous, with a narrow light brown core, shrinking upon drying involving the longitudinal splitting of the fertile heads. **Perithecia** fully immersed, subglobose to slightly depressed-spherical, 0.7–1.0 mm diam. **Ostioles** bluntly papillate to conic-papillate, 80–100 µm diam at base, black to shiny black.

**Paraphyses** copious, hyphal, hyaline, remotely septate, simple, 6.0–7.5 µm wide at base, tapering to 1.0–1.5 µm wide, embedded in mucilaginous material. **Asci** fragmentary, often few-spored, cylindrical, with apical apparatus urn-shaped, slightly constricted at upper third with an obtuse lateral rim, 5.9–7.4 × 3.6–5.0 µm (Me = 6.6 × 4.2 µm, *N* = 100), strongly bluing in Melzer’s reagent. **Ascospores** (34.1–)35.6–49.8(−52.5) × (5.9–)6.3–8.9(−9.7) µm, Q = (4.1–)4.7–6.3(−7.0) *N* = 300 (Me = 41.9 × 7.7 µm, Qe = 5.4), narrowly fusiform, slightly inequilateral, straight to slightly ventrally concave, with mostly broadly rounded ends, with a conspicuous spiraling germ slit spore-length on the dorsal side, lacking sheath or appendages; epispore smooth.

**Asexual morph** present on apical conidial pegs and branched processes but soon vanishing and not investigated during this study.

**Specimens examined: FRENCH GUIANA.** Sinnamary, Paracou, CIRAD field centre, Guyaflux plot, 5.249218 N, 52.915431 W, lowland rainforest, on dead wood, 27 Feb 2007, *Lechat, C. CLL7070* (HAST 147265); Sinnamary, Paracou, CIRAD field centre, Guyaflux plot, 5.249218 N, 52.915431 W, on dead blackened wood, 24 Jun 2012, *Fournier, J. GYJF12172* (HAST 147266); Sinnamary, Paracou, CIRAD field centre, Guyaflux plot, 5.249218 N, 52.915431 W, on dead wood, 24 Apr 2009, *Lechat, C. CLL9062* (HAST 147267); Roura, Cacao, Camp Bonaventure, 4.323044 N, 52.339371 W, 59 m, disturbed hygrophilic rainforest, on blackened wood, 20 Mar 2021, *Fournier, J. GYJF21012* (HAST 147268); Sinnamary, Paracou, CIRAD field centre, botanical trail, 5.249218 N, 52.915431 W, lowland rainforest, on dead wood, 29 Apr 2008, *Lechat, C. CLL8028* (HAST 147269); Saint-Georges, forest trail from Savane-Roche, 3.977742 N, 51.868988 W, ca. 60 m, lowland rainforest, on dead wood, 21 Apr 2009, *Lechat, C. CLL9035* (HAST 147270); Régina, Nouragues natural reserve, Inselberg camp, 4.311240 N, 52.132789 W, ca. 300 m, hygrophilic rainforest, on dead blackened wood, 19 Jun 2012, *Fournier, J. GYJF12095* (HAST 147271); Régina, Nouragues natural reserve, Inselberg camp, 4.311240 N, 52.132789 W, ca. 300 m, hygrophilic rainforest, on dead blackened wood, 17 Jun 2012, *Lechat, C. GYJF12023* (HAST 147272); Matoury, Lamirande botanical trail, Mont Grand Matoury natural reserve, 4.868037 N, 52.345827 W, ca. 70 m, lowland rainforest, on dead wood, 28 Apr 2009, *Lechat, C. CLL9108* (HAST 147273); Maripasoula, Saül, trail to Monts-La Fumée, tributary of Crique Queue-Hocco, 3.637173 N, 53.204727 W, ca. 260 m, disturbed mesophilic rainforest, on dead blackened wood, 4 Apr 2021, *Fournier, J. GYJF21417* (HAST 147274); Maripasoula, Saül, trail to Monts-La Fumée, 3.634796 N, 53.205457 W, ca. 270 m, disturbed mesophilic rainforest, on dead blackened wood, 2 Apr 2021, *Thonnel, A. GYJF21373* (HAST 147275); Macouria, Risquetout Forest, trail to Saut-Léodate, 4.899372 N, 52.555061 W, ca. 55 m, lowland rainforest, on dead wood, 9 May 2008, *Lechat, C. CLL8141* (HAST 147276); Kourou, Sentier de la Montagne des Singes, 5.068808 N, 52.69478 W, lowland rainforest, on dead wood, 27 Apr 2008, *Lechat, C. CLL8010* (HAST 147277); Saint-Georges, forest trail from Savane-Roche, 3.977742 N, 51.868988 W, ca. 60 m, lowland rainforest, on dead wood, 23 Apr 2009, *Lechat, C. CLL9061* (HAST 147278); Sinnamary, Paracou, CIRAD field centre, Guyaflux plot, 5.249218 N, 52.915431 W, on dead wood, 30 Apr 2008, *Lechat, C. CLL8040* (HAST 147279); Roura, Cacao, Camp Bonaventure, 4.326815 N, 52.341431 W, 60–65 m, disturbed hygrophilic rainforest, on dead corticated branch, 22 Mar 2021, *Fournier, J. GYJF21068* (HAST 147280).

**Known distribution**: Amazonian rainforests (Læssøe 1999); reported in the present study from French Guiana for the first time.

**Comments**: *Xylaria comosoides* was recognized as a distinct species by Læssøe (1999), differing from *X. comosa* mainly by its larger, differently shaped ascospores, which possess a long spiraling germ slit and lack appendages. Both species share similar ecology, being occasionally found together on dead twigs or branches in the litter, and are therefore easily confused. A reliable diagnostic feature in the field is stipe diameter: less than 2 mm in *X. comosa* versus 2–5 mm diam in *X. comosoides*. Moreover, the stipe of *X. comosoides* is carbonaceous, more brittle, and lacks the hairy vestiture typical of *X. comosa*. Another useful character is observed in *X. comosoides* at the junction of the stipe and fertile head, where upwardly radiating stripes are formed around the base of the fertile head. In herbarium material, fertile heads of *X. comosoides* appear consistently flattened and split lengthwise, with inrolled margins.

Both species are fairly common in French Guiana, and it is likely that many previous records of *X. comosa* from Amazonian rainforests, based solely on external morphology, actually represent *X. comosoides*.

**Fig. 13:**
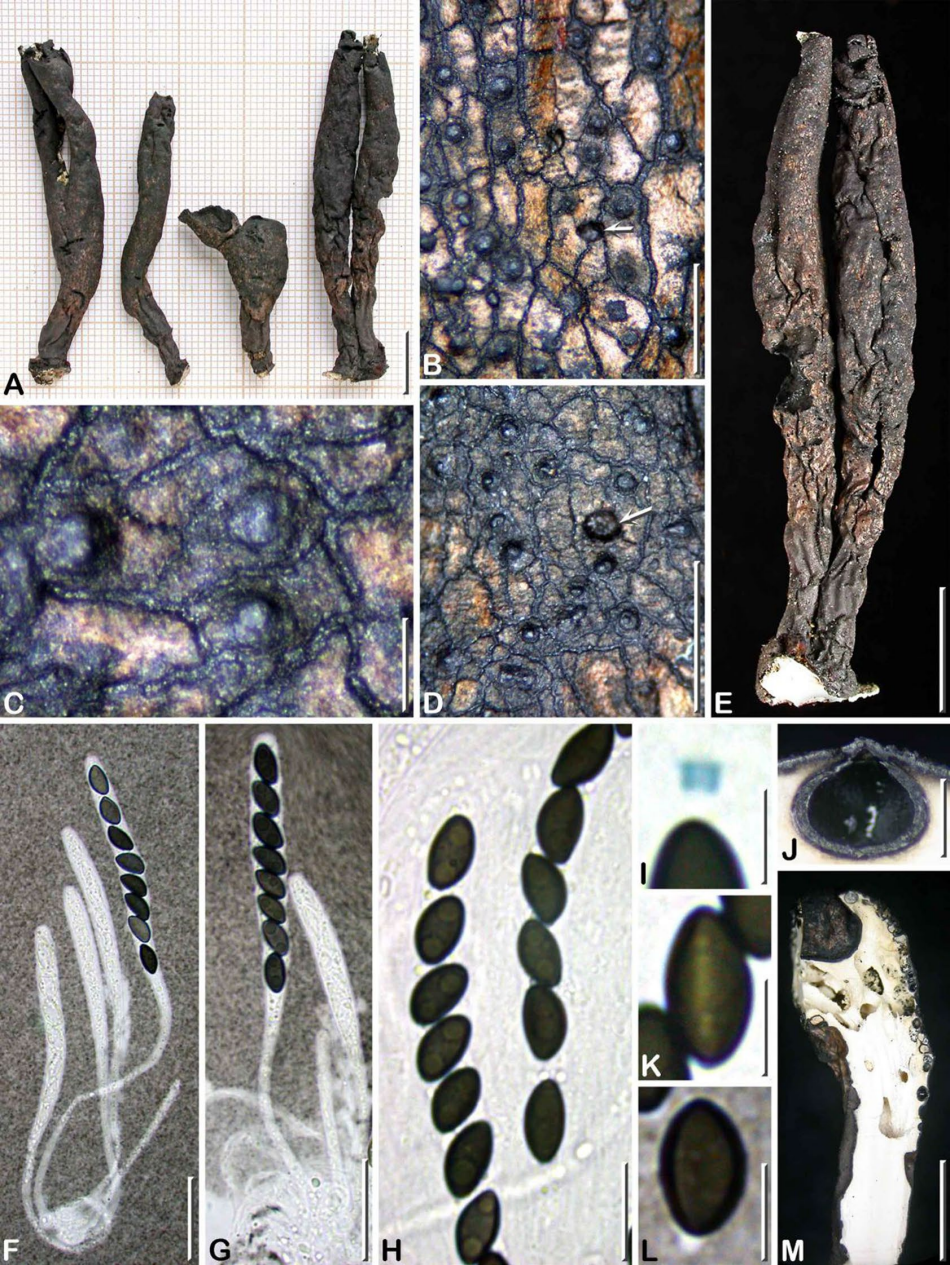
*Xylaria confragosa* (*GYJF19139* holotype). **A, E** Stromata in the dry state. **B** Stromatal surface showing cracks delimiting cream-colored to rust colored plaques, prominent ostioles and a pit (arrow). **C** Ostioles in close-up. **D** Blackening surface and a wide pit (arrow). **F, G** Immature and mature asci, in India ink. **H** Ascospores in 1% SDS. **I** Ascal apical apparatus in Melzer’s reagent. **J** Perithecium in vertical section. **K** Ascospore in ventral view showing a germ slit, in 1% SDS. **L** Ascospore in India ink showing a polar secondary appendage. **M** Stroma in longitudinal section, partly damaged by insects. Bars in **A, E** = 10 mm; **B, D** = 1 mm; **C** = 0.2 mm; **F, G** = 20 µm; **H** = 10 µm; **I, K, L** = 5 µm; **J** = 0.5 mm; **M** = 5 mm

***Xylaria confragosa*** J. Fourn., Y.-M. Ju, H.-M. Hsieh & Lechat, sp. nov. Figure 13

MycoBank MB863151

**Typification**: **FRENCH GUIANA.** Maripasoula, Saül, shortcut toward the airfield, 3.622159 N, 53.204166 W, disturbed secondary rainforest, on dead trunk, 20 Jun 2019, *Corriol, G. GYJF19139* (holotype HAST 147281).

**Etymology**: From Latin *confragosus =* rough, for the stromatal surface roughened by reticulate cracks, prominent ostioles, and pits.

**Diagnosis**: Differs from typical temperate *X. corniformis* by its more coarsely cracked surface in tones of orange-brown, larger ostioles, smaller subequilateral ascospores 7.3–8.1 × 3.8–4.5 µm, and its Neotropical distribution.

**Stromata** upright, separate or connate at base by two, subcylindrical to slightly fusiform, simple, terete, straight to slightly curved, longitudinally split with inrolled margins in the dry state, presumably with broadly rounded fertile apices, stipitate, 25–54 mm in total height, the fertile head 15–40 mm high × 6–9 mm diam, the stipe 12–15 mm high × 4–5 mm diam; surface dark orange-brown to blackish brown in places, with persistent outer layer reticulately cracked into polygonal scales coated with a vanishing cream-colored to light orange-brown pellicle, eventually blackish brown, with perithecial contours unexposed; with scattered rounded pits 150–340 µm diam; underlying crust black, leathery 50–70 µm thick; the stipes ill-defined, black, smooth, terete to flattened, glabrous, puckered, slightly swollen at base and overlain by golden brown to purple tomentum forming a collar at the very base; interior solid, spongy, white, grayish beneath the crust, largely disintegrated by insect larvae likely involving the shrinkage of stromata. **Perithecia** subglobose to depressed-spherical 0.6–0.85 mm diam. **Ostioles** obtusely papillate, black, on a low discoid base 150–210 µm diam.

**Paraphyses** hyphal, thin-walled, sparse, 6–7 µm wide at base, tapering to 1.5–2.0 µm wide above asci, embedded in mucilaginous material. **Asci** cylindrical, with (6–)8 slightly overlapping uniseriately arranged ascospores, the spore-bearing parts 53–63 × 5.5–5.8 µm, the stipes 70–110 µm long, with apical apparatus 1.1–1.4 × 1.6–2.0 µm (Me = 1.2 × 1.8 µm, *N* = 20), short-cylindrical, wider than high, with a faint upper rim, bluing in Melzer’s reagent. **Ascospores** (6.8–)7.3–8.1(−8.6) × (3.6–)3.8–4.5(−5.0) µm, Q = (1.5–)1.7–2.0(−2.2), *N* = 60 (Me = 7.7 × 4.1 µm, Qe = 1.9), ellipsoid slightly inequilateral with obtusely rounded ends, dark brown to blackish brown, unicellular, with a straight, longitudinally oriented germ slit almost spore-length on the less convex side, with a polar secondary appendage visible in India ink; epispore smooth.

**Asexual morph** on the natural substrate not observed.

**Known distribution**: French Guiana, known only from the holotype collection.

**Comments**: Based on its subclavate, leathery stromata with a reticulately cracked surface and conspicuous discoid ostioles, together with small, blackish brown ascospores with obtuse ends and an obscure germ slit, and asci bearing a small apical apparatus wider than high, *X. confragosa* resembles the north temperate *X. corniformis* (Fr.) Fr. The presence of pits on the stromatal surface, reminiscent of those encountered in *X*. *penicilliopsis* (Henn.) Y.-M. Ju [= *X. moelleroclavus* J. D. Rogers, Y.-M. Ju & Hemmes], is also noteworthy, although it may be incidental rather than taxonomically significant.

Aside from its tropical distribution, *X*. *confragosa* differs from *X. corniformis* mainly by its more orange-brown stromatal surface, larger ostioles, 150–210 µm diam vs 100–130, and smaller, subequilateral ascospores averaging 7.7 × 4.1 µm, compared to 9.2 × 4.8 µm in JF 11006 of *X*. *corniformis* (Fournier 2014). *Xylaria castorea* Berk., known from New Zealand, likewise differs by having larger ascospores 9.5–11 × 5–6 µm (Rogers and Samuels 1986).

The ITS sequence of *X*. *confragosa* shows a 97.74% similarity to JF11006. On this basis alone, some molecular taxonomists might apply the name *X*. *corniformis*. However, sequence similarities in three protein-coding loci are markedly lower (ACT 92.59%, RPB2 89.74, TUB2 89.62%), all substantially below the ITS value, arguing against conspecificity.

**Fig. 14:**
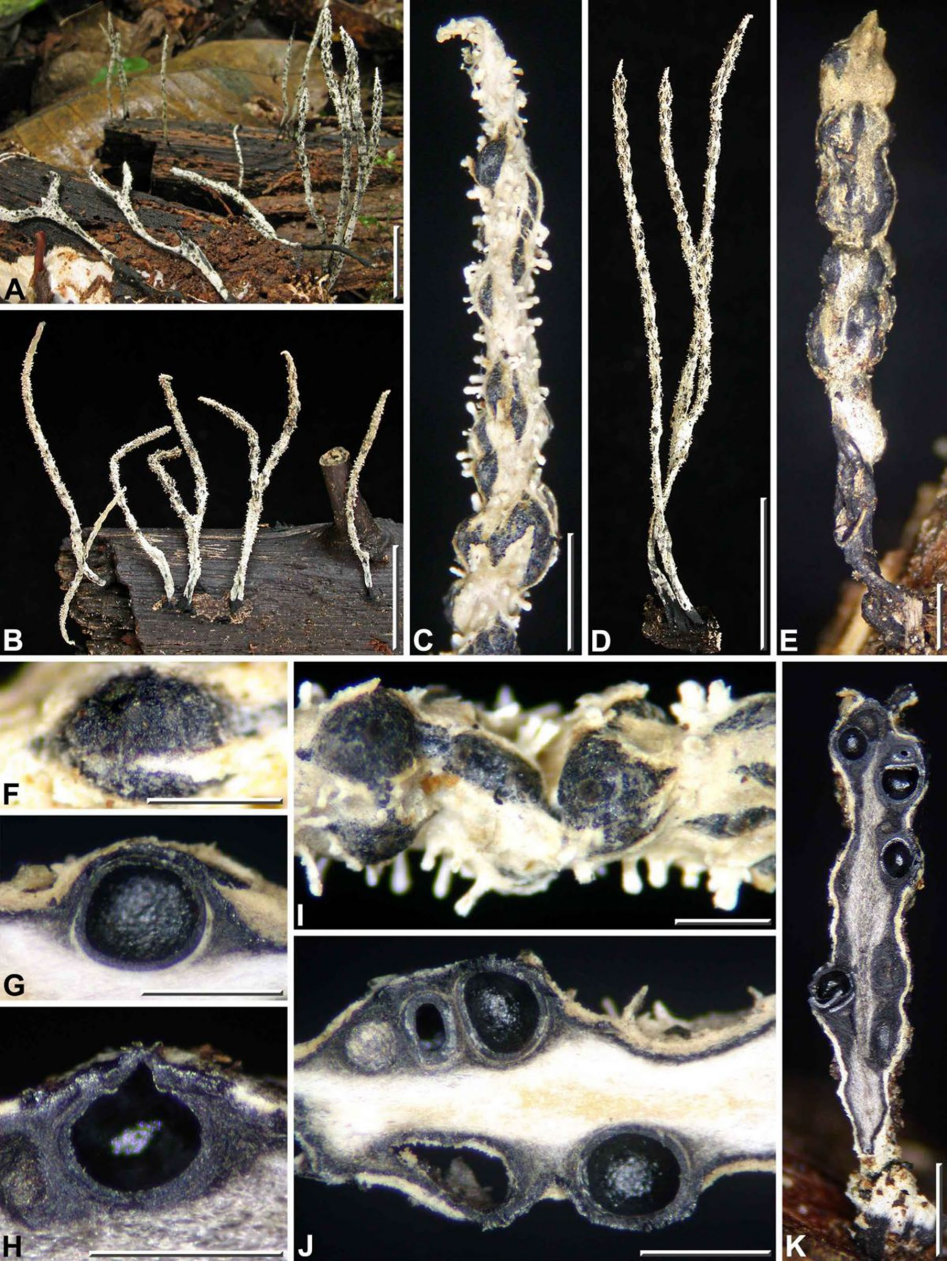
*Xylaria corriolii* (**A–D, F, G, I, J ***GYJF19151* holotype; **E, H, K ***GYJF21209*). **A** Stromata in the fresh state in situ (photo G. Corriol). **B, D** Stromata in the dry state. **C**, **I** Stromatal surface in close-up showing conidial pegs arising from the splitting outer layer. **E** Stroma lacking the associated asexual morph on surface. **F** Exposed perithecial contour showing a finely papillate ostiole. **G, H** Perithecia in vertical section. **J, K** Stromata in longitudinal section showing perithecia lying beneath a thin black crust and immersed in a discreet or well-developed black tissue, and a white or grayish interior. Bars in **A, B, D** = 10 mm; **C, E, K** = 1 mm; **F–J** = 0.5 mm

**Fig. 15:**
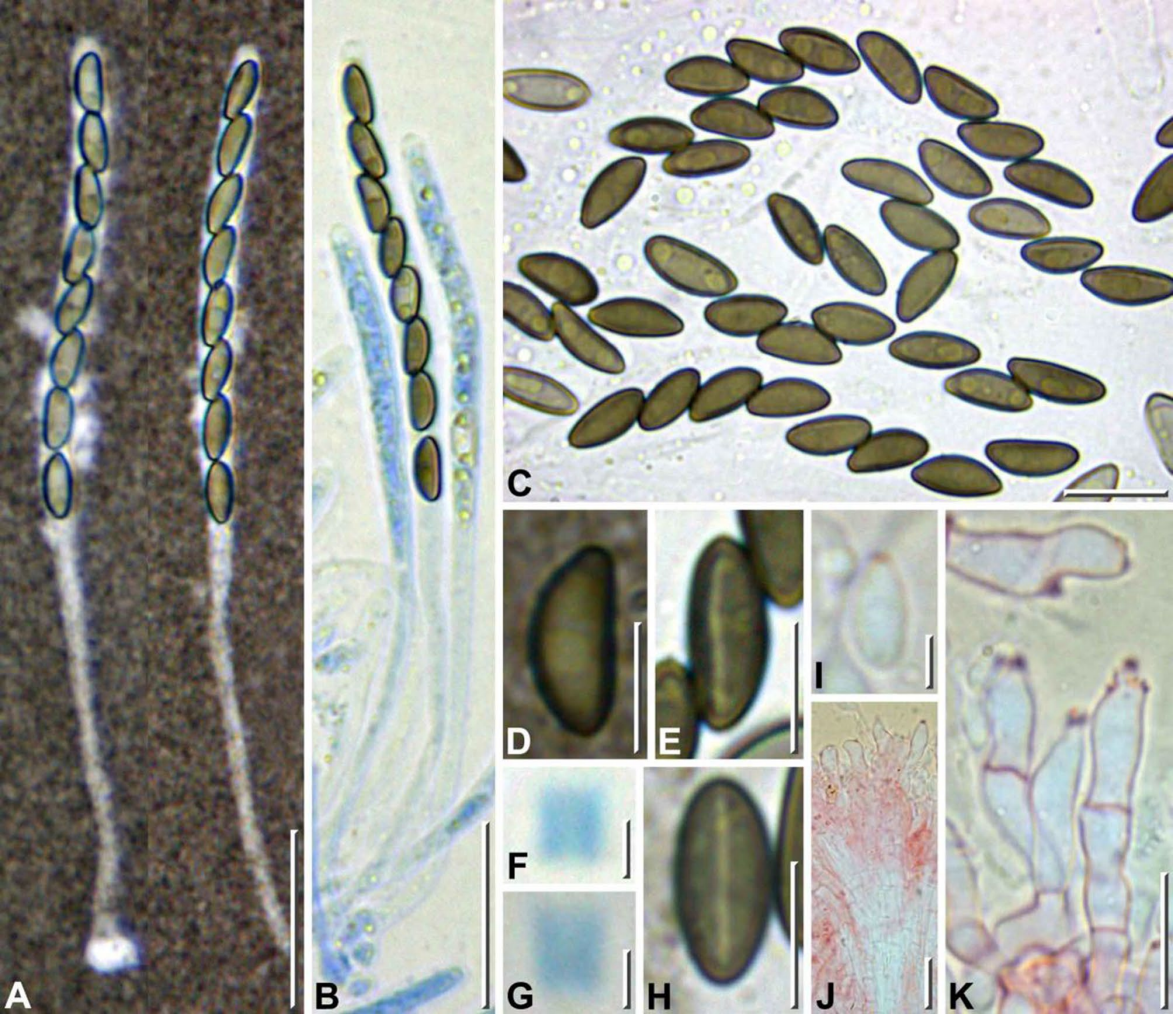
*Xylaria corriolii* (**A, C–F, I–K ***GYJF19151* holotype; **B, G, H ***GYJF21209*). **A** Immature and mature asci, in India ink. **B** Mature and immature asci, in blue Pelikan ink. **C** Ascospores mostly in side view, in 1% SDS. **D** Ascospore in India ink showing a lack of sheath or polar secondary appendages. **E, H** Ascospores in ventral view showing a germ slit, respectively in Melzer’s reagent and diluted India ink. **F, G** Ascal apical apparati, in Melzer’s reagent. **I** Conidium. **J** Palisadic conidiophores. **K** Conidiogenous cells with apical scars stained red (**I–K** in Congo red in 1% SDS). Bars in **A, B, J** = 20 µm; **C, K** = 10 µm; **D, E, H** = 5 µm; **F, G** = 1 µm; **I** = 2 µm

***Xylaria corriolii*** J. Fourn., Y.-M. Ju, H.-M. Hsieh & Lechat, sp. nov. Figures 14, 15, Table 9

**Table 9:** Ascospore dimensions in two collections of *X. corriolii* showing a fairly wide range of intraspecific variations

Collection numbers	Ascospore measurements with extreme values in parentheses	Q = quotient l/w, *N* = number of measurements	Me = mean values of l/w, Qe = mean quotient l/w
*GYJF19151* holotype	(6.6–)7.1–8.4(−8.7) × (2.7–)2.8–3.4(−3.6) µm	Q = (2.0–)2.3–2.8(−3.1), *N* = 60	Me = 7.8 × 3.1 µm, Qe = 2.6
*GYJF21209*	(5.9–)6.4–7.3(−8.1) × (2.6–)2.7–3.1(−3.2) µm	Q = (2.0–)2.2–2.6(−2.8), *N* = 60	Me = 6.8 × 2.9 µm, Qe = 2.4
**Cumulated values**	**(5.9–)6.4–8.4(−8.7) × (2.6–)2.7–3.4(−3.6) µm**	**Q = (2.0–)2.2–2.8(−3.1), N = 120**	**Me = 7.3 × 3.0 µm, Qe = 2.4**

MycoBank MB863152

**Typification**: **FRENCH GUIANA.** Maripasoula, Saül, trail head to Sentier des Gros Arbres, Crique Grand-Fossé, 3.617017 N, 53.209066 W, disturbed secondary rainforest, on rotten blackened wood and dead blackened decorticated branchlet, 21 Jun 2019, *Corriol, G. GYJF19151* (holotype HAST 147282).

**Etymology**: In honor of our friend Gilles Corriol, the French mycologist who collected the holotype material.

**Diagnosis**: Differs from related *Xylaria* species by its slightly nodulose stromata with mucronate sterile apices, a thick, white to yellowish splitting outer layer occasionally bearing densely distributed coremial pegs of the asexual morph, perithecia embedded in blackish tissue, and light brown ascospores averaging 7.3 × 3.0 µm.

**Stromata** upright to prostrate, separate to clustered by 2–3, filiform to narrowly cylindrical, simple to forked, terete to flattened in places, straight to slightly curved, with acutely mucronate sterile apices, short-stipitate, 6–46 mm in total height, the fertile head 0.8–1.3(−2.2) mm diam, the stipe; surface nodulose due to half-exposed contours of isolated perithecia or small clusters of perithecia, overlain by a thick, persistent, fibrous outer layer pure white when fresh, turning yellowish white to cream-colored and eventually yellow upon drying, splitting around perithecial contours, occasionally bearing conspicuous and densely distributed peg-like coremia all over the fertile head; underlying crust leathery, black, 20–50 µm thick, finely roughened by minute granules; the stipes ill-defined, 1–11 mm high × 1–1.5 mm diam, black, terete to flattened, glabrous, puckered, slightly swollen at base; interior solid, dense, black beneath surface and around perithecia, white to light gray and loosely fibrous at center. **Perithecia** loosely distributed, subglobose to slightly depressed-spherical, 0.35–0.45 mm diam. **Ostioles** mostly inconspicuous finely papillate to obtusely rounded, 50–70 µm wide at base, black, occasionally with a shiny black papilla.

**Paraphyses** hyphal, thin-walled, sparse, 2.0–3.0 µm wide at base, tapering to 0.8–1.0 µm wide above asci, discreetly embedded in mucilaginous material. **Asci** cylindrical, with (6–)8 slightly overlapping uniseriately arranged ascospores, the spore-bearing parts 41–57 × 4.0–5.5 µm, the stipes 34–78 µm long, fragile, with a minute apical apparatus 1.0–1.6 × 0.9–1.3 µm (Me = 1.3 × 1.1 µm, *N* = 45), short-cylindrical with a faint upper rim, quadrate in outline, bluing in Melzer’s reagent.

**Ascospores** (5.9–)6.4–8.4(−8.7) × (2.6–)2.7–3.4(−3.6) µm, Q = (2.0–)2.2–2.8(−3.1), *N* = 120 (Me = 7.3 × 3.0 µm, Qe = 2.4), narrowly ellipsoid, slightly inequilateral, with narrowly rounded ends, light to medium brown with olivaceous tone when fresh, unicellular, with a conspicuous, straight, longitudinally oriented germ slit almost spore-length on the ventral side, occasionally short or on the more convex side, without sheath or appendages; epispore smooth.

**Asexual morph** present on immature and mature stromata, comprised of peg-like coremia, cylindrical with slightly swollen apex, 120–200 µm high, 60–80 µm diam, arising from the white outer layer, concolorous, densely distributed down to the stipe. Terminal conidiogenous cells palisadic, barely prominent, cylindrical to fusoid, straight, simple to rarely furcate, 11–19 × 3.0–4.0 µm, bearing terminal denticulate conidial secession scars strongly stained by Congo red; conidia very rare, the only conidium that could be clearly observed was ellipsoid, hyaline, smooth, 4.4 × 2.0 µm, with a secession scar stained by Congo red.

**Other specimen examined (paratype)**: **FRENCH GUIANA.** Maripasoula, Saül, trail head to Sentier des Gros Arbres, 3.6201 N, 53.207989 W, ca. 210 m, disturbed mesophilic rainforest, on dead corticated branchlet 15 mm diam, 28 Mar 2021, *Fournier, J. GYJF21209* (HAST 147283).

**Known distribution**: French Guiana, known only from two collections.

**Comments**: This elegant *Xylaria* is distinctive in having slender, nodulose, and mucronate stromata, overlain by a thick, persistent, white to yellowish splitting outer layer occasionally bearing coremia all over its surface. The two cited collections differ in stromatal dimensions, presence of a superficial asexual morph, and degree of melanization of the stromatal interior. However, they share a similar outer layer, similar black tissue around perithecia, and ascospores of similar color, shape, and germ slit length, in spite of spanning a fairly wide range of dimensions (Table 9). Despite these slight differences, the combination of morphological characters is unique, and phylogenetic evidence strongly supports the recognition of *X. corriolii* as a consistent and distinct species.

*Xylaria corriolii* resembles *X. coccophora* in its nodulose outline and a yellowish outer layer. However, the outer layer of *X*. *corriolii* is uniformly cream-colored in contrast to the bipartite structure of *X. coccophora*, which is outwardly gray to tan and inwardly sulphur yellow (Fournier et al. 2020). The ascospores also differ, being smaller, 6.4–8.4 × 2.7–3.4 µm vs. 9.1–10.8 × 3.6–4.1 µm, and lighter in color. Our phylogenetic analyses confirm the distinctiveness of *X. corriolii* relative to *X. coccophora*. *Xylaria subcoccophora* San Martín & Lavin was also considered for its morphological affinities with *X. coccophora*, but it can be distinguished by a thin, white peeling outer layer, a white interior, and significantly larger ascospores, 10–11(−11.5) × 4–5 µm (San Martín et al. 2001).

The asexual morph on mature stromata of the holotype collection is reminiscent of that of *X. meliacearum* Læssøe as documented by Læssøe and Lodge (1994). Like in *X. corriolii*, the presence of conidial pegs in *X. meliacearum* was reported as inconstant across collections, making this feature difficult to use as a reliable diagnostic character. Our single collection of *X. meliacearum* (this paper) lacks conidial pegs, but, fortunately, the ascospores of both species readily distinguish them.

**Fig. 16:**
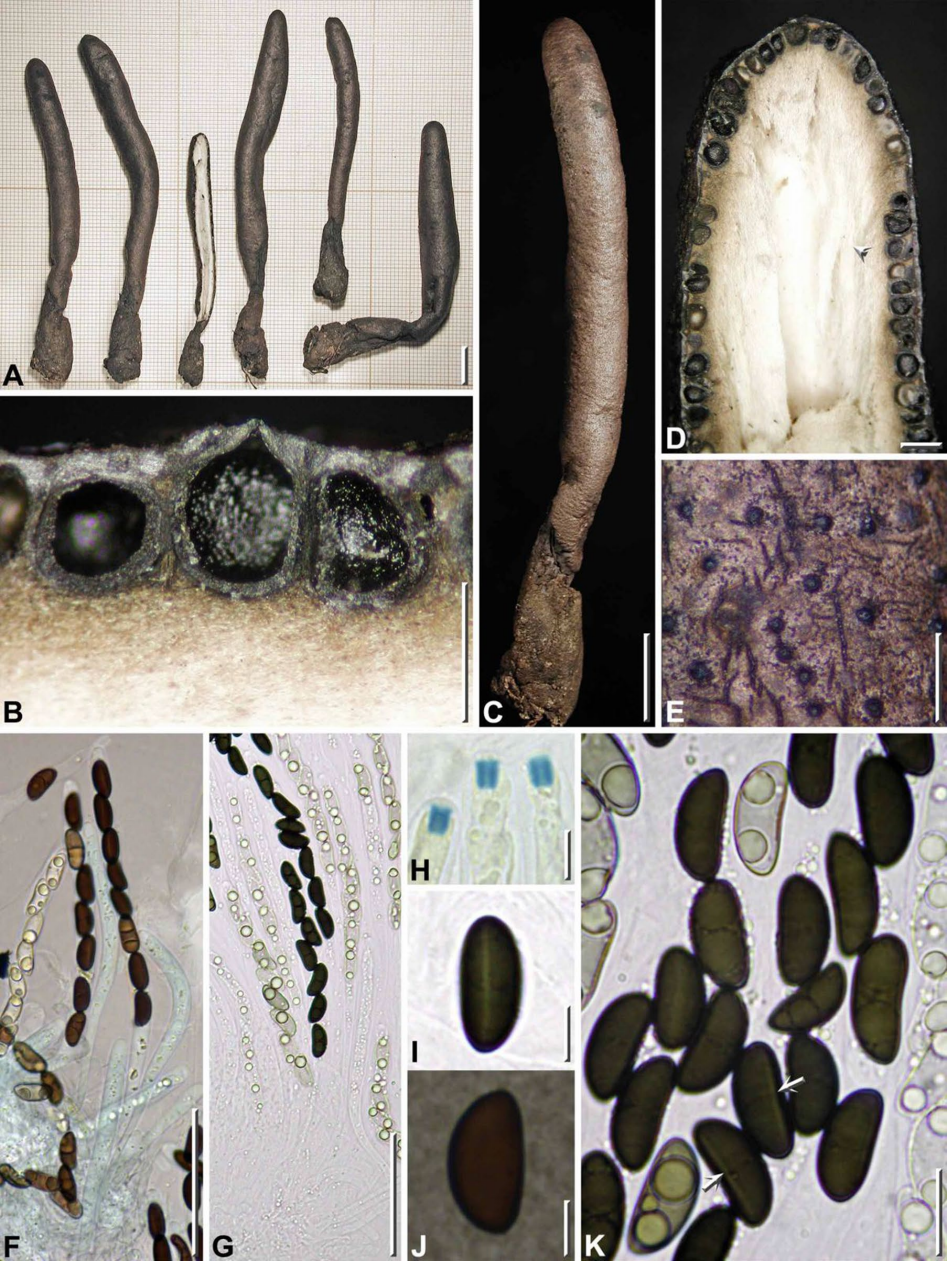
*Xylaria crassipes* (*CLL9022* holotype). **A** Habit of stromata in the dry state, showing a wide pannose base. **B** Longitudinal section of stromatal apex showing perithecia immersed in brownish tissue beneath a thick carbonaceous crust. **C** Stroma with a thick basal sleeve. **D** Vertical section of stromatal apex showing a solid white fibrous interior and fully immersed perithecia. **E** Stromatal surface in close-up showing ostioles and a loose network of shallow cracks. **F** Mature asci in chlorazol black. **G** Mature and immature asci in 1% SDS. **H** Ascal apical apparati in Melzer’s reagent. **I** Ascospore in ventral view showing a faint germ slit. **J** Ascospore in side view in India ink showing an absence of sheath or appendage. **K** Ascospores, those in ventral view showing a germ slit (arrows), in 1% SDS. Bars in **A, C** = 10 mm; **B, E** = 0.5 mm; **D** = 1 mm; **F, G** = 50 µm; **H–J** = 5 µm; **K** = 10 µm

***Xylaria crassipes*** J. Fourn., Y.-M. Ju, H.-M. Hsieh & Lechat, sp. nov. Figure 16

MycoBank MB863159

**Typification**: **FRENCH GUIANA.** Saint-Georges, forest trail from Savane-Roche, 3.977742 N, 51.868988 W, ca. 60 m, lowland rainforest, on dead wood, 21 Apr 2009, *Lechat, C. CLL9022* (holotype HAST 147284).

**Etymology**: From Latin *crassus* = thick and *pes* = foot, stipe, for the conspicuously swollen base of stipes.

**Diagnosis**: Differs from other *Xylaria* species with robust cylindrical stromata by the combination of a copper-brown, loosely reticulate surface, a carbonaceous crust, a solid white interior, and subreniform ascospores averaging 13.1 × 5.9 µm, with broadly rounded ends and a straight, nearly spore-length germ slit.

**Stromata** 62–95 mm in total height including short pannose stipes, upright, the fertile parts 6–11 mm wide, subcylindrical with broadly rounded fertile apices, simple, straight to slightly curved, hard-textured, not splitting upon drying; the stipes well-defined, 15–20 mm high, black, almost entirely sheathed in a conical sleeve 7–11 mm wide at base, composed of tightly aggregated, soft, dark orange-brown prosenchymatous tissue; surface copper-brown to dark brown, smooth, perithecial contours unexposed, just roughened by ostioles and narrow, shallow, randomly distributed linear to sinuous cracks forming a loose network; stromatal crust black, carbonaceous, 100–120 µm thick; the tissue around and beneath perithecia brownish, interior solid, whitish, fibrous to spongy. **Perithecia** immersed, subglobose, 0.5–0.6 mm diam, mostly in contact. **Ostioles** rounded, obtusely papillate, black, 70–85 µm diam.

**Paraphyses** hyphal, hyaline, remotely septate, 3.0–4.5 µm wide at base, tapering to 0.8–1.5 µm wide above asci, lacking mucilaginous coating. **Asci** cylindrical, with (6–)8 uniseriately arranged, slightly overlapping ascospores, the spore-bearing parts 95–105 µm long × 7.0–8.5 µm wide, the stipes 80–90 µm long, fragile, with apical apparatus short-cylindrical with a faint upper rim, 2.8–3.2 × 1.9–2.4 µm (Me = 3.0 × 2.1 µm, *N* = 25), bluing in Melzer’s reagent. **Ascospores** (11.7–)12.3–13.8(−14.7) × (5.0–)5.3–6.4(−6.8) µm, Q = (1.9–)2.0–2.5(−2.6), *N* = 120 (Me = 13.1 × 5.9 µm, Qe = 2.2), ellipsoid-inequilateral with broadly rounded ends, mostly reniform, unicellular, dark to blackish brown, with a narrow, straight, longitudinally oriented germ slit almost spore-length on the ventral, rarely the dorsal side; lacking sheath or appendages; epispore smooth.

**Asexual morph** on the natural substrate not observed.

**Known distribution**: French Guiana, known only from the holotype collection.

**Comments**: *Xylaria crassipes* shares most morphological characters with *X. allantoidea* (Berk.) Fr., *X. cubensis* (Mont.) Fr., and *X. flabelliformis* (Schwein.) Berk. & M.A. Curtis, to which it is moderately related. The main differences from these widespread species lie in its subcylindrical stromata with a thick pannose stipe and larger, differently shaped ascospores (Fournier et al. 2019).

*Xylaria* cf. *allantoidea* from French Guiana was described and cultured by Callan and Rogers (1990), which differs by hollow, inrolled stromata 8–15 mm diam on a long, narrow stipe 2–3 mm wide, and narrowly ellipsoid ascospores 12–13.5(−14) × 4–4.5(−5) µm.

*Xylaria nigromedullosa* Trierveiler-Pereira & A.I. Romero, described from Brazil by Trierveiler-Pereira et al. (2009), features dark brown, cylindro-clavate stromata. It is distinguished by a blackish solid interior and smaller, ellipsoid-inequilateral, blackish brown ascospores 7–9.5 × 4–5 µm. We document in this paper a slightly different collection of *X. nigromedullosa*, which likewise appears distinct from *X. crassipes.*

Two species with similar robust, cylindrical stromata are documented in this study. *Xylaria holmbergii* features dark brown, reniform ascospores that are smaller, averaging 8.9 × 4.5 µm, and leathery stromata with a corky-cracked surface. *Xylaria robusta* is phylogenetically the closest species (Fig. 1), featuring similar branched, carbonaceous stromata but differs mainly by ascospores with narrowly rounded to acute ends and a germ slit much less than spore-length.

**Fig. 17:**
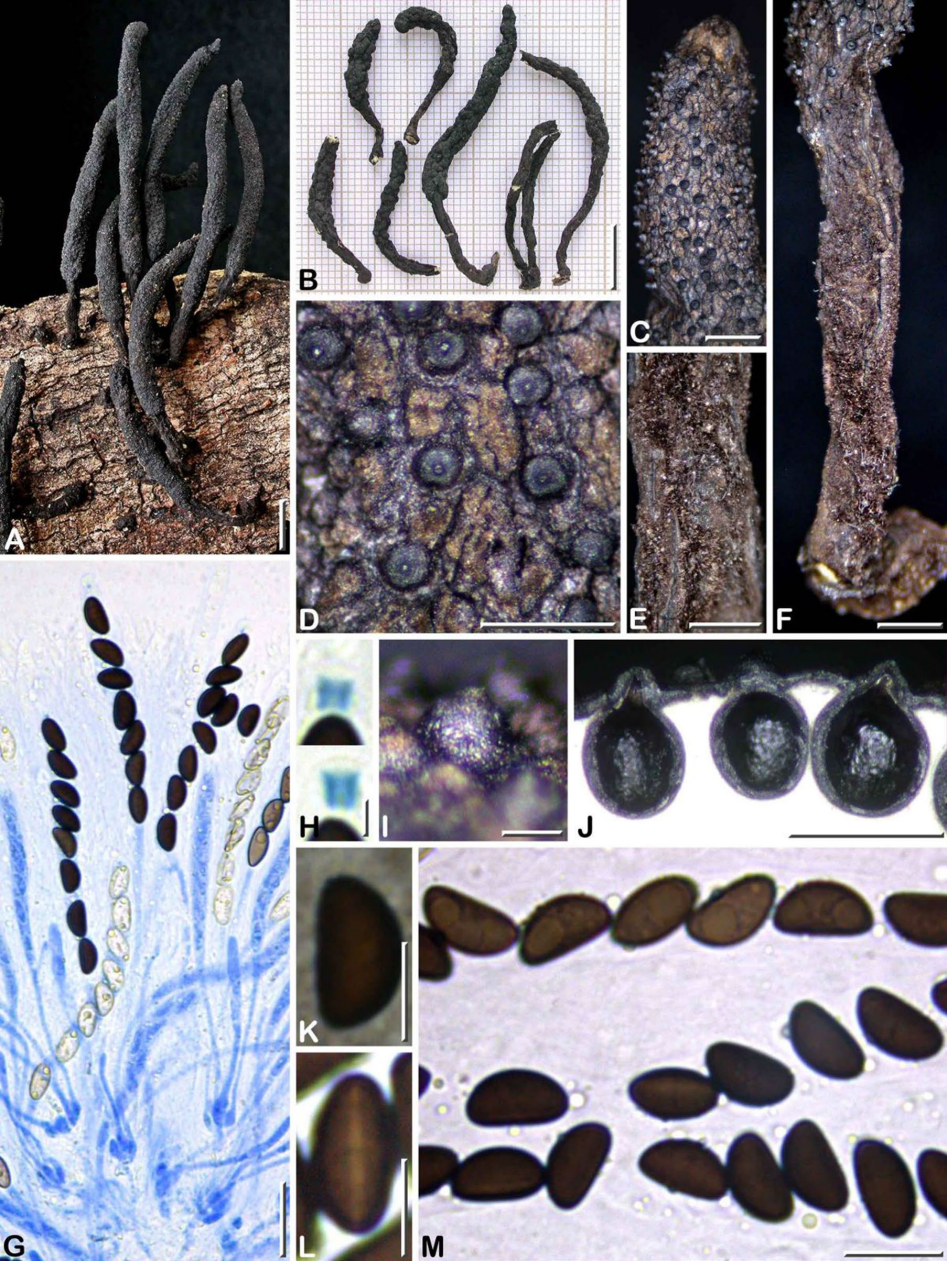
*Xylaria crustulata* (**A, C, D, F, I, J ***GYJF19024* paratype; **B, E, G, H, K–M ***GYJF21182* holotype). **A** Bunch of stromata arising from bark. **B** Variously bent stromata. **C** Stroma apically damaged, showing prominent ostioles. **D** Corky-cracked surface in close-up showing scales and ostioles. **E, F** Stipes surface in close-up showing tufts of short brown hairs. **G** Immature and mature asci, in blue Pelikan ink. **H** Ascal apical apparati in Melzer’s reagent. **I** Close-up on a prominent papillate ostiole in side view. **J** Stroma in vertical section showing perithecia immersed in a white tissue beneath a thin black crust and opening through projecting ostioles. **K** Ascospore in side view lacking a sheath or appendages, in India ink. **L** Ascospore in ventral view showing a germ slit, in Melzer’s reagent. **M** Immature and mature ascospores in side view, in 1% SDS. Bars in **A, B** = 10 mm; **C, E, F** = 1 mm; **D, J** = 0.5 mm; **G** = 20 µm; **H** = 2 µm; **I** = 100 µm; **K, L** = 5 µm; **M** = 10 µm

***Xylaria crustulata*** J. Fourn., Y.-M. Ju, H.-M. Hsieh & Lechat, sp. nov. Figure 17, Table 10

**Table 10:** Ascospore dimensions in three collections of *X. crustulata* showing a narrow variation range, compared with those of *X. rhytidophloea*

Collection numbers	Ascospore measurements with extreme values in parentheses	Q = quotient l/w, *N* = number of measurements	Me = mean values of l/w, Qe = mean quotient l/w
*GYJF19024*	(7.6–)7.9–9.4(−10.1) × (3.9–)4.3–4.8(−5.1) µm	Q = (1.6–)1.8–2.1(−2.4), *N* = 60	Me = 8.6 × 4.5 µm, Qe = 1.9
*GYJF19195*-2	(7.6–)8.1–9.2(−9.9) × (3.7–)4.2–4.9(−5.0) µm	Q = (1.6–)1.7–2.2(−2.3), *N* = 60	Me = 8.6 × 4.5 µm, Qe = 1.9
*GYJF21182* holotype	(7.8–)8.2–9.1(−9.6) × (4.3–)4.4–5.0(−5.1) µm	Q = (1.6–)1.7–2.0(−2.2), *N* = 60	Me = 8.6 × 4.7 µm, Qe = 1.8
**Cumulated values**	**(7.6–)7.9–9.4(−10.1) × (3.7–)4.2–5.0(−5.1) µm**	**Q = (1.6–)1.7–2.2(−2.4), N = 180**	**Me = 8.6 × 4.6 µm, Qe = 1.9**
*X. rhytidophloea* (this paper)	(7.5–)7.7–9.4(−10.1) × (4.0–)4.1–4.8(−5.1) µm	Q = (1.7–)1.8–2.1(−2.4), *N* = 120	Me = 8.6 × 4.5 µm, Qe = 1.9

MycoBank MB863160

**Typification**: **FRENCH GUIANA.** Maripasoula, Saül, trail head to Sentier des Gros Arbres, 3.6201 N, 53.207989 W, ca. 210 m, disturbed mesophilic rainforest, on dead corticated trunk, 27 Mar 2021, *Fournier, J. GYJF21182* (holotype HAST 147287).

**Etymology**: From Latin *crustulatus =* encrusted, covered with a crust, for the crust-like, roughened stromatal surface.

**Diagnosis**: Differs from *X. rhytidophloea* by hairy stipes, from *X. subplebeja* by smooth, uncracked stipes, and from other related species by lacking citrine-yellow tissue beneath the stromatal surface.

**Stromata** upright, scattered to gregarious, simple, occasionally connate at base by two, (6–)21–46 mm in total height, the fertile head (2.2–)5–32 mm high × 1.3–3.4 mm diam, cylindric to narrowly fusiform, rarely ellipsoid, straight to curved, apically rounded and fertile or faintly mucronate and sterile, with perithecial contours unexposed; surface blackish brown, glabrous, densely corky-cracked into minute, polygonal, isodiametric to elongated thick warts and roughened by prominent ostioles; the stipes well- to ill-defined, (1.5–)5–18 mm high × 0.6–2(−2.6) mm diam, straight to slightly curved, terete to flattened, dull brownish black, smooth apart from major cracks lengthwise, glabrous in places to covered with dense tufts of short brown hairs emerging from cracks, giving a downy appearance, at least in lower half, swollen at base; subsurface a black leathery crust 40–50 µm thick; interior solid, white, soft-textured. **Perithecia** subglobose, 0.35–0. 45 mm diam, laterally flattened when in contact. **Ostioles** finely papillate on a broadly rounded base, black, 120–170 µm diam, ca. 150 µm high, conspicuously prominent above the cracked superficial crust.

**Paraphyses** sparse, hyphal, remotely septate, thin-walled, 4.0–5.5 µm wide at base, tapering to 1.5–2 µm wide above asci, embedded in mucilaginous material. **Asci** cylindrical, with (6–)8 slightly overlapping uniseriately arranged ascospores, the spore-bearing parts 58–69 × 5.0–5.5 µm, the stipes 70–85 µm long, with apical apparatus short cylindric, apically flared and flattened with a faint lateral rim, 1.9–2.4 × 1.6–2.0 µm (Me = 2.1 × 1.8 µm, *N* = 50), bluing in Melzer’s reagent. **Ascospores** (7.6–)7.9–9.4(−10.1) × (3.7–)4.2–5.0(−5.1) µm, Q = (1.6–)1.7–2.2(−2.4), *N* = 180 (Me = 8.6 × 4.6 µm, Qe = 1.9), ellipsoid-inequilateral, with most often broadly rounded ends, dark brown, unicellular, with a conspicuous, straight, longitudinally oriented germ slit almost spore-length on the ventral side; lacking appendage or mucilaginous sheath; epispore smooth.

**Other specimens examined (paratypes)**: **FRENCH GUIANA.** Maripasoula, Saül, trail head of Sentier Roche-Bateau, 3.620498 N, 53.199309 W, disturbed secondary rainforest, ca. 240 m, on dead corticated branch, associated with *X. nelumboniformis*, 22 Jun 2019, *Fournier, J. GYJF19195-2* (HAST 147285); Maripasoula, Saül, shortcut to the airfield, 3.623524 N, 53.206542 W, ca. 240 m, disturbed secondary rainforest, on dead corticated trunk, mixed with *X. uncinata*, 17 Jun 2019, *Fournier, J. GYJF19024* (HAST 147286).

**Asexual morph** on the natural substrate not observed.

**Known distribution**: French Guiana, known from three collections.

**Comments**: Two collections of *X. crustulata* form a separate branch distant from *X. rhytidophloea*, with which they share most morphological characteristics, including ascospore dimensions (Table 10). The only differential trait is the presence of short brown hairs on the stipes of *X. crustulata*, conspicuous on most stromata. The collection *GYJF19195-2*, which features much smaller stromata, is referred to *X. crustulata* with reservation because it was not sequenced due to its scantiness and the stipe hairs are discreet and variable among stromata.

***Xylaria cubensis*** (Mont.) Fr., Nov. Act. Reg. Soc. Sci. Upsal., ser. III, 1: 126. 1851.

≡ *Hypoxylon cubense* Mont., Ann. Sci. Nat. Bot., sér. II, 13: 345. 1840.

The cited collections from French Guiana closely match those reported and illustrated from the French West Indies (Fournier et al. 2019).

**Specimens examined: FRENCH GUIANA.** Maripasoula, Saül, trail head toward Sentier des Gros Arbres, 3.620589 N, 53.208547 W, ca. 210 m, disturbed rainforest, on dead trunk, 22 Aug 2018, *Fournier, J. GYJF18072* (HAST 147288); Maripasoula, Saül, trail head of Sentier des Gros Arbres, Crique Grand-Fossé, 3.617198 N, 53.209029 W, ca. 210 m, disturbed secondary rainforest, on dead wood, 1 Apr 2021, *Fournier, J. GYJF21348* (HAST 147289); on wood, 1839, *Leprieur, F. R. 404* (syntype of *Hypoxylon cubense* PC 0086069 ex Montagne herb., isosyntype K(M) 169673 ex Montagne herb., isosyntype PC ex Leprieur herb.); on wood, 1839, *Leprieur, F. R. 403* (lectotype of *Hypoxylon cubense* PC 0086070 ex Montagne herb. 10103).

**Comments:** Although the epithet refers to Cuba, the name was first published by Montagne (1840) who cited three specimens from French Guiana: *Leprieur 403*, *404*, and *233*. Later, Montagne (1855) cited *Leprieur 403* only, which is thus considered the lectotype. *Leprieur 233* does not represent *X*. *cubensis*; it has a spiral germ slit on the ascospores, and its ascospores measure 36–39 × 7–8 μm (Dennis 1956).

**Fig. 18:**
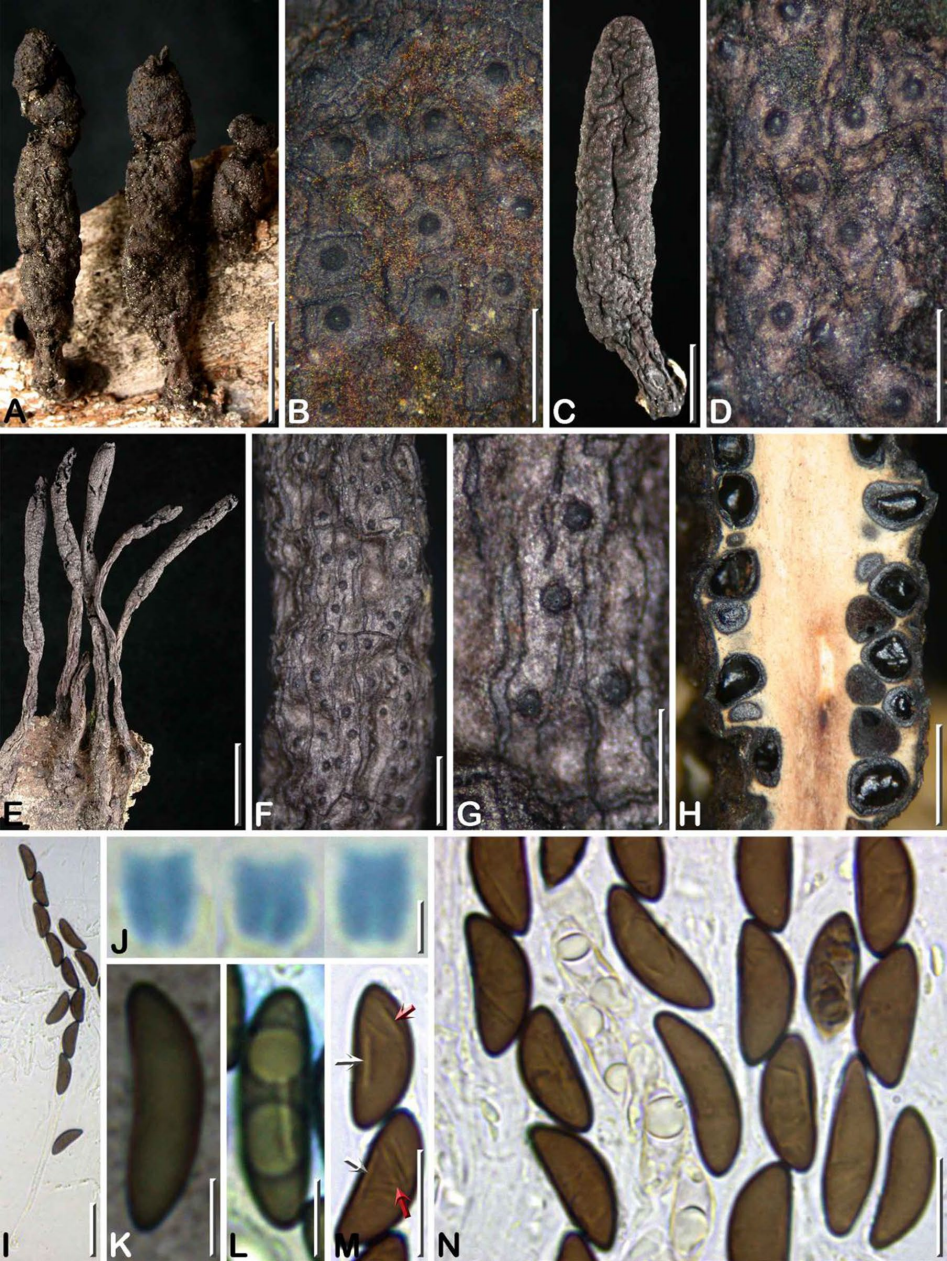
*Xylaria cuneata* (**A, B, H, I, M, N ***GYJF18149*; **C, D, K, L ***GYJF21207*; **E–G ***GYJF21393*; **J** *GYJF21381*). **A, C, E** Short- and long-stipitate mature stromata. **B, D, F, G** Stromatal surface in close-up varying from orange-brown to brownish gray, cracked into polygonal to elongate scales, and black rounded-papillate ostioles. **H** Stroma in vertical section showing a compact, white to orange-brown interior. **I** Relatively short-stipitate ascus, in 1% SDS. **J** Ascal apical apparati in Melzer’s reagent. **K** Ascospore in India ink showing a lack of appendages or mucilaginous sheath. **L** Ascospore in ventral view showing a slightly oblique germ slit, in blue Pelikan ink. **M** Ascospores showing a short ventral germ slit (white arrows) and rod-like elements showing through the epispore (red arrows). **N** Ascospores mostly in side view (**M** and **N** in 1% SDS). Bars in **A, C** = 5 mm; **B, D, G** = 0.5 mm; **E** = 10 mm; **F, H** = 1 mm; **I** = 20 µm; **J** = 2 µm; **K, L** = 5 µm; **M, N** = 10 µm

***Xylaria cuneata*** C.G. Lloyd, Mycol. Writings 7: 1180. 1923. Figure 18, Table 11

**Table 11:** Ascospore dimensions and ascal apical apparatus dimensions in five collections of *X. cuneata* from French Guiana, showing the range of intraspecific variations, compared with those reported in literature

Collection numbers	Ascospore measurements with extreme values in parentheses	Q = quotient l/w, *N* = number of measurements	Me = mean values of l/w, Qe = mean quotient l/w	Ascal apical apparatus h × w, *N* = 20
*GYJF18149*	(12.9–)13.4–15.3(−16.1) × (4.5–)4.8–5.5(−5.7) µm	Q = (2.4–)2.6–3.0(−3.3), *N* = 60	Me = 14.3 × 5.1 µm, Qe = 2.8	2.5–3.1 × 2.0–2.7 µm, Me = 2.9 × 2.3 µm
*GYJF21207*	(13.9–)14.5–16.4(−17.1) × (4.1–)4.4–5.0(−5.4) µm	Q = (2.9–)3.0–3.6(−3.9), *N* = 60	Me = 15.4 × 4.7 µm, Qe = 3.3	3.1–3.9 × 2.4–3.0 µm, Me = 3.5 × 2.8 µm
*GYJF21327-2*	(12.3–)13.3–14.8(−16.1) × (4.2–)4.5–5.1(−5.2) µm	Q = (2.4–)2.7–3.2(−3.4), *N* = 60	Me = 14.0 × 4.8 µm, Qe = 2.9	
*GYJF21381*	(12.1–)13.0–15.7(−18.5) × (4.4–)4.6–5.1(−5.4) µm	Q = (2.4–)2.6–3.3(−3.9), *N* = 60	Me = 14.1 × 4.8 µm, Qe = 2.9	2.7–3.7 × 2.1–2.6 µm, Me = 3.2 × 2.3 µm
*GYJF21393*	(12.8–)13.2–15.2(−17.6) × (4.2–)4.6–5.2(−5.3) µm	Q = (2.5–)2.7–3.2(−3.9), *N* = 60	Me = 14.1 × 4.9 µm, Qe = 2.9	
**Cumulated values**	**(12.1–)13.0–15.7(−18.5) × (4.2–)4.5–5.5(−5.7) µm**	**Q = (2.4–)2.6–3.6(−3.9), N = 300**	**Me = 14.4 × 4.9 µm, Qe = 3.0**	**2.5–3.9 × 2.0–3.0 µm, Me = 3.2 × 2.5 µm**
*X. cuneata* (Ju et al. 2016)	16–20 × 5–6 µm		Me = 18 × 5.5 µm, Qe = 3.3	
*X. montagnei* holotype (Ju et al. 2016)	13.5–16 × 4.5–6 µm		Me = 15 × 5.3 µm, Qe = 2.8	
FWI (Fournier et al. 2019)	(12.8–)13.1–17.3(−18.7) × (4.1–)4.6–5.5(−5.9) µm	Q = (2.4–)2.6–3.4(−3.8), *N* = 120	Me = 15 × 5 µm, Qe = 3.0	3.1–3.7 × 2.2–2.8 µm, Me = 3.4 × 2.6 µm

**Stromata** upright, scattered or in small clusters, (7–)12–48 mm in total height, the fertile head 4–23 mm high × 2–4 mm wide, broadly to narrowly cylindrical, occasionally subclavate, with obtusely rounded fertile apices, with perithecial contours unexposed, simple, straight to curved, becoming irregularly depressed or furrowed upon drying; surface dark orange-brown to grayish brown, with a persistent outermost layer reticulately cracked into polyhedral to elongated scales; fairly hard-textured, with subsurface a black leathery crust 50–70 µm thick; interior spongy, compact, whitish to yellowish, orange-brown in places, retracting upon drying; the stipes fairly well-defined, 3–28 mm high, 1.5–3 mm wide, dark brown to grayish brown, longitudinally cracked, glabrous, tomentose at base. **Perithecia** immersed, subglobose to depressed spherical or laterally flattened when in contact, 0.4–0.65 mm diam. **Ostioles** papillate, bluntly conical, black, 80–120 µm diam at base.

**Paraphyses** copious, hyphal, thin-walled, remotely septate, discretely guttulate, 4–7 µm wide at base, tapering to 1.5–2.0 µm wide above asci, embedded in mucilaginous material. **Asci** cylindrical, long-stipitate, with (4–6–)8 slightly overlapping uniseriately arranged ascospores, the spore-bearing parts 77–105 × 6.0–7.0 µm, the stipes 55–85 µm long, with apical apparatus 2.5–3.9 × 2.0–3.0 µm (Me = 3.2 × 2.5 µm, *N* = 60), short-cylindrical with a faint upper rim, a cupulate apex and straight to slightly convex sides, frequently attenuated at base, bluing in Melzer’s reagent. **Ascospores** (12.1–)13.0–15.7(−18.5) × (4.2–)4.5–5.5(−5.7) µm, Q = (2.4–)2.6–3.6(−3.9), *N* = 300 (Me = 14.4 × 4.9 µm, Qe = 3.0), ellipsoid strongly inequilateral to suballantoid, frequently ventrally concave, with narrowly to broadly rounded ends, medium brown, with a straight, central, longitudinally oriented to slightly oblique germ slit on the ventral side 4.5–6.0 µm long, without appendages or mucilaginous sheath; epispore smooth.

**Asexual morph** on the natural substrate not observed.

**Specimens examined: FRENCH GUIANA.** Maripasoula, Saül, trail head to Sentier des Gros Arbres, 3.620139 N, 53.208103 W, ca. 210 m, disturbed secondary rainforest, on dead decorticated wood, 25 Aug 2018, *Fournier, J. GYJF18149* (HAST 147290); Maripasoula, Saül, trail head to Sentier des Gros Arbres, 3.6201 N, 53.207989 W, ca. 210 m, on dead corticated branch, 28 Mar 2021, *Fournier, J. GYJF21207* (HAST 147291); Maripasoula, Saül, trail to Hmong village, 3.626053 N, 53.208075 W, 240–250 m, disturbed mesophilic rainforest, on dead corticated branch, 2 Apr 2021, *Fournier, J. GYJF21393* (HAST 147292); Maripasoula, Saül, trail head to Roche-Bateau, Crique-Cochon, 3.625994 N, 53.1955 W, 210–220 m, disturbed secondary rainforest, on dead corticated branch, 31 Mar 2021, *Fournier, J. GYJF21327-2* (HAST 147293); Maripasoula, Saül, trail to Monts-La-Fumée, 3.634796 N, 53.205457 W, ca. 270 m, disturbed mesophilic rainforest, on dead corticated branch, 2 Apr 2021, *Fournier, J. GYJF21381* (HAST 147294).

**Known distribution**: Neotropical (Fournier et al. 2019), including French Guiana (this paper).

**Comments**: The more collections referable to *X. cuneata* are studied, the less clear its morphological definition becomes. Compared with our two collections from the French West Indies (Fournier et al. 2019), the five collections from FG documented here substantially broaden the known range of variation in stromatal shape and size, as well as in surface color and cracking pattern. Despite these misleading morphological variations, our phylogenetic data strongly support their conspecificity. The main microscopic characteristic uniting them is the ascospore shape and size, combined with the presence of a short germ slit. Upon re-examining herbarium material, we observed the unexpected presence of refractive, rod-like elements visible through the epispore, a feature previously regarded as diagnostic of *X. rhytidosperma* J. Fourn. & Lechat, a penzigioid species with large, fusiform, inequilateral ascospores (Fournier et al. 2018c) that is not closely related to *X. cuneata*. This intriguing feature is most apparent after prolonged drying, which unfortunately limits its usefulness for studies on fresh material.

***Xylaria curta*** Fr., Nov. Act. Reg. Soc. Sci. Upsal., ser. III, 1: 126. 1851.

The cited collection from French Guiana closely matches those reported and illustrated from the French West Indies (Fournier et al. 2019).

**Specimen examined: FRENCH GUIANA.** Maripasoula, Saül, trail head to Sentier des Gros Arbres, 3.620839 N, 53.208536 W, ca. 210 m, disturbed secondary rainforest, on dead wood, 26 Mar 2021, *Fournier, J. GYJF21123* (HAST 147295).

***Xylaria cylindrica*** Lév., Ann. Sci. Nat. Bot., sér. IV, 20: 292. 1863; non Ellis & Everhart.

= *Xylaria fissilis* Ces., Atti Accad. Sci. Fis. 8: 16. 1879.

The cited collection from French Guiana closely matches those reported and illustrated as *X*. *fissilis* from the French West Indies (Fournier et al. 2020).

**Specimens examined: COLOMBIA.** Villeta, Alto del Trigo, on trunk, *Lindig 2928* (isotype of *X. cylindrica* K(M) 169676 ex Triana herb.). **FRENCH GUIANA.** Roura, Cacao, Camp Bonaventure, 4.330514 N, 52.338095 W, on blackened wood, 24 Mar 2021, *Fournier, J. GYJF21114* (HAST 147296).

**Fig. 19:**
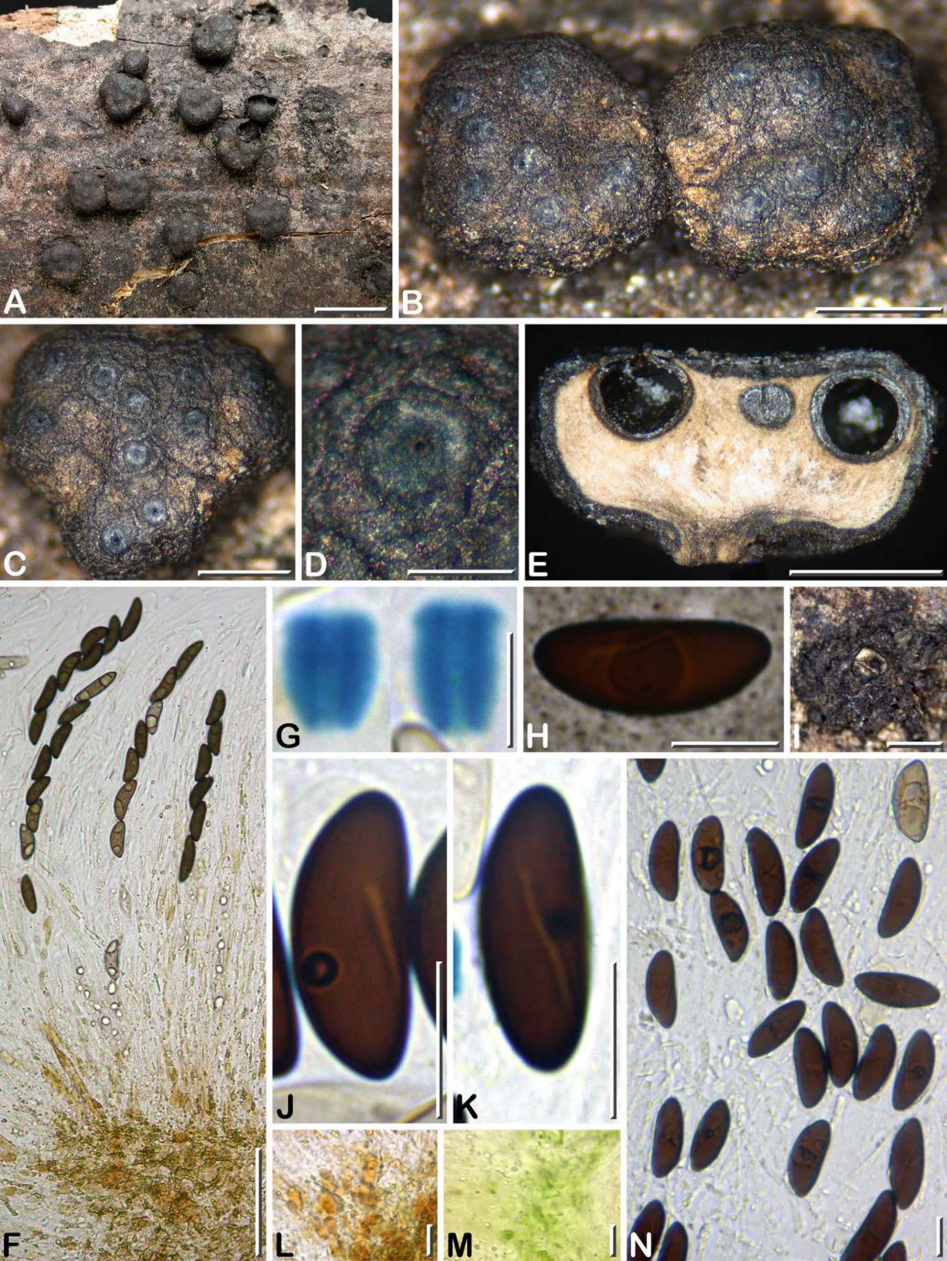
*Xylaria depressa* (**A–E, G–N ***GYJF18041* holotype; **F ***GYJF18129*). **A** Loosely distributed stromata on bark. **B, C** Stromata in top view. **D** Discoid ostiole in close-up. **E** Depressed stroma in vertical section showing a thick crust and a short basal connective. **F** Asci and basal hymenium interspersed with orange-brown amorphous material, in 1% SDS. **G** Ascal apical apparati, in Melzer’s reagent. **H** Ascospore in India ink showing an absence of sheath or appendages. **I** Blackish hyphal pad on bark surrounding a broken connective after removal of a stroma. **J, K** Ascospores in ventral view showing respectively straight and slightly sinuous germ slits, in Melzer’s reagent. **L, M** hymenial basal granules respectively in 1% SDS and 3% KOH. **N** Ascospores in 1% SDS. Bars in **A** = 5 mm; **B, C, E, I** = 1 mm; **D** = 0.2 mm; **F** = 50 µm; **G** = 5 µm; **H, J–N** = 10 µm

***Xylaria depressa*** J. Fourn., Y.-M. Ju, H.-M. Hsieh & Lechat, sp. nov. Figure 19, Table 12

**Table 12:** Ascospore dimensions in two collections of *X. depressa* showing a narrow range of intraspecific variations, compared with those reported worldwide for *X. atrosphaerica* in literature

Collection numbers	Ascospore measurements with extreme values in parentheses	Q = quotient l/w, *N* = number of measurements	Me = mean values of l/w, Qe = mean quotient l/w
*GYJF18041*	(16.0–)17.2–19.6(−20.4) × (5.6–)6.0–6.9(−7.1) µm	Q = (2.4–)2.6–3.1(−3.4), *N* = 60	Me = 18.3 × 6.5 µm, Qe = 2.8
*GYJF18129*	(15.3–)17.2–20.5(−21.8) × (5.9–)6.0–6.9(−7.2) µm	Q = (2.3–)2.5–3.3(−3.5), *N* = 60	Me = 18.6 × 6.5 µm, Qe = 2.9
**Cumulated values**	**(15.3–)17.2–20.5(−21.8) × (5.6–)6.0–6.9(−7.2) µm**	**Q = (2.3–)2.5–3.3(−3.5), N = 120**	**Me = 18.5 × 6.5 µm, Qe = 2.8**
Australia (Cooke 1894)			22 × 6(−8) µm
Sulawesi (Rogers et al. 1987)	17.6–22 × 7.3–9 µm		Me = 19.8 × 8.2 µm
FG (Callan and Rogers 1990)	(17–)18–20(−21) × 6–7 µm		Me = 19 × 6.5 µm
YMJ 92052313, Taiwan	(17.3–)19.5–22.6(−24.2) × (6.2–)6.9–8.0(−9.0) µm	Q = (2.1–)2.6–3.1(−3.4), *N* = 60	Me = 21.0 × 7.5 µm, Qe = 2.8

MycoBank MB863161

**Typification**: **FRENCH GUIANA.** Maripasoula, Saül, trail up to Hmong village, 3.6255 N, 53.208254 W, on dead corticated branch, 21 Aug 2018, *Fournier, J. GYJF18041* (holotype HAST 147297).

**Etymology**: from Latin *depressus* = lower, flattened, for the flat-topped often slightly concave stromata.

**Diagnosis**: Differs from *X. atrosphaerica* by flat-topped to slightly depressed stromata, broader ostiolar discs, the presence of rust-colored granules interspersed in the subhymenium, and slightly smaller ascospores averaging 18.5 × 6.5 µm.

**Stromata** in small groups, superficial, suborbicular, flat-topped to slightly concave, 1.3–3.4 mm diam × 1.3–1.8 mm high, subsessile, on a central narrow connective embedded in a black hyphal pad spread on bark surface. Upper surface blackish copper-brown, with a network of shallow cracks and remnants of a superficial tan to copper-brown pellicle; perithecial contours unexposed to faintly exposed; subsurface black, a carbonaceous crust 80–100 µm thick; interior off-white to brownish gray, darker in the connective, loosely fibrous. **Perithecia** subglobose, 0.5–0.6 mm diam. **Ostioles** bluntly papillate, porate, on a black discoid base 170–200 µm diam, barely prominent above stromatal surface.

**Paraphyses** hyphal, hyaline, remotely septate, simple, sparsely guttulate, containing conspicuous orange-brown amorphous material at the base, dissolving and turning green in 3% KOH, 6.0–7.0 µm wide at base, tapering to 0.8–1.5 µm wide above asci, discreetly embedded in mucilaginous material. **Asci** cylindrical, with (6–)8 uniseriately arranged, slightly to strongly overlapping ascospores, the spore-bearing parts 95–120 µm long × 8.5–10.0 µm wide, the stipes 85–105 µm long, with apical apparatus broadly cylindrical, basally attenuated, apically flattened with an obtuse, slightly convex rim, 4.7–5.6 × 3.2–3.9 µm (Me = 5.1 × 3.6 µm, *N* = 25), bluing in Melzer’s reagent. **Ascospores** (15.3–)17.2–20.5(−21.8) × (5.6–)6.0–6.9(−7.2) µm, Q = (2.3–)2.5–3.3(−3.5), *N* = 120 (Me = 18.5 × 6.5 µm, Qe = 2.8), ellipsoid-inequilateral with mostly broadly rounded ends, straight, ventrally flat to slightly concave, dark brown, with a short diagonal germ slit on the ventral side, 7.4–8.8 µm long, straight to slightly sinuous, without sheath or appendage visible in India ink; epispore smooth.

**Asexual morph** on the natural substrate not observed.

**Other specimen examined (paratype): FRENCH GUIANA.** Maripasoula, Saül, trail head to Sentier des Gros Arbres, Crique Grand-Fossé, 3.617197 N, 53.209042 W, on dead corticated branch, 24 Aug 2018, *Fournier, J. GYJF18129* (HAST 147298), largely depauperate.

**Specimens of** ***X. atrosphaerica*** **examined: AUSTRALIA.** Queensland, Brisbane, on dead stump, Jun 1918, *Bailey, F. M. 959* (holotype of *Hypoxylon atrosphaericum* K(M) 156839). **TAIWAN.** I-lan Co., Yuan-shan, Fu-shan, on bark of *Machilus thunbergii*, 23 May 2003, Ju, Y.-M. & Hsieh, H.-M. 92052313 (HAST).

**Known distribution**: French Guiana, known from this paper.

**Comments**: *Xylaria depressa* resembles *X*. *atrosphaerica* in having pulvinate stromata and a discoid area surrounding individual ostioles, but differs mainly by its smaller ascospores and the presence of conspicuous orange-brown hymenial granules. Our phylogenetic study further supports their distinction. The two collections of *X*. *depressa* that we examined correspond well with *X. atrosphaerica*, previously reported from French Guiana by Callan and Rogers (1990), in featuring small, flattened, subsessile stromata and ascospores of a similar size range, with a short diagonal germ slit. However, the conspicuous discoid base surrounding individual ostioles was not mentioned in Callan and Rogers (1990), nor were the orange-brown hymenial granules that we observed.

Although the holotype of *X. atrosphaerica* is in poor condition, which prevented Rogers et al. (1987) from observing ascospores, we were able to find a few ascospores averaging 22.6 × 7.9 µm, thereby reconfirming the ascospore size of “22 × 6 µ (rarely 8 µ)” given in the protologue (Cooke 1894).

**Fig. 20:**
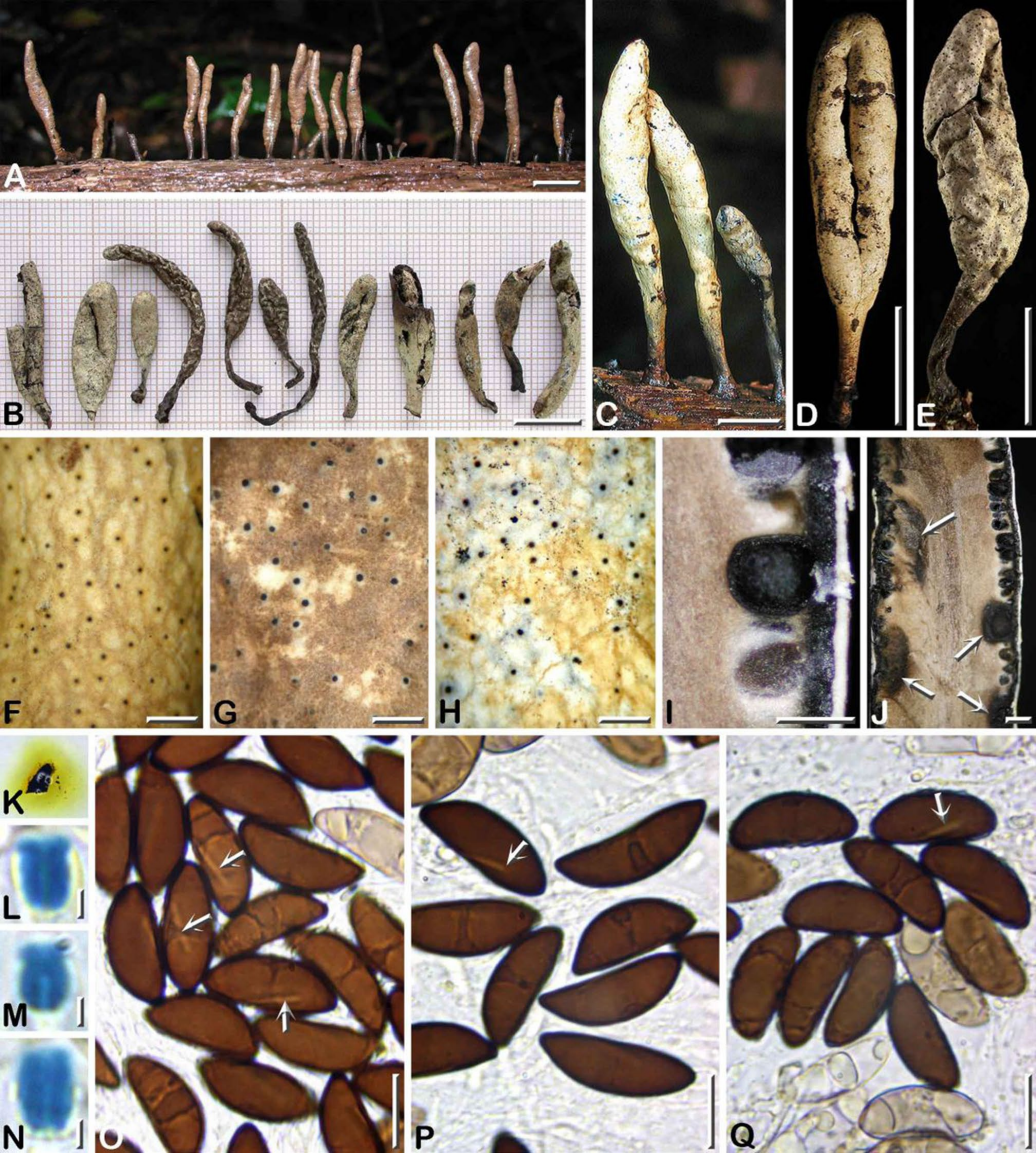
*Xylaria enterogena* (**A ***GYJF21241*; **B, L, O ***CLL7043*; **C, H ***GYJF21075*; **D, F, I–K ***CLL8002*; **E, G ***GYJF12051*; **M, P ***GYJF12068*; **N, Q ***GYJF21122*). **A** Immature wet stromata in the fresh state, in situ. **B** Variously shaped and colored stromata, those slenderer and darker ones at center immature. **C** Whitish and grayish stromata in the fresh state, in situ. **D, E** Respectively yellowish and brownish stromata in the dry state, more or less split lengthwise. **F–H** Variously colored stromatal surfaces. **I** Stromatal surface in vertical section showing perithecia immersed beneath a thick carbonaceous crust, overlain by a pure white granular layer. **J** Stroma in vertical section showing a solid brownish gray interior damaged by insect larvae (arrows). **K** KOH extractable pigments from the stromatal crust after 1 min incubation. **L–N** Ascal apical apparati, in Melzer’s reagent. **O–Q** Ascospores from three different collections in 1% SDS, some showing a germ slit (arrows). Bars in **A–E** = 10 mm; **F–I** 0.5 mm; **J** = 1 mm; **L–N** = 2 µm; **O–Q** = 10 µm

***Xylaria enterogena*** (Mont.) Fr., Nov. Act. Reg. Soc. Sci. Upsal., ser. III, 1: 127. 1851. Figure 20, Table 13

**Table 13:** Ascospore and ascal apical apparatus dimensions in ten collections of *X. enterogena* from French Guiana made during this study, showing their variation range, compared with those reported in literature for *X. enterogena* and *X. telfairii*

Collection numbers	Ascospore measurements with extreme values in parentheses	Q = quotient l/w, *N* = number of measurements	Me = mean values of l/w, Qe = mean quotient l/w	Ascal apical apparatus h × w, *N* = 25
*CLL7043*	(16.3–)16.7–19.5(−20.3) × (5.4–)5.9–7.0(−7.5) µm	Q = (2.3–)2.5–3.2(−3.8), *N* = 60	Me = 17.9 × 6.5 µm, Qe = 2.8	4.1–4.7 × 3.1–3.7 µm, Me = 4.4 × 3.3 µm
*CLL8002*	(15.1–)16.1–18.4(−19.4) × (5.6–)5.8–6.6(−6.8) µm	Q = (2.3–)2.6–3.0(−3.3), *N* = 60	Me = 17.4 × 6.2 µm, Qe = 2.8	3.9–4.6 × 3.1–3.7 µm, Me = 4.3 × 3.5 µm
*CLL8031*	(15.1–)16.0–18.7(−19.9) × (4.9–)5.4–6.4(−7.1) µm	Q = (2.4–)2.5–3.3(−3.7), *N* = 60	Me = 17.3 × 6.0 µm, Qe = 2.9	4.0–5.4 × 3.1–3.9 µm, Me = 4.7 × 3.4 µm
*GYJF12025*	(17.2–)17.8–21.2(−22.5) × (5.8–)6.0–6.8(−7.3) µm	Q = (2.6–)2.7–3.4(−3.6), *N* = 60	Me = 19.5 × 6.4 µm, Qe = 3.0	4.2–4.8 × 3.0–3.8 µm, Me = 4.5 × 3.4 µm
*GYJF12051*	(17.8–)18.3–21.2(−22.2) × (5.9–)6.4–7.3(−7.6) µm	Q = (2.5–)2.7–3.1(−3.5), *N* = 60	Me = 19.6 × 6.8 µm, Qe = 2.9	4.5–5.1 × 3.3–4.0 µm, Me = 4.7 × 3.6 µm
*GYJF12068*	(17.6–)18.2–21.2(−22.1) × (5.6–)6.2–7.0(−7.4) µm	Q = (2.5–)2.7–3.3(−3.4), *N* = 60	Me = 19.8 × 6.6 µm, Qe = 3.0	3.9–5.1 × 3.1–3.9 µm, Me = 4.5 × 3.5 µm
*GYJF19012*	(15.8–)16.9–19.8(−21.4) × (5.8–)6.4–7.5(−7.9) µm	Q = (2.1–)2.3–3.0(−3.3), *N* = 60	Me = 18.1 × 7.0 µm, Qe = 2.6	4.2–4.8 × 3.0–3.9 µm, Me = 4.5 × 3.3 µm
*GYJF21075*	(15.7–)17.3–20.4(−23.5) × (5.6–)5.8–6.6(−6.8) µm	Q = (2.5–)2.7–3.4(−3.7), *N* = 60	Me = 18.7 × 6.2 µm, Qe = 3.0	
*GYJF21122*	(14.5–)15.4–17.8(−18.7) × (5.7–)5.9–6.8(−7.4) µm	Q = (2.2–)2.4–2.9(−3.1), *N* = 60	Me = 16.7 × 6.4 µm, Qe = 2.6	
*GYJF21324*	(16.3–)16.9–19.1(−19.6) × (5.5–)6.0–6.9(−7.5) µm	Q = (2.4–)2.6–3.0(−3.3), *N* = 60	Me = 17.9 × 6.4 µm, Qe = 2.8	4.5–5.3 × 3.1–3.7 µm, Me = 4.8 × 3.4 µm
**Cumulated values**	**(14.5–)15.4–21.2(−23.5) × (4.9–)5.4–7.5(−7.9) µm**	**Q = (2.1–)2.3–3.4(−3.8), N = 600**	**Me = 18.3 × 6.5 µm, Qe = 2.8**	**3.9–5.4 × 3.0–4.0 µm, Me = 4.6 × 3.4 µm, N = 200**
Leprieur 1202 (type) FG (Joly 1968)	16–22 × 6–8 µm		Me = 19–19.5 × 6.5–7 µm	
Venezuela (Rogers et al. 1988)	(15–)16–21 × 6–7.5 µm		Me = 18.5 × 6.8 µm, Qe = 2.7	4.5 × 3 µm
FG (Callan and Rogers 1990)	17–20(−20.5) × 6–7 µm		Me = 18.5 × 6.5 µm, Qe = 2.8	7 × 4 µm
*X. telfairii* FWI (Fournier et al. 2019)	(16.2–)16.9–22.8(−24.3) × (5.7–)6.0–8.6(−9.1) µm	Q = (2.0–)2.2–3.6(−3.8), *N* = 300	Me = 19.6 × 6.9 µm, Qe = 2.8	3.5–6.0 × 2.8–4.1 µm, Me = 4.7 × 3.5 µm, *N* = 100

≡ *Hypoxylon enterogenum* Mont., Ann. Sci. Nat. Bot., sér. II, 13: 342. 1840.

**Stromata** upright, scattered to gregarious, separate, occasionally connate by two or three at base, narrowly to broadly clavate or fusoid, with obtusely to narrowly rounded fertile apex, straight to slightly curved, unbranched, 11–46 mm in total height, stipitate; fertile head 5–40 mm high × 2.5–9 mm wide, shriveling upon drying and frequently more or less split lengthwise with inrolled margins, occasionally flattened; surface color ranging from whitish to yellowish gray or brownish gray, often mixed in the same colony, without exposed perithecial contours; outermost coating bipartite, comprising a thin, superficial, colored pellicle releasing amber (47) pigments in 10% KOH and fleeting, faintly yellowish pigments in NH3, and a conspicuous underlying pure white granular layer 40–130 µm thick, frequently showing through the outermost pellicle; subsurface a black carbonaceous crust 70–100 µm thick, up to 130 µm thick at apex; interior solid, pithy to fibrous, brownish gray to tan, white around perithecia, rarely becoming hollow, not turning gelatinous or filled with liquid; the stipes usually well-defined, 3–17 mm high, 1.2–2.5 mm wide, smooth, glabrous, first concolorous with the fertile head and gradually blackening from the base which is slightly swollen. **Perithecia** subglobose to depressed-spherical, 0.4–0.6 mm diam, laterally flattened when in contact. **Ostioles** inconspicuous, discoid to faintly convex, black, minutely porate, 40–70 µm diam, opening flush with the surface or barely protruding, often masked by ascospores deposits.

**Paraphyses** copious, hyphal, thin-walled, 4.0–5.5 µm wide at base, tapering to 1.2–1.5 µm wide above asci, sparsely guttulate, discreetly embedded in mucilaginous material. **Asci** cylindrical, with (4–6–)8 slightly overlapping uniseriately arranged ascospores, the spore-bearing parts 110–125 × 9.0–11.0 µm, the stipes 62–70 µm long, with apical apparatus slightly urn-shaped, apically flattened with an obtuse lateral rim, basally attenuated, 3.9–5.4 × 3.0–4.0 µm (Me = 4.6 × 3.4 µm, *N* = 200), strongly bluing in Melzer’s reagent. **Ascospores** (14.5–)15.4–21.2(−23.5) × (4.9–)5.4–7.5(−7.9) µm, Q = (2.1–)2.3–3.4(−3.8), *N* = 600 (Me = 18.3 × 6.5 µm, Qe = 2.8), ellipsoid-inequilateral to navicular with narrowly to broadly rounded, at times slightly pinched ends, slightly ventrally concave, dark brown, unicellular, with a narrow, obliquely oriented, straight, rarely slightly sigmoid germ slit 6–9 µm long on the ventral side; lacking appendages or mucilaginous sheath; epispore smooth.

**Asexual morph** on the natural substrate not observed. Culture characteristics described by Callan and Rogers (1990).

**Specimens examined: FRENCH GUIANA.** Régina, Nouragues natural reserve, Inselberg camp, 4.311240 N, 52.132789 W, ca. 300 m, hygrophilic rainforest, on dead corticated branch, 18 Jun 2012, *Fournier, J. GYJF12051* (HAST 147299); Maripasoula, Saül, shortcut to the airfield, 3.623524 N, 53.206542 W, 210–220 m, disturbed secondary rainforest, on dead blackened wood, 17 Jun 2019, *Fournier, J. GYJF19012* (HAST 147300); Kourou, Sentier de la Montagne des Singes, 5.068808 N, 52.69478 W, lowland rainforest, on dead corticated branch, 27 Apr 2008, *Lechat, C. CLL8002* (HAST 147301); Maripasoula, Saül, trail head to Roche-Bateau, Crique-Cochon, 3.625994 N, 53.1955 W, 210–220 m, on dead corticated branch, 31 Mar 2021, *Fournier, J. GYJF21324* (HAST 147302); Maripasoula, Saül, trail head to Sentier des Gros Arbres, 3.620839 N, 53.208536 W, ca. 210 m, disturbed secondary rainforest, on dead decorticated wood, 26 Mar 2021, *Fournier, J. GYJF21122* (HAST 147303); Régina, Nouragues natural reserve, Inselberg camp, 4.311240 N, 52.132789 W, ca. 300 m, hygrophilic rainforest, on dead corticated branch, 17 Jun 2012, *Fournier, J. GYJF12025* (HAST 147304); Régina, Nouragues natural reserve, Inselberg camp, 4.311240 N, 52.132789 W, ca. 300 m, hygrophilic rainforest, on dead corticated branch, 19 Jun 2012, *Fournier, J. GYJF12068* (HAST 147305); Roura: Cacao, Camp Bonaventure, 4.326815 N, 52.341431 W, 60–65 m disturbed hygrophilic rainforest, on dead trunk, 22 Mar 2021, *Fournier, J. GYJF21075* (HAST 147306); Sinnamary, Paracou, CIRAD field centre, Guyaflux plot, 5.249218 N, 52.915431 W, lowland rainforest, on dead wood, 26 Feb 2007, *Lechat, C. CLL7043* (HAST 147307); Sinnamary, Paracou, CIRAD field centre, Guyaflux plot, 5.249218 N, 52.915431 W, lowland rainforest, on dead wood, 30 Apr 2008, *Lechat, C. CLL8031* (HAST 147308); Maripasoula, Saül, shortcut to the airfield, 3.622852 N, 53.205768 W, 210–220 m, disturbed secondary rainforest, on dead decorticated wood, 29 Mar 2021, *Fournier, J. GYJF21241 *(HAST 147309), immature.

**Known distribution**: Central and Amazonian South America (Dennis 1956).

**Comments**: *Xylaria enterogena* is common in the Guianese forests but its identification is often challenging due to a wide range of variations in stromatal shape and color, sometimes even within a single collection. It can be distinguished from similar species by the combination of small, short-stipitate, typically clavate stromata with obtusely rounded fertile apices, a grayish to yellowish even stromatal surface, inconspicuous ostioles, perithecia immersed beneath a thick carbonaceous crust, and a pure white granular layer. Its dark brown, navicular ascospores 15–21 × 5.5–7.5 µm feature narrowly to broadly rounded ends and a short, obliquely oriented germ slit.

Usually, *X*. *enterogena* is readily distinguished from *X. telfairii* (Berk.) Sacc., which features stouter, more densely pigmented stromata and a pantropical distribution. However, confusion may arise between stromata from luxuriant collections of *X. enterogena* and relatively small stromata of *X. telfairii*, since both species frequently occur in the same habitats. Ascospore dimensions overlap considerably and thus do not provide a reliable diagnostic character. In the field, the absence of internal gelatinous tissue associated with the presence of a hyaline liquid content in fresh stromata sets *X. enterogena* apart from *X. telfairii* (Fournier et al. 2019). The distinctiveness of *X. enterogena* relative to *X. telfairii* is also supported by our phylogenetic analyses.

**Fig. 21:**
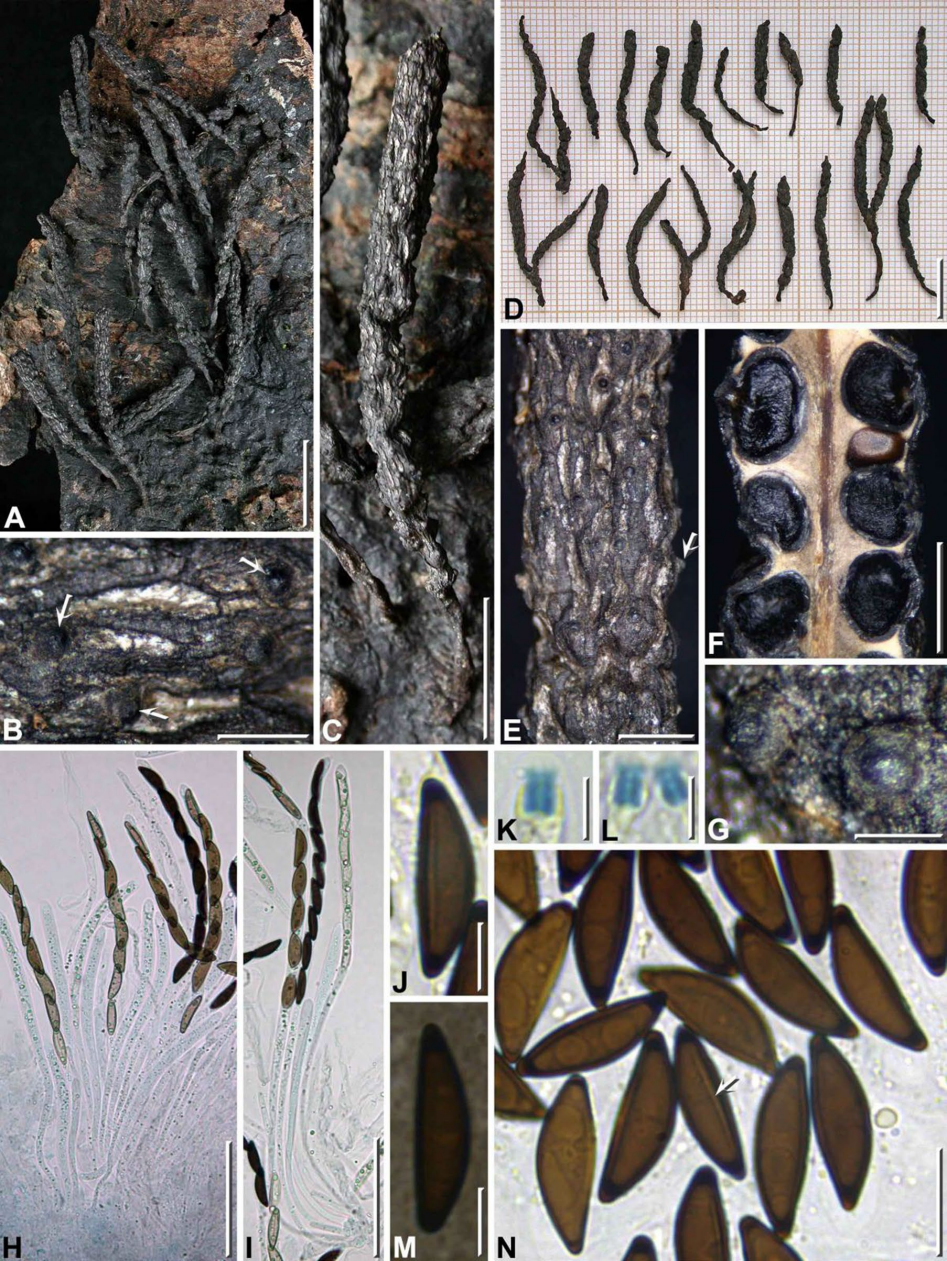
*Xylaria exechostoma* (**A–N ***GYJF21251* holotype). **A** Habit of prostrate stromata on blackened bark. **B** Stromatal surface in close-up showing silvery gray superficial stripes lacking granular coating and prominent black ostioles (arrows). **C** Individual stroma with a short well-defined stipe. **D** Variously shaped stromata. **E** Stromatal surface roughened by silvery gray stripes and ostioles (one in side view indicated by an arrow). **F** Stroma in longitudinal section. **G** Two adjacent ostioles with blackish gray fibrous base. **H, I** Immature and mature long-stipitate asci, in chlorazol black. **J** Ascospore in latero-ventral view showing a thin germ slit less than spore-length and more pigmented ends, in 1% SDS. **K, L** Ascal apical apparati in Melzer’s reagent. **M** Ascospore in India ink showing a lack of mucilaginous sheath or appendage. **N** Ascospores in 1% SDS with more pigmented ends, one showing a germ slit (arrow). Bars in **A, D** = 10 mm; **B** = 0.5 mm; **C** = 5 mm; **E, F** = 1 mm; **G** = 200 µm; **H, I** = 50 µm; **J–M** = 5 µm; **N** = 10 µm

***Xylaria exechostoma*** J. Fourn., Y.-M. Ju, H.-M. Hsieh & Lechat, sp. nov. Figure 21

MycoBank MB863162

**Typification**: **FRENCH GUIANA.** Maripasoula, Saül, shortcut toward the airfield, 3.622852 N, 53.205768 W, 210–220 m, disturbed secondary rainforest, on dead corticated trunk, 29 Mar 2021, *Fournier, J. GYJF21251* (holotype HAST 147310).

**Etymology**: From Greek εξέχος (exechos) = prominent and *στόμα* (stoma) = mouth, opening, for the prominent and conspicuous ostioles.

**Diagnosis**: Differs from *X. atractoidosperma*, the most resembling species, by its less deeply constricted stromata with a silvery-gray outer layer lacking carbonaceous granules, larger and more prominent ostioles 170–210 µm diam, and slightly shorter ascospores averaging 16.0 × 4.5 µm vs 18.1 × 4.4 µm, with a higher length/width ratio = 4.1 vs 3.6.

**Stromata** (11–)15–34 mm in total height, gregarious, upright to prostrate, simple to rarely branched or connate at base by two, the fertile parts 8–22 mm high × 1.4–2.2(−2.6) mm wide, narrowly cylindrical with faint scattered circumferential constrictions, straight to slightly curved, with mucronate to apiculate sterile apices; surface with slightly exposed perithecial contours, roughened by a thick, silvery gray outer layer splitting into narrow to wide longitudinal stripes faintly incrusted in places with small black granules, exposing a dark gray to blackish superficial layer lacking black granules; underlying crust 70–80 µm thick, black, slightly carbonaceous; interior solid, whitish to brownish, fibrous, with an orange-brown core; the stipes mostly well-defined, 1.6–13 mm long × 0.4–0.85 mm wide, black, irregularly flattened, glabrous, smooth to finely roughened, the base slightly enlarged. **Perithecia** immersed to slightly exposed, subglobose 0.50–0.60 mm diam to depressed-spherical up to 0.65 mm high × 1 mm diam. **Ostioles** conspicuous, obtusely conical, prominent, black, on a blackish gray, somewhat fibrous base, 120–170 µm high × 170–210 µm diam.

**Paraphyses** hyphal, thin-walled, 4–6 µm wide at base, tapering to 1–1.5 µm wide above asci, sparsely and minutely guttulate, embedded in mucilaginous material. **Asci** cylindrical, with (6–)8 slightly overlapping uniseriately arranged ascospores, the spore-bearing parts 95–105 × 6.0–7.5 µm, the stipes 95–110 µm long, fragile, with apical apparatus 2.5–3.1 × 1.6–2.1 µm (Me = 2.9 × 1.9 µm, *N* = 25), tubular with a faint upper rim, bluing in Melzer’s reagent. **Ascospores** (14.1–)14.9–16.5(−17.6) × (4.1–)4.3–5.1(−5.4) µm, Q = (2.8–)3.1–3.7(−3.9), *N* = 60 (Me = 15.8 × 4.7 µm, Qe = 3.4), narrowly fusiform-inequilateral with darker, narrowly rounded, frequently slightly pinched ends, olivaceous brown to dark brown, unicellular, with a fairly inconspicuous, straight, longitudinally oriented germ slit 1/2–2/3 spore-length on the ventral side, without sheath or appendages; epispore smooth.

**Asexual morph** on the natural substrate not observed.

**Known distribution**: French Guiana, known only from the type collection.

**Comments**: *Xylaria exechostoma* is strikingly similar to *X. atractoidosperma* in both outward morphology and microscopic features. They can only de distinguished through a close examination of the stromatal surface. *Xylaria exechostoma* differs by more exposed perithecial contours, giving a more nodulose appearance and less deep circumferential constrictions. Its outer layer is split into silvery-gray stripes that lack the dense granular coating of *X. atractoidosperma*. In addition, while the ostioles of *X*. *atractoidosperma* are inconspicuous those of *X*. *exechostoma* are strongly prominent, most often approaching 200 µm wide at base, a readily observed differential character by dissecting microscope. Moreover, the ascospores of *X*. *exechostoma* are slightly shorter than those of *X. atractoidosperma*, 16.0 × 4.5 µm on average vs 18.1 × 4.4 µm, with a lower length/width ratio = 3.6 vs 4.1, with their germ slit shorter and their ends more strongly pigmented at maturity.

**Fig. 22:**
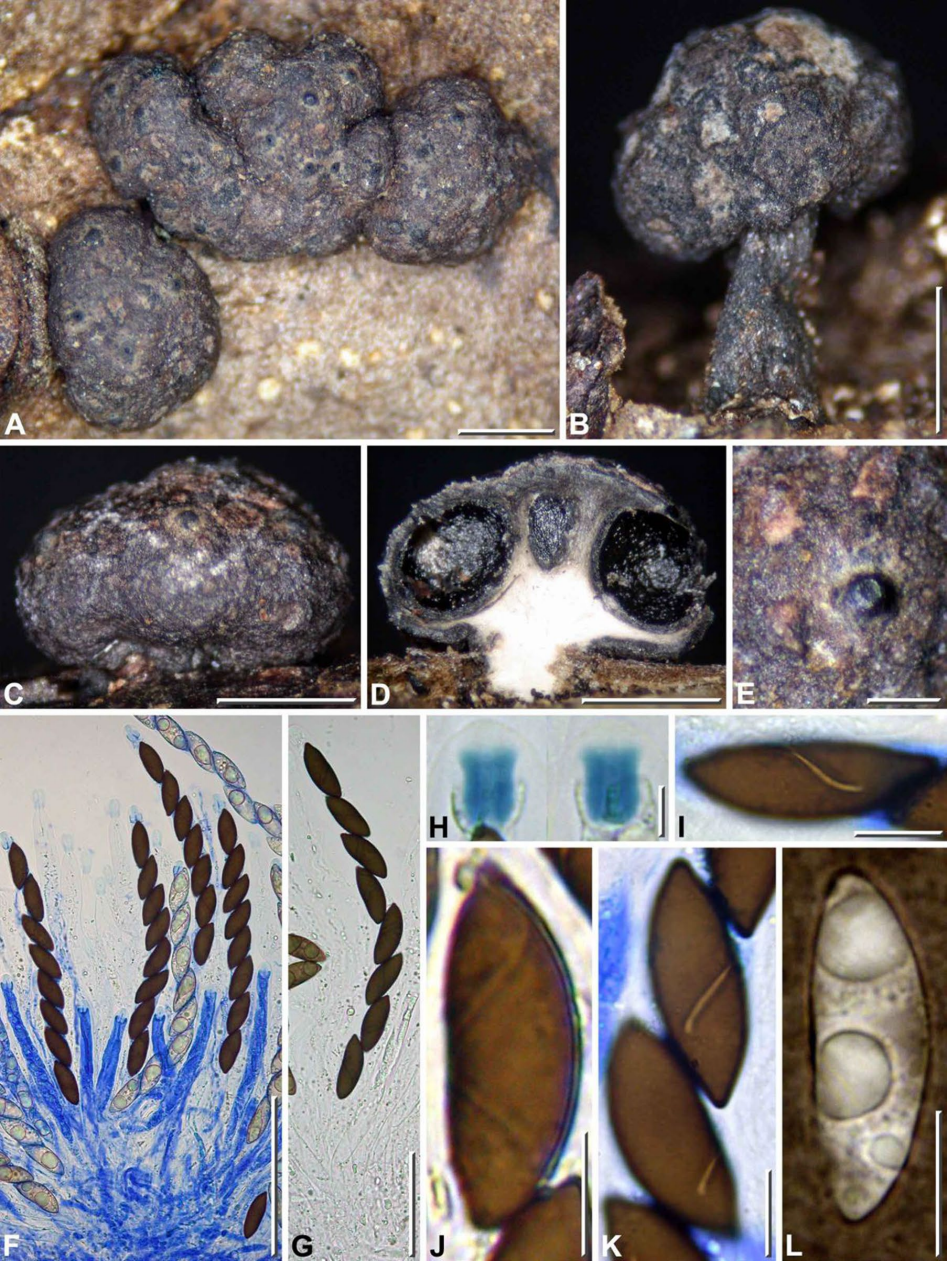
*Xylaria fallax* (**A**–**L** *GYJF21161*). **A** Coalescent pulvinate stromata. **B** Stipitate stroma. **C** Subsessile pulvinate stroma in side view showing orange-brown superficial scales on upper part. **D** Same stroma as in **C** in vertical section showing a stout central connective erumpent through bark. **E** Ostiole in close-up surrounded by superficial orange-brown scales. **F** Short-stipitate immature and mature asci, in blue Pelikan ink. **G** Mature ascus, in 1% SDS. **H** Ascal apical apparati, in Melzer’s reagent. **I, K** Ascospores in side view showing a spiraling germ slit not reaching the ends, in blue Pelikan ink. **J** Ascospore in side view with intermediate focus showing germ slit on both sides of the ascospore with inflexion point on the ventral side, in 1% SDS. **L** Immature ascospore in India ink showing an absence of sheath or appendages. Bars in **A–D** = 1 mm; **E** = 0.2 mm; **F** = 100 µm; **G** = 50 µm; **H** = 2 µm; **I–L** = 10 µm

***Xylaria fallax*** J. Fourn., Y.-M. Ju, H.-M. Hsieh & Lechat, sp. nov. Figure 22

MycoBank MB863163

**Typification**: **FRENCH GUIANA.** Maripasoula, Saül, trail head of Sentier des Gros Arbres, 3.6201 N, 53.207989 W, ca. 210 m, disturbed secondary rainforest, on dead corticated branchlet mixed with *X. meliacearum*, 27 Mar 2021, *Fournier, J. GYJF21161* (holotype HAST 147311).

**Etymology**: From Latin *fallax =* misleading, deceptive, for its confusing occurrence alongside penzigioid stromata of *X. meliacearum* on the same substrate.

**Diagnosis**: Differs from *X. meliacearum* by darker brown, larger ascospores 27.2–30.1 × 10.3–11.4 µm vs 22.8–26.0 × 6.0–7.5 µm, which feature subacute and slightly pinched ends.

**Stromata** pulvinate, orbicular, convex, few-peritheciate, separate to coalescent, sessile to subsessile, 1.5–2.3 mm diam, 0.8–1.0 mm thick, occasionally on a short stipe up to 1.4 mm × 0.5–0.6 mm wide, with faintly exposed to unexposed perithecial contours; surface blackish brown, slightly uneven, roughened by sparse brownish gray to light orange-brown patches or scales; subsurface a slightly carbonaceous, brittle crust 80 µm thick on the sides, to 120 µm thick on top; interior white, gray just beneath the crust, solid, pithy. **Perithecia** subglobose, 0.75–0.85 mm diam. **Ostioles** papillate, broadly conical, porate, black, 100–125 µm diam at base, frequently surrounded by an irregular halo of light brownish gray superficial tissue.

**Paraphyses** hyphal, hyaline, remotely septate, simple, 8.0–9.7 µm wide at base, tapering to 1.5–2.0 µm wide, discreetly embedded in mucilaginous material. **Asci** cylindrical, with (6–)8 uniseriately arranged, slightly overlapping ascospores, the spore-bearing parts (160–)175–200 µm long × 17.0–19.5 µm wide, the stipes 80–95 µm long, with apical apparatus broadly cylindrical, apically flared with an obtuse rim and basally obtusely rounded, 6.4–7.6 × 3.7–4.4 µm (Me = 6.9 × 4.0 µm, *N* = 25), bluing in Melzer’s reagent. **Ascospores** (26.1–)27.2–30.1(−31.2) × (9.8–)10.3–11.4(−12.4) µm, Q = (2.3–) 2.4–2.8 (−3.0), *N* = 60 (Me = 28.7 × 10.9 µm, Qe = 2.6), ellipsoid slightly inequilateral with narrowly rounded to subacute, frequently slightly pinched ends, straight, dark brown, with a short strongly spiraling germ slit extending on the ventral side over ca 60–75% of the circumference, without sheath or appendage visible in India ink; epispore smooth.

**Asexual morph** on the natural substrate not observed.

**Known distribution**: French Guiana, known only from the holotype collection.

**Comments**: The stromata of *X. fallax* are characterized by being few-peritheciate, pulvinate, blackish brown, sessile but occasionally shortly stipitate, with a slightly carbonaceous, brittle crust. Its ascospores are distinctive, 27.2–30.1 × 10.3–11.4 µm, dark brown, with subacute, slightly pinched ends and a ventral spiraling germ slit less than spore-length. This combination of morphological features distinguishes *X*. *fallax* from similar penzigioid *Xylaria* species.

The first confusion arose with the penzigioid stromata of *X. meliacearum* (this paper), with which *X*. *fallax* was intermixed on the same branchlet and shares a similar ventral spiraling ascospore germ slit. Besides subtle stromatal differences, ascospore dimensions, color, and morphology clearly distinguish the two species. Unfortunately, sequencing data are not available for *X*. *fallax*.

Known small penzigioid species of *Xylaria* with a spiraling germ slit are few and differ unambiguously from *X. fallax* by morphological characters. *Xylaria carabayensis* (Mont.) Y.-M. Ju, H.-M. Hsieh & J. D. Rogers was redescribed by Ju et al. (2012), who recorded much broader, equilateral ascospores, 27.5–35 × 15–19.5 µm, with a spore-length germ slit, and noted the presence of a conidial peg on the top of stromata.

*Xylaria boergesenii* (Ferd. & Winge) P. F. Cannon differs by smaller, suballantoid ascospores, 18.3–23.9 × 6.4–7.9 µm, with broadly rounded ends and a longer germ slit, and *X. entomelaina* J. Fourn. & Lechat differs by uniperitheciate stromata with a blackish interior and smaller, relatively broader ascospores, 22.7–26.2 × 9.8–11.2 µm (Fournier et al. 2018c). The final species to consider is *X. parvula* J. Fourn. & Lechat, which differs by minute uniperitheciate stromata with a thick leathery crust 80–120 µm thick and much smaller, subreniform ascospores 14.7–17.1 × 6.5–7.6 µm (Fournier et al. 2018c).

***Xylaria flabelliformis*** (Schwein.) Fr., Summa Veg. Scand. II, p. 381. 1849.

The cited collection from French Guiana closely matches those reported and illustrated from the French West Indies (Fournier et al. 2019).

**Specimen examined: FRENCH GUIANA.** Maripasoula, Saül, trail head to Sentier Roche-Bateau, Crique-Cochon, 3.625994 N, 53.1955 W, 210–220 m, disturbed secondary rainforest, on dead wood, 31 Mar 2021, *Fournier, J. GYJF21318* (HAST 147312).

**Fig. 23:**
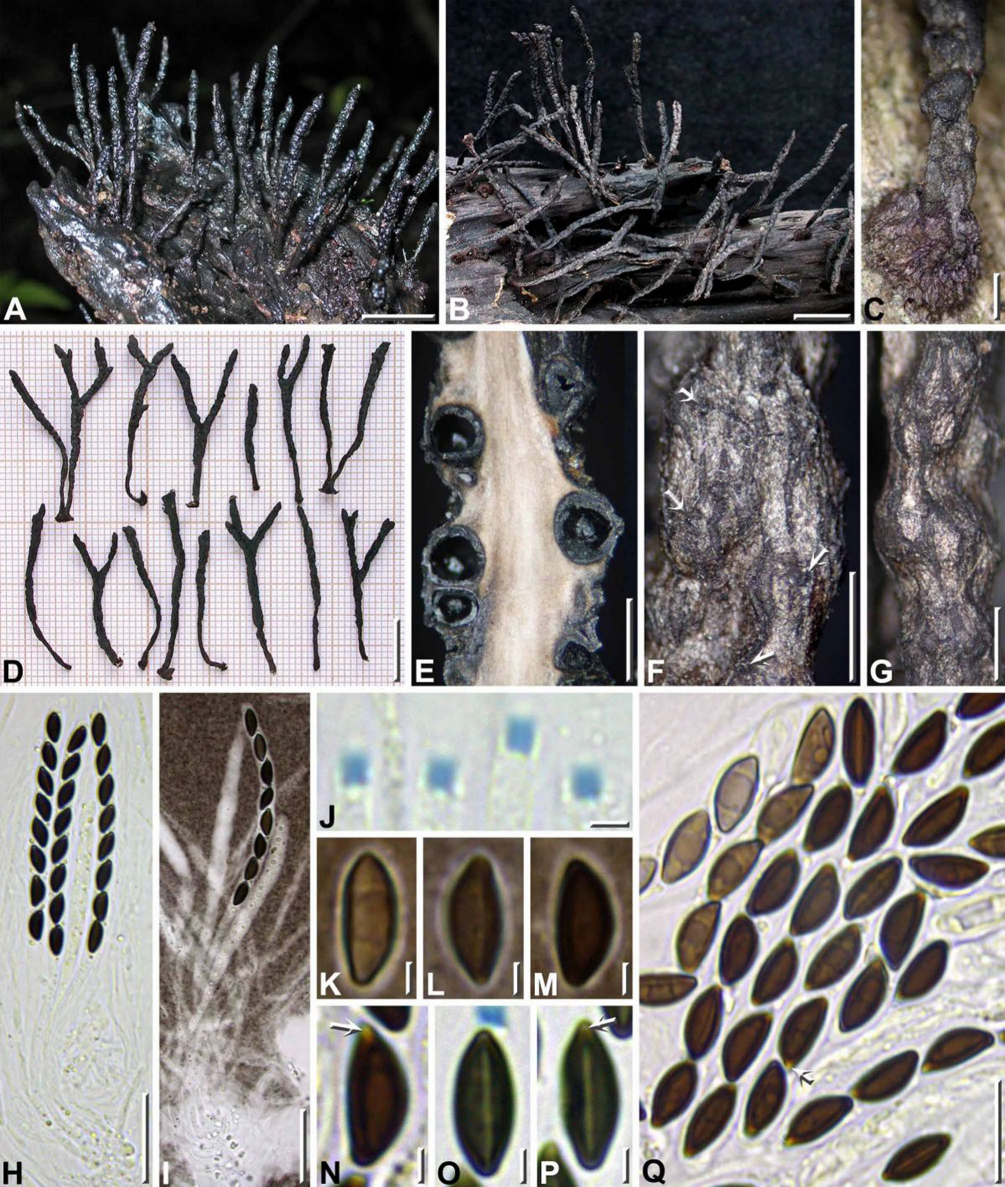
*Xylaria gigartosperma* (**A, F, G, J–Q ***GYJF21418* holotype; **B, D, H ***GYJF18075*; **C ***GYJF21423*; **E, I ***GYJF18056*). **A** Stromata in the fresh state, in situ. **B** Stromata in the dry state. **C** Pannose sleeve at base of a stipe. **D** Variously branched stromata. **E** Stroma in longitudinal section showing some partly exposed perithecia and a grayish white interior. **F** Stromatal surface showing silvery gray superficial stripes and ostioles (arrows). **G** Nodulose stromatal surface. **H** Mature asci in 1% SDS. **I** Immature and mature asci, in India ink. **J** Ascal apical apparati, in Melzer’s reagent. **K–M** Ascospores at three different states of maturity showing a thin gelatinous sheath, in India ink. **N** Ascospore showing pinched ends, one subporate (arrow), in 1% SDS. **O, P** Ascospores in ventral view showing a germ slit, in Melzer’s reagent. **Q** Variously oriented ascospores, some showing a subporate end (arrow) or a germ slit, in 1% SDS. Bars in **A, B, D** = 10 mm; **C** = 1 mm; **E–G** = 0.5 mm; **H, I** = 20 µm; **J–P** = 2 µm; **Q** = 10 µm

***Xylaria gigartosperma*** J. Fourn., Y.-M. Ju, H.-M. Hsieh & Lechat, sp. nov. Figure 23, Table 14

**Table 14:** Ascospore dimensions in five collections of *X. gigartosperma* showing the range of intraspecific variations

Collection numbers	Ascospore measurements with extreme values in parentheses	Q = quotient l/w, *N* = number of measurements	Me = mean values of l/w, Qe = mean quotient l/w
*GYJF18056*	(6.1–)6.5–7.5(−7.7) × (2.8–)3.0–3.3(−3.5) µm	Q = (1.9–)2.0–2.4(−2.6), *N* = 60	Me = 7.0 × 3.2 µm, Qe = 2.2
*GYJF18075*	(6.2–)6.5–7.4(−7.9) × (2.8–)3.0–3.4(−3.6) µm	Q = (1.9–)2.0–2.3(−2.5), *N* = 60	Me = 7.0 × 3.2 µm, Qe = 2.2
*GYJF21334*	(6.7–)7.0–7.9(−8.4) × (3.1–)3.2–3.5(−3.6) µm	Q = (1.9–)2.1–2.4(−2.5), *N* = 60	Me = 7.4 × 3.3 µm, Qe = 2.2
*GYJF21418*	(6.5–)6.8–7.7(−8.1) × (2.9–)3.0–3.5(−3.7) µm	Q = (1.9–)2.0–2.5(−2.7), *N* = 60	Me = 7.2 × 3.2 µm, Qe = 2.2
*GYJF21434*	(6.9–)7.3–8.2(−8.5) × (3.0–)3.2–3.6(−3.8) µm	Q = (2.1–)2.2–2.4(−2.6), *N* = 60	Me = 7.8 × 3.4 µm, Qe = 2.3
**Cumulated values**	**(6.1–)6.5–8.2(−8.5) × (2.8–)3.0–3.6(−3.8) µm**	**Q = (1.9–)2.0–2.5(−2.7), N = 240**	**Me = 7.3 × 3.3 µm, Qe = 2.2**

MycoBank MB863165

**Typification**: **FRENCH GUIANA.** Maripasoula, Saül, Sentier des Monts-La-Fumée, tributary of Crique Queue-Hocco, 3.637173 N, 53.204727 W, disturbed secondary rainforest, ca. 260 m, on blackened wood, 4 Apr 2021, *Fournier, J. GYJF21418* (holotype HAST 147319).

**Etymology**: From Greek *γίγαρτον* (gigarton) = grape seed, for the ascospore shape.

**Diagnosis**: Differs from morphologically similar species of *Xylaria,* particularly *X. multiplex,* by small, fusiform-inequilateral ascospores with narrowly rounded, slightly pinched ends, averaging 7.3 × 3.3 µm.

**Stromata** scattered to densely gregarious forming bush-like colonies, upright to prostrate, simple to frequently furcate or connate at base by two, straight to slightly bent, 10–45 mm in total height, the fertile head 5–35 mm high × 0.8–2.1 mm diam, cylindrical to slightly flattened, occasionally spathulate at apex, with narrowly rounded, often broken sterile apex; the stipes 2.5–15 mm high, 0.5–1.8 mm wide, most often ill-defined, cylindrical to flattened, black, puckered, glabrous to finely tomentose, the base slightly enlarged and embedded in a purplish brown, pannose, discoid to conical sleeve ca. 2.5 mm diam, remaining adherent to the substrate after the stroma has fallen off. Surface dull black, strongly nodulose with circumferential wrinkles, longitudinal furrows and perithecial contours variously exposed, with a gradually worn off silvery gray to blackish gray outer layer longitudinally split into narrow stripes; subsurface crust leathery, ca. 40 µm thick; interior white with grayish strands in places, solid, pithy. **Perithecia** subglobose to depressed-spherical or laterally flattened, 0.35–0.50 mm diam. **Ostioles** black, obtusely papillate and inconspicuous to raised-discoid (80–)100–130 µm diam at base.

**Paraphyses** hyphal, hyaline, remotely septate, simple to branched at base, 4.0–5.0 µm wide at base, tapering to 1.0–1.5 µm wide, discreetly embedded in mucilaginous material. **Asci** cylindrical, with (6–)8 uniseriately arranged, slightly overlapping ascospores, the spore-bearing parts 52–60 µm long × 4.5–5.2 µm wide, the stipes 50–58 µm long, with apical apparatus short tubular to subquadrate in outline, apically slightly flared with a faint upper rim, 1.4–1.8 × 1.1–1.4 µm (Me = 1.6 × 1.2 µm, *N* = 40), bluing in Melzer’s reagent. **Ascospores** (6.1–)6.5–8.2(−8.5) × (2.8–)3.0–3.6(−3.8) µm, Q = (1.9–)2.0–2.5(−2.7), *N* = 240 (Me = 7.3 × 3.3 µm, Qe = 2.2), fusiform-inequilateral with narrowly rounded to subacute, slightly pinched ends, with a less pigmented, conspicuous subporate area at one (apical) or both ends, dark brown, with a straight germ slit slightly less than spore-length on the ventral side, with a narrow, continuous, persistent mucilaginous sheath visible in India ink; epispore smooth.

**Asexual morph** on the natural substrate not observed.

**Other specimens examined (paratypes)**: **FRENCH GUIANA.** Maripasoula, Saül, trail to Monts-La-Fumée, tributary to Crique Queue-Hocco, 3.637173 N, 53.204727 W, ca. 260 m, disturbed mesophilic rainforest, on dead corticated branch, 4 Apr 2021, *Fournier, J. GYJF21423* (HAST 147313); Maripasoula, Saül, trail head of Sentier des Gros Arbres, Crique Grand-Fossé, 3.617198 N, 53.209029 W, ca. 210 m, on dead blackened wood, 1 Apr 2021, *Fournier, J. GYJF21434* (HAST 147314); Maripasoula, Saül, trail head toward Monts-La-Fumée, 3.629382 N, 53.206831 W, on dead corticated branch and on decorticated wood, 21 Aug 2018, *Fournier, J. GYJF18056* (HAST 147315); Maripasoula, Saül, trail head of Sentier des Gros Arbres, Crique Grand-Fossé, 3.617198 N, 53.209029 W, ca. 210 m, on dead blackened wood, 1 Apr 2021, *Fournier, J. GYJF21334* (HAST 147316); Maripasoula, Saül, trail head of Sentier des Gros Arbres, 3.620589 N, 53.208547 W, on blackened dead wood, 22 Aug 2018, *Gardiennet, A. GYJF18075* (HAST 147317); Maripasoula, Saül, trail to Monts-La-Fumée, tributary to Crique Queue-Hocco, 3.637173 N, 53.204727 W, ca. 260 m, disturbed mesophilic rainforest, on blackened dead wood, 4 Apr 2021, *Fournier, J. GYJF21414* (HAST 147318).

**Known distribution**: French Guiana, vicinity of Saül.

**Comments**: The narrowly cylindrical, upright to prostrate, nodulose, frequently branched stromata of *X. gigartosperma*, often forming dense colonies, can be easily confused with those of the widespread and variable *X. multiplex*. However, the stromata of *X. gigartosperma* differ by being less acutely pointed at the apex, less carbonaceous, and more nodulose with a whitish interior. The main distinguishing feature displayed by *X. gigartosperma* is its unusual ascospore morphology characterized by being fusiform-inequilateral, with narrowly rounded to subacute, frequently pinched ends. Together with its small ascospore dimensions, this feature separate *X*. *gigartosperma* from *X. multiplex*, where ascospores average 10.5 × 3.4 µm (Fournier et al. 2020), and from allied species that could be confused with it. One such species is the North temperate *X. hypoxylon* (L.) Grev., a broadly applied name under which previous collections of *X. gigartosperma* may have been erroneously recorded.

A pore-like structure at the ends of ascospores is noted here because it is particularly conspicuous. However, subporate ascospores are encountered in many species of *Xylaria* and *Hypoxylon,* although the functional role of these structures remains unclear. Their role in germination is plausible, since germ tubes commonly originate from ascospores ends rather than from the longitudinal slit traditionally considered the primary germination site.

**Fig. 24:**
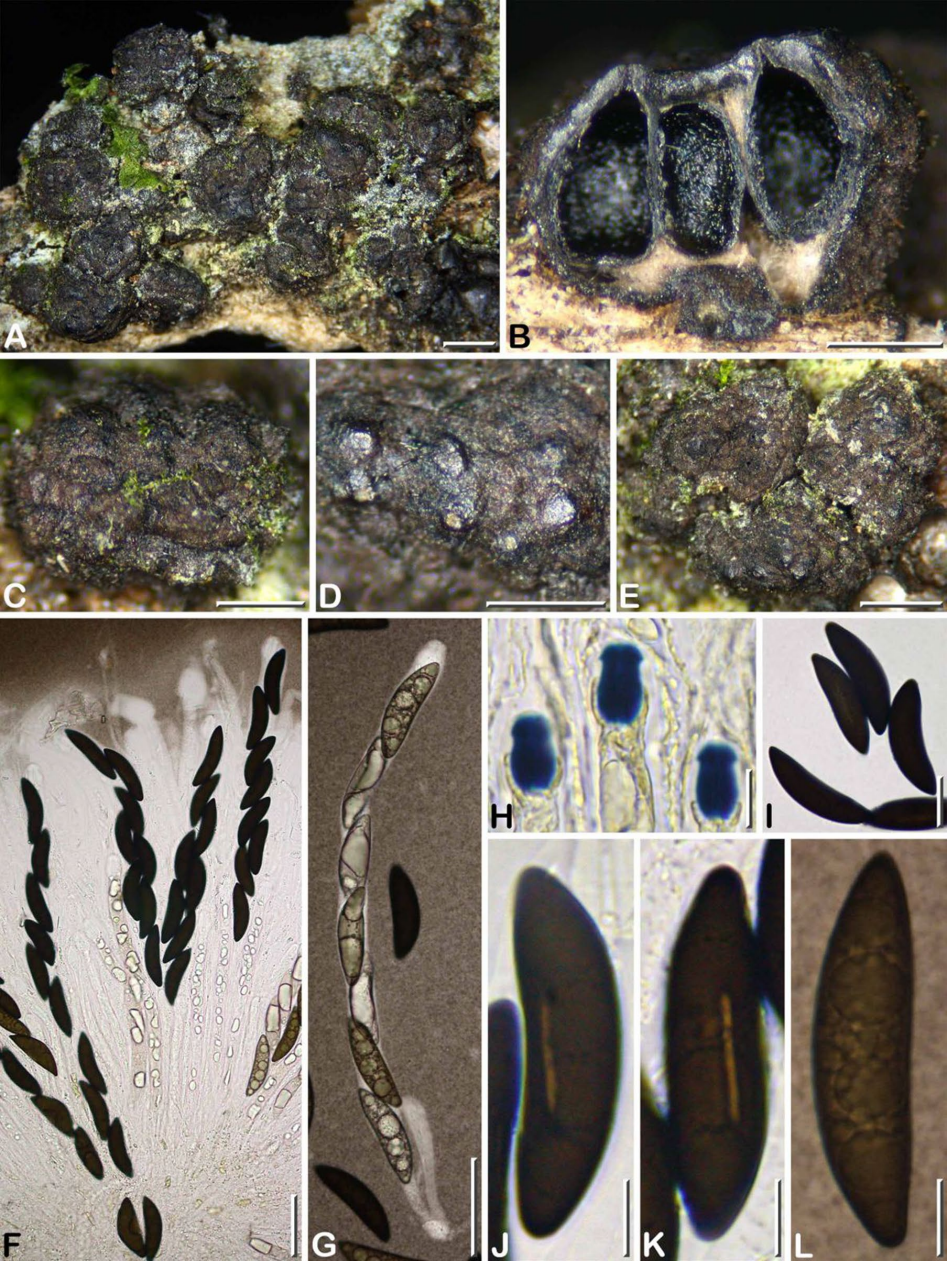
*Xylaria gigaspora* (*GYJF18099-1* holotype). **A** Colony of stromata on bark. **B** Subsessile stroma in vertical section showing perithecia immersed beneath a carbonaceous crust and a short narrow connective. **C** Stroma in top view showing a dark brown surface roughened by polygonal plaques. **D** Shiny black truncate ostioles in top view. **E** Three aggregated stromata in top view. **F** Mature asci. **G** Immature short-stipitate ascus (**F** and **G** in dilute India ink). **H** Ascal apical apparati in Melzer’s reagent. **I** Ascospores in side view. **J, K** Ascospores in ventral view showing a short germ slit (**I–K** in 1% SDS). **L** Ascospore in side view showing absence of appendages or sheath, in dilute India ink. Bars in **A, E** = 1 mm; **B–D** = 0.5 mm; **F, G** = 50 µm; **H, J–L** = 10 µm; **I** = 20 µm

***Xylaria gigaspora*** J. Fourn., Y.-M. Ju, H.-M. Hsieh & Lechat, sp. nov. Figure 24

MycoBank MB863166

**Typification**: **FRENCH GUIANA.** Maripasoula, Saül, trail head of Sentier Roche-Bateau, 3.619046 N, 53.200658 W, on dead corticated branch, associated with effete stromata of *X. nelumboniformis*, 23 Aug 2018, *Fournier, J. GYJF18099-1* (holotype HAST 147320).

**Etymology**: From Latin *Gigas, -antis =* the Giants, and *spora* = spore, for the huge ascospores characterizing this species.

**Diagnosis**: Differs from known penzigioid species of *Xylaria* with similar pulvinate, carbonaceous stromata by its small stromata barely exceeding 2 mm wide and huge, fusoid-inequilateral, blackish brown ascospores averaging 46.8 × 13.0 µm with a short germ slit.

**Stromata** gregarious, often closely aggregated, forming a small colony next to effete stromata of *X. nelumboniformis*, superficial, pulvinate, few-peritheciate, 1–2.2 mm in greatest dimension, 0.85–1.3 mm high, slightly convex on top, subsessile on a narrow, most often inconspicuous central connective. Surface dull brownish black, cracked and warty, obscured by greenish algae or lichens; subsurface crust carbonaceous, brittle, 80–100 µm thick at apex, thinner at sides; interior whitish, loosely fibrous, mostly visible at base. **Perithecia** ovoid to subglobose, 0.5–0.7 mm high × 0.4–0.7 mm diam. **Ostioles** faintly papillate-truncate, black to shiny black, becoming porate with age, most often inconspicuous.

**Paraphyses** copious, hyphal, remotely septate, thin-walled, 8–10 µm wide at base, tapering to 1.5–2.5 µm wide above asci, embedded in mucilaginous material. **Asci** cylindrical, short-stipitate, with (6–)8 overlapping uniseriately arranged ascospores, occasionally biseriately arranged, the spore-bearing parts 240–250 × 21–24(−29) µm, the stipes 32–85 µm long, with apical apparatus narrowly urn-shaped with a sharp apical rim, 12.3–14.5 × 6.7–8.2 µm (Me = 13.3 × 7.5, µm, *N* = 25), strongly bluing in Melzer’s reagent. **Ascospores** (42.0–)43.6–49.5(−53.5) × (10.6–)11.9–14.2(−14.9) µm, Q = (2.9–)3.1–4.0(−5.0), *N* = 60 (Me = 46.8 × 13.0 µm, Qe = 3.6), fusoid strongly inequilateral, straight to curved, ventrally flat to most often concave, with narrowly rounded to subacute ends, blackish brown, with a short, fairly conspicuous, longitudinal to slightly oblique straight germ slit 12–15 µm long on the ventral side. No sheath or appendages detected in India ink.

**Asexual morph** on the natural substrate not observed.

**Known distribution**: French Guiana, known only from the holotype collection.

**Comments**: *Xylaria gigaspora* is unequivocally characterized by the combination of minute, carbonaceous, penzigioid stromata rarely exceeding 2 mm wide and ascospores 43.6–49.5 × 11.9–14.2 µm, with a short, slightly oblique germ slit. It was incidentally spotted under the stereomicroscope as a small colony next to the stromata of another penzigioid *Xylaria* featuring stromata up to 8 mm wide. Its diminutive size renders it virtually undetectable in the field, which likely accounts for the absence of previous records. Its occurrence next to another *Xylaria* species does not appear to indicate a fungicolous lifestyle, and assessing the distribution of such an inconspicuous yet potentially widespread species will be a considerable challenge.

As to ascospore dimensions, the most resembling penzigioid species is *X. oxycephala* with ascospores 40.0–48.7 × 12.3–14.6 µm (this paper). *Xylaria oxycephala* differs by its subglobose, stipitate stromata topped by a pointed sterile extension and its ascospores featuring a spiraling germ slit.

***Xylaria globosa*** (Spreng.) Mont., Ann. Sci. Nat. Bot., sér. IV, 3: 103. 1855.

≡ *Sphaeria globosa* Spreng., K. Svenska Vetensk-Akad. Handl. ser. III, 8: 50. 1820; Spreng.: Fr., Syst. Mycol. II, p. 331. 1823.

≡ *Hypoxylon globosum* (Spreng.) Mont., Ann. Sci. Nat. Bot., sér. II, 13: 350. 1840.

The cited collection from French Guiana closely matches those reported and illustrated from the French West Indies (Fournier et al. 2018c).

**Specimen examined: FRENCH GUIANA.** Maripasoula, Saül, trail head to Sentier des Gros Arbres, 3.6201 N, 53.207989 W, ca. 210 m, disturbed secondary rainforest, on dead corticated branch, 27 Mar 2021, *Fournier, J. GYJF21193* (HAST 147321).

**Fig. 25:**
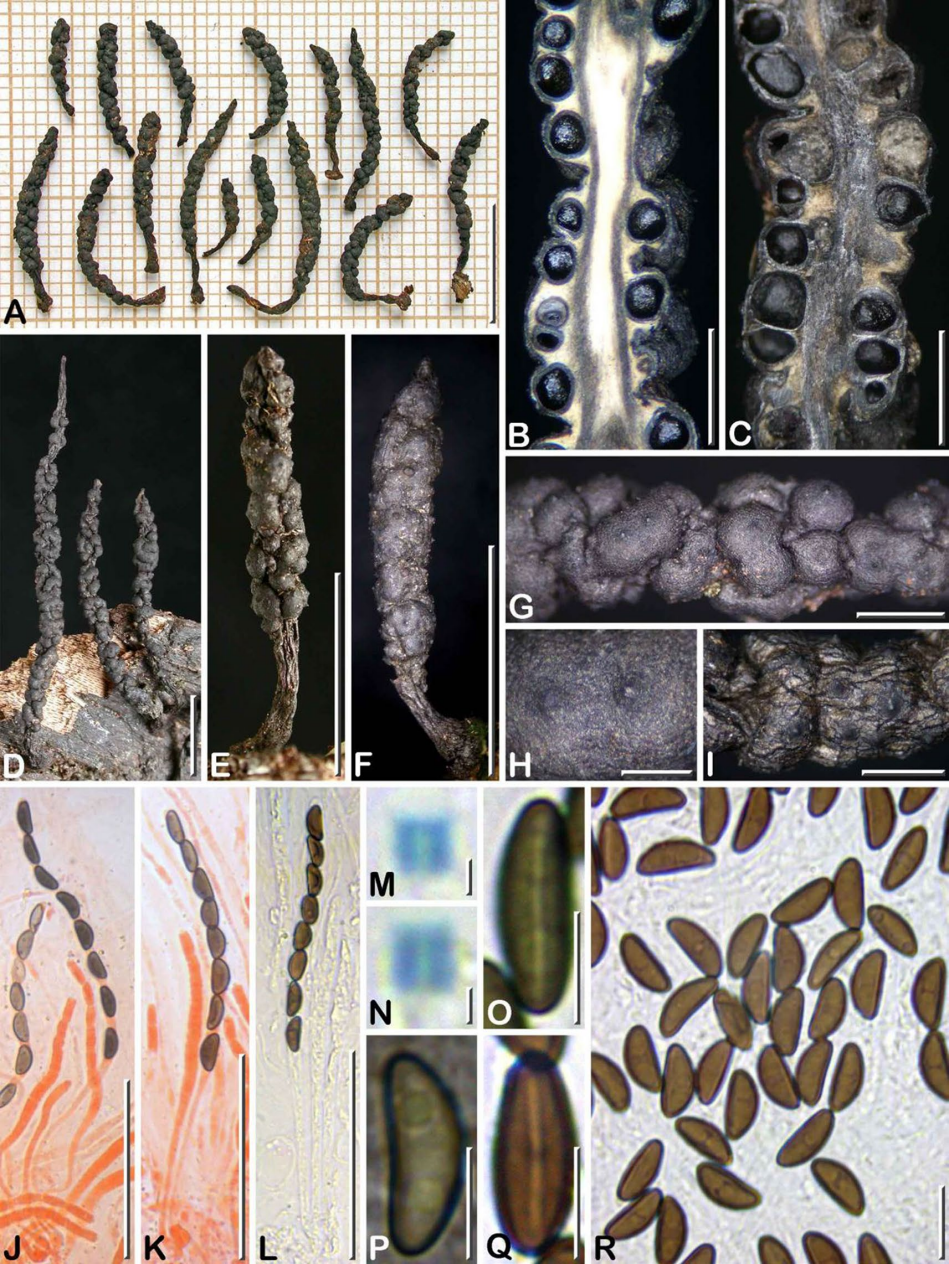
*Xylaria gracillima* (**A, E, G, H, J–M, Q ***GYJF21177*; **B, D, I, P ***GYJF19087*; **C, N, O, R ***GYJF21256*; **F ***GYJF21429*). **A** Various stromata from the same collection. **B** Young mature stroma in longitudinal section showing a reduced black core. **C** Slightly overmature stroma in longitudinal section showing a well-developed black core. **D, E** Nodulose stromata with variously developed apical extensions. **F** Stroma with weakly exposed perithecial contours. **G** Nodulose stroma with strongly exposed perithecial contours. **H** Stroma in close-up showing faintly papillate ostioles and a finely roughened surface. **I** Immature stroma in close-up showing papillate ostioles and a striped brownish gray peeling outer layer. **J, K** Asci in Congo red and 3% KOH. **L** Ascus in 1% SDS. **M, N** Ascal apical apparati in Melzer’s reagent. **O** Ascospore in the fresh state showing a ventral germ slit, in 1% SDS. **P** Ascospore in the fresh state in India ink, showing absence of mucilaginous sheath or appendage. **Q** Ascospore in the dry state showing a ventral germ slit, in 1% SDS. **R** Variously oriented ascospores in the dry state, in 1% SDS. Bars in **A** = 10 mm; **B**, **C**, **G**, **I** = 1 mm; **D–F** = 5 mm; **H** = 0.25 mm; **J–L** = 50 µm; **M, N** = 1 µm; **O–Q** = 5 µm; **R** = 10 µm

***Xylaria gracillima*** (Fr.) Fr., Nov. Act. Reg. Soc. Sci. Upsal., ser. III, 1: 128. 1851. Figure 25, Table 15

**Table 15:** Ascospore dimensions in seven collections from French Guiana compared with those referred to *X. gracillima* reported in literature from various origins

Collection numbers	Ascospore measurements with extreme values in parentheses	Q = quotient l/w, *N* = number of measurements	Me = mean values of l/w, Qe = mean quotient l/w
*GYJF19087*	(8.2–)8.7–10.2(−11.2) × (3.2–)3.5–4.1(−4.5) µm	Q = (2.2–)2.3–2.7(−3.2), *N* = 60	Me = 9.4 × 3.8 µm, Qe = 2.5
*GYJF21177*	(7.8–)8.4–9.5(−9.9) × (2.9–)3.1–3.6(−3.8) µm	Q = (2.3–)2.4–2.9(−3.1), *N* = 60	Me = 9.0 × 3.4 µm, Qe = 2.6
*GYJF21183*	(7.7–)8.3–9.3(−9.7) × (2.7–)3.1–3.5(−3.8) µm	Q = (2.3–)2.5–3.0(−3.4), *N* = 60	Me = 8.8 × 3.3 µm, Qe = 2.7
*GYJF21211*	(7.9–)8.3–9.4(−9.9) × (2.7–)3.0–3.5(−3.8) µm	Q = (2.2–)2.4–3.0(−3.3), *N* = 60	Me = 8.8 × 3.3 µm, Qe = 2.7
*GYJF21256*	(8.1–)8.4–9.4(−10.3) × (2.9–)3.2–3.7(−4.1) µm	Q = (2.2–)2.4–2.8(−3.1), *N* = 60	Me = 9.0 × 3.4 µm, Qe = 2.6
*GYJF21372*	(8.0–)8.4–9.9(−10.4) × (2.4–)3.0–3.6(−4) µm	Q = (2.2–)2.5–3.0(−3.5), *N* = 60	Me = 9.0 × 3.3 µm, Qe = 2.8
*GYJF21429*	(7.6–)8.1–9.4(−10.6) × (2.8–)3.1–3.6(−3.8) µm	Q = (2.2–)2.5–2.9(−3.4), *N* = 60	Me = 8.7 × 3.3 µm, Qe = 2.6
**Cumulated values**	**(7.6–)8.7–9.9(−11.2) × (2.4–)3.0–3.7(−4.1) µm**	**Q = (2.2–)2.3–3.0(−3.5), N = 420**	**Me = 9.0 × 3.4 µm, Qe = 2.6**
FWI (Fournier et al. 2020)	(9.6–) 10.3–12(−13.1) × (3.1–) 3.6–4.5(−4.9) µm	Q = (2.2–)2.4–3.2(−3.6), *N* = 120	Me = 11.1 × 4 µm, Qe = 2.8
Congo (Dennis 1961)	9–10.5 × 3–4 µm		Me = 9.8 × 3.5 µm, Qe = 2.8
French Guiana (Dennis 1961)	11–11.5 × 4–4.5 µm		Me = 11.3 × 4.3 µm, Qe = 2.6
México (San Martín and Rogers 1989)	10–12(−13) × 4–5 µm		Me = 11 × 4.5 µm, Qe = 2.4
Papua New Guinea (Van der Gucht 1995)	11–12.5 × 4.5 µm		Me = 11.7 × 4.6 µm, Qe = 2.5

≡ *Sphaeria gracillima* Fr., Linnaea 5: 538. 1830.

**Typification**: **FRENCH GUIANA.** Maripasoula, Saül, trail head to Sentier des Gros Arbres, 3.6201 N, 53.207989 W, on dead blackened wood, 27 Mar 2021, *Fournier, J. GYJF21177* (neotype of *X. gracillima* HAST 147325).

**Stromata** upright to prostrate, scattered to gregarious, simple, straight to strongly curved, 6–32 mm in total height, the fertile heads 4–26 mm high × 1–1.8 mm diam, cylindrical to narrowly fusiform, strongly nodulose with perithecial contours partly to fully exposed and deep circumferential wrinkles, apically ending into short to long mucronate sterile apices which are frequently broken off; surface dull black, finely roughened, devoid of peeling outer layer at maturity, but occasionally with remnants of a thin, fleeting brownish gray to silvery gray outer layer present on immature stromata; the stipes usually well-defined, 2–12 mm high × 0.35–0.85 mm diam, straight to slightly curved, blackish, flattened and longitudinally furrowed in the dry state, glabrous, tomentose and slightly swollen at the very base; subsurface a black leathery crust 30–50 µm thick; interior white to ivory between the perithecia, fibrous, with a narrow to wide continuous black layer lying just beneath the perithecia, increasing with age and eventually occupying the entire interior. **Perithecia** subglobose, fully to half-exposed, 0.30–0.50 mm diam. **Ostioles** finely papillate, black, 80–100 µm diam at base which is occasionally discoid up to 130 µm diam.

**Paraphyses** sparse, hyphal, thin-walled, 3–4 µm wide at base, tapering to 1–1.5 µm wide above asci, faintly guttulate, embedded in mucilaginous material. **Asci** cylindrical, with (6–)8 slightly overlapping uniseriately arranged ascospores, the spore-bearing parts 55–72 × 4.5–5.5 µm, the stipes 40–58 µm long, with apical apparatus short-cylindrical, apically flattened with a faint lateral rim, 1.5–2.1 × 1.2–1.6 µm (Me = 1.8 × 1.5 µm, *N* = 40), bluing in Melzer’s reagent. **Ascospores** (7.6–)8.7–9.9(−11.2) × (2.4–)3.0–3.7(−4.1) µm, Q = (2.2–)2.3–3.0(−3.5), *N* = 420 (Me = 9.0 × 3.4 µm, Qe = 2.6), ellipsoid-inequilateral, with narrowly to broadly rounded ends, ventrally flat to slightly concave, medium brown, unicellular, with a conspicuous, straight, longitudinally oriented germ slit almost spore-length on the ventral side; lacking appendages or mucilaginous sheath; epispore smooth.

**Asexual morph** on the natural substrate not observed.

**Other specimens examined: FRENCH GUIANA.** Maripasoula, Saül, trail to Monts-La-Fumée, 3.634796 N, 53.205457 W, ca. 270 m, disturbed mesophilic rainforest, on dead blackened stump, 2 Apr 2021, *Fournier, J. GYJF21372* (HAST 147322); Maripasoula, Saül, trail head to Sentier des Gros Arbres, 3.620324 N, 53.207951 W, ca. 210 m, disturbed secondary rainforest, on blackened wood, 18 Jun 2019, *Fournier, J. GYJF19087* (HAST 147323); Maripasoula, Saül, trail head to Sentier des Gros Arbres, 3.6201 N, 53.207989 W, on dead rotten wood, 27 Mar 2021, *Fournier, J. GYJF21183* (HAST 147324); Maripasoula, Saül, trail to Monts-La-Fumée, tributary to Crique Queue-Hocco, 3.637173 N, 53.204727 W, ca. 260 m, disturbed mesophilic rainforest, on dead rotten trunk, 4 Apr 2021, *Fournier, J. GYJF21429* (HAST 147326); Maripasoula, Saül, trail head to Sentier des Gros Arbres, 3.6201 N, 53.207989 W, on dead rotten trunk, 28 Mar 2021, *Fournier, J. GYJF21211* (HAST 147327); Maripasoula, Saül, shortcut to the airfield, 3.622852 N, 53.205768 W, 210–220 m, disturbed secondary rainforest, on dead blackened wood, 29 Mar 2021, *Fournier, J. GYJF21256* (HAST 147328).

**Known distribution**: French Guiana.

**Comments**: According to Dennis (1961), tropical *Xylaria* collections with short, slender, apiculate black stromata with strongly exposed perithecial contours, finely papillate ostioles, and ascospores 9–10.5 × 3–4 µm fit the concept of *X. gracillima*. The type material from French Guiana appears to be lost, but Dennis (1961) noted that a specimen bearing this name in the Fries herbarium (UPS) has slightly larger ascospores 11–11.5 × 4–4.5 µm (Table 15). However, in the Fries herbarium, F-175430 is the only specimen labeled as “*Sphaeria gracillima*?”; it originates from Cayenne, French Guiana, and is entirely sterile, consisting only of several stromatal stipes remaining on the bark. Its identity thus cannot be reconfirmed.

The seven collections from French Guiana cited herein are morphologically highly similar, and cluster together in our phylogenetic analyses. As Fries’ original material was also collected from French Guiana (Fries 1830), we designate HAST 147325 as the neotype of *X. gracillima*.

We consistently observed the presence of more or less developed black internal tissue within the stromata, a feature not reported by previous authors, but also present in *X*. *cylindrica* and *X. multiplex. Xylaria gracillima* and *X*. *cylindrica* cluster together in our phylogenetic analyses, whereas they do not show a close affinity to *X*. *multiplex*. Dennis (1961) suspected that *X. gracillima* represented a “state” of *X. multiplex* with more exposed perithecia; however, despite certain similarities, this is not supported, as the surface of *X*. *multiplex* consists of a blackish brown layer splitting in plaques encrusted with carbonaceous granules.

The status of the two collections from the French West Indies reported by Fournier et al. (2020), which differ by larger ascospores with a shorter germ slit, remains unresolved.

**Fig. 26:**
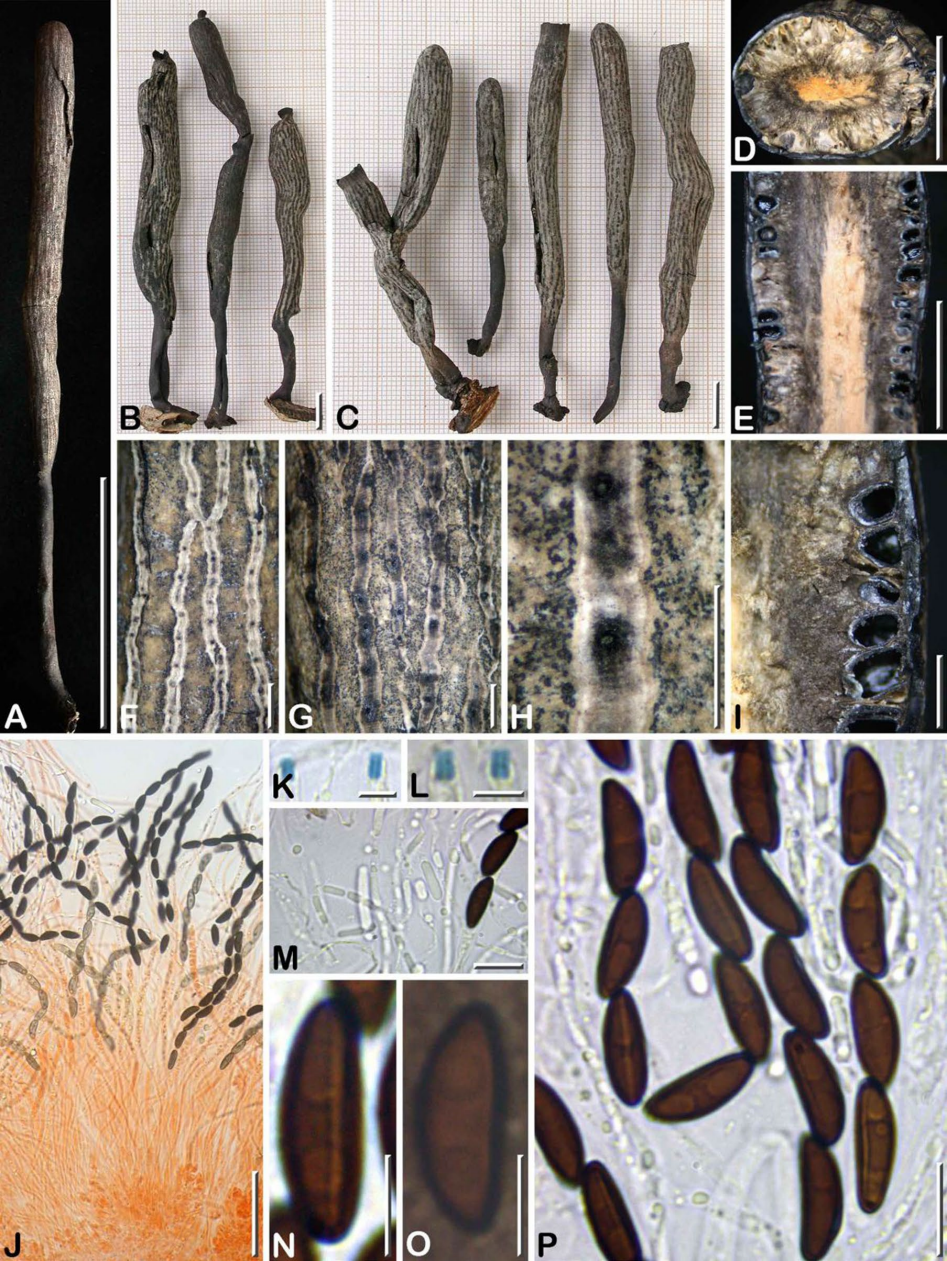
*Xylaria grammica* (**A, D, E, I ***GYJF18023*; **B ***GYJF18092*; **C, G, H ***GYJF18097*; **F ***GYJF21387*; **J, K, M, O ***GYJF19051*; **L, N, P ***GYJF18017*). **A–C** Variously shaped stromata. **D** Stroma in cross section. **E** Stroma in longitudinal section. **F** Surface of an immature stroma. **G** Surface of a mature stroma. **H** Surface of a mature stroma in close-up showing black granulations alongside the ostiolar rows. **I** Stromatal wall in longitudinal section. **J** Asci in Congo red and 3% KOH. **K, L** Ascal apical apparati in Melzer’s reagent. **M** Paraphyses with refractive content, in 1% SDS. **N** Ascospore in ventral view showing a straight germ slit, in 1% SDS. **O** Ascospore in India ink showing an absence of sheath or appendages. **P** Ascospores in 1% SDS, some showing a germ slit. Bars in **A** = 50 mm; **B, C** = 10 mm; **D, E** = 5 mm; **F, G, I** = 1 mm; **H** = 0.5 mm; **J =** 50 µm; **K, L, N, O** = 5 µm; **M, P** = 10 µm

***Xylaria grammica*** (Mont.) Fr., Nov. Act. Reg. Soc. Sci. Upsal., ser. III, 1: 128. 1851. Figure 26, Table 16

**Table 16:** Ascospore dimensions in four collections of *X. grammica* showing a narrow range of intraspecific variations, compared with those reported worldwide in literature

Collection numbers	Ascospore measurements with extreme values in parentheses	Q = quotient l/w, *N* = number of measurements	Me = mean values of l/w, Qe = mean quotient l/w
*GYJF18017*	(10.4–)10.9–12.6(−13.4) × (3.8–)4.0–4.6(−4.8) µm	Q = (2.3–)2.4–3.1(−3.4), *N* = 60	Me = 11.8 × 4.3 µm, Qe = 2.8
*GYJF18092*	(10.5–)11.0–12.8(−13.6) × (3.8–)4.0–4.7(−5.1) µm	Q = (2.2–)2.4–3.1(−3.6), *N* = 60	Me = 11.9 × 4.4 µm, Qe = 2.7
*GYJF18097*	(10.3–)10.8–12.6(−13.1) × (3.5–)4.1–4.9(−5.2) µm	Q = (2.2–)2.3–2.9(−3.3), *N* = 60	Me = 11.8 × 4.5 µm, Qe = 2.6
*GYJF19051*	(10.7–)11.4–12.9(−14.8) × (3.8–)4.1–4.8(−5.0) µm	Q = (2.3–)2.5–3.0(−3.7), *N* = 60	Me = 12.1 × 4.4 µm, Qe = 2.8
**Cumulated values**	**(10.3–)10.8–12.9(−14.8) × (3.5–)4.0–4.9(−5.2) µm**	**Q = (2.2–)2.3–3.1(−3.6), N = 240**	**Me = 11.9 × 4.4 µm, Qe = 2.7**
Neotropics (Dennis 1956)	11.5–15 × 3.5–4 µm		Me = 13.3 × 3.8 µm
Congo (Dennis 1961)	11–15 × 3.5–4 µm		Me = 13 × 3.8 µm
Taiwan (Ju and Tzean 1985)	11.5–13.5 × 4.5 µm		Me = 12.5 × 4.5 µm
North Sulawesi (Rogers et al. 1987)	(10.3-)11.7–14 × (4–)4.5–5(−6) µm		Me = 13 × 4.8 µm
México (San Martín and Rogers 1989)	(9.5–) 10–12(−12.5) × (4.5–)5–5.5 µm		Me = 11 × 5.3 µm
Papua New Guinea (Van der Gucht 1995)	(10–)10.5–13(−14) × 4–4.5 µm		Me = 11.4 × 4.4 µm

≡ *Hypoxylon grammicum* Mont., Ann. Sci. Nat. Bot., sér. II, 13: 341. 1840.

**Stromata** upright, arising singly or in small groups, frequently from dead trunks or blackened wood, rarely connate by two at base, 55–160 mm in total height, the fertile head narrowly clavate to slightly fusiform, straight to rarely slightly sinuous, 5–13 mm wide, with broadly, more rarely narrowly rounded fertile apices, occasionally with small sterile apical outgrowths, hard-textured, occasionally splitting lengthwise upon drying; surface of developing stromata mouse gray, gradually darkening with age, blackish when overmature, even, hard, lacking a peeling outer layer and exposed perithecial contours, finely roughened by minute black granulations, furrowed lengthwise by slightly sinuous, narrow whitish stripes through which ostioles emerge in linear rows; subsurface crust carbonaceous, 150–170 µm thick; interior off-white to brownish gray, solid, with a pale orange core, disintegrating and becoming hollow with age; the stipes mostly well-defined, 15–70 mm high, 3–6 mm wide, cylindrical, black, glabrous, carbonaceous, slightly enlarged and occasionally flattened at base. **Perithecia** subglobose to depressed-spherical, 0.8–1.0 mm diam. **Ostioles** obtusely papillate, barely prominent, black, 80–100 µm diam at base, surrounded by a blackish halo expanding with age.

**Paraphyses** hyphal, hyaline, rarely septate, simple, 1.0–2.8 µm wide, filled with massive refractive content dissolving into small guttules in 3% KOH, discreetly embedded in mucilaginous material. **Asci** cylindrical, with (6–)8 uniseriately arranged, slightly overlapping ascospores, the spore-bearing parts 86–94 µm long × 5.5–6.5 µm wide, the stipes 110–135 µm long, with apical apparatus tubular, apically flat with a low rim, 2.2–2.8 × 1.3–1.9 µm (Me = 2.6 × 1.6 µm, *N* = 45), bluing in Melzer’s reagent. **Ascospores** (10.3–)10.8–12.9(−14.8) × (3.5–)4.0–4.9(−5.2) µm, Q = (2.2–)2.3–3.1(−3.6), *N* = 240 (Me = 11.9 × 4.4 µm, Qe = 2.7), ellipsoid-inequilateral with most often narrowly rounded ends, frequently slightly ventrally concave, dark brown to blackish brown, unicellular, with a narrow, straight, longitudinally oriented germ slit almost spore-length on the ventral side, without sheath or appendages; epispore smooth.

**Asexual morph** on the natural substrate not observed.

**Specimens examined: FRENCH GUIANA.** Maripasoula, Saül, way up to Hmong village, 3.626053 N, 53.208075 W, 240–250 m, on dead wood, 2 Apr 2021, *Fournier, J. GYJF21387* (HAST 147329), immature; Maripasoula, Saül, trail head to Roche-Bateau, 3.619046 N, 53.200658 W, ca. 240 m, disturbed mesophilic rainforest, on dead corticated branch, 23 Aug 2018, *Fournier, J. GYJF18092* (HAST 147330); Maripasoula, Saül, trail head to Sentier des Gros Arbres, Crique Grand-Fossé, 3.6201 N, 53.207989 W, on dead trunk, 27 Mar 2021, *Fournier, J. GYJF21156* (HAST 147331), immature; Maripasoula, Saül, trail head to Sentier des Gros Arbres, Crique Grand-Fossé,3.620839 N, 53.208536 W, ca. 210 m, on dead trunk, 26 Mar 2021, *Fournier, J. GYJF21145* (HAST 147332); Maripasoula, Saül, trail head to Sentier des Gros Arbres, Crique Grand-Fossé, 3.617017 N, 53.209066 W, on dead trunk, 21 Jun 2019, *Fournier, J. GYJF19162* (HAST 147333); Maripasoula, Saül, shortcut to the airfield, 3.623524 N, 53.206542 W, 210–220 m, disturbed secondary rainforest, on dead wood, 17 Jun 2019, *Fournier, J. GYJF19051* (HAST 147334); Maripasoula, Saül, trail head to Roche-Bateau, 3.619046 N, 53.200658 W, ca. 240 m, disturbed mesophilic rainforest, on dead trunk, 23 Aug 2018, *Fournier, J. GYJF18097* (HAST 147335); Maripasoula, Saül, edge of the forest next to Carbets du bord, 3.623598 N, 53.210338 W, disturbed mesophilic rainforest, on dead blackened trunk, 20 Aug 2018, *Lechat, C. GYJF18002* (HAST 147336); Maripasoula, Saül, edge of the forest next to Carbets du bord, 3.623598 N, 53.210338 W, disturbed mesophilic rainforest, on dead blackened trunk, 19 Aug 2018, *Fournier, J. GYJF18017* (HAST 147337); Maripasoula, Saül, edge of the forest next to Carbets du bord, 3.624299 N, 53.209938 W, on dead trunk, 20 Aug 2018, *Fournier, J. GYJF18023* (HAST 147338).

**Known distribution**: Pantropical (Dennis 1956, 1958, 1961; Ju and Tzean 1985; Rogers et al. 1987; San Martín and Rogers 1989; Van der Gucht 1995; Hladki and Romero 2005).

**Comments**: *Xylaria grammica* is a distinctive species, remarkable by its tall, narrow, subclavate stromata featuring a surface striped by slightly sinuous paler bands in which ostioles open in linear rows. It is common in French Guiana, from which the holotype originates, and has also been reported from many tropical forests worldwide. Minor variations in ascospore dimensions (Table 16) do not suggest the existence of geographically distinct taxa.

Dennis (1961) discussed the status of *X. venustula* Sacc., which mainly differs from *X. grammica* by its smaller, narrowly cylindrical stromata 50 × 2–3 mm with a pointed apex. A collection of *X. venustula* from Taiwan was shown by Hsieh et al. (2010) to be phylogenetically unambiguously distant from *X. grammica*.

**Fig. 27:**
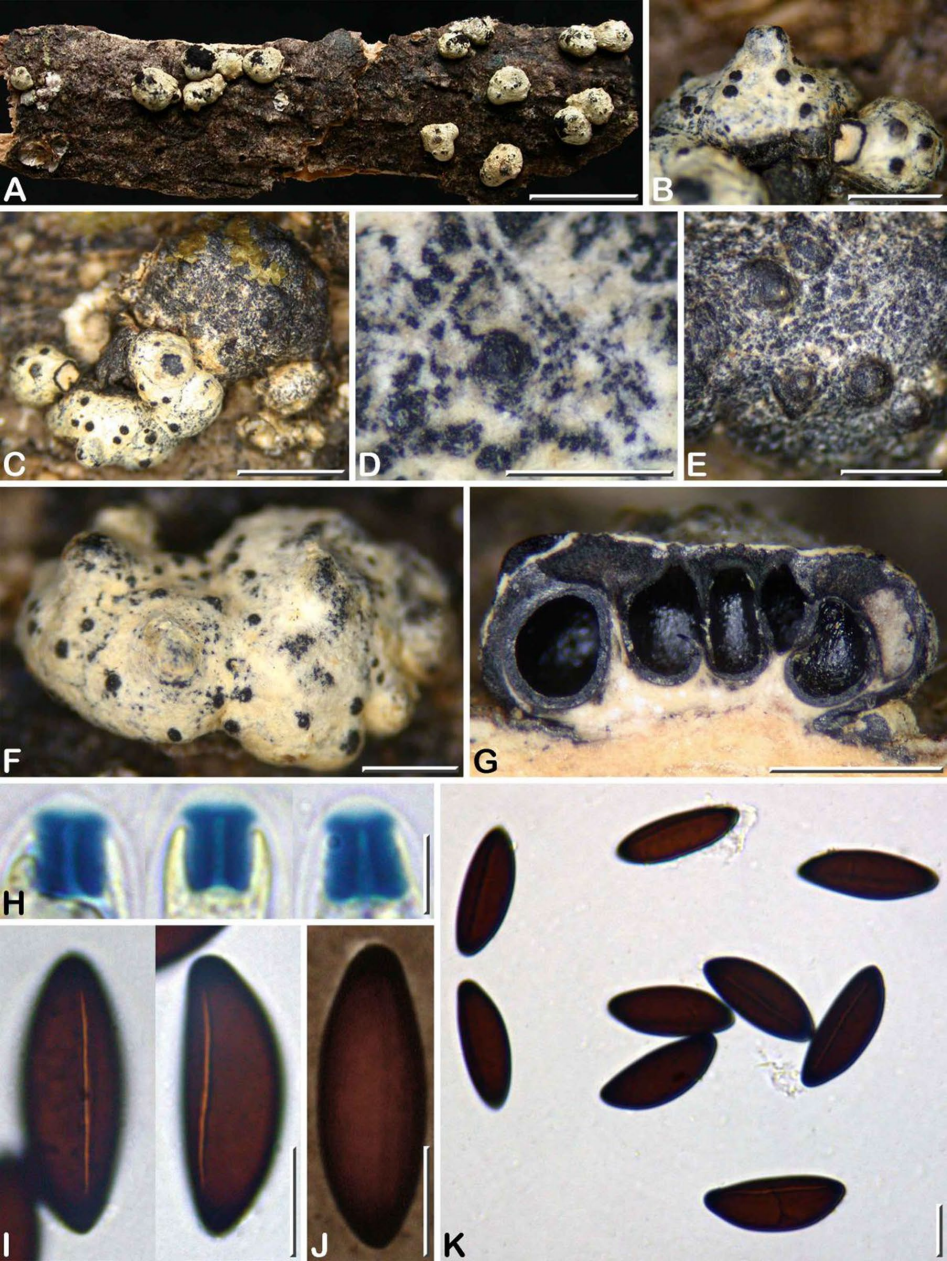
*Xylaria guadalupensis* (*CLL5437* holotype). **A** Scattered pulvinate stromata on bark. **B** Stroma in side view showing a centrally umbonate top coated with a cream-colored outer layer and ostioles underlined by black ejected ascospores deposits. **C** Small mature cream-colored stromata gathered around a bigger blackish overmature stroma. **D** Ostiolar region in close-up with black granules showing through the wearing off superficial layer. **E** Surface of a degraded overmature stroma showing densely distributed superficial granules and prominent, bluntly papillate ostioles. **F** Coalescent umbonate mature stromata. **G** Stroma in vertical section showing a broadly attached base. **H** Ascal apical apparati in Melzer’s reagent. **I** Ascospores in ventral view showing a germ slit, in 1% SDS. **J** Ascospore in dorsal view showing a lack of sheath or appendages, in India ink. **K** Mature ejected ascospores, in 1% SDS. Bars in **A** = 5 mm; **B, F, G** = 1 mm; **C** = 2 mm; **D, E** = 0.5 mm; **H** = 5 µm; **I–K** = 10 mm

***Xylaria guadalupensis*** J. Fourn., Y.-M. Ju, H.-M. Hsieh & Lechat, sp. nov. Figure 27, Table 17

**Table 17:** Ascospore and ascal apical apparatus dimensions from two different stromata from the holotype collection of *X. guadalupensis*, showing the variation range between ascospores in the hymenium and those ejected on the stromatal surface

Collection numbers	Ascospore measurements with extreme values in parentheses, *N* = number of measurements	Me = mean values of l/w, Qe = mean quotient l/w	Ascal apical apparatus, *N* = 25
*CLL5437* hymenial ascospores	(21.1–)23.1–26.6(−27.4) × (7.8–)8.2–9.3(−9.6) µm	Me = 24.7 × 8.7 µm, Qe = 2.9	4.7–5.8 × 3.5–4.4 µm, Me = 5.3 × 3.9 µm
*CLL5437* ejected ascospores	(22.2–)23.6–26.4(−27.7) × (8.3–)8.6–10.2(−10.6) µm	Me = 24.9 × 9.3 µm, Qe = 2.7	
**Cumulated values**	**(21.1–)23.1–26.6(−27.7) × (7.8–)8.2–10.2(−10.6) µm, Q = (2.2–)2.4–3.0(−3.4), N = 120**	**Me = 24.8 × 9.0 µm, Qe = 2.8**	

MycoBank MB 863167

**Typification: FRENCH WEST INDIES.** GUADELOUPE: Basse-Terre, on a dead corticated branch, Nov 2005, *Lechat, C. CLL5437* (holotype HAST 142968).

**Etymology:** After the Caribbean island of Guadeloupe, where the holotype was collected.

**Diagnosis:** Differs from *X. cantareirensis* by its frequently umbonate stromata, more prominent ostioles, lack of associated synnemata, and smaller ascospores 24.8 × 9.0 µm vs 28.3 × 10.9 µm.

**Stromata** scattered to loosely clustered, occasionally in contact and coalescent, subglobose to depressed-spherical or pulvinate, convex, frequently centrally umbonate, 2–8-peritheciate, 1.0–1.8 mm high × 1.0–3.4 mm diam, sessile, on a broad to somewhat restricted central connective. Surface of developing and mature stromata entirely coated by a thick, finely granular cream-colored superficial layer, gradually wearing off from the base, longer persistent on top, revealing a grayish black carbonaceous subsurface coarsely incrusted with black, carbonaceous, superficial granules; perithecial contours unexposed; subsurface crust weakly carbonaceous, 60–90 µm thick, black; interior whitish to yellowish, solid to fibrous. **Perithecia** subglobose to laterally flattened, 0.60–1.0 mm diam. **Ostioles** bluntly papillate, prominent, black, 130–170 µm diam.

**Paraphyses** copious, hyphal, thin-walled, filiform, sparsely guttulate, 5.0–6.0 µm wide at base, tapering to 1.8–2.5 µm wide above asci, embedded in mucilaginous material. **Asci** fragmentary, not measured, short-stipitate, with apical apparatus 4.7–5.8 × 3.5–4.4 µm (Me = 5.3 × 3.9 µm, *N* = 25), cylindrical with straight parallel sides to frequently trapezoid flaring at base, with a faintly prominent upper rim, strongly bluing in Melzer’s reagent.

**Ascospores** (21.1–)23.1–26.6(−27.7) × (7.8–)8.2–10.2(−10.6) µm, Q = (2.2–)2.4–3.0(−3.4), *N* = 120 (Me = 24.8 × 9.0 µm, Qe = 2.8), ellipsoid-inequilateral with broadly to most often narrowly rounded ends, unicellular, dark brown, with a longitudinal, straight germ slit slightly less than spore-length on the ventral side; lacking secondary appendages or mucilaginous sheath; epispore smooth.

**Asexual morph** on the natural substrate not observed.

**Known distribution**: Guadeloupe (French West Indies), known from only one collection.

**Comments**: *Xylaria guadalupensis* is proposed to accommodate a collection initially identified as *X. cantareirensis* (Fournier et al. 2018c), which proved morphologically distinct after examination of the lectotype from Brazil (this paper). Its isolated position in our phylogram, clearly distant from the collections regarded as closely related to *X. cantareirensis*, supports its recognition as a distinct taxon.

**Fig. 28:**
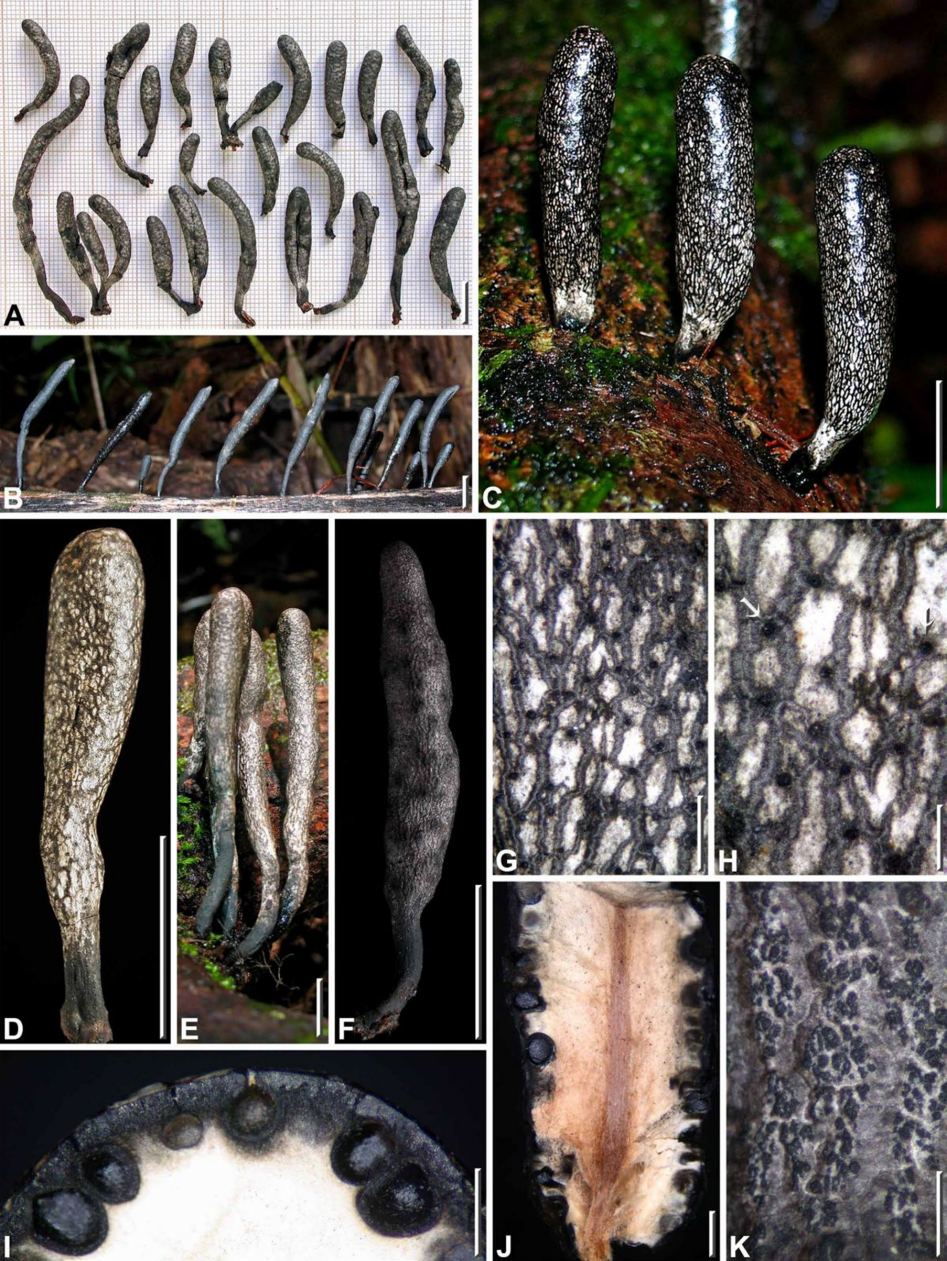
*Xylaria guianica* (**A ***CLL9025*; **B ***GYJF21015*; **C, G–I ***GYJF21240* holotype; **D ***CLL7072*; **E, J ***GYJF21408*; **F, K ***GYJF12228*). **A** Variously shaped stromata in the dry state, some longitudinally split. **B** Blackish stromata in the fresh state, in situ, some showing a conical apex. **C** Broadly cylindrical subsessile stromata in the fresh state, in situ. **D** Clavate stroma in the dry state. **E** Bunch of narrowly clavate, long-stipitate stromata in the fresh state, in situ. **F** Overmature stroma having lost the superficial white scales. **G, H** Stromatal surface in close-up at different magnifications, showing appressed white scale and small black ostioles. **I** Stromatal apex in vertical section showing perithecia fully immersed beneath a thick carbonaceous crust. **J** Stromatal base in vertical section showing a faintly pigmented interior with a reddish brown core extending into the stipe. **K** Stromatal surface of a blackish overmature stroma showing black granular deposits where the white scales were formerly present. Bars in **A–F** = 10 mm; **G, J** = 1 mm; **H, I, K** = 0.5 mm

**Fig. 29:**
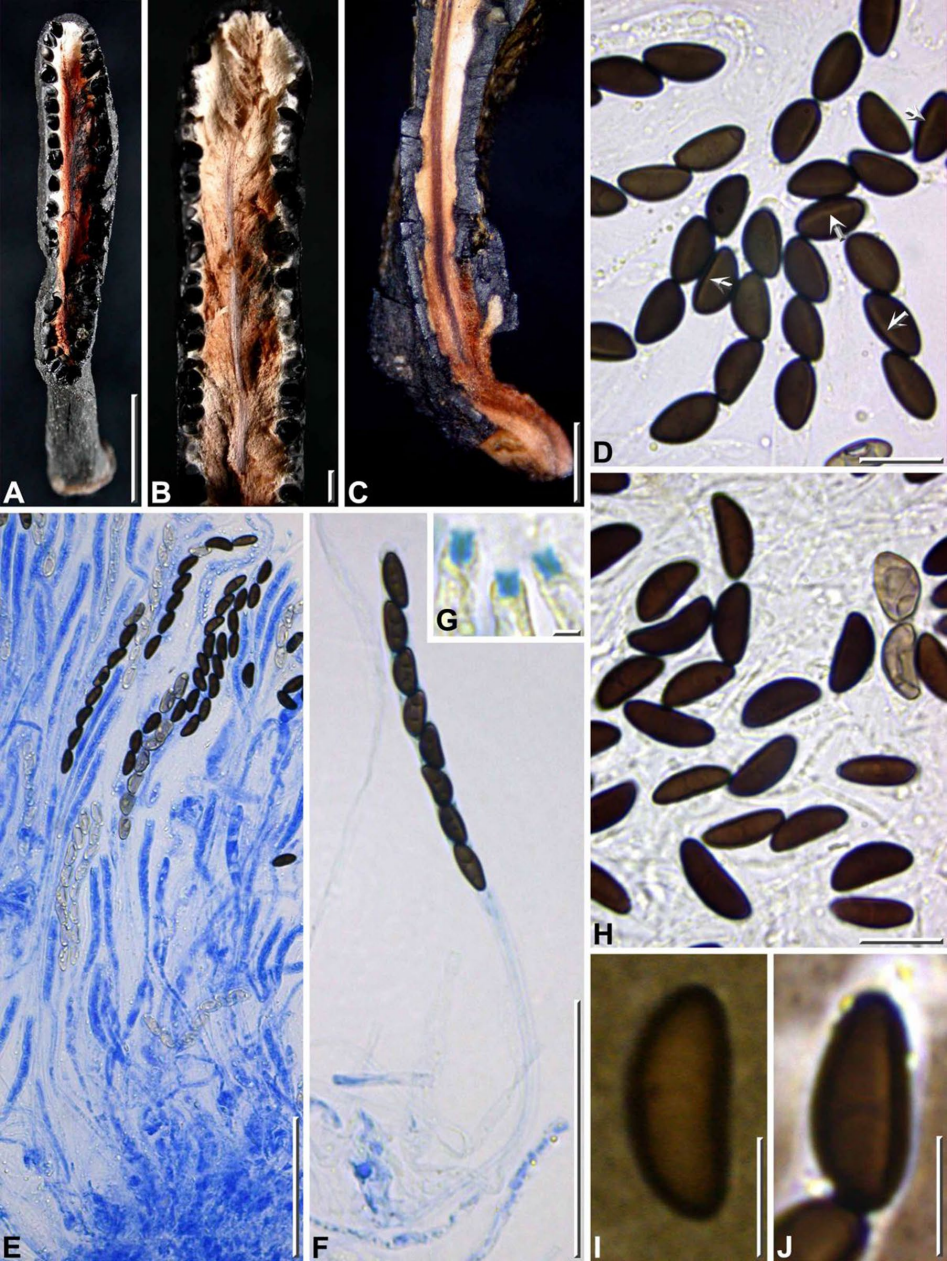
*Xylaria guianica* (**A, B ***GYJF12029*; **C, I ***CLL9025*; **D, E–G, J ***GYJF21240* holotype; **H ***CLL8005*). **A, B** Mature stromata in vertical section showing a hollow orange-brown interior. **C** Vertical section of a stromatal stipe showing a thick carbonaceous crust and a brick interior at base, **D, H** Variously shaped ascospores in 1% SDS, some showing germ slits (arrows in **D**). **E** Bunch of immature and mature asci, in blue Pelikan ink. **F** Long-stipitate mature ascus, in blue Pelikan ink. **G** Ascal apical apparati in Melzer’s reagent. **I** Ascospore in side view in India ink showing an absence of sheath or appendage. **J** Ascospore in ventral view still in the ascus, showing a germ slit, in India ink. Scale bars: **A** = 5 mm; **B, C** = 1 mm; **D, H** = 10 µm; **E, F** = 50 µm; **G** = 2 µm; **I, J** = 5 µm

***Xylaria guianica*** J. Fourn., Y.-M. Ju, H.-M. Hsieh & Lechat, sp. nov. Figures 28, 29, Table 18

**Table 18:** Ascospore and ascal apical apparatus dimensions in twelve collections of *X. guianica* showing a fairly wide variation range of ascospore dimensions

Collection numbers	Ascospore measurements with extreme values in parentheses	Q = quotient l/w, *N* = number of measurements	Me = mean values of l/w, Qe = mean quotient l/w	Ascal apical apparatus h × w, *N* = 20
*CLL7093*	(8.1–)8.6–9.8(−10.1) × (3.5–)3.9–4.6(−4.8) µm	Q = (1.9–)2.0–2.4(−2.6), *N* = 60	Me = 9.1 × 4.2 µm, Qe = 2.1	2.0–2.3 × 1.3–1.4 µm, Me = 2.1 × 1.4 µm
*CLL8005*	(9.1–)9.4–10.6(−12.2) × (3.7–)3.9–4.6(−5) µm	Q = (2.0–)2.1–2.5(−2.7), *N* = 60	Me = 10.0 × 4.3 µm, Qe = 2.3	1.8–2.2 × 1.4–1.6 µm, Me = 2.0 × 1.5 µm
*CLL9025*	(8.2–)8.6–9.8(−10.6) × (3.7–)3.9–4.5(−4.7) µm	Q = (1.9–)2.0–2.4(−2.6), *N* = 60	Me = 9.1 × 4.2 µm, Qe = 2.2	1.8–2.1 × 1.2–1.5 µm, Me = 1.9 × 1.4 µm
*GYJF12029*	(8.0–)8.4–9.6(−10.0) × (3.5–)3.7–4.2(−4.4) µm	Q = (1.9–)2.0–2.5(−2.7), *N* = 60	Me = 8.9 × 4.0 µm, Qe = 2.3	
*GYJF12090*	(8.3–)8.5–9.6(−10.5) × (3.5–)3.7–4.3(−4.6) µm	Q = (2.0–)2.1–2.5(−2.7), *N* = 60	Me = 9.1 × 4.0 µm, Qe = 2.3	2.0–2.3 × 1.3–1.7 µm, Me = 2.1 × 1.5 µm
*GYJF12228*	(9.4–)9.5–10.8(−12.6) × (3.8–)4.0–4.6(−5.1) µm	Q = (1.9–)2.2–2.6(−2.8), *N* = 60	Me = 10.2 × 4.4 µm, Qe = 2.3	
*GYJF18048*	(8.7–)9.1–10.3(−11.6) × (3.5–)3.8–4.5(−4.8) µm	Q = (2.0–)2.1–2.6(−2.9), *N* = 60	Me = 9.7 × 4.1 µm, Qe = 2.4	
*GYJF21007*	(7.7–)8.3–9.7(−10.4) × (3.7–)3.9–4.5(−4.7) µm	Q = (1.8–)2.0–2.4(−2.7), *N* = 60	Me = 8.9 × 4.2 µm, Qe = 2.2	1.9–2.2 × 1.3–1.6 µm, Me = 2.1 × 1.5 µm
*GYJF21015*	(9.3–)9.5–10.9(−11.4) × (3.6–)3.9–4.6(−4.8) µm	Q = (2.0–)2.2–2.7(−2.9), *N* = 60	Me = 10.2 × 4.2 µm, Qe = 2.4	2.0–2.4 × 1.4–1.7 µm, Me = 2.3 × 1.5 µm
*GYJF21240* holotype	(7.6–)8.2–9.8(−10.1) × (3.7–)3.8–4.5(−4.6) µm	Q = (1.8–)2.0–2.4(−2.7), *N* = 60	Me = 8.9 × 4.1 µm, Qe = 2.2	1.9–2.5 × 1.3–1.6 µm, Me = 2.2 × 1.4 µm
*GYJF21408*	(7.5–)7.9–9.3(−10.0) × (3.6–)3.8–4.3(−4.8) µm	Q = (1.7–)1.9–2.4(−2.6), *N* = 60	Me = 8.6 × 4.1 µm, Qe = 2.1	2.0–2.2 × 1.3–1.5 µm, Me = 2.1 × 1.4 µm
*JLC-G5*	(8.1–)8.6–9.7(−10.5) × (3.8–)3.9–4.7(−4.8) µm	Q = (1.9–)2.0–2.3(−2.6), *N* = 60	Me = 9.1 × 4.3 µm, Qe = 2.1	
**Cumulated values**	**(7.5–)7.9–10.9(−12.6) × (3.5–)3.7–4.7(−5.1) µm**	**Q = (1.7–)1.9–2.7(−2.9), N = 720**	**Me = 9.3 × 4.2 µm, Qe = 2.2**	**1.8–2.5 × 1.2–1.7 µm, Me = 2.1 × 1.5 µm**

MycoBank MB863168

**Typification**: **FRENCH GUIANA.** Maripasoula, Saül, shortcut to the airfield, 3.622852 N, 53.205768 W, 210–220 m, disturbed secondary rainforest, on dead blackened wood, 29 Mar 2021, *Fournier, J. GYJF21240* (holotype HAST 147341).

**Etymology**: After (French) Guiana where all collections originate.

**Diagnosis**: Differs from similar species with clavate, strongly carbonaceous stromata by its superficial reticulate network delimiting persistent white scales, an orange-brown interior and ascospores averaging 9.3 × 4.2 µm, with mostly broadly rounded ends and a straight germ slit nearly spore-length.

**Stromata** (12–)18–48(−63) mm in total height, upright, the fertile parts 7–40 mm high, 3–8 mm wide, subcylindrical to subclavate, terete, occasionally flattening upon drying, with broadly rounded, occasionally conical fertile apices, simple, exceptionally forked, loosely scattered, rarely in small bunches of 2–4 connate at base, straight to slightly curved, very hard-textured, becoming hollow and sometimes splitting upon drying, stipitate to subsessile; the stipes well- to ill-defined, 4–30 mm high, 1.8–3(−4) µm wide, carbonaceous, terete to flattened-twisted in the dry state, glabrous, white in upper half, blackish and slightly swollen at base, entirely black with age; surface smooth with unexposed perithecial contours, silvery gray to dark gray, a dense network of dark gray shallow cracks delimiting mostly elongated, angular, long persistent, pure white, tightly appressed scales which, as they wear off with age, reveal the presence of underlying, clustered, black carbonaceous granules; subsurface a strongly carbonaceous black crust 150–200 µm thick; the tissue around perithecia white, restricted to a thin layer surrounding them, underlying interior solid, fibrous, whitish at apex when young, vinaceous brown in the fresh state, fading to light orange-brown and shrinking upon drying, more pigmented at base with an orange-brown core extending into the stipe, distinctly brick (59) at full maturity and overmature state. **Perithecia** fully immersed, subglobose to laterally flattened, 0.55–0.85 mm diam, mostly in contact. **Ostioles** discoid, flat, level with the surface, black, ca. 80 µm diam, opening between the white scales.

**Paraphyses** hyphal, hyaline, remotely septate, 4.5–5.0 µm wide at base, tapering to 1.0–1.5 µm wide above asci, discreetly embedded in mucilaginous coating. **Asci** cylindrical, with (6–)8 uniseriately arranged, slightly overlapping ascospores, the spore-bearing parts 67–78 µm long × 5.5–6.5 µm wide, the stipes 80–105 µm long, fragile, with apical apparatus tubular, occasionally apically flared, with a faint upper rim, 1.8–2.5 × 1.2–1.7 µm (Me = 2.1 × 1.5 µm, *N* = 140), bluing in Melzer’s reagent. **Ascospores** (7.5–)7.9–10.9(−12.6) × (3.5–)3.7–4.7(−5.1) µm, Q = (1.7–)1.9–2.7(−2.9), *N* = 720 (Me = 9.3 × 4.2 µm, Qe = 2.2), ellipsoid-inequilateral with mostly broadly rounded ends, ventrally flat to slightly convex or concave, unicellular, dark brown, with a narrow, straight, longitudinally oriented germ slit almost spore-length on the ventral side; lacking sheath or appendages; epispore smooth.

**Asexual morph** on the natural substrate not observed.

**Other specimens examined (paratypes)**: **FRENCH GUIANA.** Saint-Georges, forest trail from Savane-Roche, 3.977742 N, 51.868988 W, ca. 60 m, lowland rainforest, on dead wood, 21 Apr 2009, *Lechat, C. CLL9025* (HAST 147339); Roura, Cacao, Camp Bonaventure, 4.323044 N, 52.339371 W, 59 m, disturbed hygrophilic rainforest, on dead trunk, 20 Mar 2021, *Fournier, J. GYJF21007* (HAST 147340); Roura, Cacao, Camp Bonaventure, 4.326815 N, 52.341431 W, 60–65 m, on dead corticated branch, 22 Mar 2021, *Fournier, J. GYJF21051* (HAST 147342); Régina, Nouragues natural reserve, Inselberg camp, 4.311240 N, 52.132789 W, ca. 300 m, hygrophilic rainforest, on dead corticated branch, 17 Jun 2012, *Lechat, C. GYJF12029* (HAST 147343); Régina, Nouragues natural reserve, Inselberg camp, 4.311240 N, 52.132789 W, ca. 300 m, hygrophilic rainforest, on dead corticated branch, 19 Jun 2012, *Fournier, J. GYJF12090* (HAST 147344); Roura, Cacao, Camp Bonaventure, 4.323044 N, 52.339371 W, 59 m, disturbed hygrophilic rainforest, on dead corticated branch, 20 Mar 2021, *Fournier, J. GYJF21015* (HAST 147345); Maripasoula, Saül, trail to Monts-La Fumée, tributary of Crique Queue-Hocco, 3.637173 N, 53.204727 W, ca. 260 m, disturbed mesophilic rainforest, on dead corticated branch, 4 Apr 2021, *Fournier, J. & Thonnel, A. GYJF21408* (HAST 147346); Maripasoula, Saül, trail head to Roche-Bateau, 3.619046 N, 53.200658 W, ca. 230 m, disturbed mesophilic rainforest, on dead rotten wood, 23 Aug 2018, *Fournier, J. GYJF18101* (HAST 147347), depauperate; Saint-Georges, forest trail from Savane-Roche, 3.977742 N, 51.868988 W, ca. 60 m, lowland rainforest, on dead wood, 26 Apr 2010, *Lechat, C. CLL10033* (HAST 147348); Maripasoula, Saül, trail head to Monts-La-Fumée, 3.629382 N, 53.206831 W, disturbed mesophilic rainforest, on dead wood, 21 Mar 2005, *Cheype, J.-L. JLC-G5* (HAST 147349); Sinnamary, Saint-Elie, Saint-Elie botanical trail, 5.292020 N, 53.05262 W, mesophilic rainforest, on dead corticated branch, 26 Jun 2012, *Fournier, J. GYJF12228* (HAST 147350); Sinnamary, Paracou, CIRAD field centre, Guyaflux plot, 5.249218 N, 52.915431 W, lowland rainforest, on dead wood, 27 Feb 2007, *Lechat, C. CLL7072* (HAST 147351); Kourou, Sentier de la Montagne des Singes, 5.068808 N, 52.69478 W, lowland rainforest, on dead corticated branch, 27 Apr 2008, *Lechat, C. CLL8005* (HAST 147352); Maripasoula, Saül, trail head to Monts-La-Fumée, 3.629382 N, 53.206831 W, disturbed mesophilic rainforest, on dead corticated branch, 21 Aug 2018, *Gardiennet, A. GYJF18048* (HAST 147353); Maripasoula, Saül, trail head toward Mont-La Fumée, 3.6255 N, 53.208254 W, 230 m, disturbed secondary rainforest, on dead wood, 19 Jun 2019, *Fournier, J. GYJF19091-2* (HAST 147354); Roura, Cacao, Camp Bonaventure, 4.326815 N, 52.341431 W, 60–65 m, secondary hygrophilic rainforest, on blackened wood, 22 Mar 2021, *Fournier, J. GYJF21085* (HAST 147355); Macouria, Risquetout Forest, trail to Saut-Léodate, 4.899372 N, 52.555061 W, ca. 55 m, lowland rainforest, on dead corticated branch, 1 Mar 2007, *Lechat, C. CLL7093* (HAST 147356).

**Known distribution**: French Guiana.

**Comments**: *Xylaria guianica* can be readily recognized in the field by its rather small, subclavate, strongly carbonaceous stromata with a broadly rounded apex; persistent, pure white, tightly appressed scales delineated by a dense network of blackish, shallow cracks; inconspicuous discoid ostioles; and a vinaceous stromatal interior. Its dark brown ascospores, 7.9–10.9 × 3.7–4.7 µm, are fairly variable in shape and dimensions but clearly distinguish *X*. *guianica* from *X. dealbata* and *X. macouriana*, which share a similar stromatal surface (this paper).

The vinaceous color of the stromatal interior is a diagnostic feature when observed in fresh material but may be difficult to discern in young or barely mature stromata in the dried state, unless the orange-brown core at the base of the stipe is visible.

During the identification of our first collections, we compared our material with *X. pallida* Berk. & Cooke. Examination of the holotype [**BRAZIL.** Papunha, on sticks, 5 Nov 1873, *Trail 21* (K(M) 57346)] revealed notable differences despite the shared cream-white, reticulately cracked surface. *Xylaria pallida* differs by having small, stipitate stromata with a sterile apex, larger ascospores 11–13 12 × 4.5–5 µm [10.5–12 × 4.5–5 µm in Dennis (1956)], and a straight ascospore germ slit less than spore-length. The holotype includes only one mature stroma, which was not sectioned to assess the interior color.

It is noteworthy that *X. collabens* (Mont.) Fr. [as *X tuberoides* Rehm in Fournier et al. (2019)] is the only known species featuring a similarly loose, fibrous, vinaceous interior visible in fresh material. It differs from *X. guianica* by its subglobose stromata 5–18 mm diam and narrowly fusoid-inequilateral ascospores 23–28.9 × 6–7.4 µm with a short germ slit.

Seventeen collections of *X. guianica* show that the species is widespread in French Guiana across diverse habitats. Its distribution is probably not restricted to this part of the Amazonian basin, which warrants confirmation through further fieldwork.

**Fig. 30:**
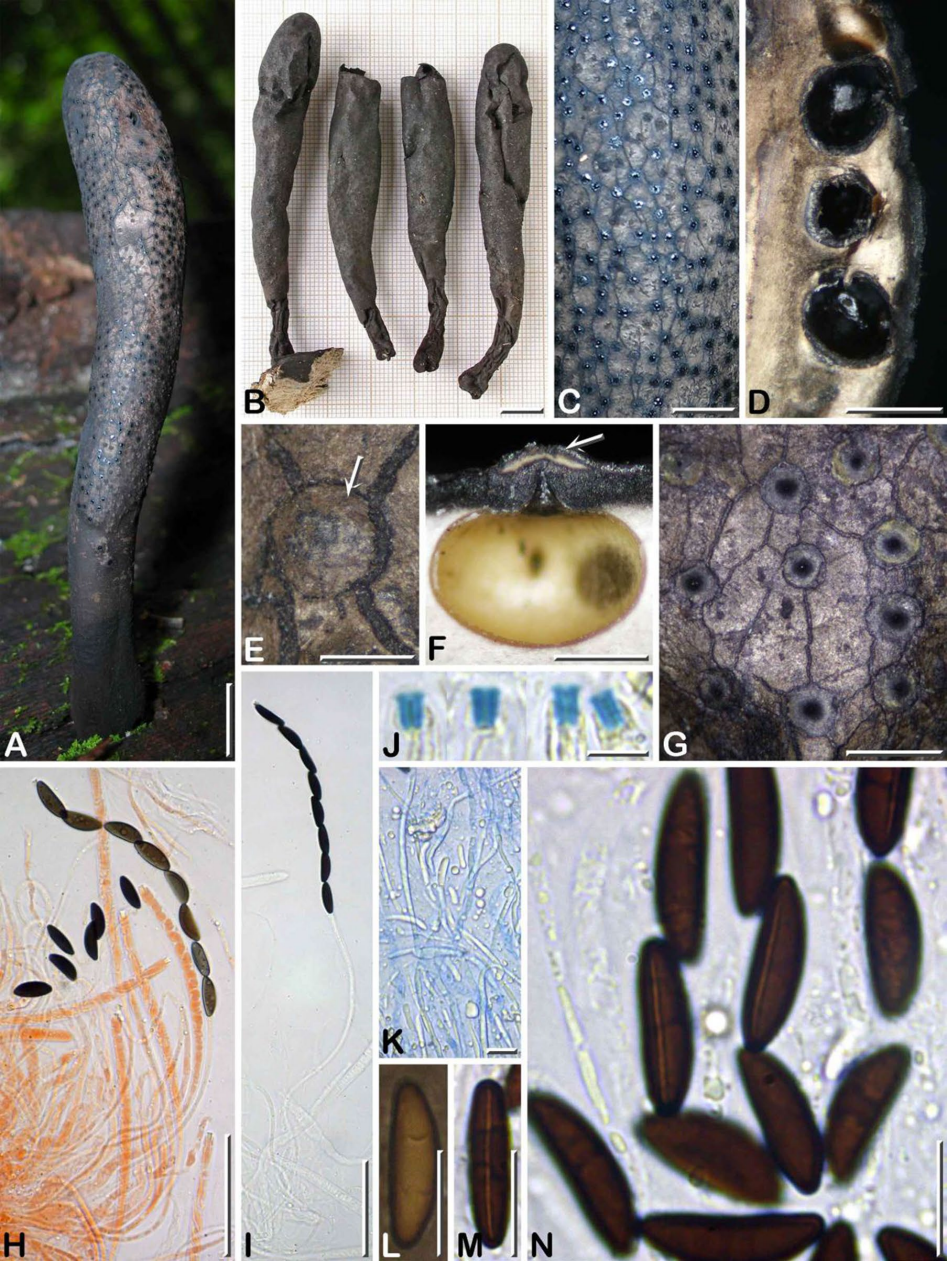
*Xylaria guyanensis* (**A, C ***GYJF21353*; **B, G ***GYJF19125*; **D, L ***GYJF18117*; **E, F ***JLC-G8*; **H–J, N ***GYJF21279*; **K, M ***GYJF18019*). **A** Stroma in the fresh state, in situ. **B** Stromata in the dry state. **C** Surface of a fresh stroma showing a network of cracks joining shiny black ostioles. **D** Stromatal wall in longitudinal section. **E** Surface of an immature stroma in close-up showing an ostiolar operculum (arrow). **F** Immature perithecium in vertical section showing an operculum (arrow) and a basal layer of white tissue covering a papillate ostiole. **G** Surface of a mature stroma showing ostiolar discs in a network of superficial shallow cracks. **H, I** Asci respectively in Congo red and 3% KOH. **J** Ascal apical apparati in Melzer’s reagent. **K** Paraphyses with massive refractive content, in blue Pelikan ink. **L** Ascospore in India ink showing an absence of sheath or appendages. **M** Ascospore in ventral view showing a straight germ slit in Melzer’s reagent. **N** Variously oriented ascospores, in 1% SDS. Bars in **A, B** = 10 mm; **C** = 5 mm; **D, G** = 1 mm; **E, F** = 0.5 mm; **H, I** = 50 µm; **J** = 5 µm; **K–N** = 10 µm

***Xylaria guyanensis*** (Mont.) Fr., Nov. Act. Reg. Soc. Sci. Upsal., ser. III, 1: 127. 1851. Figure 30, Table 19

**Table 19:** Ascospore dimensions in four collections of *X. guyanensis* showing a wide range of intraspecific variations, compared with those reported from the holotype and those from a collection from Mexico

Collection numbers	Ascospore measurements with extreme values in parentheses	Q = quotient l/w, *N* = number of measurements	Me = mean values of l/w, Qe = mean quotient l/w
*GYJF18019*	(15.7–)16.2–18.4(−19.4) × (4.4–)4.7–5.3(−5.4) µm	Q = (3.0–)3.2–3.8(−4.3), *N* = 60	Me = 17.2 × 5.0 µm, Qe = 3.5
*GYJF18117*	(15.7–)15.9–18.8(−20.0) × (4.3–)4.6–5.4(−5.7) µm	Q = (2.9–)3.2–3.9(−4.1), *N* = 60	Me = 17.3 × 5.0 µm, Qe = 3.5
*GYJF19125*	(14.3–)15.2–17.5(−19.4) × (4.1–)4.3–5.3(−5.8) µm	Q = (2.8–)3.1–3.8(−4.4), *N* = 60	Me = 16.3 × 4.8 µm, Qe = 3.4
*GYJF21279*	(13.3–)14.4–17.0(−18.3) × (3.9–)4.6–5.3(−6.0) µm	Q = (2.5–)2.8–3.5(−4.4), *N* = 60	Me = 15.5 × 5.0 µm, Qe = 3.1
**Cumulated values**	**(13.3–)14.4–18.8(−20.0) × (3.9–)4.3–5.4(−6.0) µm**	**Q = (2.5–)2.8––3.9(−4.4), N = 240**	**Me = 16.6 × 5.0 µm, Qe = 3.3**
FG holotype (Dennis 1956)	14–21 × 5–8 µm		Me = 17.5 × 6.5 µm, Qe = 2.7
Mexico (San Martín and Rogers 1989)	(14–)15–17.5 × 4.5–6 µm		Me = 16.3 × 5.3 µm, Qe = 3.1

≡ *Hypoxylon guyanense* Mont., Ann. Sci. Nat. Bot., sér. II, 13: 343. 1840.

**Stromata** upright, arising singly or in small groups from blackened wood on ill-defined stipes, 45–92 mm in total height, the fertile head clavate to fusiform, rarely narrowly cylindrical or strongly flattened, straight to slightly curved, (5–)8–15(−23) mm wide, with broadly rounded fertile apices, shriveling and occasionally splitting lengthwise upon drying; surface of developing stromata grayish brown, blackish brown at maturity, even, lacking a peeling outer layer and exposed perithecial contours, finely roughened by ostioles and a dense network of shallow cracks delimiting polygonal plaques and ostiolar discs; subsurface crust carbonaceous, 150–170 µm thick; interior off-white to brownish gray, solid, slightly gelatinous in the fresh state, compact and becoming partly hollow in the dry state; the stipes mostly ill-defined, 14–30 mm high, 3–6 mm wide, black, glabrous, shriveled, slightly enlarged at base. **Perithecia** spaced, subglobose to depressed spherical, 0.9–1.2 mm diam. **Ostioles** finely conic-papillate, black, ca 120 µm diam at base, located in a slightly sunken gray to dark gray discoid area 380–540 µm diam; observation of immature stromata shows that discs result from the dehiscence of a circular operculum raised by the swelling of a white basal tissue, either in one piece or by stellate fragmentation.

**Paraphyses** hyphal, hyaline, rarely septate, simple, 1.0–2.7 µm wide, filled with massive refractive content dissolving into small guttules in 3% KOH, discreetly embedded in mucilaginous material. **Asci** cylindrical, with (6–)8 uniseriately arranged, slightly overlapping ascospores, the spore-bearing parts 100–125 µm long × 6.0–7.0 µm wide, the stipes 85–180 µm long, with apical apparatus tubular, occasionally slightly attenuated at base, apically flat with a fairly conspicuous rim, 2.8–3.4 × 1.7–2.3 µm (Me = 3.1 × 2.0 µm, *N* = 50), bluing in Melzer’s reagent. **Ascospores** (13.3–)14.4–18.8(−20.0) × (3.9–)4.3–5.4(−6.0) µm, Q = (2.5–)2.8––3.9(−4.4), *N* = 240 (Me = 16.6 × 5.0 µm, Qe = 3.3), narrowly ellipsoid-inequilateral with broadly rounded ends, frequently slightly ventrally concave, dark brown to blackish brown, unicellular, with a narrow, straight, longitudinally oriented germ slit almost spore-length on the ventral side, without sheath or appendages; epispore smooth.

**Asexual morph** on the natural substrate not observed.

**Specimens examined: FRENCH GUIANA.** Maripasoula, Saül, trail head to Sentier des Gros Arbres, 3.620564 N, 53.208511 W, on dead blackened wood, 24 Aug 2018, *Fournier, J. GYJF18117* (HAST 147357); Maripasoula, Saül, trail head to Sentier des Gros Arbres, 3.617198 N, 53.209029 W, on dead blackened log, 1 Apr 2021, *Fournier, J. GYJF21353* (HAST 147358); Maripasoula, Saül, trail to Monts-La-Fumée, on dead wood, 21 Mar 2005, *Cheype, J.-L. JLC-G8* (HAST 147359), immature; Maripasoula, Saül, trail head to Sentier des Gros Arbres, 3.6201 N, 53.207989 W, on dead blackened wood, 28 Mar 2021, *Fournier, J. GYJF21226* (HAST 147360), immature; Maripasoula, Saül, edge of the forest next to Carbets du bord, 3.623598 N, 53.210338 W, disturbed mesophilic rainforest, on dead blackened trunk, 19 Aug 2018, *Fournier, J. GYJF18013* (HAST 147361); Maripasoula, Saül, shortcut to the airfield, 3.622159 N, 53.204166 W, disturbed secondary rainforest, 210–220 m, on dead blackened log on marshy ground, 20 Jun 2019, *Fournier, J. GYJF19125* (HAST 147362); Maripasoula, Saül, edge of the forest next to Carbets du bord, 3.623598 N, 53.210338 W, disturbed mesophilic rainforest, on dead blackened wood, 19 Aug 2018, *Fournier, J. GYJF18019* (HAST 147363); Maripasoula, Saül, trail head to Roche-Bateau, Crique-Cochon, 3.625994 N, 53.1955 W, 210–220 m, disturbed mesophilic rainforest, on dead blackened trunk, 31 Mar 2021, *Fournier, J. GYJF21309* (HAST 147364); Maripasoula, Saül, edge of the village, around the remarkable fromager (*Ceiba pentandra*), 3.624963 N, 53.208523 W, disturbed secondary rainforest, on dead blackened log, 26 Aug 2018, *Fournier, J. GYJF18171* (HAST 147365), immature; Maripasoula, Saül, trail head to Roche-Bateau, 3.62056 N, 53.199899 W, ca. 240 m, disturbed mesophilic rainforest, on dead blackened trunk, 30 Mar 2021, *Fournier, J. GYJF21279* (HAST 147366).

**Known distribution**: Neotropical: French Guiana, Guatemala, Trinidad, Venezuela (Dennis 1956), México (San Martín and Rogers 1989).

**Comments**: *Xylaria guyanensis* is readily recognized in the field by its stature and its reticulately cracked surface with shallow cracks delineating wide, conspicuous ostiolar discs. The latter character also occurs in *X. adscendens* (Fr.) Fr., which differs by more slender stromata with an acute apex, smaller ostiolar discs, and smaller ascospores averaging 10.5 × 4.1 µm (Fournier et al. 2020). The development of the ostiolar discs, observable on immature stromata, is peculiar in that it results from the fragmentation of a superficial operculum raised by the swelling of an underlying layer of white tissue. This process is so far unknown in *Xylaria* and reminiscent of that seen in *Annulohypoxylon* Y.-M. Ju, J. D. Rogers & H.-M. Hsieh (Fournier and Lechat 2016; Fournier et al. 2016). In agreement with previous records (Table 19), the ascospores of *X. guyanensis* are shown to be fairly variable in shape and dimensions. An additional unreported character, unusual within *Xylaria* but also present in *X. grammica* (this paper), is the massive refractive content of the paraphyses.

As discussed by Dennis (1956) and San Martín and Rogers (1989), *X. poitei* Lév. is another species with massive stromata sharing similarities with *X. guyanensis*. It differs by its wider pantropical distribution, larger stromata initially coated with a white superficial pellicle, and lack of well-defined ostiolar discs.

**Fig. 31:**
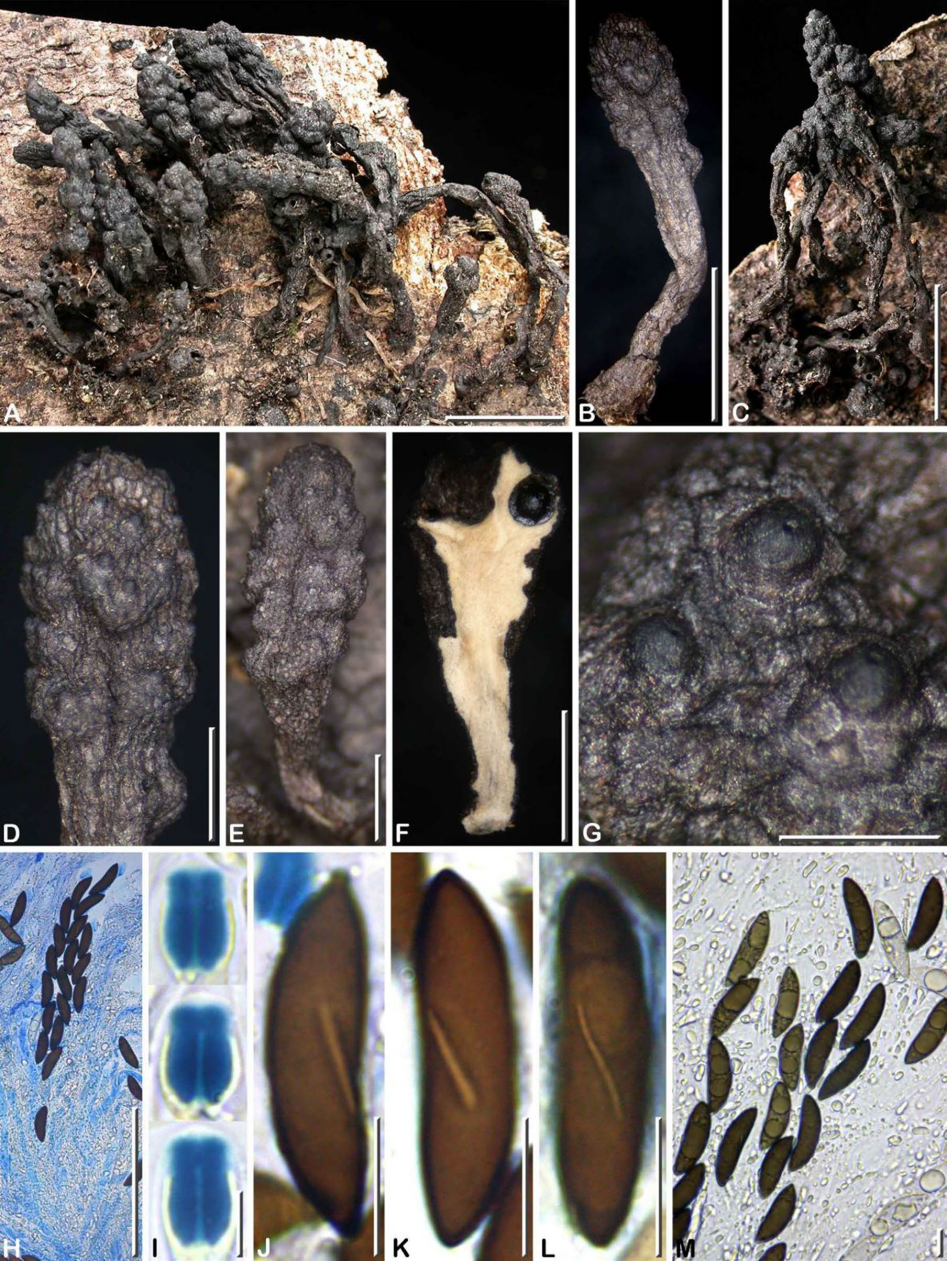
*Xylaria haemorrhoidalis* (**A–D, F–K, M ***GYJF21188*; **E, L ***GYJF21276*). **A, C** Clusters of prostrate stromata on bark. **B, D, E** Stromata in side view showing a nodulose and scaly surface and a pannose base in **B. F** Stroma in vertical section showing white solid internal tissue down to the base of stipe. **G** Stromatal surface in close-up showing dome-shaped ostioles and thick polygonal scales. **H** Bunch of asci in strongly guttulate hamathecium, in blue Pelikan ink. **I** Ascal apical apparati. **J–L** Ascospores in ventral view showing a germ slit (**I–L** in Melzer’s reagent). **M** Ascospores in the fresh state in guttulate hamathecium, in 1% SDS. Bars in **A, C** = 10 mm; **B** = 5 mm; **D–F** = 2 mm; **G** = 0.5 mm; **H** = 100 µm; **I** = 5 µm; **J–M** = 10 µm

***Xylaria haemorrhoidalis*** Berk. & Broome, J. Linn. Soc., Bot. 14: 117. 1873. Figure 31, Table 20

**Table 20:** Ascospore and ascal apical apparatus dimensions in two collections of *X. haemorrhoidalis* showing their variation range, compared with those reported for *X. globosa* from the French West Indies by Fournier et al. (2019) and two from French Guiana

Collection numbers	Ascospore measurements with extreme values in parentheses	Q = quotient l/w, *N* = number of measurements	Me = mean values of l/w, Qe = mean quotient l/w	Ascal apical apparatus h × w, *N* = 25
*GYJF21188*	(23.1–)24.7–27.6(−29.6) × (7.2–)7.5–9.2(−9.8) µm	Q = (2.6–)2.8–3.5(−3.9), *N* = 60	Me = 26.1 × 8.4 µm, Qe = 3.1	6.8–8.2 × 4.3–5.1 µm, Me = 7.5 × 4.7 µm
*GYJF21276*	(20.8–)23.2–26.0(−27.4) × (6.6–)7.0–8.2(−8.7) µm	Q = (2.6–)2.9–3.6(−3.9), *N* = 60	Me = 24.4 × 7.5 µm, Qe = 3.3	6.5–7.7 × 4.3–5.1 µm, Me = 7.1 × 4.7 µm
**Cumulated values**	**(20.8–)23.2–27.6(−29.6) × (6.6–)7.0–9.2(−9.8) µm**	**Q = (2.6–)2.8–3.6(−3.9), N = 120**	**Me = 25.2 × 7.95 µm, Qe = 3.2**	**6.5–7.7 × 4.3–5.1, Me = 7.3 × 4.7 µm**
*X. globosa* FWI (Fournier et al. 2018c)	(20.6–)21.2–30.2(−31.4) × (6.3–)6.7–9.3(−10.0) µm	Q = (2.4–)2.6–4.0(−4.5), *N* = 600	Me = 25.0 × 7.9 µm, Qe = 3.2	6.9–10.3 × 4.4–5.5 µm, Me = 8.4 × 5.0 µm
*X. globosa* FG *GYJF18069*	(20.2–)22.3–26.0(−27.7) × (6.8–)7.3–8.3(−8.6) µm	Q = (2.6–)2.8–3.5(−3.9), *N* = 60	Me = 24.2 × 7.8 µm, Qe = 3.1	6.1–6.9 × 4.0–4.8 µm, Me = 6.4 × 4.4 µm
*X. globosa* FG *GYJF21193*	(22.6–)24.6–28.1(−29.7) × (7.3–)7.7–8.9(−9.6) µm	Q = (2.6–2.8–3.5(−3.8), *N* = 60	Me = 26.3 × 8.3 µm, Qe = 3.2	

**Stromata** in small clusters, gregarious, superficial, upright to prostrate, simple, stipitate, 7–28 mm in total length, the fertile head 3–10 mm high × 2.5–4 mm diam, cylindrical to subclavate, apically obtusely rounded and fertile, strongly nodulose with half- to almost fully exposed perithecial contours; surface blackish, corky-cracked into rather small dark gray to blackish gray polygonal scales; subsurface leathery, ca. 40 µm thick; the stipes well-defined, 4–19 mm high, 0.7–1.2 mm wide, black, cracked, glabrous with a slightly swollen pannose base; interior whitish down to the stipe, solid. **Perithecia** subglobose, 0.5–0.8 mm diam. **Ostioles** conspicuous, dome-shaped to slightly pulvinate, dull black, 200–250 µm diam at base.

**Paraphyses** copious, hyphal, thin-walled, 6–8 µm wide at base, tapering to 1.5–2.0 µm wide above asci, discreetly embedded in mucilaginous material, conspicuously guttulate, oily guttules 2.5–4.0 µm wide, globose to oblong. **Asci** cylindrical, with (4–6–)8 overlapping uniseriately arranged ascospores, the spore-bearing parts 145–180 × 11–13 µm, the stipes 125–144 µm long, with apical apparatus slightly urn-shaped, apically flattened with a faint obtuse rim, 6.5–7.7 × 4.3–5.1 µm (Me = 7.3 × 4.7 µm, *N* = 50), strongly bluing in Melzer’s reagent. **Ascospores** (20.8–)23.2–27.6(−29.6) × (6.6–)7.0–9.2(−9.8) µm, Q = (2.6–)2.8–3.6(−3.9), *N* = 120 (Me = 25.2 × 8.0 µm, Qe = 3.2), fusiform-inequilateral to navicular in side view, frequently ventrally concave, with narrowly rounded, at times slightly pinched ends, medium to dark brown, unicellular, with a conspicuous, oblique, straight to slightly sigmoid germ slit on the flattened side, much less than spore-length, lacking appendages or mucilaginous sheath; epispore smooth.

**Asexual morph** on the natural substrate not observed.

**Specimens examined: FRENCH GUIANA.** Maripasoula, Saül, trail head to Sentier des Gros Arbres, 3.6201 N, 53.207989 W, ca. 210 m, disturbed secondary rainforest, on dead corticated trunk, 27 Mar 2021, *Fournier, J. GYJF21188* (HAST 147367); Maripasoula, Saül, trail head to Roche-Bateau, 3.62056 N, 53.199899 W, ca. 240 m, disturbed secondary rainforest, on dead corticated trunk, 30 Mar 2021, *Fournier, J. GYJF21276* (HAST 147368). **SRI LANKA.** Central and Southern Part, on wood, 1879, *Thwaites, G. H. K. 31* (holotype of *X. haemorrhoidalis* K(M) 144077 ex Berkeley herb.).

**Known distribution**: French Guiana, from two collections in the vicinity of Saül, Sri Lanka (Berkeley and Broome 1873), Taiwan (Hsieh et al. 2010).

**Comments:** The two collections documented above share strongly nodulose, leathery stromata with an obtusely rounded fertile apex, a corky-cracked surface, conspicuous ostioles, and a well-defined narrow stipe. This combination of features suggests affinity with members of the PO clade (Hsieh et al. 2010), supported by the presence of large, dark brown, fusiform-inequilateral to navicular ascospores with a short diagonal germ slit. Ascospore dimensions are identical to those of *X. globosa* (Table 20), suggesting that these specimens may represent an atypical form of that species, an interpretation consistent with the considerable morphological variability known in *X*. *globosa*.

However, a closer examination revealed several differences from typical *X. globosa*: the perithecial contours more prominently exposed; ostioles are dome-shaped rather than discoid; superficial scales are smaller; and the orange internal tissue at the stipe base, characteristic of *X*. *globosa*, is lacking. The ascospore germ slit also slightly differs, being less slanted, more oblique than diagonal, and the paraphyses contain a much more conspicuous oily content. In culture, the exudation droplets are clear to orangish rather orange-red to blood-red as in *X*. *globosa*. These features, together with the phylogenetic distance from *X. globosa* and other members of the PO clade indicated by our phylogram (Fig. 1), support their recognition as distinct.

Our phylogenetic analyses suggest the French Guiana material corresponds to *X. haemorrhoidalis*, a poorly documented species from Sri Lanka, described in the protologue as having subglobose to oblong, sessile and roughened stromata but with few additional details. We examined the holotype of *X. haemorrhoidalis*, and consider the two specimens cited herein to be conspecific with it.

**Fig. 32:**
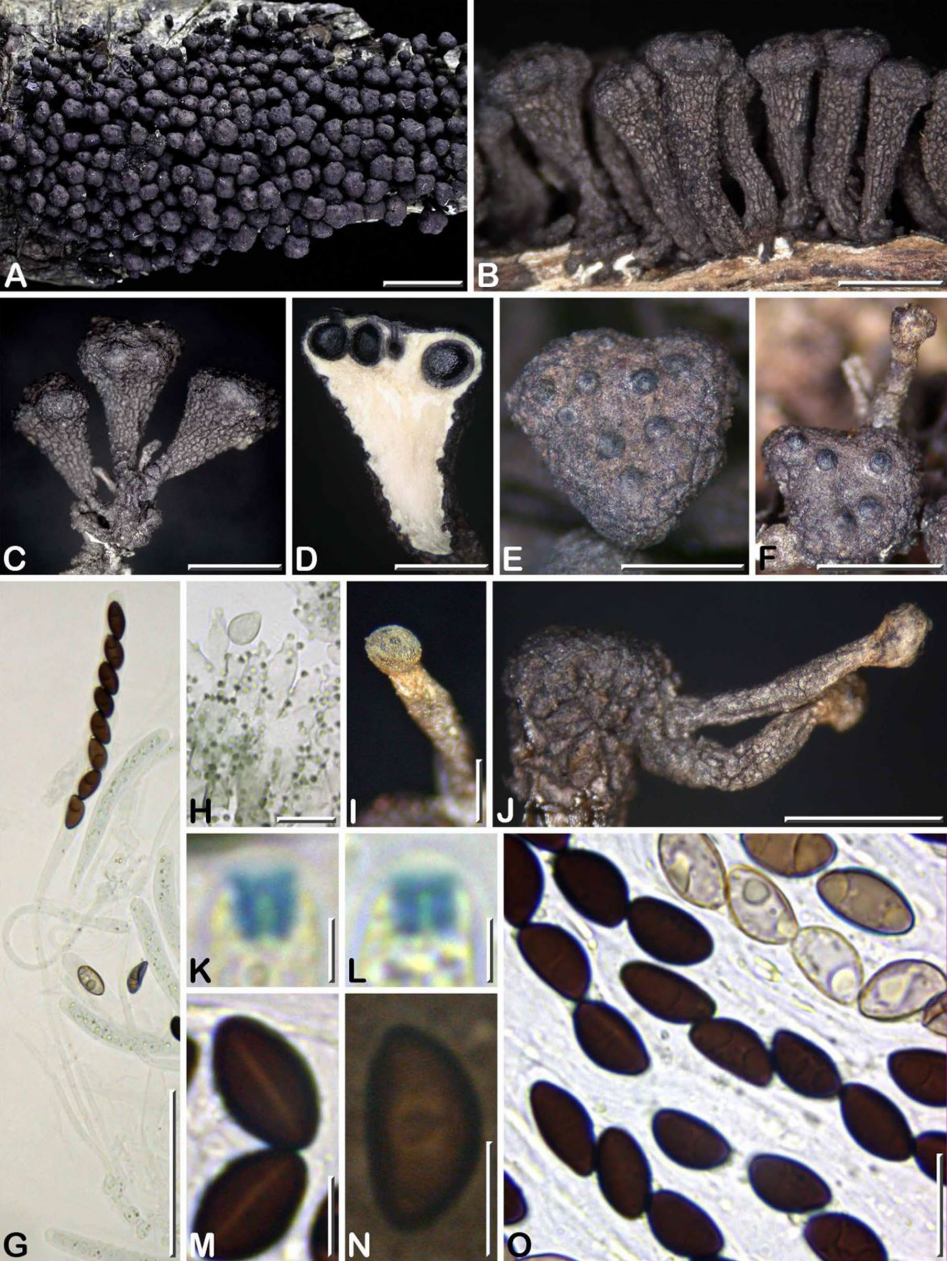
*Xylaria heliscus* (**A, B, O ***GYJF21096*; **C–E, G, K, M, N ***GYJF21268*; **F ***GYJF21117*; **H–J ***GYJF21098*; **L ***YZ07034*). **A** Densely aggregated stromata on bark in top view. **B** Stromata in side view. **C** Bunch of stromata originating from a ramified common base. **D** Stroma in vertical section showing a thin superficial crust and a white compact interior. **E** Fertile head in top view showing an almost smooth surface and large discoid ostioles. **F, J** Fertile heads showing lateral stipitate sterile outgrowths. **G** Long-stipitate ascus, in chlorazol black. **H** Conidiogenous cells and a conidium, in 1% SDS. **I** Conidioma with greenish fertile top. **K, L** Ascal apical apparati, in Melzer’s reagent. **M** Two ascospores in ventral view showing a germ slit, in 1% SDS. **N** Ascospore in India ink showing an absence of sheath or appendage. **O** Mature and immature ascospores in 1% SDS. Bars in **A** = 5 mm; **B, C** = 2 mm; **D–F, J** = 1 mm; **G** = 50 µm; **H, O** = 10 µm; **I** = 0.5 mm; **K, L** = 2 µm; **M, N** = 5 µm

***Xylaria heliscus*** (Mont.) J. D. Rogers & Y.-M. Ju, Mycotaxon 68: 370. 1998. Figure 32, Table 21

**Table 21:** Ascospore and ascal apical apparatus dimensions in four collections of *X. heliscus* from French Guiana and one from Hainan showing their variation range, compared with those reported in literature

Collection numbers	Ascospore measurements with extreme values in parentheses	Q = quotient l/w, *N* = number of measurements	Me = mean values of l/w, Qe = mean quotient l/w	Ascal apical apparatus h × w, *N* = 20
*GYJF12117* FG	(8.1–)8.5–9.9(−10.3) × (4.4–)4.5–5.4(−5.6) µm	Q = (1.6–)1.7–2.1(−2.2), *N* = 60	Me = 9.1 × 4.9 µm, Qe = 1.9	
*GYJF21096* FG	(8.1–)8.7–10.2(−11.2) × (4.5–)4.7–5.4(−5.8) µm	Q = (1.5–)1.6–2.1(−2.2), *N* = 60	Me = 9.3 × 5.1 µm, Qe = 1.8	1.9–2.3 × 1.5–2.0 µm, Me = 2.1 × 1.8 µm
*GYJF21098* FG	(8.2–)8.6–9.9(−10.4) × (4.3–)4.7–5.5(−5.7) µm	Q = (1.5–)1.6–2.0(−2.3), *N* = 60	Me = 9.2 × 5.1 µm, Qe = 1.8	
*GYJF21268* FG	(7.9–)8.6–9.7(−10.1) × (4.4–)4.7–5.5(−5.8) µm	Q = (1.5–)1.6–2.0(−2.3), *N* = 60	Me = 9.1 × 5.1 µm, Qe = 1.8	2.0–2.3 × 1.6–2.0 µm, Me = 2.1 × 1.8 µm
*ZY07034* Hainan	(8.4–)9.0–10.3(−10.8) × (4.5–)4.9–5.6(−5.9) µm	Q = (1.6–)1.7–2.0(−2.2), *N* = 60	Me = 9.6 × 5.2 µm, Qe = 1.8	1.7–2.1 × 1.6–1.8 µm, Me = 1.9 × 1.7 µm
**Cumulated values**	**(7.9–)8.5–10.3(−10.8) × (4.4–)4.5–5.6(−5.9) µm**	**Q = (1.5–)1.6–2.1(−2.3), N = 300**	**Me = 9.3 × 5.1 µm, Qe = 1.8**	**1.7–2.3 × 1.6–2.0 µm, Me = 2.0 × 1.8 µm**
Indonesia (Rogers et al. 1987)	9–10 × 5–6 µm		Me = 9.5 × 5.5 µm, Qe = 1.7	2.2 × 1.5 µm
México (San Martín and Rogers 1993b)	(8–)8.5–10(−11) × (4.5–)5–5.5 µm		Me = 9.5 × 5.3 µm, Qe = 1.8	
Brazil, FG, Malaysia & Philippines (Rogers and Ju 1998)	9–11 × (4.5–)5–6 µm		Me = 10.0 × 5.5 µm, Qe = 1.8	

≡ *Hypoxylon heliscus* Mont., Ann. Sci. Nat. Bot., sér. II, 13: 355. 1840.

≡ *Kretzschmaria heliscus* (Mont.) Massee, Bull. Misc. Inf., Kew no. 138: 118. 1898.

**Stromata** narrowly turbinate, upright to somewhat prostrate, loosely to densely gregarious, forming small to large colonies over several centimeters, 2.0–4.0 mm in total height, simple to most often connate at base by 2–3 or arising from a common intricate base comprised of black, contorted and ramified, fragile, cylindrical to flattened rhizomorphic structures; fertile heads flat-topped to slightly convex, 1.2–2.4 mm diam, 0.65–1.0 mm high, few-peritheciate, irregularly rounded, almost smooth on top, slightly corky-cracked at margin, with perithecial contours slightly exposed; the stipes narrowly obconical, 0.8–2.9 mm high, coarsely cracked into polygonal warts in upper part, ending into a narrow, weakly warty to smooth lower part 0.25–0.40 mm wide often difficult to distinguish from the basal rhizomorphs; upper surface blackish to blackish brown, glabrous, roughened by ostioles; subsurface a thin leathery crust 30–40 µm thick; interior solid, dense, white. **Perithecia** subglobose, 0.40–0.50 mm diam, fully immersed. **Ostioles** finely papillate, at the center of a black, flat to sometimes slightly raised disk 120–170 µm diam.

**Paraphyses** sparse, hyphal, remotely septate, thin-walled, 4.5–5.0 µm wide at base, tapering to 1.5–2 µm wide above asci, discreetly embedded in mucilaginous material. **Asci** cylindrical, with (6–)8 slightly overlapping uniseriately arranged ascospores, the spore-bearing parts 60–68 × 6.0–6.5 µm, the stipes 30–70 µm long, with apical apparatus quadrate in optical view, apically flattened with a faint lateral rim, 1.7–2.3 × 1.6–2.0 µm (Me = 2.0 × 1.8 µm, *N* = 60), bluing in Melzer’s reagent. **Ascospores** (7.9–)8.5–10.3(−10.8) × (4.4–)4.5–5.6(−5.9) µm, Q = (1.5–)1.6–2.1(−2.3), *N* = 300 (Me = 9.3 × 5.1 µm, Qe = 1.8), ellipsoid-inequilateral, with most often broadly rounded ends, ventrally flat to slightly convex, dark brown, unicellular, with a fairly conspicuous, straight, longitudinally oriented germ slit almost spore-length on the ventral side; lacking appendage or mucilaginous sheath; epispore smooth.

**Asexual morph** present on top of upright conidiomata with a swollen head, mixed with overmature stromata or from lateral outgrowths, forming a grayish green velvety layer with short greenish conidiogenous cells 3.7–5.0 µm diam featuring prominent, blackish green denticular secession scars; conidia broadly ellipsoid to ovoid, greenish, smooth-walled, 5.5–7.5 × 4.0–5.0 µm.

**Specimens examined: FRENCH GUIANA.** Roura, Cacao, Camp Bonaventure, 4.328545 N, 52.339082 W, 60–65 m, on dead corticated branch, 23 Mar 2021, *Fournier, J. GYJF21098* (HAST 147369); Roura, Cacao, Camp Bonaventure, 4.328545 N, 52.339082 W, 60–65 m, on dead corticated branch, 23 Mar 2021, *Fournier, J. GYJF21096* (HAST 147370); Régina, Nouragues natural reserve, Inselberg camp, trail to Pararé 0.5 km from the camp, 4.311240 N, 52.132789 W, ca. 300 m, hygrophilic rainforest, on dead corticated branch, 21 Jun 2012, *Fournier, J. GYJF12117* (HAST 147371), overmature; Maripasoula, Saül, trail head to Roche-Bateau, 3.62056 N, 53.199899 W, ca. 240 m, disturbed mesophilic rainforest, on dead corticated trunk, 30 Mar 2021, *Fournier, J. GYJF21268* (HAST 147372).

**Other specimen examined: CHINA.** Hainan, Wuzhi mountain, ca 700 m, hygrophilic rainforest, on a dead corticated branch, 7 Jul 2007, *Zhang, Y. YZ07034* (JF).

**Known distribution**: Pantropical: Brazil, French Guiana, Malaysia, Philippines (Rogers and Ju 1998); China (Hainan) (this paper); México (San Martín and Rogers 1993a); Indonesia (North Sulawesi) (Rogers et al. 1987).

**Comments**: *Xylaria heliscus* was described from French Guiana by Montagne (1840) as *Hypoxylon heliscus* before being accommodated successively under various names in the *Xylaria*ceous genera *Poronia* Willd., *Rhopalopsis* Cooke, *Kretzschmaria* Fr., and eventually assigned to *Xylaria* by Rogers and Ju (1998). The considerable morphological variation observed among the five collections reported and illustrated here likely account for the hectic taxonomic history of this species.

Among our four Guianese collections, two produce stromata that arise directly and separately from the bark, while the others bear stromata that are more loosely distributed and often arise from an intricate rhizomorphic structure. The two latter collections connected to a rhizomorphic network are largely overmature and feature vertical to lateral, often sterile extensions that could represent conidiomata. Because the microscopic characteristics of the four Guianese collections are identical (Table 21), these variations in stromatal configuration are not considered taxonomically significant but are more likely attributable to environmental factors.

In contrast, the collection from Hainan deviates by having slightly larger ascospores, stromata densely clothed in reddish-brown hairs, and perithecia embedded within a subsurface layer of yellowish tissue, rendering its identification as *X. heliscus* questionable. Collections deviating from typical *X. heliscus* by a hairy coating and slightly larger ascospores were reported as *X*. cf. *heliscus* by San Martín and Rogers (1993a) from México and by Ju and Rogers (1999) from Taiwan, suggesting that a complex of closely related species remains to be clarified.

**Fig. 33:**
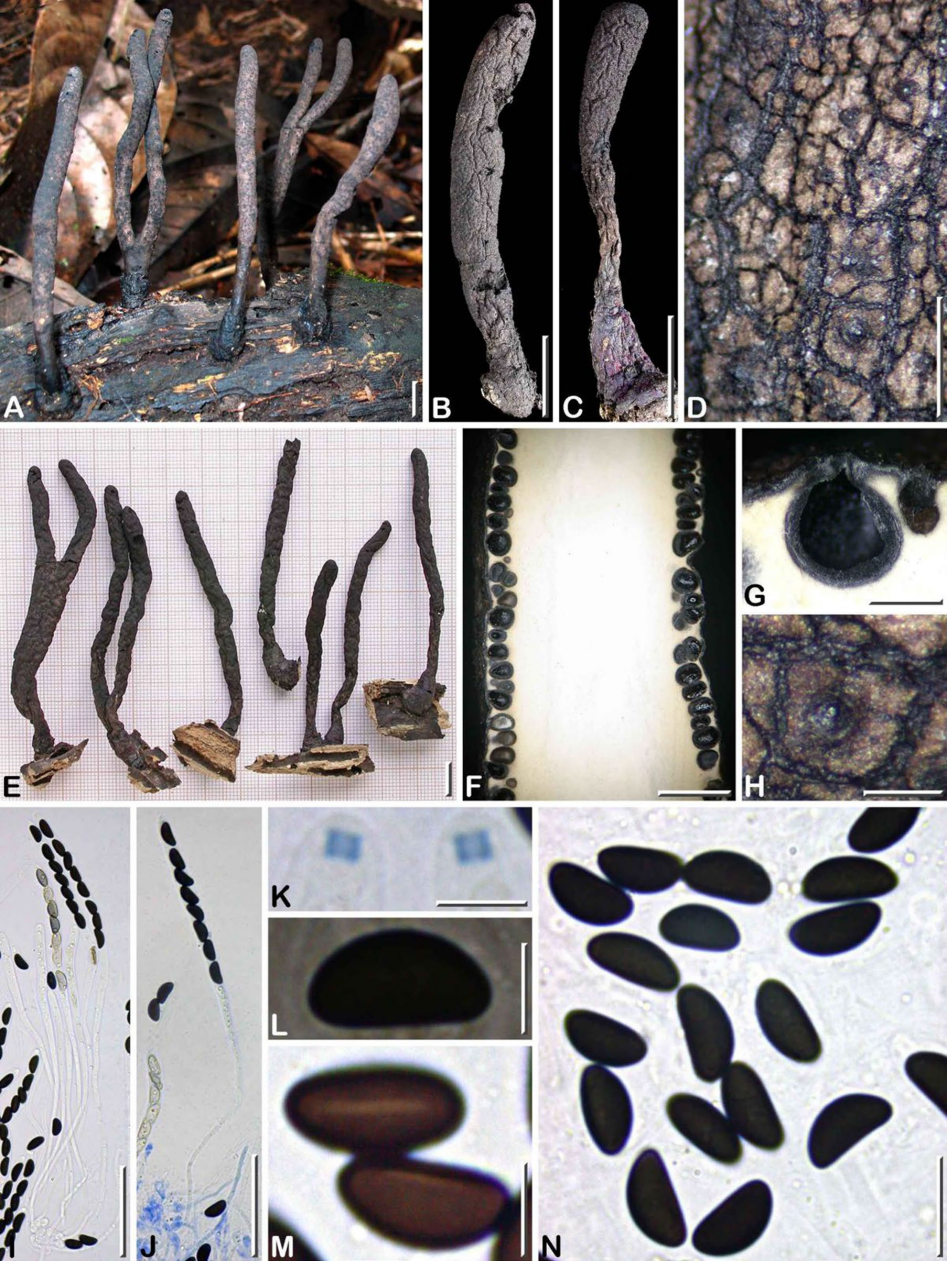
*Xylaria holmbergii* (**A, D–N ***GYJF21257* holotype; **B ***GYJF21370-2*; **C ***GYJF21138*). **A** Habit of stromata in situ in the fresh state. **B, C, E** Habit of stromata in the dry state. **D** Stromatal surface in close-up showing a reticulately-cracked outermost brown layer and ostioles. **F** Stroma in vertical section showing densely distributed perithecia and solid white interior. **G** Perithecium in vertical section, immersed under a thin leathery crust and opening through a prominent ostiole. **H** Stromatal surface in close-up showing an ostiole emerging through a polygonal scale. **I** Bunch of immature and mature long-stipitate asci, in 1% SDS. **J** Mature ascus in blue Pelikan ink. **K** Ascal apical apparati in Melzer’s reagent. **L** Ascospore in side view in India ink showing an absence of sheath or appendage. **M** Barely mature ascospores in ventral view showing short germ slits. **N** Variously shaped ascospores in side view (**M** and **N** in 1% SDS). Bars in **A–C, E** = 10 mm; **D, G** = 0.5 mm; **F** = 2 mm; **H** = 0.2 mm; **I, J** = 50 µm; **K–M** = 5 µm; **N** = 10 µm

***Xylaria holmbergii*** Speg., An. Mus. Nac. Buenos Aires 6: 261. 1898, as “*holmbergi*.” Figure 33, Table 22

**Table 22:** Ascospore dimensions in four collections of *X. holmbergii* showing a narrow range of intraspecific variations, compared with those of closely related species

Collection numbers	Ascospore measurements with extreme values in parentheses	Q = quotient l/w, *N* = number of measurements	Me = mean values of l/w, Qe = mean quotient l/w
*GYJF21257*	(8.0–)8.2–9.5(−10.2) × (4.1–)4.3–4.7(−4.9) µm	Q = (1.7–)1.8–2.2(−2.4), *N* = 60	Me = 8.9 × 4.5 µm, Qe = 2.0
*GYJF21138*	(7.9–)8.2–9.4(−10.0) × (3.7–)4.0–4.7(−5.2) µm	Q = (1.7–)1.8–2.2(−2.6), *N* = 60	Me = 8.8 × 4.4 µm, Qe = 2.0
*GYJF21286*	(8.1–)8.3–9.4(−10.2) × (4.0–)4.3–5.1(−5.5) µm	Q = (1.5–)1.7–2.1(−2.2), *N* = 60	Me = 8.8 × 4.6 µm, Qe = 1.9
*GYJF21370-2*	(8.4–)8.7–9.7(−10.2) × (4.1–)4.3–5.0(−5.2) µm	Q = (1.7–)1.8–2.1(−2.4), *N* = 60	Me = 9.2 × 4.7 µm, Qe = 2.0
**Cumulated values**	**(7.9–)8.2–9.7(−10.2) × (3.7–)4.0–5.1(−5.5) µm**	**Q = (1.5–)1.7–2.2(−2.6), N = 240**	**Me = 8.9 × 4.5 µm, Qe = 2.0**
*X. corniformis* (Fournier 2014)	8.8–9.8(−10.0) × (4.4–)4.5–5.0(−5.4) μm	Q = (1.7)1.8–2.1(2.2), *N* = 36	Me = 9.2 × 4.8 μm, Qe = 1.9
*X. ellisii* (Ibrahim et al. 2020)	(8−)9–9.5(−10) × (4.5−)5–5.5(−6) μm		Me = 9.25 × 5.25 µm, Qe = 1.8
*X. confragosa* (this paper)	(6.8–)7.3–8.1(−8.6) × (3.6–)3.8–4.5(−5.0) µm	Q = (1.5–)1.7–2(−2.2), *N* = 60	Me = 7.7 × 4.1 µm, Qe = 1.9

= *Xylaria violaceopannosa* Starb., Bih. Kongl. Svenska Vetensk.-Akad. Handl. 27, Afd. III (9): 24. 1901.

**Stromata** upright, loosely clustered, separate or connate at base by two, narrowly cylindrical to slightly clavate or slightly fusiform, simple, straight to slightly curved, with broadly rounded fertile apices, wrinkled in the dry state but not split, stipitate, 42–80 mm in total height, 5–9 mm wide; surface uniformly dull copper-brown, lacking exposed perithecial contours, reticulately cracked into minute polygonal scales 0.30–0.45 mm diam, separated by black shallow cracks; subsurface a black leathery crust 40–60(−85) µm thick; interior white to cream-colored, solid, densely fibrous, shrinking upon drying but not turning hollow; the stipes ill-defined, 8–28 mm high, concolorous in upper half, smooth, glabrous apart from a blackish conspicuously swollen pannose base of a dense, brown, occasionally superficially purple tomentum. **Perithecia** crowded, subglobose to laterally flattened, 0.45–0.75 mm diam. **Ostioles** prominent, obtusely conic-papillate, frequently apically truncate, dull to shiny black, 100–150 µm diam at base.

**Paraphyses** copious, hyphal, hyaline, remotely septate, 4.0–4.5 µm wide at base, tapering to 0.8–1.5 µm wide above asci, discreetly embedded in mucilaginous material. **Asci** cylindrical, long-stipitate, with (6–)8 uniseriately arranged, slightly overlapping ascospores, the spore-bearing parts 60–78 × 5.0–6.5 µm, the stipes 90–120 µm long, with apical apparatus 1.4–2.0 × 1.7–2.0 µm (Me = 1.7 × 1.8 µm, *N* = 40), short-cylindrical with a fait upper rim, cuboid in outline, slightly wider than high, bluing in Melzer’s reagent. **Ascospores** (7.9–)8.2–9.7(−10.2) × (3.7–)4.0–5.1(−5.5) µm, Q = (1.5–)1.7–2.2(−2.6), *N* = 240 (Me = 8.9 × 4.5 µm, Qe = 2.0), ellipsoid-inequilateral with broadly rounded ends, ventrally flat to slightly concave, occasionally slightly reniform, blackish brown, with an inconspicuous, straight, longitudinally oriented short germ slit 4.0–5.5 µm long on the ventral side; lacking appendages or mucilaginous sheath; epispore smooth.

**Asexual morph** on the natural substrate not observed.

**Specimens examined**: **ARGENTINA.** Misiones, on wood, 1884, *Holmberg, C. L.* (holotype of *X. holmbergii* LPS 3310). **FRENCH GUIANA.** Maripasoula, Saül, shortcut to the airfield, 3.622852 N, 53.205768 W, 210–220 m, disturbed secondary rainforest, on dead blackened wood, 29 Mar 2021, *Fournier, J. GYJF21257* (HAST 147509); Maripasoula, Saül, trail head to Sentier des Gros Arbres, 3.620839 N, 53.208536 W, ca. 210 m, disturbed mesophilic rainforest, on dead blackened wood, 26 Mar 2021, *Fournier, J. GYJF21138* (HAST 147508); Maripasoula, Saül, trail head to Sentier Roche-Bateau, 3.62056 N, 53.199899 W, ca. 240 m, disturbed secondary rainforest, on dead blackened wood, 30 Mar 2021, *Fournier, J. & Thonnel, A. GYJF21286* (HAST 147510); Maripasoula, Saül, trail to Monts-La-Fumée, 3.634796 N, 53.205457 W, ca. 270 m, disturbed mesophilic rainforest, on dead corticated branch, 2 Apr 2021, *Fournier, J. GYJF21370-2* (HAST 147511); near Cayenne, on wood, *Leprieur, F. R. 1221*, as *X. corniformis* (PC ex Montagne herb. 10102). **PARAGUAY.** San Antonio pr. Asuncion, on wood, 20 Jul 1893, *Lindman, E. 385B* (holotype of *X. violaceopannosa* S F43668).

**Known distribution**: Argentina, Brazil, French Guiana, Paraguay.

**Comments**: Our collections of *X. holmbergii* documented herein are characterized by tall, subcylindrical to clavate or fusiform stromata with an obtusely rounded fertile apex, an orange-brown to dull brown reticulately cracked surface, a thin leathery subsurface, and an ill-defined stipe with a thick pannose base. Their small blackish ascospores with broadly rounded ends are similar to those of *X. corniformis* (Ju et al. 2009; Fournier 2014) and *X. ellisii* J. B. Tanney, Seifert & Y.-M. Ju (Ibrahim et al. 2020), but differ by the presence of a shorter germ slit.

*Xylaria holmbergii* can be distinguished from *X. crassipes* (this paper), which has larger ascospores 12.2–13.8 × 5.3–6.3 µm with a germ slit almost spore-length. *Xylaria robusta* (this paper) features tall, branched, subcylindrical stromata and differs mainly by a brownish black, finely grooved surface lacking scales, a thick carbonaceous crust, and ascospores averaging 13.6 × 5.5 µm with narrowly rounded to acute ends. *Xylaria confragosa* (this paper) is phylogenetically and morphologically close to the *X. corniformis* complex but differs by smaller ascospores averaging 7.7 × 4.1 µm with a polar secondary appendage.

This species is strongly reminiscent of the temperate species *X. corniformis* (Fr.) Fr. by its finely reticulately cracked surface and small blackish brown ascospores but differs by its tropical occurrence and the presence of a conspicuous pannose base occasionally showing a purple tone. However, their morphological similarity is not supported by our phylogenetic analyses.

*Xylaria corniformis* as identified and illustrated by Theissen (1909a, Plate IX-5) corresponds well to *X. holmbergii*. Hamme and Guerrero (1994), who examined material identified by Rick, considered *X. violaceopannosa* to be a possible synonym of *X. holmbergii*. Their suspicion is confirmed by our study of the type specimens of *X*. *holmbergii* and *X*. *violaceopannosa*. Our Guianese collections also fit well the description of *X*. cf. *holmbergii* provided by San Martín (1992), but deviate by their leathery texture.

**Fig. 34:**
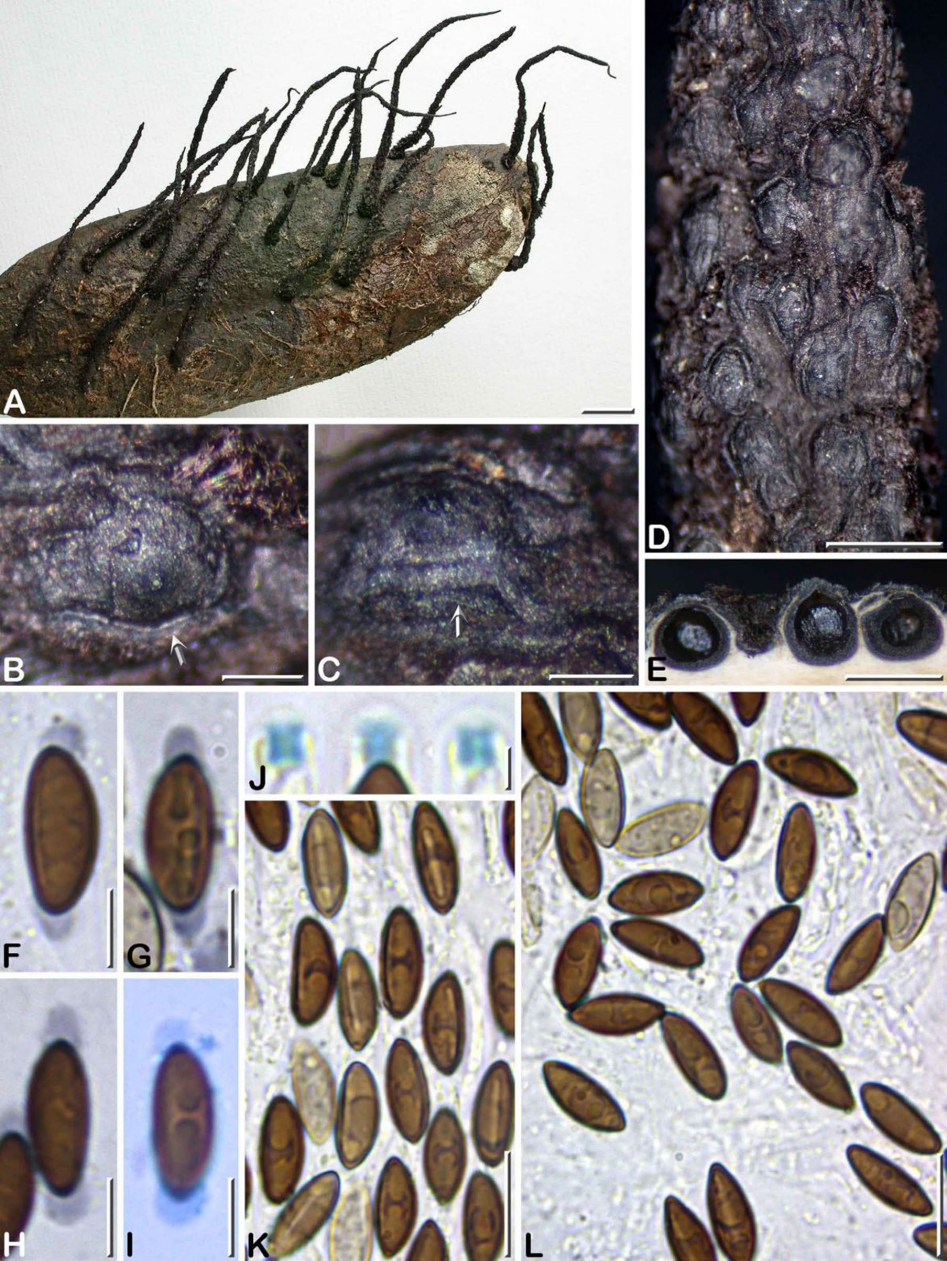
*Xylaria ianthinovelutina* (*GYJF19175*). **A** Habit of stromata on a woody pod of *Amphilophium paniculatum*. **B, C** Stromatal surface in close-up showing slightly exposed perithecial contours, finely papillate ostioles and blackish superficial stripes (arrows). **D** Stromatal surface roughened by perithecial mounds, striped outer layer and scattered tufts of reddish brown tomentum. **E** Stroma in longitudinal section showing perithecia immersed beneath a thin crust and a whitish solid interior. **F–H** Ascospores showing bipolar appendages, in aqueous nigrosin. **I** Ascospore showing bipolar appendages, in blue Pelikan ink. **J** Ascal apical apparati, in Melzer’s reagent. **K** Ascospores mostly in ventral view showing germ slits of variable length. **L** Variously oriented ascospores (**K** and **L** in 1% SDS). Bars in **A** = 10 mm; **B, C** = 0.2 mm; **D** = 1 mm; **E** = 0.5 mm; **F–I** = 5 µm; **J** = 2 µm; **K, L** = 10 µm

***Xylaria ianthinovelutina*** (Mont.) Fr., Nov. Act. Reg. Soc. Sci. Upsal., ser. III, 1: 128. 1851. Figure 34, Table 23

**Table 23:** Ascospore dimensions in the above collection of *X. ianthinovelutina* from French Guiana, compared with those previously reported in literature

Collection numbers	Ascospore measurements with extreme values in parentheses	Q = quotient l/w, *N* = number of measurements	Me = mean values of l/w, Qe = mean quotient l/w
*GYJF19175* FG	(8.9–)9.2–10.5(−11.3) × (3.6–)3.7–4.3(−4.5) µm	Q = (2.2–)2.3–2.8(−3.0), *N* = 60	Me = 10.0 × 4.0 µm, Qe = 2.5
Tropical America (Dennis 1956)	10–13 × 4–6 µm		Me = 11.5 × 5 µm, Qe = 2.3
Tropical Africa (Dennis 1958)	8–13 × (3–)4–6 µm		Me = 10.5 × 4.5 µm, Qe = 2.6
México (San Martín and Rogers 1989)	10–12(−13) × 4–5 µm		Me = 11.0 × 4.5 µm, Qe = 2.45
Various countries (Ju et al. 2018)	(9–)9.5–11(−12) × (3.5–)4–4.5(−5) µm	*N* = 60	Me = 10.3 × 4.3 µm, Qe = 2.6
FWI (Fournier et al. 2020)	(7.5–)9.0–11.3(−12.6) × (3.4–)3.7–4.7(−5.1) µm	Q = (1.9–)2.1–2.8(−3.2), *N* = 480	Me = 10.1 × 4.2 µm, Qe = 2.4

≡ *Hypoxylon ianthinovelutinum* Mont., Ann. Sci. Nat. Bot., sér. II, 13: 348. 1840, as “*xanthinovelutinum*.”

**Stromata** loosely gregarious, upright, separate, narrowly cylindrical, appearing wider at base owing to thick tomentum, simple, terete, straight to slightly curved, with apiculate to filiform sterile apices, stipitate; 23–55 mm in total height, the fertile head 13–22 mm high × 1.2–1.9 mm diam, the stipe 8–30 mm high, up to 3.5 mm diam; surface brownish black, nodulose with perithecial contours moderately to strongly exposed emerging from dense tufts of golden to purplish brown tomentum, overlain by a blackish outer layer splitting longitudinally into wide persistent stripes bearing the tomentum; the stipes ill-defined, black, flattened, longitudinally puckered, entirely densely tomentose, the base slightly swollen; subsurface crust leathery, 35–50 µm thick; interior solid, whitish. **Perithecia** subglobose to depressed-spherical, 0.35–0.45 mm diam. **Ostioles** finely papillate, dull to shiny black, frequently on a discoid, occasionally faintly raised base 150–170 µm diam.

**Paraphyses** hyphal, filiform, remotely septate, thin-walled, faintly guttulate, 5.5–6.8 µm wide at base, 1.0–1.5 µm wide at apex, embedded in mucilaginous material. **Asci** cylindrical, with (6–)8 slightly overlapping uniseriately arranged ascospores, the spore-bearing parts 70–80 × 4.8–5.5 µm, the stipes 52–65 µm long, with apical apparatus 1.6–2.0 × 1.3–1.5 µm (Me = 1.8 × 1.4 µm, *N* = 20), short-cylindrical with a faint upper rim, bluing in Melzer’s reagent. **Ascospores** (8.9–)9.2–10.5(−11.3) × (3.6–)3.7–4.3(−4.5) µm, Q = (2.2–)2.3–2.8(−3.0), *N* = 60 (Me = 10.0 × 4.0 µm, Qe = 2.5), ellipsoid to fusiform, slightly inequilateral, with mostly narrowly rounded to subacute ends, brown, unicellular, with a conspicuous longitudinally oriented germ slit 4.5–7.7 (Me = 6.1 µm long on average, *N* = 25) on the ventral side, with a thin mucilaginous coating swelling at both ends to form subglobose secondary appendages barely visible in water, stained by blue Pelikan ink or aqueous nigrosin; epispore smooth.

**Asexual morph** on the natural substrate not observed. Culture characteristics on OMA were described by Ju and Rogers (1999) based on material from Taiwan.

**Specimen examined**: **FRENCH GUIANA.** vicinity of Cayenne, on fallen fruits of *Hymenaea courbaril* (Fabaceae), Feb 1839, *Leprieur, F. R. 574* (holotype of *Hypoxylon ianthinovelutinum* PC ex Montagne herb., isotypes K(M) 156833 ex Berkeley herb., UPS F-176036 ex Fries herb.); Maripasoula, Saül, trail head to Roche-Bateau, 3.620498 N, 53.199309 W, disturbed secondary rainforest, on dead woody fruit of the liana *Amphilophium paniculatum* (Bignoniaceae) in the leaf litter, 22 Jun 2019, *Corriol, G. GYJF19175* (HAST 147373).

**Known distribution**: Pantropical.

**Comments**: The type material was collected from French Guiana, and the recent collection *GYJF19175* conforms in all respects to *X. ianthinovelutina* as described by Dennis (1956, 1958),Ju et al. (2018), San Martín and Rogers (1989), and Fournier et al. (2020). Stromatal branching appears variable, but the combination of mucronate to filiform apices, a densely tomentose and nodulose surface, ascospores 10–11 × 4–5 µm with narrowly rounded ends, a slightly less than spore-length germ slit, and bipolar secondary appendages characterizes this species. Although most often reported from fabaceous hosts, *X. ianthinovelutina* can also be encountered on woody fruits of tropical trees or lianas belonging to various families.

**Fig. 35:**
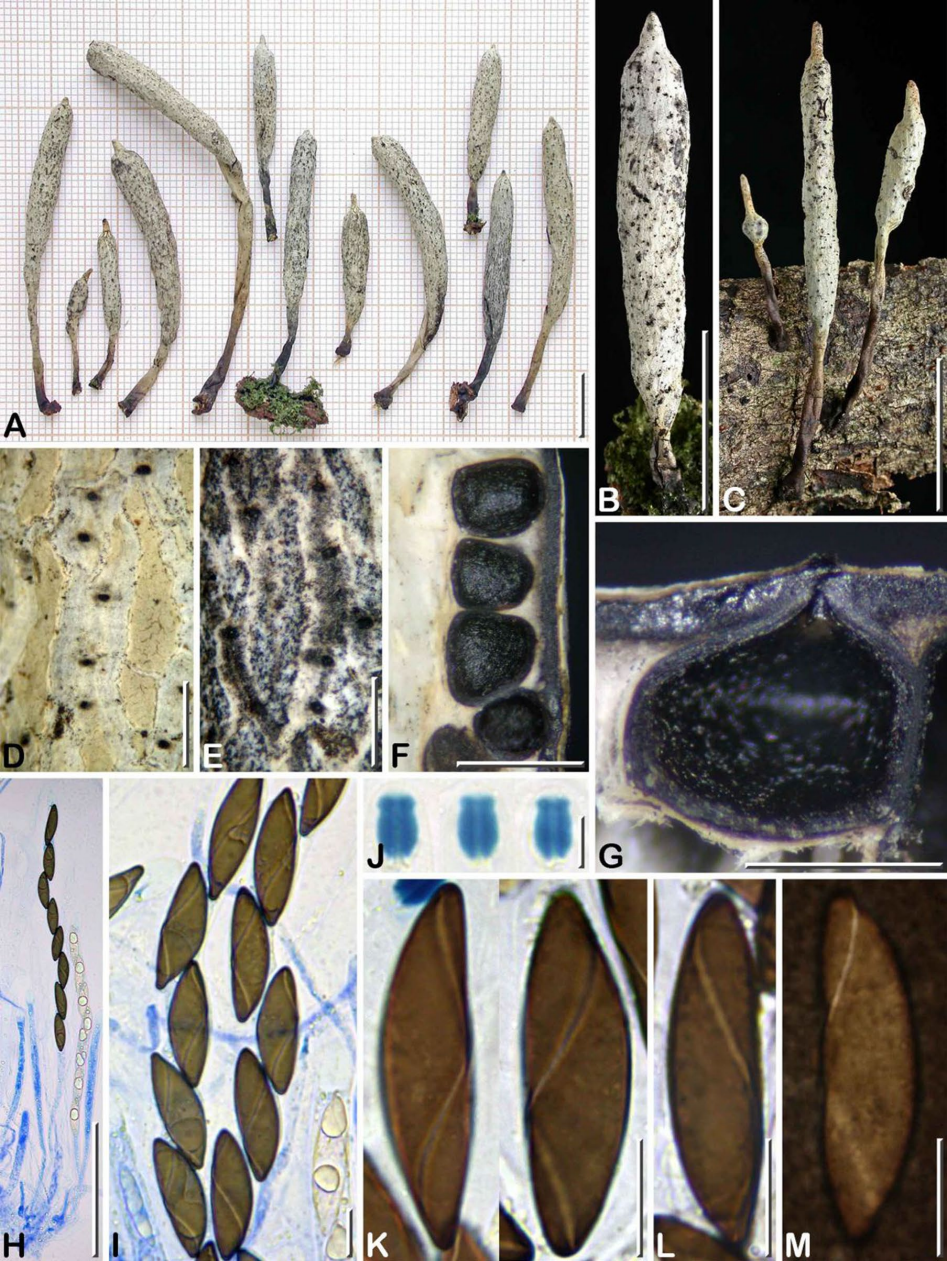
*Xylaria kegeliana* (**A, B, D–M ***GYJF21164*; **C ***GYJF21398*). **A–C** Variously shaped stromata. **D** Surface of a mature stroma showing ostioles in a linear row. **E** Surface of a mature stroma having lost its whitish outer layer, showing ostioles and black granulations. **F** Stromatal wall in longitudinal section. **G** Perithecium in vertical section showing an ostiole piercing the carbonaceous crust and the superficial whitish outer layer. **H** Immature and mature asci, in blue Pelikan ink. **I** Ascospores and paraphyses, in blue Pelikan ink. **J** Ascal apical apparati in Melzer’s reagent. **K–M** Ascospores in side view showing germ slits, respectively in Melzer’s reagent, 1% SDS and India ink. Bars in **A–C** = 10 mm; **D–F** = 1 mm; **G** = 0.5 mm; **H** = 100 µm; **I, K–M** = 10 µm; **J** = 5 µm

***Xylaria kegeliana*** (Lév.) Fr., Nov. Act. Reg. Soc. Sci. Upsal., ser. III, 1: 125. 1851. Figure 35, Table 24

**Table 24:** Ascospore dimensions in two collections of *X. kegeliana* showing a narrow range of intraspecific variations, compared with those reported in literature

Collection numbers	Ascospore measurements with extreme values in parentheses	Q = quotient l/w, *N* = number of measurements	Me = mean values of l/w, Qe = mean quotient l/w
*GYJF21164*	(26.3–)27.4–30.7(−32.1) × (5.9–)6.4–7.4(−7.9) µm	Q = (3.5–)3.8–4.6(−5.2), *N* = 60	Me = 28.9 × 6.9 µm, Qe = 4.2
*GYJF21398*	(24.3–)26.6–31.0(−33.7) × (6.3–)6.9–8.0(−8.4) µm	Q = (3.2–)3.4–4.3(−5.0), *N* = 60	Me = 28.7 × 7.4 µm, Qe = 3.9
**Cumulated values**	**(24.3–)26.6–31.0(−33.7) × (5.9–)6.4–8.0(−8.4) µm**	**Q = (3.2–)3.4–4.6(−5.2), N = 120**	**Me = 28.8 × 7.2 µm, Qe = 4.0**
Surinam holotype (Dennis 1956)	25–30 × 6–8.5 µm		Me = 27.5 × 7.3 µm, Qe = 3.8
Surinam holotype (Joly 1968)	24–34 × 6–9.5 µm		Me = 28.3 × 7.0 µm, Qe = 4.0
México (San Martín and Rogers 1989)	(27–)28–33.5(−35) × 6–7.5(−8) µm		Me = 30.1 × 6.8 µm, Qe = 4.6

≡ *Sphaeria kegeliana* Lév., Ann. Sci. Nat. Bot., sér. III, 5: 256. 1846.

**Stromata** upright, scattered, arising singly or in small groups, (12–)19–57 mm in total height, the fertile head cylindrical to narrowly clavate, straight to curved, (1.5–)3–10 mm wide, with apiculate sterile apices, hard-textured, rarely splitting lengthwise upon drying; surface yellowish white, more yellowish in the dry state, gradually slightly blackening with age, even, lacking exposed perithecial contours, with a thin, appressed, cream-colored outer layer splitting into wide elongated stripes, concealing minute black granulations showing as they wear off; black ostioles are distributed between these stripes, either randomly or in short linear rows; subsurface crust carbonaceous, 120–150 µm thick; interior white to slightly yellowish, fibrous between perithecia, compact, somewhat disintegrating and becoming hollow at center with age; the stipes mostly well-defined, (5–)12–38 mm high, 0.8–2.5 mm wide, cylindrical, glabrous, whitish to light reddish brown, blackened and enlarged at base. **Perithecia** subglobose to depressed-spherical, 0.6–0.9 mm diam. **Ostioles** obtusely papillate to hemispherical, black, 80–120 µm diam at base, occasionally appearing distributed in linear rows.

**Paraphyses** hyphal, hyaline, rarely septate, simple, 6.5–9.5 µm wide at base, tapering to 1.5–2.0 µm wide above asci, minutely guttulate, discreetly embedded in mucilaginous material. **Asci** cylindrical, with (6–)8 uniseriately arranged, slightly overlapping ascospores, the spore-bearing parts 180–200 µm long × 9.0–11.5 µm wide, the stipes 95–125 µm long, with apical apparatus narrowly urn-shaped, attenuated at base, apically flat with an obtuse rim, 5.4–6.3 × 3.4–3.9 µm (Me = 5.9 × 3.7 µm, *N* = 25), bluing in Melzer’s reagent. **Ascospores** (24.3–)26.6–31(−33.7) × (5.9–)6.4–8.0(−8.4) µm, Q = (3.2–)3.4–4.6(−5.2), *N* = 120 (Me = 28.8 × 7.2 µm, Qe = 4.0), fusiform-inequilateral with narrowly rounded, occasionally slightly pinched ends, ventrally flat to slightly convex, dark brown, unicellular, with a conspicuous spiraling germ slit on the ventral side extending almost to the ends; lacking sheath or appendages; epispore smooth.

**Asexual morph** on the natural substrate not observed.

**Specimens examined**: **FRENCH GUIANA.** Maripasoula, Saül, trail head of Sentier des Gros Arbres, 3.6201 N, 53.207989 W, ca. 210 m, disturbed secondary rainforest, on dead corticated branch above the soil, 3 Apr 2021, *Fournier, J. GYJF21398* (HAST 147374); Maripasoula, Saül, trail head of Sentier des Gros Arbres, 3.6201 N, 53.207989 W, ca. 210 m, disturbed secondary rainforest, on dead moss-covered liana above the soil, 27 Mar 2021, *Fournier, J. GYJF21164* (HAST 147375). **SURINAME.** Paramaribo, on wood, Jun 1844, *Kegel 575* (holotype of *Sphaeria kegeliana* PC 0096745, isotype K(M)).

**Known distribution**: Surinam (Dennis 1956; Joly 1968), México (San Martín and Rogers 1989), French Guiana (this paper).

**Comments**: *Xylaria kegeliana* is characterized by whitish, cylindrical, hard-textured, apiculate stromata and fusiform ascospores 26.6–31.0 × 6.4–8.0 µm, with a long spiraling germ slit. Its presumed affinities with *X. dealbata* and *X. grammica* were discussed by Dennis (1956) and San Martín and Rogers (1989). *Xylaria kegeliana* is a distinctive and rarely reported species, here recorded from French Guiana for the first time. The holotype was collected in Suriname, which belongs to the same general biogeographical region as French Guiana.

**Fig. 36:**
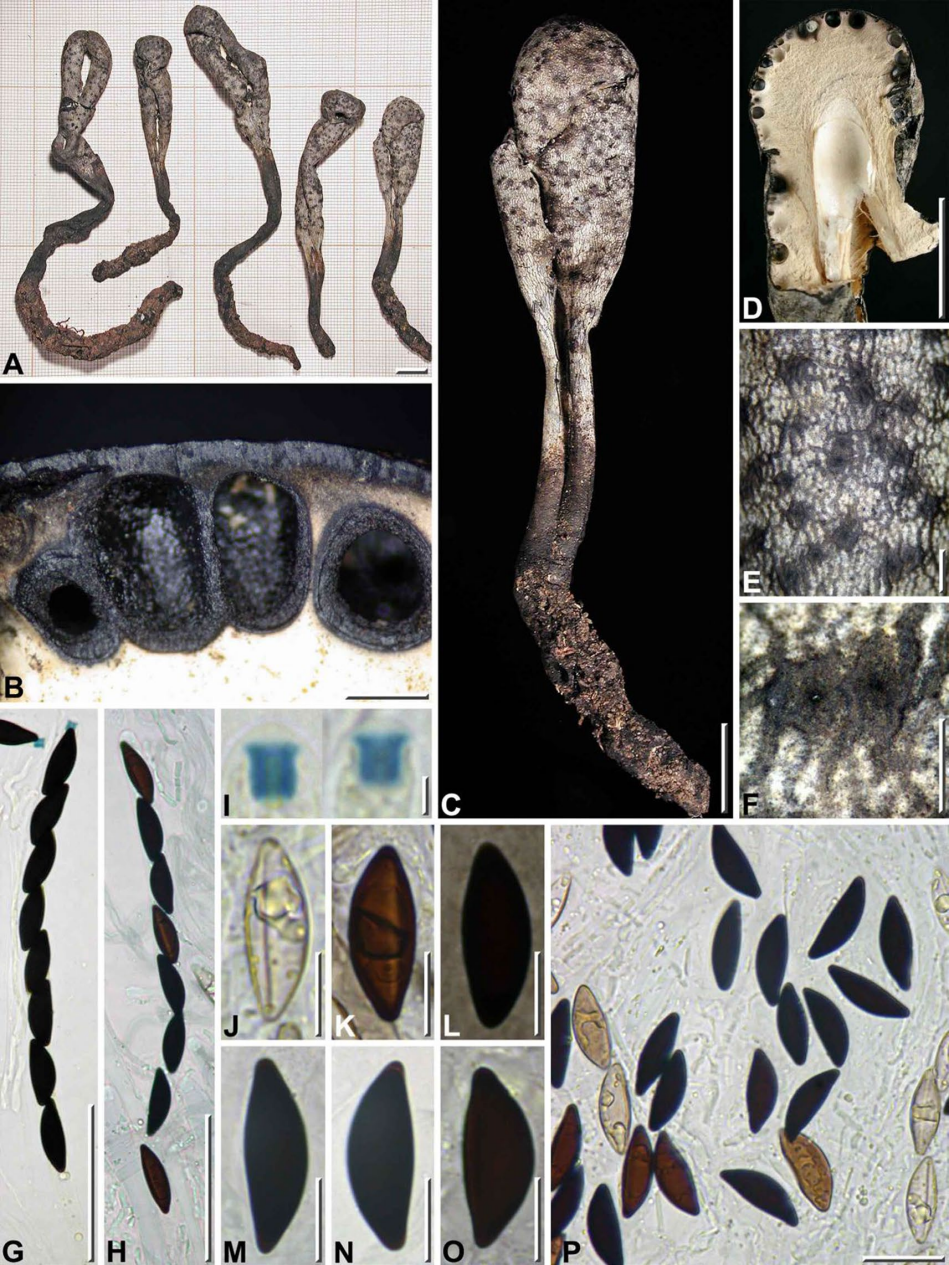
*Xylaria macouriana* (*CLL8095* holotype). **A** Habit of stromata in the dry state, arising from reddish brown rooting stipes. **B** Vertical section of stromatal apex showing perithecia beneath a thick carbonaceous crust. **C** Longitudinally split stroma with blackish stipe and a rooting stipe incrusted with soil particles. **D** Vertical section of stromatal apex showing a solid cream-white interior with center turning hollow. **E** Stromatal surface showing a network of low blackish ridges joining black ostiolar areas over a white background. **F** Two adjacent ostiolar areas in close-up showing minute ostioles. **G, H** Spore-bearing parts of mature asci, in Melzer’s reagent and chlorazol black respectively. **I** Ascal apical apparati in Melzer’s reagent. **J** Immature subhyaline ascospore showing a straight ventral germ slit almost spore-length, in 1% SDS. **K** Immature pigmented ascospore showing a ventral germ slit slightly less than spore-length, in black Pelikan ink. **L** Ascospore in India ink showing the absence of sheath or appendage. **M–O** Ascospores in side view showing slightly pinched narrowly rounded ends, in chlorazol black, 1% SDS and black Pelikan ink respectively. **P** Ascospores at different states of maturity, in 1% SDS. Bars in **A, C, D** = 10 mm; **B, F** = 0.5 mm; **E** = 1 mm; **G, H** = 50 µm; **I** = 2 µm; **J–O** = 10 µm; **P** = 20 µm

***Xylaria macouriana*** J. Fourn., Y.-M. Ju, H.-M. Hsieh & Lechat, sp. nov. Figure 36, Table 25

**Table 25:** Synoptic table of morphological differences between *X. macouriana* and *Penzigia cretacea, X. dealbata* and *X. fockei* reported in literature

	Stroma	Stipe	Interior	Ascospores	Germ slit	Ascospore ends	Ascal apical apparatus
*P. cretacea*, Australia	25–35 mm, subglobose	10–40 mm	Woody?	25–30 × 12–20 µm		oblong	
*X. fockei* PNG (Van der Gucht 1995)	30 × 3–5 mm	20–50 × 10–20 mm, concolorous	White to yellowish brown, hollow	30–35(−37) × 7–8 µm	Oblique 1/2–2/3	acute	6 × 3.5 µm
*X. fockei* Congo (Dennis 1961)	35 × 15 mm			24–33 × 6–11 µm		apiculate	
*X. dealbata* Surinam (Dennis 1956)	30 × 10 mm total		White, blackening and hollow	24–33 × 6–11 µm		acute	
*X. dealbata* North Sulawesi (Rogers et al. 1987)	30–60 × 25 mm total		Brownish to blackish, hollow	26–32.5 × (6–) 6.6–7.3 µm	Straight to undulating 1/2–2/3	acute	5.9–7.3 × 3.7–5 µm
*X. dealbata* Venezuela (Rogers et al. 1988)	40 × 5–7 mm	Up to 50 mm, concolorous	Tan to blackish, hollow	26.5–32 × 7.5–9 µm	<spore-length, parallel to oblique	abruptly acute	6.5 × 4 µm
*X. fockei* Congo (Dennis 1961)	35 × 15 mm		Wood-colored	24–33 × 6–11 µm		apiculate	
*X. fockei* PNG (Van der Gucht 1995)	30 × 3–5 mm	20–50 × 10–20 mm, concolorous	White to yellowish brown hollow	30–35(−37) × 7–8 µm	Oblique, 1/2–2/3	acute	6 × 3.5 µm
*X. macouriana* FG *CLL8095*	40–50 × 10–18 mm	Blackish at base	White, hollow	20.3–23 × 6.9–8.1 µm	≤spore-length, straight	Blunt pinched	3.0 × 2.5 µm

MycoBank MB863169

**Typification**: **FRENCH GUIANA.** Macouria, vicinity of Cayenne, Risquetout Forest, trail to Saut-Léodate, 4.899372 N, 52.555061 W, ca. 55 m, lowland rainforest, on dead buried wood, 6 May 2008, *Lechat, C. CLL8095* (holotype HAST 147376).

**Etymology**: From Macouria, the collecting site.

**Diagnosis**: Differs from *X. dealbata* and allies by smaller ascospores 21.6 × 7.5 µm on average with bluntly rounded, slightly pinched ends and a straight germ slit almost spore-length, and asci with smaller apical apparati.

**Stromata** 70–150 mm in total height including long rooting stipes, the fertile parts 35–60 mm high × 10–18 mm wide, simple, clavate, apically broadly rounded, vertically split, shrunk and deformed upon drying, very hard-textured; the stipes 15–40 mm high, 4–8 mm wide, more or less well-defined, flattened and often split, blackish, carbonaceous, glabrous, with a slightly swollen base arising from orange-brown rooting stipes 30–70 mm long, tomentose and incrusted with soil particles. Surface gray, smooth, mottled, with a white persistent background covered by a dense reticulate network of slightly prominent blackish lines delimiting narrow white scales and blackish polygonal ostiolar areas 250–350 µm diam; stromatal crust just beneath surface black, carbonaceous, 120–170 µm thick; interior whitish to ivory, solid, homogeneous, woody, becoming centrally hollow in places. **Perithecia** fully immersed, subglobose to laterally flattened, 1–1.2 mm high × 0.6–0.85 mm diam. **Ostioles** opening at the center of blackish polygonal areas, faintly papillate to nonpapillate, inconspicuous, black.

**Paraphyses** hyphal, hyaline, remotely septate, 4–6 µm wide at base, tapering to 1.5–2 µm wide above asci, embedded in mucilaginous material. **Asci** fragmentary, cylindrical, with (6–)8 uniseriately arranged, slightly overlapping ascospores, the spore-bearing parts 145–165 µm long × 8.5–10.0 µm wide, the stipes 55–95 µm long, fragile, with apical apparatus cuboid to slightly urn-shaped in outline, with a faint to conspicuous upper rim, 2.6–3.4 × 2.3–2.8 µm (Me = 3.0 × 2.5 µm, *N* = 18), bluing in Melzer’s reagent. **Ascospores** (19.5–)20.3–23(−24.9) × (6.5–)6.9–8.1(−8.8) µm, Q = (2.5–)2.6–3.2(−3.5) *N* = 60 (Me = 21.6 × 7.5 µm, Qe = 2.9), fusiform-inequilateral with bluntly to narrowly rounded, slightly pinched ends, blackish brown, opaque at maturity, with a straight, parallel to the sides germ slit slightly less than spore-length on the ventral side, only visible on immature ascospores; lacking sheath or appendages; epispore smooth.

**Asexual morph** on the natural substrate not observed.

**Known distribution**: French Guiana, only from the holotype collection.

**Comments**: Collections of tropical *Xylaria* species featuring large, globose to clavate, hard-textured, stipitate stromata with a whitish surface covered by a dense reticulate network of blackish lines delimiting narrow white scales and blackish ostiolar areas, combined with large, blackish, fusiform ascospores, have usually been referred to *X. dealbata* Berk. & M. A. Curtis or *X. fockei* (Miq.) Cooke. Both names were synonymized by Dennis (1961) who also regarded *Penzigia cretacea* (Berk. & Broome) Sacc. & Paol. as “a probable large state” of *X. dealbata* (Dennis 1956). However, data summarized in Table 25 reveal discrepancies in microscopic features, suggesting that these three species may in fact be distinct.

The collection documented above was initially assigned to *X. dealbata* because its stromatal morphology matches well that of the species (Dennis 1956; Rogers et al. 1987, 1988), the luxuriant growth from buried wood likely accounting for the large stromata and long rooting stipes. A closer examination of microscopic characters, however, shows clear deviations from the concept of *X. dealbata* accepted by these authors. The ascospores (Table 25) are smaller, with only slight overlap in dimensions, and differ in having narrowly rounded, slightly pinched ends rather than sharply acute ones. The germ slit, when observable on immature ascospores, consistently appears almost spore-length and parallel to the sides, differing from that recorded for *X. dealbata*. Moreover, the ascal apical apparatus is significantly smaller and more cuboid in outline. The difference from *P. cretacea* and *X. fockei* lies mainly in their even larger ascospore dimensions. *Xylaria macouriana* is here proposed as distinct from *X. dealbata* and its supposed allies.

**Fig. 37:**
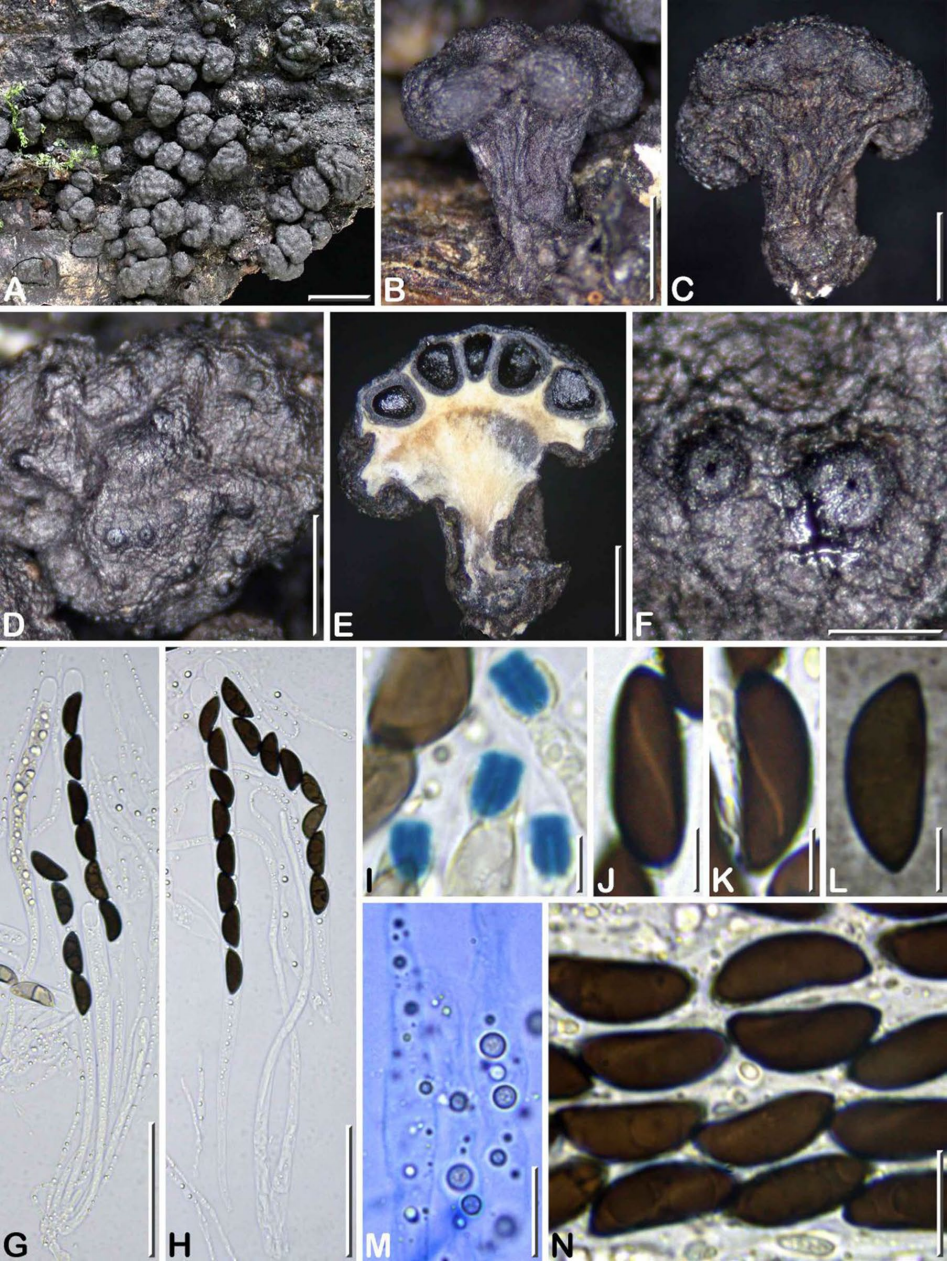
*Xylaria mamillata* (*GYJF21291* holotype). **A** Crowded stromata on bark, in top view. **B, C** Stromata in side view showing a bumpy fertile head and a well-defined stipe. **D** Stromatal surface showing bumps, shallow cracks and ostioles. **E** Stroma in vertical section showing perithecia beneath a thin leathery crust. **F** Ostioles in close-up. **G, H** Four- and eight-spored asci, in 1% SDS. **I** Ascal apical apparati in Melzer’s reagent. **J, K** Ascospores showing a germ slit, respectively in ventral and side view, in Melzer’s reagent. **L** Ascospore in India ink showing absence of sheath or appendage. **M** Guttulate paraphyses in blue Pelikan ink. **N** Ascospores in side view, some showing a faint germ slit, in 1% SDS. Bars in **A** = 5 mm; **B–E** = 1 mm; **F** = 0.2 mm; **G, H** = 50 µm; **I–L** = 5 µm; **M**, **N** = 10 µm

***Xylaria mamillata*** J. Fourn., Y.-M. Ju, H.-M. Hsieh & Lechat, sp. nov. Figure 37

MycoBank MB863170

**Typification**: **FRENCH GUIANA.** Maripasoula, Saül, trail head to Sentier Roche-Bateau, 3.62056 N, 53.199899 W, ca. 240 m, disturbed secondary rainforest, on dead corticated trunk, 30 Mar 2021, *Fournier, J. GYJF21291* (holotype HAST 147377).

**Etymology**: From Latin *mamillatus =* bearing small protuberances like nipples or mammae, referring the bumpy surface of the stromatal head.

**Diagnosis**: Differs from known penzigioid or kretzschmarioid species of *Xylaria* with similar small, turbinate, soft-textured stromata by the combination of a convex, bumpy fertile head lacking thick scales and ascospores averaging 15.5 × 5.7 µm with a sigmoid germ slit.

**Stromata** solitary to most often densely clustered, 2.3–3.5 mm high, 1.5–4.2 mm diam, stalked, turbinate; fertile head convex and bumpy with deep wrinkles and slightly to strongly exposed perithecial contours; surface blackish, minutely cracked into appressed polygonal blackish gray scales; subsurface a leathery crust 40–60 µm thick; interior solid, compact, pithy to waxy, cream-white with orange-brown zones beneath the perithecial layer; the stipes well-defined, 1.3–2.5 mm high, tapering to the base, dark gray to black, puckered, glabrous in upper part, slightly tomentose at base. **Perithecia** (1–)8–50 per stroma, subglobose to laterally flattened, 0.4–0.6(−0.75) mm diam. **Ostioles** raised-discoid, black, 120–210 µm diam, black to shiny black, with a central pore or minute papilla.

**Paraphyses** copious, hyphal, remotely septate, thin-walled, 5.5–7.0 µm wide at base, tapering to 1.5–2.0 µm wide above asci, discreetly embedded in mucilaginous material, with numerous scattered oily guttules 1.0–3.5 µm diam. **Asci** cylindrical, with (4–)8 slightly overlapping uniseriately arranged ascospores, the spore-bearing parts 103–123 × 7.0–8.0 µm, the stipes 70–115 µm long, fragile, with apical apparatus broadly tubular to slightly urn-shaped, attenuated at base, with an obtuse, weakly prominent apical rim, 4.9–5.9 × 3.2–3.8 µm (Me = 5.5 × 3.5 µm, *N* = 24), strongly bluing in Melzer’s reagent. **Ascospores** (13.3–)14.6–16.6(−17.4) × (5.2–)5.4–6.1(−6.8) µm, Q = (2.2–)2.5–3.0(−3.2), *N* = 60 (Me = 15.5 × 5.7 µm, Qe = 2.7), ellipsoid-inequilateral to navicular, ventrally slightly concave, with narrowly rounded, slightly pinched ends, dark brown to blackish brown, with a fairly conspicuous, sigmoid germ slit ca. 2/3 spore-length on the ventral side; lacking appendages or mucilaginous sheath; epispore smooth.

**Asexual morph** on the natural substrate not observed.

**Known distribution**: French Guiana, known only from the holotype collection.

**Comments**: *Xylaria mamillata* is characterized by its small, gregarious, turbinate black stromata with a bumpy fertile head whose surface is minutely cracked and lacks thick scales, a glabrous and smooth obconical stipe, and ascospores averaging 15.5 × 5.7 µm with acute to slightly pinched ends and a sigmoid germ slit. This combination of features sets it apart from similar species, particularly its nodulose fertile head with thin, appressed scales. The ascospores of *X. mamillata* are strikingly similar in shape and germ slit morphology to those of *X. scruposa*, but smaller (15.5 × 5.7 µm on average vs 19.4 × 6.5 µm). The two species are readily distinguished by stromatal morphology and surface characters (Fournier et al. 2019). The collections described herein as *Xylaria* sp. aff. *X*. *scruposa* have ascospores within the same size range as *X. mamillata* and appear closely related in our phylogram; however, their divergent stromatal morphology supports the recognition of *X. mamillata* as a distinct species within the complex of taxa related to *X. scruposa*.

**Fig. 38:**
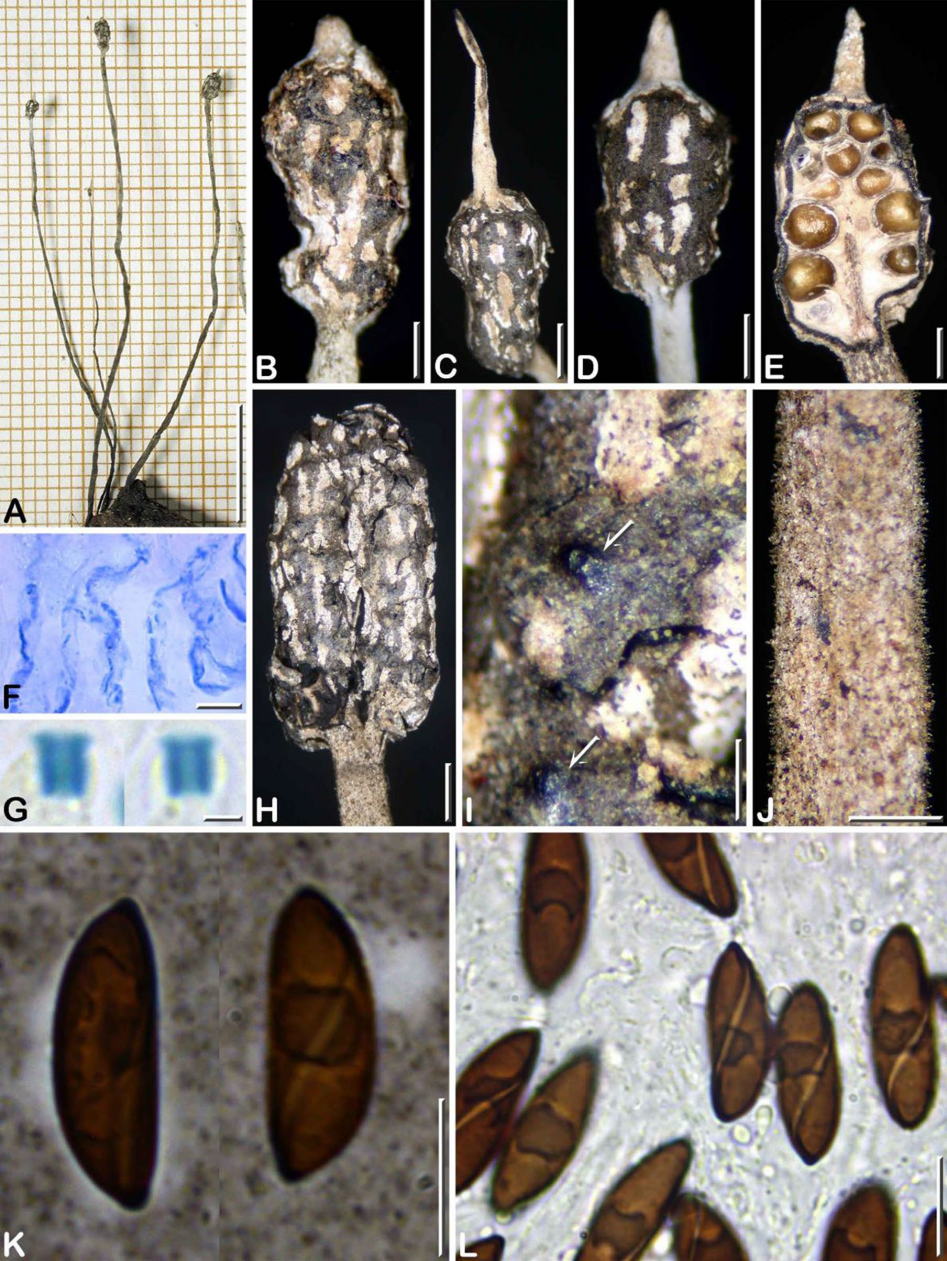
*Xylaria melaniae* (*GYJF12098* holotype). **A** Bundle of stromata arising from the base of a dead petiole. **B–D** Fertile heads of immature stromata with apical extensions of various length. **E** Immature stroma in vertical section. **F** Paraphyses in blue Pelikan ink. **G** Ascal apical apparati in Melzer’s reagent. **H** Somewhat crushed head of a mature stroma. **I** Surface of a mature stroma in close-up showing two ostioles (arrows). **J** Minutely downy stipe. **K** Ascospores in India ink showing variously distributed mucilaginous material. **L** Ascospores in 1% SDS, some in ventral view showing a spiraling germ slit. Bars in **A** = 10 mm; **B, D, E, J** = 0.5 mm; **C, H** = 1 mm; **F, K, L** = 10 µm; **G** = 2 µm; **I** = 0.2 mm

***Xylaria melaniae*** J. Fourn., Y.-M. Ju, H.-M. Hsieh & Lechat, sp. nov. Figure 38

MycoBank MB863171

**Typification**: **FRENCH GUIANA.** Régina, Nouragues natural reserve, Inselberg camp, trail 11, 4.311240 N, 52.132789 W, ca. 300 m, hygrophilic rainforest, on dead leaf petioles 2–4 mm diam in the leaf litter, 19 Jun. 2012, *Roy, M. GYJF12098* (holotype HAST 147378), largely immature.

**Etymology:** Named after Mélanie Roy, the collector of the holotype.

**Diagnosis**: Differs from known species of the *X. heloidea* group by the combination of ellipsoid fertile heads with unexposed perithecial contours and a whitish outer layer splitting into stripes or elongated scales, and fusiform-inequilateral ascospores 16.9–20.1 × 6.1–7.1 µm, with a spiraling germ slit and lacking secondary appendages.

**Stromata** scattered or in small bundles on dead unidentified leaf petioles, upright, 37–55 mm in total height, long-stipitate, with distinctly differentiated fertile heads; the fertile heads oblong to irregularly ellipsoid, (1.5–)2.8–5.6 mm high × 1.0–2.8 mm diam, apically sterile with mucronate to spike-shaped extensions 0.25–3.2 mm high, the stipes filiform, 30–51 mm high × 0.25–0.85 mm diam; surface dark gray to blackish, with unexposed to slightly exposed perithecial contours, glabrous, with a beige to pure white outer layer splitting into stripes or elongated scales; underlying crust brittle, black, slightly carbonaceous, 60–80 µm thick; the stipes terete to flattened, slightly carbonaceous, flexible, blackish at base, upwardly grayish to beige, minutely downy; interior loosely fibrous, white with an indistinct brownish core. **Perithecia** subglobose, ca 0.40 mm diam. **Ostioles** finely papillate, apically truncate and porate, black, 60–80 µm diam at base.

**Paraphyses** hyphal, thin-walled, remotely septate, faintly guttulate, 5–7 µm wide at base, tapering to 1.5–2 µm wide above asci, embedded in mucilaginous material. **Asci** fragmentary, not in measurable condition, with apical apparatus short-cylindrical with a sharp upper rim, 2.8–3.3 × 2.0–2.5 µm (Me = 3.0 × 2.3 µm, *N* = 25), bluing in Melzer’s reagent. **Ascospores** (16.4–)16.9–20.1(−21.6) × (5.8–)6.1–7.1(−7.6) µm, Q = (2.4–)2.5–3.1(−3.3), *N* = 60 (Me = 18.7 × 6.7 µm, Qe = 2.8), fusiform-inequilateral, with narrowly rounded to subacute ends, medium brown, reddish brown in the dry state, unicellular, with a conspicuous spiraling germ slit extending on the ventral side over slightly more than half spore-length, rarely on the dorsal side, lacking a defined sheath or appendages but frequently with attached remnants of mucilaginous material; epispore smooth.

**Asexual morph** on the natural substrate not observed. The white pruina coating the tip of an immature stroma proved sterile.

**Known distribution**: French Guiana, known only from the holotype collection.

**Comments**: Because the combination of characters displayed by *X*. *melaniae* does not match any species treated in the recent monograph of caulicolous *Xylaria* species (Ju and Hsieh 2023), *X. melaniae* is here introduced as a distinct taxon. The species most similar in ascospore morphology and approximate dimensions is *X. meliacearum* Læssøe, which differs markedly by a filiform fertile part with strongly exposed perithecial contours and frequently a sulphur-yellow coating (Læssøe and Lodge 1994; Ju and Hsieh 2023). Moreover, the ascospores of *X. meliacearum* are longer, 21.5–27.5 × 5.5–7.0 µm according to Ju and Hsieh (2023) and 19.1–30.0 × 4.6–6.6 µm according to Læssøe and Lodge (1994), and apical apparatus is significantly larger, respectively 5.5–7.0 × 3.5–4.5 µm and 4.0–5.9 × 2.6–4.0 µm as reported by these authors.

**Fig. 39:**
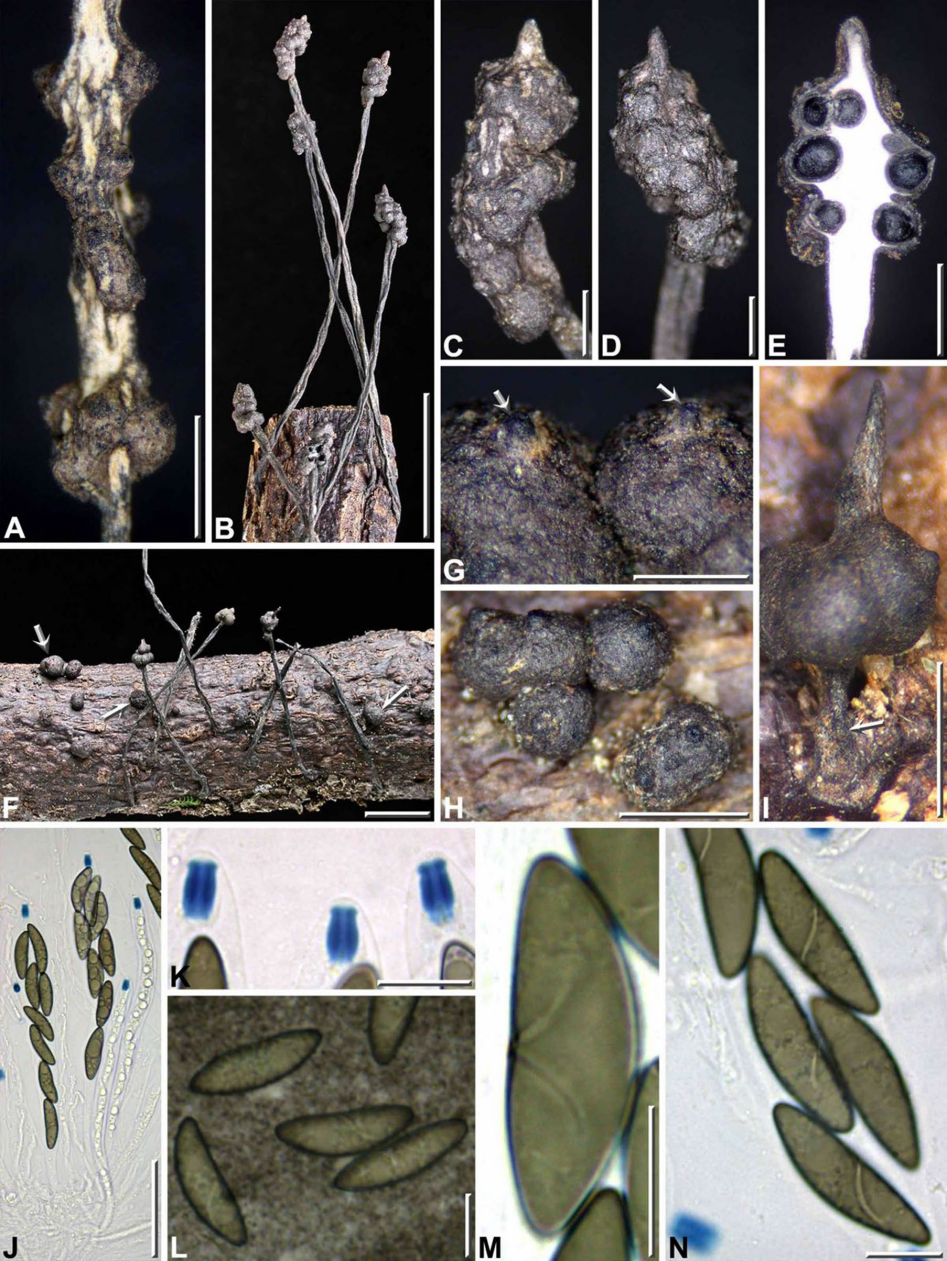
*Xylaria meliacearum* (*GYJF21160*). **A** Immature stroma with a discontinuous white peeling outer layer. **B** Long-stipitate mature stromata. **C, D** Fertile heads in side view showing apiculate apices and exposed perithecial contours with superficial remnants of a white to gray outer layer. **E** Fertile head in vertical section. **F** Mixed long-stipitate and penzigioid (arrows) stromata on host surface. **G** Two ostioles (arrows) on a fertile head in close-up featuring a vaguely circular halo of tan superficial tissue. **H** Uni- to biperitheciate sessile stromata. **I** Few-peritheciate strongly apiculate stroma on a short stipe (arrow). **J** Immature and mature asci in Melzer’s reagent. **K** Ascal apical apparati. **L** Ascospores in India ink showing an absence of sheath or appendages. **M** Ascospore in side view showing a short ventral spiraling germ slit. **N** Variously oriented ascospores showing a ventral germ slit (**K, M, N** in Melzer’s reagent). Bars in **A, C–E, H, I** = 1 mm; **B** = 10 mm; **F** = 5 mm; **G** = 0.5 mm; **J** = 50 µm; **K–N** = 10 µm

***Xylaria meliacearum*** Læssøe, in Læssøe & Lodge, Mycologia 86: 441. 1994. Figure 39, Table 26

**Table 26:** Comparison table of ascospore and ascal apical apparatus dimensions in the two stromatic forms of *GYJF21160* with those of *X. meliacearum, X. chordaeformis, X. fallax* and *X. luxurians*

Collection numbers	Ascospore measurements with extreme values in parentheses	Q = quotient l/w, *N* = number of measurements	Me = mean values of l/w, Qe = mean quotient l/w	Ascal apical apparatus h × w (µm), *N* = 25
*GYJF21160* (long-stipitate)	(22.1–)22.8–25.0(−26.1) × (5.7–)6.0–6.8(−7.8) µm	Q = (3.1–) 3.4–4.1(−4.6), *N* = 60	Me = 23.9 × 6.4 µm, Qe = 3.7	5.2–5.9 × 2.9–3.6 µm, Me = 5.6 × 3.4 µm
*GYJF21160* (penzigioid)	(21.8–)23.3–26.0(−27.3) × (6.2–)6.4–7.5(−8.0) µm	Q = (3.0–)3.2–3.9(−4.2), *N* = 60	Me = 24.5 × 7.1 µm, Qe = 3.5	5.3–6.1 × 3.4–4.0 µm, Me = 5.6 × 3.7 µm
**Cumulated values**	**(21.8–)22.8–26.0(−27.3) × (5.7–)6.0–7.5(−8.0) µm**	**Q = (3.0–)3.2–4.1(−4.6), N = 120**	**Me = 24.2 × 6.8 µm, Qe = 3.6**	**5.2–6.1 × 2.9–4.0 µm, Me = 5.6 × 3.6 µm**
*X. meliacearum* (Læssøe and Lodge 1994)	(18.5–)19.1–30.0(−33.0) × (4.0–)4.6–6.6(−7.9) μm		Me = 24.2 × 5.8 µm, Qe = 4.2	4.0–5.9 × 2.6–4.0 µm, Me = 5.0 × 3.3 µm
*X. chordaeformis* (Ju et al. 2016)	21.5–24.5 × 5.5–7.0(−7.5) μm		Me = 23.0 × 6.3 µm, Qe = 3.7	
*X. fallax* (this paper)	(26.1–)27.2–30.1(−31.2) × (9.8–)10.3–11.4(−12.4) µm	Q = (2.3–) 2.4–2.8 (−3.0), *N* = 60	Me = 28.7 × 10.9 µm, Qe = 2.6	6.4–7.6 × 3.7–4.4 µm, Me = 6.9 × 4.0 µm
*X. luxurians* (Ju et al. 2016)	20.0–24.5 × (6.0–)6.5–7.5(−8.0) µm		Me = 22.3 × 7.0 µm, Qe = 3.6	

**Immature stromata** upright, filiform, 30–40 mm in total height, 0.35–0.55 mm diam, with scattered superficial perithecia distributed in upper part, not reaching the tip, partly coated by a whitish to yellowish peeling outer layer. **Mature stromata** of two types. **Upright to prostrate long-stipitate stromata** 12–37 µm in total height, simple, comprised of a fertile head and a wiry stipe; the fertile heads 1.8–4.9 mm high × 1.4–1.7 mm broad, subglobose to ellipsoid with a sharply pointed sterile apex, nodulose with exposed perithecial contours; surface blackish, finely roughened, with superficial grayish remnants of a vanishing outer layer; subsurface a leathery crust 50–60 µm thick; the stipes wiry, unbranched, slightly enlarged at base, 10–32 mm high × 0.4–0.7 mm broad, blackish, glabrous, flexible, puckered; interior white, solid, pithy. **Perithecia** subglobose, 0.6–0.7 mm diam. **Ostioles** obtusely papillate, black, 120–130 µm diam at base, occasionally surrounded by a circular halo of tan superficial tissue. **Associated penzigioid stromata** on bark sessile to shortly stipitate, uni- to few-peritheciate, 0.65–2.6 mm high, 1.1–2.2(−3.0) mm broad, uniperitheciate stromata subglobose, separate to coalescent in small groups, few-peritheciate stromata occasionally forming a small shortly stipitate and apiculate stroma; surface, crust, interior, perithecia and ostioles as in the stipitate form.

**Paraphyses** copious, hyphal, hyaline, remotely septate, simple, 7.5–11.5 µm wide at base, tapering to 1.5–2 µm wide, embedded in mucilaginous material. **Asci** cylindrical, with (6–)8 uniseriately, occasionally biseriately arranged, slightly overlapping ascospores, the spore-bearing parts 154–185 µm long × 14.5–19.5 µm wide, the stipes 90–100 µm long, with apical apparatus urn-shaped with an obtuse upper rim, 5.2–6.1 × 2.9–4.0 µm (Me = 5.6 × 3.6 µm, *N* = 50), bluing in Melzer’s reagent. **Ascospores** (21.8–)22.8–26.0(−27.3) × (5.7–)6.0–7.5(−8.0) µm, Q = (3.0–)3.2–4.1(−4.6) *N* = 120 (Me = 24.2 × 6.8 µm, Qe = 3.6), fusiform-inequilateral with narrowly rounded ends, straight to most often slightly curved, light olivaceous brown in the fresh state, light to medium brown in the dry state, with a short strongly spiraling germ slit extending on the ventral side over ca 60–70% of the circumference, without sheath or appendage visible in India ink; epispore smooth.

**Asexual morph** on the natural substrate not observed but described by Læssøe and Lodge (1994) in culture.

**Specimen examined: FRENCH GUIANA.** Maripasoula, Saül, trail head of Sentier des Gros Arbres, 3.6201 N, 53.207989 W, ca. 210 m, disturbed secondary rainforest, on dead corticated branchlet, 27 Mar 2021, *Fournier, J. GYJF21160* (HAST 147379).

**Known distribution**: Ecuador, Costa Rica, Puerto Rico (Læssøe and Lodge 1994); French Guiana (this paper).

**Comments**: This puzzling collection was not assigned to *X. meliacearum* until phylogenetic data unambiguously demonstrated its conspecificity with a Puerto Rican collection. Unlike our Guianese collection, the Puerto Rican collection was originally described as featuring strap-like stromata with free perithecia intermixed with short, clavate anamorphic coremia, and a surface roughened by sulphur-yellow squamules. However, on account of the substantial morphological variation observed across multiple collections of *X. meliacearum*, Læssøe and Lodge (1994) broadened the species concept to include sessile, penzigioid stromata; perithecia aggregated in cylindrical stromata with a cream-colored to grayish-white outer layer lacking sulphur-yellow tones; absence of coremia; and highly variable ascospores (18.5–)19.1–30.0(−33.0) × (4.0–)4.6–6.6(−7.9) μm. Our present collection fits well this broadened concept, and the mean ascospore dimensions match those of *X. meliacearum* (Table 26) as does the distinctive germ slit morphology.

It is noteworthy that the discussions on the main morphological differences between *X*. *meliacearum* and the closely related *X. chordaeformis* C. G. Lloyd and *X. luxurians* (Rehm) C. G. Lloyd by Læssøe and Lodge (1994) and Ju et al. (2016) emphasize ascospore dimensions and the shape and location of the germ slit as the most discriminating characters. These same characteristics also support that penzigioid stromata belong to the same species as the long, stipitate forms, and that *X. fallax*, associated with the present collection of *X. meliacearum*, is recognized as a distinct species (this paper).

**Fig. 40:**
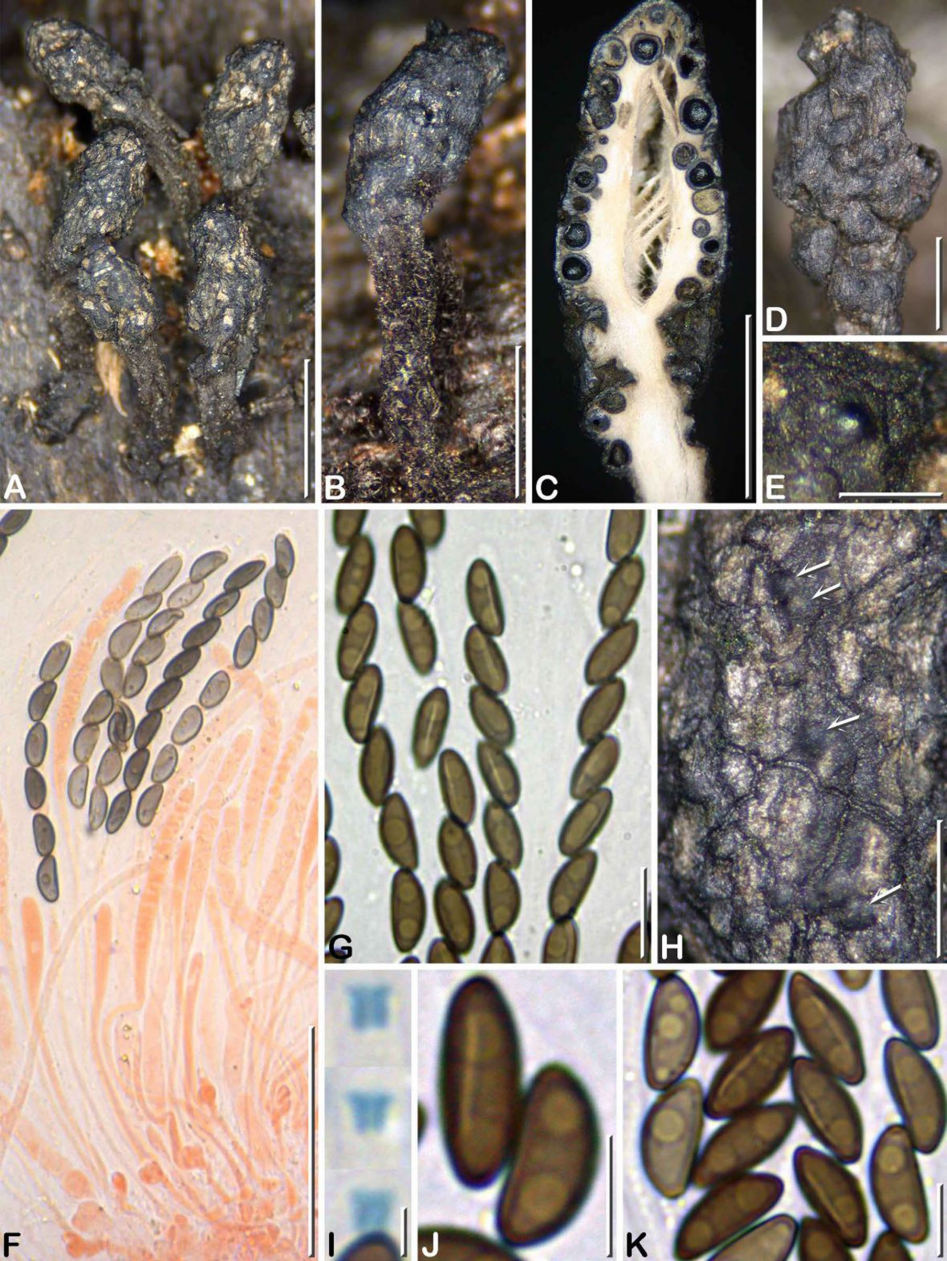
*Xylaria minor* (**A, C, E, H ***GYJF18005*; **B, I–K ***GYJF18128* holotype; **D, F, G ***GYJF21141*). **A** Clustered stromata. **B** Isolated stroma with hairy-tomentose stipe. **C** Stroma in vertical section. **D** Atypical stroma with a nodulose surface. **E** Shiny black ostiole in close-up. **F** Immature and mature asci in Congo red and 3% KOH. **G** Variously oriented ascospores in 1% SDS, some showing a germ slit. **H** Stromatal surface in close-up showing gray scales and ostioles (arrows). **I** Ascal apical apparati in Melzer’s reagent. **J, K** Ascospores in Melzer’s reagent, some showing germ slits. Bars in **A** = 5 mm; **B, C** = 2 mm; **D** = 1 mm; **E** = 0.2 mm; **F** = 50 µm; **G** = 10 µm; **H** = 0.5 mm; **I** = 2 µm; **J, K** = 5 µm

***Xylaria minor*** J. Fourn., Y.-M. Ju, H.-M. Hsieh & Lechat, sp. nov. Figure 40, Table 27

**Table 27:** Ascospore and ascal apical apparatus dimensions in eight collections of *X. minor* from French Guiana showing their variation range, compared with those of *X. spegazziniana*

Collection numbers	Ascospore measurements with extreme values in parentheses	Q = quotient l/w, *N* = number of measurements	Me = mean values of l/w, Qe = mean quotient l/w	Ascal apical apparatus h × w, *N* = 25
*GYJF18005*	(6.7–)7.0–8.5(−9.5) × (3.0–)3.3–3.8(−4.1) µm	Q = (1.8–)1.9–2.4(−2.8), *N* = 60	Me = 7.9 × 3.6 µm, Qe = 2.2	
*GYJF18128* holotype	(6.5–)7.6–8.4(−8.9) × (3.1–)3.4–3.9(−4.0) µm	Q = (1.9–)2.0–2.4(−2.6), *N* = 60	Me = 8.0 × 3.6 µm, Qe = 2.2	1.6–2.0 × 1.2–1.4 µm, Me = 1.7 × 1.3 µm
*GYJF19001*	(7.4–)7.8–9.2(−9.7) × (3.2–)3.4–4.0(−4.3) µm	Q = (1.8–)2.1–2.5(−2.8), *N* = 60	Me = 8.5 × 3.7 µm, Qe = 2.3	
*GYJF19128*	(7.2–)7.7–8.9(−9.7) × (3.3–)3.5–4.1(−4.4) µm	Q = (1.9–)2.0–2.4(−2.5), *N* = 60	Me = 8.3 × 3.8 µm, Qe = 2.2	
*GYJF21057*	(7.0–)7.4–8.4(−8.8) × (3.1–)3.3–3.8(−4.1) µm	Q = (2.0–)2.1–2.5(−2.8), *N* = 60	Me = 7.9 × 3.5 m, Qe = 2.3	1.5–1.7 × 1.3–1.5 µm, Me = 1.6 × 1.4 µm
*GYJF21066*	(7.6–)8.0–9.3(−10.3) × (3.4–)3.6–4.2(−4.6) µm	Q = (1.8–)2.0–2.5(−2.7), *N* = 60	Me = 8.6 × 3.9 µm, Qe = 2.2	
*GYJF21141*	(7.4–)7.7–8.7(−9.2) × (3.1–)3.3–3.8(−3.9) µm	Q = (2.0–)2.2–2.5(−2.6), *N* = 60	Me = 8.2 × 3.5 µm, Qe = 2.3	
*GYJF21428*	(6.8–)7.2–8.4(−8.8) × (3.1–)3.3–3.8(−4) µm	Q = (1.8–)2.0–2.4(−2.6), *N* = 60	Me = 7.8 × 3.6 µm, Qe = 2.2	
**Cumulated values**	**(6.5–)7.0–9.3(−10.3) × (3.0–)3.3–4.2(−4.6) µm**	**Q = (1.8–)1.9–2.5(−2.8), N = 480**	**Me = 8.2 × 3.7 µm, Qe = 2.2**	
*X. spegazziniana* (this paper)	(7.9–)8.2–10.6(−11.4) × (2.7–)3.3–4.4(−4.7) µm	Q = (1.8–)2.2–2.8(−3.5), *N* = 300	Me = 9.3 × 3.8 µm, Qe = 2.45	1.8–2.3 × 1.3–1.7 µm, Me = 2.1 × 1.5 µm

MycoBank MB863172

**Typification**: **FRENCH GUIANA.** Maripasoula, Saül, Sentier des Gros Arbres, Crique Grand-Fossé, 3.617197 N, 53.209042 W, disturbed mesophilic rainforest, on dead blackened wood of *Ceiba pentadra*, 24 Aug 2018, *Gardiennet, A. GYJF18128* (holotype HAST 147385).

**Etymology**: From Latin *minor* = small, reduced, referring to the small and inconspicuous stromata.

**Diagnosis**: Differs from *X. spegazziniana,* the most akin species, by significantly smaller stromata less than 12 mm high, and smaller ascospores averaging 8.2 × 3.7 µm vs 9.3 × 3.8 µm, with the germ slit consistently on the ventral side.

**Stromata** loosely scattered or in small groups on blackened wood or bark, upright to prostrate, 3–12 mm in total height, simple, straight to slightly curved, the fertile head subclavate to fusoid with obtusely rounded fertile apex, terete to flattened, 3.2–6.7 mm high × 1.3–2.1 mm diam, on usually well-defined stipes 2.1–4.2 mm long × 0.2–1.3 mm wide, terete to strap-like, black, fragile, downy to densely hairy-tomentose, with a slightly pannose and swollen base; surface blackish, irregularly cracked, occasionally deeply wrinkled, with superficial gray scales vanishing with age; perithecial contours unexposed to conspicuously exposed; crust leathery to faintly carbonaceous, brittle, 40–60 µm thick; interior white with sometimes orange marks, solid, fibrous, becoming loosely fibrous. **Perithecia** fairly crowded, subglobose, 0.3–0.35 mm diam. **Ostioles** black to shiny black, obtusely to more rarely sharply papillate, ca. 80 µm diam at base, occasionally on a discoid base 100–130 µm diam.

**Paraphyses** hyphal, thin-walled, 4.5–5.5 µm wide at base, tapering to 0.8–1.0 µm wide above asci, discreetly embedded in mucilaginous material. **Asci** cylindrical, with (6–)8 slightly overlapping uniseriately arranged ascospores, the spore-bearing parts 48–57 × 5.0–6.0(−6.5) µm, the stipes 40–65 µm long, with apical apparatus 1.5–2.0 × 1.2–1.5 µm (Me = 1.65 × 1.35 µm, *N* = 50), short-cylindrical to slightly trapezoid with a fairly marked upper rim, bluing in Melzer’s reagent. **Ascospores** (6.5–)7.0–9.3(−10.3) × (3.0–)3.3–4.2(−4.6) µm, Q = (1.8–)1.9–2.5(−2.8), *N* = 480 (Me = 8.2 × 3.7 µm, Qe = 2.2), ellipsoid-inequilateral with mostly narrowly rounded ends, ventrally flat, brown with olivaceous tone in the fresh state, unicellular, with a conspicuous, straight, longitudinally oriented germ slit 2/3 to 3/4 spore-length on the ventral side, without sheath or appendages; epispore smooth.

**Known distribution**: French Guiana, known from this paper.

**Other specimens examined (paratypes)**: **FRENCH GUIANA.** Roura, Cacao, Camp Bonaventure, 4.326815 N, 52.341431 W, 60–65 m, secondary hygrophilic rainforest, on dead blackened wood, 22 Mar 2021, *Fournier, J. GYJF21066* (HAST 147380); Maripasoula, Saül, trail head to Monts-La-Fumée, tributary to Crique Queue-Hocco, 3.637173 N, 53.204727 W, ca. 260 m, disturbed mesophilic rainforest, on dead wood, 4 Apr 2021, *Fournier, J. GYJF21428* (HAST 147381); Maripasoula, Saül, edge of the forest next to Carbets du bord lodge, 3.623598 N, 53.210338 W, disturbed mesophilic rainforest, on dead blackened stump, 23 Jun 2019, *Fournier, J. GYJF19218* (HAST 147382); Maripasoula, Saül, edge of the forest next to Carbets du bord lodge, 3.623598 N, 53.210338 W, disturbed mesophilic rainforest, on dead blackened stump, 19 Aug 2018, *Fournier, J. GYJF18005* (HAST 147383); Maripasoula, Saül, trail head to Sentier des Gros Arbres, 3.620839 N, 53.208536 W, ca. 210 m, disturbed mesophilic rainforest, on dead blackened wood, 26 Mar 2021, *Fournier, J. GYJF21141 *(HAST 147384); Roura, Cacao, Camp Bonaventure, 4.326815 N, 52.341431 W, 60–65 m, secondary hygrophilic rainforest, on dead blackened wood, 22 Mar 2021, *Fournier, J. GYJF21057* (HAST 147386); Maripasoula, Saül, trail head to Sentier des Gros Arbres, 3.620967 N, 53.208689 W, disturbed mesophilic rainforest, on dead corticated branch, 16 Jun 2019, *Fournier, J. GYJF19001* (HAST 147387).

**Comments**: *Xylaria minor* can be regarded as *X. spegazziniana* in miniature, from which it differs mainly by minute stromata less than 12 mm high vs 4–60 mm high and slightly smaller ascospores averaging 8.2 × 3.7 µm vs 9.7 × 3.9 µm, with a length/width ratio of 2.2 vs 2.4 (Table 27). They share many characters, including obtusely ended stromata with a thin leathery crust coated with a gray outer layer splitting into short evanescent stripes, small conic-papillate ostioles, subglobose perithecia, and a white fibrous interior. The stromatal surface of *X. spegazziniana* is typically strongly nodulose with deep circumferential wrinkles, but such stromata occasionally occur in *X. minor* as well, rendering the taxonomic value of this character sometimes ambiguous.

The most discriminating features appear to be the ascospore dimensions and germ slit position. The smaller and relatively wider ascospores of *X. minor* are consistently correlated with its smaller stromata. Moreover, the germ slit is slightly shorter and consistently on the ventral side, whereas in *X. spegazziniana* it is frequently on the dorsal side. We therefore propose the new name *X. minor* on account of its reduced size consistently observable in the numerous collections made during this survey. Our phylogenetic analyses also support *X. minor* distinct from *X. spegazziniana* but confirm that they are closely related, being sister clades to each other.

***Xylaria multiplex*** (Kunze) Fr., in Fr., Nov. Act. Reg. Soc. Sci. Upsal., ser. III, 1: 127. 1851.

≡ *Sphaeria multiplex* Kunze, in Fr., Linnaea 5: 536. 1830.

The cited collection from French Guiana closely matches those reported and illustrated from the French West Indies (Fournier et al. 2020).

**Specimen examined: FRENCH GUIANA.** Maripasoula, Saül, trail head of Sentier des Gros Arbres, 3.6201 N, 53.207989 W, ca. 210 m, disturbed secondary rainforest, on dead blackened wood, 26 Mar 2021, *Fournier, J. GYJF21148* (HAST 147219).

***Xylaria muscula*** C. G. Lloyd, Mycol. Writings 6: 994. 1920.

The cited collection from French Guiana closely matches those reported and illustrated from the French West Indies (Fournier et al. 2020).

**Specimen examined: FRENCH GUIANA.** Maripasoula, Saül, trail head to Sentier des Gros Arbres, 3.620139 N, 53.208103W, ca. 210 m, disturbed secondary rainforest, on dead corticated branch, 25 Aug. 2018, *Fournier, J. GYJF18148* (JF).

**Fig. 41:**
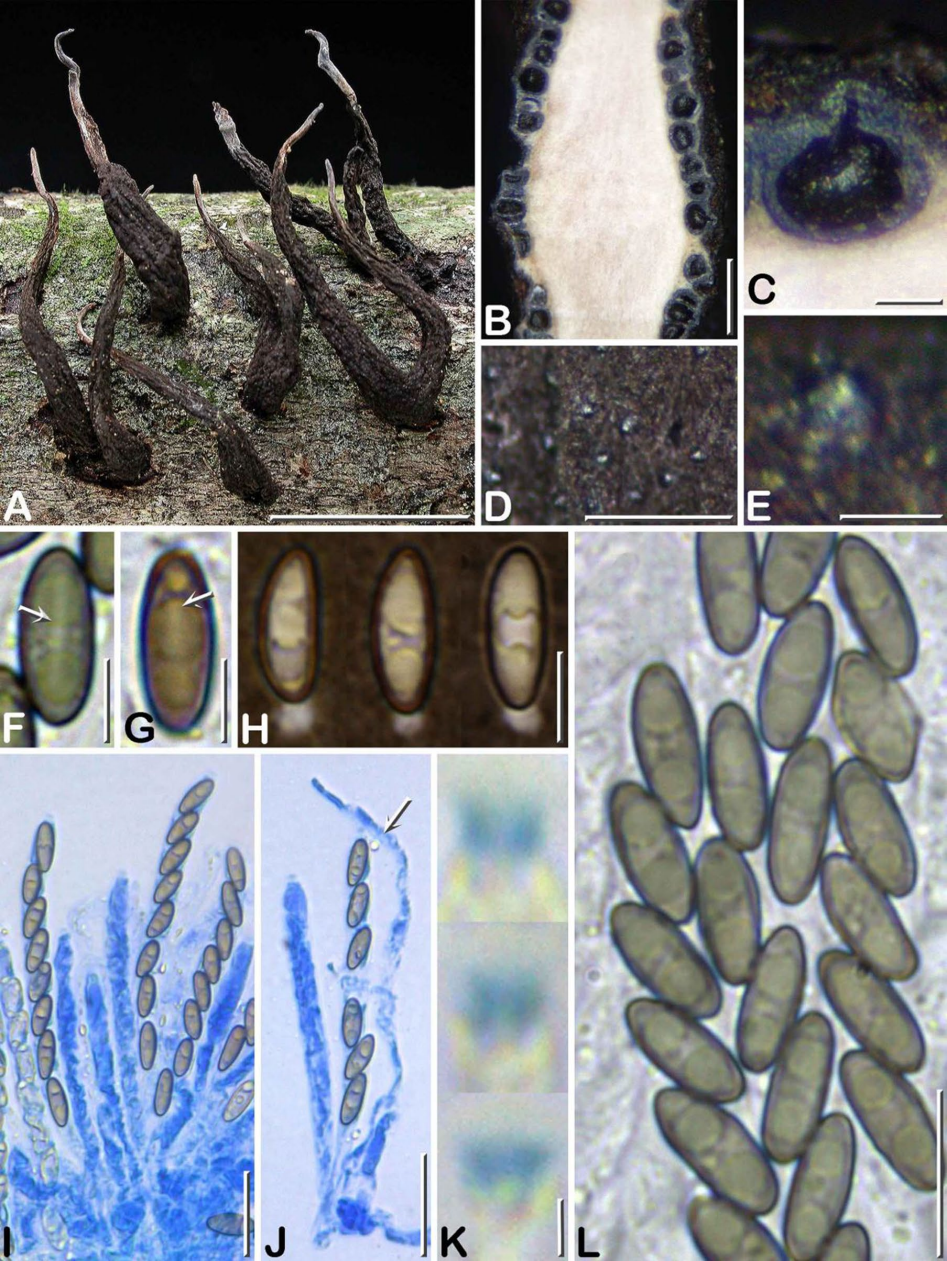
*Xylaria myosurus* (*GYJF21363*). **A** Clustered stromata on bark. **B** Stroma in vertical section showing perithecia crowded beneath a thin superficial crust and lying above a whitish compact interior. **C** Perithecium in vertical section. **D** Stromatal surface showing shiny black conic papillate ostioles on a minutely granular background. **E** Ostiole in close-up. **F** Ascospore in the fresh state showing a faint germ slit (arrow), in 1% SDS. **G** Ascospore in the dry state showing a faint germ slit (arrow), in heated chloral-lactophenol. **H** Ascospores showing a polar secondary appendage, in India ink. **I** Hymenium in blue Pelikan ink, showing mature and immature asci. **J** Six-spored short-stipitate ascus and a paraphysis (arrow), in blue Pelikan ink. **K** Variously shaped ascal apical apparati, in Melzer’s reagent. **L** Ascospores in the fresh state, in 1% SDS. Bars in **A** = 10 mm; **B, D** = 0.5 mm; **C, E** = 100 µm; **F–H** = 5 µm; **I, J** = 20 µm; **K** = 1 µm; **L** = 10 µm

***Xylaria myosurus*** Mont., Ann. Sci. Nat. Bot., sér. IV, 3: 110. 1855. Figure 41

**Stromata** in loose clusters, separate or connate at base by two, upright to somewhat prostrate, narrowly subulate with a basal dark brown fertile part, subsessile, tapering to a grayish acuminate sterile apex, 6–15 mm high, 1.5–3 mm wide at base; surface minutely wrinkled giving a granular appearance, roughened by minute prominent ostioles, glabrous; crust 20–40 µm thick including the outgrowths, slightly leathery in the dry state; interior whitish, solid, soft-textured to pithy, yellowish gray around perithecia, **Perithecia** crowded, subglobose to slightly depressed-spherical, frequently laterally flattened by mutual pressure, 0.18–0.25 mm diam, fully immersed to slightly raising the surface. **Ostioles** conic-papillate, shiny black, ca. 80 µm diam at base.

**Paraphyses** sparse, hyphal, thin-walled, remotely septate, 4.0–5.2 µm wide at base, tapering to 1.5–2 µm wide above asci, discreetly embedded in mucilaginous material. **Asci** cylindrical, with (6–)8 slightly overlapping uniseriately arranged ascospores, the spore-bearing parts 57–75 × 4.5–6.0 µm, the stipes 16–24 µm long, with a minute apical apparatus rectangular to wedge-shaped in optical view, wider than high, 0.9–1.1 × 1.3–1.6 µm (Me = 1.0 × 1.4 µm, *N* = 12), bluing in Melzer’s reagent.

**Ascospores** (7.5–)8.0–9.6(−10.2) × (2.9–)3.1–3.9(−4.1) µm, Q = (2.1–)2.2–2.9(−3.2), *N* = 120 (Me = 8.7 × 3.3 µm, Qe = 2.5), narrowly ellipsoid, nearly equilateral, with broadly rounded ends, light olivaceous brown in the fresh state, light brown in the dry state, unicellular, with an inconspicuous, straight, longitudinally oriented germ slit almost spore-length; with a polar secondary appendage visible in India ink, detaching readily; epispore smooth.

**Asexual morph** on natural substrate not observed.

**Specimens examined**: **FRENCH GUIANA.** Maripasoula, Saül, trail to Monts-La-Fumée, 3.634796 N, 53.205457 W, ca. 270 m, mesophilic rainforest, on small corticated branch, 4 Apr 2021, *Fournier, J. GYJF21363* (HAST 147389); Cayenne, on wood, *Leprieur, F. R. 1412* (holotype PC 0086065 ex Montagne herb., isotype K(M) 156832 ex Berkeley herb.).

**Known distribution**: Argentina (Hladki and Romero 2007); Brazil (Theissen 1909a; Rick 1934); French Guiana (Dennis 1956; this paper); NZ? (Rogers and Samuels 1986).

**Comments**: *Xylaria myosurus* was described from French Guiana by Montagne (1855), and the only known subsequent records are from Brazil (Theissen 1909a; Rick 1934) and Argentina (Hladki and Romero 2007). This species is distinctive by its small brown subulate stromata tapering to a gray acuminate apex, as illustrated by Dennis (1956) and Joly (1968) from the holotype and by Theissen (1909a) from a Brazilian collection. The epithet *myosurus* means “mouse tail” in Greek, which is perfectly suggestive of the stromatal morphology. The phylogenetic affinities of this rarely reported species are difficult to infer from morphology alone. Our phylogenetic analyses placed *X*. *myosurus* next to *X*. *turbiniformis*, which has stromatal morphology entirely different from the former but is similar in ascospore morphology, except for having a conspicuous, short germ slit rather than an inconspicuous, nearly spore-length one.

***Xylaria nelumboniformis*** Hai X. Ma, Lar. N. Vassiljeva & Yu Li, in Ma, Vasilyeva & Li, Mycotaxon 132: 300. 2017.

= *Xylaria peltiformis* J. Fourn. & Lechat, in Fournier, Lechat & Courtecuisse, Ascomycete.org 10: 165. 2018.

The cited collections from French Guiana closely match those reported and illustrated as *X*. *peltiformis* from the French West Indies (Fournier et al. 2018c).

**Specimens examined: FRENCH GUIANA.** Maripasoula, Saül, trail head to Sentier Roche-Bateau, Crique-Cochon, 3.625994 N, 53.1955 W, 210–220 m, disturbed secondary rainforest, on dead corticated branch, 31 Mar 2021, *Fournier, J. GYJF21306* (HAST 147390); Maripasoula, Saül, trail head toward Roche-Bateau, 3.6619046 N, 53.200658 W, disturbed rainforest, on dead corticated branch, 23 Aug 2018, *Fournier, J. GYJF18099* (HAST 147431).

**Fig. 42:**
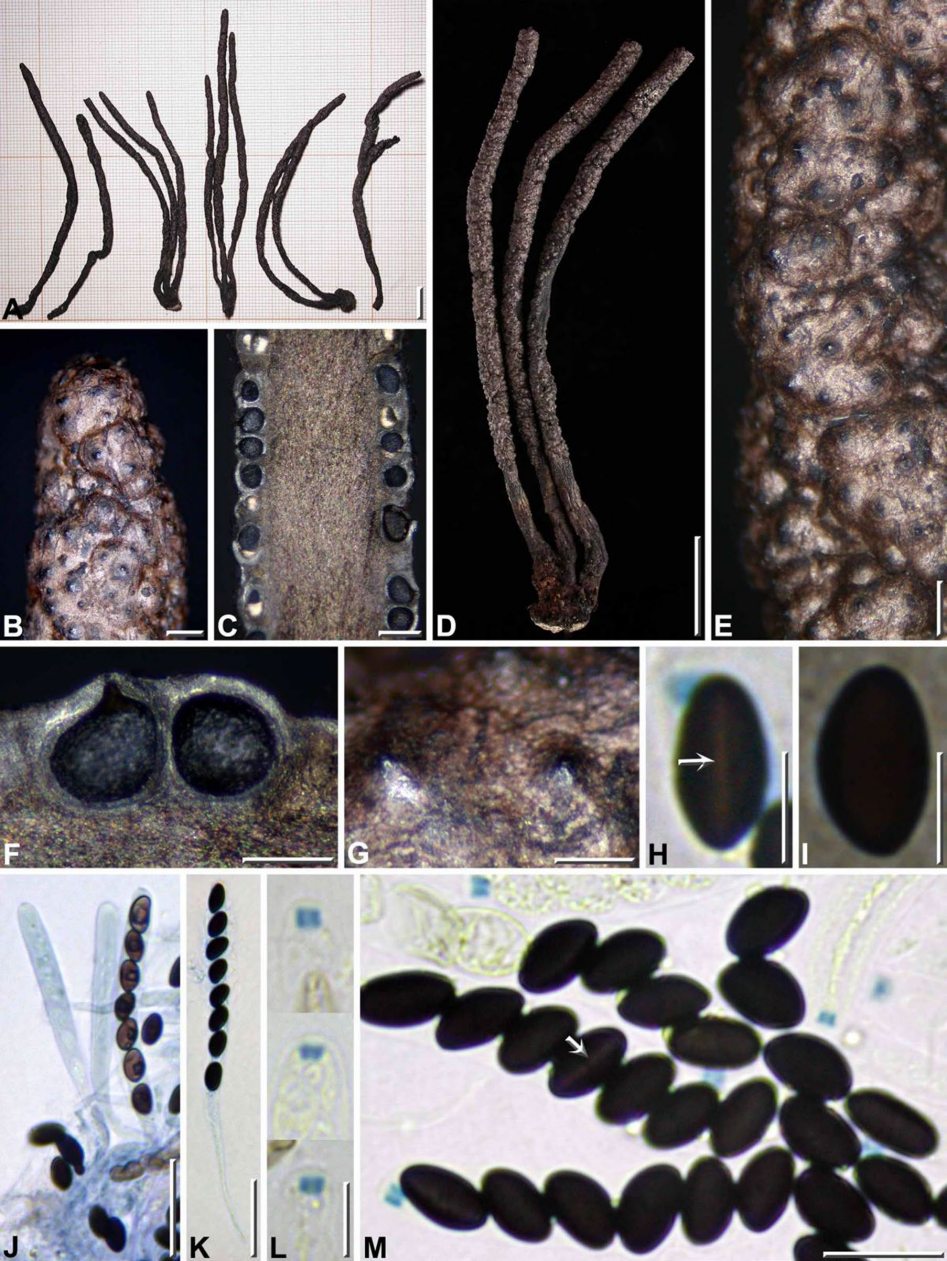
*Xylaria nigromedullosa* (*CLL9011*). **A** Stromata. **B** Obtusely rounded fertile stromatal apex showing slightly exposed perithecial contours. **C** Stroma in longitudinal section showing a solid blackish brown interior. **D** Three stromata connate at base. **E** Stromatal surface showing wrinkles and papillate ostioles. **F** Two adjacent perithecia immersed beneath a thin black carbonaceous crust. **G** Two papillate ostioles in close-up. **H** Ascospore showing a faint germ slit (arrow), in Melzer’s reagent. **I** Ascospore in India ink showing a lack of sheath or appendage. **J** Immature asci, in chlorazol black. **K** Mature ascus in blue Pelikan ink. **L** Ascal apical apparati, in Melzer’s reagent. **M** Variously oriented ascospores, one showing a germ slit (arrow), in Melzer’s reagent. Bars in **A, D** = 10 mm; **B, C, E** = 0.5 mm; **F, G** = 0.2 mm; **H, I, L** = 5 µm; **J, K** = 20 µm; **M** = 10 µm

***Xylaria nigromedullosa*** Trierveiler-Pereira & A.I. Romero, Mycotaxon 107: 150. 2009. Figure 42

**Stromata** narrowly cylindrical, terete to rarely flattened in places, simple but often fasciculate in groups of 2–3 connate at base, straight to slightly curved, 50–92 mm in total height, the fertile parts 25–80 mm high × 1.8–3.0(−4.4) mm wide, brittle, with obtusely rounded fertile apices; the stipes well-defined, 9–27 mm high, 1.2–2.0 mm wide, black, puckered, smooth to finely grooved in upper part, faintly tomentose below and with a swollen pannose base. Surface copper-brown to blackish brown, lacking a peeling outer layer, nodulose with faintly exposed perithecial mounds and shallow wrinkles delimiting small groups of perithecia, roughened by prominent ostioles; stromatal crust just beneath surface black, carbonaceous, 40–60 µm thick; interior blackish brown, black when moistened or in aged stromata, solid, fibrous. **Perithecia** subglobose to slightly depressed-spherical, 0.2–0.4 mm diam, often in contact. **Ostioles** conic papillate, conspicuous, black, slightly shiny.

**Paraphyses** not observed, apparently lacking. **Asci** mostly fragmentary, cylindrical, with (6–)8 uniseriately arranged, slightly overlapping ascospores, the spore-bearing parts 46–55 µm long × 5.5–6.0 µm wide, the stipes 38–45 µm long, fragile, with apical apparatus quadrate to slightly cuneate in outline, slightly wider than high, 1.3–1.5 × 1.5–1.8 µm (Me = 1.4 × 1.7 µm, *N* = 20), bluing in Melzer’s reagent. **Ascospores** (6.7–)6.9–7.8(−8.5) × (3.6–)3.9–4.5(−4.8) µm, Q = (1.5–)1.6–2.0(−2.2), *N* = 60 (Me = 7.4 × 4.2 µm, Qe = 1.8), ellipsoid, slightly inequilateral with broadly rounded ends, blackish brown, opaque, with a faint straight germ slit almost spore-length, best seen in Melzer’s reagent, without sheath or appendages; epispore smooth.

**Asexual morph** on the natural substrate not observed.

**Specimen examined: FRENCH GUIANA.** Macouria, vicinity of Cayenne, Risquetout Forest, trail to Saut-Léodate, 4.899372 N, 52.555061 W, ca. 55 m, lowland rainforest, on dead wood, 19 Apr 2009, *Lechat, C. CLL9011* (HAST 147391).

**Known distribution**: Brazil (Trierveiler-Pereira et al. 2009); French Guiana (this paper).

**Comments**: This collection conforms well with *X. nigromedullosa*, sharing the key features of a wrinkled surface, blackish interior and small, blackish ascospores with broadly rounded ends. Photographs and a brief description of this collection were sent to L. Trierveiler-Pereira, who described this species from Brazil, and agreed that it was most likely conspecific (pers. comm.).

Some differences from the original description were noticed, including taller stromata with a more narrowly cylindrical shape, a thinly carbonaceous and brittle crust, a more deeply wrinkled surface, and slightly smaller ascospores and apical apparati. Additional collections of this remarkable species will be needed to determine whether these differences fall within natural intraspecific variation.

**Fig. 43:**
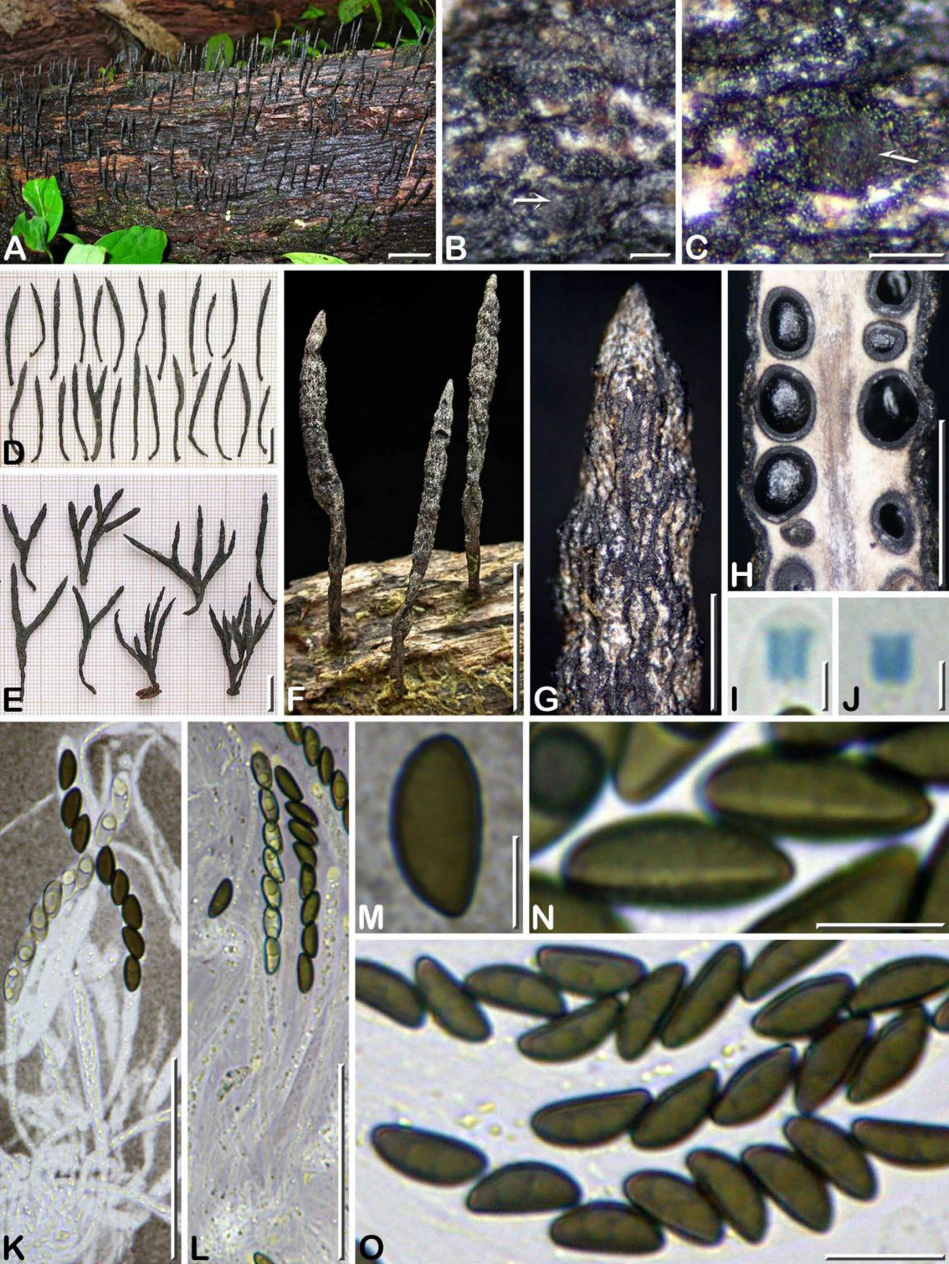
*Xylaria nigrosticta* (**A, C, D, F, G, J, N, O ***GYJF21407* holotype; **B, E ***GYJF21305*; **H, I, K–M ***GYJF19043*). **A** Needle-shaped stromata in the fresh state on a trunk in situ. **B, C** Stromatal surfaces in close-up showing dull black ostioles (arrows) and dense clusters of shiny black granulations. **D** Simple, variously curved needle-shaped stromata. **E** Forked and ramified stromata. **F** Three upright stromata in the dry state. **G** Acuminate sterile stromatal apex showing a white striped superficial layer. **H** Stroma in longitudinal section showing perithecia immersed beneath a thin black crust and a solid white interior with a gray core. **I, J** Ascal apical apparati in Melzer’s reagent. **K, L** Hymenium showing paraphyses and immature and mature asci, in India ink and black Pelikan ink respectively. **M** Ascospore in India ink lacking a sheath or secondary appendages. **N** Ascospores in ventral view showing a germ slit, in Melzer’s reagent. **O** Ascospores in 1% SDS, mostly in side view. Bars in **A** = 20 mm; **B, C** = 0.2 mm; **D–F** = 10 mm; **G, H** = 1 mm; **I, J** = 2 µm; **K, L** = 50 µm; **M, N** = 5 µm; **O** = 10 µm

***Xylaria nigrosticta*** J. Fourn., Y.-M. Ju, H.-M. Hsieh & Lechat, sp. nov. Figure 43, Table 28

**Table 28:** Ascospore and ascal apical apparatus dimensions in eight collections of *X. nigrosticta*, showing a fairly narrow variation range, compared with those of the most closely related species regarding stromatal morphology

Collection numbers	Ascospore measurements with extreme values in parentheses	Q = quotient l/w, *N* = number of measurements	Me = mean values of l/w, Qe = mean quotient l/w	Ascal apical apparatus h × w, *N* = 20
*GYJF19043*	(8.4–)8.7–9.9(−10.5) × (3.2–)3.6–4.0(−4.3) µm	Q = (2.0–)2.2–2.7(−2.8), *N* = 60	Me = 9.3 × 3.8 µm, Qe = 2.4	1.6–2.0 × 1.3–1.6 µm, Me = 1.8 × 1.4 µm
*GYJF19120*	(8.8–)9.1–10.0(−11.0) × (3.3–)3.5–4.2(−4.4) µm	Q = (2.1–)2.2–2.7(−2.9), *N* = 60	Me = 9.6 × 3.9 µm, Qe = 2.5	1.5–1.9 × 1.3–1.5 µm, Me = 1.7 × 1.4 µm
*GYJF21229*	(8.1–)8.8–10.0(−10.5) × (3.4–)3.6–4.3(−4.6) µm	Q = (2.0–)2.1–2.7(−2.8), *N* = 60	Me = 9.4 × 3.9 µm, Qe = 2.4	1.8–2.3 × 1.5–1.8 µm, Me = 2.0 × 1.6 µm
*GYJF21238*	(8.0–)8.4–9.7(−10.3) × (3.5–)3.6–4.3(−4.6) µm	Q = (1.8–) 2.1–2.6 (−2.9), *N* = 60	Me = 9.1 × 3.9 µm, Qe = 2.3	1.8–2.1 × 1.3–1.7 µm, Me = 1.9 × 1.5 µm
*GYJF21242*	(8.2–)8.6–10.0(−10.6) × (3.5–)3.7–4.3(−4.4) µm	Q = (2.0–)2.1–2.6 (−2.9), *N* = 60	Me = 9.3 × 4.0 µm, Qe = 2.4	1.9–2.3 × 1.4–1.6 µm, Me = 2.1 × 1.5 µm
*GYJF21305*	(7.4–)9.0–10.2(−11.1) × (3.4–)3.5–4.1 µm	Q = (1.9–)2.3–2.8(−2.9), *N* = 60	Me = 9.6 × 3.8 µm, Qe = 2.5	1.6–2.0 × 1.4–1.6 µm, Me = 1.9 × 1.5 µm
*GYJF21407* holotype	(8.8–)9.1–10.7(−11.3) × (3.5–)3.7–4.5(−4.7) µm	Q = (2.1–)2.2–2.7(−3.0), *N* = 60	Me = 9.9 × 4.0 µm, Qe = 2.5	1.8–2.1 × 1.5–1.8 µm, Me = 1.9 × 1.7 µm
*GYJF21433*	(8.4–)9.0–10.5(−11.1) × (3.3–)3.6–4.2(−4.4) µm	Q = (2.1–)2.3–2.7(−2.9), *N* = 60	Me = 9.7 × 3.9 µm, Qe = 2.5	1.7–2.0 × 1.4–1.6 µm, Me = 1.8 × 1.5 µm
**Cumulated values**	**(7.4–)8.4–10.7(−11.3) × (3.2–)3.5–4.5(−4.7) µm**	**Q = (1.8–) 2.1–2.8(−3.0), N = 480**	**Me = 9.5 × 3.9 µm, Qe = 2.4**	**1.5–2.3 × 1.3–1.8 µm, Me = 1.9 × 1.5 µm**
*X. pallidocylindracea*	(7.7–)8.1–9.3(−10) × (3.5–)3.8–4.5(4.7–) µm	Q = (1.7–)1.9–2.3(−2.5), *N* = 120	Me = 8.6 × 4.1 µm, Qe = 2.1	
*X. muscandae* holotype (Ju et al. 2016)	10–11 × 4.5–5.0 µm		Me = 10.5 × 4.8 µm, Qe = 2.2	
*X. oligotoma* (this paper)	(7.2–)7.5–9.9(−10.4) × (2.5–)2.9–4.1(−4.3) µm	Q = (1.9–)2.1–3.1(−3.7), *N* = 840	Me = 8.7 × 3.4 µm, Qe = 2.6	

MycoBank MB863173

**Typification**: **FRENCH GUIANA.** Maripasoula, Saül, trail to Monts-La-Fumée, next to a tributary of Crique Queue-Hocco, 3.637173 N, 53.204727 W, ca. 260 m, on dead trunk, 4 Apr 2021, *Fournier, J. GYJF21407* (holotype HAST 147398).

**Etymology**: from Latin *niger* = black and *stictus* = dotted, referring to the densely distributed hemispherical clusters of black granulations on stromatal surface.

**Diagnosis**: Differs from *X. muscandae*, the most resembling species, by a thinner and less felty superficial whitish layer, more densely distributed superficial black granulations, and slightly smaller ascospores 8.4–10.7 × 3.5–4.5 µm vs 10–11 × 4.5–5.0 µm.

**Stromata** forming large colonies on dead trunks, upright, scattered, separate, rarely connate at base, cylindrical to narrowly fusiform with a mucronate sterile apex, simple and terete, occasionally branched and flattened, straight to slightly curved at base, 15–35 mm in total height, the fertile heads 11–30 mm high × 1.0–1.7(−2.6) mm diam, the stipes 5–11 mm high × 0.6–1.2 mm diam; stouter, more flattened and branched stromata up to 35 mm in total height and 3.1 mm wide occur more frequently on dead branches, but both forms may be mixed on the same substrate; surface blackish, with unexposed perithecial contours, coarsely roughened by scattered stripes or scales of a fibrous whitish peeling outer layer, superficially dirty gray and white below, and widely distributed hemispherical, coalescent clusters of shiny black granulations 20–60(−80) µm diam that are present on surface of white stripes and as well on the underlying crust; underlying crust black, leathery, 40–50 µm thick; the stipes ill-defined, black, smooth, finely tomentose turning glabrous with time, longitudinally furrowed, slightly swollen at base; interior solid, spongy, whitish with a grayish core, turning light brown in old stromata. **Perithecia** subglobose to depressed-spherical, 0.5–0.7 mm diam, laterally flattened when in contact. **Ostioles** obtusely conical, occasionally on a slightly raised-discoid base, 100–160 µm diam at base, dull black, often inconspicuous or obscured by black granulations.

**Paraphyses** hyphal, thin-walled, 2.0–2.5 µm wide at base, tapering to 1.0–1.5 µm wide above asci, with oily contents, embedded in mucilaginous material. **Asci** cylindrical, with (6–)8 slightly overlapping uniseriately, occasionally biseriately arranged ascospores, the spore-bearing parts 54–65 × 5.5–6.5 µm, the stipes 56–72 µm long, with apical apparatus 1.5–2.3 × 1.3–1.8 µm (Me = 1.9 × 1.5 µm, *N* = 160), short-cylindrical to slightly tubular, with a faint upper rim, bluing in Melzer’s reagent. **Ascospores** (7.4–)8.4–10.7(−11.3) × (3.2–)3.5–4.5(−4.7) µm, Q = (1.8–) 2.1–2.8(−3.0), *N* = 480 (Me = 9.5 × 3.9 µm, Qe = 2.4), ellipsoid-inequilateral with narrowly to broadly rounded ends, ventrally flat to occasionally slightly concave, brown to dark brown, unicellular, with a conspicuous, straight, longitudinally oriented germ slit almost spore-length on the ventral side, without sheath or appendages; epispore smooth.

**Asexual morph** on the natural substrate not observed.

**Other specimens examined (paratypes)**: **FRENCH GUIANA.** Maripasoula, Saül, shortcut toward the airfield, 3.622159 N, 53.204166 W, disturbed secondary rainforest, on dead blackened and mossy trunk, 20 Jun 2019, *Fournier, J. GYJF19120* (HAST 147392); Maripasoula, Saül, trail head of Sentier des Gros Arbres, 3.6201 N, 53.207989 W, on dead corticated trunk, 29 Mar 2021, *Fournier, J. GYJF21242* (HAST 147393); Maripasoula, Saül, trail to Monts-La-Fumée, next to a tributary of Crique Queue-Hocco, 3.637173 N, 53.204727 W, ca. 260 m, on dead decorticated wood, 4 Apr 2021, *Fournier, J. GYJF21433* (HAST 147394); Maripasoula, Saül, shortcut toward the airfield, 3.622159 N, 53.204166 W, disturbed secondary rainforest, on dead wood, 17 Jun 2019, *Fournier, J. GYJF19043* (HAST 147395); Maripasoula, Saül, trail head of Sentier Roche-Bateau, Crique-Cochon, 3.625994 N, 53.1955 W, 210–220 m, disturbed secondary rainforest, on dead corticated trunk, 29 Mar 2021, *Fournier, J. GYJF21305* (HAST 147396); Maripasoula, Saül, trail head of Sentier des Gros Arbres, 3.6201 N, 53.207989 W, on dead decorticated branch, 28 Mar 2021, *Fournier, J. GYJF21229* (HAST 147397); Maripasoula, Saül, shortcut toward the airfield, 3.622852 N, 53.205768 W, 210–220 m, disturbed secondary rainforest, on dead trunk, 29 Mar 2021, *Fournier, J. GYJF21238* (HAST 147399).

**Known distribution**: French Guiana.

**Comments**: In the field, *X. nigrosticta* can hardly be distinguished from the numerous other *Xylaria* species with small-sized, cylindrical and acuminate stromata. It can be diagnosed by the combination of dense, rounded clusters of shiny black granulations overlying all over the stromatal surface where remnants of a bipartite dirty gray and white fibrous outer layer split into elongate stripes, a thin leathery crust, obtusely conical ostioles, and brown inequilateral ascospores averaging 9.5 × 3.9 µm with a conspicuous straight germ slit. The stromata vary from narrowly cylindrical and unbranched, most often in dense colonies on dead trunks, to more flattened, stouter and highly branched when occurring on dead branches.

*Xylaria nigrosticta* must be compared with *X. muscandae* C. G. Lloyd, known from Sri Lanka, Martinique, and Panama (Ju et al. 2016; Fournier et al. 2020), which shares a similar habit and a grayish peeling outer layer associated with superficial black granulations. *Xylaria muscandae* can be distinguished from *X. nigrosticta* by a thicker, more felty outer layer, more loosely distributed black granulations on the surface and slightly larger ascospores 10–11 × 4.5–5.0 µm (10–11.5 × 3.6–4.4 µm for the Neotropical collections). This is supported by a 95.7% of ITS similarity between *X. nigrosticta* and a collection of *X. muscandae* from Martinique.

*X.* *oligotoma* is another resembling species, commonly found in the same sites, which likewise features small acuminate stromata with a surface roughened by black granulations. It differs by more nodulose stromata due to slightly exposed perithecial contours, a light brown to tan peeling outer layer, black granulations arranged in longitudinal ridges, and slightly smaller ascospores averaging 8.7 × 3.4 µm (this paper).

*Xylaria pallidocylindracea* J. Fourn. & Lechat resembles *X. nigrosticta* but can be separated by less sharply mucronate stromata with a more carbonaceous crust overlain by a pure white striped layer, and dark brown ascospores averaging 8.6 × 4.1 µm.

*Xylaria piloides*, described elsewhere herein, is phylogenetically the closest species in our phylogram. It can be readily distinguished from *X. nigrosticta* by its larger fusiform ascospores averaging 16.1 × 4.9 µm with a short ventral germ slit.

**Fig. 44:**
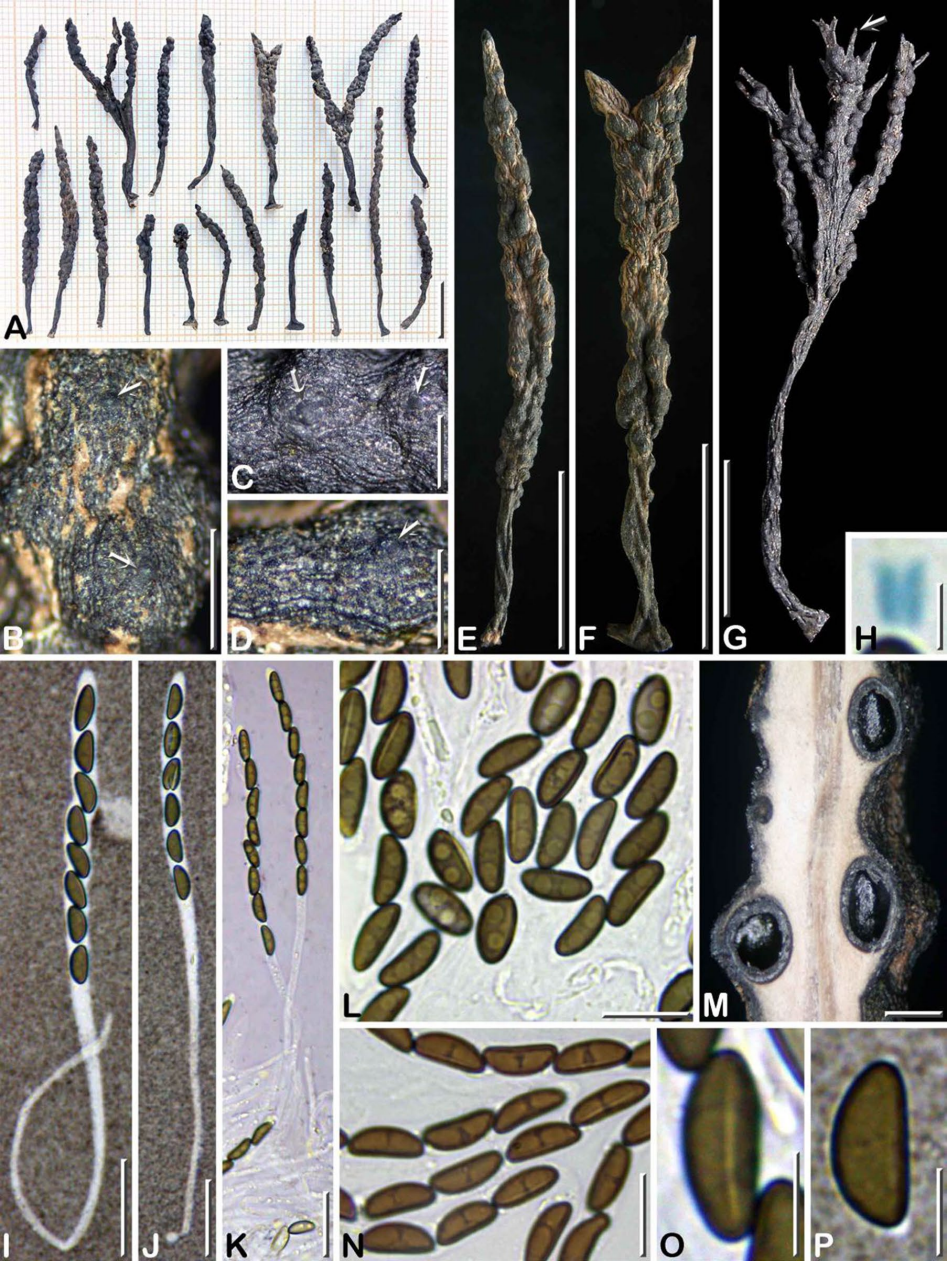
*Xylaria oligotoma* (**A–F, H–M, O, P ***GYJF19208*; **G, N ***CLL9040*). **A** Simple and diversely branched stromata. **B–D** Stromatal surface in close-up showing ostioles (arrows), superficial granulations distributed in linear to sinuous ridges and remnants of a tan peeling outer layer. **E, F** Nodulose stromata with remnants of a tan peeling outer layer. **G** Ramified stroma with multiple apical spines (arrow). **H** Ascal apical apparatus in Melzer’s reagent. **I, J** Eight- and six-spored asci in India ink. **K** Eight-spored asci in black Pelikan ink. **L, N** Ascospores in 1% SDS, respectively in the fresh and in the dry state, some showing a germ slit. **M** Stroma in vertical section showing scattered perithecia beneath a thin leathery crust and a white interior with a grayish core. **O** Ascospore in ventral view showing a germ slit, in 1% SDS. **P** Ascospore in India ink showing bipolar secondary appendages. Bars in **A, E–G** = 10 mm; **B–D**, **M** = 0.5 mm; **H** = 2 µm; **I–K** = 20 µm; **L, N** = 10 µm; **O**, **P** = 5 µm

***Xylaria oligotoma*** Sacc. & Paol., Atti del Reale Istituto Veneto di Scienze Lettere ed Arti, sér. VI, 6: 404. 1888. Figure 44, Table 29

**Table 29:** Ascospore dimensions in fifteen collections of *X. oligotoma* showing a fairly wide range of intraspecific variations

Collection numbers	Ascospore measurements with extreme values in parentheses	Q = quotient l/w, *N* = number of measurements	Me = mean values of l/w, Qe = mean quotient l/w
*CLL7031*	(7.7–)8.1–9.2(−9.7) × (2.9–)3.2–3.6(−3.8) µm	Q = (2.2–)2.4–2.8(−3.2) *N* = 60	Me = 8.6 × 3.4 µm, Qe = 2.6
*CLL9004*	(8.1–)8.3–9.1(−9.6) × (2.8–)3.1–3.7(−3.8) µm	Q = (2.2–)2.4–2.9(−3.0) *N* = 60	Me = 8.7 × 3.4 µm, Qe = 2.6
*CLL9040*	(8.3–)8.6–9.6(−10.0) × (3.1–)3.2–3.6(−3.9) µm	Q = (2.3–)2.5–2.8(−3.0), *N* = 60 (	Me = 9.0 × 3.4 µm, Qe = 2.7
*GYJF18045*	(7.3–)8.0–9.2(−9.7) × (2.6–)2.9–3.4(−3.8) µm	Q = (2.4–)2.5–3.0(−3.3) *N* = 60	Me = 8.6 × 3.2 µm, Qe = 2.7
*GYJF19200*	(7.6–)8.1–9.1(−9.7) × (2.7–)3.0–3.4(−3.6) µm	Q = (2.3–)2.5–3.0(−3.3) *N* = 60	Me = 8.6 × 3.2 µm, Qe = 2.7
*GYJF19208*	(7.6–)8.1–9(−9.7) × (3–)3.1–4.1(−4.3) µm	Q = (1.9–)2.1–2.7(−3.1), *N* = 60	Me = 8.6 × 3.6 µm, Qe = 2.4
*GYJF21004*	(8.0–)8.6–9.9(−10.4) × (3.0–)3.2–3.6(−3.8) µm	Q = (2.2–)2.4–2.9(−3.3), *N* = 60	Me = 9.2 × 3.4 µm, Qe = 2.7
*GYJF21055*	(7.8–)8.2–9.3(−9.7) × (3.0–)3.2–3.7(−3.9) µm	Q = (2.2–)2.3–2.8(−3.2), *N* = 60	Me = 8.7 × 3.4 µm, Qe = 2.5
*GYJF21059*	(7.3–)7.9–9.2 (−9.6) × (2.8–)2.9–3.5(−3.6) µm	Q = (2.3–)2.4–3.0(−3.1), *N* = 60	Me = 8.5 × 3.3 µm, Qe = 2.6
*GYJF21060*	(7.7–)8.0–8.9(−9.7) × (2.9–)3.1–3.6(−4.1) µm	Q = (2.2–)2.4–2.8(−2.9), *N* = 60	Me = 8.5 × 3.3 µm, Qe = 2.6
*GYJF21063*	(7.5–)7.8–8.8(−9.7) × (3.0–)3.1–3.5–3.7) µm	Q = (2.2–)2.3–2.7(−2.8), *N* = 60	Me = 8.2 × 3.3 µm, Qe = 2.5
*GYJF21135-2*	(8.0–)8.4–9.7(−10.3) × (3.1–)3.3–3.8(−3.9) µm	Q = (2.2–)2.4–2.8(−3.0), *N* = 60	Me = 9.0 × 3.5 µm, Qe = 2.6
*GYJF21265*	(7.2–)7.5–8.9(−9.8) × (2.8–)3.0–3.5(−3.9) µm	Q = (2.1–)2.2–2.8(−3.1), *N* = 60	Me = 8.2 × 3.3 µm, Qe = 2.5
*GYJF21322*	(7.8–)7.9–8.8(−9.2) × (0.03–)3.2–3.6(−3.8) µm	Q = (2.1–)2.3–2.7(−2.8), *N* = 60	Me = 8.4 × 3.4 µm, Qe = 2.5
*GYJF21412*	(8.2–)8.6–9.9(−10.4) × (2.5–)3.1–3.5(−3.6) µm	Q = (2.4–)2.6–3.1(−3.7), *N* = 60	Me = 9.2 × 3.2 µm, Qe = 2.8
**Cumulated values**	**(7.2–)7.5–9.9(−10.4) × (2.5–)2.9–4.1(−4.3) µm**	**Q = (1.9–)2.1–3.1(−3.7), N = 900**	**Me = 8.7 × 3.4 µm, Qe = 2.6**

**Stromata** upright, separate to clustered, cylindrical to narrowly fusiform with a sharply mucronate, frequently broken sterile apex, typically simple and terete, occasionally branched and flattened, straight to slightly curved, (8–)14–45 mm in total height, the fertile head 5–35 mm high × 1.2–3 mm diam, the stipe 3–22 mm high × 0.8–1.5(−2.4) mm wide; surface blackish, nodulose with strongly exposed perithecial contours, roughened by scattered elongated scales or stripes of a fibrous brownish gray to tan or orange-brown peeling outer layer and widely distributed shiny black superficial black granulations; underlying crust black, leathery, 40–50(−80) µm thick, superficially roughened by longitudinal, straight to sinuous low ridges frequently bearing shiny black granulations; the stipes mostly ill-defined, black, smooth to finely granular, terete, straight to curved, glabrous, slightly swollen at base; interior solid, spongy, whitish with a narrow grayish core. **Perithecia** subglobose to depressed-spherical, 0.65–0.85 mm diam, rarely in contact. **Ostioles** often inconspicuous, obtusely conical, dull black, 80–100 µm diam at base, rarely on a discoid base 120 µm diam.

**Paraphyses** hyphal, thin-walled, 2–3 µm wide at base, tapering to 1–1.5 µm wide above asci, minutely guttulate, embedded in mucilaginous material. **Asci** cylindrical, with (6–)8 slightly overlapping uniseriately arranged ascospores, the spore-bearing parts 58–67 × 4.5–5.5 µm, the stipes 80–100 µm long, with apical apparatus 1.4–2.0 × 1.1–1.6 µm (Me = 1.7 × 1.3 µm, *N* = 40), short-cylindrical to slightly tubular, with a faint upper rim, bluing in Melzer’s reagent. **Ascospores** (7.2–)7.5–9.9(−10.4) × (2.5–)2.9–4.1(−4.3) µm, Q = (1.9–)2.1–3.1(−3.7), *N* = 900 (Me = 8.7 × 3.4 µm, Qe = 2.6), ellipsoid-inequilateral to suballantoid with broadly rounded ends, medium brown, unicellular, with a conspicuous, straight, longitudinally oriented germ slit slightly less than spore-length on the ventral side, with 1(−2) minute polar secondary appendages visible in India ink, occasionally associated with a narrowly appressed sheath surrounding the whole spore; epispore smooth.

**Asexual morph** on the natural substrate not observed.

**Specimens examined**: **FRENCH GUIANA.** Roura, Cacao, Camp Bonaventure, 4.323044 N, 52.339371 W, 59 m, disturbed hygrophilic rainforest, on dead decorticated branch, 20 Mar 2021, *Fournier, J. GYJF21004* (HAST 147400); Roura, Cacao, Camp Bonaventure, 4.326815 N, 52.341431 W, 60–65 m, disturbed hygrophilic rainforest, on dead blackened wood, 22 Mar 2021, *Fournier, J. GYJF21061* (HAST 147401); Roura, Cacao, Camp Bonaventure, 4.326815 N, 52.341431 W, 60–65 m, disturbed hygrophilic rainforest, on dead blackened wood, 22 Mar 2021, *Fournier, J. GYJF21060* (HAST 147402); Roura, Cacao, Camp Bonaventure, 4.326815 N, 52.341431 W, 60–65 m, disturbed hygrophilic rainforest, on dead decorticated wood, 22 Mar 2021, *Fournier, J. GYJF21063* (HAST 147403); Roura, Cacao, Camp Bonaventure, 4.330514 N, 52.338095 W, disturbed hygrophilic rainforest, on dead blackened wood, 24 Mar 2021, *Fournier, J. GYJF21113* (HAST 147404); Maripasoula, Saül, trail head toward Roche-Bateau, 3.620498 N, 53.199309 W, disturbed secondary rainforest, on dead blackened wood, 22 Jun 2019, *Fournier, J. GYJF19200* (HAST 147405); Roura, Cacao, Camp Bonaventure, 4.326815 N, 52.341431 W, 60–65 m, disturbed hygrophilic rainforest, on dead decorticated branch, 22 Mar 2021, *Fournier, J. GYJF21059* (HAST 147406); Roura, Cacao, Camp Bonaventure, 4.326815 N, 52.341431 W, 60–65 m, disturbed hygrophilic rainforest, on dead decorticated branch, 22 Mar 2021, *Fournier, J. GYJF21055* (HAST 147407); Roura, Cacao, Camp Bonaventure, 4.323044 N, 52.339371 W, 59 m, on blackened wood, 20 Mar 2021, *Fournier, J. GYJF21022* (HAST 147408); Régina, vicinity of airstrip, 4.316743 N, 52.131763 W, disturbed marshy rainforest, on dead wood, 22 Apr 2009, *Lechat, C. CLL9040* (HAST 147409); Roura, Cacao, Camp Bonaventure, 4.323044 N, 52.339371 W, 59 m, on blackened wood, 20 Mar 2021, *Fournier, J. GYJF21010* (HAST 147410); Maripasoula, Saül, trail head to Sentier des Gros Arbres, 3.620839 N, 53.208536 W, ca. 210 m, disturbed secondary rainforest, on dead wood, 26 Mar 2021, *Fournier, J. GYJF21135-2* (HAST 147411); Sinnamary, Saint-Elie, Saint-Elie botanical trail, 5.292020 N, 53.05262 W, mesophilic rainforest, on dead corticated branch, 25 Feb 2007, *Lechat, C. CLL7031* (HAST 147412); Sinnamary, Saint-Elie, Saint-Elie botanical trail, 5.292020 N, 53.05262 W, mesophilic rainforest, on dead corticated branch, 16 Apr 2009, *Lechat, C. CLL9004* (HAST 147413); Maripasoula, Saül, trail head toward Roche-Bateau, Crique-Cochon, 3.625994 N, 53.1955 W, disturbed secondary rainforest, on dead decorticated branch, 31 Mar 2021, *Fournier, J. GYJF21322* (HAST 147414); Maripasoula, Saül, trail head toward Roche-Bateau, 3.620498 N, 53.199309 W, disturbed secondary rainforest, on dead blackened branch, 22 Jun 2019, *Fournier, J. GYJF19208* (HAST 147415); Maripasoula, Saül, trail to Monts-La Fumée, tributary of Crique Queue-Hocco, 3.637173 N, 53.204727 W, ca. 260 m, disturbed mesophilic rainforest, on dead corticated branch, 4 Apr 2021, *Fournier, J. & Thonnel, A. GYJF21412* (HAST 147416); Maripasoula, Saül, trail head toward Roche-Bateau, 3.62056 N, 53.199899 W, 240 m, disturbed secondary rainforest, on dead blackened wood, 30 Mar 2021, *Fournier, J. GYJF21265* (HAST 147417); Maripasoula, Saül, trail head toward Monts-La-Fumée, 3.629382 N, 53.206831 W, disturbed secondary rainforest, on dead blackened wood, 21 Aug 2018, *Fournier, J. GYJF18045* (HAST 147418).

**Known distribution**: French Guiana (this paper); Malaysia (Saccardo and Paoletti 1888); Papua New Guinea (Dennis 1974; Van der Gucht 1995).

**Comments**: *Xylaria oligotoma* is commonly encountered in French Guiana, but not always readily distinguished from other species with needle-shaped stromata. It is diagnosed by narrowly cylindrical, mostly simple, leathery stromata with sharply a pointed apex, with a nodulose outline due to exposed perithecial contours. Its surface is distinctive in being roughened by tan to orange-brown stripes of a peeling outer layer and dense minute black granulations frequently arranged in linear or sinuous ridges. Its long-stipitate asci and ellipsoid-inequilateral ascospores averaging 8.7 × 3.4 µm, with broadly rounded ends and a germ slit slightly less than spore-length, further characterize this species. The different interpretations of *X. oligotoma* found in the literature were summarized by Fournier et al. (2020).

The stromatal illustrations of *X. pumila* (Fr.) Mont. provided by Joly (1968) closely resemble *X*. *oligotoma*. However, the three parts of *Leprieur 1212* that we examined [**FRENCH GUIANA.** on wood, *Leprieur, F. R. 1212*, as *X. pumila* (PC ex Leprieur herb., PC ex Montagne herb. 10124 & 10125)] correspond to *X. coccophora*. Unfortunately, the identity of *Sphaeria pumila* remains unsettled, as Fries’ original material could not be located at UPS.

*Xylaria nigrosticta* (this paper) is a species frequently encountered around Saül, featuring similar stromata that are narrowly cylindrical and have a densely granular surface. It differs mainly from *X. oligotoma* by stromata lacking exposed perithecial contours and coated with remnants of a white peeling outer layer, and by darker brown, slightly larger ascospores averaging 9.5 × 3.9 µm. Phylogenetically, *X*. *oligotoma*, *X. pallidocylindracea*, and *X*. *vinosa* form a clade next to that of *X*. *coccophora,* while *X. nigrosticta*, although morphologically similar, appears more distantly related.

**Fig. 45:**
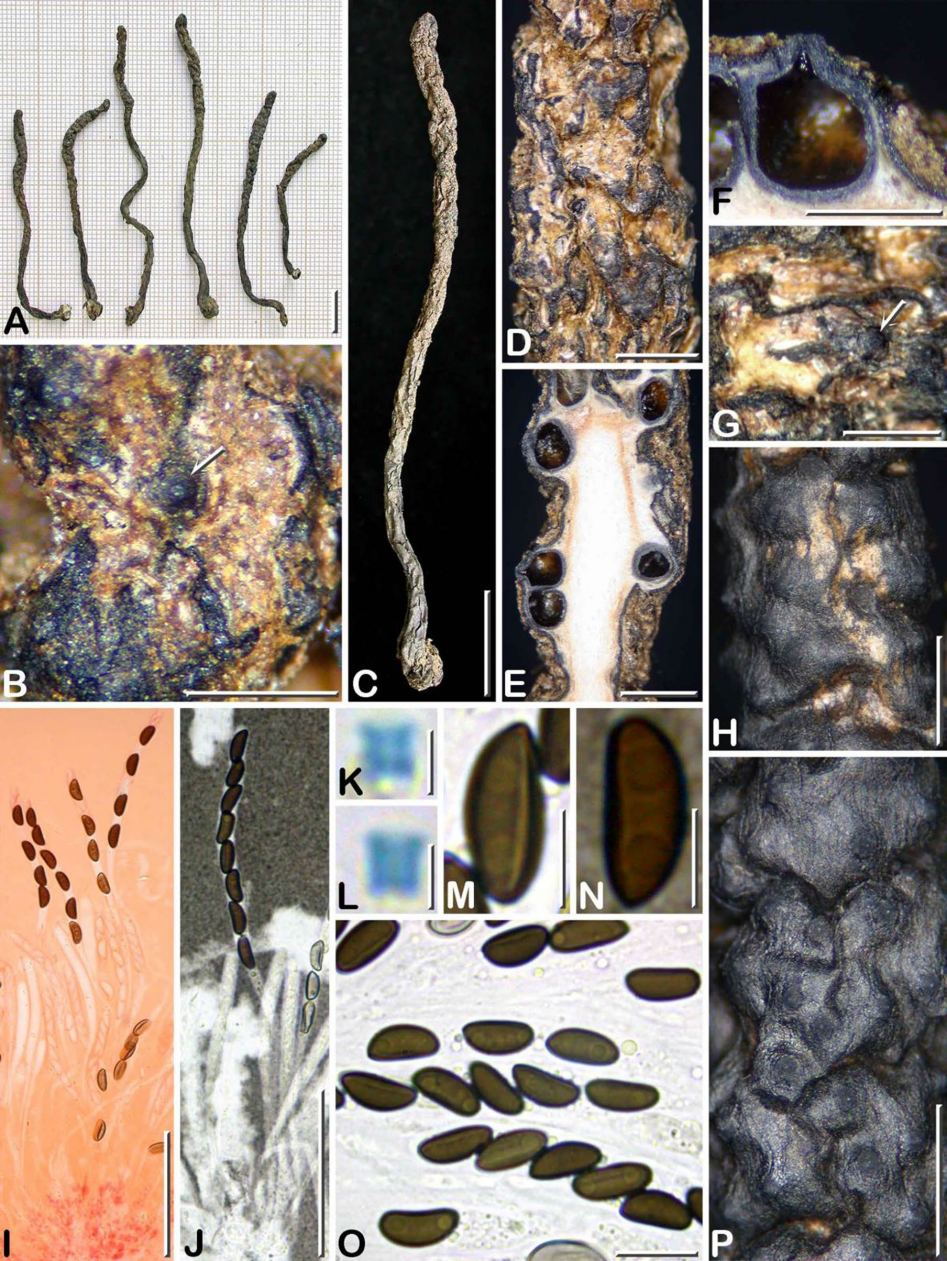
*Xylaria orichalcea* (**A–G, J, K, M–O ***GYJF19225* holotype; **H, I, L, P ***CLL5534*). **A** Stromata in the dry state. **B, G** Stromatal surface in close-up showing ostioles (arrows) surrounded by golden-yellow scaly coating. **C** Barely mature stroma in the dry state. **D** Stromatal surface of a barely mature stroma. **E** Stroma in longitudinal section showing perithecia immersed beneath a thin black crust and white interior. **F** Perithecium in vertical section. **H** Sparse golden-yellow scales remaining on stromatal surface of a mature stroma. **I** Few-spored asci, in Congo red in 1% SDS. **J** Mature ascus in India ink. **K, L** Ascal apical apparati in Melzer’s reagent. **M** Ascospore in ventral view showing a germ slit, in 1% SDS. **N** Ascospore in India ink showing a lack of sheath or polar secondary appendages. **O** Ascospores in 1% SDS. **P** Deeply wrinkled surface of a mature stroma showing ostioles with a discoid base. Bars in **A, C** = 10 mm; **B, F, G** = 0.5 mm; **D, E, H, P** = 1 mm; **I, J** = 50 µm; **K, L** = 2 µm; **M, N** = 5 µm; **O** = 10 µm

***Xylaria orichalcea*** J. Fourn., Y.-M. Ju, H.-M. Hsieh & Lechat, sp. nov. Figure 45, Table 30

**Table 30:** Ascospore and ascal apical apparatus dimensions in one collection of *X. orichalcea* from French Guiana and one from Martinique showing their variation range, compared with those of *X. scutulata* and *X. spegazziniana*

Collection numbers	Ascospore measurements with extreme values in parentheses	Q = quotient l/w, *N* = number of measurements	Me = mean values of l/w, Qe = mean quotient l/w	Ascal apical apparatus h × w, *N* = 25
*GYJF19225*	(7.6–)8.0–9.9(−10.2) × (3.3–)3.7–4.3(−4.5) µm	Q = (1.8–)2.0–2.5(−2.9), *N* = 60	Me = 8.9 × 4.0 µm, Qe = 2.2	1.8–2.1 × 1.4–1.7 µm, Me = 1.9 × 1.6 µm
*FWI CLL5534*	(7.7–)8.4–9.7(−10.4) × (3.7–)3.9–4.5(−4.8) µm	Q = (1.9–)2.0–2.2(−2.3), *N* = 60	Me = 9.1 × 4.2 µm, Qe = 2.1	1.9–2.2 × 1.5–1.9 µm, Me = 2.0 × 1.8 µm
**Cumulated values**	**(7.6–)8.0–9.9(−10.4) × (3.3–)3.7–4.5(−4.8) µm**	**Q = (1.8–)2.0–2.5(−2.9), N = 120**	**Me = 9.0 × 4.1 µm, Qe = 2.2**	**1.8–2.2 × 1.4–1.9 µm, Me = 2.0 × 1.7 µm**
*X. spegazziniana* (as *X. multiplex* var. *microsperma*) (Fournier et al. 2020)	(8.1–)8.9–10.8(−11.8) × (3.3–)3.7–4.5(−4.8) µm	Q = (1.9–)2.2–2.9(−3), *N* = 480	Me = 9.7 × 3.9 µm, Qe = 2.4	1.7–2.2 × 1.4–1.8 µm, Me = 2.0 × 1.6 µm
*X. scutulata*	(6.9–)7.3–9.7(−10.1) × (3.2–)3.4–4.5(−4.7) µm	Q = (1.6–)1.8–2.6(−2.9), *N* = 720	Me = 8.4 × 4.0 µm, Qe = 2.1	1.6–2.3 × 1.3–1.7 µm, Me = 1.9 × 1.5 µm

MycoBank MB863174

**Typification**: **FRENCH GUIANA.** Maripasoula, Saül, trail toward Roche-Bateau, Crique-Cochon, 3.618752 N, 53.198359 W, disturbed secondary rainforest, emerging from the buried part of wooden steps in muddy part of the trail, 23 Jun 2019, *Fournier, J. GYJF19225* (holotype HAST 147419).

**Etymology**: From Latin *orichalcum* = a legendary metallic alloy close to brass, referring to the vanishing, dull golden-yellow color of the pellicle on the stromatal surface.

**Diagnosis**: Differs from *X. spegazziniana*, which has similar deeply wrinkled stromata, by a vanishing golden yellow brown superficial coating and slightly smaller, more pigmented ascospores with broadly rounded ends and a germ slit consistently on the ventral side.

**Stromata** in a small group, upright, separate, subcylindrical to slightly clavate, simple to rarely branched, terete to flattened, straight to slightly curved, with broadly rounded fertile apices, stipitate, 32–68 mm in total height, the fertile head 15–33 mm high × 2–5 mm wide, shrinking upon drying, eventually becoming hollow and splitting lengthwise; surface nodulose to cerebriform with deep circumferential and oblique constrictions appearing upon drying, with a dull golden-yellow to dull yellow brown outer layer cracked into large, irregular, vanishing scales, with perithecial contours unexposed to slightly exposed; underlying crust leathery, black, 35–40 µm thick, superficially roughened by fine cracks and sparse longitudinal furrows; the stipes ill-defined, (3.0–)17–45 mm high × 1.5–4.0 mm wide, blackish brown, straight to most often contorted, terete to flattened, puckered to cracked, glabrous, the very base slightly swollen and tomentose; interior solid, spongy to fibrous, white to cream-colored, occasionally with a thin light orange line beneath the perithecia, eventually disintegrating. **Perithecia** subglobose to slightly depressed-spherical 0.45–0.6 mm diam. **Ostioles** finely conic-papillate, on a low, dull black discoid base (120–)180–200 µm diam.

**Paraphyses** hyphal, thin-walled, sparse, 4–6 µm wide at base, tapering to 1.5–2 µm wide above asci, embedded in mucilaginous material. **Asci** cylindrical, with (4–6–)8 slightly overlapping uniseriately arranged ascospores, the spore-bearing parts (50–)58–65 × 5.0–5.5 µm, the stipes 60–90 µm long, with apical apparatus 1.8–2.2 × 1.2–1.9 µm (Me = 2.0 × 1.6 µm, *N* = 70), short-cylindrical to quadrate with a faint upper rim, bluing in Melzer’s reagent. **Ascospores** (7.6–)8.0–9.9(−10.4) × (3.3–)3.7–4.5(−4.8) µm, Q = (1.8–)2.0–2.5(−2.9), *N* = 120 (Me = 9.0 × 4.1 µm, Qe = 2.2), ellipsoid-inequilateral with mostly broadly rounded ends, ventrally flat or slightly concave, brown to dark brown with olivaceous tone when fresh, unicellular, with a conspicuous, straight, longitudinally oriented germ slit almost spore-length on the ventral side, without sheath or appendages; epispore smooth.

**Asexual morph** on the natural substrate not observed.

**Other specimen examined (paratype): FRENCH WEST INDIES.** Martinique, Schoelcher, Rivière Duclos, Fontaine Didier, meso- to hygrophilic rainforest, on dead blackened wood, 4 Dec 2005, *Lechat, C. CLL5534* (HAST 145161).

**Known distribution**: French Guiana; Martinique.

**Comments**: *Xylaria orichalcea* is diagnosed by narrowly subcylindrical stromata with an obtuse fertile apex, the surface coated with a golden yellow brown outer layer that cracks into large scales and becomes strongly nodulose to cerebriform upon drying. Additional differential characters are ostioles on a wide discoid base and brown to dark brown ascospores averaging 9.0 × 4.1 µm with broadly rounded ends. Typically, the outer layer vanishes at maturity, but traces can still be detected on the black stromatal surface, especially at the bottom of deep constrictions. Comparison of ITS sequences showed a 99.84% similarity with a previously unnamed collection from Martinique (*Xylaria* sp. *CLL5534*) (Fournier et al. 2020), which differs from the Guianese collection by more carbonaceous stromata splitting lengthwise upon drying but is otherwise identical.

*Xylaria orichalcea* resembles *X. scutulata* and is phylogenetically closely related to it; the latter is described elsewhere herein and was collected in the same area. *Xylaria scutulata* differs by a white to cream-colored persistent outer layer cracked into a reticulate to mesh-like pattern, smaller and less wrinkled stromata, and less conspicuous ostioles. Microscopic characters are highly similar in both species and do not allow reliable separation.

A comparison with *X. spegazziniana*, which likewise features stromata deeply shrinking upon drying, shows the latter differs by lacking a golden brown outer layer and by having smaller ostioles lacking a discoid base. Its slightly longer ascospores (Table 30) are less pigmented and narrowly ended, and the germ slit is located either on the dorsal or ventral side (Fournier et al. 2020).

**Fig. 46:**
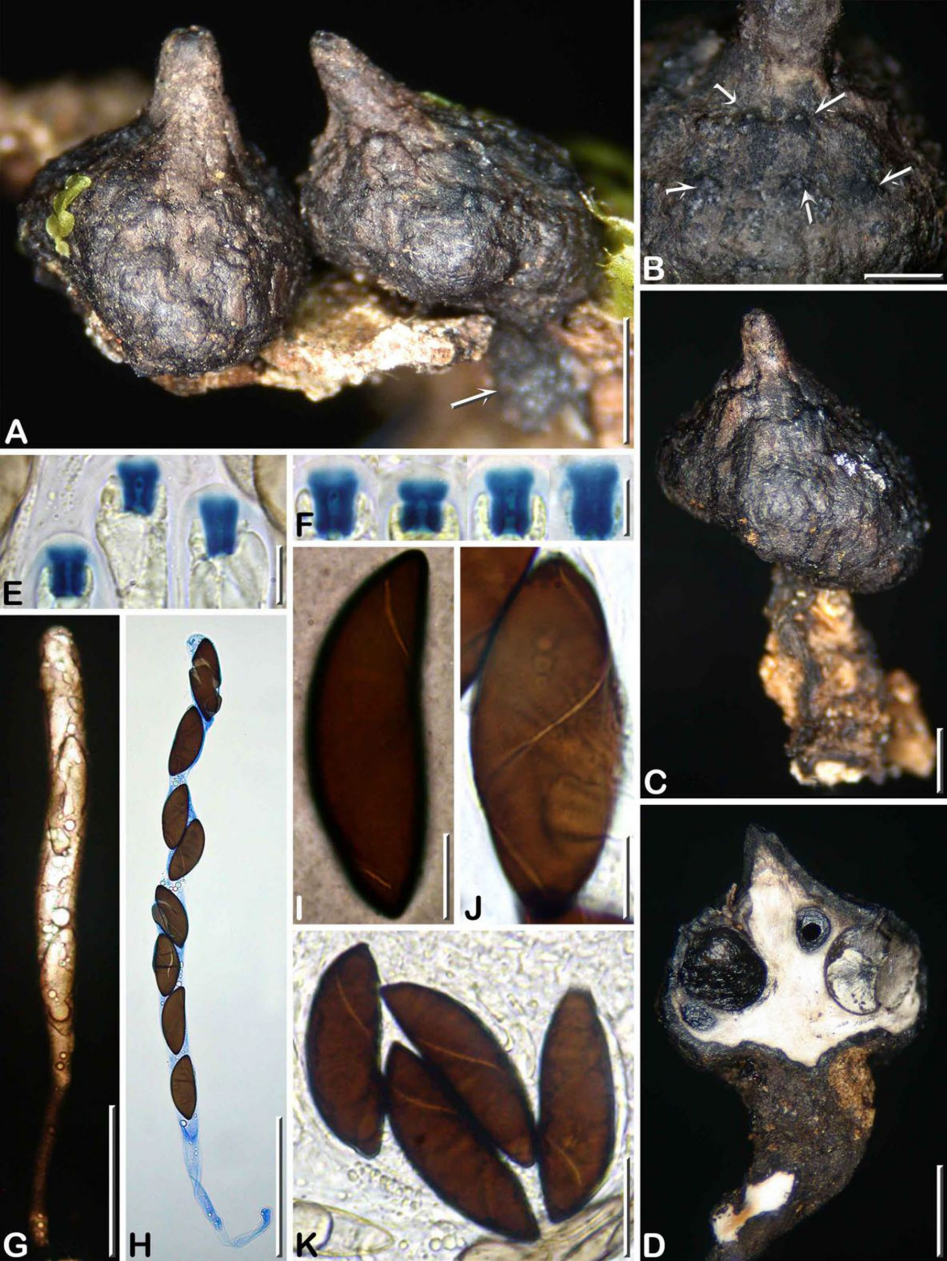
*Xylaria oxycephala* (*GYJF18018* holotype). **A** Two adjacent fertile heads erumpent from bark, one with a short stipe visible beneath the bark (arrow). **B** Stromatal apex in close-up showing ostioles distributed in upper half (arrows). **C** Stipitate stroma in side view. **D** Stroma in longitudinal section. **E, F** Ascal apical apparati in Melzer’s reagent. **G** Immature ascus in India ink. **H** Mature ascus with some damaged ascospores, in blue Pelikan ink. **I** Ascospore in side view in India ink showing a germ slit almost reaching the ends and absence of sheath or appendages. **J, K** Variously oriented ascospores in 1% SDS, showing long spiraling germ slits. Bars in **A, C, D** = 1 mm; **B** = 0.5 mm; **E, F, I, J** = 10 µm; **G, H** = 100 µm; **K** = 20 µm

***Xylaria oxycephala*** J. Fourn., Y.-M. Ju, H.-M. Hsieh & Lechat, sp. nov. Figure 46

MycoBank MB863175

**Typification**: **FRENCH GUIANA.** Maripasoula, Saül, edge of the forest next to Carbets du bord, 3.623598 N, 53.210338 W, at the base of dead corticated standing shrub, 20 Aug 2018, *Fournier, J. GYJF18018* (holotype HAST 147420).

**Etymology**: From Greek $\mathop o\limits^,$*ξύς* (*oxús*) = pointed and *κεφαλή* (*kephalê*) = head, referring to the globose fertile head topped by a pointed sterile extension.

**Diagnosis**: Differs from known penzigioid *Xylaria* species by the combination of subglobose, stipitate stromata with an apical pointed sterile extension and ascospores 40.0–48.7 × 12.3–14.6 µm with a spiraling germ slit.

**Stromata** scattered, erumpent from bark, isolated or fused by two, appearing sitting on the bark but borne on a well-defined stipe developing beneath the bark (arrow on image A). Fertile head subglobose to irregularly depressed-spherical, few-peritheciate, 2.8–4 mm diam × 2.8–4.7 mm high, including a conspicuous central pointed apex 0.65–1.3 mm high. Stipe 1–3(−8) mm high × 0.8–2 mm wide, black, smooth, glabrous. Surface black, with shallow cracks and a persistent, horny, tan outer layer coating the apex and splitting into large scales on upper sides; perithecial contours unexposed; crust carbonaceous, 100–120 µm thick, up to 170 µm thick at the apex; interior white, fibrous, persistent. **Perithecia** subglobose, 0.6–1.1 mm diam. **Ostioles** barely papillate, black most often inconspicuous, located in upper half of the stroma.

**Paraphyses** copious, hyphal, remotely septate, thin-walled, 6–8 µm wide at base, tapering to 1.5–2.5 µm wide above asci, embedded in mucilaginous material. **Asci** cylindrical, short-stipitate, with (6–)8 overlapping uniseriately arranged ascospores, the spore-bearing parts 227–240 × 21.5–25 µm, the stipes 65–133 µm long, with apical apparatus tubular, often apically flared, with a prominent obtuse apical rim, 6.9–8.8 × 4.6–5.7 µm (Me = 7.8 × 5.2 µm, *N* = 20), strongly bluing in Melzer’s reagent. **Ascospores** (37.9–)40.0–48.7(−51.6) × (11.7–)12.3–14.6(−15.9) µm, Q = (2.8–)2.9–3.7(−4.2), *N* = 60 (Me = 44.9 × 13.5 µm, Qe = 3.3), broadly fusoid, strongly inequilateral, straight, ventrally flat to slightly concave, rarely faintly convex, with narrowly rounded to subacute, occasionally pinched ends, dark brown, with a long spiraling germ slit on the ventral side, almost reaching the ends. No sheath or appendages detected in India ink.

**Asexual morph** on the natural substrate not observed.

**Known distribution**: French Guiana, known only from the holotype collection.

**Comments**: *Xylaria oxycephala* is diagnosed by small, shortly stipitate, subglobose, carbonaceous stromata with a stout conical apex overlain by a tan horny layer splitting downwards into large, irregular plaques. This stromatal configuration resembles that of some penzigioid species related to *X. arbuscula*, such as *X*. *xylarioides* (Speg.) Hladki & A. I. Romero, from which it differs by huge ascospores averaging 44.9 × 13.5 µm with a long spiraling germ slit. *Xylaria gigaspora*, described elsewhere herein, features ascospores in the same size range but differs by stromatal morphology and a short, straight to oblique germ slit.

Phylogenetic analyses show a strong affinity with *X. kegeliana*, with which *X*. *oxycephala* also shares a long spiraling germ slit on the ascospores, but from which it can be distinguished by stromatal shape and color, and by ascospores about twice as large.

*Xylaria stilbohypoxyloides* Hladki & A.I. Romero, known from Argentina, is reminiscent of *X. oxycephala* in stromatal outline, with stipes emerging through the bark. It differs mainly by smaller ascospores 28–32.5 × 8–9 μm with a straight to slightly oblique germ slit (Hladki and Romero 2010).

**Fig. 47:**
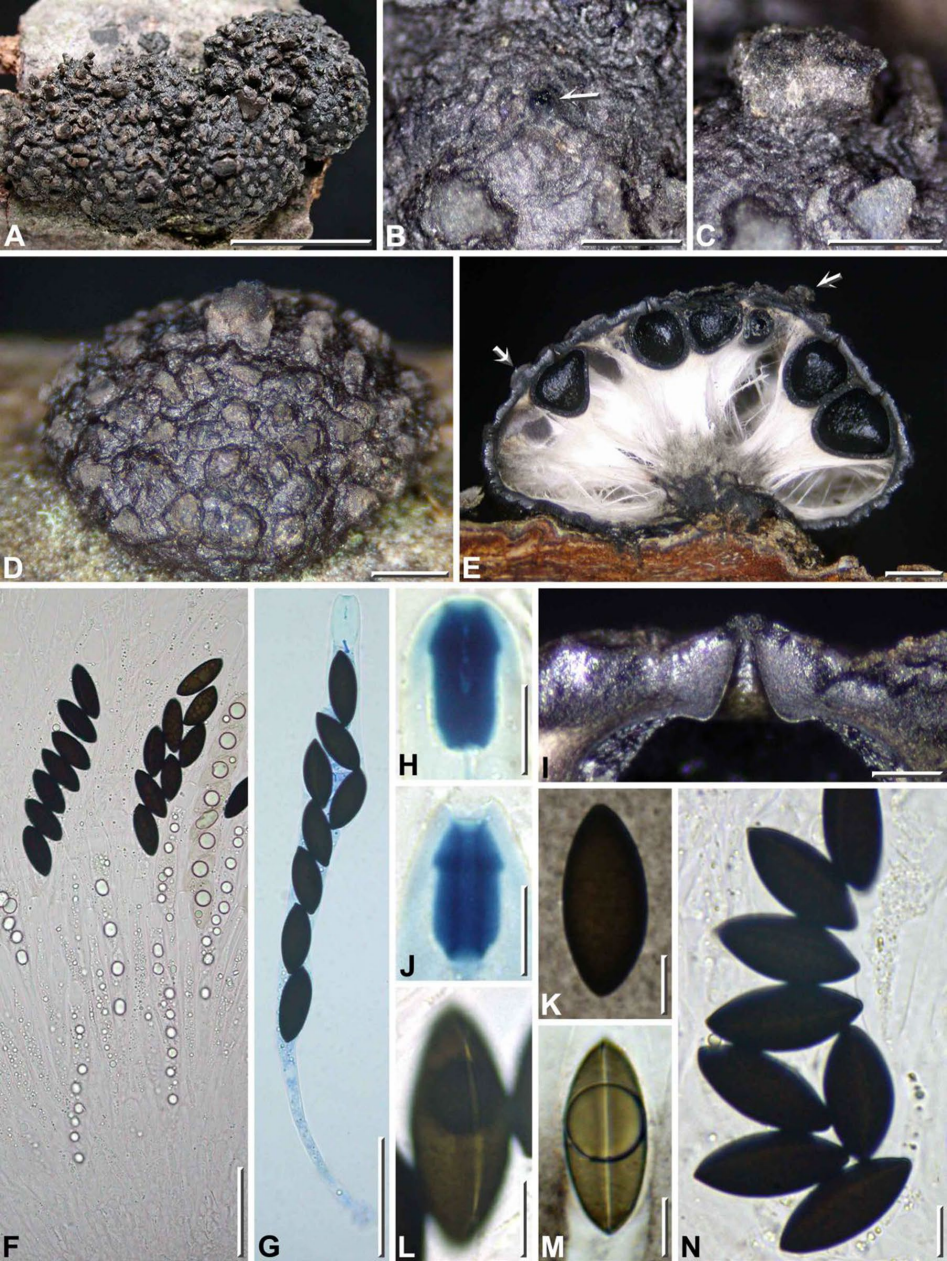
*Xylaria pachylepidota* (*GYJF21104* holotype). **A** Habit of four coalescent stromata. **B** Stromatal surface showing a black papillate ostiole (arrow) and scattered warts. **C** Thick superficial wart in close-up. **D** Pulvinate stroma in side view. **E** Sessile stroma in vertical section showing perithecia immersed beneath a carbonaceous crust, a partly lacunose interior, a blackish connective and some superficial warts (arrows). **F** Mature asci in 1% SDS. **G** Mature short-stipitate ascus, in blue Pelikan ink. **H, J** Ascal apical apparati in Melzer’s reagent. **I** Ostiolar region in vertical section showing a thick carbonaceous crust. **K** Ascospore in India ink showing the absence of sheath or appendages. **L** Submature ascospore showing a germ slit, in 1% SDS. **M** Submature ascospore in the ascus showing a conspicuous germ slit, in India ink. **N** Mature ascospores in 1% SDS. Bars in **A** = 5 mm; **B, C** = 0.5 mm; **D, E** = 1 mm; **F, G** = 50 µm; **H, J–N** = 10 µm; **I** = 0.2 mm

***Xylaria pachylepidota*** J. Fourn., Y.-M. Ju, H.-M. Hsieh & Lechat, sp. nov. Figure 47

MycoBank MB863176

**Typification**: **FRENCH GUIANA.** Roura, Cacao, Camp Bonaventure, 4.330514 N, 52.338095 W, ca. 65 m, hygrophilic secondary rainforest, on dead corticated branch, 24 Mar 2021, *Fournier, J. GYJF21104* (holotype HAST 147421).

**Etymology**: From Greek *παχύς* (pachus)=thick and *λεπιδωτός* (lepidotus) = squamulose, for the unusually thick superficial scales on stroma.

**Diagnosis**: Differs from known penzigioid species of *Xylaria* with similar pulvinate and strongly carbonaceous stromata by the combination of thick, wart-like superficial scales and blackish-brown ellipsoid-equilateral ascospores with narrowly rounded to subacute ends, averaging 30.3 × 12.7 µm.

**Stromata** solitary or coalescent, superficial, pulvinate to depressed-spherical, 4–8 mm diam × 3–4 mm thick, sessile, attached to the bark by a central, narrow, black, carbonaceous connective; surface blackish, without exposed perithecial contours, densely covered by thick polygonal to cylindrical warts up to 0.5–0.6 mm thick, black, carbonaceous, coated with a gray to brownish gray powdery sterile tissue; hard-textured, subsurface a carbonaceous crust 170–210 µm thick; interior white, gray around the connective and in places beneath the outer crust, radially fibrous, partly lacunose. **Perithecia** subglobose, 1.0–1.15 mm diam. **Ostioles** inconspicuous, scattered, finely conic-papillate, black, ca. 120 µm diam at base, opening between the warts.

**Paraphyses** copious, hyphal, remotely septate, thin-walled, 7–10 µm wide at base, tapering to 1.5–2.5 µm wide above asci, embedded in mucilaginous material. **Asci** cylindrical, with (4–)8 slightly overlapping uniseriately arranged ascospores, occasionally biseriately arranged in upper half, the spore-bearing parts 160–225 × 21–35 µm, the stipes 70–145 µm long, fragile, with apical apparatus roughly tubular, attenuated at both ends, apically cupulate, with a sharp lateral rim at upper third, 12.3–14.7 × 6.4–7.3 µm (Me = 13.4 × 6.8 µm, *N* = 25), strongly bluing in Melzer’s reagent. **Ascospores** (23.7–)28.4–32.8(−34.8) × (11.0–)11.8–13.6(−14.6) µm, Q = (2.0–)2.2–2.7(−3.0), *N* = 60 (Me = 30.3 × 12.7 µm, Qe = 2.4), ellipsoid-equilateral, occasionally subcitriform, with narrowly rounded to subacute, occasionally slightly pinched ends, blackish brown, unicellular, with a straight, longitudinally oriented germ slit spore-length, fairly inconspicuous at full maturity; lacking appendages or mucilaginous sheath; epispore smooth.

**Asexual morph** on the natural substrate not observed.

**Known distribution**: French Guiana, known only from the holotype collection.

**Comments**: *Xylaria pachylepidota* is characterized by pulvinate, sessile, carbonaceous stromata with a surface cracked into thick wart-like scales, large blackish equilateral ascospores with acute ends and a straight, spore-length gem slit, and asci with a large apical apparatus. This combination of morphological characters does not correspond to any known species. The genus *Kretzschmaria* Fr. was considered because the superficial warts recall coremial remnants as encountered in that genus, but they apparently lack traces of conidiogenesis. The boundaries between *Xylaria* and *Kretzschmaria* are sometimes challenging, but the microscopical characteristics of *X. pachylepidota* do not correspond to any known species of *Kretzschmaria* either.

**Fig. 48:**
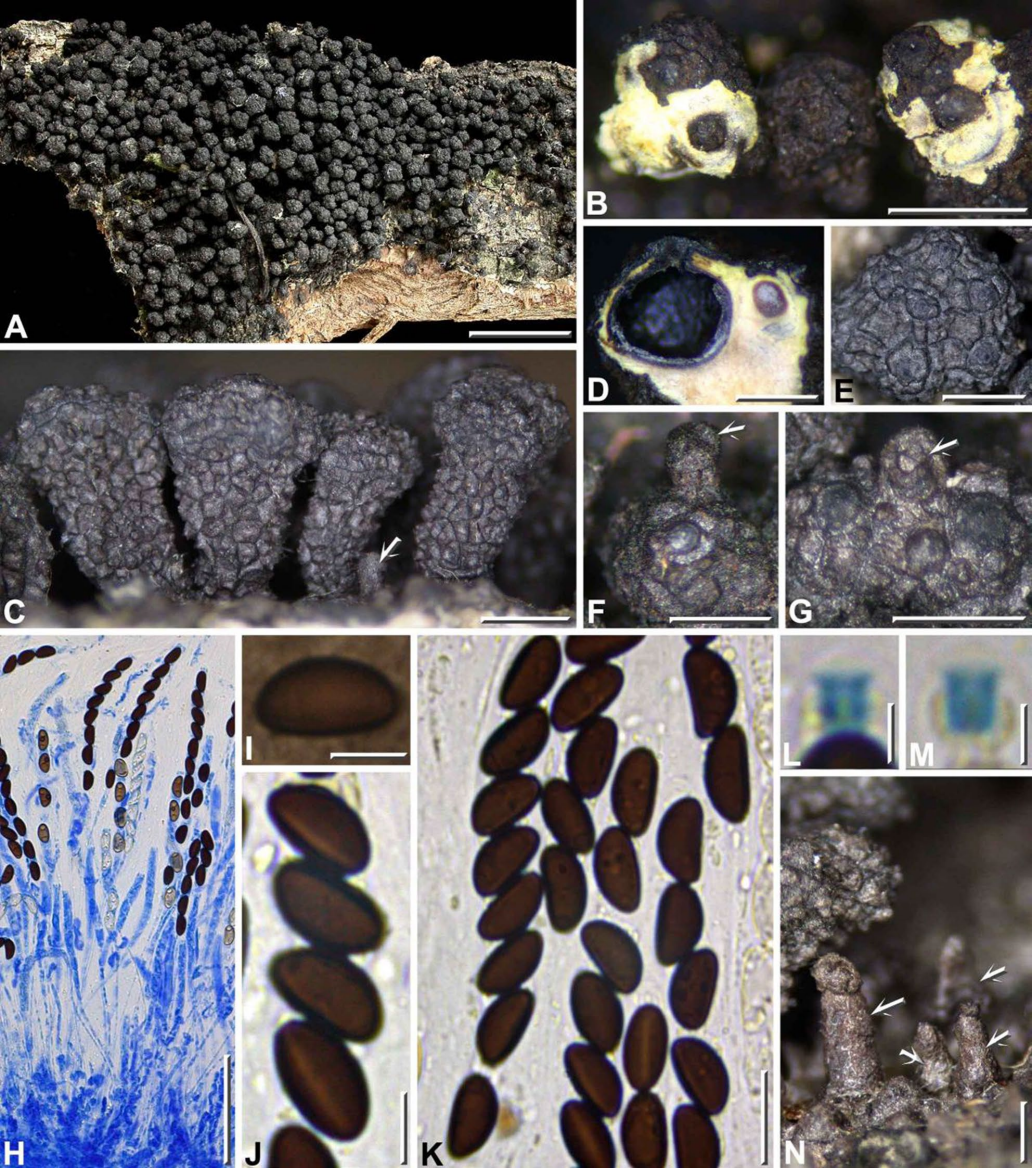
*Xylaria palifera* (**A, C, D, M, N ***GYJF19187* holotype; **B ***GYJF19174*; **E ***GYJF21035*; **F, G ***GYJF21105*; **H–L ***GYJF21070*). **A** Fragment of a dense colony on bark. **B** Stromatal heads with bruised surface showing a yellowish underlying tissue (zoom in). **C** Turbinate and flat-topped stromata in side view, associated with a peg-like structure (arrow). **D** Fertile head in vertical section showing a perithecium lined by a thin yellowish layer. **E** Fertile head in top view showing a cracked surface and black discoid ostioles. **F, G** Fertile heads in side view showing central peg-like structures and large discoid ostioles. **H** Long-stipitate asci, in blue Pelikan ink. **I** Ascospore in India ink showing a lack of sheath or appendages. **J, K** Ascospores in 1% SDS, some in ventral view showing a germ slit. **L, M** Ascal apical apparati, in Melzer’s reagent. **N** Peg-like structures (arrows) in side view arising from black stromatic swellings. Bars in **A** = 10 mm; **B, C, E** = 1 mm; **D, F, G, N** = 0.5 mm; **H** = 50 µm; **I, J** = 5 µm; **K** = 10 µm; **L, M** = 2 µm

***Xylaria palifera*** J. Fourn., Y.-M. Ju, H.-M. Hsieh & Lechat, sp. nov. Figure 48, Table 31

**Table 31:** Ascospore and ascal apical apparatus dimensions in five collections of *X. palifera* showing their variation range, compared with those reported from the holotype of *X. martinicensis* var. *microspora* from Martinique

Collection numbers	Ascospore measurements with extreme values in parentheses	Q = quotient l/w, *N* = number of measurements	Me = mean values of l/w, Qe = mean quotient l/w	Ascal apical apparatus h × w, *N* = 20
*GYJF19174*	(8.3–)8.6–9.6(−10.0) × (4.1–)4.3–4.9(−5.2) µm	Q = (1.6–)1.8–2.2(−2.4), *N* = 60	Me = 9.0 × 4.6 µm, Qe = 2.0	1.9–2.1 × 1.6–1.9 µm, Me = 2.0 × 1.7 µm
*GYJF19187* holotype	(7.7–)9.2–10.8(−11.5) × (4.4–)4.6–5.3(−5.7) µm	Q = (1.6–)1.7–2.3(−2.5), *N* = 60	Me = 9.9 × 5.0 µm, Qe = 2.0	2.0–2.3 × 1.7–1.9 µm, Me = 2.2 × 1.8 µm
*GYJF21035*	(8.7–)9.2–10.9(−12.3) × (4.6–)4.8–5.5(−5.8) µm	Q = (1.6–)1.7–2.1(−2.4), *N* = 60	Me = 10.0 × 5.1 µm, Qe = 2.0	2.0–2.3 × 1.7–1.9 µm, Me = 2.2 × 1.8 µm
*GYJF21070*	(7.8–)8.5–9.7(−10.1) × (4.1–)4.4–5.0(−5.2) µm	Q = (1.7–)1.8–2.1(−2.3), *N* = 60	Me = 9.1 × 4.7 µm, Qe = 1.9	1.9–2.2 × 1.4–1.8 µm, Me = 2.1 × 1.6 µm
*GYJF21105*	(7.2–)8.1–9.2 (−9.6) × (4.0–)4.4–5.1(−5.6) µm	Q = (1.5–)1.6–2.0(−2.1), *N* = 60	Me = 8.7 × 4.8 µm, Qe = 1.8	1.9–2.2 × 1.4–1.8 µm, Me = 2.0 × 1.6 µm
**Cumulated values**	**(7.2–)8.1–10.9(−12.3) × (4.0–)4.3–5.5(−5.8) µm**	**Q = (1.5–)1.6–2.3(−2.5), N = 300**	**Me = 9.3 × 4.8 µm, Qe = 1.9**	**1.9–2.3 × 1.4–1.9 µm, Me = 2.1 × 1.7 µm, N = 100**
*X. martinicensis* var. *microspora* (Fournier et al. 2020)	(8.2–)9.0–10.4(−10.8) × (3.9–)4.6–5.2(−5.5) μm	Q = (1.5–)1.8–2.2(−2.7), *N* = 60	Me = 9.6 × 4.9 μm, Qe = 2.0	1.9–2.4 × 1.8–2.1 µm, Me = 2.1 × 1.9 µm

MycoBank MB863177

**Typification**: **FRENCH GUIANA.** Maripasoula, Saül, trail head to Roche-Bateau, 3.620498 N, 53.199309 W, ca. 230 m, disturbed mesophilic rainforest, on dead corticated trunk, 22 Jun 2019, *Fournier, J. GYJF19187* (holotype HAST 147423).

**Etymology:** From Latin *palum* = pole, by extension a cylindrical structure and *fero* = to bear, referring to the central peg-like outgrowth present on or around young stromata.

**Diagnosis:** Differs from *X. martinicensis* var. *microspora* by smaller stromata and ostioles, less sharply defined stipes, and the frequent presence of central, sterile peg-like structures on the stromatal apex or between stromata.

**Stromata** kretzschmarioid-turbinate to penzigioid-subglobose, most often densely gregarious, rarely in small scattered clusters, simple, arising in linear rows or in bunches from black rhizomorph-like swellings erumpent from bark, subsessile to stipitate, 0.8–4.5 mm total height, the fertile head almost flat-topped to slightly convex, (0.6–)1.0–2.3 mm diam, 1–15-peritheciate, not sharply delimited from the stipe; developing and young stromata bearing a central, rarely lateral peg-like structure up to 200 µm high, 160–200 µm diam, short cylindric with a bluntly rounded apex, dull black, suggesting a coremial structure but lacking conidiogenous tissue, even at early stages; similar erect structures that can be interpreted as primordia arising from black stromatic swellings are often present around the base of stipes; surface blackish brown to dull black, glabrous, coarsely cracked into prominent polyhedral scales or warts, not to faintly nodulose, with perithecial contours unexposed; the stipe (0–)0.7–3.1 mm high × 0.4–1.5 diam in its widest part, surface brownish black, glabrous, coarsely cracked into thick polyhedral warts from the very base, lacking basal tomentum; subsurface black, leathery, brittle, ca. 40 µm thick; interior solid, whitish, soft-textured, typically with a faint citrine-yellow layer just beneath the black superficial crust, embedding the perithecia, best seen when the tissue is hydrated, due to minute yellowish granules accumulated within the hyphae, not changing color nor dissolving in 3% KOH. **Perithecia** subglobose to laterally flattened, 0.40–0.65 mm diam. **Ostioles** finely papillate, on a conspicuous, flat to convex, black raised-discoid base (170–)210–290 µm diam.

**Paraphyses** copious, hyphal, thin-walled, 6–9 µm wide at base, tapering to 2 µm wide above asci, sparsely guttulate, discreetly embedded in mucilaginous material. **Asci** cylindrical, long-stipitate, with (6–)8 overlapping uniseriately arranged ascospores, the spore-bearing parts 63–72 × 6.0–6.5(−8.5) µm, the stipes 80–90 µm long, with apical apparatus short-cylindrical to quadrate with a faint upper rim, 1.9–2.3 × 1.4–1.9 µm (Me = 2.1 × 1.7 µm, *N* = 100), bluing in Melzer’s reagent. **Ascospores** (7.2–)8.1–10.9(−12.3) × (4.0–)4.3–5.5(−5.8) µm, Q = (1.5–)1.6–2.3(−2.5), *N* = 300 (Me = 9.3 × 4.8 µm, Qe = 1.9), ellipsoid-inequilateral, with mostly broadly rounded ends, dark brown, unicellular, with a conspicuous, straight, longitudinally oriented germ slit almost spore-length on the less convex side when inequilateral; appendage or mucilaginous sheath absent; epispore smooth.

**Asexual morph** on the natural substrate not observed, supposed coremial structures being consistently sterile.

**Other specimens examined (paratypes): FRENCH GUIANA.** Roura, Cacao, Camp Bonaventure, 4.326815 N, 52.341431 W, 60–65 m, on dead corticated trunk, 22 Mar 2021, *Fournier, J. GYJF21070* (HAST 147422); Roura, Cacao, Camp Bonaventure, 4.330514 N, 52.338095 W, 60–65 m, on dead corticated branch, 24 Mar 2021, *Fournier, J. GYJF21105* (HAST 147424), depauperate; Maripasoula, Saül, trail head to Roche-Bateau, 3.620498 N, 53.199309 W, ca. 230 m, disturbed mesophilic rainforest, on dead corticated trunk, associated with *X. scruposa*, 22 Jun 2019, *Fournier, J. GYJF19174* (HAST 147425); Roura, Cacao, Camp Bonaventure, 4.323472 N, 52.338502 W, ca. 70 m, hygrophilic secondary rainforest, on dead corticated trunk, 21 Mar 2021, *Fournier, J. GYJF21035* (HAST 147426).

**Known distribution**: French Guiana, known from five collections from two different sites.

**Comments**: We document here five collections of a small kretzschmarioid taxon that we tentatively referred to *X*. *martinicensis* var. *microspora* based on similar stromatal features, including a coarsely corky-cracked surface extending to the stipe and a swollen fertile head with a thin yellowish layer underlying its surface. Microscopically, asci and ascospores are identical (Table 31), and the only obvious differences from the holotype from Martinique are the significantly smaller stromata and ostioles and less sharply defined stipes (Fournier et al. 2020). The presence of a central, sterile peg-like structure on the top of some fertile heads or between the stipes in our Guianese collections is likewise a character not observed in the holotype collection of *X*. *martinicensis* var. *microspora*. These differences are supported by our phylogenetic analyses, which place *X. palifera* close to but distinct from *X. martinicensis* and its var. *microspora*.

The main phenotypic difference from *X. heliscus* as documented in this paper is the presence of a faintly yellow tissue immediately beneath the surface. Although both species are kretzschmarioid, they are not immediately closely related phylogenetically, being separated by several xylarioid species.

**Fig. 49:**
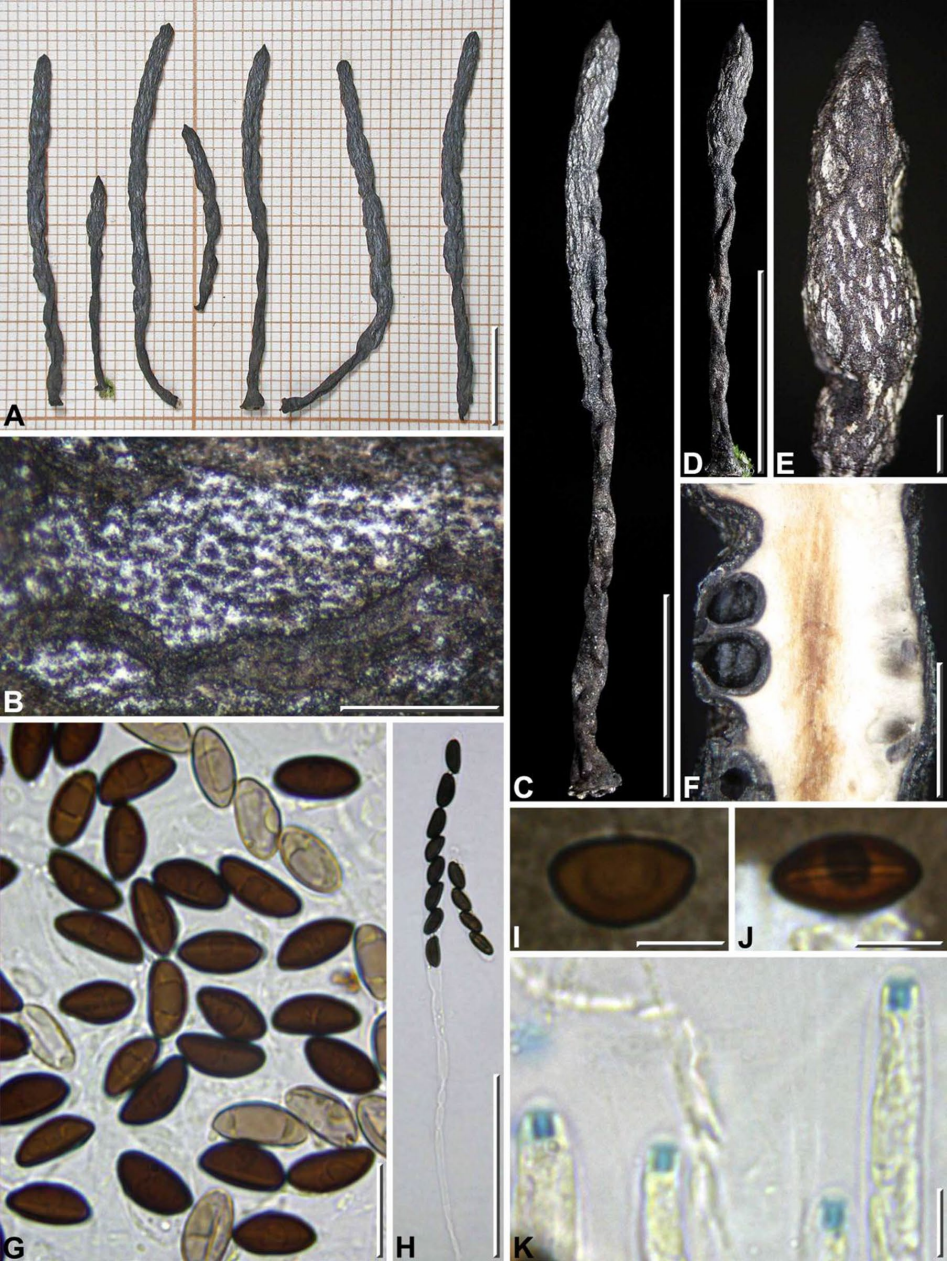
*Xylaria pallidocylindracea* (**A, C–E, G–K ***CLL8006*; **B, F ***GYJF12193*). **A, C, D** Habit of stromata. **B** Stromatal surface in close-up showing a white plaque densely incrusted with black granules. **E** Stromatal upper half showing a mucronate apex, white superficial stripes and a deep circumferential constriction. **F** Stroma in longitudinal section showing perithecia between two depressions and a white interior with an orange-brown core. **G** Mature and immature ascospores, some showing a germ slit, in 1% SDS. **H** Long-stipitate ascus, in 1% SDS. **I, J** Ascospores in India ink showing absence of sheath or appendage and a germ slit in **J, K** Ascal apical apparati in Melzer’s reagent. Bars in **A, C, D** = 10 mm; **B** = 0.5 mm; **E, F** = 1 mm; **G** = 10 µm; **H** = 50 µm; **I–K** = 5 µm

***Xylaria pallidocylindracea*** J. Fourn. & Lechat, Ascomycete.org 12 (3):137. 2020. Figure 49, Table 32

**Table 32:** Ascospore dimensions from the two known collections of *X. pallidocylindracea*

Collection numbers	Ascospore measurements with extreme values in parentheses	Q = quotient l/w, *N* = number of measurements	Me = mean values of l/w, Qe = mean quotient l/w
*CLL8006*	(7.7–)8.1–9.3(−10) × (3.5–)3.8–4.5(−4.7) µm	Q = (1.7–)1.9–2.3(−2.5) µm, *N* = 60	Me = 8.7 × 4.1 µm, Qe = 2.1
*GYJF12193*	(7.7–)8.1–9.0(−9.7) × (3.6–)3.9–4.5(−4.6) µm	Q = (1.7–)1.9–2.3(−2.5) µm, *N* = 60	Me = 8.5 × 4.1 µm, Qe = 2.1
**Cumulated values**	**(7.7–)8.1–9.3(−10) × (3.5–)3.8–4.5(−4.7) µm**	**Q = (1.7–)1.9–2.3(−2.5) µm, N = 120**	**Me = 8.6 × 4.1 µm, Qe = 2.1**

**Stromata** 22–40 mm in total height, the fertile parts 9–30 mm high × 1.8–2.0 mm wide, narrowly cylindrical with circumferential constrictions, straight to slightly curved, with apices sterile and mucronate; surface with unexposed perithecial contours, roughened by a pure white outer layer splitting into large longitudinal stripes densely incrusted with small black granules, exposing the dark gray underlying crust; interior solid, white, fibrous, with a yellowish to orange-brown core; outer crust 70–80 µm thick, slightly carbonaceous; the stipes ill-defined, 10–18 mm long × 1.3–1.5 mm, black, irregularly flattened, glabrous, smooth with sparse black granules at surface, the base slightly enlarged. **Perithecia** immersed, subglobose, 0.40–0.55 µm diam. **Ostioles** papillate, inconspicuous.

**Paraphyses** hyphal, hyaline, remotely septate, simple, 4–5 µm wide at base, tapering to 1.0–1.5 µm wide, embedded in mucilaginous material. **Asci** cylindrical, with (6–)8 uniseriately arranged, slightly overlapping ascospores, the spore-bearing parts 60–72 µm long × 5.0–5.8 µm wide, the stipes up to 90 µm long, fragile, with apical apparatus tubular with a faint upper rim, 2.2–2.6 × 1.3–1.8 µm (Me = 2.4 × 1.6 µm, *N* = 20), bluing in Melzer’s reagent. **Ascospores** (7.7–)8.1–9.3(−10) × (3.5–)3.8–4.5(−4.7) µm Q = (1.7–)1.9–2.3(−2.5), *N* = 120 (Me = 8.6 × 4.1 µm, Qe = 2.1), ellipsoid-inequilateral with broadly to narrowly rounded ends, dark brown, with a conspicuous straight germ slit slightly less than spore-length on the ventral side, lacking sheath or appendage; epispore smooth.

**Asexual morph** on the natural substrate not observed.

**Specimens examined**: **FRENCH GUIANA.** Kourou, Sentier de la Montagne des Singes, 5.07098 N, 52.696447 W, ca. 60 m, lowland rainforest, on dead wood, 27 Apr 2008, *Lechat, C. CLL8006* (HAST 147201); Sinnamary, Paracou, CIRAD field centre, Guyaflux plot, 5.249218 N, 52.915431 W, lowland rainforest, on dead wood, 25 Jun 2012, *Fournier, J. GYJF12193* (HAST 147603).

**Known distribution**: French Guiana and the French West Indies (Fournier et al. 2020).

**Comments**: Tropical *Xylaria* species with needle-shaped stromata that are apically mucronate and sterile are numerous and cannot be readily distinguished without careful examination of surface features, conspicuousness of ostioles, interior color and texture, and ascospore morphology.

The Guianese collections of *X. pallidocylindracea* are characterized by slightly carbonaceous stromata with circumferential constrictions and a persistent, pure white outer layer that splits into elongated plaques or stripes. This white outer layer is densely covered, even from early stages, with minute carbonaceous granular deposits. In contrast, the finely grooved, dark gray stromatal surface lacks black granular deposits and conspicuous ostioles.

The holotype of *X. pallidocylindracea* from Martinique (Fournier et al. 2020) lacks black granules on the white superficial stripes. Instead, granules occur on the underlying surface between the stripes, from which small conic-papillate ostioles emerge. The ITS sequences from specimens of both Martinique and French Guiana reconfirm their conspecificity.

*Xylaria sinuosirima* is another related species from French Guiana, sharing a similar stromatal surface but differing mainly by having a blackish interior and paler brown ascospores averaging 10.8 × 4.6 µm with a sigmoid germ slit (this paper). *Xylaria nigrosticta* also resembles *X. pallidocylindracea* in stromatal morphology but differs by having superficial granules aggregated into conspicuous clusters and slightly larger, more slender ascospores averaging 9.5 × 3.9 µm (this paper). Our phylogenetic analyses (Fig. 1) confirm the distinctiveness of these three species, *X*. *pallidocylindracea*, *X*. *sinuosirima*, and *X*. *nigrosticta*.

**Fig. 50:**
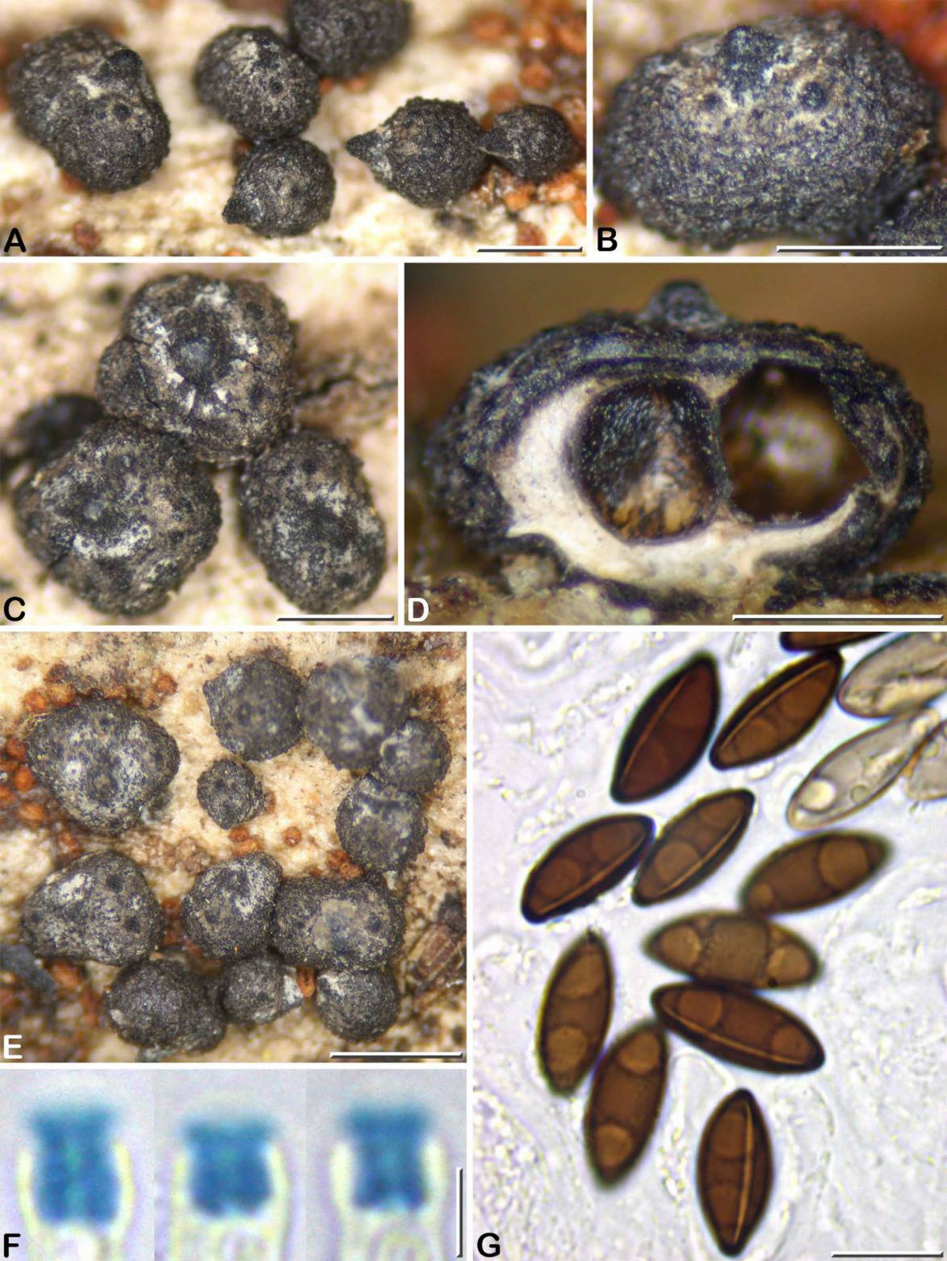
*Xylaria papillatoides* (*GYJF18140*). **A, C, E** Stromata clustered on host surface, in lateral or top view. **B** Stroma in close-up showing a central umbo, ostioles and surface roughened by black granules. **D** Stroma in vertical section. **F** Tubular to slightly urn-shaped ascal apical apparati in Melzer’s reagent. **G** Ascospores in 1% SDS, some in ventral view showing a germ slit. Bars in **A, B, D** = 0.5 mm; **C, E** = 1 mm; **F** = 3 µm; **G** = 10 µm

***Xylaria papillatoides*** J. Fourn. & Lechat, Ascomycete.org 10(4): 161. 2018. Figure 50, Table 33

**Table 33:** Ascospore and ascal apical apparatus dimensions in *GYJF18140*, compared with those of *X. papillatoides* reported in literature for collections from Guadeloupe and Martinique

Collection numbers	Ascospore measurements with extreme values in parentheses	Q = quotient l/w, *N* = number of measurements	Me = mean values of l/w, Qe = mean quotient l/w	Ascal apical apparatus h × w
*GYJF18140*	(14.2–)14.4–16.2(−16.8) × (5.4–)6.1–7.0(−7.2) µm	Q = (2.1–)2.2–2.6(−2.7), *N* = 60	Me = 15.3 × 6.5 µm, Qe = 2.4	2.8–3.7 × 2.1–2.7 µm, Me = 3.2 × 2.3 µm
Original description	(13.1–)13.6–17.2(−17.9) × (4.8–)5.3–6.8(−7.4) µm	Q = (2.0–)2.2–3.0(−3.2), *N* = 240	Me = 14.6 × 5.8 µm, Qe = 2.5	3.0–4.1 × 2.3–3.3 µm, Me = 3.6 × 2.7 µm

**Stromata** gregarious to loosely scattered, subglobose 0.4–0.5 mm diam when uniperitheciate to pulvinate 0.75–1.2 mm wide × 0.5–0.7 mm high when 2–5-peritheciate, with a pointed to blunt, sterile, usually persistent central umbo, subsessile, on a narrow central connective; surface blackish gray, eventually black, without or with barely exposed perithecial contours, roughened by shallow cracks and encrusted with small black, carbonaceous, superficial granules; with a white, poorly developed outer layer vaguely spread around the central umbo; subsurface crust slightly carbonaceous, brittle, 60–80 µm thick, black; interior white, solid, fibrous. **Perithecia** immersed, subglobose 0.35–0.42 mm diam. **Ostioles** bluntly papillate, mostly faintly prominent, black, 50–80 µm diam.

**Paraphyses** and **Asci** as in Fournier et al. (2018c); ascal apical ring tubular to slightly urn-shaped with a marked upper rim, 2.8–3.7 × 2.1–2.7 µm (Me = 3.2 × 2.3 µm, *N* = 25), bluing in Melzer’s reagent. **Ascospores** (14.2–)14.4–16.2(−16.8) × (5.4–)6.1–7.0(−7.2) µm, Q = (2.1–)2.2–2.6(−2.7), *N* = 60 (Me = 15.3 × 6.5 µm, Qe = 2.4), ellipsoid-inequilateral with narrowly rounded ends, unicellular, brown to dark brown, with a conspicuous, straight, longitudinal to rarely slightly oblique germ slit spore-length on the flattened side, lacking secondary appendages or mucilaginous sheath; epispore smooth.

**Asexual morph** on the natural substrate not observed.

**Specimen examined: FRENCH GUIANA.** Maripasoula, Saül, Trail head to Sentier des Gros Arbres, 3.620139 N, 53.208103 W, ca. 210 m, disturbed secondary rainforest, on dead corticated liana 15 mm diam, associated with an unidentified effete Nectriaceae, 25 Aug 2018, *Fournier, J. GYJF18140* (HAST 147427).

**Known distribution**: French Guiana, Guadeloupe, Martinique.

**Comments**: *Xylaria papillatoides* was originally described from four collections from Guadeloupe and Martinique, featuring small penzigioid stromata bearing a central umbo coated with a persistent white outer layer typically splitting around the base of the umbo, and ellipsoid-inequilateral ascospores 13.6–17.2 × 5.3–6.8 µm with narrowly rounded ends and a conspicuous straight germ slit (Fournier et al. 2018c). The collection documented here fits this definition well in most respects, deviating only by smaller stromata, a less conspicuous white outer layer, and slightly larger ascospores. This collection is abundant but unfortunately mostly immature, which may account for the discrepancies observed.

**Fig. 51:**
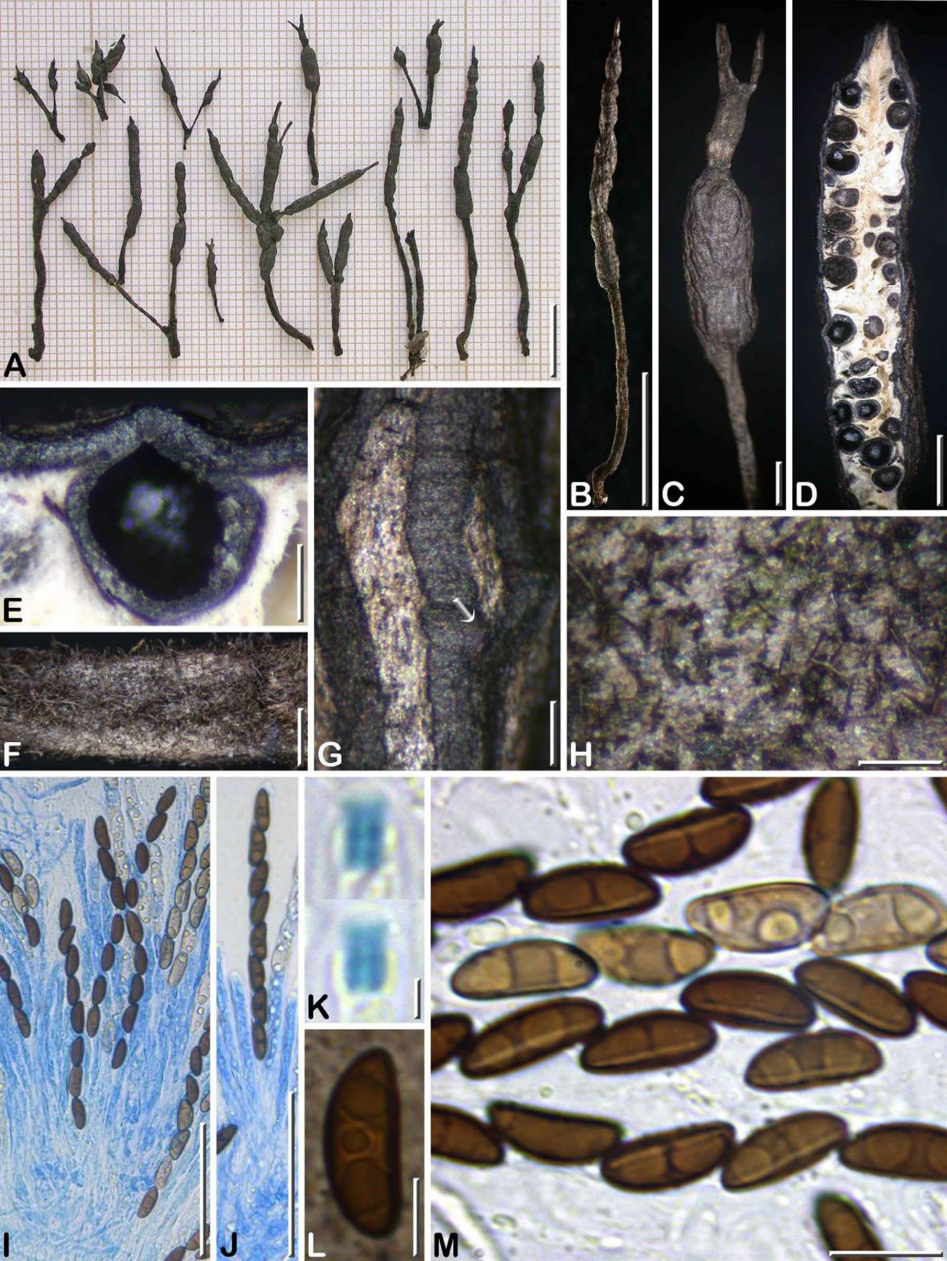
*Xylaria partita* (*GYJF21144*). **A** Diversely branched and constricted stromata. **B** Needle-shaped stroma. **C** Short-cylindrical stoma with a forked apical extension. **D** Stroma in longitudinal section. **E** Perithecium beneath a carbonaceous crust, in vertical section. **F** Hairy surface of a stipe. **G** Stromatal surface in close-up showing minutely dotted gray stripes and an obscurely papillate ostiole (arrow). **H** Short black hairs at the base of a fertile head. **I, J** Asci in blue Pelikan ink. **K** Ascal apical apparati in Melzer’s reagent. **L** Ascospore in India ink showing a lack of mucilaginous sheath or appendage. **M** Ascospores in 1% SDS, most of them showing a germ slit spore-length. Bars in **A, B** = 10 mm; **C, D** = 1 mm; **E, G, H** = 0.2 mm; **F** = 0.5 mm; **I, J** = 50 µm; **K** = 2 µm; **L** = 5 µm; **M** = 10 µm

***Xylaria partita*** C.G. Lloyd Mycol. Writings 5: 675. 1917. Figure 51, Table 34

**Table 34:** Ascospore, ascal apical apparatus and germ slit dimensions in six collections of *X. arbuscula* from French Guiana and Martinique featuring hairy stipes, compared with those reported from Martinique by Fournier et al. (2020) and compared with those recorded from the holotype of *X. partita*

Collection numbers	Ascospore measurements with extreme values in parentheses	Q = quotient l/w, *N* = number of measurements	Me = mean values of l/w, Qe = mean quotient l/w	Ascal apical apparatus h × w, *N* = 20	Germ slit length, *N* = 10
*GYJF21144* FG *X. partita*	(9.6–)10.7–12.2(−12.6) × (4.1–)4.4–5.0(−5.4) µm	Q = (2.0–)2.2–2.6(−2.7), *N* = 60	Me = 11.3 × 4.8 µm, Qe = 2.4	2.8–3.5 × 1.6–2.1 µm, Me = 3.1 × 1.8 µm	(6–)8–10 µm
*GYJF21228* FG	(10.2–)11.3–13.2(−13.9) × (3.9–)4.6–5.2(−5.6) µm	Q = (1.9–)2.3–2.7(−2.8), *N* = 60	Me = 12.2 × 4.9 µm, Qe = 2.5	2.5–2.9 × 1.7–2.0 µm, Me = 2.7 × 1.8 µm	7–9 µm
*GYJF21388* FG	(10.2–)10.6–12.2(−13.0) × (4.0–)4.4–5.0(−5.4) µm	Q = (2.2–)2.3–2.6(−3.1), *N* = 60	Me = 11.4 × 4.7 µm, Qe = 2.4	2.7–3.0 × 1.7–2 µm, Me = 2.8 × 1.8 µm	8–9 µm
*CLL9095* FG	(11.4–)11.8–13.3(−13.8) × (4.6–)4.9–5.8(−6.1) µm	Q = (2.0–)2.1–2.6(−2.7), *N* = 60	Me = 12.4 × 5.3 µm, Qe = 2.4	2.5–2.9 × 1.9–2.0 µm, Me = 2.7 × 2.0 µm	8–9.5 µm
*CLL6156* Martinique	(11.1–)11.5–13.5(−15) × (4.3–)4.6–5.2(−5.5) µm	*N* = 60	Me = 12.6 × 4.9 µm, Qe = 2.6	2.8 × 1.8 µm	(6–)8–10 µm
*CLL8295* Martinique	(10.6–)10.9–12.3(−12.7) × (4.0–)4.5–5.3(−5.7) µm	*N* = 60	Me = 11.6 × 5.0 µm, Qe = 2.4		6–7 µm
*X. partita* holotype (Ju et al. 2016)	10–12 × 4.5–5.5 µm		Me = 11 × 5 µm, Qe = 2.2		
*X. arbuscula* s. l. (Fournier et al. 2020)	(10.3–)10.9–15.4(−17) × (4–)4.3–5.8(−6.4) µm	*N* = 1020	Me = 12.6 × 5 µm, Qe = 2.5	2.7–3.3 × 1.8–2.0 µm, Me = 3.0 × 1.9 µm, *N* = 240	6–9(−12) µm

**Stromata** 11–41 mm in total height, scattered, upright, simple to most often branched or connate at base by two, the fertile parts 3–22 mm high × 1.0–1.9 mm wide, narrowly cylindrical with scattered deep circumferential constrictions, straight, with short to long sterile apical extensions, simple to forked; surface blackish, finely transversally grooved, with unexposed perithecial contours, hairy in places, roughened by a thick, grayish, becoming blackish horny outer layer splitting into narrow longitudinal stripes irregularly flecked by blackish dots likely remnants of hairs; underlying crust 70–80 µm thick, black, slightly carbonaceous; interior loosely fibrous, white, with a light orange core; the stipes well-defined, 4–20 mm long × 0.5–1.2 mm wide, blackish gray, strap-like, densely hairy, shaggy at base. **Perithecia** subglobose, rarely depressed-spherical, 0.40–0.60 mm diam, fully immersed. **Ostioles** bluntly papillate to barely differentiated, inconspicuous.

**Paraphyses** hyphal, thin-walled, 2.5–3.0 µm wide at base, tapering to 1–1.5 µm wide above asci, sparsely and minutely guttulate, embedded in mucilaginous material. **Asci** cylindrical, with (6–)8 slightly overlapping uniseriately arranged ascospores, the spore-bearing parts 83–92 × 5.5–7.0 µm, the stipes 65–85 µm long, with apical apparatus 2.8–3.5 × 1.6–2.1 µm (Me = 3.1 × 1.8 µm, *N* = 25), tubular to slightly urn-shaped with an obtuse upper rim, bluing in Melzer’s reagent. **Ascospores** (9.6–)10.7–12.2(−12.6) × (4.1–)4.4–5.0(−5.4) µm, Q = (2.0–)2.2–2.6(−2.7), *N* = 60 (Me = 11.3 × 4.8 µm, Qe = 2.4), ellipsoid-inequilateral with broadly rounded ends, medium brown to dark brown, unicellular, with a conspicuous, straight, longitudinally oriented germ slit almost spore-length on the ventral side, without sheath or appendages; epispore smooth.

**Asexual morph** on the natural substrate not observed.

**Specimens examined: FRENCH GUIANA.** Maripasoula, Saül, trail head to Sentier des Gros Arbres, 3.620839 N, 53.208536 W, ca. 210 m, disturbed secondary rainforest, on dead corticated branchlet, 26 Mar 2021, *Lechat, C. GYJF21144* (HAST 147428); Maripasoula, Saül, trail up to Hmong village, 3.626053 N, 53.208075 W, 240–250 m, disturbed secondary rainforest, on bark, 2 Apr 2021, *Fournier, J. GYJF21388* (HAST 147208); Sinnamary, Paracou, CIRAD field centre, plot 16, 5.249218 N, 52.915431 W, lowland rainforest, on dead corticated branch, 27 Apr 2009, *Lechat, C. CLL9095* (HAST 147209); Maripasoula, Saül, trail head to Sentier des Gros Arbres, 3.6201 N, 53.207989 W, disturbed secondary rainforest, on dead corticated branchlet, 28 Mar 2021, *Fournier, J. GYJF21228* (HAST 147210).

**Known distribution**: Neotropical. French Guiana, Puerto Rico.

**Comments**: Among collections referable to *X. arbuscula* in a broad sense, collection *GYJF21144* appeared somewhat atypical in exhibiting hair remnants on the gray superficial stripes of the stroma and hairs on the stipe, along with slightly smaller ascospores bearing an almost spore-length germ slit. Our phylogenetic analyses indicate that this collection is distinct from typical *X. arbuscula* and, based on ascospore characteristics, corresponds well to the concept of *X. partita*, as recently redefined in a revision of Lloyd’s type specimens by Ju et al. (2016). The presence of hairs has not been reported for *X*. *partita*, but Ju and Hsieh (2023) emphasized this feature in distinguishing *X. maitlandii* (Dennis) D. Hawksworth from typical *X. arbuscula.* Ju and Hsieh (2023) examined the type material of *X. maitlandii* from Uganda (Dennis 1958), confirming it as a hairy counterpart of *X. arbuscula*, and recognized it as distinct on account of dense hairs covering the striped outer peeling layer, regarding it as unique within the *X. arbuscula* group.

Collection *GYJF21144* differs from *X. maitlandii* by having short hair remnants restricted to the stripes, producing a grayish, uneven stromatal surface. It also possesses a thicker carbonaceous crust (70–80 µm vs 40 µm) and paler, narrower ascospores (11.3 × 4.8 µm, Qe = 2.4) than those of *X*. *maitlandii* (10.9 × 6.4 µm, Qe = 1.7).

These observations prompted us to reexamine herbarium specimens identified as *X. arbuscula* from French Guiana and Martinique that exhibited varying degrees of hairiness, at least on the stipes. Our results, summarized in Table 34, reveal no consistent correlation between hairiness, ascospore dimensions, and germ slit length. Therefore, the presence of hairs appears to hold limited taxonomic value within this group closely related to *X. partita*.

**Fig. 52:**
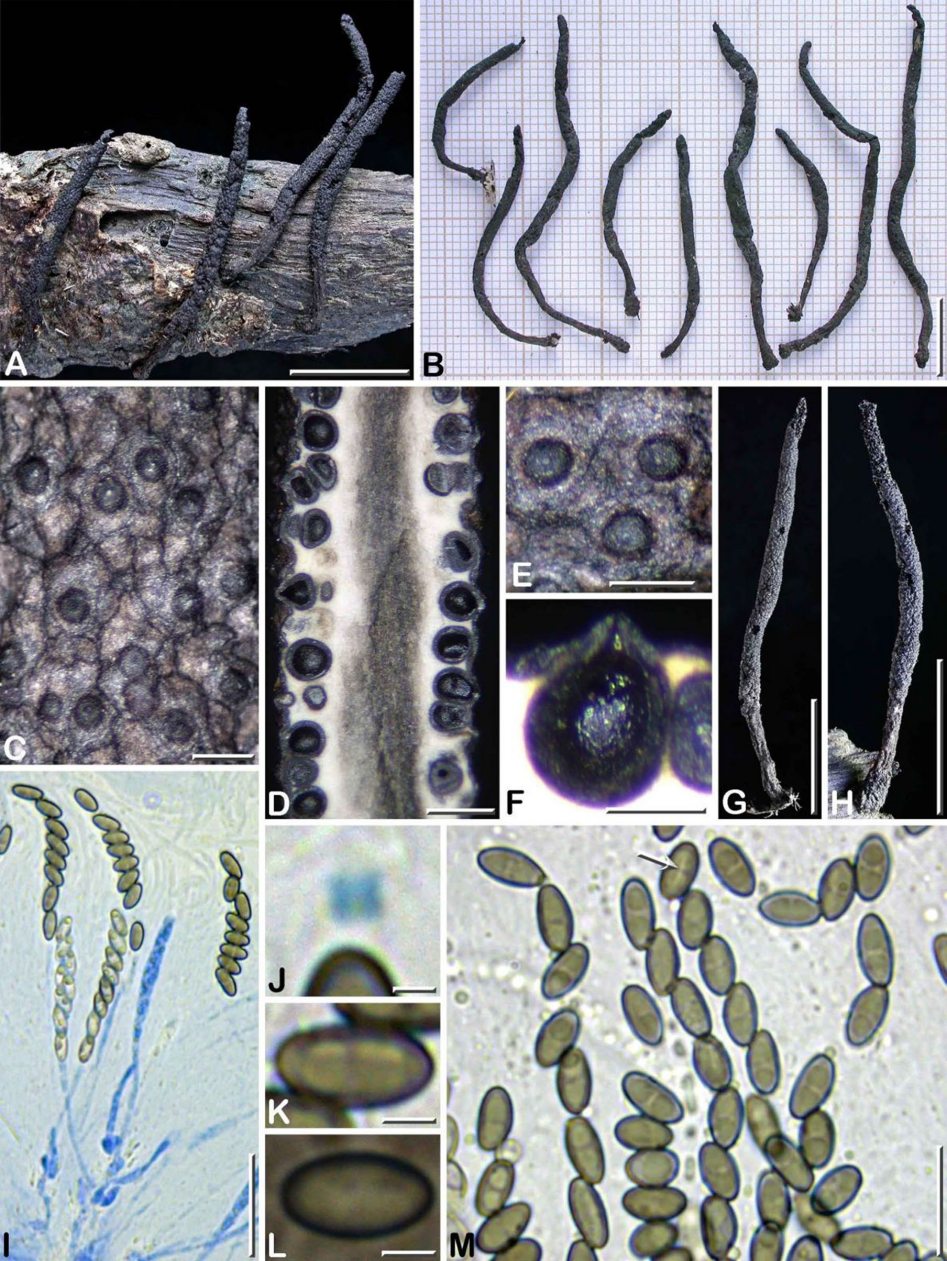
*Xylaria parvispora* (**A–C, E, G–M ***GYJF21168* holotype; **D, F ***GYJF21304* paratype). **A, B, G, H** Variously curved stromata. **C, E** Stromatal surface in close-up showing superficial cracking and ostioles. **D** Stroma in longitudinal section showing perithecia immersed beneath a thin black crust and interior with a conspicuous black core. **F** Perithecium in vertical section. **I** Asci in blue Pelikan ink. **J** Ascal apical apparatus in Melzer’s reagent. **K** Ascospore showing a short germ slit, in 1% SDS. **L** Ascospore in India ink showing absence of sheath or appendage. **M** Ascospores in 1% SDS, one showing a germ slit (arrow). Bars in **A, B, G, H** = 10 mm; **C, E, F** = 0.2 mm; **D** = 0.5 mm; **I** = 20 µm; **J** = 1 µm; **K, L** = 2 µm; **M** = 10 µm

***Xylaria parvispora*** J. Fourn., Y.-M. Ju, H.-M. Hsieh & Lechat, sp. nov. Figure 52

MycoBank MB863178

**Typification**: **FRENCH GUIANA.** Maripasoula, Saül, trail head to Sentier des Gros Arbres, 3.6201 N, 53.207989 W, ca. 210 m, disturbed mesophilic rainforest, on dead decorticated blackened branch, 27 Mar 2021, *Fournier, J. GYJF21168* (holotype HAST 147430).

**Etymology**: From Latin *parvus =* small, for the small ascospores.

**Diagnosis**: Differs from *X. feejeensis*, outwardly the most resembling known species, by smaller and paler ascospores averaging 5.6 × 2.9 µm vs 9.2 × 4.7 µm, with broadly rounded ends and a short germ slit.

**Stromata** upright, scattered or in small clusters, simple, (12–)23–45 mm in total height, 1.3–2.6 mm diam, cylindrical, terete, straight to curved, apically narrowly rounded and fertile but fragile and frequently broken, with perithecial contours unexposed; surface blackish brown, glabrous, densely reticulately cracked into minute polygonal scales and roughened by ostioles; subsurface a black leathery crust 30–40 µm thick; interior solid, soft-textured, white with a wide blackish core; the stipes ill-defined, 3.6–12 mm high × 0.8–1.3 mm diam, straight to slightly curved, terete to flattened, blackish, irregularly cracked and glabrous in upper half, slightly hairy or tomentose in lower part, slightly swollen at base. **Perithecia** subglobose, 0.28–0.34 mm diam. **Ostioles** minutely papillate, raised discoid to obtusely rounded, black, 100–130 µm diam, ca. 80 µm high, prominent above the cracked surface.

**Paraphyses** sparse, hyphal, remotely septate, thin-walled, 3–4 µm wide at base, tapering to 0.8–1.5 µm wide above asci, embedded in mucilaginous material. **Asci** cylindrical, with 8 slightly overlapping uniseriately arranged ascospores, the spore-bearing parts 34–41 × 3.7–5.0 µm, the stipes 40–50 µm long, with apical apparatus quadrate in optical view, apically flattened with a faint lateral rim, 0.8–1.1 × 1.0–1.2 µm (Me = 1.0 × 1.1 µm, *N* = 20), bluing in Melzer’s reagent. **Ascospores** (4.8–)5.2–6.0(−6.5) × (2.6–)2.8–3.1(−3.3) µm, Q = (1.6–)1.8–2.1(−2.4), *N* = 120 (Me = 5.6 × 2.9 µm, Qe = 1.9), ellipsoid-equilateral with broadly rounded ends, light olivaceous brown in the fresh state, unicellular, with a fairly conspicuous, straight, longitudinally oriented germ slit much less than spore-length; lacking appendage or mucilaginous sheath; epispore smooth.

**Asexual morph** on the natural substrate not observed.

**Other specimen examined (paratype): FRENCH GUIANA.** Maripasoula, Saül, trail head to Sentier Roche-Bateau, Crique-Cochon, 3.625994 N, 53.1955 W, 210–220 m, mesophilic disturbed rainforest, on dead corticated branch, 31 Mar 2021, *Fournier, J. GYJF21304* (HAST 147429).

**Known distribution**: French Guiana, known only from two collections.

**Comments**: The stromata of *X. parvispora* share with those of *X. feejeensis* the same narrowly cylindrical outline and a minutely cracked surface with prominent raised-discoid ostioles. However, they differ in being narrower, 1.3–2.6 mm vs 2–5(−14) mm with slightly smaller ostioles, 100–130 µm diam vs 120–170(−200) µm diam, and in having an interior with a black core. *Xylaria parvispora* is unambiguously distinguished from *X. feejeensis* by significantly smaller and paler brown ascospores averaging 5.6 × 2.9 µm with broadly rounded ends and a short germ slit, whereas those of the latter are dark brown averaging 9.2 × 4.7 µm with narrowly rounded ends and a long germ slit almost spore-length (Ju and Hsieh 2019; Fournier et al. 2020). It is remarkable that *X. feejeensis* was not collected in FG during this study, although it has a pantropical distribution and is common in Martinique and Guadeloupe (Fournier et al. 2020).

*Xylaria botuliformis* Rehm also has small ascospores 6.6–7.8 × 3.4–4.0 μm (Ju and Hsieh 2019), but they are slightly larger than those of *X*. *parvispora*, which measure 5.2–6.0 × 2.8–3.1 µm. The stromata of *X. botuliformis*, like those of *X*. *parvispora*, are also similar to *X*. *feejeensis*. Despite that these three species share similar morphologies, they do not show close relatedness in our phylogenetic analyses.

The new species *X. crustulata* (this paper) is similar to *X. parvispora* in stromatal shape and surface characteristics but differs by dark brown to blackish brown ellipsoid-inequilateral ascospores averaging 8.6 × 4.5 µm with a long germ slit.

*Xylaria aemulans* Starbäck was described from Brazil as having small corky-cracked stromata and small ascospores 6–7.5 × 4–5 µm (Starbäck 1901). Dennis (1958) commented on *X. aemulans*, stating that it is a small-spored variant of *X. feejeensis*. Its holotype [**BRAZIL.** Matto Grosso, Cuyabá, on wood, 10 Apr 1894, *Malme, G. A.* (S F5633)], however, has larger ascospores, 8–10 × 4.5–5 µm; *X*. *aemulans* is therefore considered a synonym of *X*. *feejeensis* and distinct from *X. parvispora*.

***Xylaria penicilliopsis*** (Henn.) Y.-M. Ju, in Réblová et al., IMA Fungus 7: 141. 2016.

≡ *Moelleroclavus penicilliopsis* Henn., Hedwigia 41: 15. 1902.

= *Xylaria moelleroclavus* J. D. Rogers, Y.-M. Ju & Hemmes, Mycol. Res. 101: 345. 1997.

The cited collection from French Guiana closely matches those reported and illustrated as *X. moelleroclavus* from the French West Indies (Fournier et al. 2019).

**Specimens examined: FRENCH GUIANA.** Sinnamary, Paracou, CIRAD field centre, Guyaflux plot, 5.249218 N, 52.915431 W, lowland rainforest, on dead wood, 27 Feb 2007, *Lechat, C. CLL7080A* (HAST 147616); Macouria, Risquetout Forest, trail to Saut-Léodate, 4.899372 N, 52.555061 W, ca. 55 m, lowland rainforest, on dead corticated trunk, 6 May 2008, *Lechat, C. CLL8109* (HAST 147617); Régina, Nouragues natural reserve, Inselberg camp, 4.311240 N, 52.132789 W, ca. 300 m, hygrophilic rainforest, on blackened wood, 18 Jun. 2012, *Fournier, J. GYJF 12060* (JF).

**Fig. 53:**
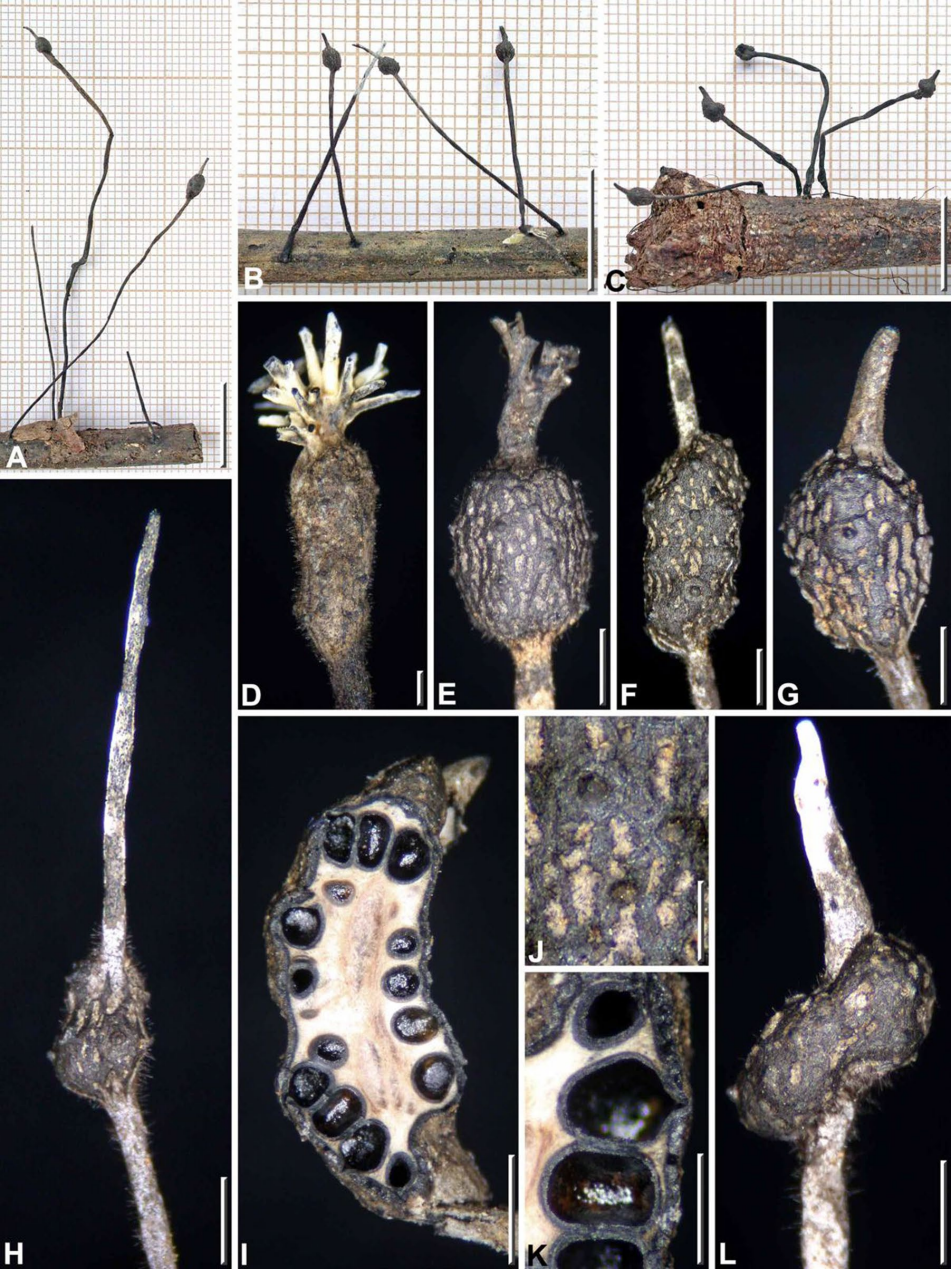
*Xylaria petiolorum* (*GYJF19201* holotype). **A–C** Variously shaped and stipitate stromata. **D** Immature stroma showing a hairy brownish surface and a bundle of remnants of conidiomata. **E–G** Fertile heads with branched or spike-like apical processes. **H, L** Immature stromatal heads with spike-like conidiomata coated with white pruinose asexual morph. **I** Stromatal head in vertical section. **J** Blackish surface with brownish gray scales and prominent ostioles. **K** Stromatal crust in vertical section and underlying perithecia. Bars in **A–C** = 10 mm; **D–I, L** = 1 mm; **J, K** = 0.5 mm

**Fig. 54:**
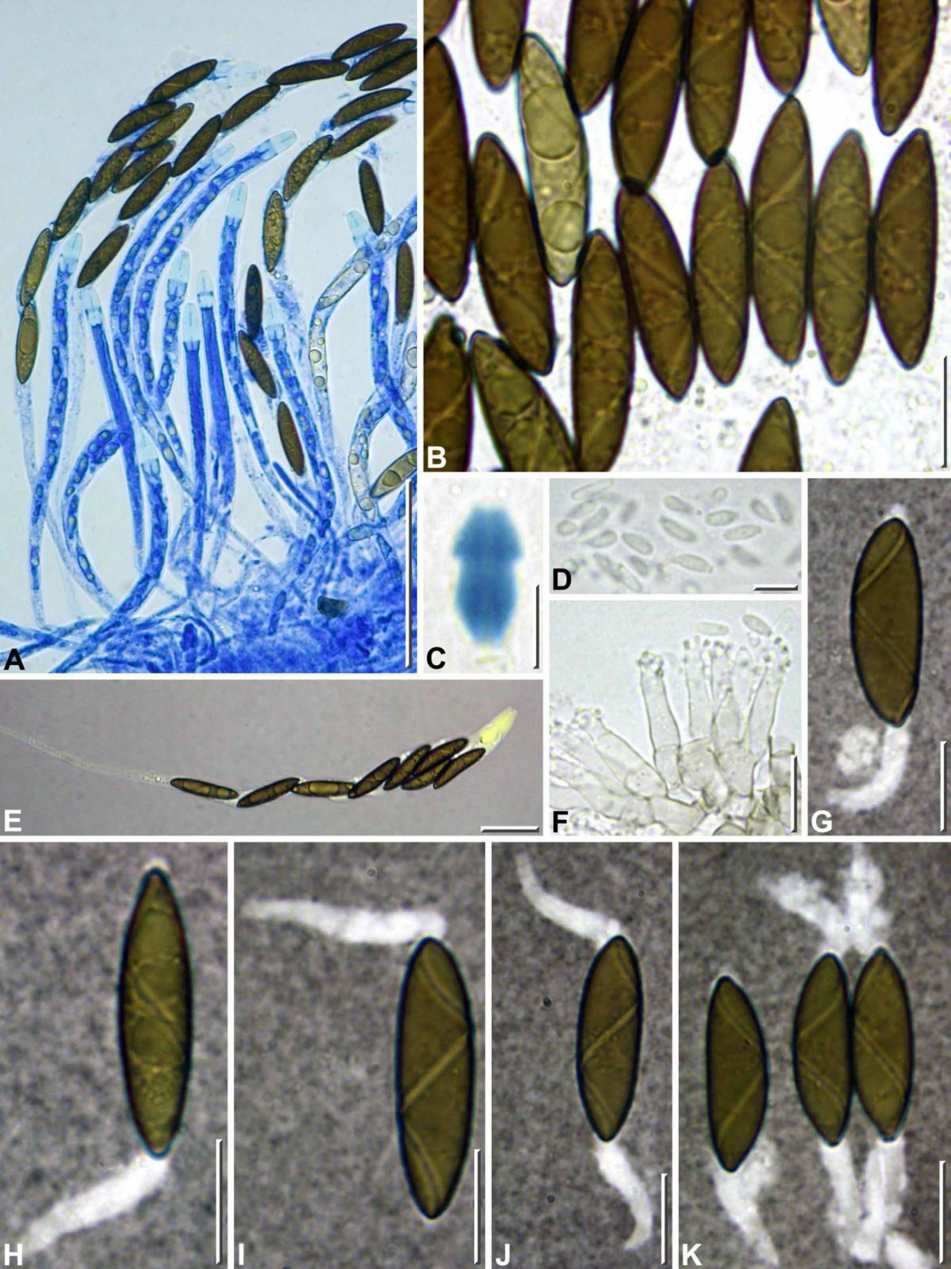
*Xylaria petiolorum* (*GYJF19201* holotype). **A** Bunch of immature and mature asci, in blue Pelikan ink. **B** Ascospores in 1% SDS, most of them showing a germ slit. **C** Ascal apical apparatus in Melzer’s reagent. **D** Conidia in 1% SD. **E** Ascus with broken stipe and multiseriate ascospores in upper part, in black Pelikan ink. **F** Conidiogenous cells, in 1% SDS. **G–K** Ascospores in India ink showing variously shaped elongated uni- or bipolar appendages and mucilaginous remnants where appendages detached. Bars in **A** = 50 µm; **B, F–K** = 10 µm; **C, D** = 5 µm; **E** = 20 µm

***Xylaria petiolorum*** J. Fourn., Y.-M. Ju, H.-M. Hsieh & Lechat, sp. nov. Figures 53, 54

MycoBank MB863179

**Typification**: **FRENCH GUIANA.** Maripasoula, Saül, trail head toward Roche-Bateau, 3.620498 N, 53.199309 W, disturbed secondary rainforest, on dead unidentified leaf petioles 3–6 mm diam in the leaf litter, 22 Jun 2019, *Fournier, J. GYJF19201* (holotype HAST 147433).

**Etymology:** From Latin *petiolus* = small stipe, petiole, referring to its occurrence on leaf petioles.

**Diagnosis**: Differs from known species related to *X. comosa* by its occurrence on leaf petioles and ascospores 20.4–23.4 × 5.2–6.5 µm, with a spiraling germ slit and bearing conspicuous secondary appendages.

**Stromata** scattered or in small groups on dead unidentified leaf petioles, upright, separate, (9–)15–55 mm in total height, long-stipitate, with distinctly differentiated fertile heads; the fertile heads ellipsoid, oblong or subglobose, 1.4–6.0 mm high × 1.2–2.4 mm diam, apically sterile with spike-shaped or ramified apical extensions, the stipes 6.0–52 mm high × 0.25–0.75 mm diam; surface brownish gray to gray, becoming blackish at maturity, with unexposed to slightly exposed perithecial contours; with brownish gray outer layer rather thick, fibrous, cracking into small elongated scales gradually worn off with age, frequently bearing scattered black stiff hairs; underlying crust hard-textured, black, slightly carbonaceous, ca. 80 µm thick; the stipes whitish gray to blackish, terete to slightly flattened, slightly carbonaceous but flexible, clothed in scattered black stiff hairs, slightly swollen at base; interior solid to loosely fibrous, white with orange-brown traces. **Perithecia** subglobose 0.40–0.65 mm diam, laterally flattened when in contact. **Ostioles** obtusely papillate, prominent, black, 120–150 µm diam.

**Paraphyses** hyphal, thin-walled, sparse, 8–11 µm wide at base, tapering to 1.5–2 µm wide above asci, embedded in mucilaginous material. **Asci** cylindrical to fusiform, with (4–6–)8 slightly overlapping uniseriately arranged ascospores, occasionally 2–3-seriate in upper part, the spore-bearing parts 72–138 × 11–16 µm, the stipes 57–123 µm long, with apical apparatus 6.4–8.5 × 3.7–4.1 µm (Me = 7.6 × 3.9 µm, *N* = 20), strongly constricted at upper third, apically trapezoid with sharp lateral rims, doliform in lower part, strongly bluing in Melzer’s reagent. **Ascospores** (19.6–)20.4–23.4(−24.5) × (4.7–)5.2–6.5(−6.9) µm, Q = (3.2–)3.4–4.1(−4.7), *N* = 60 (Me = 21.7 × 5.8 µm, Qe = 3.8), fusiform-equilateral to slightly inequilateral, with narrowly rounded ends, medium brown with olivaceous tone when fresh, unicellular, with a conspicuous spiraling germ slit coiled once all over the spore length with inflexion point on the dorsal side when inequilateral, with bipolar, persistent flattened cap-like appendages and bipolar conical secondary appendages 9–17 µm long × 3–3,5 µm wide at base, readily detached; epispore smooth.

**Asexual morph** on the natural substrate present as a white pruina on filiform tips of conidiogenous stromata or on apical extensions. Conidiogenous cells palisadic, cylindrical, straight to slightly geniculate, 11–17 × 2.7–4 µm, bearing terminal denticulate conidial secession scars. Conidia produced holoblastically in sympodial sequence, narrowly ellipsoid, hyaline, smooth, 3.5–4.5 × 1.3–1.8 µm.

**Other specimen examined (paratype)**: **FRENCH GUIANA.** Maripasoula, Saül, trail head toward Roche-Bateau, 3.620498 N, 53.199309 W, disturbed secondary rainforest, on dead unidentified leaf petioles 3–6 mm diam in the leaf litter, 22 Jun 2019, *Corriol, G. GYJF19191* (HAST 147432), immature.

**Known distribution**: French Guiana, known only from two collections.

**Comments**: This tiny *Xylaria* appears in many respects as a miniature of species in the *X. comosa* complex, with long-stipitate stromata bearing a well-defined stipe and a rounded, hard-textured fertile head with conspicuous apical conidial spikes and grayish scales on the surface. Its ascospores resemble those of *X. comosoides* in having a long spiraling germ slit and those of *X. comosa* in having long and fragile appendages. The apical apparatus is strikingly similar in shape to that of *X. comosa.* Its position in our phylogram within the clade containing *X. comosa* and allies was expectable. *Xylaria petiolorum* is easily separated from known species occurring on dead petioles (Ju and Hsieh 2023). Material of this species is unfortunately mostly immature.

**Fig. 55:**
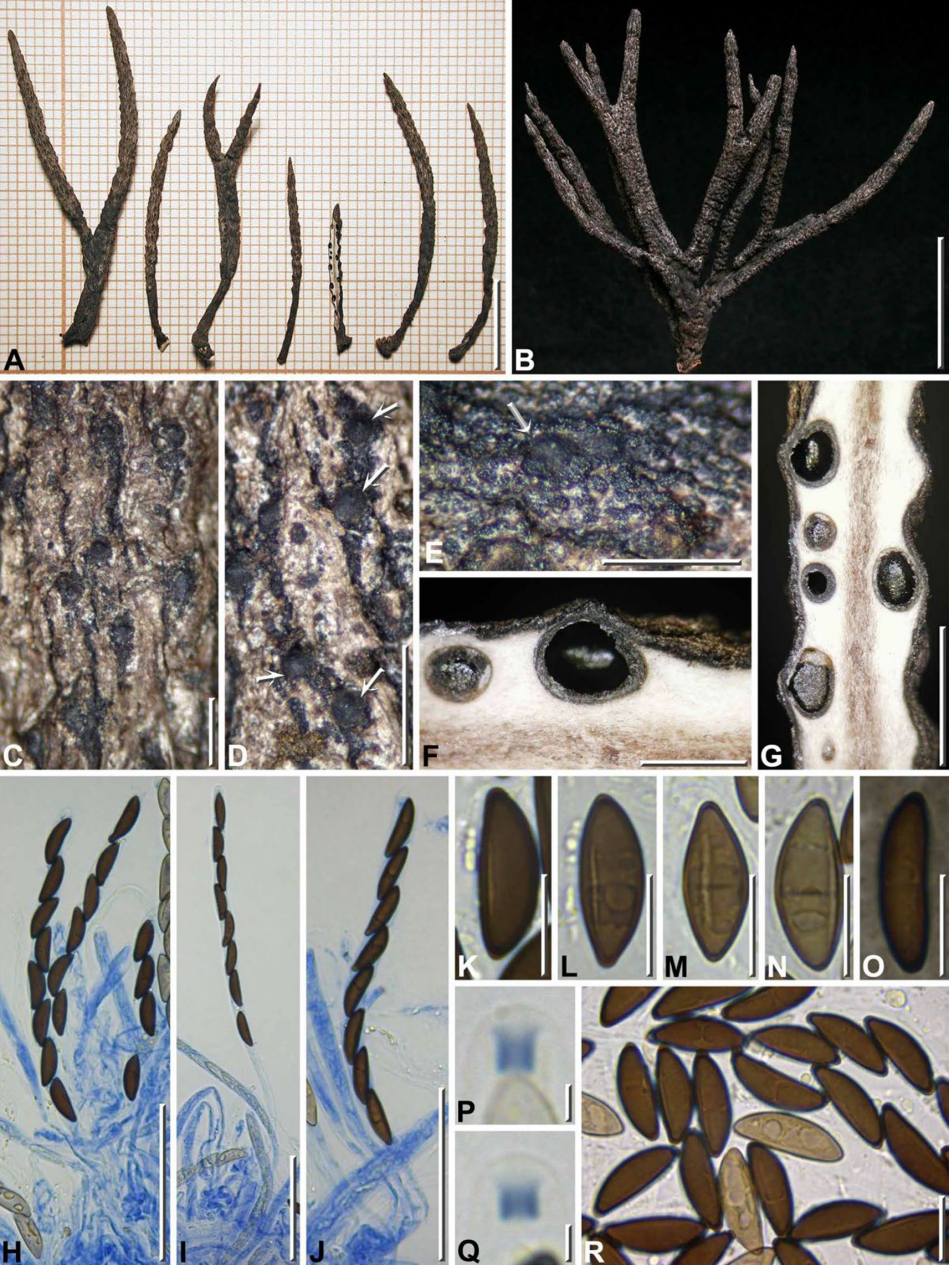
*Xylaria piloides* (**A, D, F, G, J–R ***CLL7081* holotype; **B, C, E ***CLL9073*). **A** Simple to furcate stromata. **B** Densely ramified stroma. **C** Surface of a young stroma with thick dirty gray felty coating through which black ostioles emerge. **D** Stromatal surface in close-up showing grayish scales and black prominent ostioles (arrows). **E** Stromatal surface in close-up showing clusters of shiny black granules around an ostiole (arrow). **F** Stroma in longitudinal section showing a slightly exposed perithecium immersed beneath a thin black crust. **G** Stroma in longitudinal section showing scattered depressed-spherical perithecia and a white interior with a pale orange-brown core. **H** 4-, 6- and 8-spored asci. **I** Long-stipitate ascus. **J** Short-stipitate ascus (**H–J** in blue Pelikan ink). **K, L** Mature ascospores in ventral view showing a germ slit. **M, N** Immature ascospores in ventral view showing a germ slit (**K–N** in 1% SDS). **O** Ascospore in India ink showing absence of sheath or appendage. **P, Q** Ascal apical apparati in Melzer’s reagent. **R** Mature and immature ascospores, in 1% SDS. Bars in **A, B** = 10 mm; **C, G** = 1 mm; **D–F** = 0.5 mm; **H–J** = 50 µm; **K–O**, **R** = 10 µm **P, Q** = 2 µm

***Xylaria piloides*** J. Fourn., Y.-M. Ju, H.-M. Hsieh & Lechat, sp. nov. Figure 55, Table 35

**Table 35:** Ascospore dimensions from the two known collections of *X. piloides*

Collection numbers	Ascospore measurements with extreme values in parentheses	Q = quotient l/w, *N* = number of measurements	Me = mean values of l/w, Qe = mean quotient l/w
*CLL7081* holotype	(15.5–)15.9–17.6(−18.4) × (5.1–)5.3–6.2(−6.7) µm	Q = (2.5–)2.7–3.2(−3.3), *N* = 60	Me = 16.7 × 5.8 µm, Qe = 2.9
*CLL9073*	(14.2–)15.1–17.2(−17.7) × (4.5–)4.9–5.7(−6.0) µm	Q = (2.6–)2.8–3.4(−3.7), *N* = 60	Me = 16.2 × 5.2 µm, Qe = 3.1
**Cumulated values**	**(14.2–)15.1–17.6(−18.4) × (4.5–)4.9–6.2(−6.7) µm**	**Q = (2.5–)2.7–3.4(−3.7), N = 120**	**Me = 16.4 × 5.5 µm, Qe = 3.0**

MycoBank MB863180

**Typification**: **FRENCH GUIANA.** Sinnamary, Paracou, CIRAD field centre, Guyaflux plot, 5.249218 N, 52.915431 W, lowland rainforest, on dead corticated branch, 28 Feb 2007, *Lechat, C. CLL7081* (holotype HAST 147434).

**Etymology**: From Greek *πιλο-ειδής* (*pilo-eides*) = felt-like, for the remarkable felty, thick and dirty gray splitting outer layer.

**Diagnosis**: Differs from the known species of *Xylaria* with needle-shaped stromata by the combination of a felty, thick and dirty gray splitting outer layer with large raised-discoid ostioles and fusiform-inequilateral ascospores 16.1 × 4.9 µm on average with a short ventral germ slit.

**Stromata** gregarious, 12–46 mm in total height, the fertile parts 4–33 mm high × 1.2–3.5 mm wide, simple to furcate or branching from the base, terete to flattened, straight to slightly curved, apically sterile, mucronate to sharply pointed; the stipes 5–15 mm high, 1–2.5 mm wide, ill-defined, terete to flattened, black, puckered, glabrous to partly tomentose, with a swollen base. Surface blackish, slightly nodulose with faintly exposed perithecial contours, coated with a thick, dull brownish gray felty outer layer splitting into elongated stripes, gradually wearing off with age, revealing superficial grayish remnants incrusted with clusters of minute shiny black granules; stromatal crust just beneath surface black, leathery, 40–50 µm thick; interior white, solid, fibrous, with an orange-brown core. **Perithecia** scattered to crowded, subglobose 0.40–0.50 mm diam to depressed-spherical, 0.30–0.45 mm high × 0.60–0.75 mm diam. **Ostioles** conspicuous, black, dome-shaped to raised-discoid, 120–170 µm diam at base.

**Paraphyses** hyphal, hyaline, remotely septate, simple to branched at base, 6–8 µm wide at base, tapering to 1.5–2 µm wide, embedded in mucilaginous material. **Asci** cylindrical, with (6–)8 uniseriately arranged slightly overlapping ascospores, the spore-bearing parts 97–124 µm long × 6.2–7.0 µm wide, the stipes 36–80 µm long, fragile, with apical apparatus short-tubular, apically slightly flared with a faint upper rim, 1.8–2.3 × 1.6–2.0 µm (Me = 2.0 × 1.8 µm, *N* = 25), bluing in Melzer’s reagent. **Ascospores** (14.2–)15.1–17.6(−18.4) × (4.5–)4.9–6.2(−6.7) µm, Q = (2.5–)2.7–3.4(−3.7), *N* = 120 (Me = 16.4 × 5.5 µm, Qe = 3.0), fusiform-inequilateral with narrowly rounded ends, medium brown in the dry state, with a fairly conspicuous straight germ slit ½–2/3 spore-length on the ventral side, lacking sheath or appendage; epispore smooth.

**Asexual morph** on the natural substrate not observed.

**Other specimen examined (paratype)**: **FRENCH GUIANA.** Macouria, vicinity of Cayenne, Risquetout Forest, trail to Saut-Léodate, 4.899372 N, 52.555061 W, ca. 55 m, lowland rainforest, on dead wood, 25 Apr 2009, *Lechat, C. CLL9073* (HAST 147435).

**Known distribution**: French Guiana, known from two collections.

**Comments**: *Xylaria piloides* is diagnosed by slightly nodulose, needle-shaped stromata coated with a thick felty gray outer layer splitting into elongated stripes, associated with prominent black ostioles. It is noteworthy that a great variation in stroma dimensions and branching pattern can be observed between the two collections examined, confirming once again that surface characters are more consistent and taxonomically more informative than the branching pattern.

Moreover, ascospores of *X. piloides* are distinctive by their shape, dimensions, and germ slit morphology, making it readily distinguished from akin species. *Xylaria scabra* (this paper) appears to be the most resembling species based on its stromata overlain with a gray outer layer and fusoid-inequilateral ascospores with narrowly rounded ends. It can be distinguished from *X. piloides* by more strongly nodulose and wrinkled stromata and darker brown, larger ascospores averaging 19.7 × 5.8 µm with a germ slit slightly less than spore-length.

Pairwise alignment of ITS sequences shows that material collected in Ecuador assigned to *X. fissilis* (= *X. cylindrica*) by Vandegrift et al. (2023) has only 87.63% similarity to verified material of the species from the French West Indies, but 99.4% similarity to *X. piloides*. This strongly suggests that the known distribution of *X. piloides* extends to Ecuador, pending morphological confirmation. *Xylaria cylindrica* and *X. piloides* indeed share similar ascospores, which likely accounts for the questionable identification; however, *X. cylindrica* differs mainly by stromata that are apically obtusely rounded, with a strongly carbonaceous texture, a blackish interior, and lacking a gray felty outer layer (Fournier et al. 2020).

**Fig. 56:**
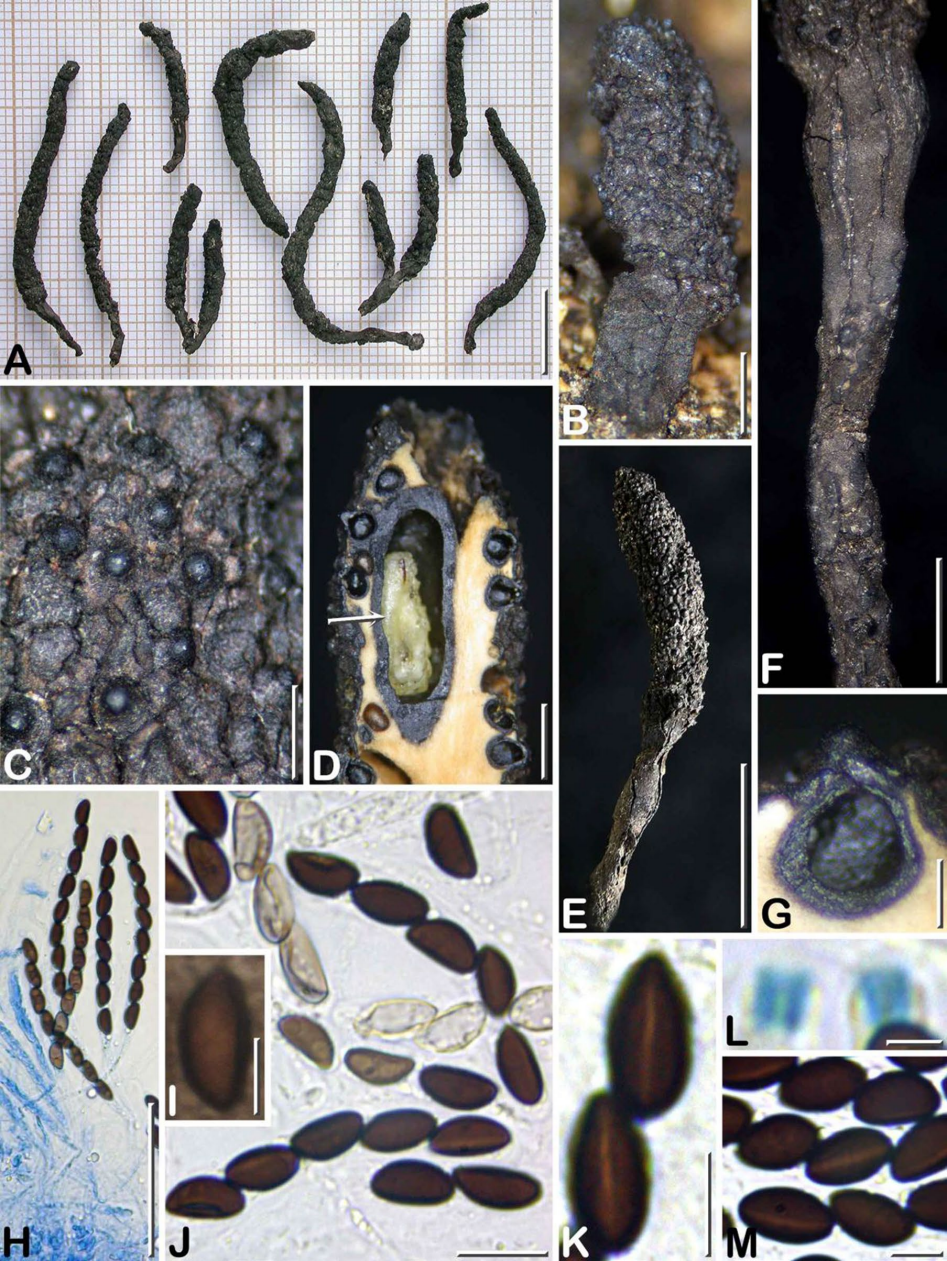
*Xylaria rhytidophloea* (**A, C–L ***GYJF21174*; **B, M ***GYJF21033*). **A** Variously bent cylindric stromata. **B, E** Stromata showing a corky-cracked surface and smooth glabrous stipes. **C** Close-up on stromatal surface roughened by blackish brown scales and shiny black ostioles. **D** Damaged apical region of a stroma in longitudinal section associated with an encysted insect nymph (arrow). **F** Glabrous smooth to faintly cracked surface of a stipe. **G** Perithecium in vertical section beneath a thin crust and a prominent ostiole. **H** Bunch of asci in blue Pelikan ink. **I** Ascospore in side view lacking a sheath or appendages, in India ink. **J, M** Mature and immature ascospores, some showing a ventral germ slit, in 1% SDS. **K** Ascospores in ventral view showing a germ slit, in 1% SDS. **L** Ascal apical apparati in Melzer’s reagent. Bars in **A** = 10 mm; **B, D** = 1 mm; **C** = 0.5 mm; **E** = 5 mm; **F** = 2 mm; **G** = 0.2 mm; **H** = 50 µm; **I, K, M** = 5 µm; **J** = 10 µm; **L** = 2 µm

***Xylaria rhytidophloea*** Mont., Ann. Sci. Nat. Bot., sér. IV, 3: 101. 1855. Figure 56, Table 36

**Table 36:** Ascospore and ascal apical apparatus dimensions in two collections of *X. rhytidophloea*

Collection numbers	Ascospore measurements with extreme values in parentheses	Q = quotient l/w, *N* = number of measurements	Me = mean values of l/w, Qe = mean quotient l/w	Ascal apical apparatus h × w, *N* = 25
*GYJF21033*	(8.1–)8.2–9.4(−10.1) × (4.0–)4.2–4.8(−5.1) µm	Q = (1.7–)1.8–2.1(−2.4), *N* = 60	Me = 8.7 × 4.5 µm, Qe = 1.9	1.8–2.2 × 1.6–1.9 µm, Me = 2.0 × 1.7 µm
*GYJF21174*	(7.5–)7.7–8.9(−9.3) × (4.0–)4.1–4.7(−4.8) µm	Q = 1.7–2.1(−2.3), *N* = 60	Me = 8.4 × 4.4 µm, Qe = 1.9	1.7–2.0 × 1.4–1.7 µm, Me = 1.8 × 1.6 µm
**Cumulated values**	**(7.5–)7.7–9.4(−10.1) × (4.0–)4.1–4.8(−5.1) µm**	**Q = (1.7–)1.8–2.1(−2.4), N = 120**	**Me = 8.6 × 4.5 µm, Qe = 1.9**	**Me = 1.9 × 1.7 µm**

**Stromata** upright to prostrate, loosely scattered, simple to occasionally connate at base by 2, arising in linear rows or in bunches from black swellings erumpent from bark, 5–35 mm in total height, the fertile head 1.8–3 mm diam, cylindrical to subfusiform, straight to curved, irregularly constricted, apically rounded and fertile or broken or damaged by insects larvae, brittle, with perithecial contours unexposed; surface blackish brown to blackish, glabrous, densely corky-cracked into minute, polygonal, thick warts and roughened by conspicuous ostioles; the stipes usually well-defined, 1.5–8.0 mm high × 0.7–1.4 mm diam, straight to slightly curved, terete to slightly flattened, dull brownish black, entirely glabrous, smooth apart from major cracks lengthwise, swollen at base; subsurface a black leathery crust 30–50 µm thick; interior solid, soft-textured, white, yellowish with age or when parasitized by insects larvae. **Perithecia** subglobose, 0.38–0.45 mm diam, laterally flattened when in contact. **Ostioles** bluntly papillate on a broadly rounded, discoid to conical base, shiny black, 120–170 µm diam, up to 150 µm high, mostly conspicuously prominent above the cracked superficial crust.

**Paraphyses** sparse, hyphal, remotely septate, thin-walled, 4.0–5.0 µm wide at base, tapering to 1.5–2 µm wide above asci, discreetly embedded in mucilaginous material. **Asci** cylindrical, with (6–)8 slightly overlapping uniseriately, occasionally biseriately arranged ascospores, the spore-bearing parts 62–68 × 5.2–6.6 µm, the stipes 55–63 µm long, with apical apparatus short cylindric to quadrate, frequently slightly attenuated at base, slightly higher than wide, apically flared and flattened with a faint lateral rim, 1.7–2.0 × 1.6–1.9 µm (Me = 1.9 × 1.7 µm, *N* = 50), bluing in Melzer’s reagent. **Ascospores** (7.5–)7.7–9.4(−10.1) × (4.0–)4.1–4.8(−5.1) µm, Q = (1.7–)1.8–2.1(−2.4) *N* = 120 (Me = 8.6 × 4.5 µm, Qe = 1.9), ellipsoid-inequilateral, with narrowly to most often broadly rounded ends, dark brown, unicellular, with a conspicuous, straight, longitudinally oriented germ slit almost spore-length on the ventral side; lacking appendage or mucilaginous sheath; epispore smooth.

**Asexual morph** on the natural substrate not observed.

**Specimens examined**: **FRENCH GUIANA.** Roura, Cacao, Camp Bonaventure, 4.323472 N, 52.338502 W, ca. 70 m, hygrophilic secondary rainforest, on dead corticated trunk, 21 Mar 2021, *Fournier, J. GYJF21033* (HAST 147436); Maripasoula, Saül, trail head to Sentier des Gros Arbres, 3.6201 N, 53.207989 W, ca. 210 m, disturbed mesophilic rainforest, on dead corticated branch, 27 Mar 2021, *Fournier, J. GYJF21174* (HAST 147437); Cayenne, on wood, *Leprieur, F. R.* (holotype of *X. rhytidophloea* PC ex Montagne herb. 10130, isotypes K(M) 111865, PC).

**Known distribution**: French Guiana.

**Comments**: The rather vague taxonomic concept of *X. rhytidophloea* is based on the protologue (Montagne 1855) for material collected in French Guiana, in which the species was diagnosed by short, finely reticulately cracked stipes (*tenuissime rugoso-reticulatis*) and slightly prominent (*prominulis*) ostioles. However, examination of the type collection at PC, which shows smooth and glabrous stipes, further challenged this concept. *Xylaria rhytidophloea* was not reported by other authors, apart from Papua New Guinea by Dennis (1974), who regarded it as a synonym of *X. feejeensis* (Berk.) Fr. Both species indeed share similar morphological traits but differ in ascospore shape (Fournier et al. 2020).

During the present study, several collections referable to *X. rhytidophloea* became available and showed consistent morphological characteristics, including ascospore morphology and dimensions. A further problem may arise when two different but very similar species occur mixed on the same substrate, which happened twice. Phylogenetic comparison of these collections resulted in a clade containing several distinct species, all clearly distant from *X. feejeensis*, but related to *X. martinicensis* and *X. palifera*, and to *X. intracolorata* and *X. plebeja,* with the latter two species known from South East Asia.

In addition to the phylogenetic data supporting the distinctiveness of all above species, *X. rhytidophloea* can be distinguished from the three latter species and from *X. uncinata* (described herein) by lacking a citrine-yellow or orange-yellow layer of tissue embedding the perithecia. It also differs from *X. subplebeja* (described herein) by a smooth stipe vs corky-cracked and from *X. crustulata* (described herein) by a glabrous vs hairy stipe.

**Fig. 57:**
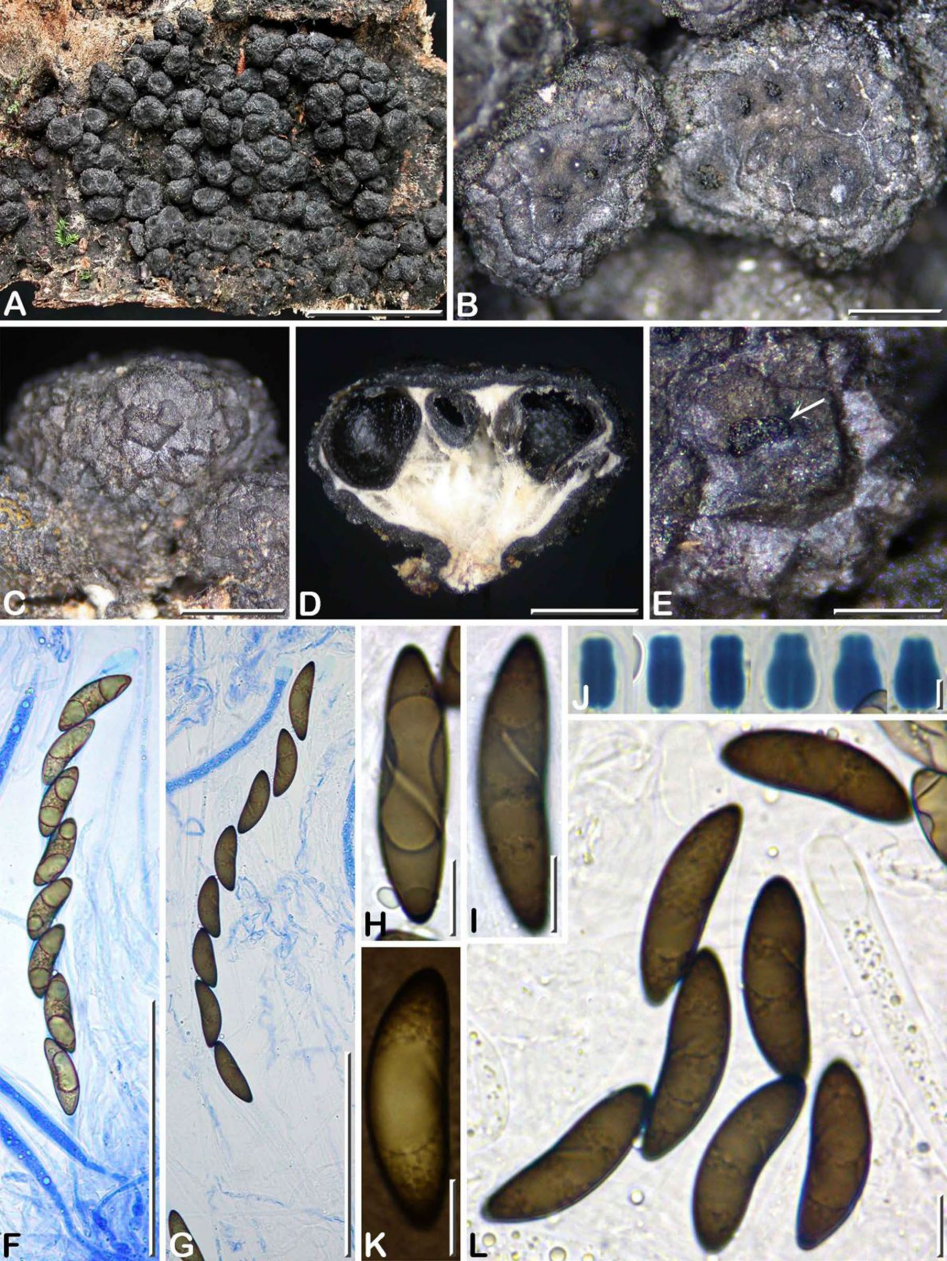
*Xylaria rhytidosperma* (*GYJF21191*). **A** Crowded stromata on bark. **B** Two adjacent stromata in top view. **C** Turbinate stroma in side view showing thick lateral polygonal warts. **D** Stroma in vertical section showing a carbonaceous crust and a narrow central connective. **E** Edge of a stroma in close-up showing thick marginal warts and an ostiole (arrow) obscured by ejected ascospores. **F** Immature ascus and partly collapsed paraphyses. **G** Mature ascus (**F** and **G** in blue Pelikan ink). **H, I** Ascospores respectively in ventral and side view showing a short oblique and sigmoid germ slit, in 1% SDS. **J** Variously shaped ascal apical apparati, in Melzer’s reagent. **K** Ascospore in India ink showing absence of sheath or appendages. **L** Ascospores in side view, in 1% SDS. Bars in **A** = 10 mm; **B–D** = 1 mm; **E** = 0.5 mm; **F, G** = 100 µm; **H, I, K, L** = 10 µm; **J** = 5 µm

***Xylaria rhytidosperma*** J. Fourn. & Lechat, Ascomycete.org 10 (4): 168. 2018. Figure 57

**Stromata** kretzschmarioid, densely clustered, turbinate, subsessile, flat-topped, 1.8–3.5 mm high, 1.3–3.6 mm diam, the lower part obconical ending into a narrow central connective; surface blackish brown and plane on top, even, lacking exposed perithecial contours, at sides blackish and coarsely cracked into thick polygonal to rhomboid warts, especially at the margin of upper part; subsurface a carbonaceous brittle crust 80–100(−210) µm thick including warts; interior white, loosely fibrous, radiating from the basal connective. **Perithecia** (1–)3–8 per stroma, subglobose, 0.9–1.2 mm diam. **Ostioles** obtusely papillate with ill-defined contours, barely prominent, porate, dull black.

**Paraphyses** hyphal, remotely septate, thin-walled, 4.5–10 µm wide at base, tapering to 1.5–2.0 µm wide above asci, embedded in mucilaginous material **Asci** 6–8-spored, cylindrical, short-stipitate, the spore-bearing parts 180–245 × 14–18 µm, the stipes 70–90 µm long, with apical apparatus urn-shaped to almost tubular with an obtuse upper rim, 11.2–13.5 × 5.7–8.2 µm (Me = 12.3 × 6.9 µm, *N* = 25), strongly bluing in Melzer’s reagent. **Ascospores** (27.2–)29.9–34.3(−37.8) × (8.9–)9.4–11.0(−11.6) µm, Q = (2.5–)2.9–3.5(−3.8), *N* = 60 (Me = 32.0 × 10.0 µm, Qe = 3.2), ellipsoid-inequilateral to navicular, ventrally concave, mostly with narrowly rounded ends, dark brown, with a conspicuous, obliquely oriented and slightly sigmoid germ slit ca. ½ spore-length on the ventral side; lacking appendages or mucilaginous sheath; epispore smooth.

**Specimens examined: FRENCH GUIANA.** Maripasoula, Saül, trail head toward Sentier des Gros Arbres, 3.620139 N, 53.208103 W, ca. 210 m, disturbed rainforest, on dead woody liana, 25 Aug 2018, *Fournier, J. GYJF18151* (HAST 147438); Maripasoula, Saül, trail head to Sentier des Gros Arbres, 3.6201 N, 53.207989 W, ca. 210 m, disturbed secondary rainforest, on dead corticated branch, 27 Mar 2021, *Fournier, J. GYJF21191* (HAST 147439).

**Asexual morph** on the natural substrate not observed.

**Known distribution**: French Guiana; the French West Indies (Guadeloupe, Martinique).

**Comments**: In our phylogenetic analysis, the collections from French Guiana cluster tightly with the isotype of *X. rhytidosperma* from Martinique, and their ascospores exhibited rod-like structures beneath the epispore after two years of storage in the herbarium. These collections show several morphological deviations from the original description (Fournier et al. 2018c), including the lack of associated coremioid structures, flat-topped stromata with a smooth and even ostiolar area, and the absence of the rod-like structures beneath the epispore prior to long storage. The latter trait may be misleading: although diagnostic, we failed to observe it in freshly collected and gently air-dried material. We assume that it appears only after intense drying or prolonged desiccation, and thus may be interpreted as an artifact. The effect of short, intense drying on an electric device was not tested, but it was likely the reason for the misinterpretation in Fournier et al. (2018c).

Theissen (1909b) described *Hypoxylon verrucosum* Theiss., a penzigioid *Xylaria* from Brazil similar in stromatal outline, but its type material [**BRAZIL.** on wood, *Theissen, F.* (lectotype S F44668 ex Bresadola herb.)] differs mainly by ascospores having a straight germ slit much less than spore-length, despite similar shape and dimensions.

***Xylaria rickii*** Theiss., Ann. Mycol. 6: 342. 1908.

The cited collection from French Guiana closely matches those reported and illustrated from the French West Indies (Fournier et al. 2020).

**Specimen examined: FRENCH GUIANA.** Maripasoula, Saül, village, northern edge of Carbets du bord lodge, 3.623598 N, 53.210338 W, ca. 210 m, disturbed rainforest, on dead blackened branchlet on the ground, 19 Aug 2018, *Fournier, J. GYJF18004* (HAST 147440).

**Fig. 58:**
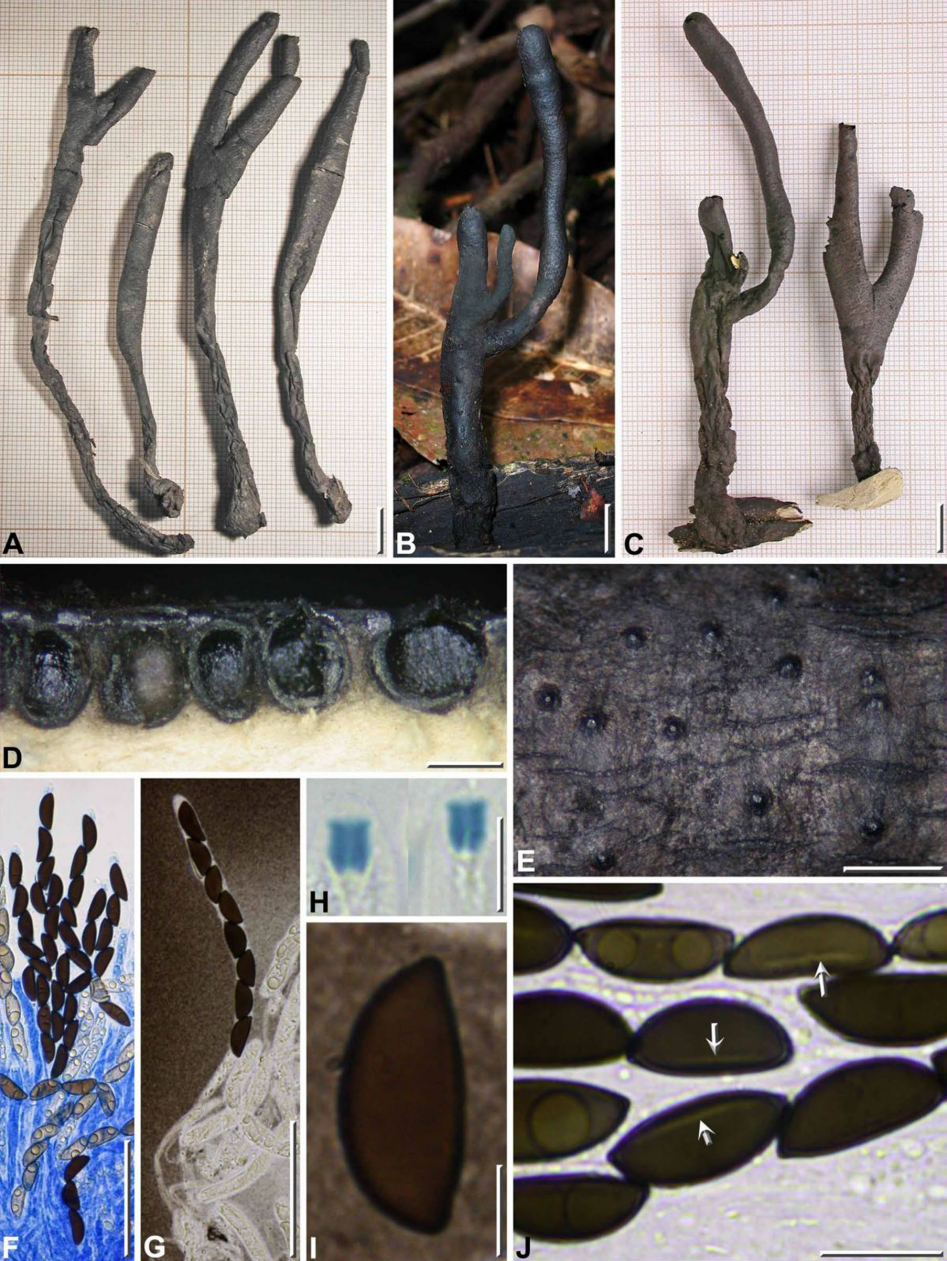
*Xylaria robusta* (**A, J ***CLL10051*; **B–I ***GYJF21137* holotype). **A, C** Stromata in the dry state. **B** Stroma in the fresh state in situ. **D** Stroma in longitudinal section showing perithecia immersed beneath a thick carbonaceous crust and showing a solid whitish fibrous interior. **E** Stromatal surface in close-up showing shiny black ostioles and a loose network of shallow longitudinal cracks. **F** Bunch of mature asci, in blue Pelikan ink. **G** Mature ascus featuring an intact stipe, in India ink. **H** Ascal apical apparati in Melzer’s reagent. **I** Ascospore in side view in India ink showing an absence of sheath or appendage. **J** Ascospores, those in ventral view showing a germ slit (arrows), in 1% SDS. Bars in **A–C** = 10 mm; **D, E** = 0.5 mm; **F, G** = 50 µm; **H, I** = 5 µm; **J** = 10 µm

***Xylaria robusta*** J. Fourn., Y.-M. Ju, H.-M. Hsieh & Lechat, sp. nov. Figure 58, Table 37

**Table 37:** Ascospore and ascal apical apparatus dimensions in three collections of *X. robusta* showing a fairly wide variation range

Collection numbers	Ascospore measurements with extreme values in parentheses	Q = quotient l/w, *N* = number of measurements	Me = mean values of l/w, Qe = mean quotient l/w	Ascal apical apparatus h × w, *N* = 20
*CLL10051*	(11.9–)13.0–15.2(−15.9) × (4.2–)5.0–6.0(−6.2) µm	Q = (2.2–)2.4–2.9(−3.0), *N* = 60	Me = 14.1 × 5.5 µm, Qe = 2.6	2.5–2.9 × 1.8–2.0 µm, Me = 2.7 × 1.9 µm
*GYJF18153*	(11.3–)11.9–14.3(−15.1) × (5.0–)5.2–5.8(−6.0) µm	Q = (2.0–)2.1–2.6(−2.9), *N* = 60	Me = 13.1 × 5.5 µm, Qe = 2.4	
*GYJF21137* holotype	(12.1–)12.8–14.9(−15.8) × (4.7–)5.2–5.9(−6.3) µm	Q = (2.0–)2.3–2.7(−3.1), *N* = 60	Me = 13.7 × 5.5 µm, Qe = 2.5	2.8–3.1 × 2.0–2.4 µm, Me = 3.0 × 2.2 µm
**Cumulated values**	**(11.3–)11.9–15.2(−15.9) × (4.2–)5.0–6.0(−6.3) µm**	**Q = (2.0–)2.1–2.9(−3.1), N = 180**	**Me = 13.6 × 5.5 µm, Qe = 2.5**	**2.5–3.1 × 1.8–2.4 µm, Me = 2.9 × 2.1 µm**

MycoBank MB863183

**Typification**: **FRENCH GUIANA.** Maripasoula, Saül, trail head to Sentier des Gros Arbres, 3.620139 N, 53.208103 W, ca. 210 m, disturbed secondary rainforest, on dead blackened wood, 26 Mar 2021, *Fournier, J. GYJF21137* (holotype HAST 147443).

**Etymology**: From Latin *robustus* = robust, for the tall and stout stromata.

**Diagnosis**: Differs from similar species with robust cylindrical stromata and a carbonaceous crust by dark brown navicular ascospores averaging 13.6 × 5.5 µm, with narrowly rounded to subacute ends and a germ slit 1/2–2/3 spore-length.

**Stromata** 75–115 mm in total height, upright, the fertile parts 35–53 mm high × 6–9 mm wide, subcylindrical to flattened with broadly rounded fertile apices, simple to most often furcate, straight to slightly curved, hard-textured, not splitting upon drying but readily broken; the stipes ill-to well-defined, 24–65 mm high, 4–9 mm wide, longitudinally puckered, black, smooth, glabrous, swollen and felty-tomentose at base; surface brownish black, smooth to faintly bumpy in lower part, lacking a peeling outer layer, with perithecial contours unexposed, just roughened by ostioles and narrow, shallow, longitudinal linear to sinuous cracks forming a loose network; stromatal crust black, carbonaceous, 100–130 µm thick; the tissue around perithecia faintly brownish; interior solid, whitish to ivory, fibrous, turning hollow in age. **Perithecia** crowded, immersed, subglobose, 0.4–0.6 mm diam, occasionally laterally flattened. **Ostioles** conspicuous, rounded-papillate, shiny black, 100–120(−170) µm diam at base.

**Paraphyses** hyphal, hyaline, remotely septate, 4.0–4.5 µm wide at base, tapering to 0.8–1.5 µm wide above asci, discreetly embedded in mucilaginous coating. **Asci** cylindrical, with (4–6–)8 uniseriately arranged, slightly to strongly overlapping ascospores, the spore-bearing parts 88–107 µm long × 7.8–9.5 µm wide, the stipes 60–97 µm long, fragile, with apical apparatus tubular to slightly urn-shaped with a faint upper rim, 2.5–3.1 × 1.8–2.4 µm (Me = 2.9 × 2.1, *N* = 40), bluing in Melzer’s reagent. **Ascospores** (11.3–)11.9–15.2(−15.9) × (4.2–)5.0–6.0(−6.3) µm, Q = (2.0–)2.1–2.9(−3.1), *N* = 180 (Me = 13.6 × 5.5 µm, Qe = 2.5), ellipsoid-inequilateral to navicular with narrowly rounded to subacute, occasionally faintly pinched ends or lower end beaked, ventrally flat to convex, rarely slightly concave, unicellular, dark brown to blackish brown, with a narrow, ventral, straight, longitudinally oriented germ slit ½ to 2/3 spore-length; lacking sheath or appendages; epispore smooth.

**Asexual morph** on the natural substrate not observed.

**Other specimens examined (paratypes)**: **FRENCH GUIANA.** Régina, road to Saint-Georges, PK 167.7, hygrophilic rainforest, on dead wood, 28 Apr 2010, *Lechat, C. CLL10051* (HAST 147441); Maripasoula, Saül, trail head to Sentier des Gros Arbres, 3.620839 N, 53.208536 W, ca. 210 m, disturbed secondary rainforest, on dead blackened wood, 25 Aug 2018, *Fournier, J. GYJF18153* (HAST 147442), depauperate.

**Known distribution**: French Guiana, known from the three cited collections.

**Comments**: *Xylaria robusta* is diagnosed by tall, cylindrical to flattened, frequently branched, carbonaceous stromata with a brownish black surface lacking a peeling outer layer and roughened by conspicuous, shiny black ostioles. Its navicular dark brown ascospores 12–15 × 5.0–6.0 µm with acute ends and a germ slit much less than spore-length contribute to setting it apart from outwardly similar species.

*Xylaria robusta* shares most morphological characters with *X. allantoidea* (Berk.) Fr., *X. cubensis* (Mont.) Fr. and *X. flabelliformis* (Schwein.) Fr. The main differences with these widely distributed species lie in its subcylindrical stromata with a thick pannose stipe and its larger and differently shaped ascospores (Fournier et al. 2019).

Callan and Rogers (1990) described and cultured *X.* cf. *allantoidea* from French Guiana, which differs from *X*. *robusta* by hollow inrolled stromata 8–15 mm diam on a long narrow stipe 2–3 mm wide and narrowly ellipsoid ascospores 12–13.5(−14) × 4–4.5(−5) µm.

*Xylaria nigromedullosa* Trierveiler-Pereira & A.I. Romero was described from Brazil by Trierveiler-Pereira et al. (2009), featuring dark brown cylindro-clavate stromata. It is distinguished from *X*. *robusta* by a blackish solid interior and smaller ellipsoid-inequilateral blackish brown ascospores 7–9.5 × 4–5 µm.

Two species documented in the present study have similar robust cylindrical stromata. *Xylaria holmbergii* differs by smaller dark brown reniform ascospores averaging 8.9 × 4.5 µm and by leathery stromata with a corky-cracked surface. *Xylaria crassipes* is the phylogenetically most closely related species and can be distinguished by subreniform ascospores averaging 13.1 × 5.9 µm, with broadly rounded ends and a longer germ slit.

**Fig. 59:**
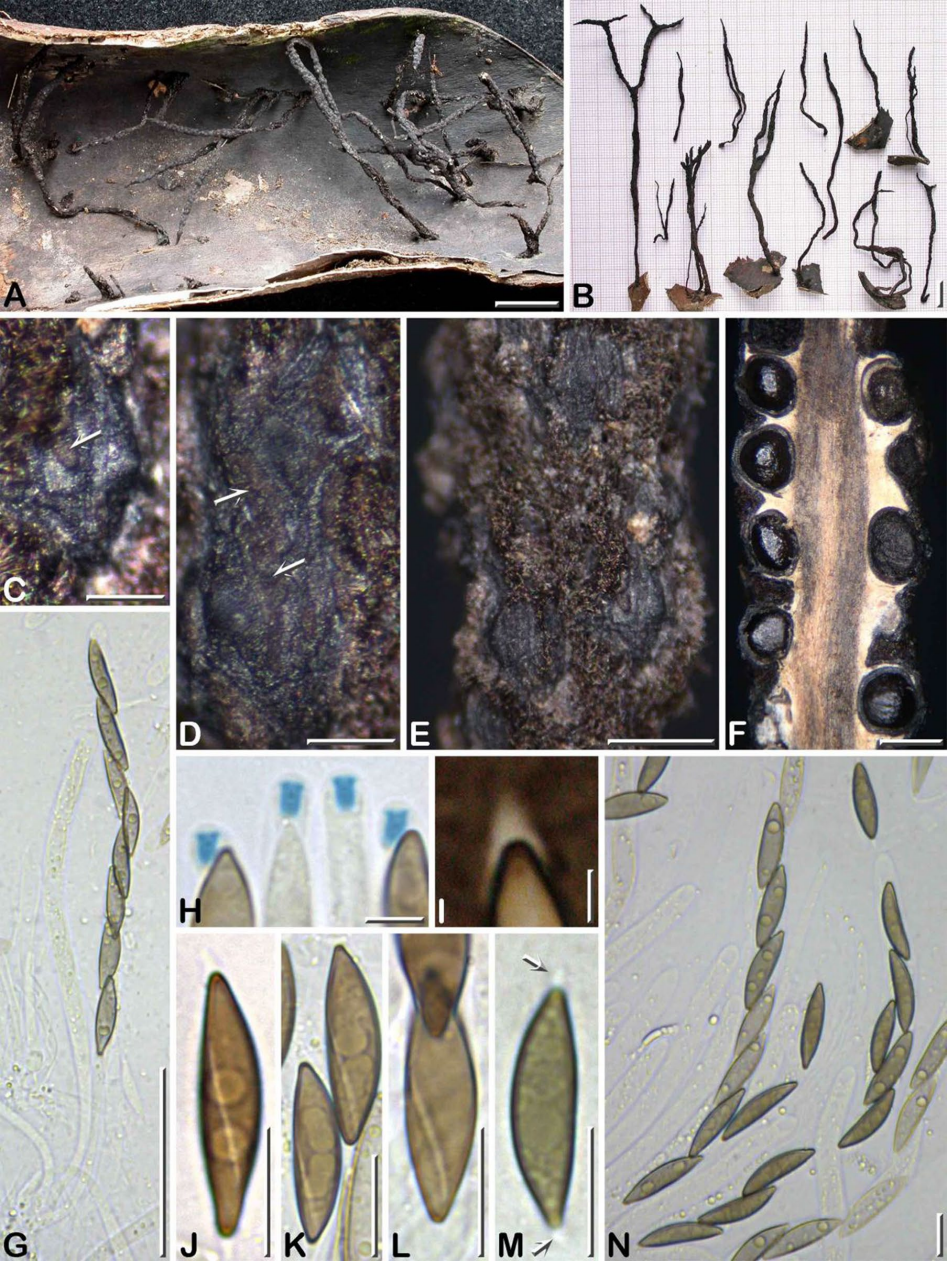
*Xylaria rossmaniae* (*GYJF21009*). **A** Stromata arising from the inner side of a split pod of *Eperua*. **B** Variously shaped stromata. **C** Perithecial mound in side view showing a papillate ostiole and a blackish brown longitudinal stripe (arrow). **D** Stromatal surface in close-up showing superficial blackish stripes (arrows) with remnants of tomentum. **E** Stromatal surface in close-up showing tufts of a dark brown tomentum between perithecial mounds. **F** Stroma in longitudinal section showing spaced perithecia and a tan to brown core. **G** Ascus in 1% SDS. **H** Ascal apical apparati, in Melzer’s reagent. **I** Ascospore end with a pointed appendage, in India ink. **J–L** Ascospores in ventral view showing a germ slit, respectively in 1% SDS, aqueous nigrosin and Melzer’s reagent. **M** Ascospore in side view showing bipolar refractive appendages (arrows), in diluted India ink. **N** Immature and mature ascospores in the fresh state, in 1% SDS. Bars in **A, B** = 10 mm; **C, D** = 0.2 mm; **E, F** = 0.5 mm; **G** = 50 µm; **H** = 5 µm; **I** = 2 µm; **J–N** = 10 µm

***Xylaria rossmaniae*** Y.-M. Ju, J.D. Rogers & H.-M. Hsieh. Mycologia 110: 744. 2018. Figure 59

**Stromata** upright to prostrate, straight to contorted, scattered, isolated to connate at base, cylindrical to slightly flattened, simple to dichotomously branched 1–2 times, 22–100 mm in total height, with apiculate to filiform sterile apices, stipitate; the fertile head 11–32 mm high × 1.0–1.8 mm wide, surface brownish black, slightly nodulose with perithecial contours more or less exposed, emerging from dense tufts of reddish brown tomentum, overlain by a thick blackish brown outer layer splitting longitudinally into persistent stripes bearing the tomentum; subsurface crust leathery, 40–60 µm thick; interior solid, whitish, with a tan to brownish core developing with age; the stipes ill-defined, 11–62 mm high × 0.6–2.4 mm wide, black, terete to flattened, longitudinally puckered, entirely tomentose, the base slightly swollen. **Perithecia** subglobose to depressed-spherical, 0.40–0.60 mm diam. **Ostioles** finely to obtusely papillate, often inconspicuous, dull black, 40–80(−100) µm diam at base, occasionally on a slightly raised-discoid base up to 150 µm diam.

**Paraphyses** hyphal, copious, hyaline, simple, remotely septate, 3–4 µm wide at base, tapering to 1.0–1.5 µm wide, minutely guttulate, discreetly embedded in mucilaginous material. **Asci** cylindrical, with (6–)8 uniseriately arranged, slightly overlapping ascospores, the spore-bearing parts 103–115 µm long × 6.0–7.8 µm wide, the stipes 48–60 µm long, with apical apparatus tubular with a marked upper rim, 2.6–3.2 × 1.5–1.9 µm (Me = 2.9 × 1.7 µm, *N* = 25), bluing in Melzer’s reagent. **Ascospores** (16.3–)17.5–20.1(−21.1) × (4.3–)4.5–5.1(−6.6) µm, Q = (3.0–)3.5–4.2(−4.4), *N* = 60, (Me = 18.7 × 4.9 µm, Qe = 3.8), fusiform-inequilateral with narrowly rounded to subacute, occasionally slightly pinched ends, light brown, with an oblique, slightly sigmoid germ slit almost spore-length on the ventral side; with a thin hyaline sheath swelling at both ends to form acute, triangular refractive secondary appendages, not stained by aqueous nigrosin even after prolonged incubation; epispore smooth.

**Asexual morph** on the natural substrate not observed.

**Specimen examined: FRENCH GUIANA.** Roura, Cacao, Camp Bonaventure, 4.323044 N, 52.339371 W, ca. 60 m, hygrophilic secondary rainforest, on inner side of dead pods of *Eperua falcata* (Fabaceae) in the leaf litter, 20 Mar 2021, *Fournier, J. GYJF21009* (HAST 147444).

**Known distribution**: French Guiana (this paper); Venezuela (Ju et al. 2018), as *X*. cf. *ianthinovelutina* in Rogers et al. (1988).

**Comments**: *Xylaria rossmaniae* is diagnosed by filiform stromata with sterile apices and a slightly nodulose, strongly tomentose surface, traits characteristic of *X. ianthinovelutina* and related species occurring on woody, mostly fabaceous pods. It is clearly distinguished by light brown, narrowly fusiform-inequilateral ascospores with an oblique, slightly sigmoid germ slit and pointed bipolar secondary appendages (Ju et al. 2018). *Xylaria rossmaniae* was previously known only from the type collection made in Venezuela, from which the Guianese material deviates only by slightly larger ascospores averaging 18.7 × 4.9 µm vs 15.4 × 4.2 µm. The presence of a thick striped outer layer, from which the tomentum originates, can only be detected in overmature stromata where the tomentum has gradually disappeared in a way similar to what occurs in *X. ianthinovelutina* (Fournier et al. 2020, and this paper).

**Fig. 60:**
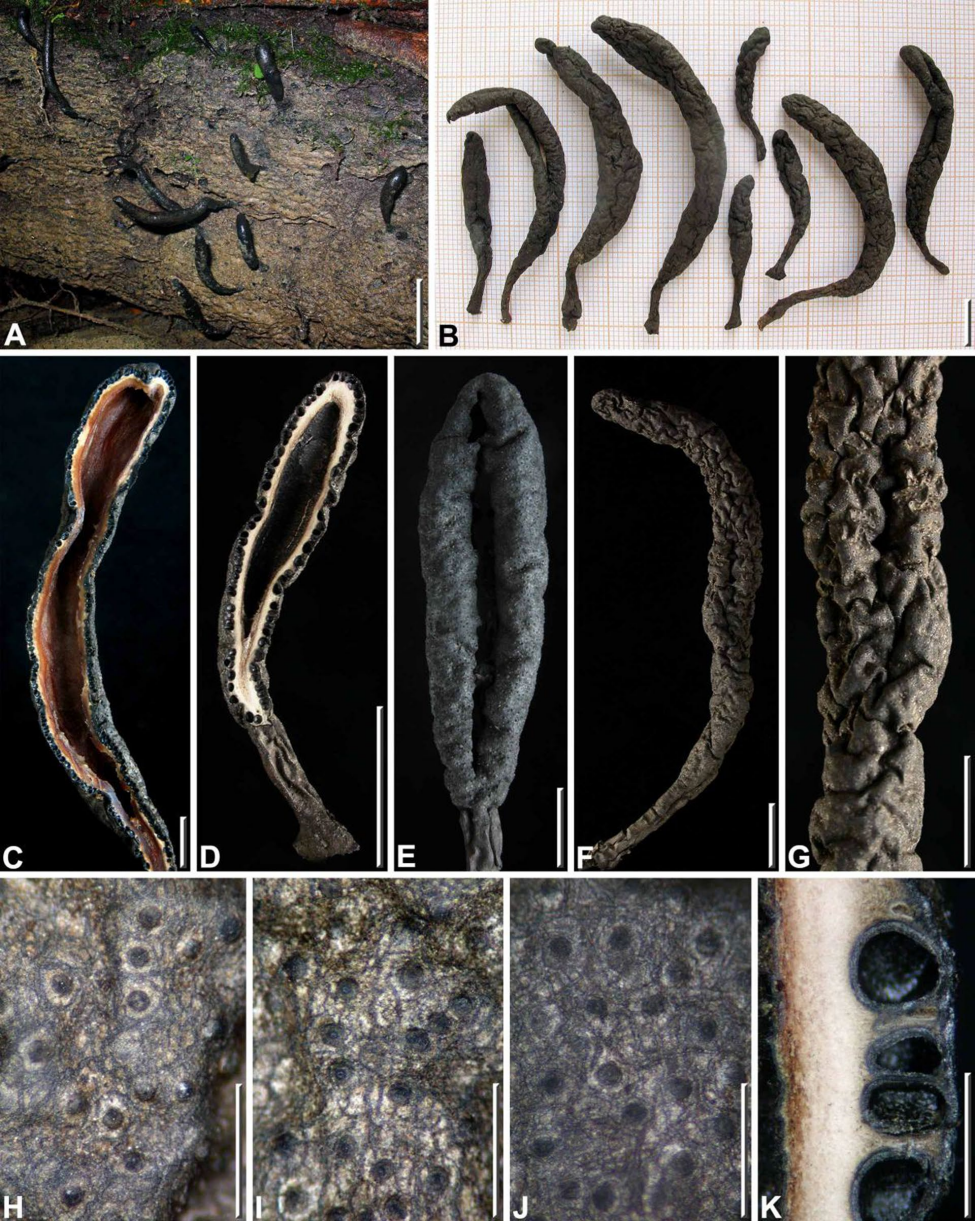
*Xylaria saulensis* (**A ***GYJF21319*; **B–E, H–K ***GYJF19318* holotype; **F, G ***GYJF21410*). **A** Turgid stromata in the fresh state, in situ. **B** Longitudinally split or with cerebriform surface stromata in the dry state. **C** Rehydrated stroma in vertical section showing a hollow interior with reddish gelatinous wall. **D** Stroma in the dry state in vertical section showing a black hollow interior. **E** Longitudinally split and weakly wrinkled stroma. **F, G** Deeply shriveled stroma with cerebriform surface. **H–J** Stromatal surface at various stages of development showing ostioles and a vanishing off-white to gray superficial layer forming persistent grayish halos around them. **K** Stroma in longitudinal section showing perithecia beneath a thin black crust and embedded in a thin layer of whitish spongy tissue. Bars in **A** = 50 mm; **B–D, F** = 10 mm; **E, G** = 5 mm; **H–K** = 1 mm

**Fig. 61:**
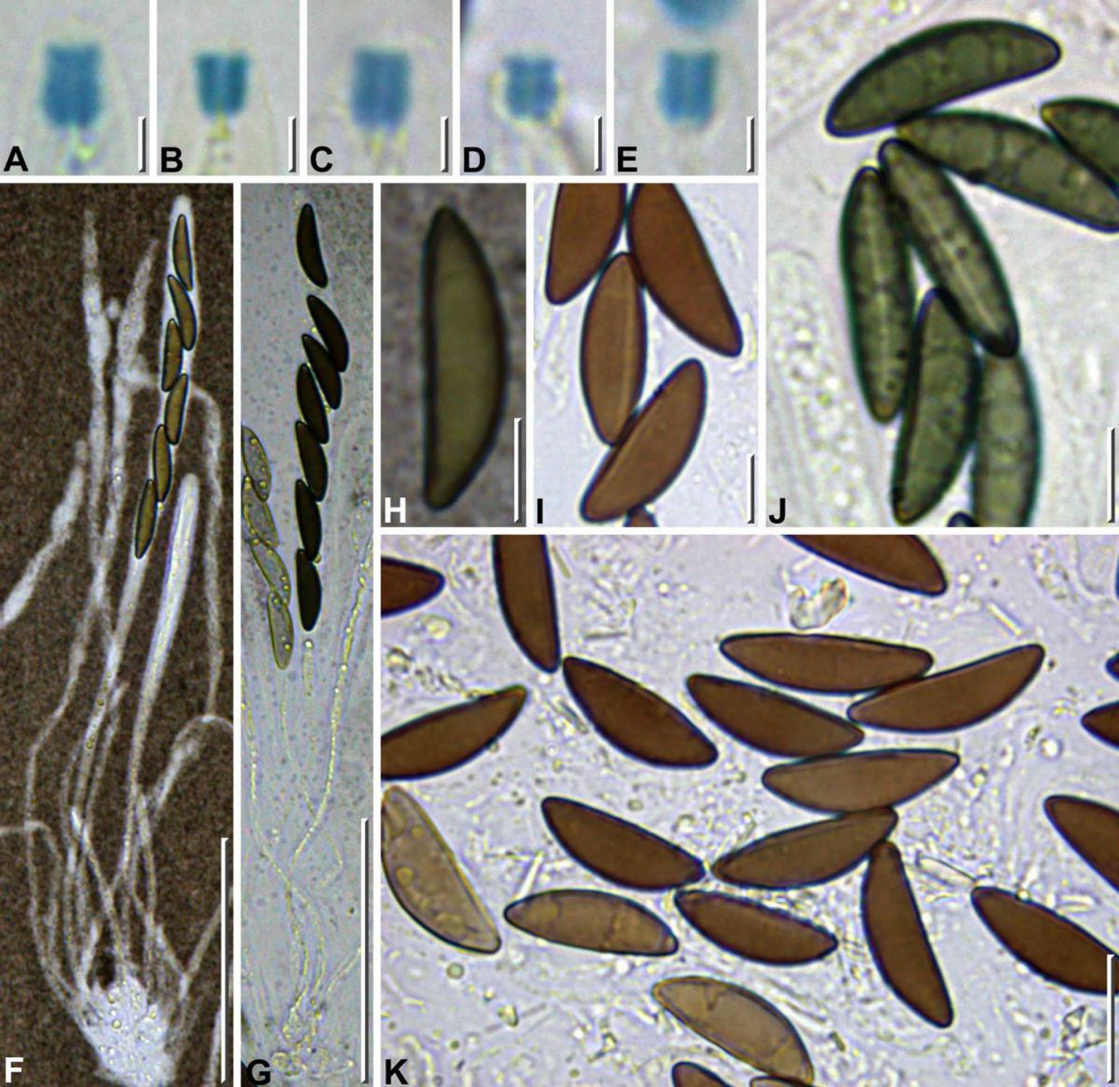
*Xylaria saulensis* (**A, J ***GYJF19178*; **B, C ***GYJF21410*; **D, F–I, K ***GYJF19138* holotype; **E ***GYJF19213*). **A–E** Ascal apical apparati in Melzer’s reagent. **F, G** Respectively six- and seven-spored asci, in India ink. **H** Ascospore in India ink showing an absence of sheath or appendages. **I** Ascospores from herbarium material, one in ventral view showing a germ slit. **J** Ascospores in the fresh state, some showing a ventral germ slit. **K** Mature and immature ascospores in side view (**I–K** in 1% SDS). Bars in **A–E** = 2 µm; **F, G** = 50 µm; **H–J** = 5 µm; **K** = 10 µm

**Fig. 62:**
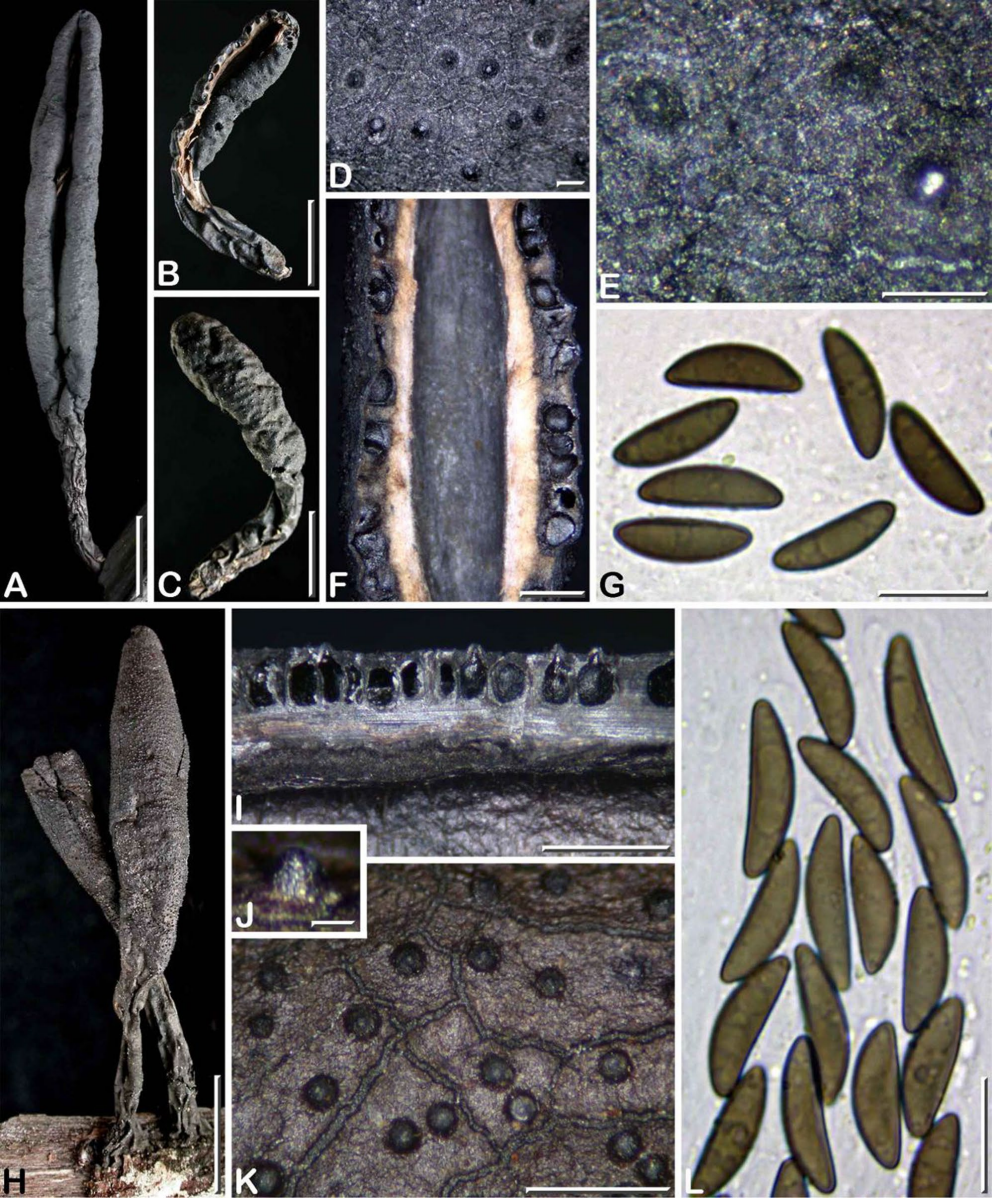
*Xylaria saulensis* (**A, D–G ***GYJF21011*; **B, C ***GYJF21013*; **H–L ***GYJF21260*). **A** Vertically split fusoid stroma in the dry state. **B, C** Clavate stromata in the dry state. **D, E, K** Stromatal surface in close-up showing ostioles and a network of shallow cracks. **F** Stroma in longitudinal section showing a hollow interior lined with a black layer. **G, L** Ascospores in side view, in 1% SDS. **H** Two adjacent fusoid stromata in the dry state. **I** Stroma in longitudinal section showing an entirely black hollow interior. **J** Close up on an ostiole in side view. Bars in **A, H** = 10 mm; **B, C** = 5 mm; **D, E** = 0.2 mm; **F, I** = 1 mm; **J** = 100 µm; **K** = 0.5 mm; **G, L** = 10 µm

***Xylaria saulensis*** J. Fourn., Y.-M. Ju, H.-M. Hsieh & Lechat, sp. nov. Figures 60, 61, 62, Table 38

**Table 38:** Ascospore dimensions in nine collections of *X. saulensis* showing the range of intraspecific variations

Collection numbers	Ascospore measurements with extreme values in parentheses	Q = quotient l/w, *N* = number of measurements	Me = mean values of l/w, Qe = mean quotient l/w
*GYJF19003*	(12.9–)13.6–15.5(−17.0) × (3.0–)3.4–4.1(−4.3) µm	Q = (3.2–)3.5–4.4(−5.0), *N* = 60	Me = 14.4 × 3.7 µm, Qe = 3.9
*GYJF19138*	(12.3–)12.8–15.0(−16.3) × (3.1–)3.3–4.1(−4.7) µm	Q = (2.8–)3.3–4.3(−4.6), *N* = 60	Me = 13.9 × 3.7 µm, Qe = 3.8
*GYJF19178*	(11.3–)12.3–14.3(−14.9) × (3.0–)3.3–4.0(−4.2) µm	Q = (3.0–)3.3–4.0(−4.4), *N* = 60	Me = 13.1 × 3.6 µm, Qe = 3.6
*GYJF19213*	(12.7–)13.4–15.5(−18.5) × (3.3–)3.5–4.0(−4.4) µm	Q = (3.1–)3.5–4.3(−5.1), *N* = 60	Me = 14.4 × 3.8 µm, Qe = 3.8
*GYJF21011*	(10.1–)10.8–12.3(−13.8) × (3.1–)3.3–4.0(−4.4) µm	Q = (2.6–)2.9–3.6(−3.8), *N* = 60	Me = 11.6 × 3.7 µm, Qe = 3.2
*GYJF21013*	(11.0–)11.6–13.9(−14.8) × (3.3–)3.5–4.0(−4.4) µm	Q = (3.0–)3.1–3.6(−3.7), *N* = 60	Me = 12.6 × 3.8 µm, Qe = 3.4
*GYJF21252*	(12.9–)13.6–16.1(−17.3) × (3.7–)3.9–5.0(−5.1) µm	Q = (2.7–)3.0–3.9(−4.3), *N* = 60	Me = 15.0 × 4.4 µm, Qe = 3.4
*GYJF21260*	(12.4–)13.1–15.2(−17.0) × (3.5–)3.7–4.2(−4.6) µm	Q = (3.1–)3.3–4.0(−4.5), *N* = 60	Me = 14.3 × 4.0 µm, Qe = 3.6
*GYJF21410*	(12.9–)13.2–15.0(−16.6) × (3.4–)3.5–4.1(−4.4) µm	Q = (3.1–)3.4–4.2(−4.4), *N* = 60	Me = 14.1 × 3.8 µm, Qe = 3.7
**Cumulated values**	(10.1–)11.6–16.1(−18.5) × (3.0–)3.3–5.0(−5.1) µm	Q = (2.6–)2.9–4.4(−5.1), *N* = 540	Me = 13.7 × 3.8 µm, Qe = 3.6

MycoBank MB863184

**Typification**: **FRENCH GUIANA.** Maripasoula, Saül, shortcut toward the airfield, 3.622159 N, 53.204166 W, disturbed secondary rainforest, on dead trunk, 20 Jun 2019, *Corriol, G. GYJF19138* (holotype HAST 147450).

**Etymology**: From Saül, the place where most collections come from.

**Diagnosis**: Differs from *X. allantoidea* and resembling species by stromata with a thinner, less carbonaceous black outer crust, frequently deeply shriveling or splitting upon drying and featuring a grayish, reticulately cracked coating, a hollow interior, and ascospores averaging 14.2 × 3.8 µm.

**Stromata** scattered to loosely gregarious, upright, straight to curved, 25–80 mm in total height, the fertile head 22–63 mm high, 3–12 mm wide, fusiform to clavate, stipitate, with broadly rounded fertile apices, becoming hollow upon drying and shriveling, more rarely splitting lengthwise; surface brownish to dull black, strongly convoluted in the dry state due to deep wrinkles, with unexposed perithecial contours; with a thin, appressed, minutely cracked, persistent whitish to gray superficial pellicle, gradually worn off but forming persistent discoid halos around the ostioles; subsurface crust leathery to slightly carbonaceous 50–60(−80) µm thick; interior comprising a narrow whitish spongy layer embedding the perithecia and a central cavity lined by a reddish gelatinous tissue, filled with liquid or not in the fresh state, this tissue turning black in the dry state; the stipes usually well-defined, 8–25 mm high, 1.5–5 mm wide, black, shriveled, glabrous, swollen to discoid at base. **Perithecia** crowded, subglobose, laterally flattened when in contact, 0.45–0.70 mm diam. **Ostioles** discoid to obtusely papillate, weakly prominent, dull black, 80–125 µm diam at base.

**Paraphyses** hyphal, hyaline, remotely septate, simple, 5–7 µm wide at base, tapering to 1.5–2 µm wide, discreetly embedded in mucilaginous material. **Asci** cylindrical, with (6–)8 uniseriately arranged, slightly overlapping ascospores, the spore-bearing parts 70–80 × 8–9(−11) µm, the stipes 70–95 µm long, with apical apparatus short-cylindrical to slightly urn-shaped, with a faint upper rim, 1.9–3.3 × 1.5–2.3 µm (Me = 2.5 × 1.8 µm, *N* = 90), bluing in Melzer’s reagent. **Ascospores** (10.1–)11.6–16.1(−18.5) × (3.0–)3.3–5.0(−5.1) µm Q = (2.6–)2.9–4.4(−5.1) *N* = 540 (Me = 13.7 × 3.8 µm, Qe = 3.6), narrowly fusiform-inequilateral with narrowly rounded ends, ventrally flat to slightly convex, rarely slightly concave, medium brown with olivaceous tone in the fresh state, unicellular, with a narrow, straight, longitudinally oriented germ slit almost spore-length on the ventral side, without sheath or appendages; epispore smooth.

**Asexual morph** on the natural substrate not observed.

**Other specimens examined (paratypes**): **FRENCH GUIANA.** Roura. Cacao, Camp Bonaventure, 4.323044 N, 52.339371 W, ca. 60 m, hygrophilic secondary rainforest, on dead blackened wood, 20 Mar 2021, *Fournier, J. GYJF21011* (HAST 147445); Roura. Cacao, Camp Bonaventure, 4.323044 N, 52.339371 W, ca. 60 m, hygrophilic secondary rainforest, on dead blackened wood, 20 Mar 2021, *Fournier, J. GYJF21013* (HAST 147446); Maripasoula, Saül, trail head toward Roche-Bateau, Crique-Cochon, 3.625994 N, 53.1955 W, 210–220 m, disturbed secondary rainforest, on dead blackened wood, 31 Mar 2021, *Fournier, J. GYJF21325* (HAST 147447); Maripasoula, Saül, trail head toward Roche-Bateau, 3.620498 N, 53.199309 W, disturbed secondary rainforest, at the base of dead trunk, 22 Jun 2019, *Corriol, G. GYJF19188* (HAST 147448); Maripasoula, Saül, trail head to Sentier des Gros Arbres, 3.620967 N, 53.208689 W, disturbed secondary rainforest, on dead trunk, 16 Jun 2019, *Fournier, J. GYJF19003* (HAST 147449); Maripasoula, Saül, trail to Monts-La Fumée, tributary of Crique Queue-Hocco, 3.637173 N, 53.204727 W, ca. 260 m, disturbed mesophilic rainforest, on dead trunk, 4 Apr 2021, *Fournier, J. GYJF21410* (HAST 147451); Maripasoula, Saül, trail head toward Roche-Bateau, Crique-Cochon, 3.625994 N, 53.1955 W, 210–220 m, disturbed secondary rainforest, on dead corticated branch, 31 Mar 2021, *Fournier, J. GYJF21330* (HAST 147452); Maripasoula, Saül, trail head toward Roche-Bateau, 3.620498 N, 53.199309 W, disturbed secondary rainforest, on dead trunk, 22 Jun 2019, *Lechat, C. GYJF19213* (HAST 147453); Maripasoula, Saül, trail head toward Roche-Bateau, Crique-Cochon, 3.625994 N, 53.1955 W, 210–220 m, disturbed secondary rainforest, on dead blackened wood, 31 Mar 2021, *Fournier, J. GYJF21299* (HAST 147454); Maripasoula, Saül, trail head toward Roche-Bateau, 3.620498 N, 53.199309 W, disturbed secondary rainforest, on dead wood, 22 Jun 2019, *Fournier, J. GYJF19178* (HAST 147455); Maripasoula, Saül, shortcut toward the airfield, 3.622852 N, 53.205768 W, 210–220 m, disturbed secondary rainforest, on dead blackened wood, 29 Mar 2021, *Fournier, J. GYJF21252* (HAST 147456); Maripasoula, Saül, shortcut toward the airfield, 3.622159 N, 53.204166 W, disturbed secondary rainforest, on dead trunk, 20 Jun 2019, *Fournier, J. GYJF19140* (HAST 147457); Maripasoula, Saül, trail head to Sentier des Gros Arbres, 3.620839 N, 53.208536 W, disturbed secondary rainforest, on dead corticated trunk, 3 Apr 2021, *Fournier, J. GYJF21403* (HAST 147458); Maripasoula, Saül, trail head toward Roche-Bateau, Crique-Cochon, 3.625994 N, 53.1955 W, 210–220 m, disturbed secondary rainforest, on dead trunk, 31 Mar 2021, *Fournier, J. GYJF21319* (HAST 147459); Maripasoula, Saül, shortcut to the airfield, 3.622852 N, 53.205768 W, 210–220 m, disturbed mesophilic rainforest, on dead, partly decorticated branch, 29 Mar 2021, *Fournier, J. GYJF21260* (HAST 147460); Maripasoula, Saül, shortcut toward the airfield, 3.622159 N, 53.204166 W, disturbed secondary rainforest, on dead blackened wood, 20 Jun 2019, *Fournier, J. GYJF19128* (HAST 147461); Maripasoula, Saül, trail head toward Roche-Bateau, Crique-Cochon, 3.625994 N, 53.1955 W, 210–220 m, disturbed secondary rainforest, on dead blackened wood, 31 Mar 2021, *Fournier, J. GYJF21327* (HAST 147462).

**Known distribution**: French Guiana, mostly in the vicinity of Saül.

**Comments**: The robust stromata of *X. saulensis* make it distinctive in the field and evoke species related to the *X. cubensis* group in the PO clade (Hsieh et al. 2010). It can be distinguished from those species by its soft-textured stromata that, when cut longitudinally, reveal a hollow interior inconstantly filled with liquid. Moreover, the stromatal surface bears a thin, minutely cracked whitish pellicle and becomes strongly convoluted upon drying. Its fusiform-inequilateral, medium brown ascospores averaging 14.2 × 3.8 µm and provided with a long straight germ slit support its recognition as a distinct species. These features set it apart from *X. penicilliopsis* (Henn.) Y.-M. Ju [= *X. moelleroclavus* J. D. Rogers, Y.-M. Ju & Hemmes], another robust species with a convoluted surface and hollow interior, which has larger ascospores averaging 18.8 × 6.4 µm and a short, most often oblique germ slit (Fournier et al. 2019).

*Xylaria saulensis* was widely collected around Saül in 2019 and 2021, showing little morphological variation from the description given and illustrated above, except for three slightly divergent collections briefly documented below (Fig. 62). One of these, *GYJF21260*, also comes from Saül but attracted our attention by the combination of more prominent, bluntly papillate to conical ostioles on the stromatal surface and an entirely black, woody interior. Two collections from Camp Bonaventure, *GYJF21011* and *GYJF21013*, differ from typical *X. saulensis* by having the stromatal surface that does not shrivel on drying and does not show a cracked grayish reticulate coating, and by having smaller ascospores averaging 12.1 × 3.8 µm (Table 38). The inclusion of these three collections in *X. saulensis* is supported by our phylogenetic analyses, suggesting that the divergent features represent intraspecific variations.

**Fig. 63:**
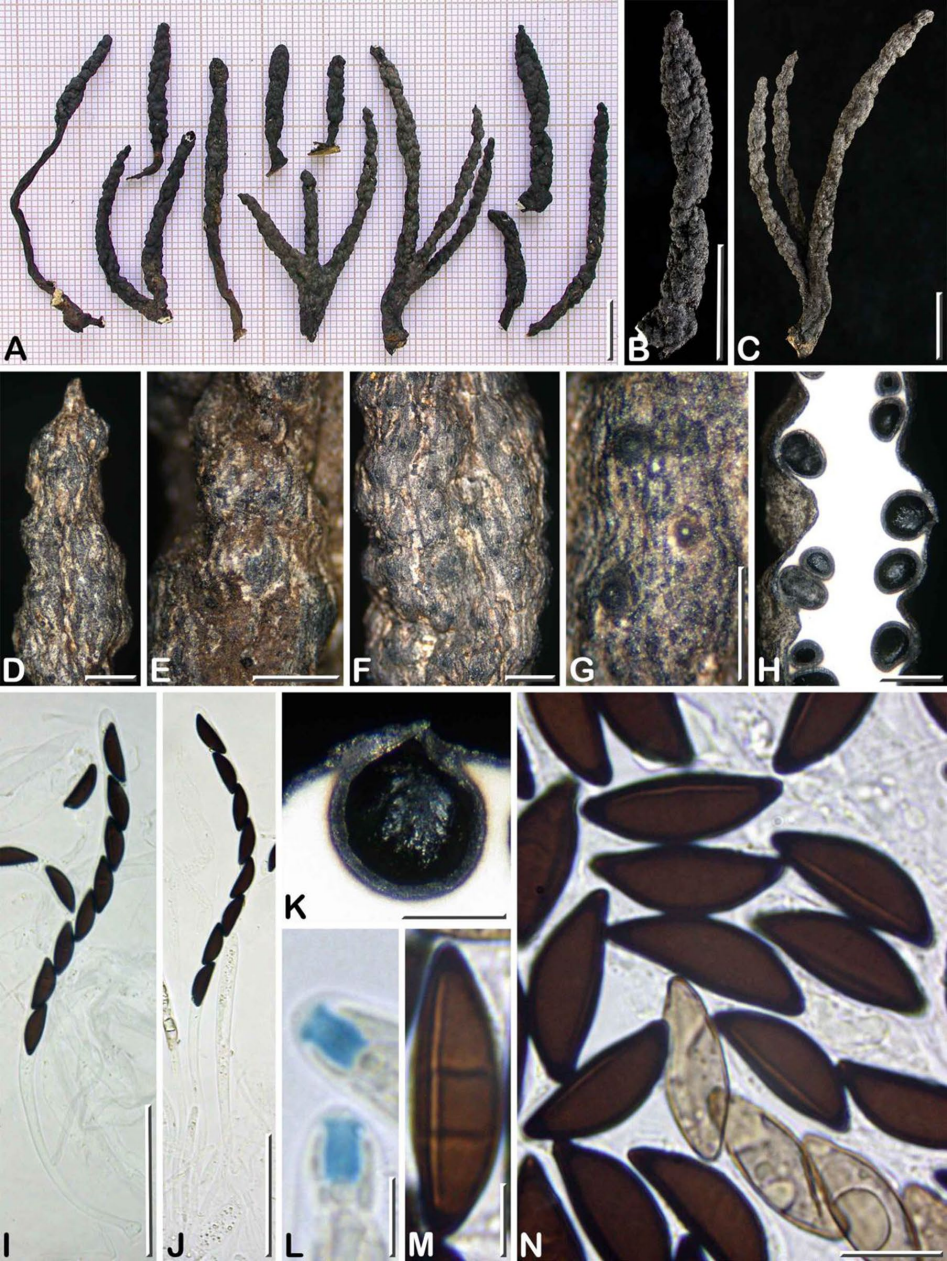
*Xylaria scabra* (*GYJF18104* holotype). **A** Variously branched and stipitate stromata. **B** Fertile part of a stroma with deeply wrinkled surface. **C** Barely mature stromata connate at base with grayish surface. **D** Mucronate apex of a stroma with vertically split gray outer layer. **E** Surface of a young stroma with gray outer layer overlain by a reddish brown fleeting superficial coating. **F** Surface of a mature stroma. **G** Stromatal surface in close-up showing small papillate ostioles and black granulations either scattered or in linear rows. **H** Stroma in longitudinal section showing loosely distributed perithecia embedded in a white and solid tissue. **I, J** Eight-spored asci, respectively in chlorazol black and 1% SDS. **K** Perithecium in vertical section, lying beneath a thin black leathery crust. **L** Ascal apical apparati in Melzer’s reagent. **M** Barely mature ascospore in ventral view showing a germ slit. **N** Mature and immature ascospores, in 1% SDS. Bars in **A–C** = 10 mm; **D–F, H** = 1 mm; **G, K** = 0.5 mm; **I, J** = 50 µm; **L, M** = 5 µm; **N** = 10 µm

***Xylaria scabra*** J. Fourn., Y.-M. Ju, H.-M. Hsieh & Lechat, sp. nov. Figure 63, Table 39

**Table 39:** Ascospore and ascal apical apparatus dimensions in two collections of *X. scabra* showing their variation range, compared with those of *C. piloides*

Collection numbers	Ascospore measurements with extreme values in parentheses	Q = quotient l/w, *N* = number of measurements	Me = mean values of l/w, Qe = mean quotient l/w	Ascal apical apparatus h × w, *N* = 20
*GYJF18091* paratype	(18.0–)18.5–21.4(−22.3) × (5.0–) 5.3–6.1 (−6.7) µm	Q = (2.9–) 3.1–3.8 (−4.2), *N* = 60	Me = 19.9 × 5.7 µm, Qe = 3.5	Me = 4.4 × 2.8 µm
*GYJF18104* holotype	(17.0–)18.0–20.8(−22.4) × (4.9–)5.3–6.2(−6.6) µm	Q = (2.8–)3.0–3.7(−3.9), *N* = 60	Me = 19.4 × 5.8 µm, Qe = 3.3	Me = 4.0 × 2.4 µm
**Cumulated values**	**(17.0–)18.0–21.4(−22.4) × (4.9–)5.3–6.2(−6.7) µm**	**Q = (2.8–)3.0–3.8 (−4.2), N = 120**	**Me = 19.7 × 5.8 µm, Qe = 3.4**	**Me = 4.2 × 2.6 µm**
*X. piloides* (this paper)	(13.8–)15.2–16.9(−18.6) × (4.0–)4.3–5.4(−6.1) µm	Q = (2.8–)2.9–3.7(−4.0), *N* = 120	Me = 16.1 × 4.9 µm, Qe = 3.3	Me = 2.0 × 1.8 µm

MycoBank MB863185

**Typification**: **FRENCH GUIANA.** Maripasoula, Saül, trail head to Roche-Bateau, 3.619046 N, 53.200658 W, disturbed mesophilic rainforest, on big dead corticated liana lying on the ground, mixed with *X.* cf. *scruposa*, 23 Aug 2018, *Fournier, J. GYJF18104* (holotype HAST 147463).

**Etymology**: From Latin *scaber =* rough, for the stromatal surface roughened by exposed perithecial contours, deep wrinkles, a gray splitting outer layer, and black granulations.

**Diagnosis**: Differs from *X. piloides* by more nodulose stromata and darker brown, larger ascospores averaging 19.7 × 5.8 µm vs 16.1 × 4.9 µm, with a longer germ slit.

**Stromata** scattered, upright, straight to slightly curved, simple to occasionally forked or connate at base by 2–3, 16–58 mm in total height, easily broken in the dry state, the fertile head cylindrical to slightly flattened, with short apiculate sterile apex, 11–38 mm high × 2.5–4.0 mm diam, on well- to ill-defined blackish glabrous stipes 3–32 mm high × 1.5–3.0 mm diam with slightly enlarged base. Surface gray to dull black, strongly nodulose with circumferential wrinkles and perithecial contours often strongly exposed; outer layer bipartite, the outermost layer reddish brown, fragile, evanescent but leaving a brownish tone on the underlying grayish layer that splits longitudinally into narrow stripes, revealing minute black carbonaceous granulations; subsurface crust leathery, 50–70 µm thick; interior white to yellowish, solid, pithy. **Perithecia** scattered, subglobose, 0.70–0.85 mm diam. **Ostioles** obtusely papillate, black, 80–100 µm diam, occasionally seated on a black, flat discoid base up to 170–200 µm diam.

**Paraphyses** hyphal, hyaline, remotely septate, simple to branched at base, 6–8 µm wide at base, tapering to 1.5–2 µm wide, embedded in mucilaginous material. **Asci** cylindrical, with (4–6–)8 uniseriately arranged, slightly overlapping ascospores, the spore-bearing parts (80–)115–135 µm long × 7.5–9.0 µm wide, the stipes 72–90 µm long, with apical apparatus narrowly urn-shaped with a marked upper rim, 3.9–5.0 × 2.0–3.2 µm (Me = 4.2 × 2.6 µm, *N* = 40), bluing in Melzer’s reagent. **Ascospores** (17.0–)18.0–21.4(−22.4) × (4.9–)5.3–6.2(−6.7) µm, Q = (2.8–)3.0–3.8 (−4.2), *N* = 120 (Me = 19.7 × 5.8 µm, Qe = 3.4), fusiform-inequilateral with narrowly rounded to subacute, slightly pinched ends, dark brown, with a fairly conspicuous straight germ slit slightly less than spore-length on the ventral side, lacking sheath or appendage; epispore smooth.

**Asexual morph** on the natural substrate not observed.

**Other specimen examined (paratype)**: **FRENCH GUIANA.** Maripasoula, Saül, trail head to Roche-Bateau, 3.619046 N, 53.200658 W, disturbed mesophilic rainforest, on dead decorticated wood, 22 Aug 2018, *Fournier, J. GYJF18091* (HAST 147464).

**Known distribution**: French Guiana, known from two collections.

**Comments**: *Xylaria scabra* is diagnosed by cylindrical, nodulose stromata with a short mucronate sterile apex, roughened by deep circumferential to oblique wrinkles, a black surface encrusted with minute black granulations, overlain with a reddish brown to gray outer layer splitting longitudinally into narrow stripes, and dark brown fusiform-inequilateral ascospores with narrowly rounded ends, averaging 19.7 × 5.8 µm.

The stromata of *X. spegazziniana* (this paper) are somewhat similar in their irregular and nodulose outline, but *X. spegazziniana* differs from *X*. *scabra* in lacking black superficial granulations and having much smaller ascospores, averaging 9.7 × 3.9 µm.

*Xylaria scabra* shares many traits with *X. piloides* (this paper), which differs by much less nodulose stromata with a thicker felty outer layer and smaller, lighter brown ascospores averaging 16.1 × 4.9 µm (Table 39) with a shorter germ slit. Smaller ascal apical apparati and more prominent ostioles further distinguish *X. piloides* from *X. scabra.*

*Xylaria laurentii* P. Henn. [**DEMOCRATIC REPUBLIC OF CONGO.** Kasai Prov., Dibele, 22°50‘E, 4°07’ S, on wood, 22 Nov 1903, *Laurent, E. & M.* (holotype of *X. laurentii* BR, isotype S F5852)] was also considered because of its nodulose stromata overlain with a gray outer layer and ascospores 18–22 × 4–6 µm as stated in Dennis (1961) [as *Xylosphaera*]. It differs from *X*. *scabra* by lighter ascospores with a much less than spore-length germ slit.

***Xylaria schweinitzii*** Berk. & M. A. Curtis, J. Acad. Nat. Sci. Philadelphia, ser. II, 2: 284. 1854.

The cited collection from French Guiana closely matches those reported and illustrated from the French West Indies (Fournier et al. 2019).

**Specimen examined: FRENCH GUIANA.** Maripasoula, Saül, trail head to Sentier des Gros Arbres, 3.620839 N, 53.208536 W, ca. 210 m, disturbed mesophilic rainforest, on dead wood, 26 Mar 2021, *Fournier, J. GYJF21128* (HAST 147465).

***Xylaria scruposa*** (Fr.) Fr., Nov. Act. Reg. Soc. Sci. Upsal., ser. III, 1: 127. 1851.

≡ *Sphaeria scruposa* Fr., Elench. Fung. II, p. 55. 1828.

≡ *Hypoxylon scruposum* (Fr.) Mont., Ann. Sci. Nat. Bot., sér. II, 13: 349. 1840.

The cited collection from French Guiana closely matches those reported and illustrated from the French West Indies (Fournier et al. 2020).

**Specimen examined: FRENCH GUIANA.** Maripasoula, Saül, shortcut to the airfield, 3.623524 N, 53.206542 W, disturbed secondary rainforest, ca. 210 m, dead trunk, 16 Jun. 2019, *Fournier, J. GYJF 19017* (JF).

**Fig. 64:**
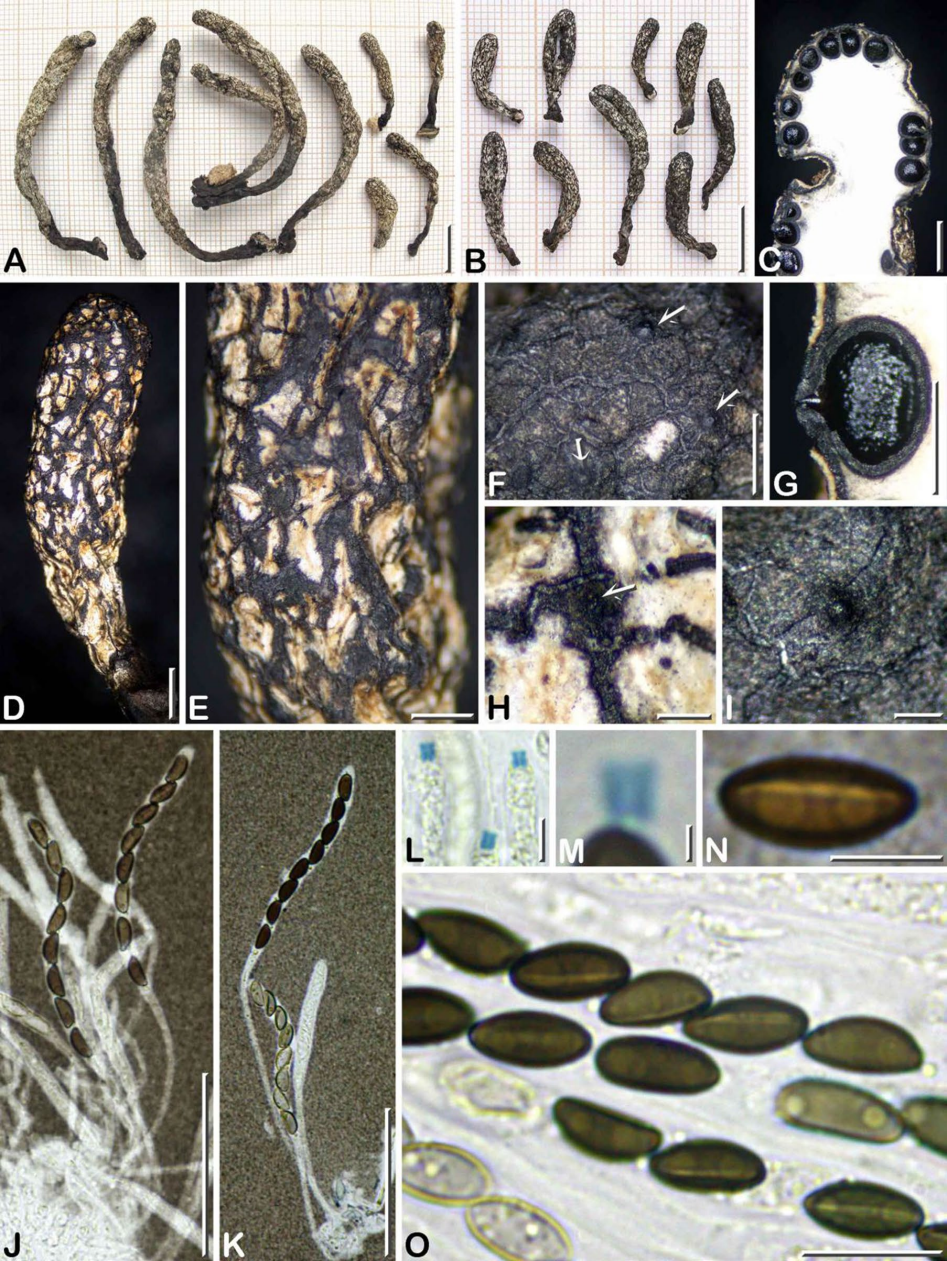
*Xylaria scutulata* (**A, L, O ***GYJF19176* holotype; **B, D–F ***GYJF21034*; **C, G–K, N ***GYJF19126*; **M ***GYJF21118*). **A, B** Stromata in the dry state, some split with inrolled margins in B. **C** Fertile stromatal apex in vertical section. **D** Small clavate stroma in side view. **E** Stromatal surface showing white polygonal scales superficially orange-brown in places. **F** Finely cracked surface of an old stroma with a single remaining white scale and black ostioles (arrows). **G** Perithecium in vertical section. **H** Stromatal surface in close-up showing an ostiole (arrow) opening between white scales. **I** Shiny black conical ostiole on a finely cracked blackish surface. **J, K** Immature and mature asci in India ink. **L, M** Ascal apical apparati in Melzer’s reagent. **N** Ascospore in India ink showing a ventral germ slit and a lack of sheath or appendages. **O** Ascospores in 1% SDS, some in ventral view showing a germ slit. Bars in **A, B** = 10 mm; **C, D** = 1 mm; **E–G** = 0.5 mm; **H, I** = 0.2 mm; **J, K** = 50 µm; **L, N** = 5 µm; **M** = 1 µm; **O** = 10 µm

***Xylaria scutulata*** J. Fourn., Y.-M. Ju, H.-M. Hsieh & Lechat, sp. nov. Figure 64, Table 40

**Table 40:** Ascospore dimensions in twelve collections of *X. scutulata* showing a fairly wide range of intraspecific variations, compared with those of *X. curta* and *X. spegazziniana*

Collection numbers	Ascospore measurements with extreme values in parentheses	Q = quotient l/w, *N* = number of measurements	Me = mean values of l/w, Qe = mean quotient l/w
*CLL10045*	(7.6–)8.1–9.3(−10.1) × (3.2–)3.4–4.0(−4.3) µm	Q = (2.0–)2.1–2.6(−2.7), *N* = 60	Me = 8.7 × 3.7 µm, Qe = 2.3
*GYJF19126*	(7.7–)8.1–9.1(−9.5) × (3.5–)3.6–4.2(−4.4) µm	Q = (1.8–)2–2.3(−2.5), *N* = 60	Me = 8.6 × 3.9 µm, Qe = 2.2
*GYJF19176* holotype	(7.5–)8–9.3(−10.0) × (3.3–)3.6–4.2(−4.5) µm	Q = (1.8–)2–2.5(−2.9), *N* = 60	Me = 8.6 × 3.9 µm, Qe = 2.2
*GYJF19215*	(7.2–)7.9–8.9(−9.5) × (3.3–)3.5–3.9(−4.1) µm	Q = (1.8–)2.1–2.5(−2.7), *N* = 60	Me = 8.4 × 3.7 µm, Qe = 2.3
*GYJF21034*	(7.4–)8.1–9.4(−9.7) × (3.6–)3.9–4.4(−4.5) µm	Q = (1.9–)2–2.3(−2.5), *N* = 60	Me = 8.7 × 4.1 µm, Qe = 2.1
*GYJF21039*	(8.2)8.6–9.7(−9.9) × (3.9–)4.0–4.4(−4.6) µm	Q = 2.0–2.4(−2.5), *N* = 60	Me = 9.1 × 4.2 µm, Qe = 2–2
*GYJF21100*	(7.5–)8.1–9.1(−9.9) × (3.6–)3.7–4.2(−4.6) µm	Q = (1.9–)2–2.3(−2.5), *N* = 60	Me = 8.6 × 4.0 µm, Qe = 2.1
*GYJF21118*	(7.1–)7.6–8.5(−9.4) × (3.4–)3.7–4.1(−4.3) µm	Q = (1.8–)1.9–2.2(−2.6), *N* = 60	Me = 8.1 × 3.9 µm, Qe = 2.1
*GYJF21255*	(7.2–)7.8–8.8 (−9.6) × (3.4–)3.7–4.2(−4.5) µm	Q = (1.8–)1.9–2.4(−2.7), *N* = 60	Me = 8.3 × 3.9 µm, Qe = 2.1
*GYJF21375*	(7.1–)7.8–9.0(−9.5) × (3.6–)3.7–4.3(−4.4) µm	Q = (1.7–)1.9–2.2(−2.4), *N* = 60	Me = 8.3 × 4.0 µm, Qe = 2.1
*GYJF21378*	(7.1–)7.4–8.5(−9.1) × (3.6–)3.9–4.5(−4.7) µm	Q = (1.7–)1.8–2.1(−2.4), *N* = 60	Me = 8.0 × 4.2 µm, Qe = 1.9
*GYJF21391*	(6.9–)7.3–8.3(−9.0) × (3.7–)3.9–4.3(−4.5) µm	Q = (1.6–)1.8–2.0(−2.2), *N* = 60	Me = 7.8 × 4.1 µm, Qe = 1.9
**Cumulated values**	**(6.9–)7.3–9.7(−10.1) × (3.2–)3.4–4.5(−4.7) µm**	**Q = (1.6–)1.8–2.6(−2.9), N = 720**	**Me = 8.4 × 4.0 µm, Qe = 2.1**
*X. curta* (Fournier et al. 2020)	(7.2–)8.0–10.7(−11.1) × (3.1–)3.5–4.4(−4.9) µm	Q = (1.8–)2.1–2.8(−3.1), *N* = 720	Me = 9.2 × 3.9 µm, Qe = 2.3
*X. spegazziniana* (= *X. multiplex* var. *microsperma*) (Fournier et al. 2020)	(8.1–)8.9–10.8(−11.8) × (3.3–)3.5–4.5(−4.8) µm	Q = (1.9–)2.2–2.9(−3.0), *N* = 480	Me = 9.7 × 3.9 µm, Qe = 2.4

MycoBank MB863186

**Typification**: **FRENCH GUIANA.** Maripasoula, Saül, trail head toward Roche-Bateau, 3.620498 N, 53.199309 W, disturbed secondary rainforest, on dead log, 22 Jun 2019, *Fournier, J. GYJF19176* (holotype HAST 147475).

**Etymology**: From Latin *scutulatus* = chequered, referring to the distribution pattern of white scales on the stromatal surface.

**Diagnosis**: Differs from *X. curta* by a thinner, leathery crust, smaller ostioles lacking a discoid base, and slightly smaller, darker brown ascospores with mostly broadly rounded ends.

**Stromata** in small groups, upright, separate, rarely connate at base, ranging from narrowly subcylindrical to short-clavate, simple, terete to flattened, most often curved, with broadly rounded fertile apices, stipitate, 9–50 mm in total height, the fertile head 7–42 mm high × 2–6(−9) mm wide, shrinking upon drying, occasionally splitting lengthwise with inwardly inrolled margins; surface yellowish white to cream-colored due to a thick persistent outer layer splitting into white polygonal scales coated with a vanishing light orange-brown layer, with perithecial contours unexposed; subsurface crust leathery, black, 50–70 µm thick, superficially minutely cracked, showing through the cracks of the whitish outer layer and forming a black reticulate pattern; the stipes 4–20 mm high × 0.8–3 mm wide, often ill-defined, black, terete to flattened, glabrous, cracked, swollen and tomentose at base; interior solid, spongy, white. **Perithecia** subglobose 0.50–0.60 mm diam, to depressed-spherical 0.75 mm diam. **Ostioles** obtusely papillate, dull to shiny black, 80–120 µm diam at base, often ill-distinct, opening in black cracks between the white scales, more conspicuous on old blackish weathered stromata.

**Paraphyses** hyphal, thin-walled, sparse, 6–7 µm wide at base, tapering to 1.5–2 µm wide above asci, embedded in mucilaginous material. **Asci** cylindrical, with (6–)8 slightly overlapping uniseriately arranged ascospores, the spore-bearing parts 58–67 × 5.0–5.5 µm, the stipes 70–90 µm long, with apical apparatus 1.6–2.3 × 1.3–1.7 (Me = 1.9 × 1.5 µm, *N* = 75), short-cylindrical with a faint upper rim, bluing in Melzer’s reagent. **Ascospores** (6.9–)7.3–9.7(−10.1) × (3.2–)3.4–4.5(−4.7) µm Q = (1.6–)1.8–2.6(−2.9), *N* = 720 (Me = 8.4 × 4.0 µm, Qe = 2.1), ellipsoid-inequilateral with narrowly to most often broadly rounded ends, brown to dark brown, unicellular, with a conspicuous, straight, longitudinally oriented germ slit almost spore-length on the ventral side, without sheath or appendages; epispore smooth.

**Asexual morph** on the natural substrate not observed.

**Other specimens examined (paratypes)**: **FRENCH GUIANA.** Maripasoula, Saül, shortcut toward the airfield, 3.622159 N, 53.204166 W, disturbed secondary rainforest, on dead blackened wood, 20 Jun 2019, *Fournier, J. GYJF19126* (HAST 147476); Roura, Cacao, Camp Bonaventure, 4.330514 N, 52.338095 W, 60–65 m, on dead standing trunk, 24 Mar 2021, *Fournier, J. GYJF21118* (HAST 147477); Roura, Cacao, Camp Bonaventure, 4.328545 N, 52.339082 W, 60–65 m, on dead corticated trunk, 23 Mar 2021, *Fournier, J. GYJF21100* (HAST 147478); Roura, Cacao, Camp Bonaventure, 4.323472 N, 52.338502 W, ca. 70 m, disturbed hygrophilic rainforest, on dead corticated trunk, 21 Mar 2021, *Fournier, J. GYJF21034* (HAST 147479); Roura, Cacao, Camp Bonaventure, 4.323472 N, 52.338502 W, ca. 70 m, disturbed hygrophilic rainforest, on dead blackened wood, 21 Mar 2021, *Fournier, J. GYJF21039* (HAST 147480); Régina, banks of Approuague River in a *Theobroma* plantation next to the village, 4.341207 N, 52.110479 W, ca. 20 m, marshy rainforest, on dead wood, 28 Apr 2010, *Lechat, C. CLL10048* (HAST 147481), largely immature; Maripasoula, Saül, trail up to Hmong village, 3.626053 N, 53.208075 W, 240–250 m, on dead blackened wood, 2 Apr 2021, *Fournier, J. GYJF21391* (HAST 147482); Maripasoula, Saül, trail head toward Roche-Bateau, 3.620498 N, 53.199309 W, disturbed secondary rainforest, on dead log, 22 Jun 2019, *Lechat, C. GYJF19215* (HAST 147483); Maripasoula, Saül, trail to Monts-La-Fumée, 3.634796 N, 53.205457 W, ca. 270 m, disturbed secondary rainforest, on dead blackened wood, 2 Apr 2021, *Fournier, J. GYJF21375* (HAST 147484); Maripasoula, Saül, shortcut to the airfield, 3.622852 N, 53.205768 W, 210–220 m, disturbed secondary rainforest, on dead blackened wood, 29 Mar 2021, *Fournier, J. GYJF21255* (HAST 147485); Maripasoula, Saül, trail to Monts-La-Fumée, 3.634796 N, 53.205457 W, ca. 270 m, disturbed secondary rainforest, on dead blackened wood, 2 Apr 2021, *Fournier, J. GYJF21378* (HAST 147486).

**Known distribution**: French Guiana.

**Comments**: Based on its narrowly clavate stromata overlain with conspicuous cream-colored scales, *X. scutulata* inevitably recalls young *X. curta* in the field. A closer examination shows that it differs by soft-textured stromata strongly shrinking upon drying due to a thinner leathery crust, smaller ostioles lacking a raised-discoid base, and slightly darker, smaller ascospores with more broadly rounded ends. This set of differential characters, consistently observed in the twelve cited collections of *X. scutulata*, supports its segregation from *X*. *curta*, which is in agreement with our phylogenetic analyses. Phylogenetically, *X. scutulata* appears more closely related to *X. orichalcea* than to *X. curta*, in agreement with the morphological comparison discussed under *X. orichalcea* in this paper.

San Martín and Rogers (1989) described and illustrated a collection of *X*. cf. *curta* from México similar in outline to *X. scutulata*. They did not document in detail the crust thickness nor the ostiolar morphology but recorded slightly larger ascospore dimensions of (9.5–)10–11 × 4–4.5 µm with little overlap. The status of this material remains uncertain.

**Fig. 65:**
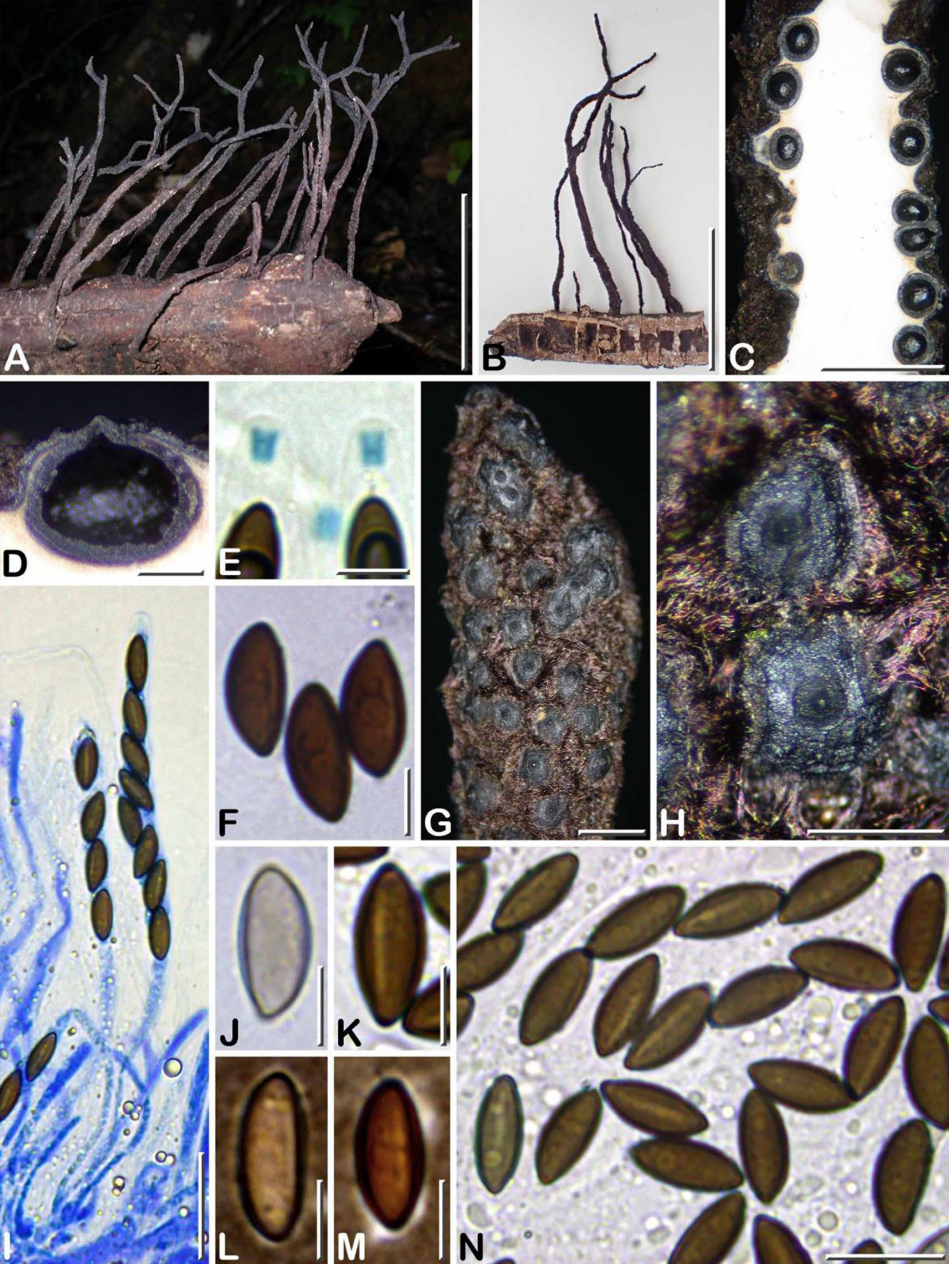
*Xylaria siliquarum* (**A ***GYJG* 21157; **B–N ***GYJF18165* holotype). **A** Habit of stromata arising from a pod, in situ. **B** Stromata arising from a split pod. **C** Stroma in longitudinal section showing perithecia immersed beneath a thin crust and a white solid interior. **D** Half-exposed perithecium in vertical section showing a papillate ostiole. **E** Ascal apical apparati, in Melzer’s reagent. **F** Mature ascospores after 15 min incubation in aqueous nigrosin, showing the absence of bipolar appendages. **G, H** Stromatal surface showing black slightly exposed perithecial contours emerging from a dense purplish brown tomentum. **I** Four- and eight-spored mature asci, in blue Pelikan ink. **J** Immature ascospore after 15 min incubation in aqueous nigrosin, lacking appendages. **K** Ascospore in ventral view showing a short germ slit, in 1% SDS. **L, M** Respectively immature and mature ascospores in India ink, showing reduced remnants of a mucilaginous coating on their wall. **N** Ascospores in 1% SDS, some in ventral view showing a germ slit. Bars in **A, B** = 50 mm; **C, G** = 1 mm; **D** = 0.2 mm; **E, F, J–M** = 5 µm; **H** = 0.5 mm; **I** = 20 µm; **N** = 10 µm

***Xylaria siliquarum*** J. Fourn., Y.-M. Ju, H.-M. Hsieh & Lechat, sp. nov. Figure 65, Table 41

**Table 41:** Ascospore dimensions in two collections of *X. siliquarum*, showing a narrow range of intraspecific variation, compared with those reported in literature for *X. ianthinovelutina*

Collection numbers	Ascospore measurements with extreme values in parentheses	Q = quotient l/w, *N* = number of measurements	Me = mean values of l/w, Qe = mean quotient l/w
*GYJF18165* holotype	(9.5–)9.9–10.8(−11.1) × (3.5–)4.0–4.4(−4.8) µm	Q = (2.0–)2.3–2.5(−2.8), *N* = 60	Me = 10.3 × 4.2 µm, Qe = 2.4
*GYJF 21157*	(9.2–)9.5–10.3(−10.5) × (3.4–)3.7–4.2(−4.3) µm	Q = (2.2–)2.4–2.7(−2.9), *N* = 60	Me = 9.9 × 3.9 µm, Qe = 2.5
**Cumulated values**	**(9.2–)9.5–10.8(−11.1) × (3.4–)3.7–4.4(−4.8) µm**	**Q = (2.0–)2.3–2.7(−2.9), N = 120**	**Me = 10.1 × 4.1 µm, Qe = 2.5**
*X. ianthinovelutina* (Ju et al. 2018)	(9–)9.5–11(−12) × (3.5–)4–4.5(−5) μm		10.3 ± 0.7 × 4.0 ± 0.3 μm, *N* = 60
*X. ianthinovelutina* (Fournier et al. 2020)	(7.5–)9–11.3(−12.6) × (3.4–)3.7–4.7(−5.1) µm	Q = (1.9–)2.1–2.8(−3.2), *N* = 480	Me = 10.1 × 4.2 µm, Qe = 2.4

MycoBank MB863187

**Typification**: **FRENCH GUIANA.** Maripasoula, Saül, way up to the “Belvédère de la Montagne Pelée”, 3.621515 N, 53.213768 W, ca. 240 m, disturbed secondary rainforest, on dead woody pod of presumably *Inga* (Fabaceae), 26 Aug 2018, *Courtecuisse, R. GYJF18165* (holotype HAST 147487).

**Etymology**: From Latin *siliqua, ae =* pod, referring to the substrate.

**Diagnosis**: Differs from *X. ianthinovelutina* by stouter stromata often apically branching at right angle and ascospores lacking bipolar appendages.

**Stromata** gregarious, separate, upright, arising in more or less linear rows along the axis of the pod, 30–110 mm in total height, subcylindrical in upper part, flattened below, simple to most often apically branched at right angle, straight to contorted, the fertile heads 18–42 mm high × (0.8–)1.3–2.6(−4) mm diam, with blunt or faintly apiculate sterile apices; surface brownish black, nodulose with perithecial contours strongly exposed emerging from dense tufts of dark brown to purplish brown tomentum, roughened by low, black, longitudinal crests or furrows; the stipes ill-defined, up to 80 mm high and up to 5 mm wide toward base, blackish, flattened, densely and entirely tomentose, the base slightly swollen; subsurface crust leathery, 30–40 µm thick; interior homogeneous, compact, white. **Perithecia** subglobose to slightly depressed-spherical, 0.35–0.50 mm diam. **Ostioles** papillate, dull black, on a low to slightly raised discoid base 120–200 µm diam.

**Paraphyses** hyphal, filiform, thin-walled, sparingly septate, weakly guttulate, 0.8–2 µm wide at apex, embedded in discreet mucilaginous material. **Asci** cylindrical, with (4–)8 slightly overlapping uniseriately arranged ascospores, the spore-bearing parts 64–72 × 5.5–6.0 µm, the stipes 40–50 µm long, with apical apparatus 1.9–2.4 × 1.5–1.8 µm (Me = 2.2 × 1.6 µm, *N* = 25), short-tubular with a faint upper rim, bluing in Melzer’s reagent. **Ascospores** (9.2–)9.5–10.8(−11.1) × (3.4–)3.7–4.4(−4.8) µm, Q = (2.0–)2.3–2.7(−2.9) *N* = 120 (Me = 10.1 × 4.1 µm, Qe = 2.5), ellipsoid slightly inequilateral with narrowly rounded to subacute ends, brown, unicellular, with a conspicuous, less than spore-length, longitudinally oriented germ slit 4.2–7 µm long on the ventral side, the shorter ones frequently displaced toward one end, with discreet remnants of a thin mucilaginous coating visible in India ink and lacking bipolar secondary appendages visible in water or stained by aqueous nigrosin; epispore smooth.

**Asexual morph** on the natural substrate not observed.

**Known distribution**: French Guiana, known from two collections.

**Other specimen examined (paratype)**: **FRENCH GUIANA.** Maripasoula, Saül, trail head to Sentier des Gros Arbres, 3.6201 N, 53.207989 W, ca. 210 m, disturbed secondary rainforest, on dead woody pod of presumably *Inga* (Fabaceae), 27 Mar 2021, *Fournier, J. GYJF21157* (HAST 147488).

**Comments**: *Xylaria siliquarum* is distinctive by its stout stromata, which frequently branch apically at right angle, are densely tomentose over the entire surface, and bear ostioles on a wide discoid base. It occurs on dead woody pods resembling those of the fabaceous genus *Inga*. It shares with *X. ianthinovelutina* a similarly densely tomentose stromatal surface and ascospores in the same size range (Table 41), but differs by ascospores lacking secondary appendages, even on immature ascospores, a character regarded as highly diagnostic in this group by Ju et al. (2018). Owing to the lack of appendages, it keys out to *X. apeibae* Mont. in Ju et al. (2018), a species also present at the same site and likewise allied to *X. ianthinovelutina*, but differing by its specificity for dead, sea urchin-like fruits of *Apeiba*, more slender stromata, and light brown ascospores (Ju et al. 2018; this paper).

The general habit of *X. siliquarum* also recalls that of *X. vivantii* Y.-M. Ju, J. D. Rogers, J. Fourn. & H.-M. Hsieh, known from Brazil, Guadeloupe and Martinique on fruits of *Magnolia* species, but that species is readily distinguished by significantly larger ascospores (14.5–)15–16.5(−17.5) × (4.5–)4.5–5.5(−6.0) μm with a long obliquely oriented germ slit and bipolar secondary appendages (Ju et al. 2018).

*Xylaria fabacearum* R. H. Perera, E. B. G. Jones & K. D. Hyde and *X. fabaceicola* R. H. Perera, E. B. G. Jones & K. D. Hyde, two species recently described from Thailand on woody fabaceous pods, also appear closely related to *X. siliquarum*. According to the protologues, *X*. *fabacearum* differs by lacking a tomentose stromatal surface and by having ascospores with a spore-length germ slit, whereas *X*. *fabaceicola* has smaller ascospores 7.5–10 × 3.4–4.8 µm (Me = 8.5 × 4.2 μm) bearing bipolar secondary appendages. These differential features were stated by the authors but not well illustrated (Perera et al. 2020). In their phylogenetic analyses, *X. fabaceicola* clusters with *X. ianthinovelutina*, while *X. fabacearum* appears only distantly related. *Xylaria aleuriticola* Hai X. Ma, A. H. Zhu & Yu Li, recently described from Yunnan (China) on fruits of *Aleurites moluccana* (Euphorbiaceae), was shown by ITS-RPB2–TUB2 analyses to be phylogenetically close to *X. fabaceicola* (Zhu et al. 2024). It differs from *X. siliquarum* mainly by smaller ascospores, averaging 8.1 × 3.6 μm, and by occurring on a distantly related host.

**Fig. 66:**
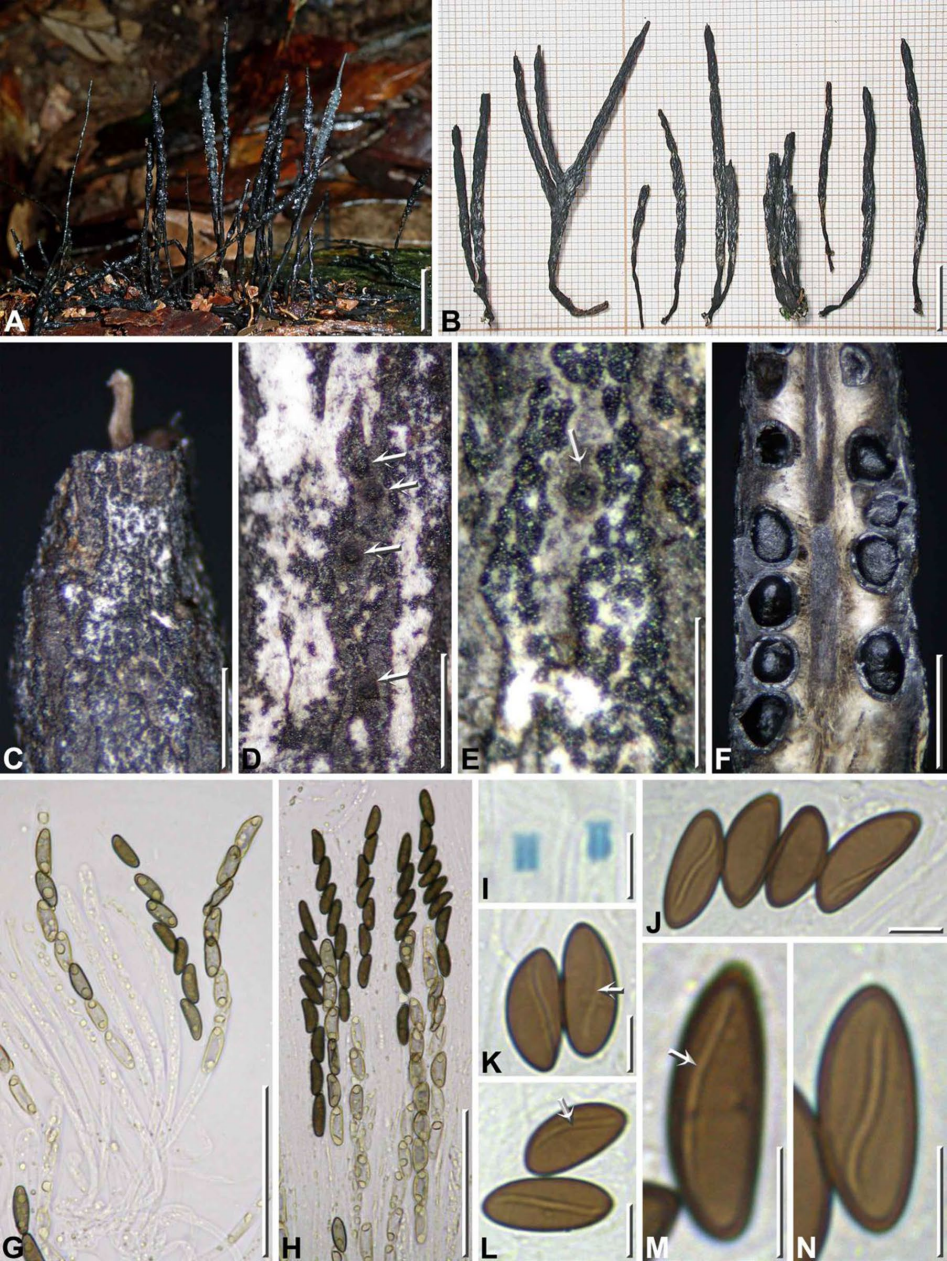
*Xylaria sinuosirima* (**A, C, E, I ***GYJF12173*; **B, D, F–H, J–N ***CLL8098* holotype). **A** Clustered stromata in situ. **B** Simple, rarely branched stromata. **C** Broken stromatal apex and granular surface. **D** Stromatal surface showing white superficial stripes partly associated with black granules and ostioles (arrows). **E** Stromatal surface showing white superficial stripes strongly associated with black granules and one ostiole (arrow) on a gray area lacking granules. **F** Stroma in longitudinal section showing a thick carbonaceous superficial crust and a whitish to gray fibrous interior with a blackish core. **G** Seven-spored immature asci, in 1% SDS. **H** Immature and mature asci, in 1% SDS. **I** Ascal apical apparati in Melzer’s reagent. **J–N** Ascospores showing a variously sinuous germ slit either on the ventral side or on the dorsal side (arrows). Bars in **A, B** = 10 mm; **C, D** = 0.5 mm; **E** = 0.25 mm; **F** = 1 mm; **G, H** = 50 µm; **I–N** = 5 µm

***Xylaria sinuosirima*** J. Fourn., Y.-M. Ju, H.-M. Hsieh & Lechat, sp. nov. Figure 66, Table 42

**Table 42:** Ascospore dimensions from the two known collections of *X. sinuosirima*

Collection numbers	Ascospore measurements with extreme values in parentheses	Q = quotient l/w, *N* = number of measurements	Me = mean values of l/w, Qe = mean quotient l/w
*CLL8098*	(9.5–)9.7–11.6(−12.7) × (4.1–)4.3–5.1(−5.4) µm	Q = (1.9–)2.0–2.6(−2.8), *N* = 60	Me = 10.6 × 4.6 µm, Qe = 2.3
*GYJF12173*	(8.4–)9.9–12.0(−15.3) × (3.4–)3.9–4.9(−5.3) µm	Q = (1.9–)2.1–2.8(−3.5), *N* = 60	Me = 10.9 × 4.5 µm, Qe = 2.5
**Cumulated values**	**(8.4–)9.7–12.0(−15.3) × (3.4–)3.9–5.1(−5.4) µm**	**Q = (1.9–)2.0–2.8(−3.5), N = 120**	**Me = 10.8 × 4.6 µm, Qe = 2.4**

MycoBank MB863188

**Typification**: **FRENCH GUIANA.** Macouria, vicinity of Cayenne, Risquetout Forest, trail to Saut-Léodate, 4.899372 N, 52.555061 W, ca. 55 m, lowland rainforest, on dead trunk, 6 May 2008, *Lechat, C. CLL8098* (holotype HAST 147490).

**Etymology**: From Latin *sinuosus =* sinuous, curved, and *rima =* slit, referring to the sigmoid ascospore germ slit.

**Diagnosis**: Differs from known species of *Xylaria* with needle-shaped stromata coated with white stripes by having ascospores averaging 10.8 × 4.6 µm that bear a variably sigmoid germ slit either on the ventral or the dorsal side.

**Stromata** gregarious, 17–48 mm in total height, the fertile parts 9–32 mm high × 1.8–2.0(−2.6) mm wide, narrowly cylindrical with faint circumferential constrictions, straight to slightly curved, simple to occasionally branched above the stipe or 2–3 arising from a common base, with apices sterile and mucronate, the tip readily broken off; surface with unexposed perithecial contours, roughened by a pure white outer layer splitting into large longitudinal stripes, first smooth, revealing dense clusters of shiny black carbonaceous granules as it peels off, on a long-persistent whitish background; surface dark gray between the stripes, lacking granular coating; outer crust 80–120(−150) µm thick, carbonaceous and brittle; interior solid, fibrous, whitish to gray, blackish beneath the outer crust, with a conspicuous black core; the stipes ill- to well-defined, 5–16 mm long × 0.8–1.6 mm, black, cylindrical to irregularly strap-like or slightly twisted, glabrous, smooth to sparsely granular, the base slightly enlarged. **Perithecia** fully immersed, subglobose, 0.40–0.55 µm diam. **Ostioles** often inconspicuous, papillate, blackish, ca. 80 µm diam.

**Paraphyses** hyphal, hyaline, remotely septate, simple, 4–5 µm wide at base, tapering to 1.0–1.5 µm wide, embedded in mucilaginous material. **Asci** cylindrical, with (6–7–)8 uniseriately arranged, slightly overlapping ascospores, the spore-bearing parts 66–82 µm long × 6.0–6.8 µm wide, the stipes 45–60 µm long, with apical apparatus tubular to slightly urn-shaped, with a faint upper rim, 2.2–2.6 × 1.4–1.8 µm (Me = 2.4 × 1.6 µm, *N* = 25), bluing in Melzer’s reagent. **Ascospores** (8.4–)9.7–12.0(−15.3) × (3.4–)3.9–5.1(−5.4) µm, Q = (1.9–)2.0–2.8(−3.5), *N* = 120 (Me = 10.8 × 4.6 µm, Qe = 2.4), ellipsoid-inequilateral with broadly to narrowly rounded ends, straight to suballantoid, medium brown, with a conspicuous sigmoid, occasionally diagonal to slightly sinuous germ slit less to slightly less than spore-length on the ventral or on the dorsal side, lacking sheath or appendage; epispore smooth.

**Asexual morph** on the natural substrate not observed.

**Other specimen examined (paratype)**: **FRENCH GUIANA.** Sinnamary, Paracou, CIRAD field centre, Guyaflux plot, 5.249218 N, 52.915431 W, lowland rainforest, on dead corticated trunk, 24 Jun. 2012, *Fournier, J. GYJF12173* (HAST 147489).

**Known distribution**: French Guiana, only known from two collections.

**Comments**: *Xylaria sinuosirima* can be distinguished from akin species with needle-shaped stromata, especially *X. pallidocylindracea* (Fournier et al. 2020; this paper), by its more strongly carbonaceous and brittle texture, black superficial granules that are at first concealed beneath white stripes and become exposed as the white stripes wear off, and a gray to blackish interior with a black core. Besides this combination of characteristics, *X. sinuosirima* is set apart by ascospores featuring a typically sigmoid germ slit on the ventral side, but fairly often also located on the dorsal side and obliquely oriented. Although somewhat variable, the germ slit morphology is characteristic and clearly supports the recognition of *X. sinuosirima* as a distinct species. Phylogenetically, *X*. *sinuosirima* belongs to a clade in which most species are characterized by a stromatal surface encrusted with granules, including *X*. *candelabroides*, *X*. *exechostoma*, *X*. *nigrosticta*, *X*. *oligotoma*, *X. pallidocylindracea*, *X*. *piloides*, and *X*. *venustula*.

**Fig. 67:**
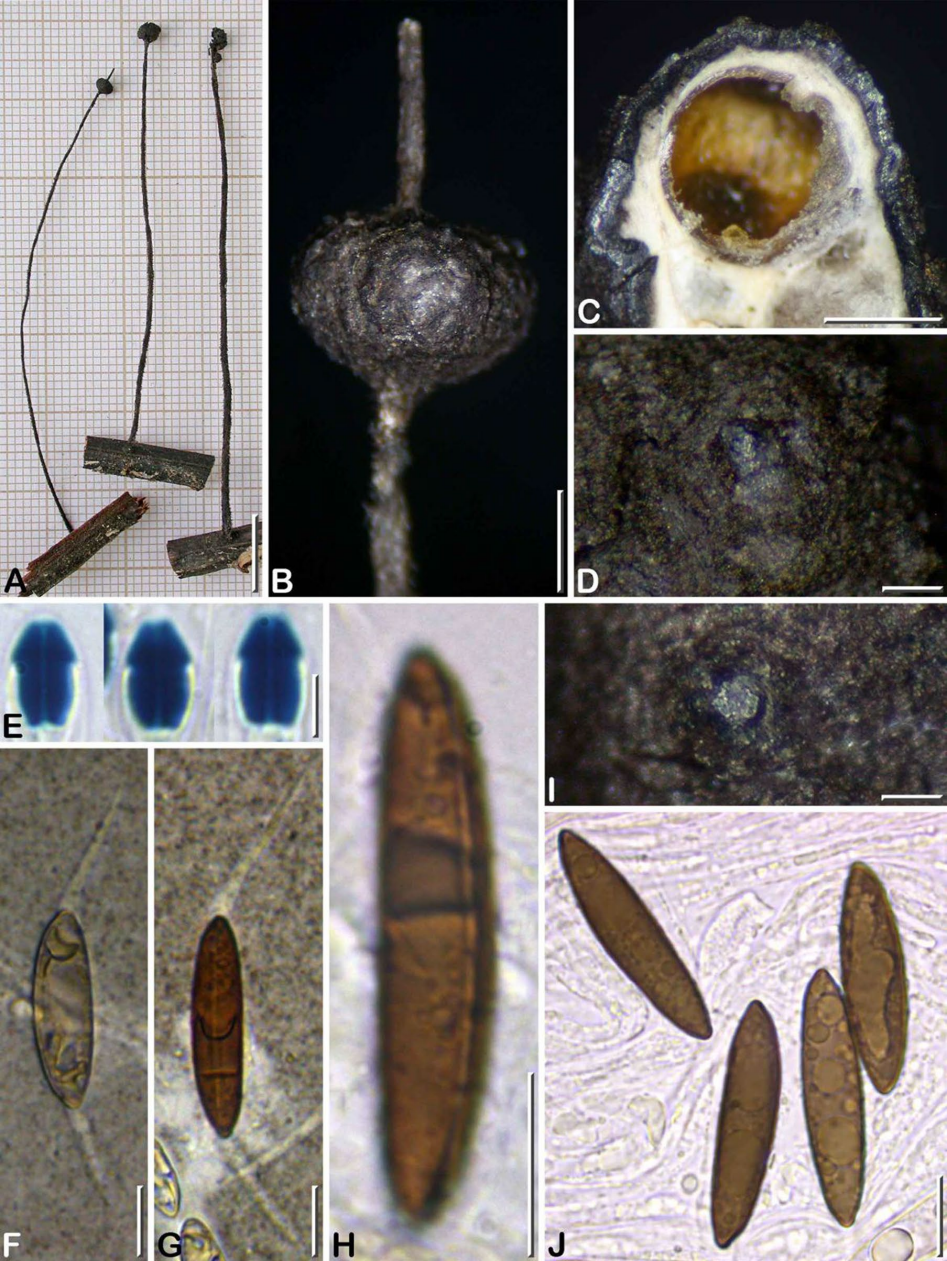
*Xylaria* sp. cf. *X. axifera* (*GYJF12067*). **A** Barely mature stromata (left) and mature stroma (right). **B** Fertile part of a barely mature stroma in side view. **C** Fertile part of a barely mature stroma in vertical section. **D, I** Ostioles in close-up. **E** Ascal apical apparati, in Melzer’s reagent. **F, G** Respectively immature and mature ascospores showing bipolar appendages, in India ink. **H** Ascospore showing a thin germ slit on the dorsal side, in 1% SDS. **J** Ascospores in 1% SDS. Bars in **A** = 10 mm; **B** = 1 mm; **C** = 0.5 mm; **D, I** = 100 µm; **E** = 5 µm; **F–H, J** = 10 µm

***Xylaria *sp.** cf. *X. axifera* Mont. Figure 67, Table 43

**Table 43:** Ascospore dimensions of *X. axifera* from literature compared with those of *GYJF12067*

Collection numbers	Ascospore measurements with extreme values in parentheses	Q = quotient l/w, *N* = number of measurements	Me = mean values of l/w, Qe = mean quotient l/w
*X. axifera* (Læssøe and Lodge 1994)	(19.1–)20.7–28.0(−31.0) × (5.6–)6.1–7.1(−7.5) µm		Me = 24.4 × 6.4 µm, Qe = 3.8
*X. axifera* (Ju and Hsieh 2023)	(21.5–)22.5–24.5(−26) × (6.5–)7–8(−9) μm	*N* = 40	23.6 ± 1.0 × 7.4 ± 0.6 μm, Qe = 3.2
*X. axifera GYJF21028* (this paper)	(19.2–)20.7–23.4(−24.2) × (5.2–)5.3–6.0(−6.2) µm	Q = (3.4–)3.6–4.3(−4.6), *N* = 50	Me = 22.0 × 5.6 µm, Qe = 3.9
*GYJF12067*	(28.8–)29.8–33.0(−34.9) × (6.1–)6.5–8.1(−8.7) µm	Q = (3.3–)3.9–4.7(−5.4), *N* = 60	Me = 31.3 × 7.3 µm, Qe = 4.3

**Stromata** upright, scattered, separate, 53–63 mm in total height, featuring a globose to subglobose fertile head 2.2–2.7 mm diam borne on a filiform stipe 50–60 mm high × 0.4–0.9 mm diam, topped by a short, fragile filiform extension 1.7 mm high; fertile head few-peritheciate, with perithecial contours unexposed; surface black, glabrous, obscurely cracked and scaly, lacking a peeling outer layer; subsurface a brittle, slightly carbonaceous crust 80–90 mm thick; interior loosely fibrous, whitish; the stipes black, arising from a slightly swollen base, hairy over the entire length, more densely so toward base. **Perithecia** subglobose, 0.7–0.8 mm diam. **Ostioles** conic-papillate, occasionally apically truncate and shiny black, 120–170 µm diam at base.

**Paraphyses** not seen. **Asci** fragmentary and not measured, with apical apparatus 7.9–8.8 × 4.7–5.4 µm (Me = 8.5 × 5.1 µm, *N* = 25), acorn-shaped with doliform lower part and conical upper part with sharp lateral rim, strongly bluing in Melzer’s reagent.

**Ascospores** (28.8–)29.8–33.0(−34.9) × (6.1–)6.5–8.1(−8.7) µm, Q = (3.3–)3.9–4.7(−5.4), *N* = 60 (Me = 31.3 × 7.3 µm, Qe = 4.3), narrowly fusiform, straight, slightly inequilateral with narrowly rounded to subacute, occasionally slightly pinched ends, medium brown, with a thin straight germ slit nearly spore-length on the dorsal side; with bipolar filiform, slightly falciform, easily detached secondary appendages 15–26 µm long and no mucilaginous sheath; epispore smooth.

**Asexual morph** on the natural substrate not observed.

**Specimen examined: FRENCH GUIANA.** Régina, Nouragues natural reserve, Inselberg camp, trail 11, 4.311240 N, 52.132789 W, ca. 300 m, hygrophilic rainforest, on dead leaf petioles 2–4 mm diam in the leaf litter, 19 Jun 2012, *Fournier, J. GYJF12067* (HAST 147217), largely immature.

**Known distribution**: French Guiana

**Comments**: By the combination of occurrence on dead leaf petioles, a subglobose fertile head lacking exposed perithecial contours and topped by a filiform extension, and narrowly fusiform ascospores with a straight dorsal germ slit and bipolar filiform appendages, this *Xylaria* species approaches *X. axifera*. However, it differs by lacking the reddish-brown outer layer and waxy olivaceous coating, and by having much longer stipes and longer ascospores with shorter filiform appendages, and we therefore refrain from equating it with *X. axifera*. The latter is well documented in the literature, with stromata that are morphologically fairly uniform but with ascospore dimensions that are rather variable, although never attaining those of the present collection and consistently exhibiting a lower length/width ratio (Table 43). The scantiness and largely immature condition of this collection, together with the absence of cultures and phylogenetic data, prevent us from establishing it as a distinct species at this time, pending further collections.

**Fig. 68:**
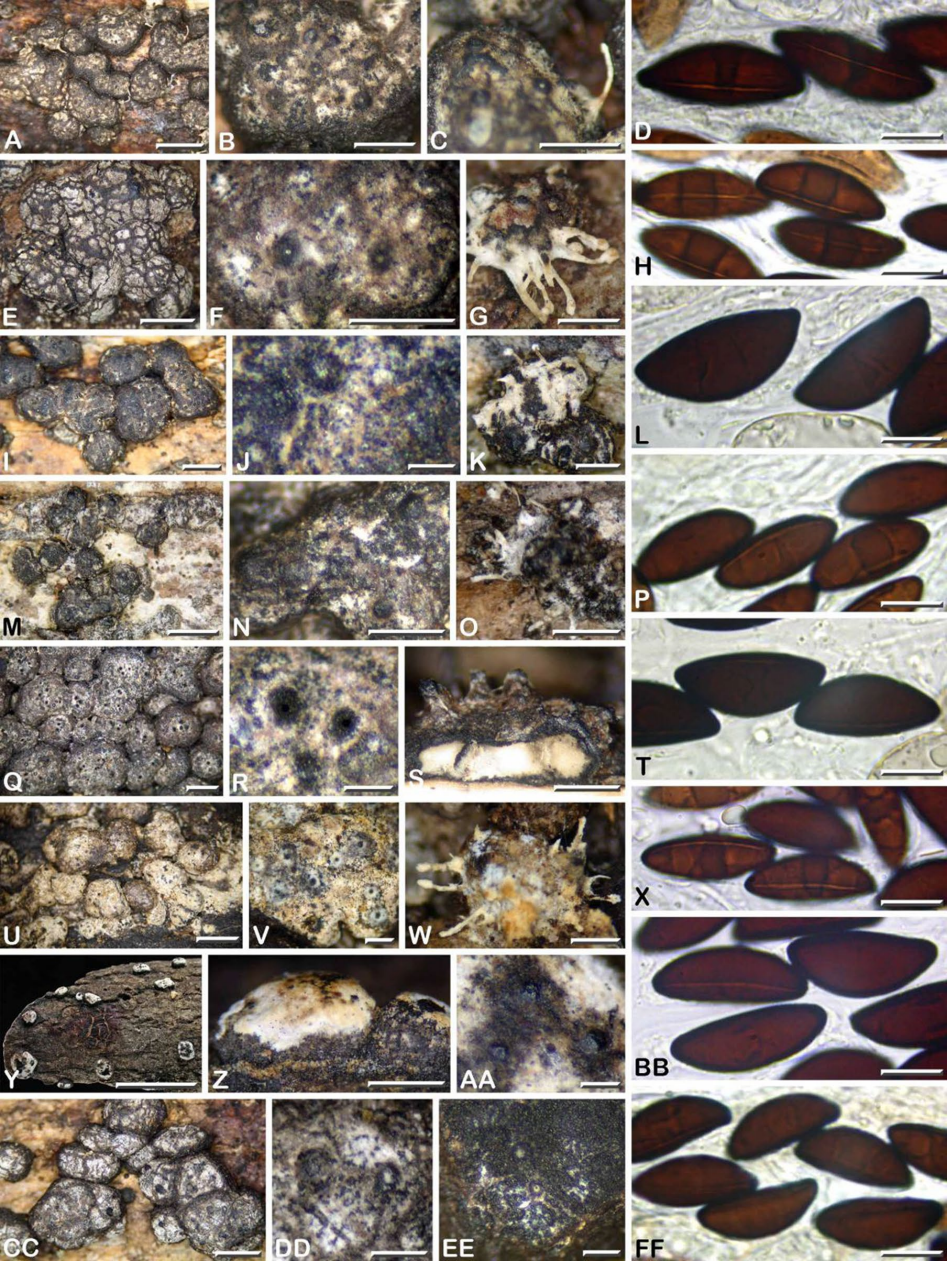
Synoptic comparative plate gathering a collection of *X. cantareirensis* from Brazil and eight related collections provisionally referred to *Xylaria* sp. cf. *X. cantareirensis* (**A–D** *Xylaria cantareirensis Rogerson 78.259*; **E–H ***CLL9055*; **I–L ***CLL7103*; **M–P ***CLL7103-2*; **Q–T ***GYJF21131*; **U–X ***MJF10052*; **Y–BB ***CLL0863*; **CC, DD, FF ***CLL8275*; **EE ***MJF14028*). **A, E, I, M, Q, U, Y, CC** Variously distributed and coalescent stromata on host surface. **B, F, J, N, R, V, Z, AA, DD, EE** Stromatal surface in close-up showing a white to cream-colored coating, ostioles and superficial black granules. **C, G, K, O, S, W** Sterile, rarely fertile synnemata or outgrowths, mostly on developing stromata. **D, H, L, P, T, X, BB, FF** Ascospores in 1% SDS, some showing a germ slit. Bars in **A, E, U** = 2 mm; **B, C, F, G, K, N, O, V, W** = 0.5 mm; **D, H, L, P, T, X, BB, FF** = 10 µm; **I, Y** = 10 mm; **J** = 100 µm; **M, Q, S, Z, CC** = 1 mm; **R, AA, DD, EE** = 0.2 mm

***Xylaria *sp.** cf. *X. cantareirensis* (Henn.) J. Fourn. & Lechat. Figure 68, Table 44

**Table 44:** Ascospore dimensions in *X. cantareirensis* from Brazil and nine related collections from French Guiana and Martinique referable to *Xylaria* sp. cf. *X. cantareirensis*

Collection numbers	Ascospore measurements with extreme values in parentheses	Q = quotient l/w, *N* = number of measurements	Me = mean values of l/w, Qe = mean quotient l/w
*Rogerson 78.259* Brazil	(25.1–)26.5–29.7(−30.6) × (8.9–)9.4–10.9(−11.7) µm	Q = (2.4–)2.6–3(−3.2), *N* = 60	Me = 28.1 × 10.1 µm, Qe = 2.8
*CLL9055* FG	(19.5–)19.9–22.2(−23.2) × (6.9–)7.5–8.7(−9.3) µm	Q = (2.3–)2.4–2.8(−3.1), *N* = 60	Me = 21.1 × 8.1 µm, Qe = 2.6
*CLL7103* FG	(21.9–)23.4–27.8(−30.0) × (7.5–)8.2–10.2(−11.2)	Q = (2.4–)2.5–3.1(−3.5), *N* = 120	Me = 25.7 × 9.1 µm, Qe = 2.8
*CLL7103-2* FG	(15.9–)17.2–20.4(−22.6) × (6.3–)7.2–8.7(−9.5) µm	Q = (2.0–)2.1–2.6(−3.1), *N* = 120	Me = 18.8 × 8.0 µm, Qe = 2.4
*GYJF21131* FG	(22.1–)23.0–26.9(−28.4) × (8.2–)8.8–10.3(−10.9) µm	Q = (2.2–)2.3–2.9(−3.1), *N* = 60	Me = 24.9 × 9.6 µm, Qe = 2.6
*MJF10052* Martinique	(18.3–)19.9–23–8(−25.9) × (7.0–)7.4–9.0(−9.6) µm	Q = (2.2–)2.4–3.1(−3.4), *N* = 180	Me = 22.2 × 8.2 µm, Qe = 2.7
*CLL0863* Martinique	(20.8–)21.7–25.2(−26.6) × (7.8–)8.4–10.0(−10.8) µm	Q = (2.2–)2.3–2.9(−3.1), *N* = 120	Me = 23.6 × 9.2 µm, Qe = 2.6
*CLL8275* Martinique	(19.0–)19.5–21.8(−22.9) × (7.0–)7.7–9.2(−9.8) µm)	Q = (2.0–)2.2–2.7(−3.0), *N* = 60	Me = 20.7 × 8.4 µm, Qe = 2.5
*MJF14028* Martinique	(16.5–)19.6–22.5(−24.4) × (7.3–)7.9–9.4(−10.6) µm	Q = (1.9–)2.2–2.7(−3.0), *N* = 60	Me = 20.9 × 8.6 µm, Qe = 2.5
*CLL2212* Martinique	(18.5–)19.4–21.7(−23.0) × (7.2–)7.6–8.9(−9.3) µm	Q = (2.2–)2.3–2.7(−2.9), *N* = 60	Me = 20.5 × 8.2 µm, Qe = 2.5

*Xylaria cantareirensis* is a penzigioid taxon described from Brazil, characterized by small, few-peritheciate, pulvinate, sessile, gregarious and often coalescent stromata with unexposed perithecial contours. The stromata bear a blackish, weakly carbonaceous crust coated by a cream-colored to white superficial layer that gradually cracks into polygonal or ill-defined scales and carries scattered filiform to clavate sterile outgrowths. As the superficial layer wears off, the underlying blackish crust becomes brownish gray and appears encrusted with persistent, scattered or clustered black carbonaceous granules. Ostioles are discoid to papillate and weakly prominent. Asci are eight-spored, with a cylindrical apical apparatus that strongly blues in Melzer’s reagent and is topped by a prominent upper rim. Ascospores are fairly large, averaging 28.1 × 10.1 µm, dark, brown, ellipsoid-inequilateral with subacute ends, lacking appendages, and possessing a ventral germ slit almost spore-length (this paper).

The nine collections related to *X. cantareirensis* that we studied (Table 44) correspond more or less to this concept in stromatal morphology, differing mainly in the density of black superficial granules and occasionally in the shape and dimensions of the ostioles. While some of the collections have filiform to clavate white outgrowths, *GYJF21131* has spiny to truncate ones, and no such outgrowths are present in four collections from Martinique: *CLL0863*, *CLL8275*, *MJF14028*, and *CLL2212*. Their ascospores suggest notable differences in their dimensions, shape, and germ slit position, which is occasionally dorsal in some of them; however, these differences proved difficult to correlate with stromatal features. Collection *CLL7103-2*, which has the smallest ascospores of the group, was found intermixed with *CLL7103*, which differs by larger multiperitheciate stromata and significantly larger ascospores (Table 44). Collections *CLL8275*, *CLL2212*, and *MJF14028* share smaller ascospores averaging 20.5 × 8.4 µm (Table 44), a more heavily granular stromatal surface, and occurrence in Martinique, suggesting that they may represent a different taxonomic entity. However, cultures and molecular data from typical *X*. *cantareirensis* are not available, preventing us from resolving its relationships with the studied collections.

This unresolved situation recalls the results presented by Fournier et al. (2018c), which were based solely on morphology and lacked Guianese representatives. The most rewarding phylogenetic result concerns collection *CLL5437*, which was accepted as *X. cantareirensis* in Fournier et al. (2018c) based on ascospore dimensions but proved to be markedly distant from the group discussed above. It is therefore described herein as *X. guadalupensis*.

**Specimens examined: FRENCH GUIANA.** Macouria, vicinity of Cayenne, Risquetout Forest, trail to Saut-Léodate, 4.899372 N, 52.555061 W, ca. 55 m, lowland rainforest, on dead corticated woody liana 8–15 mm diam, 1 May 2007, *Lechat, C. CLL7103-2* (HAST 147221); Macouria, vicinity of Cayenne, Risquetout Forest, trail to Saut-Léodate, 4.899372 N, 52.555061 W, ca. 55 m, lowland rainforest, on dead corticated liana 8–15 mm diam, 1 May 2007, *Lechat, C. CLL7103* (HAST 147222); Maripasoula, Saül, trail head to Sentier des Gros Arbres, 3.620839 N, 53.208536 W, ca. 210 m, disturbed secondary rainforest, on dead decorticated branch, 26 Mar 2021, *Fournier, J. GYJF21131* (HAST 147223); Régina, vicinity of airstrip, 4.316743 N, 52.131763 W, disturbed marshy rainforest, on dead corticated branchlet, 22 Apr 2009, *Lechat, C. CLL9055* (HAST 147224). **FRENCH WEST INDIES.** *MJF10052*, *MJF14028*, *CLL0863*, *CLL2212*, and *CLL8275*, see Fournier et al. (2018c) for collecting data.

**Fig. 69:**
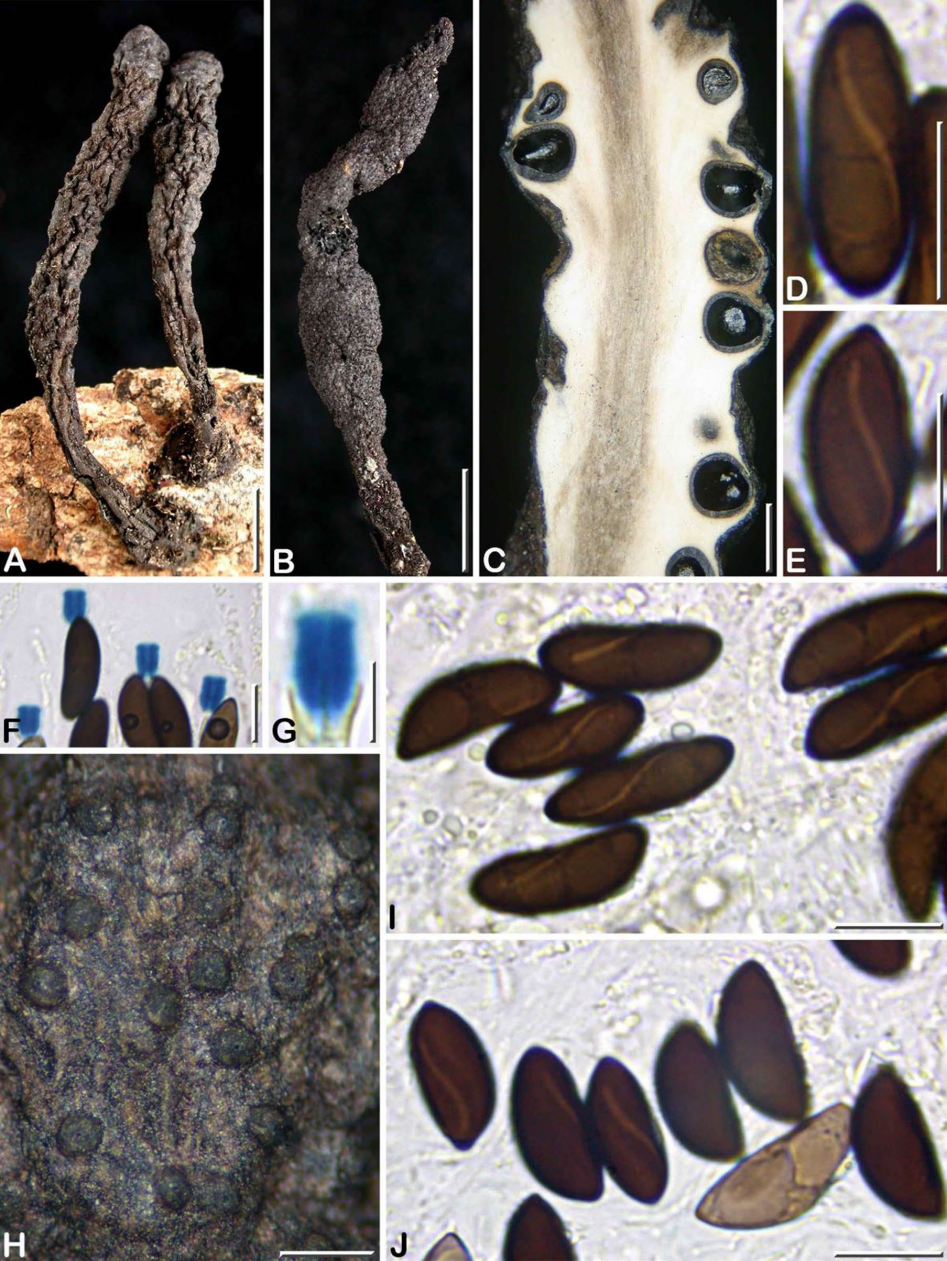
*Xylaria* species complex cf. *X. scruposa* (**A ***GYJF18113*; **B, E, H, J ***GYJF18083*; **C, D, I ***GYJF18122*; **F, G ***GYJF18104-1*). **A, B** Stromata in the dry state. **C** Stroma in vertical section. **D, E** Ascospores in ventral view showing a germ slit, in 1% SDS. **F, G** Ascal apical apparati, in Melzer’s reagent. **H** Stromatal surface in close-up showing brown appressed scales and raised-discoid ostioles. **I, J** Ascospores in 1% SDS, some showing a germ slit. Bars in **A, B** = 5 mm; **C** = 1 mm; **D–F**, **I, J** = 10 µm; **G** = 5 µm; **H** = 0.5 mm

***Xylaria*** **species complex** cf. *X. scruposa* (Fr.) Fr. Figure 69, Table 45

**Table 45:** Ascospore dimensions in nine collections of small-spored collections of *X. scruposa* from FG, compared with those of presumably typical collections from the French West Indies and French Guiana, respectively

Collection numbers	Ascospore measurements with extreme values in parentheses	Q = quotient l/w, *N* = number of measurements	Me = mean values of l/w, Qe = mean quotient l/w
*CLL5088*	(13.7–)14.4–16.2(−17.1) × (5.5–)5.8–6.5(−6.8) μm	Q = (2.2–)2.3–2.7(−2.9), *N* = 60	Me = 15.4 × 6.1 μm, Qe = 2.5
*CLL5567*	(12.7–)13.7–15.8(−17.4) × (5.3–)5.6–6.3(−6.5) μm	Q = (2.2–)2.3–2.8(−2.9), *N* = 60	Me = 14.7 × 6.0 μm, Qe = 2.5
*MJF13362*	(13.5–)14.1–15.9(−17.0) × (5.0–)5.4–6.2(−6.5) μm	Q = (2.3–)2.4–2.9(−3.1), *N* = 60	Me = 15.1 × 5.7 μm, Qe = 2.6
*GYJF21274*	(13.1–)13.5–15.3(−16.0) × (5.2–)5.4–6.2(−6.5) µm	Q = (2.1–)2.2–2.8(−3.0), *N* = 60	Me = 14.3 × 5.8 µm, Qe = 2.5
*GYJF21272* (taxon I-1)	(16.9–)18.3–20.9(−22.1) × (5.9–)6.4–7.3(−7.8) µm	Q = (2.5–)2.6–3.3(−3.6), *N* = 60	Me = 19.6 × 6.8 µm, Qe = 2.9
*GYJF18083* (taxon I-2)	(13.3–)13.7–15.6(−17.7) × (4.9–)5.5–6.5(−6.8) µm	Q = (2.0–)2.3–2.8(−3.2), *N* = 60	Me = 14.9 × 6.0 µm, Qe = 2.5
*GYJF21067* (taxon II-1)	(12.1–)13.6–16.2(−17.5) × (4.8–)5.3–6.3(−6.7) µm	Q = (2.1–)2.3–3.0(−3.4), *N* = 60	Me = 14.8 × 5.7 µm, Qe = 2.6
*GYJF21285* (taxon II-1)	(13.0–)13.8–16.1(−17.2) × (5.4–)5.6–6.2(−6.7) µm	Q = (2.2–)2.3–2.8(−3.0), *N* = 60	Me = 14.8 × 5.9 µm, Qe = 2.5
*GYJF18104-1* (taxon II-2)	(12.8–)14.2–16.0(−17.0) × (5.2–) 5.5–6.2 (−6.6) µm	Q = (2.1–)2.3–2.8(−3.0), *N* = 60	Me = 15.0 × 5.9 µm, Qe = 2.5
*GYJF18133* (taxon II-3)	(13.4–)13.9–16.0(−18.7) × (5.1–)5.7–6.5(−6.7) µm	Q = (2.1–)2.2–2.8(−3.0), *N* = 60	Me = 14.9 × 6.1 µm, Qe = 2.4
*GYJF18113* (taxon II-3)	(15.6–)16.2–19.0(−20.4) × (5.8–)6.1–6.8(−7.1) µm	Q = (2.3–)2.5–2.9(−3.2) *N* = 60	Me = 17.6 × 6.5 µm Qe = 2.7
*GYJF18122* (taxon II-3)	(15.0–)15.8–18.4(−19.4) × (5.6–)5.8–6.6(−7.1) µm	Q = (2.3–)2.5–3.0(−3.3) *N* = 60	Me = 17.0 × 6.3 µm Qe = 2.7
**Cumulated values**	**(12.1–)13.5–16.2(−18.7) × (4.8–)5.3–6.5(−6.8) μm**	**Q = (2.2–)2.3–3.0(−3.4), N = 540**	**Me = 14.9 × 5.9 µm, Qe = 2.5**
*X. scruposa* FWI (Fournier et al. 2020)	(14.2–)15.7–24.1(−28.1) × (4.9–)5.4–8.1(−9.1) μm	Q = (2.3–)2.5–3.6(−4.5), *N* = 720	Me = 19.4 × 6.5 μm, Qe = 3

The five taxa closely related to *X*. *scruposa* shown in the phylogram fall within the broad range of stromatal morphological variation known for this species, but, except for *X*. sp. I-1, they share consistently smaller ascospores, possibly correlated with a slightly longer germ slit. This pattern was already observed during the survey of this species in the French West Indies, and it is striking to encounter the same situation in our numerous collections from French Guiana.

We initially hypothesized that a small-spored taxon within *X. scruposa* might be revealed by phylogenetic data by assessing the degree of sequence divergence between typical and small-spored material. This seems to be supported among the Guianese collections but indicates the presence of more than one taxon. These collections are temporarily separated into five taxa, which group into two distinct subclades: *X*. sp. I-1 and *X*. sp. I-2 are sister to the kretzschmarioid *X*. *mamillata* and the others, whereas *X*. sp. II-1, *X*. sp. II-2, and *X*. sp. II-3, form another subclade. Several collections with intermediate ascospore dimensions add further ambiguity, leaving this group unresolved at the current stage.

**Specimen examined for X. sp. I-1**: **FRENCH GUIANA.** Maripasoula, Saül, trail head to Sentier Roche-Bateau, 3.62056 N, 53.199899 W, ca. 240 m, disturbed secondary rainforest, on dead blackened wood, 30 Mar 2021, *Fournier, J. GYJF21272* (HAST 147472).

**Specimen examined for X. sp. I-2**: **FRENCH GUIANA.** Maripasoula, Saül, trail head to Sentier des Gros Arbres, 3.620589 N, 53.208547 W, on dead trunk, 22 Aug 2018, *Fournier, J. GYJF18083* (HAST 147471).

**Specimens examined for X. sp. II-1**: **FRENCH GUIANA.** Maripasoula, Saül, trail head to Sentier Roche-Bateau, 3.62056 N, 53.199899 W, ca. 240 m, disturbed secondary rainforest, on dead wood, 30 Mar 2021, *Fournier, J. GYJF21285* (HAST 147473); Roura, Cacao, Camp Bonaventure, 4.326815 N, 52.341431 W, 60–65 m, secondary hygrophilic rainforest, on dead corticated branch, 22 Mar 2021, *Fournier, J. GYJF21067* (HAST 147474).

**Specimen examined for X. sp. II-2**: **FRENCH GUIANA.** Maripasoula, Saül, trail head to Roche-Bateau, 3.619046 N, 53.200658 W, on big dead corticated liana on the ground, 23 Aug 2018, *Fournier, J. GYJF18104-1* (HAST 147468).

**Specimens examined for X. sp. II-3**: **FRENCH GUIANA.** Maripasoula, Saül, trail head to Sentier des Gros Arbres, 3.620564 N, 53.208511 W, on dead wood, 24 Aug 2018, *Fournier, J. GYJF18113* (HAST 147467); Maripasoula, Saül, trail head to Sentier des Gros Arbres, Crique Grand-Fossé, 3.617197 N, 53.209042 W, on dead corticated branch, 24 Aug 2018, *Fournier, J. GYJF18133* (HAST 147469); Maripasoula, Saül, trail head to Sentier des Gros Arbres, 3.620564 N, 53.208511 W, on dead wood, 24 Aug 2018, *Gardiennet, A. GYJF18122* (HAST 147470).

**Fig. 70:**
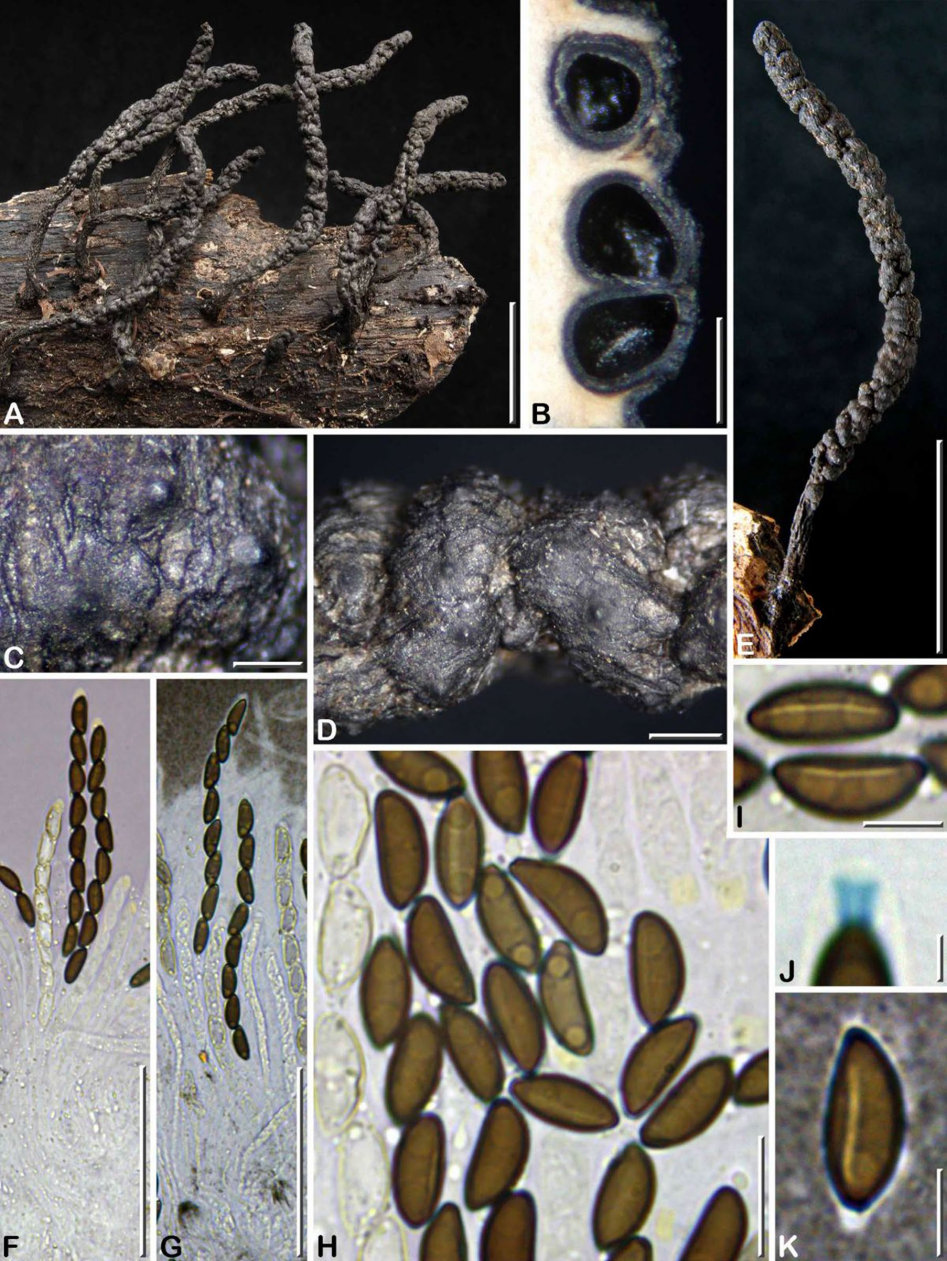
*Xylaria spegazziniana* (*GYJF19027* holotype). **A, E** Stromata in the dry state showing a nodulose surface. **B** Stromatal surface in vertical section. **C, D** Stromatal surface in close-up showing deep constrictions, ostioles and remnants of a striped silvery gray outer layer. **F, G** Asci, respectively in black Pelikan ink and India ink. **H** Ascospores in 1% SDS, some showing a germ slit. **I** Two ascospores showing a germ slit, either on dorsal or on ventral side. **J** Ascal apical apparatus in Melzer’s reagent. **K** Ascospore in ventral view showing a germ slit and a thin mucilaginous coating, in India ink. Bars in **A, E** = 10 mm; **B, D** = 0.5 mm; **C** = 0.2 mm; **F, G** = 50 µm; **H** = 10 µm; **I, K** = 5 µm; **J** = 2 µm

***Xylaria spegazziniana*** J. Fourn., Y.-M. Ju, H.-M. Hsieh & Lechat, sp. nov. Figure 70, Table 46

**Table 46:** Ascospore dimensions in five collections of *X. spegazziniana* from French Guiana, showing the range of intraspecific variations, compared with those reported in literature

Collection numbers	Ascospore measurements with extreme values in parentheses	Q = quotient l/w, *N* = number of measurements	Me = mean values of l/w, Qe = mean quotient l/w
*GYJF19027* holotype	(8.6–)9.1–10.5(−11.4) × (3.5–)3.8–4.3(−4.6) µm	Q = (1.9–)2.2–2.7(−2.9), *N* = 60	Me = 9.7 × 4.0 µm, Qe = 2.4
*GYJF19089*	(8.3–)8.8–10.2(−10.6) × (2.7–)3.6–4.2(−4.4) µm	Q = (2.0–)2.2–2.8(−3.5), *N* = 60	Me = 9.4 × 3.8 µm, Qe = 2.5
*GYJF21065*	(7.9–)8.2–9.4(−10.7) × (2.7–)3.3–3.9(−4.2) µm	Q = (2.0–)2.2–2.7(−3.0), *N* = 60	Me = 8.8 × 3.6 µm, Qe = 2.5
*GYJF21361*	(8.4–)9.1–10.6(−11.3) × (3.5–)3.7–4.4(−4.5) µm	Q = (2.1–)2.2–2.7(−2.9), *N* = 60	Me = 9.7 × 4.0 µm, Qe = 2.4
*GYJF21399*	(8.1–)8.4–9.9(−10.5) × (3.2–)3.5–4.0(−4.7) µm	Q = (1.8–)2.3–2.7(−2.9), *N* = 60	Me = 9.1 × 3.7 µm, Qe = 2.5
**Cumulated values**	**(7.9–)8.2–10.6(−11.4) × (2.7–)3.3–4.4(−4.7) µm**	**Q = (1.8–)2.2–2.8(−3.5), N = 300**	**Me = 9.3 × 3.8 µm, Qe = 2.45**
(Spegazzini 1884)	8–9 × 3–3.5 µm		Me = 8.5 × 3.3 µm
(Dennis 1956)	6–9 × 3–4 µm		Me = 7.5 × 3.5 µm
FWI (Fournier et al. 2020)	(8.1–)8.9–10.8(−11.8) × (3.3–)3.5–4.5(−4.8) µm	Q = (1.9–)2.2–2.9(−3), *N* = 480	Me = 9.7 × 3.9 µm, Qe = 2.4

MycoBank MB863189

**Typification**: **FRENCH GUIANA.** Maripasoula, Saül, shortcut to the airfield, 3.623524 N, 53.206542 W, 210–220 m, disturbed secondary rainforest, on dead blackened wood, 17 Jun 2019, *Fournier, J. GYJF19027* (holotype HAST 147493).

**Etymology**: In honor of the renowned Italian-Argentinian mycologist Carolo Spegazzini (1858–1926) who first recognized this species as undescribed.

**Diagnosis**: Differs from similar species with narrowly cylindrical, leathery stromata by a deeply constricted surface with a fleeting, striped, silvery gray outer layer and ascospores averaging 9.3 × 3.8 µm, with narrowly rounded ends and a germ slit either on the ventral or on the dorsal side.

**Stromata** scattered or in small or large groups, separate, occasionally connate at base by 2–3, mostly narrowly cylindrical to subclavate, simple, terete to slightly flattened, straight to strongly curved, with broadly rounded fertile apices or apiculate sterile apices, subsessile to long-stipitate, 7–32 mm in total height, the fertile head 5–22 mm high × 1.5–4.2 mm diam, the stipe to 1.8–12 mm high × 0.8–1.5 mm diam; surface blackish gray before maturity due to a thin, striped, silvery gray superficial pellicle, eventually black at maturity, finely roughened, usually strongly nodulose with irregularly arranged, at times circumferential deep wrinkles and furrows, with perithecial contours barely to strongly exposed; the stipes ill- to sharply-defined, black, puckered, terete to flattened, glabrous, swollen and slightly tomentose at the very base; subsurface crust leathery, 35–50 µm thick; interior solid, spongy, white to yellowish. **Perithecia** subglobose 0.5–0.7 mm diam, laterally flattened when crowded. **Ostioles** finely conic-papillate, faintly prominent, shiny black at maturity, 80–120 µm diam at base.

**Paraphyses** sparse, hyphal, filiform, thin-walled, 4.0–5.0 µm wide at base, 1.0–1.5 µm wide at apex, embedded in mucilaginous material. **Asci** cylindrical, with (6–)8 slightly overlapping uniseriately arranged ascospores, the spore-bearing parts 72–79 × 4.5–5.5 µm, the stipes 55–90 µm long, with apical apparatus 1.8–2.3 × 1.3–1.7 µm (Me = 2.1 × 1.5 µm *N* = 50), short-cylindrical with a faint upper rim, bluing in Melzer’s reagent. **Ascospores** (7.9–)8.2–10.6(−11.4) × (2.7–)3.3–4.4(−4.7) µm, Q = (1.8–)2.2–2.8(−3.5), *N* = 300 (Me = 9.3 × 3.8 µm, Qe = 2.45), ellipsoid-inequilateral with narrowly, less frequently broadly rounded ends, medium brown, unicellular, with a conspicuous longitudinally oriented germ slit slightly less than spore-length on the ventral side, occasionally to frequently on the dorsal side or shorter or misplaced; a thin mucilaginous coating is often present just after release from the ascus but does not constitute a well-defined mucilaginous sheath or appendages; epispore smooth.

**Asexual morph** on the natural substrate not observed.

**Other specimens examined (paratypes)**: **FRENCH GUIANA.** Maripasoula, Saül, trail head to Sentier des Gros Arbres, 3.620839 N, 53.208536 W, ca. 210 m, disturbed secondary rainforest, on dead blackened wood, 3 Apr 2021, *Fournier, J. GYJF21399* (HAST 147491); Maripasoula, Saül, trail head to Sentier des Gros Arbres, 3.620324 N, 53.207951 W, ca. 210 m, disturbed secondary rainforest, on dead blackened wood, 18 Jun 2019, *Fournier, J. GYJF19089* (HAST 147492); Maripasoula, Saül, trail head to Sentier des Gros Arbres, Crique Grand-Fossé, 3.617198 N, 53.209029 W, ca. 210 m, disturbed secondary rainforest, on dead blackened wood, 1 Apr. 2021, *Fournier, J. GYJF21361* (HAST 147494); Roura: Cacao, Camp Bonaventure, 4.326815 N, 52.341431 W, 60–65 m, disturbed hygrophilic rainforest, on dead corticated branch, 22 Mar 2021, *Fournier, J. GYJF21065* (HAST 147495).

**Known distribution**: Neotropical: Argentina (Spegazzini 1884), Guyana, Jamaica, Paraguay (Dennis 1956), French Guiana (this paper) and Martinique (Fournier et al. 2020).

**Comments**: *Xylaria spegazziniana* was documented by Fournier et al. (2020) as *X. multiplex* var. *microsperma* (Speg.) Dennis, based on abundant material collected in Martinique and on examination by YMJ of the specimen illustrated by Dennis (1956) for this taxon [**GUYANA.** Essequibo River, Moraballi Creek, near Bartica, on dead log, 23 Oct 1929, *Martyn, E. B. R660*, as *X. biceps* var. *microsperma* (K(M) 169679)]. That name was accepted only with reservations by Fournier et al. (2020), because the material shows strong morphological divergence from typical *X. multiplex* (Kunze) Fr. and from *X. biceps* Speg., from which var. *microsperma* was segregated to accommodate this small-ascospored taxon. As discussed by Fournier et al. (2020), *X. biceps* was synonymized with *X. arbuscula* Sacc. by Dennis (1956) who preferred to treat *X. biceps* var. *microsperma* as a small-ascospored variety of *X. multiplex*.

The type material of *X. biceps* var. *microsperma* was not located in Spegazzini’s herbarium (LPS), but isotypes were later studied in European herbaria [**PARAGUAY.** Guarapi, on wood, 19 Jul 1881, *Balansa, B.*, *Pl. du Paraguay 2772* (isotypes of *X. biceps* var. *microsperma* K(M) 169680, PAD, PC)]. Ironically, these isotypes have ascospores in the same size range as *X*. *multiplex* (Dennis 1956), and thus do not represent a “small-ascospored” variety. Since the present fungus cannot be assigned to any known name, we thus propose the new name *X. spegazziniana*.

Our phylogenetic analyses clearly show that this taxon is unambiguously distant from both *X. arbuscula* and X. *multiplex*, confirming closer affinities with *X. curta* Fr., as suspected from phenotypic data, and the closest relationship with *X. minor* (this paper). *Xylaria minor* can be distinguished from *X*. *spegazziniana* by consistently smaller stromata less than 12 mm high, hairy-tomentose stipes, smaller perithecia, and slightly smaller ascospores with a germ slit consistently on the ventral side. In the absence of phylogenetic support, these differences might have been regarded as too slight to justify the recognition of a separate species.

*Xylaria spegazziniana* is mainly characterized by narrowly cylindrical, leathery-textured stromata with a mostly rounded fertile apex and a strongly nodulose surface with a fleeting silvery gray superficial outer layer that splits into narrow stripes. Its ascospores are remarkable in that the germ slit is fairly frequently located on the dorsal side. *Xylaria spegazziniana* appears to be more widespread in the French West Indies than in French Guiana, which accounts for the slight differences between the description given here, based on five collections, and that provided by Fournier et al. (2020), which was based on 49 collections.

**Fig. 71:**
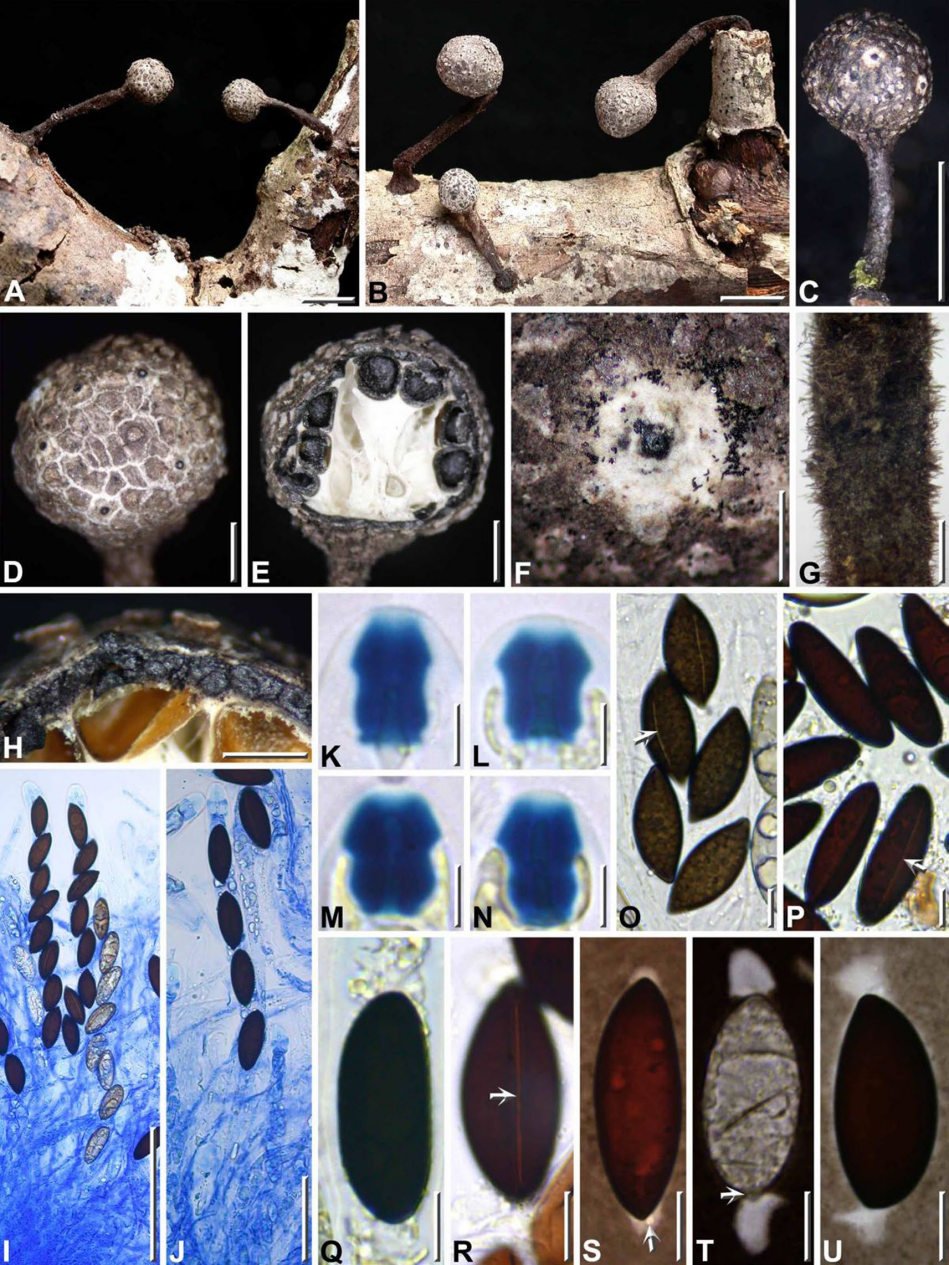
*Xylaria squamulosa* (**A, B, D–H, M, N, R, S ***CLL7077*; **C, Q ***GYJF19028*; **I–L, O, T, U ***GYJF21031*; **P ***CLL9080*). **A** Barely mature stromata with intact stipes. **B** Barely mature stromata with partly broken stipes showing a discoid base. **C** Mature stroma in side view. **D** Fertile head of a barely mature stroma showing a cracked surface with pale brown scales and ostioles. **E** Fertile head of a mature stroma in vertical section. **F** Close-up on ostiolar area of a mature stroma, showing ejected ascospores at periphery. **G** Close-up on hairy stipe surface. **H** Immature stroma in vertical section showing a thick carbonaceous crust. **I** Eight-spored asci. **J** Four spored ascus (**I** and **J** in blue Pelikan ink). **K–N** Ascal apical apparati, in Melzer’s reagent. **O, P** Variously shaped ascospores, some showing a narrow germ slit (arrows), in 1% SDS. **Q** Obtusely ended ascospore, in Melzer’s reagent. **R** Ascospore in dorsal view showing a germ slit (arrow) in 1% SDS. **S–U** Ascospores showing refractive primary appendages (arrows) and secondary appendages, in India ink. Bars in **A–C** = 5 mm; **D, E** = 1 mm; **F, G, H** = 0.5 mm; **I** = 100 µm; **J** = 50 µm; **K–N** = 5 µm; **O–U** = 10 µm

***Xylaria squamulosa*** San Martín & J. D. Rogers, Mycotaxon 34: 368. 1989. Figure 71, Table 47

**Table 47:** Ascospore and ascal apical apparatus dimensions in four collections of *X. squamulosa* showing their variation range, compared with those reported from the protologue

Collection numbers	Ascospore measurements with extreme values in parentheses	Q = quotient l/w, *N* = number of measurements	Me = mean values of l/w, Qe = mean quotient l/w	Ascal apical apparatus h × w, *N* = 20
*CLL7077*	(27.8–)29.6–33.5(−34.4) × (10.7–)11.4–13.2(−13.8) µm	Q = (2.3–)2.4–2.8(−2.9), *N* = 60	Me = 31.1 × 12.2 µm, Qe = 2.5	8.8–10.1 × 6.6–7.7 µm, Me = 9.6 × 7.2 µm
*CLL9080*	(32.6–)35.6–43.0(−45.9) × (11.1–)11.9–14.3(−17.0) µm	Q = (2.5–)2.7–3.3(−3.7), *N* = 60	Me = 39.1 × 13.2 µm, Qe = 3.0	
*GYJF19028*	(28.1–)30.0–35.6(−36.9) × (11.6–)12.1–14.1(−14.9) µm	Q = (2.1–)2.3–2.8(−3.0), *N* = 60	Me = 32.9 × 13.0 µm, Qe = 2.5	
*GYJF21031*	(27.0–)28.0–31.4(−32.7) × (10.4–)11.2–13.4(−14.2) µm	Q = (2.1–)2.2–2.6(−2.8), *N* = 60	Me = 29.7 × 12.4 µm, Qe = 2.4	9.2–11.2 × 7.0–7.8 µm, Me = 10.3 × 7.4 µm
**Cumulated values**	**(27.0–)28.0–43.0(−45.9) × (10.4–)11.2–14.3(−17.0) µm**	**Q = (2.1–)2.2–3.3(−3.7), N = 240**	**Me = 33.2 × 12.7 µm, Qe = 2.6**	**8.8–11.2 × 6.6–7.8 µm, Me = 10.0 × 7.3 µm**
Mexico (San Martín and Rogers 1989)	(29–)31–37(−40) × (11.6–)12–13(−14) µm		Me = 34 × 12.5 µm, Qe = 2.7	11–13 × 6–7 µm, Me = 12 × 6.5 µm

**Stromata** scattered on host surface, upright to prostrate, rarely connate at base by two, (5–)10–15 mm in total height, distinctly stipitate, the fertile parts subglobose 2.5–4.5 mm diam, apically widely rounded and fertile, with unexposed perithecial contours, not splitting upon drying; the stipes narrowly cylindrical to filiform, 2.5–10 mm high, 0.7–1.3 mm wide, well-defined, with a distinctly discoid base, slightly carbonaceous and brittle, black, densely hairy before full maturity, hairs pale brown, darkening and loosening with age. Surface brownish gray before full maturity, with a fairly thick superficial layer reticulately cracked into light brown polygonal scales on a white background, wearing off with age and leaving thin, appressed, gray to whitish scales on a blackish background; stromatal crust just beneath surface black, carbonaceous, 120–170 µm thick; interior white, solid, radially fibrous, becoming partly lacunose upon drying. **Perithecia** subglobose to slightly depressed-spherical, 0.75–1.3 mm diam, fully immersed. **Ostioles** discoid-annulate, barely prominent, 120–170 µm diam, black, located at the center of a gray to white polygonal area 0.3–0.5 mm wide.

**Paraphyses** copious, hyphal, hyaline, remotely septate, simple, 8.0–12.5 µm wide at base, tapering to 1.5–2.0 µm wide, embedded in mucilaginous material. **Asci** fragmentary in most collections, cylindrical, with (4–6–)8 uniseriately arranged, slightly overlapping ascospores, the spore-bearing parts (140–)188–235 µm long × 17–23 µm wide, the stipes 90–140 µm long, with apical apparatus fairly variable, more or less strongly constricted at upper third, apically cupulate and trapezoid with sharp lateral rims, with concave to convex sides in lower part, the base flattened, 8.8–11.2 × 6.6–7.8 µm (Me = 10.0 × 7.3 µm, *N* = 40), strongly bluing in Melzer’s reagent. **Ascospores** (27.0–)28.0–43.0(−45.9) × (10.4–)11.2–14.3(−17.0) µm, Q = (2.1–)2.2–3.3(−3.7), *N* = 240 (Me = 33.2 × 12.7 µm, Qe = 2.6), highly variable in shape and dimensions, ranging from ellipsoid-inequilateral with narrowly rounded and pinched ends, occasionally almost citriform, to oblong with broadly rounded ends, ventrally convex, dark brown, with a narrow straight germ slit spore-length or nearly so on the dorsal side, longitudinally oriented, with a minute refractive primary appendage present at one end in some collections, coated with a small obtusely rounded, fairly persistent secondary appendage, that may be bipolar, more conspicuous and elongated to triangular in some collections, visible on freshly released ascospores, most often evanescent, best seen in India ink; epispore smooth.

**Asexual morph** on the natural substrate not observed.

**Specimens examined**: **FRENCH GUIANA.** Maripasoula, Saül, shortcut to the airfield, 3.623524 N, 53.206542 W, 210–220 m, disturbed secondary rainforest, on dead corticated branch, 17 Jun 2019, *Lechat, C. GYJF19028* (HAST 147496); Macouria, Risquetout Forest, trail to Saut-Léodate, 4.899372 N, 52.555061 W, ca. 55 m, lowland rainforest, on dead wood, 25 Apr 2009, *Lechat, C. CLL9080* (HAST 147497); Roura, Cacao, Camp Bonaventure, 4.323472 N, 52.338502 W, 70 m, on corticated branch, 21 Mar 2021, *Fournier, J. GYJF21031* (HAST 147498); Sinnamary, Paracou, CIRAD field centre, Guyaflux plot, 5.249218 N, 52.915431 W, lowland rainforest, on dead wood, 27 Feb 2007, *Lechat, C. CLL7077* (HAST 147499).

**Known distribution**: First described from Mexico (San Martín and Rogers 1989) and reported herein for the first time from French Guiana; likely widespread in Amazonian rainforests if accepted in a wider sense (Læssøe 1999).

**Comments**: Our collections cited above correspond well to *X. squamulosa* as described by San Martín and Rogers (1989), in exhibiting small stromata with a globose, hard-textured, scaly fertile head borne on a narrow stipe and large ascospores averaging 33.2 × 12.7 µm, with a long, straight germ slit on the dorsal side. However, our collections show a wide range of variation in ascospore shape and size (Table 47), as well as in the presence and shape of secondary appendages, suggesting the possibility of cryptic species that would require wider sampling and further investigation. This is the view taken by Læssøe (1999) who preferred to group small *X. comosa*-like collections with larger ascospores under *X*. aff. *comosa* rather than using the name *X. squamulosa*.

We follow the concept of *X. squamulosa* of San Martín and Rogers (1989) for our collections, which consistently have a small, rounded fertile head without conidial processes, borne on a short filiform stipe, and broader ascospores than *X. comosa*. However, we recognize that the taxonomy of small stromatal species deviating from typical *X. comosa* remains unclear.

**Fig. 72:**
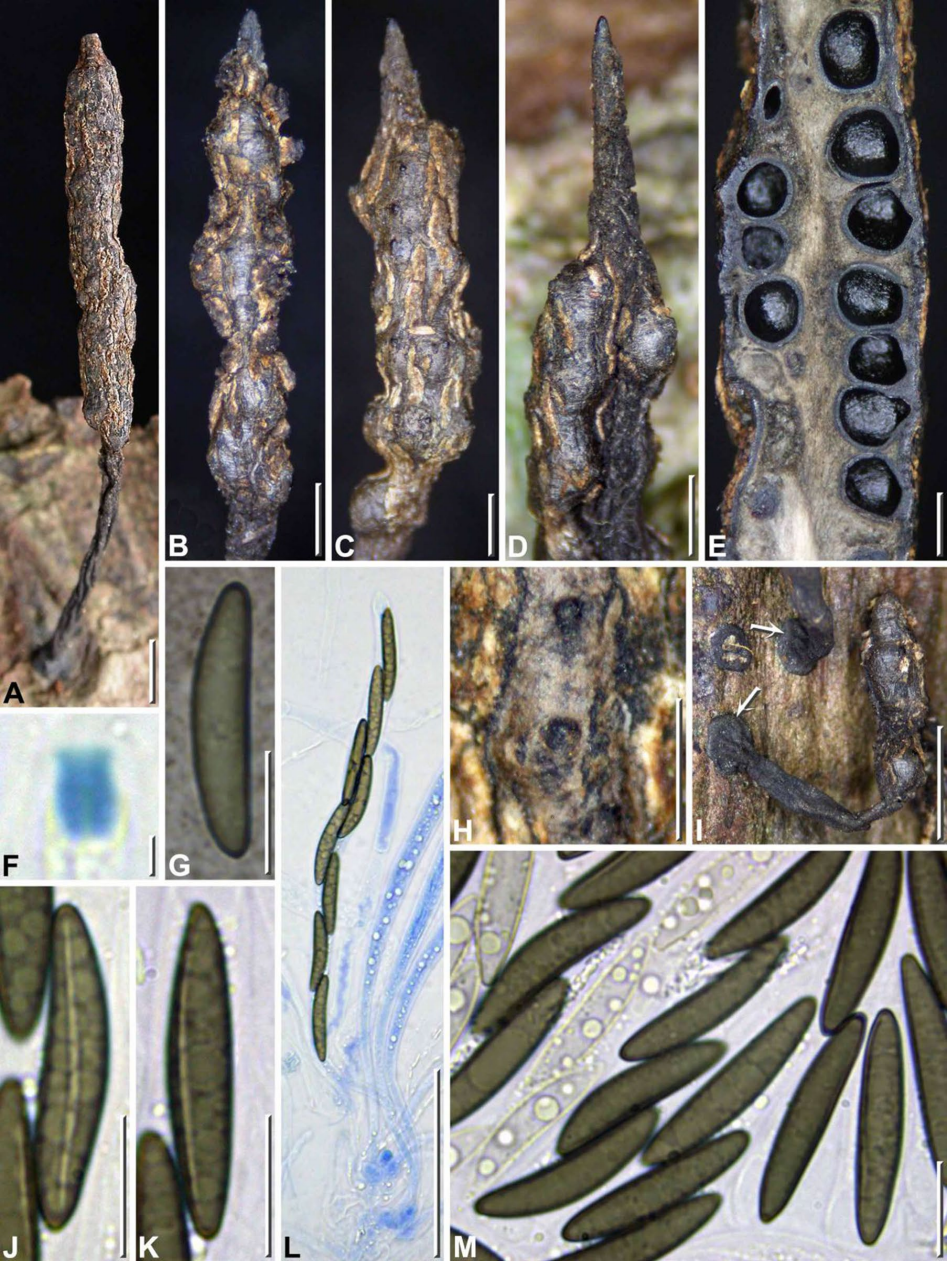
*Xylaria stupenda* (*GYJF21045* holotype). **A** Weakly nodulose stroma with broken apiculus. **B–D** Nodulose, sharply apiculate fertile heads showing a thick, yellowish to tan splitting outer layer. **E** Stroma in longitudinal section showing a gray to dark gray interior. **F** Ascal apical apparatus in Melzer’s reagent. **G** Ascospore in India showing absence of sheath or appendages. **H** Stromatal surface in close-up showing two slightly prominent ostioles. **I** Prostrate stroma with a curved stipe originating from a wide discoid base (arrows). **J, K** Ascospores showing a germ slit respectively on the ventral and the dorsal side. **L** Mature ascus, in blue Pelikan ink. **M** Variously oriented ascospores, in 1% SDS. Bars in **A, I** = 2 mm; **B–D** = 1 mm; **E, H** = 0.5 mm; **F** = 2 µm; **G, J, K, M** = 10 µm; **L** = 50 µm

***Xylaria stupenda*** J. Fourn., Y.-M. Ju, H.-M. Hsieh & Lechat, sp. nov. Figure 72

MycoBank MB863190

**Typification**: **FRENCH GUIANA.** Roura, Cacao, Camp Bonaventure, 4.323472 N, 52.338502 W, ca. 70 m, hygrophilic secondary rainforest, on dead corticated fallen trunk, 21 Mar 2021, *Fournier, J. GYJF21045* (holotype HAST 147500).

**Etymology:** from Latin *stupendus* = stupendous, amazing, referring to the striking disproportion between the fairly large, narrowly fusiform, curved ascospores and the minute, nodulose stromata.

**Diagnosis**: Differs from known *Xylaria* species featuring minute, nodulose, apiculate stromata by narrowly fusiform, medium brown, curved ascospores 21.9–24.7 × 4.3–5.0 µm.

**Stromata** upright to prostrate, discrete, isolated or in loose clusters, 8–19 mm in total height, the fertile head 3.0–13 mm high, 1.3–2.0 mm diam, cylindrical, almost terete to most often nodulose with strongly exposed perithecial contours, with a sharply mucronate to apiculate sterile apex; outer layer thick, tan, splitting into irregular elongated stripes; subsurface crust gray to blackish, finely roughened, leathery, 40–50 µm thick; interior loosely fibrous, light gray, darker gray beneath surface and around perithecia; the stipes sharply delimited, often strap-like and twisted, black, smooth, glabrous or finely hairy in places, 2.5–6.5 mm high, 0.4–0.9 mm diam, originating from a conspicuous black, discoid, glabrous base. **Perithecia** subglobose, 0.40–0.55 mm diam. **Ostioles** black, finely conic-papillate, 100–120 µm diam at base, often inconspicuous.

**Paraphyses** hyphal, thin-walled, remotely septate, 6–7.5 µm diam at base, tapering to 1.5–2.0 µm above asci, embedded in mucilaginous material. **Asci** cylindrical to narrowly fusiform, with (6–)8 slightly overlapping uniseriately arranged ascospores, the spore-bearing parts 120–130 × 8–10 µm, the stipes 50–70 µm long, with apical apparatus 2.6–3.2 × 1.7–2.4 µm (Me = 3.0 × 2.1 µm, *N* = 25), tubular to slightly urn-shaped, with a faint upper rim, bluing in Melzer’s reagent. **Ascospores** (20.9–)21.9–24.7(−25.6) × (3.8–)4.3–5.0(−5.2) µm, Q = (4.4–)4.7–5.5(−5.9), *N* = 60 (Me = 23.3 × 4.6 µm, Qe = 5.1), narrowly fusiform-inequilateral with narrowly rounded ends, most often slightly curved, ventrally concave, medium olivaceous brown in the fresh state, with a conspicuous germ slit almost spore-length, straight to slightly sinuous, most often on the ventral side, occasionally on the dorsal side; no sheath or appendages detected in India ink; epispore smooth.

**Asexual morph** on the natural substrate not observed.

**Known distribution**: French Guiana, known only from the holotype.

**Comments**: The minute, discrete stromata of *X*. *stupenda* were first mistaken in the field for a reduced form of *X. coccophora* because of their black, nodulose surface with a thick yellowish splitting outer layer. The ascospores are clearly different from those of *X. coccophora* in dimensions, color, and shape, strongly supporting the recognition of a distinct species.

*Xylaria stupenda* is strikingly similar, in stromatal and ascospore morphology, to *X. tenuispora* (Dennis) D. Hawksw., described from dead pods of *Macrolobium dewevrei* from Congo by Dennis (1961) [as *Xylosphaera*]. Besides its occurrence on dead fabaceous pods in a geographically distant region, *X*. *tenuispora* differs by a gray outer layer splitting longitudinally and significantly larger and more slender ascospores 36–42 × 4–5 µm.

Our phylogenetic data suggest a close affinity with *X. atractoidosperma*, from which *X*. *stupenda* mainly differs by significantly smaller stromata and much larger, differently shaped ascospores.

**Fig. 73:**
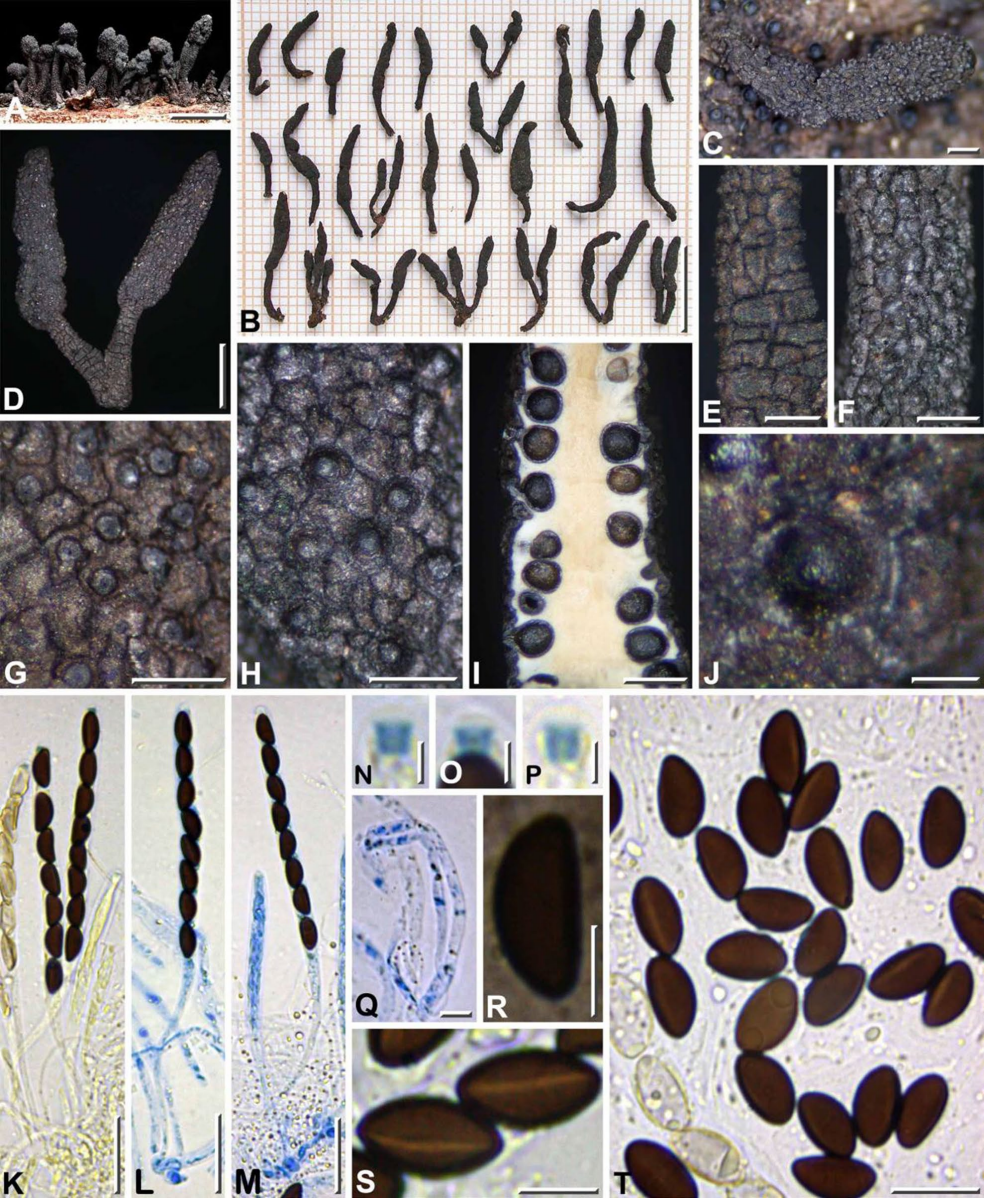
*Xylaria subplebeja* (**A, H, K–O, Q, R, T ***GYJF21117* holotype; **B, D, E, G, I, J, P, S ***CLL7061*; **C, F ***GYJF21046*). **A** Habit of upright stromata in side view arising from bark. **B** Variously shaped stromata from the same collection. **C** Clavate stroma on bark. **D** Two stromata connate at base. **E, F** Cracked stipe surface in close-up. **G, H** Close-up on stromatal surface roughened by small brownish to blackish scales surrounding black ostioles. **I** Stroma in longitudinal section. **J** Close-up on a prominent conical ostiole in top view. **K** Mature asci in Melzer’s reagent. **L, M** Mature asci and paraphyses in blue Pelikan ink. **N–P** Ascal apical apparati in Melzer’s reagent. **Q** Paraphyses in blue Pelikan ink. **R** Ascospore in side view lacking a sheath or appendages, in India ink. **S** Ascospores in ventral view showing a germ slit, in Melzer’s reagent. **T** Mature ascospores, some showing a ventral germ slit, in 1% SDS. Bars in **A** = 5 mm; **B** = 10 mm; **C** = 1 mm; **D** = 2 mm; **E–I** = 0.5 mm; **J** = 0.1 mm; **K–M** = 20 µm; **N–P** = 2 µm; **Q–S** = 5 µm; **T** = 10 µm

***Xylaria subplebeja*** J. Fourn., Y.-M. Ju, H.-M. Hsieh & Lechat, sp. nov. Figure 73, Table 48

**Table 48:** Ascospore dimensions in six collections of *X. subplebeja* showing a fairly wide variation range

Collection numbers	Ascospore measurements with extreme values in parentheses	Q = quotient l/w, *N* = number of measurements	Me = mean values of l/w, Qe = mean quotient l/w
*CLL7061*	(7.9–)8.3–9.2(−10.0) × (3.5–)3.8–4.5(−4.7) µm	Q = (1.8–)1.9–2.3(−2.6), *N* = 60	Me = 8.8 × 4.2 µm, Qe = 2.1
*GYJF19196*	(7.1–)8.0–9.2(−9.6) × (3.7–)4.0–4.5(−4.8) µm	Q = (1.7–)1.9–2.2(−2.3), *N* = 60	Me = 8.6 × 4.3 µm, Qe = 2.0
*GYJF21046*	(8.3–)8.9–10.2(−10.7) × (3.7–)4.0–4.5(−5.1) µm	Q = (1.7–)2.0–2.5(−2.6), *N* = 60	Me = 9.5 × 4.3 µm, Qe = 2.2
*GYJF21083*	(7.7–)8.0–9.2(−9.6) × (3.9–)4.0–4.6(−4.9) µm	Q = (1.7–)1.8–2.2(−2.3), *N* = 60	Me = 8.6 × 4.3 µm, Qe = 2.0
*GYJF21097*	(8.4–)8.5–9.6(−10.0) × (3.8–)4.0–4.8(−5.0) µm	Q = (1.8–)1.9–2.3(−2.5), *N* = 60	Me = 9.1 × 4.4 µm, Qe = 2.1
*GYJF21117* holotype	(7.8–)8.2–8.9(−9.3) × (3.9–)4.0–4.7(−4.8) µm	Q = (1.7–)1.8–2.1(−2.3), *N* = 60	Me = 8.6 × 4.4 µm, Qe = 2.0
**Cumulated values**	**(7.1–)8.0–10.2(−10.7) × (3.5)3.8–4.8(−5.1) µm**	**Q = (1.7–)1.8–2.5(−2.6) N = 360**	**Me = 8.9 × 4.3 µm, Qe = 2.1**

MycoBank MB863191

**Typification**: **FRENCH GUIANA.** Roura, Cacao, Camp Bonaventure, 4.330514 N, 52.338095 W, ca. 60 m, hygrophilic secondary rainforest, on dead corticated trunk, 24 Mar 2021, *Fournier, J. GYJF21117* (holotype HAST 147504).

**Etymology**: Referring to its similarity to *X. plebeja*.

**Diagnosis**: Differs from *X. plebeja* and *X. uncinata* by lacking a citrine-yellow layer around perithecia and from *X. rhytidophloea* by the coarsely cracked surface of the entire stipe.

**Stromata** upright to prostrate, gregarious, simple to frequently connate at base by 2–3, arising in linear rows or in bunches from black swellings erumpent from bark, (3.0–)6.0–18 mm in total height, the fertile head (2.0–)3.0–13 mm high × 1.0–3.0 mm diam, cylindrical to most often subfusiform, straight to curved, apically rounded and fertile or less frequently faintly mucronate and sterile, brittle, with perithecial contours unexposed; surface blackish to blackish brown, glabrous, densely corky-cracked into minute, thick polygonal warts and roughened by conspicuous ostioles; the stipes usually well-defined, 1.0–10.0 mm high × 0.8–1.3 mm diam, straight to slightly curved, terete to flattened, blackish, glabrous, with superficial cracks over the whole length delimiting large polygonal to rectangular plaques or small isodiametric scales or warts, slightly swollen at base; subsurface a black leathery crust 30–40 µm thick; interior solid, soft-textured, pure white, occasionally with a yellowish white core. **Perithecia** subglobose, 0.25–0.30(−0.40) mm diam, laterally flattened when in contact. **Ostioles** finely papillate on a broadly rounded, discoid to conical base, black, 120–170 µm diam, up to 150 µm high, more or less conspicuously prominent above the cracked superficial layer.

**Paraphyses** sparse, hyphal, remotely septate, thin-walled, 4.0–5.0 µm wide at base, tapering to 1.5–2 µm wide above asci, discreetly embedded in mucilaginous material. **Asci** cylindrical, with (6–)8 slightly overlapping uniseriately, occasionally biseriately arranged ascospores, the spore-bearing parts 57–74 × 5.0–6.5(−8.0) µm, the stipes 52–65 µm long, with apical apparatus quadrate in optical view, slightly wider than high, apically flared and flattened with a faint lateral rim, 1.3–1.7 × 1.5–1.8 µm (Me = 1.5 × 1.7 µm, *N* = 40), bluing in Melzer’s reagent. **Ascospores** (7.1–)8.0–10.2(−10.7) × (3.5–)3.8–4.8(−5.1) µm, Q = (1.7–)1.8–2.5(−2.6), *N* = 360 (Me = 8.9 × 4.3 µm, Qe = 2.1), ellipsoid-inequilateral with mostly broadly rounded ends, dark brown, unicellular, with a conspicuous, straight, longitudinally oriented germ slit almost spore-length on the ventral side; lacking appendages or mucilaginous sheath; epispore smooth.

**Asexual morph** on the natural substrate not observed.

**Other specimens examined (paratypes**): **FRENCH GUIANA.** Maripasoula, Saül, trail head to Sentier des Gros Arbres, 3.6201 N, 53.207989 W, ca. 210 m, disturbed mesophilic rainforest, on bark, 27 Mar 2021, *Fournier, J. GYJF21187* (HAST 147501); Roura, Cacao, Camp Bonaventure, 4.323472 N, 52.338502 W, ca. 70 m, hygrophilic secondary rainforest, on dead corticated trunk, 21 Mar 2021, *Fournier, J. GYJF21046* (HAST 147502); Sinnamary, Paracou, CIRAD field centre, Guyaflux plot, 5.249218 N, 52.915431 W, lowland rainforest, on dead corticated branch, 26 Feb 2007, *Lechat, C. CLL7061* (HAST 147503); Roura, Cacao, Camp Bonaventure, 4.328545 N, 52.339082 W, 60–65 m, on dead corticated trunk, 23 Mar 2021, *Fournier, J. GYJF21097* (HAST 147505); Roura, Cacao, Camp Bonaventure, 4.326815 N, 52.341431 W, 60–65 m, on dead corticated trunk, 22 Mar 2021, *Fournier, J. GYJF21083* (HAST 147506); Maripasoula, Saül, trail head to Sentier Roche-Bateau, 3.620498 N, 53.199309 W, ca. 230 m, disturbed secondary rainforest, on dead corticated branch, *Fournier, J. GYJF19196* (HAST 147507).

**Known distribution**: French Guiana.

**Comments**: *Xylaria subplebeja* is diagnosed by gregarious, small-sized, subfusiform, stipitate stromata less than 20 mm high, brittle, superficially roughened by warts and prominent ostioles, and frequently connate in groups of two to three at the base. The stipe is glabrous and entirely cracked into rectangular or polygonal scales. This latter character is shared by *X*. *palifera* (this paper), *X. plebeja* Ces., and *X. uncinata* (this paper), all of which differ from *X*. *subplebeja* by having the perithecia embedded in a citrine-yellow tissue layer.

The four species above, *X. crustulata* (this paper), and *X. martinicensis* J. Fourn. & Lechat and its var. *microspora* J. Fourn. & Lechat form a well-supported phylogenetic group, showing strong affinities to *X. heliscus* (Mont.) J. D. Rogers & Y.-M. Ju, *X. intracolorata* (J. D. Rogers, Callan & Samuels) J.D. Rogers & Y.-M. Ju, and *X. rhytidophloea* Mont. As all these species share similar ascospore morphology, their distinction relies mainly on subtle differences in stromatal shape, as well as stromatal surface features, ostiolar morphology, and stromatal interior characters.

***Xylaria telfairii*** (Berk.) Fr., Nov. Act. Reg. Soc. Sci. Upsal., ser. III, 1: 127. 1851.

≡ *Sphaeria telfairii* Berk., Ann. Nat. Hist., Mag. Zool. Bot. Geol. 3: 397. 1839.

The cited collection from French Guiana closely matches those reported and illustrated from the French West Indies (Fournier et al. 2019).

**Specimen examined: FRENCH GUIANA.** Sinnamary, Paracou, CIRAD field centre, Guyaflux plot, 5.249218 N, 52.915431 W, lowland rainforest, on dead corticated branch, 23 Jun 2012, *Fournier, J. GYJF 12149* (JF).

**Fig. 74:**
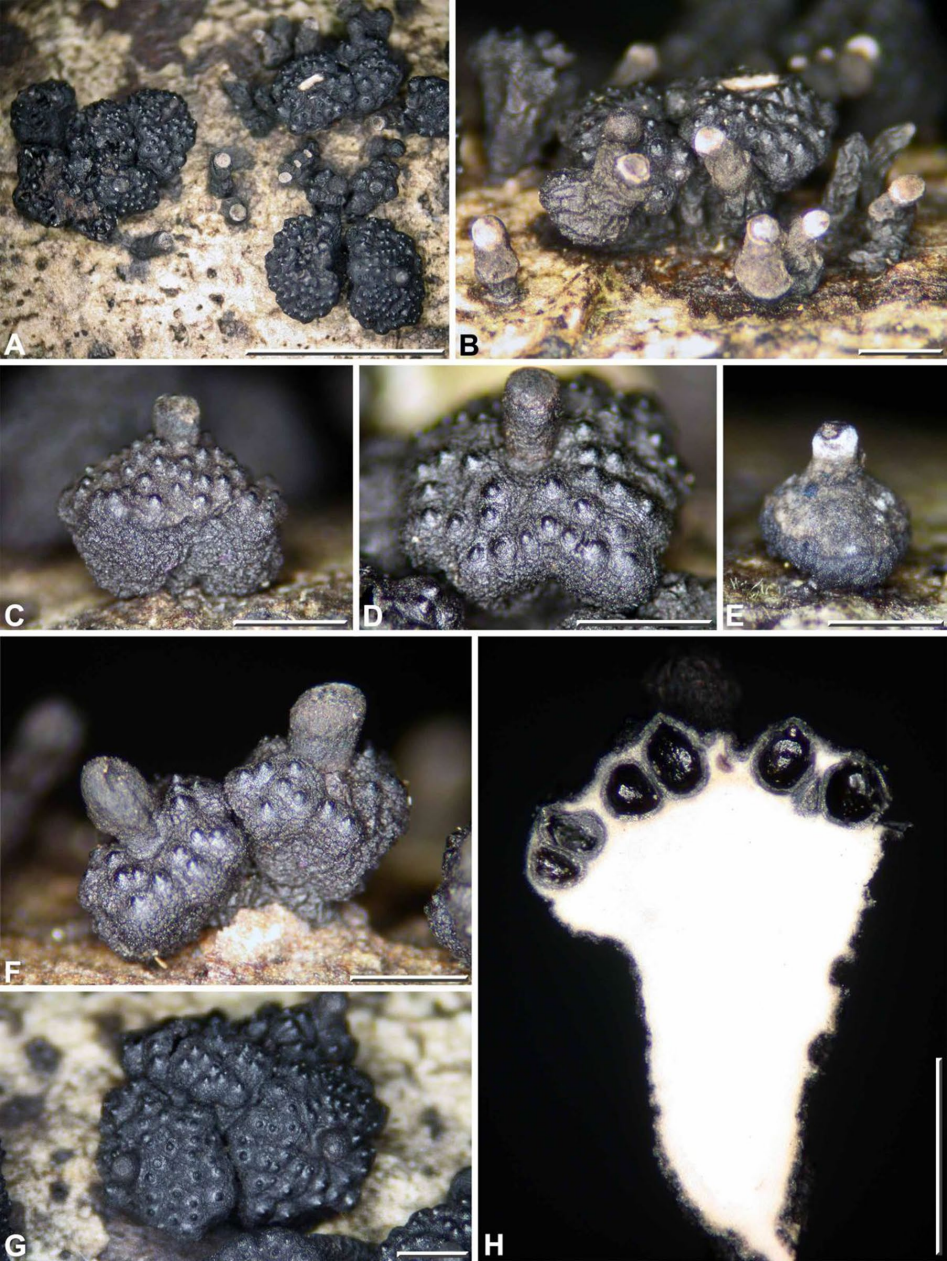
*Xylaria turbiniformis* (*GYJF12141* holotype). **A, B** Clustered stromata on bark, associated with coremial structures. **C, D, F** Spinning top-shaped stromata in side view, showing papillate ostioles and finely corky-cracked lower half. **E** Juvenile stroma. **G** Coalescent stromata with reduced central structure. **H** Stipitate stroma in vertical section. Bars in **A** = 5 mm; **B–H** = 1 mm

**Fig. 75:**
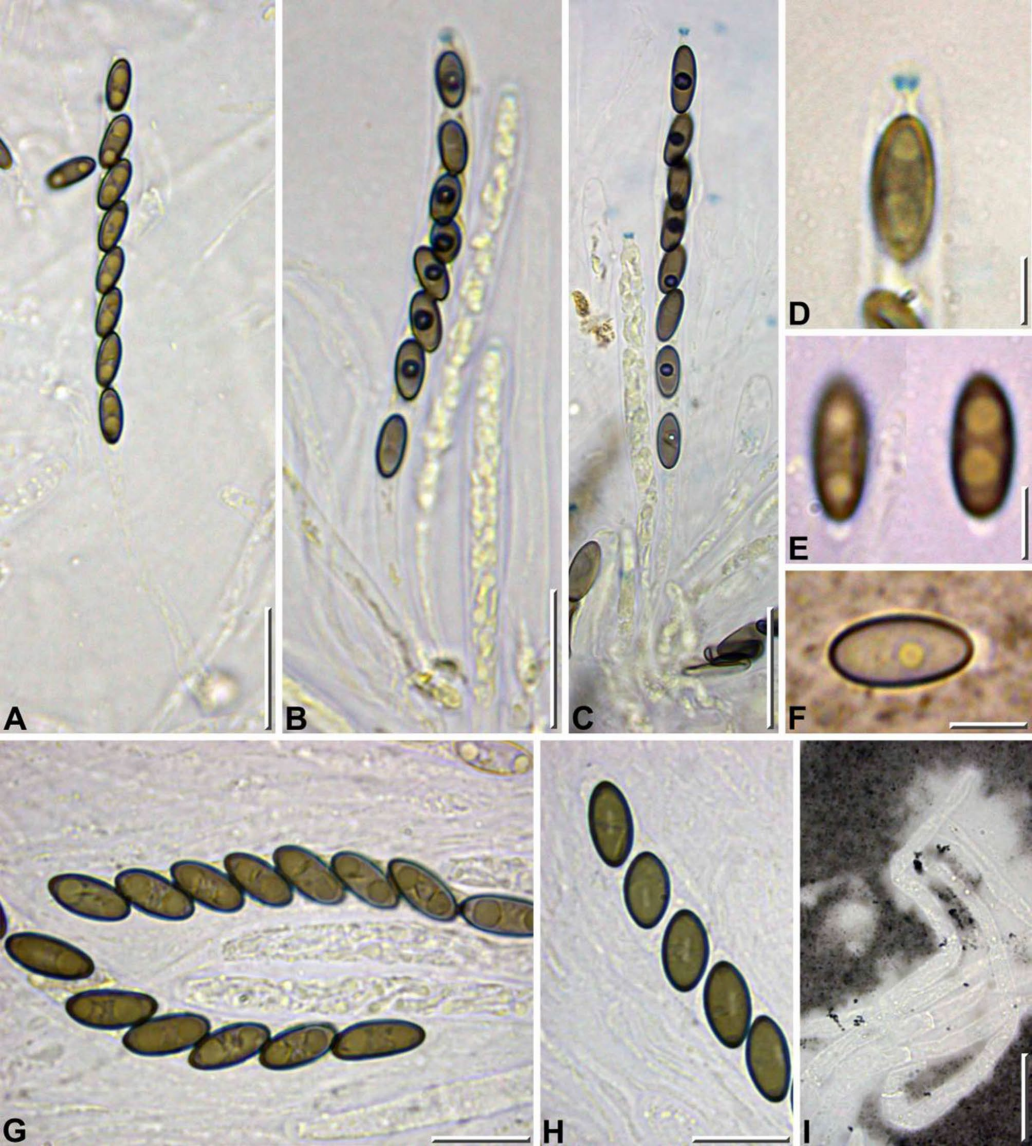
*Xylaria turbiniformis* (*GYJF12141* holotype). **A** Mature ascus in 1% SDS. **B, C** Immature and mature asci in Melzer’s reagent. **D** Ascus apex with apical apparatus wider than high, in Melzer’s reagent. **E** Ascospores showing a polar secondary appendage, in aqueous nigrosin. **F** Ascospore showing a polar secondary appendage, in dilute India ink. **G** Ascospores in 1% SDS. **H** Laterally swollen ascospores showing short germ slits, in 1% SDS. **I** Paraphyses in India ink. Bars in **A–C** = 20 µm; **D–F** = 5 µm; **G–I** = 10 µm

***Xylaria turbiniformis*** J. Fourn., Y.-M. Ju, H.-M. Hsieh & Lechat, sp. nov. Figures 74, 75

MycoBank MB863192

**Typification**: **FRENCH GUIANA.** Sinnamary, Paracou, CIRAD field centre, trail to Guyaflux, 5.249218 N, 52.915431 W, lowland rainforest, on dead corticated branch, 23 Jun 2012, *Fournier, J. GYJF12141* (holotype HAST 147512).

**Etymology**: From Latin *turbo, turbinis =* spinning top and *forma* = shape, referring to the stromatal shape resulting from development around a persistent, central coremial peg.

**Diagnosis**: Differs from known penzigioid species of *Xylaria* associated with coremia by stromata developing around a central upright coremial structure, with a thin leathery crust, coarsely papillate ostioles, and ellipsoid-equilateral, light to medium-brown ascospores averaging 9.0 × 3.8 µm, with a very short germ slit and a polar secondary appendage.

**Anamorphic stromata** superficial, upright, cylindrical to flask-shaped, 1–2 mm high × 0.5–0.7 mm diam at base, with a light grayish brown powdery coating, apically white and bluntly rounded, sterile, the base becoming gradually spherical and blackish.

**Teleomorphic stromata** resulting from a basal swelling of anamorphic stromata, separate to coalescing in small clusters, black, glabrous, 1.6–2.2 mm high × 1.0–2.5 mm diam, with short, obconical, finely corky-cracked stipes and discoid, flat to convex fertile parts usually bearing a central cylindrical sterile remnant of the previous anamorphic state, at times broken or lacking; surface finely corky-cracked, roughened by slightly exposed perithecial contours and projecting ostioles, crust ca. 40 µm thick, leathery, lacking a peeling outer layer; interior pure white, spongy, homogeneous, extending into the stipe. **Perithecia** subglobose to ovoid, 0.25–0.30 mm high × 0.25 mm diam. **Ostioles** coarsely papillate, conical or bluntly rounded, shiny black, 80–90 µm diam at base.

**Paraphyses** hyphal, hyaline, remotely septate, simple, 3.0–3.5 µm wide at base, tapering above asci, embedded in mucilaginous material. **Asci** cylindrical, with (6–)8 uniseriately arranged, slightly overlapping ascospores, the spore-bearing parts 62–72 µm long × 5.0–5.5 µm broad, the stipes 15–45 µm long, with apical apparatus discoid to faintly trapezoid, wider than high, 0.7–1.1 × 1.4–1.9 µm (Me = 0.9 × 1.6 µm, *N* = 18), bluing in Melzer’s reagent. **Ascospores** (7.8–)8.4–9.8(−10.5) × (3.4–)3.6–4.1(−4.3) µm, Q = (2.0–)2.1–2.7(−3.1) *N* = 60 (Me = 9.0 × 3.8 µm, Qe = 2.4), ellipsoid-equilateral with mostly broadly rounded ends, light olivaceous brown in the fresh state, medium-brown in the dry state, with a short, central, straight germ slit 2.0–3.0 µm long and a hemispherical polar secondary appendage not stained in aqueous nigrosin, persistent, also visible by contrast in dilute India ink; epispore smooth.

**Asexual morph** on the natural substrate not observed.

**Known distribution**: French Guiana, known only from the type collection.

**Comments**: This penzigioid *Xylaria* is remarkable for its short-stipitate stromata developing around the base of an anamorphic structure that persists as a conspicuous central peg. It resembles *X. frustulosa* in gross morphology, especially in having pulvinate stromata with a thin leathery crust and coarsely papillate ostioles, combined with small equilateral ascospores. However, the ascospores of *X. turbiniformis* differ from those of *X. frustulosa* in being larger, with more narrowly rounded ends, slightly darker brown, and in possessing a short, relatively conspicuous germ slit and a polar appendage.

It should be compared with *X. carabayensis* (Mont.) Y.-M. Ju, H.-M. Hsieh & J. D. Rogers, which also has small penzigioid stromata with a central coremial peg but differs mainly by much larger, darker brown ascospores 27.5–35 × 15–19.5 µm, with a long sigmoid to spiral germ slit (Ju et al. 2012).

In our phylogenetic analyses, *X*. *turbiniformis* clusters with *X*. *myosurus*, which also has light brown, nearly equilateral ascospores with broadly rounded ends, similar in size and averaging 8.7 × 3.3 µm. However, *X*. *myosurus* differs markedly by having upright stromata and an inconspicuous, nearly spore-length ascospore germ slit.

**Fig. 76:**
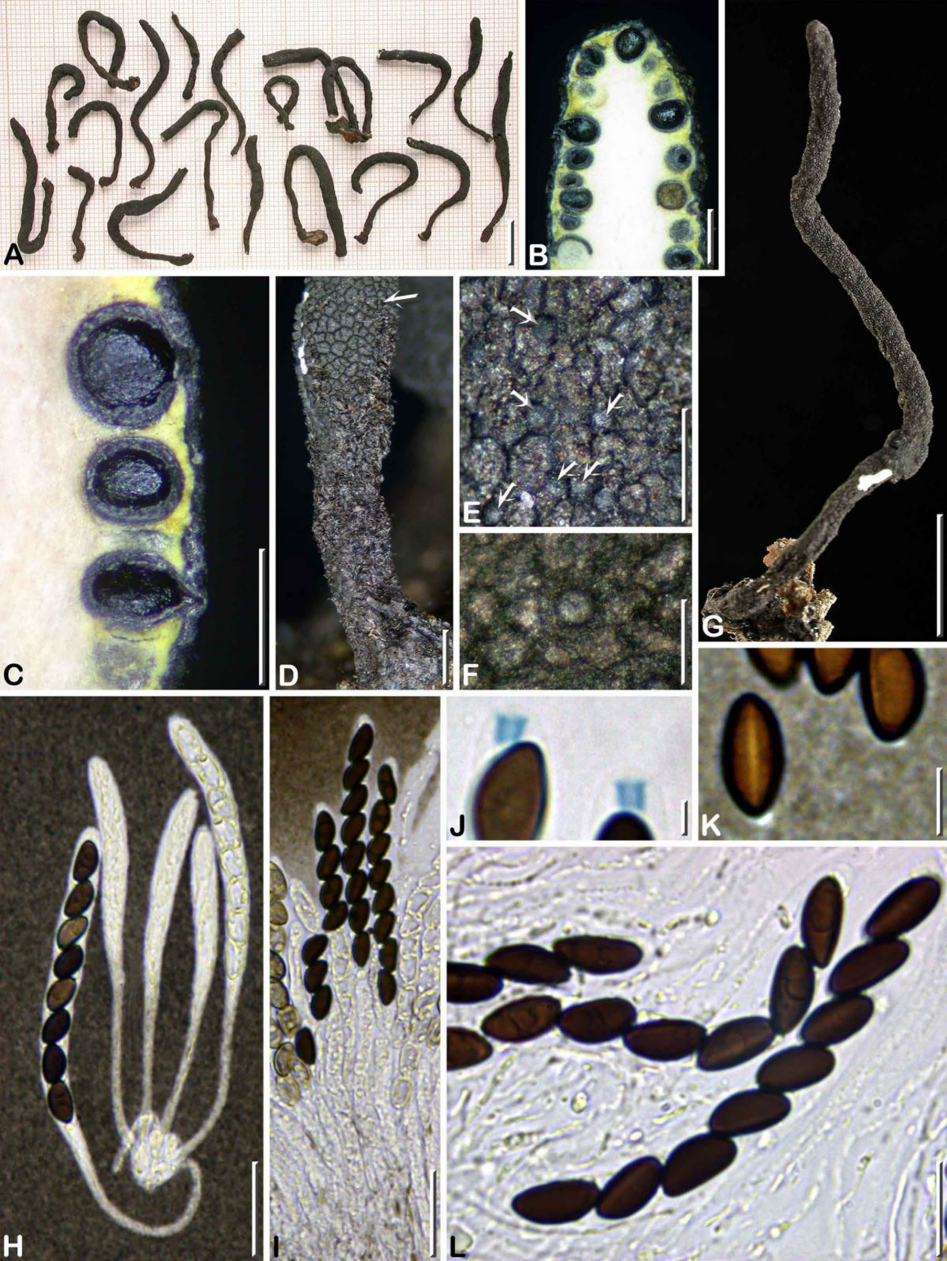
*Xylaria uncinata* (**A, C, E, F, L ***GYJF21195* holotype; **B, D, G–K ***GYJF19024-2* paratype). **A** Variously bent stromata. **B** Stromatal apex in longitudinal section showing perithecia lining the apex and a citrine-yellow tissue present beneath surface. **C** Vertical section showing perithecia immersed in a yellow tissue beneath a thin black crust. **D** Stipe in side view showing a reticulately cracked surface in upper part (arrow) and a hairy-tomentose coating in lower part. **E** Stromatal surface in close-up showing bluntly papillate ostioles (arrows) barely emerging among small warts. **F** Close-up on a black ostiole and surrounding warts. **G** Sinuous upright stroma (note the white flesh of the stipe in the bruised part). **H, I** Bunches of immature and mature asci, in India ink. **J** Ascal apical apparati in Melzer’s reagent. **K** Ascospores showing a germ slit and sparse mucilaginous remnants on ends, in India ink. **L** Mature ascospores, some showing a germ slit, in 1% SDS. Bars in **A** = 10 mm; **B, C, E** = 0.5 mm; **D** = 1 mm; **F** = 0.2 mm; **G** = 5 mm; **H, I** = 20 µm; **J** = 2 µm; **K** = 5 µm; **L** = 10 µm

***Xylaria uncinata*** J. Fourn., Y.-M. Ju, H.-M. Hsieh & Lechat, sp. nov. Figure 76, Table 49

**Table 49:** Ascospore dimensions in two collections of *X. uncinata* showing their variation range, compared with those recorded from two Australasian collections of *X. plebeja*

Collection numbers	Ascospore measurements with extreme values in parentheses	Q = quotient l/w, *N* = number of measurements	Me = mean values of l/w, Qe = mean quotient l/w
*GYJF19024-2*	(7.3–)7.6–8.5(−9.4) × (3.7–)4.0–4.5(−4.8) µm	Q = (1.6–)1.7–2.1(−2.3), *N* = 60	Me = 8.1 × 4.2 µm, Qe = 1.9
*GYJF21195*	(7.2–)7.6–9.0(−9.4) × (3.7–)4.0–4.6(−4.7) µm	Q = (1.6–)1.7–2.2(−2.4), *N* = 60	Me = 8.3 × 4.3 µm, Qe = 1.9
**Cumulated values**	**(7.2–)7.6–9.0(−9.4) × (3.7–)4.0–4.5(−4.8) µm**	**Q = (1.6–)1.7–2.1(−2.4), N = 120**	**Me = 8.2 × 4.3 µm, Qe = 1.9**
*X. plebeja* Hainan *YZ070813*	(8.2–)8.5–9.6(−9.8) × (3.9–)4.2–5.0(−5.4) µm	Q = (1.7–)1.8–2.2(−2.4), *N* = 60	Me = 9.1 × 4.6 µm, Qe = 2.0
*X. plebeja* Taiwan *YM-J & HM-H 92031212*	(7.9–)8.3–9.4(−9.8) × (3.7–)3.9–4.5(−4.6) µm	Q = (1.8–)1.9–2.3(2.5), *N* = 60	Me = 8.8 × 4.2 µm, Qe = 2.1

MycoBank MB863193

**Typification**: **FRENCH GUIANA.** Maripasoula, Saül, trail head of Sentier Roche-Bateau, 3.62056 N, 53.199899 W, ca. 240 m, disturbed secondary rainforest, on dead wood, 30 Mar 2021, *Fournier, J. GYJF21295* (holotype HAST 147514).

**Etymology**: From Latin *uncinatus =* hooked, bent, referring to the typically strongly curved or twisted stromata.

**Diagnosis**: Differs from *X. plebeja* by narrowly cylindrical, terete, strongly bent stromata, smaller ostioles, slightly smaller ascospores, and a Neotropical distribution.

**Stromata** scattered to gregarious, upright, simple, 12–45 mm in total height, the fertile head 6–30 mm high × 1.5–2.5(−3.0) mm diam, narrowly cylindrical, terete to slightly flattened in places, slightly sinuous to most often strongly curved, brittle, apically rounded and fertile, with perithecial contours unexposed; the stipes ill-defined, 4–12 mm high × 1.5–2 mm diam, straight to slightly curved, terete to flattened, blackish, flesh white lacking yellow tone, reticulately cracked in upper part, tomentose in lower part and slightly swollen at base; surface blackish brown, glabrous, densely reticulately cracked into minute, polygonal, thick warts and roughened by ostioles; subsurface a black leathery crust 25–40 µm thick; interior solid, whitish, soft-textured, with a conspicuous citrine-yellow layer embedding the perithecia, due to minute yellowish granules accumulated within the hyphae, not changing color nor dissolving in 3% KOH. **Perithecia** globose to subglobose, 0.25–0.40 mm diam, laterally flattened when in contact. **Ostioles** obtusely rounded to flattened, dull black, 80–120 µm diam, barely prominent above the cracked surface.

**Paraphyses** sparse, hyphal, thin-walled, 5–8 µm wide at base, tapering to 1.5–2.5 µm wide above asci, embedded in mucilaginous material. **Asci** cylindrical, with 8 slightly overlapping uniseriately arranged ascospores, the spore-bearing parts 54–66 × 5.0–6.0 µm, the stipes 48–52 µm long, with apical apparatus short-cylindrical to quadrate, apically flattened with a faint lateral rim, 1.6–2.0 × 1.4–1.7 µm (Me = 1.8 × 1.6 µm, *N* = 20), bluing in Melzer’s reagent. **Ascospores** (7.2–)7.6–9.0(−9.4) × (3.7–)4.0–4.5(−4.8) µm, Q = (1.6–)1.7–2.1(−2.4), *N* = 120 (Me = 8.2 × 4.3 µm, Qe = 1.9), ellipsoid slightly inequilateral, with most often broadly rounded ends, dark brown, unicellular, with a conspicuous, straight, longitudinally oriented germ slit almost spore-length on the ventral side; lacking a mucilaginous sheath and secondary appendages; epispore smooth.

**Asexual morph** on the natural substrate not observed.

**Other specimen examined (paratype)**: **FRENCH GUIANA.** Maripasoula, Saül, shortcut to the airfield, 3.623524 N, 53.206542 W, ca. 200 m, disturbed secondary rainforest, on dead corticated trunk, mixed with *X. crustulata*, 17 Jun 2019, *Fournier, J. GYJF19024-2* (HAST 147513).

**Known distribution**: French Guiana, only known from two collections.

**Comments**: *Xylaria uncinata* was initially overlooked because it was growing intermixed with stromata of *X. crustulata*, from which it differs in gross morphology by more slender stromata and smaller ostioles. Closer examination showed additional differences, including smaller superficial warts and less deeply cracked stipes that are hairy-tomentose at the base. The most informative character is revealed in section: the perithecia are embedded in a citrine-yellow tissue layer. A colored tissue layer embedding the perithecia is distinctive and, to our knowledge, is otherwise reported in *X*. *intracolorata* (J. D. Rogers, Callan & Samuels) J. D. Rogers & Y.-M. Ju, *X. luteostromata* C. G. Lloyd and its var. *macrospora* J. D. Rogers & Samuels, *X. martinicensis* J. Fourn. & Lechat and its var. *microspora* J. Fourn. & Lechat, *X*. *palifera* (this paper), and *X. plebeja* Ces.

*Xylaria luteostromata* and its var. *macrospora,* known respectively from the Philippines (Ju et al. 2016) and New Zealand (Rogers and Samuels 1986), differ from *X*. *uncinata* by more robust stromata, an orange perithecium-embedding tissue layer, and larger ascospores, respectively 8.5–11 × 4.5–5.5 µm and 11.5–14 × 5.5–6.5 µm.

*Xylaria martinicensis* and its var. *microspora*, known from Martinique, and *X. palifera* differ from *X. uncinata* by more coarsely warted stromata, kretzschmarioid to penzigioid stromata, wider ostioles, and larger ascospores, respectively 12.9–15.3 × 5.9–7.0 μm and 9.0–10.4 × 4.6–5.2 μm (Fournier et al. 2020), and 9.3 × 4.8 µm in *X*. *palifera*. *Xylaria intracolorata*, known from Indonesia and Taiwan, is another kretzschmarioid species, differs from *X*. *uncinata* by an orange perithecium-embedding tissue layer and larger ascospores (Rogers et al. 1987; Rogers and Ju 1998; Ju and Rogers 1999).

Phylogenetically, *X. subplebeja* is the closest species to *X*. *uncinata*. It has ascospores of similar dimensions (Table 49) but differs by more robust stromata, 4–9(−15) mm diam, and wider ostioles, 150–170 µm diam. *Xylaria plebeja* is another resembling species and is sister to the subclade containing *X*. *crustulata*, *X. martinicensis* and its var. *microspora*, *X*. *palifera*, *X*. *subplebeja*, and *X*. *uncinata*. It differs from *X*. *uncinata* mainly by more robust stromata, larger ascospores, and an Asian distribution.

Notably, none of the taxa mentioned above is known to have the narrowly cylindrical and deeply curved stromata characteristic of *X. uncinata*. The key feature shared by most of these species is the presence of the yellow to orange tissue layer embedding the perithecia. This tissue layer may be inconspicuous, especially in dried or old material. The coloration is enhanced by rehydration of the tissue and must be checked carefully before concluding that it is present or absent.

**Fig. 77:**
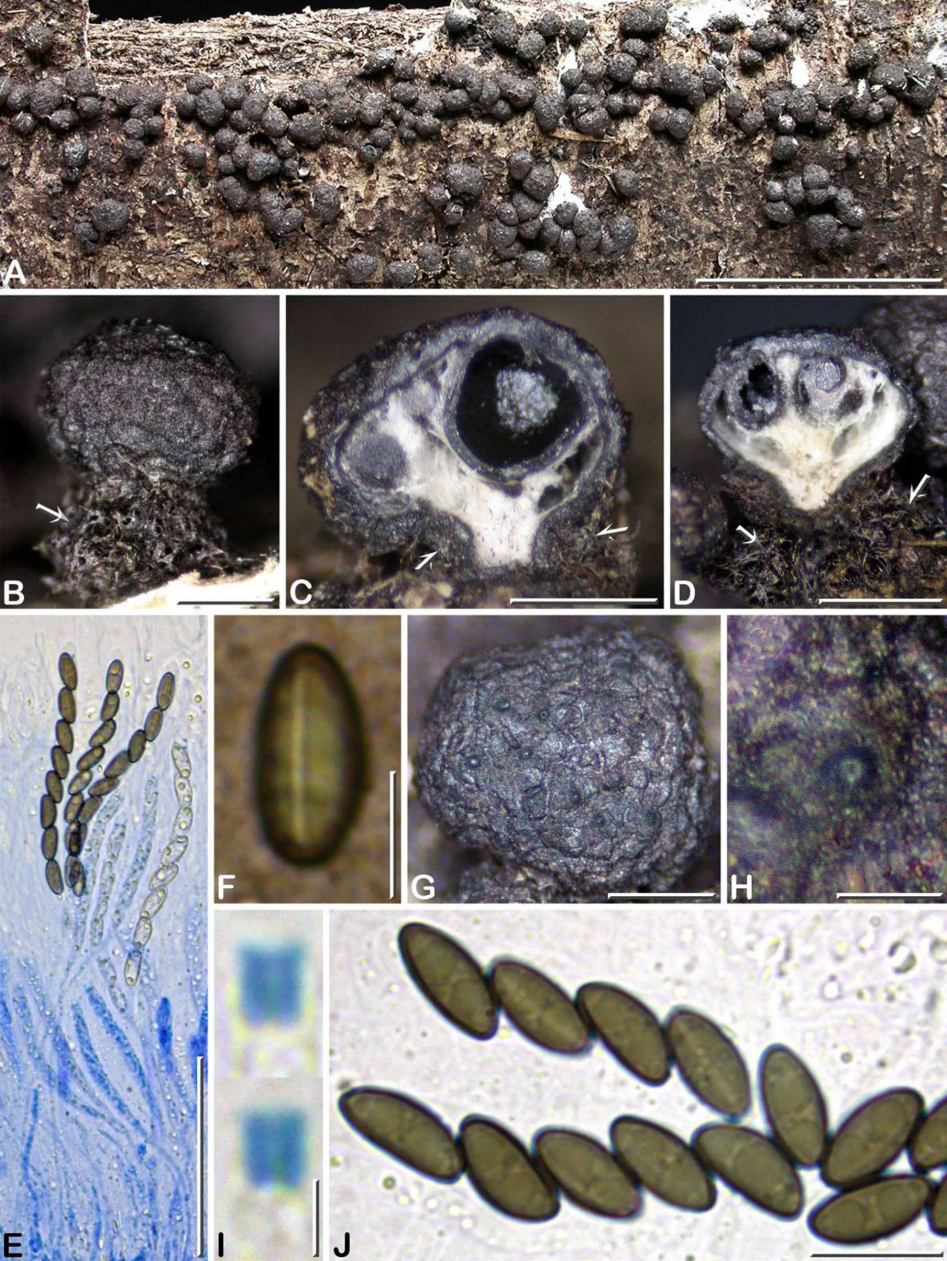
*Xylaria villosipes* (*GYJF21323* holotype). **A** Habit of gregarious stromata on bark. **B** Isolated stroma in side view showing a basal pad of dark brown tomentum (arrow). **C, D** Stromata in vertical section showing perithecia beneath a carbonaceous crust and basal tomentum around a short stipe (arrows). **E** Mature and immature asci in blue Pelikan ink. **F** Ascospore in ventral view in India ink showing a germ slit and absence of sheath or appendage. **G** Stoma in top view showing a finely cracked surface and scattered ostioles. **H** Ostiole in close-up. **I** Ascal apical apparati in Melzer’s reagent. **J** Ascospores in 1% SDS. Bars in **A** = 10 mm; **B–D, G** = 0.5 mm; **H** = 100 µm; **E** = 50 µm; **F** = 5 µm; **I** = 2 µm; **J** = 10 µm

***Xylaria villosipes*** J. Fourn., Y.-M. Ju, H.-M. Hsieh & Lechat, sp. nov. Figure 77

MycoBank MB863194

**Typification**: **FRENCH GUIANA.** Maripasoula, Saül, trail head to Sentier Roche-Bateau, Crique-Cochon, 3.625994 N, 53.1955 W, 210–220 m, disturbed secondary rainforest, on dead corticated branch, 31 Mar 2021, *Fournier, J. GYJF21323* (holotype HAST 147515).

**Etymology**: From Latin *villosus =* hairy and *pes* = foot, referring to the dense tomentum around the basal connective of stromata.

**Diagnosis**: Distinguished by the combination of small, gregarious, subglobose, penzigioid stromata rarely over 1 mm diam, lacking exposed perithecial contours; a faintly cracked surface with inconspicuous ostioles; a thin carbonaceous subsurface layer; a densely hairy-tomentose basal connective; and ellipsoid-inequilateral ascospores averaging 9.3 × 4.1 µm.

**Stromata** superficial, gregarious, clustered in small groups, separate to occasionally coalescent, subglobose, more or less depressed-spherical, upper part convex, 0.8–1.2 mm high, 0.7–1.4(−1.8) mm diam, subsessile, the lower part ending into a narrow central connective surrounded by a dense, conspicuous, persistent sleeve of dark brown stiff hairs basally spreading on the substrate; surface blackish, lacking exposed perithecial contours, cracked into small polygonal scales, some bearing remnants of a silvery gray superficial pellicle; subsurface a thin, carbonaceous, brittle crust 40–70 µm thick; interior white, loosely fibrous, lacunose, radiating from the basal connective. **Perithecia** (1–)2–15 per stroma, subglobose to obovoid, 0.25–0.5 mm diam. **Ostioles** obtusely papillate, barely prominent, porate, dull black, 60–70 µm diam at base.

**Paraphyses** hyphal, hyaline, remotely septate, simple, minutely guttulate, 2.5–3.0 µm wide at base, tapering to 0.8–1.0 µm wide, embedded in thick mucilaginous material. **Asci** 6–8-spored, cylindrical, short-stipitate, the spore-bearing parts 63–80 × 6–6.5 µm, the stipes 80–90 µm long, with apical apparatus shortly tubular to quadrate in outline, apically flattened with a faint rim, 1.8–2.3 × 1.4–1.7 µm (Me = 2.0 × 1.6 µm, *N* = 25), bluing in Melzer’s reagent. **Ascospores** (8.3–)8.7–10.0(−10.8) × (3.5–)3.9–4.3(−4.5) µm, Q = (2.0–)2.1–2.5(−2.7), *N* = 60 (Me = 9.3 × 4.1 µm, Qe = 2.3), ellipsoid slightly inequilateral, mostly with broadly rounded ends, medium brown, olivaceous in the fresh state, with a conspicuous straight germ slit spore-length on the ventral side; lacking appendages or mucilaginous sheath.

**Asexual morph** on the natural substrate not observed.

**Other specimen examined (paratype)**: **FRENCH WEST INDIES.** Martinique, Case-Pilote, Fond Bourlet, Prise d’Eau, hygrophilic rainforest, on dead corticated wood, 21 Aug 2005, *Lechat, C. CLL5109* (HAST 142997).

**Known distribution**: French Guiana; Martinique (Fournier et al. 2018c).

**Comments**: This penzigioid *Xylaria* is characterized by minute, gregarious stromata with a thin, brittle carbonaceous crust and a short connective surrounded by a persistent sleeve of dark brown hairs. The latter feature is highly unusual among penzigioid species of *Xylaria*. The microscopic features are not distinctive, but the stromatal morphology suggests an undescribed taxon, which is supported by its relatively isolated position in our phylogram.

This combination of characters corresponds well to that of the undescribed species *Lechat CLL5109* from Martinique reported by Fournier et al. (2018c), in which hairy stipes were noted and illustrated, albeit less developed than in the Guianese collection. The conidia produced on the natural substrate in *Lechat CLL5109* conform to those obtained from the holotype material (unpublished data), confirming their conspecificity.

**Fig. 78:**
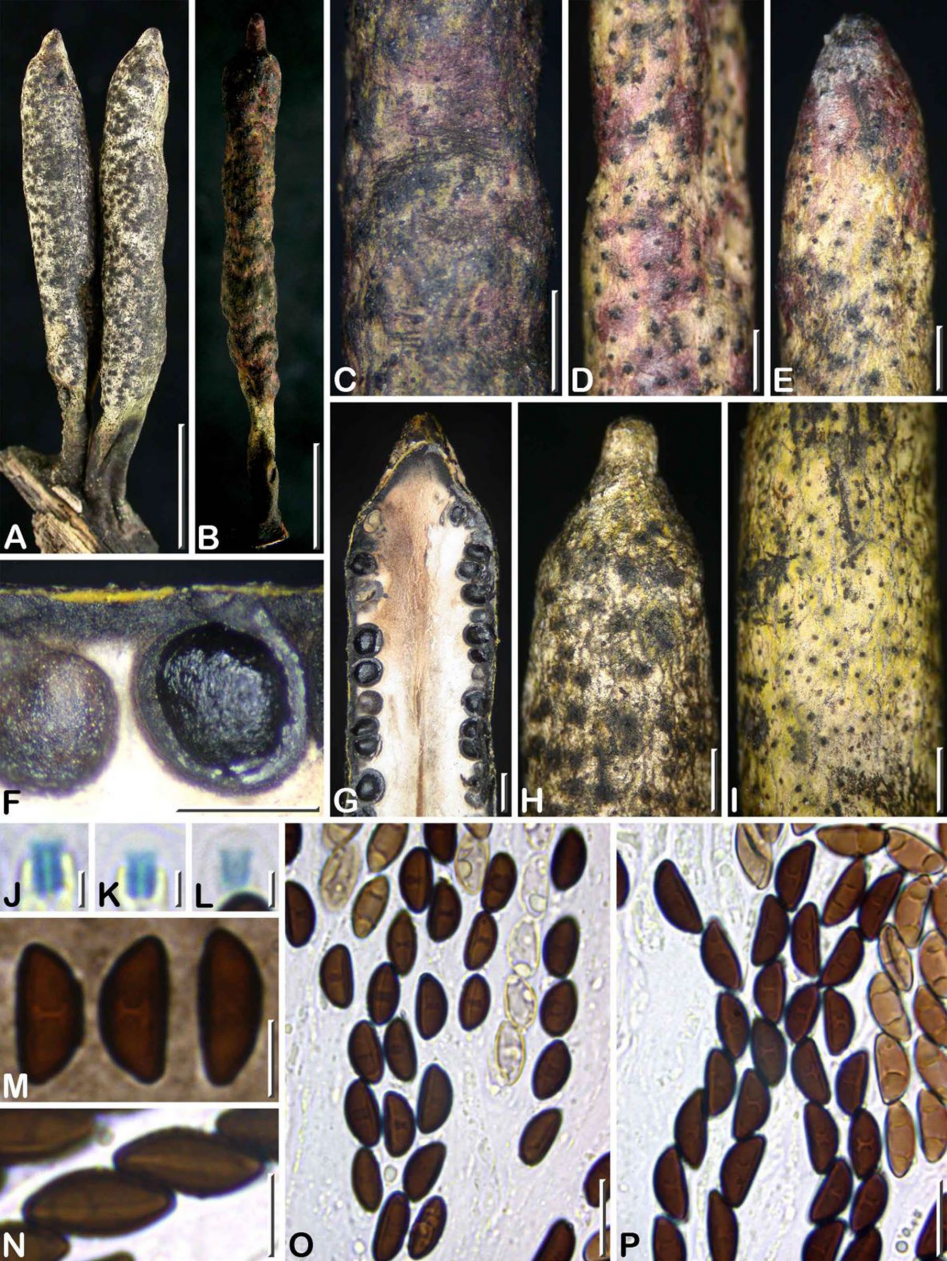
*Xylaria vinosa* (**A, G, N ***GYJF18050*; **B, C ***GYJF21208-2*; **D, E, L, O ***GYJF21086*; **F, H–K, M, P ***GYJF19148*). **A** Stromata with grayish surface mottled with black. **B** Stroma with blackish and purple surface. **C** Stromatal surface with mixed grayish, blackish and purple zones. **D, E** Stromatal surface with purple marks on an olivaceous yellow background. **F** Stromatal crust in vertical section showing a perithecium beneath a thick carbonaceous layer coated by a thin yellow pellicle. **G** Apex of a stroma in longitudinal section. **H** Apex of a stroma with grayish surface mottled with blackened ostiolar areas. **I** Olivaceous yellow stromatal surface dotted with small black ostioles. **J–L** Ascal apical apparati, in Melzer’s reagent. **M** Ascospores in side view, in India ink. **N** Ascospores in ventral view showing a germ slit. **O, P** Variously oriented ascospores (**N–P** in 1% SDS). Bars in **A** = 10 mm; **B** = 5 mm; **C–E**, **G–I** = 1 mm; **F** = 0.5 mm; **J–L** = 2 µm; **M, N** = 5 µm; **O, P** = 10 µm

***Xylaria vinosa*** J. Fourn., Y.-M. Ju & Lechat, in Fournier, Lechat & Courtecuisse, Ascomycete.org 12 (3): 156. 2020. Figure 78, Table 50

**Table 50:** Ascospore dimensions in eight collections of *X. vinosa* from French Guiana showing a fairly wide variation range, compared with those of *X. vinosa* reported from the French West Indies by Fournier et al. (2020)

Collection numbers	Ascospore measurements with extreme values in parentheses	Q = quotient l/w, *N* = number of measurements	Me = mean values of l/w, Qe = mean quotient l/w
*GYJF18050*	(8.3–)8.8–10.1(−11.0) × (3.6–)3.8–4.4(−4.7) µm.	Q = (2.0–)2.1–2.5(−2.8), *N* = 60	Me = 9.5 × 4.1 µm, Qe = 2.3
*GYJF18087*	(8.3–)9.1–10.3(−10.5) × (3.5–)3.8–4.5(−4.7) µm	Q = (2.0–)2.1–2.6(−2.8), *N* = 60	Me = 9.6 × 4.2 µm, Qe = 2,3
*GYJF19083*	(8.2–)8.7–10.0(−10.5) × (3.8–)3.9–4.4(−4.6) µm	Q = 2.0–2.4(−2.7), *N* = 60	Me = 9.3 × 4.2 µm, Qe = 2.2
*GYJF19148*	(8.5–)8.9–10.4(−11.1) × (3.5–)3.8–4.3(−4.5) µm	Q = (2.0–)2.2–2.7(−2.9), *N* = 60	Me = 9.5 × 4.0 µm, Qe = 2.4
*GYJF19208-2*	(7.3–)8.0–9.4(−9.9) × (3.4–)3.8–4.4(−4.8) µm	Q = (1.8–)2.0–2.4(−2.5), *N* = 60	Me = 8.7 × 4.0 µm, Qe = 2.2
*GYJF21086*	(7.5–)8.1–9.0(−9.4) × (3.6–)3.8–4.3(−4.6) µm	Q = (1.8–)1.9–2.3 (−2.4), *N* = 60	Me = 8.6 × 4.1 µm, Qe = 2.1
*GYJF21208*	(8.9–)9.2–10.1(−11.0) × (3.7–)4.0–4.5(−4.8) µm	Q = (1.9–)2.1–2.5(−2.7), *N* = 60	Me = 9.7 × 4.2 µm, Qe = 2.3
*GYJF21237*	(8.5–)8.8–9.8(−10.4) × (3.8–)4.2–5.0(−5.3) µm	Q = (1.8–)1.9–2.2(−2.5), *N* = 60	Me = 9.3 × 4.5 µm, Qe = 2.1
**Cumulated values**	**(7.3–)8.0–10.4(−11.1) × (3.1–)3.8–5.0(−5.3) µm**	**Q = (1.8–)1.9–2.7(−2.9), N = 480**	**Me = 9.3 × 4.2 µm, Qe = 2.2**
X. *vinosa* with olivaceous tone *MJF14022*	(9.0–)9.5–11.1(−11.6) × (3.6–)4.0–4.5(−4.9) μm	Q = (2.1–)2.2–2.6(−2.8), *N* = 60	Me = 10.1 × 4.2 μm, Qe = 2.4
*X. vinosa* FWI (Fournier et al. 2020)	(8.2–)9.3–11.1(−11.7) × (3.4–)3.7–4.8(−5.2) μm	Q = (1.9–)2.1–2.8(−3.0), *N* = 360	Me = 10.2 × 4.3 μm, Qe = 2.4

**Stromata** scattered or in small to rarely large groups, upright, separate, rarely connate at base by 2–3, simple, rarely branched, mostly narrowly fusiform, straight to curved, terete, with unexposed perithecial contours, occasionally slightly constricted or flattened in places, most often with a distinctly mucronate to conical sterile apex, subsessile to long-stipitate, 23–88 mm in total height, the fertile head 11–42 mm high × 3–4(−5) mm diam; surface even to finely reticulately-cracked and scaly, light to dark gray with scattered olivaceous yellow, more rarely purple marks, or entirely olivaceous yellow with or without purple marks, gradually blackening; subsurface black, hard-textured, a slightly carbonaceous crust 150–170 µm thick, overlain by a gray to yellow appressed outer layer not changing color in presence of NH3 or lactic acid but readily yielding curry-colored yellow pigments in 3% KOH; the stipes usually well-defined, 6–48 mm high × 1.5–3 mm diam, black, glabrous, frequently with yellow marks, terete to flattened, puckered, enlarged at base; interior whitish to orange-brown in places, solid, fibrous. **Perithecia** subglobose 0.4–0.7 mm diam, laterally flattened when crowded. **Ostioles** obtusely papillate, barely prominent, black, often inconspicuous, 80–100 µm diam at base.

**Paraphyses** copious, hyphal, remotely septate, thin-walled, conspicuously guttulate, 4–6 µm wide at base, tapering to 1.0–1.5 µm wide above asci, embedded in mucilaginous material. **Asci** cylindrical, long-stipitate, with (6–)8 slightly overlapping uniseriately arranged ascospores, the spore-bearing parts (55–)62–75 × 5.0–5.5 µm, the stipes 90–120 µm long, with apical apparatus tubular with a faint upper rim, 1.8–2.5 × 1.3–1.6 µm (Me = 2.2 × 1.5 µm, *N* = 50), bluing in Melzer’s reagent.

**Ascospores** (7.3–)8.0–10.4(−11.1) × (3.1–)3.8–5.0(−5.3) µm, Q = (1.8–)1.9–2.7(−2.9), *N* = 480 (Me = 9.3 × 4.2 µm, Qe = 2.2), ellipsoid-inequilateral with narrowly to broadly rounded ends, brown to dark brown, unicellular, with a thin, straight, longitudinally oriented germ slit slightly less than spore-length on the ventral side; lacking appendages or mucilaginous sheath; epispore smooth.

**Specimens examined: FRENCH GUIANA.** Maripasoula, Saül, trail head to Roche-Bateau, disturbed secondary rainforest, 3.620498 N, 53.199309 W, on dead blackened wood, 22 Jun 2019, *Fournier, J. GYJF19208-2* (HAST 147516); Roura, Cacao, Camp Bonaventure, 4.323044 N, 52.339371 W, ca. 60 m, hygrophilic secondary rainforest, on dead corticated branch, 23 Mar 2021, *Fournier, J. GYJF21086* (HAST 147517); Maripasoula, Saül, shortcut to the airfield, 3.622159 N, 53.204166 W, disturbed secondary rainforest, on dead trunk, 20 Jun 2019, *Fournier, J. GYJF19148* (HAST 147518); Maripasoula, Saül, trail head to Sentier des Gros Arbres, 3.620324 N, 53.207951 W, ca. 210 m, disturbed secondary rainforest, on dead blackened wood, 18 Jun 2019, *Fournier, J. GYJF19083* (HAST 147519); Maripasoula, Saül, trail head to Roche-Bateau, 3.619046 N, 53.200658 W, disturbed secondary rainforest, on blackened wood, 23 Aug 2018, *Gardiennet, A. GYJF18087* (HAST 147520); Maripasoula, Saül, trail head to Sentier des Gros Arbres, 3.6201 N, 53.207989 W, ca. 210 m, disturbed secondary rainforest, on dead blackened wood, 28 Mar 2021, *Fournier, J. GYJF21208* (HAST 147521); Maripasoula, Saül, shortcut to the airfield, 3.622852 N, 53.205768 W, 210–220 m, disturbed secondary rainforest, on dead blackened wood, 29 Mar 2021, *Fournier, J. GYJF21237* (HAST 147522); Maripasoula, Saül, trail head to Monts La-Fumée, 3.629382 N, 53.206831 W, disturbed secondary rainforest, on dead hanging decorticated branch, 21 Aug 2018, *Fournier, J. GYJF18050* (HAST 147523).

**Comments:** *Xylaria vinosa* was described from Guadeloupe and Martinique (FWI) to accommodate collections with fusiform, hard-textured, mucronate stromata with a buff to purple surface and dark brown, inequilateral ascospores averaging 10.2 × 4.3 μm (Fournier et al. 2020). Two deviating stromata with an olivaceous surface, mixed with typical purple stromata and featuring ascospores in the same size range, were noted but not included under *X. vinosa* because molecular data were lacking.

The numerous collections from French Guiana reported herein differ from the original definition of *X. vinosa* by having more narrowly fusiform stromata, most often with an olivaceous yellow surface closely matching the two deviating stromata from Martinique, and by slightly smaller ascospores (Table 50). However, many stromata show more or less extensive purple marks on the olivaceous yellow surface, and most other morphological characters agree with *X. vinosa*, strongly suggesting conspecificity of the Guianese collections with the species.

This interpretation is partly supported by ITS sequence similarities, but some discrepancies among collections are not clearly correlated with surface color or ascospores dimensions. At present, it seems more difficult to segregate a distinct species than to merge all collections in *X*. *vinosa*, thus rendering the species highly variable.

### Species reported from French Guiana but not recently recollected by Fournier and coworkers

***Xylaria aenea*** Mont., Ann. Sci. Nat. Bot., sér. IV, 3: 100. 1855.

The type of this species was collected from French Guiana, and it was recently documented by Ju and Hsieh (2014).

**Specimens examined: FRENCH GUIANA.** Cayenne, on wood, *Leprieur, F. R. 1399* (lectotype PC 0096744 ex Montagne herb., isolectotype PC 0086075 ex Leprieur herb.), immature. **VENEZUELA.** Aragua, Collonia Tovar, on wood, *Fendler, A. 253* (epitype K(M) 169675 ex Berkeley herb.).

**Comments:** The Montagne type from French Guiana is immature; therefore, Ju and Hsieh (2014) designated an epitype following the concept of Dennis (1956).

***Xylaria appendiculata*** Ferd. & Winge, Bot. Tidsskr. 29: 17. 1908.

This leaf-inhabiting species was recently documented from French Guiana by Ju and Hsieh (2023).

**Specimens examined: FRENCH GUIANA.** ca. 10 km SW of Saul, track from Saul leading SW toward Mt. Galbao, elev. ca. 200 m, on dead leaves, 7–9 Jan 1986, *Samuels, G. J. & Boise, J. GS2643* (HAST 145966 ex NY); ca. 20 km SW of Saul, Mt. Galbao, Upland moist forest with relatively few epiphytes and mosses on trees, alt. 650 m, on dead leaves, 22 Jan 1986, *Samuels, G. J. & Boise, J. GS3164* (HAST 145967 ex NY).

***Xylaria aristata*** **Mont. var**. ***aristata***, Ann. Sci. Nat. Bot., sér. IV, 3: 106. 1855.

The type of this leaf-inhabiting species was collected from French Guiana, and it was recently documented by Ju and Hsieh (2023).

**Specimens examined: FRENCH GUIANA.** Cayenne City, on dead dicot leaves, Mar 1986, *Samuels, G. J. GS4508* (BPI 881467 ex NY); on fallen leaves, *Leprieur, F. R. 1209* (holotype (in two packets) PC 0096736 ex Montagne herb. 10093, PC 0096737 ex Montagne herb. 10094).

***Xylaria delicatula*** Starb., Bih. Kongl. Svenska Vetensk.-Akad. Handl. 27, Afd. 3 (9): 18. 1901.

This leaf-inhabiting species was recently documented from French Guiana by Ju and Hsieh (2023).

**Specimens examined: FRENCH GUIANA.** km 16 on road between Sinnamary and St. Elie, Orstom Research Area “Ecerex”, primary forest, on decaying dicot leaves, 20 Feb–1 Mar 1986, *Samuels, G. J. GS4039* (BPI 881417 ex NY); between SW of Saul, track between Saul and Mt. Galbao, alt. 450–500 m, on decaying leaves, 11 Jan 1986, *Samuels, G. J. & Boise, J. GS2775* (HAST 145973).

***Xylaria hyperythra*** (Mont.) Fr., Nov. Act. Reg. Soc. Sci. Upsal., ser. III, 1: 127. 1851.

≡ *Hypoxylon hyperythrum* Mont., Ann. Sci. Nat. Bot., sér. II, 13: 343. 1840.

The type of *X*. *hyperythra* was collected from French Guiana. This species was recently reported and illustrated from the French West Indies (Fournier et al. 2019).

**Specimen examined: FRENCH GUIANA.** on wood, *Leprieur, F. R. 1201* (lectotype of *Hypoxylon hyperythrum* PC ex Montagne).

**Comments:** Montagne (1840) did not cite specimens until Montagne (1855), where two specimens were cited: *Leprieur 411* (pro parte) and *1201*. The latter was cited by Dennis (1956). *Leprieur 411* is immature, possibly *X*. *grammica*.

***Xylaria microceras*** (Mont.) Fr., Nov. Act. Reg. Soc. Sci. Upsal., ser. III, 1: 128. 1851.

≡ *Hypoxylon microceras* Mont., Ann. Sci. Nat. Bot., sér. II, 13: 348. 1840.

The type of *X. microceras* was collected from French Guiana. This species was recently reported and illustrated from the French West Indies (Fournier et al. 2020).

**Specimens examined: FRENCH GUIANA.** “in sylvis montosis Kau et Sinamariensibus”, on rotten wood, 1838–1839, *Leprieur, F. R. 242* (holotype of *Hypoxylon microceras* PC ex Montagne herb. 7203, isotypes K(M) 144078, BPI 739380, PC ex Desmazières herb., PC ex Tulasne herb., UPS F-176038 ex Fries herb.).

**Comments:** The locality is not indicated on the type packets but is documented in the protologue (Montagne 1840) as “in sylvis montosis Kau et Sinamariensibus.” (in the mountainous forests of Kaw and Sinnamary). The type material thus likely comprises collections from two localities.

***Xylaria phyllocharis*** Mont., Ann. Sci. Nat. Bot., sér. IV, 3: 108. 1855.

The type of this leaf-inhabiting species was collected from French Guiana, and it was recently documented by Ju and Hsieh (2023).

**Specimens examined: FRENCH GUIANA.** Cayenne, on fallen leaves, *Leprieur, F. R. 1208* (holotype PC 0086048 ex Montagne herb., isotype PC 0086071 ex Leprieur herb.).

***Xylaria rhizocola*** (Mont.) Fr., Nov. Act. Reg. Soc. Sci. Upsal., ser. III, 1: 127. 1851.

≡ *Hypoxylon rhizocola* Mont., Ann. Sci. Nat. Bot., sér. II, 13: 344. 1840.

*Xylaria rhizocola* is seed-inhabiting and was recently documented from Brazil and French Guiana by Ju et al. (2018).

**Specimen examined: FRENCH GUIANA.** in forests beside the river Inipi, on a buried seed, Apr 1836, *Leprieur, F. R. 235* (holotype of *Hypoxylon rhizocola* PC 0096747).

***Xylaria rhizomorpha*** (Mont.) Mont., Ann. Sci. Nat. Bot., sér. IV, 3: 107. 1855.

≡ *Hypoxylon rhizomorpha* Mont., Ann. Sci. Nat. Bot., sér. II, 13: 341. 1840.

This species is known only from the type collection from French Guiana, for which a description and illustrations were provided by Rogers et al. (2005). Whether it is associated with termite nests remains to be confirmed.

**Specimen examined: FRENCH GUIANA.** on ground, May 1836, *Leprieur, F. R. 234* (holotype of *Hypoxylon rhizomorpha* PC, isotype S F5609 ex Sydow herb.).

***Xylaria ruginosa*** Mont., Ann. Sci. Nat. Bot., sér. IV, 3: 103. 1855.

This species is known only from the type collection from French Guiana, for which a description and illustrations were provided by Dennis (1956).

**Specimen examined: FRENCH GUIANA.** near Cayenne, on trunk, *Leprieur, F. R.*, *Crypt. Guyan. 485* (holotype PC 0096739).

**Comments:** Montagne (1855) indicated that no Leprieur number (“Coll. n …”) is available. The cited PC specimen is the only packet of *X*. *ruginosa* located in PC; it contains a single stroma branched several times at the stipe and undoubtedly representing the holotype. The stroma is glued on a paper card, on which “Crypt. Guyan. n° 485” and “Leprieur absque n°” were written by Montagne himself. The material is overmature, and its interpretation awaits additional material available for study.

***Xylaria spiculaticlavata*** Y.-M. Ju & H.-M. Hsieh, Bot. Stud. 64(19): 36. 2023.

This leaf-inhabiting species is known only from the type collection from French Guiana, for which a description and illustrations were provided by Ju and Hsieh (2023).

**Specimen examined: FRENCH GUIANA.** on a fallen leaf, *Leprieur, F. R. 1208?*, as *X. phyllocharis* (holotype PC 0086071 ex Leprieur herb.).

## Discussion

### Taxonomic approach adopted in the present study

The taxonomic approach followed in this study is based on the integration of detailed morphological analysis and phylogenetic inference. Material in good condition was selected for cultivation, and all collections and related type or authentic materials were subjected to thorough morphological characterization, allowing the assignment of known species names or the recognition of potentially undescribed taxa. These species hypotheses were then tested against multi-locus phylogenetic results obtained from cultures or occasionally from well-preserved specimens and were corroborated when both morphological and phylogenetic approaches proved to be largely congruent. This integrative framework proved effective in resolving species boundaries and in supporting the recognition of a substantial number of new species. It also highlights the continued importance of morphology when interpreted in conjunction with molecular data. This framework is consistent with the view of Wheeler (2004), in which species delimitation and classification are inherently hypothesis-driven processes, and each species represents a testable hypothesis about the distribution of characters. Taxonomic evaluations thus provide an efficient means of simultaneously testing multiple species hypotheses through comparative analysis.

### Recent *Xylaria* surveys from other regions

A comparable number of newly described species was reported by Li et al. (2024) in their study of *Xylaria* diversity in subtropical and tropical China, although their methodological approach is not always clearly articulated. In some cases, priority appears to have been given to phylogenetic data, while morphological characterization was often insufficiently detailed or precise to allow clear distinction between newly proposed taxa and previously described species. For example, *X*. *puerensis* Y.-P. Wu and Q.-R. Li possesses ostioles individually encircled by a disc, a feature characteristic of *X. papulis* C. G. Lloyd and its morphologically similar species such as *X. adscendens* and *X. mali*; however, it was discussed and compared with *X*. *curta* Fr. and *X*. *karyophthora*, two species lacking ostiolar discs, apparently because these three species clustered together in their phylogenetic analysis. On the other hand, nine of their newly described specie—*X. fanglanii* Y.-P. Wu and Q.-R. Li, *X. jinghongensis* Y.-P. Wu and Q.-R. Li, *X. liboensis* Y.-P. Wu and Q.-R. Li, *X. pseudoanisopleura* Y.-P. Wu and Q.-R. Li, *X. rhombostroma* Y.-P. Wu and Q.-R. Li, *X. serratifoliae* Y.-P. Wu and Q.-R. Li, *X. shishangensis* Y.-P. Wu and Q.-R. Li, *X. yaorenshanensis* Y.-P. Wu and Q.-R. Li, and *X. yinggelingensis* Y.-P. Wu and Q.-R. Li—differ only marginally from *X*. *haemorrhoidalis*, but the criteria used to delimit these species were not discussed or only briefly addressed. Notably, comparison of ITS sequences indicates that none of the species newly described herein from French Guiana correspond to those reported by Li et al. (2024), suggesting distinct regional assemblages despite similar levels of diversity.

Vandegrift et al. (2023), in a study of fungal diversity in a cloud forest in Ecuador, acknowledged the difficulty of morphological identification in *Xylaria*, noting that the expertise of “alphataxonomists” is required for “precise identifications, descriptions of novel taxa, nomenclatural considerations, inferring evolutionary relationships, identifying target taxa for additional sequencing, etc.” To address this difficulty, they adopted an approach combining high-quality digital photography with ITS barcode sequencing, with data made publicly available through repositories such as GenBank and iNaturalist. While this strategy facilitates rapid biodiversity assessment, it also has inherent limitations. In particular, photographs often depict immature stromata that lack diagnostic features and generally fail to capture critical microscopic characters. As a result, identifications based solely on such data remain provisional and depend on subsequent examination by experienced taxonomists. An example illustrating the limitation is the identification of *X. fissilis* (= *X. cylindrica*) by Vandegrift et al. (2023). As discussed under *X*. *piloides*, pairwise alignment of ITS sequences indicates that Ecuadorian material identified as *X. fissilis* shares only 87.63% similarity with verified material from the French West Indies but 99.4% similarity with *X. piloides*.

A more recent study by Cruz et al. (2026), also based on Ecuadorian material, adopts a taxonomic approach closer to that of Li et al. (2024). However, the resulting taxonomic concepts are not always convincing, reflecting difficulties in accurately assessing morphological features. For instance, *X. aenea* reported in that study has ascospore morphology consistent with the species concept provided by Ju and Hsieh (2014), but its stromata are narrower and possess a roughened surface, rather than the broader stromata with a fairly plane surface and prominent wrinkles described by Ju and Hsieh (2014). The ITS sequence (OR088132) of *X*. *aenea* in Cruz et al. (2026) shows 97.8% similarity to the Venezuelan material listed in Table 1. Taken together, the Ecuadorian collection, although closely related to *X*. *aenea*, differs in both stromatal morphology and ITS sequence and therefore warrants re-examination.

### Limitations and perspectives of the present study

Several potential limitations of the present study should be considered. First, a relatively high proportion of newly described species are based on single collections. This situation is common in tropical taxonomic surveys, where many taxa appear to be rare or are only infrequently encountered. Nevertheless, it highlights the need for additional collections to better assess intraspecific variability. Future progress in the taxonomy of *Xylaria* in tropical regions would benefit from the involvement of resident mycologists, allowing for more extensive and repeated sampling, as well as more precise ecological observations. However, such expertise remains scarce.

Second, information on host substrates remains incomplete for many taxa, as is often the case in tropical studies. In particular, stromata fruiting on dead woody substrates on the ground are often difficult to associate with their host plants. Improved knowledge on host associations would require closer collaboration with local botanists capable of accurately identifying host plants.

Cultures have been obtained from many of the studied *Xylaria* species and will be included in a future publication. Incorporating these data into the present study would substantially increase the length of this already extensive paper. Here, cultures and anamorphic features are mentioned only briefly in cases where they contribute to species delimitation.

## Conclusion

The present study demonstrates a remarkably high *Xylaria* diversity in French Guiana, revealed through the integration of detailed morphological observations and molecular phylogenetic analyses. This combined approach uncovers a substantially greater level of diversity than previously recognized and a high proportion of newly described species. Several species complexes, including those related to *X*. *cantareirensis* and *X*. *scruposa*, are shown to involve significant taxonomic uncertainty, highlighting the need for further revision. Our findings emphasize the importance of continued sampling, particularly of fresh material suitable for culturing, sequencing. They also underscore the significance of French Guiana as a key region for advancing our understanding of the taxonomy, evolution, and biogeography of *Xylaria* in the Neotropics. Despite advances in molecular systematics, robust taxonomy in *Xylaria* still depends on the integration of careful morphological observation with phylogenetic data. Approaches that rely heavily on one of these two components risk generating unstable or poorly supported taxonomic frameworks. By adopting an integrative approach, the present study contributes to a more balanced and reproducible understanding of species diversity within this complex genus.

## Electronic supplementary material

Below is the link to the electronic supplementary material.

Supplementary Material 1: Additional file 1: Aligned concatenated dataset of α-ACT, RPB2, and β-TUB (RPB2-TUB-ACT dataset).

Supplementary Material 2: Additional file 2: The complete ML phylogenetic tree.

## Data Availability

Specimens have been deposited at the HAST herbarium, and cultures are available at BCRC. DNA sequences have been deposited at GenBank. Newly described species and new type designations were registered at MycoBank.
